# Semiquantitative proteomic analysis of human hippocampal tissues from Alzheimer’s disease and age-matched control brains

**DOI:** 10.1186/1559-0275-10-5

**Published:** 2013-05-01

**Authors:** Ilijana Begcevic, Hari Kosanam, Eduardo Martínez-Morillo, Apostolos Dimitromanolakis, Phedias Diamandis, Uros Kuzmanov, Lili-Naz Hazrati, Eleftherios P Diamandis

**Affiliations:** 1 Department of Laboratory Medicine and Pathobiology, University of Toronto, Toronto, ON, Canada; 2 Samuel Lunenfeld Research Institute, Department of Pathology and Laboratory Medicine, Mount Sinai Hospital, Toronto, ON, Canada; 3 Tanz Centre for Research in Neurodegenerative Diseases, University of Toronto, Toronto, ON, Canada; 4 Department of Clinical Biochemistry, University Health Network, Toronto, ON, Canada; 5 Department of Pathology and Laboratory Medicine, Mount Sinai Hospital, 60 Murray St., Box 32, Floor 6, Rm. L6-201, Toronto, ON, M5T 3L9, Canada

**Keywords:** Alzheimer’s disease, Cerebrospinal fluid, Hippocampus, Human brain, Mass spectrometry

## Abstract

**Background:**

Alzheimer’s disease (AD) is the most common type of dementia affecting people over 65 years of age. The hallmarks of AD are the extracellular deposits known as amyloid β plaques and the intracellular neurofibrillary tangles, both of which are the principal players involved in synaptic loss and neuronal cell death. Tau protein and Aβ fragment 1–42 have been investigated so far in cerebrospinal fluid as a potential AD biomarkers. However, an urgent need to identify novel biomarkers which will capture disease in the early stages and with better specificity remains. High-throughput proteomic and pathway analysis of hippocampal tissue provides a valuable source of disease-related proteins and biomarker candidates, since it represents one of the earliest affected brain regions in AD.

**Results:**

In this study 2954 proteins were identified (with at least 2 peptides for 1203 proteins) from both control and AD brain tissues. Overall, 204 proteins were exclusively detected in AD and 600 proteins in control samples. Comparing AD and control exclusive proteins with cerebrospinal fluid (CSF) literature-based proteome, 40 out of 204 AD related proteins and 106 out of 600 control related proteins were also present in CSF. As most of these proteins were extracellular/secretory origin, we consider them as a potential source of candidate biomarkers that need to be further studied and verified in CSF samples.

**Conclusions:**

Our semiquantitative proteomic analysis provides one of the largest human hippocampal proteome databases. The lists of AD and control related proteins represent a panel of proteins potentially involved in AD pathogenesis and could also serve as prospective AD diagnostic biomarkers.

## Background

Alzheimer’s disease (AD) is a progressive neurodegenerative disease mainly affecting people over the age 65. The hallmarks of AD are the extracellular deposits known as amyloid β (Aβ) plaques and the intracellular neurofibrillary tangles (NFT), the principal players thought to be involved in synaptic loss and neuronal cell death [1,2]. Currently, diagnosis of AD is based on clinical criteria that are relied on neuropsychological examination, mental status testing and insight into the medical history of the patients. However, still, the gold standard for AD diagnosis remains histological examination of post mortem brain regions. Furthermore, there are no accurate methods to track the efficacy of new therapies. Hence, there is a desperate need for specific biomarkers that proactively identify evolving cases of AD and may lend way to more favorable medical outcomes [3]. Cerebrospinal fluid (CSF) has been so far the most promising source of potential protein biomarkers. CSF amyloid β 1–42 fragment (Aβ 1–42) has shown about 50% decrease in AD patients in comparison to cognitively normal individuals [4,5], however it is not consistent in distinguishing AD from other forms of dementia [6]. Other prospective candidates, total tau (T-tau) and phosphorylated tau (P-tau) levels have been found increased in CSF AD cases compared to controls [7]. Although T-tau levels have a trend to be elevated in other neurodegenerative diseases as well [8], indicating the lack of specificity, P-tau levels may discriminate AD from other types of dementias [9,10]. The combination of these three biomarkers represents markers for Aβ depositions as well as neuronal injury and have confirmed good diagnostic accuracy in early AD by multicenter studies in CSF [10]. In addition, measurement of Aβ 1–42, T-tau and P-tau levels in CSF are included in the diagnostic criteria for diagnosis of mild cognitive impairment due to AD [11]. Human brain tissue proteomics have been studied gradually in the last decade [12-14]. A recent proteomic study with mass spectrometry analysis has demonstrated a total of 197 proteins differentially abundant in AD versus controls, after examining the temporal lobe region [15], whereas in another study 18 proteins were identified in hippocampus region with altered protein level that are involved in different cellular functions in AD pathology [16]. Together with temporal lobe, hippocampus is one of the earliest affected regions in AD pathology, when memory and cognitive functions are already impaired [17,18]. Therefore, proteomic analysis of AD hippocampus, combined with pathway analysis, could help in defining the etiology of the disease as well as identify potential biomarkers and therapeutic targets. We present here one of the first comprehensive proteomic analyses of the hippocampal region of three brains affected by AD and three age-matched controls.

## Results and discussion

**Figure 1:**
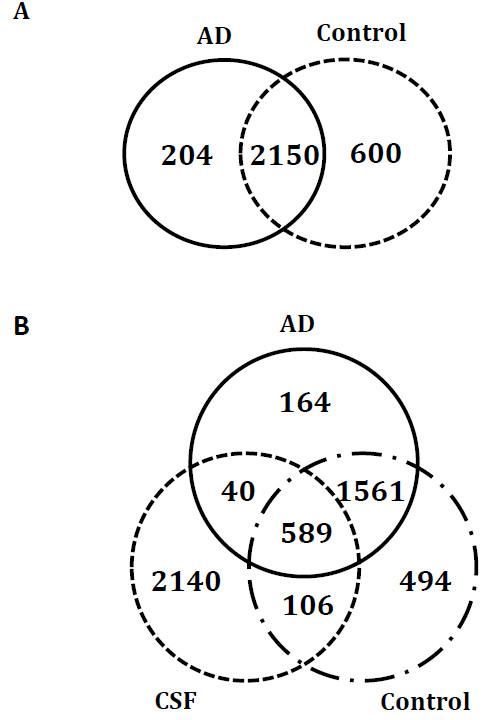
**Overlap of the identified proteins in Alzheimer’s disease (AD), control samples and CSF proteome.** (**A**) Overlap of the proteins identified in post-mortem hippocampal tissue specimens from AD patients and age-matched healthy controls. AD and control pools (n = 3) were analysed in triplicates. (**B**) Overlap of AD and control proteins with literature-based CSF proteome [23]. The comparison reveals that 40 AD-specific proteins identified in the current study were also present in the CSF database.

From the proteomic analysis of 6 hippocampal tissue specimens (pool of 3 AD and pool of 3 controls), 2954 proteins were identified, with at least two peptides for 1203 of them. A total of 2354 proteins were detected in AD tissues and 2750 in control tissues, with 204 proteins exclusively detected in AD and 600 in controls (Figure 1A, Additional file 1). Furthermore, 1605 proteins were identified in all the three AD technical replicates, and 1755 proteins were identified in all the three control technical replicates. Of 204 AD-exclusive proteins, 124 were identified with ≥2 peptides. Two hundred fifty five proteins in 600 control-exclusive proteins were identified with ≥2 peptide hits. Analysis of technical and biological replicates is necessary to ensure the accuracy and biological significance of proteomic datasets. Pooling of biological replicates does not allow statistical comparisons, but produces sufficient sample to enhance the detection of low-abundance proteins through technical replicates and helps to balance the between subject variability. In the current study, we pooled biological replicates and performed SCX fractionation of each pool in triplicate. A total of 120 fractions were analysed with approximately 300 hours of instrument time. An ideal proteomic study includes both biological and technical replicates. With this study, we intended to generate a comprehensive hippocampal proteome dataset and build a platform for future translational investigations in AD. Therefore, we pooled our biological samples to save precious instrument time but included technical triplicates to enhance confidence in protein identification and increase the depth of proteomic analysis. Despite the shortcomings of our pooling strategy, our study unveiled the largest hippocampal proteome database reported to date. We believe that the proteome presented here provides a valuable resource for researchers aiming to develop novel AD biomarkers and therapeutic targets. Post-mortem interval (PMI) is a critical factor affecting integrity of the proteome in post-mortem tissues. A short PMI (<2 h) is advantageous in proteomics studies of human tissues, since PMI-prone artifacts, such as proteolytic degradation, insolubility and oxidation/nitration of certain proteins, are minimized and results more likely represent the intrinsic situation in AD and control brains. However, due to scarcity of post-mortem tissues, it is not always possible to secure tissues with short PMIs. In the current study, 2 of 6 tissues have a PMI of < 4 h and the rest were collected at ~12 h PMI. A recent study using 2-dimenstional gel electrophoresis (2DE) showed that only a small percentage (6.5% of ~2500 proteins) of brain proteins underwent proteolytic degradation after 48 h PMI with no significant changes in their solubility [19]. Techniques such as 2DE and western blots could only capture intact proteins, leaving out proteolytic peptides, and this severely impacts the quality of proteomic analysis. On the other hand, sample preparation methods used in the current study, e.g. strong cation exchange chromatography, will ensure the capture of total proteome that includes proteolytically degraded peptides as well as trypsin-generated peptides. In addition, we also avoided the dialysis procedure to prevent loss of low molecular weight proteolytic peptides. Therefore, the overall effect of proteolytic degradation during PMI is minimized.

It should be noted that when the same amount of total protein was processed for proteomic analysis; more proteins were identified in controls, compared to AD tissues. Lower number of protein identifications in AD tissues may be attributed to inherent insolubility of protein aggregates which renders them inaccessible to trypsin proteolytic activity. To the best of our knowledge, the current report presents the largest hippocampal proteome dataset published to date. Moesin (MSN), heat shock protein beta-1 (HSPB1), S100B protein (S100B), and chloride intracellular channel protein 1 (CLIC1) were the top four over-expressed proteins (≥ 4.5-fold measured in terms of spectral counts) in AD tissues. Elongation factor 1-alpha 2 (EEF1A2), 2-oxoglutarate dehydrogenase (OGDH), isoform 1 of immunoglobulin superfamily member 8 (IGSF8) and actin-related protein 2 (ACTR2) are highly down-regulated proteins (≥ 4.5-fold) in AD tissues. Top 150 highly over-expressed and under-expressed protein fold changes are presented as additional information.

**Figure 2:**
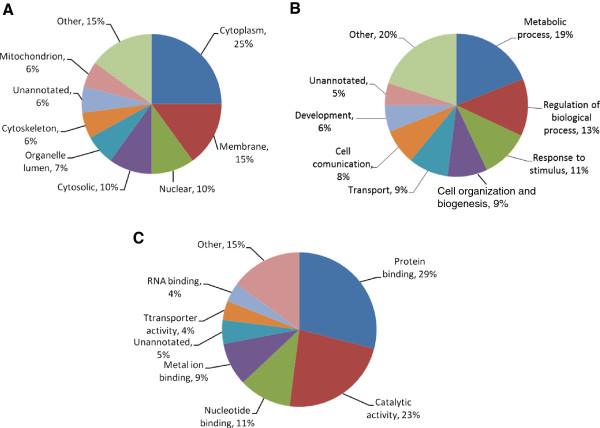
**Gene Ontology (GO) analysis.** Diagram showing cellular localization (**A**), biological processes (**B**) and molecular mechanism (**C**) for AD hippocampal proteome. Gene Ontology information was retrieved from ‘Protein center’ database.

**Figure 3:**
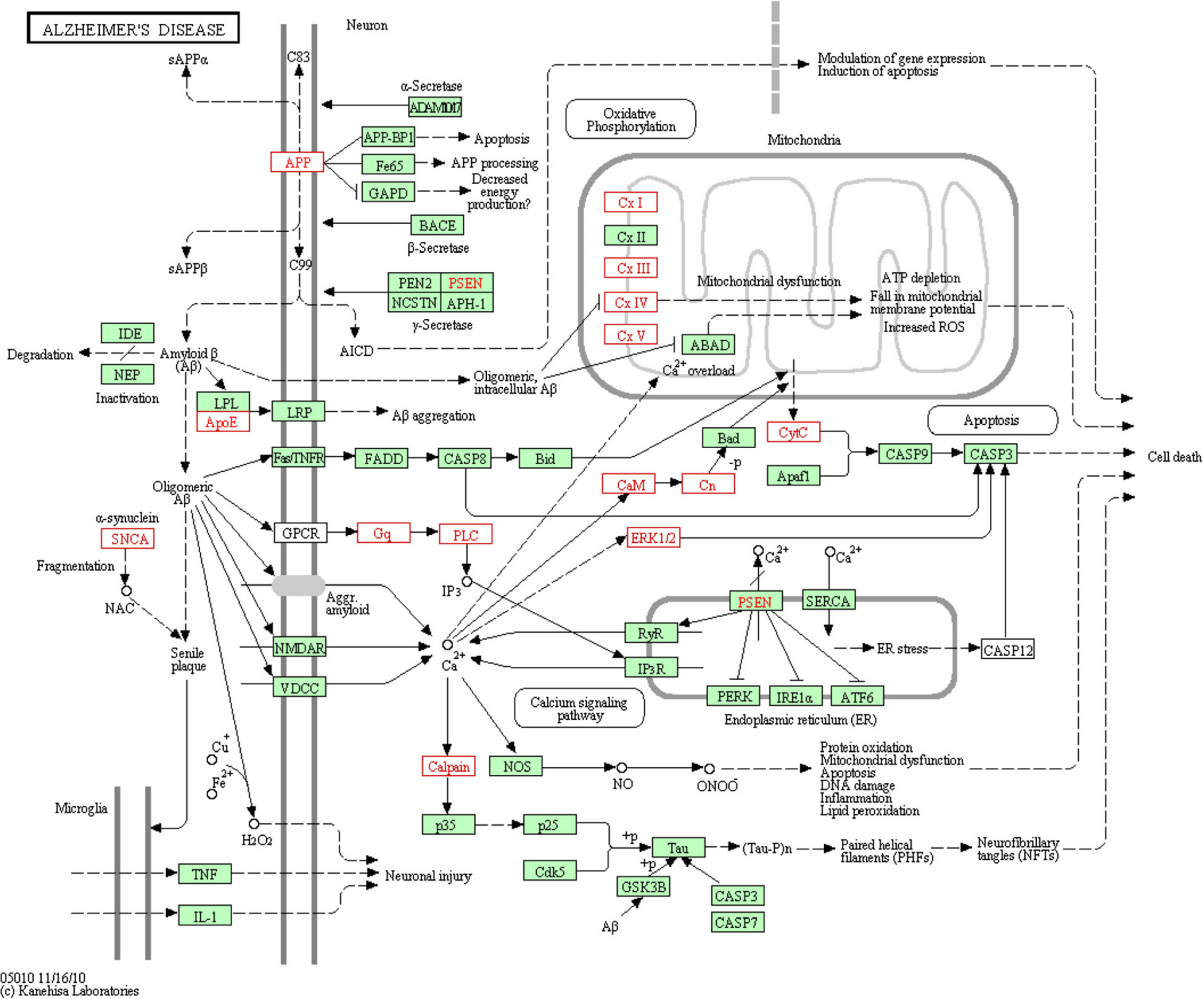
**Alzheimer’s disease pathway from the KEGG database.** AD pathway (hsa05010) presents the up-regulated (marked in red) and down regulated (marked in green) proteins identified in the current proteomic study. Proteins involved in beta-amyloid aggregation, calcium signalling pathway and mitochondrial dysfunction are identified as significantly up-regulated by spectral counting methods.

We relied upon “Protein Center” (Thermo Fisher Scientific, USA) to retrieve Gene Ontology information for cellular localization, biological processes and molecular function of AD proteins. As expected, majority of identified proteins were of membranous and cytoplasmic origin and were associated with metabolic processes, protein binding and catalytic functions (Figure 2). Proteins identified in AD pool were compared against the human proteome database to identify statistically significant over-represented gene ontology functions using BinGO (Cytoscape plugin) enrichment map. A p-value threshold of <0.001 and a false discovery rate of FDR < 5% were used to confidently predict enriched GO terms among AD proteins. Protein binding (p = 3.28E-82), catalytic activity (p = 8.72E-37), oxido-reductase activity (p = 7.78E-25), adenyl nucleotide binding (p = 1.10E-9), SNARE binding (4.56E-8) and syntaxin binding (1.5E-6) were the most significantly enriched molecular functions of AD proteins. Among the biological processes, cellular metabolic processes (p = 1.05E-42), primary metabolic process (p = 8.21E-23), vesicle mediated transport (p = 1.16E-23), cellular ketone metabolic process (p = 3.08E-21), oxidative phosphorylation (1.01E-17) and positive regulation of ubiquitin activity (p = 3.15E-9) were of high statistical significance. We employed “Protein center” pathway analysis tools to explore the plausible pathological relevance of differential expression (calculated by spectral counting) of hippocampal proteins between AD and Control groups. A total of 55 canonical pathways were identified as over-represented due to this differential expression. Parkinson’s disease (p = 9.10E-10), Alzheimer’s disease (p = 2.07E-7), synaptic vesicle cycle (p = 0.001) and long term depression (p = 0.01) were of considerable relevance to AD etiology. Figure 3 presents KEGG AD pathway (hsa05010) [20] with the up-regulated proteins marked in red and down-regulated marked in green. Fifty four of 157 proteins involved in this pathway were detected in this study (see Additional file 2). These proteins were associated with critical pathological aspects of AD; proteolytic processing of amyloid precursor protein (APP), down-regulation of oxidative phosphorylation, mitochondrial dysfunction, calcium dysfunction and Aβ aggregation. Recent research suggests that dysregulation of calcium homeostasis in aged brains aberrantly activates calpains (cysteine proteases), which, in turn, initiate the proteolytic degradation of key neuronal proteins, resulting in poor synaptic transmission and memory loss [21,22]. Additionally, calpains mediate the activation of extracellular signal-regulated kinase ½ (ERK1/2), which induces hyper phosphorylation of cytoskeletal proteins, including tau, triggering a cascade of cellular events leading to the self-assembly of NFT. The overexpression of calpains (CAPN1 and 2) and ERK1/2 in the current study coincides with the aforementioned literature findings [22].

The underlying objective of the current study was to segregate promising candidate biomarkers from the list of 2954 proteins for future verification studies. To this end, we considered the 204 and 600 proteins that were identified exclusive to AD tissues and control tissues, respectively. The failure to detect these proteins does not endorse their absence; however, it does imply that these proteins are differentially expressed. Mere differential expression of a protein in AD tissues does not qualify the protein to be a biomarker, unless its disease-specific higher expression in tissues is reflected in easily accessible bio-fluids such as CSF or serum. In this light, we compared the current hippocampal proteome with literature-based CSF proteome [23]; 25% of 2954 tissue proteins were present in CSF (see Additional file 1). A considerable finding is that 40 of the 204 AD-exclusive proteins and 106 of 600 control-exclusive proteins were also detected in CSF (Figure 1B). Secretory origin is one of the most important qualifications of biomarker candidate [24]. It is well-established that majority of CSF and serum proteins are of extracellular and secretory origin. Therefore, we assume that extracellular and secreted proteins, identified in CSF and up/down-regulated in AD tissues are favourable candidates for biomarker verification. As most of the 40 and 106 proteins that are present in CSF proteome were either extracellular/secretory or membranous origin, therefore, it is worthwhile to include these proteins as a potential source of candidate biomarkers that need to be further studied and verified in CSF samples (please see Additional files 3 and 4). Please see Additional files 5 and 6 for differentially-expressed proteins in AD and Control tissue pools.

## Conclusion

Hippocampus is one of the primary regions of the brain affected by Alzheimer’s disease. This structure is known to host tangles and plaques in the earliest phases of the disease cascade, even before the appearance of clinical symptoms. The proteome of such a pivotal region represent a promising source of diagnostic markers and molecular targets for therapeutic intervention. Herein, we performed proteomic analysis of freshly-frozen post-mortem hippocampal tissue sections from Alzheimer’s patients (n = 3) and age-matched controls (n = 3). Our detailed proteomic analysis utilizing offline multidimensional chromatography coupled with the LTQ-Orbitrap XL mass spectrometer and semiquantitative spectral counting methods identified 2954 proteins, one of the largest human hippocampal proteome database published to date. We applied a hypothesis-driven set of filtering criteria, based on protein’s cellular origin and identification in the cerebrospinal fluid proteome to find proteins that can be used as potential biomarkers in cerebrospinal fluid.

## Methods

Post-mortem frozen brain hippocampal tissues were obtained with Research Ethics Board approval from the University Health Network, Toronto, Canada. Three pathologically confirmed AD tissues (all three had Braak stage 6/6) were obtained from three female patients (aged 69, 75 and 98 years) with PMI of 13, 4 and 19.5 hours, respectively, while three control tissues were obtained from one female (aged 77 years) and two male patients (aged 78 and 80 years) with PMI of 12, 12 and 4 hours, respectively. Control patients were diagnosed with non-metastatic colon cancer, cardiovascular disease and heart failure, respectively. Prior to digestion, frozen tissue sections from both AD and controls were cut and weighted (~150 mg wet weight). Proteins from these six brain tissues were extracted and solubilized using 0.2% RapiGest (Waters Corporation, Milford, USA) in 50 mM ammonium bicarbonate. Briefly, tissue samples were homogenized (Polytron PT3100, Capitol Scientific, Austin, USA) at 15,000 rpm, for 15 s and sonicated on ice three times for 15 s with MISONIX immersion tip sonicator (Q SONICA LLC, CT, USA). The samples were centrifuged at 15,000 g at 4°C for 20 min; the supernatants were collected and measured for total protein content. Three AD tissues and three control tissues were pooled separately and an equal amount (3 mg) of protein from each pool was processed. Proteins were reduced and alkylated with 5 mM dithiothreitol and 15 mM iodoacetamide. To digest the proteins, sequencing grade trypsin (Promega, WI, USA) was added, at an enzyme to substrate ratio of 1:50 and the digestion was carried out at 37°C for 18 hours. Fractionation of acidified tryptic-peptides was performed on a PolySULFOETHYL aspartamide strong cation exchange (SCX) column (2.1 mmID × 200 mm; 5 μ; 200 °A; The Nest Group, Inc., MA, USA) connected to an Agilent 1100 HPLC system. SCX fractionation was performed in triplicate for AD and control pools, and 20 fractions were collected per chromatographic run. This amounted to a total of 120 SCX fractions, which were then subjected to LC-MS/MS analysis after a brief desalting procedure. A 60 min linear gradient method was operated with buffer A → B (Buffer A: 0.26 M formic acid (FA) in 5% acetonitrile, B: 0.26 M FA in 5% acetonitrile and 1 M ammonium formate) at a flow rate of 250 μL/min. SCX fractionation was performed in triplicate for AD and control pools. The peptides from SCX fractions were desalted and injected onto a nano-LC system (Proxeon Biosystems, Odense, Denmark) connected online to LTQ-Orbitrap XL mass spectrometer (Thermo Fisher Scientific, San Jose, CA, USA). A 90 min linear gradient reverse-phase chromatography (Buffer A: 0.1% FA in water and B: 0.1% FA in acetonitrile) at a flow rate of 400 nL/min was performed to resolve peptides on a C18 column (75 μM × 5 cm). The mass spectra were acquired in data-dependent mode. The MS spectra were searched against the non-redundant IPI human database (version 3.71 containing both forward and reverse protein sequences) using two search engines, separately: Mascot, version 2.1.03 (Matrix Science) and the Global Proteome Machine manager, version 2006.06.01. The following parameters were used: (I) enzyme: trypsin; (II) one missed cleavage allowed; (III) fixed modification: carbamidomethylation of cysteines; (IV) variable modifications: oxidation of methionines; (V) MS^1^ tolerance, 7 ppm; and (VI) MS^2^ tolerance, 0.4 Da. The resulting Mascot DAT and X! Tandem XML files were merged using Scaffold® (version 2.06, Proteome Software Inc., Portland, Oregon) with ‘MudPIT’ (multidimensional protein identification technology) option checked. Scaffold data was filtered using the X! Tandem Log E (min 3.0) and Mascot ion-score filters [ion score 15, 30 (+2) and 40 (+3)] in order to obtain a protein false-positive rate (FPR) of  ≤ 1%. FPR = 2 × (number of proteins identified by searching the reverse sequences)/(the total number of identified proteins). Scaffold® protXML reports were exported and uploaded into Protein Center (Proxeon Biosystems, Odense, Denmark) to create Venn diagrams.

The proteomic data associated with this manuscript may be downloaded from ProteomeCommons.org Tranche using the following hash Khn5Yg/CHsUFAZFaObXXCrT75bIRXHdWuJLgEDPWwgT + A5+/62Ijmc4Y/jhNS1GTXxORV7gfkaIbskPpU6RCbwIDDF4AAAAAAAADIg==Encrypt passcode: ecPC48nIVr0iD6OzSDSa

The data can be viewed with Scaffold (ver. 2.6) viewer, a freeware available on http://www.proteomesoftware.com/Scaffold/Scaffold_viewer.htm.

## Abbreviations

AD: Alzheimer’s disease; Aβ: Amyloid β; NFT: Neurofibrillary tangles; CSF: Cerebrospinal fluid.

## Competing interests

The authors declare that they have no competing interests.

## Authors’ contributions

IB, HK and EMM participated in the study design, acquisition of mass spectrometry data, analysis and interpretation of proteomic data. AD and UK performed bioinformatics and pathway analysis of proteomic data. PD, LNZ and EPD contributed to the study design, evaluation of data and revision of the manuscript. All authors read and approved the final manuscript.

## Supplementary Material

Additional file 1 Comparison of hippocampal proteome data with literature-compiled CSF proteome. Click here for file

Additional file 2 List of over-represented pathways predicted by Protein Center Pathway analysis. Click here for file

Additional file 3 List of 40 CSF proteins that were found exclusively in Alzheimer's hippocampal tissues. Click here for file

Additional file 4 One hundred six CSF proteins that were identified exclusively in Control tissue pools. Click here for file

Additional file 5 Up-regulated proteins in Alzheimer's (AD) tissues in comparison to Control tissues. Click here for file

Additional file 6 Up-regulated proteins in ‘Control’ tissues in comparison to Alzheimer's (AD) tissues. Click here for file
