# Supplementary material for: Semiquantitative proteomic analysis of human hippocampal tissues from Alzheimer’s disease and age-matched control brains
Source: Clin Proteomics. 2013 May 1;10(1):5. doi: 10.1186/1559-0275-10-5 (PMC3648498; doi:10.1186/1559-0275-10-5)
Supplement: Additional file 1 — Comparison of hippocampal proteome data with literature-compiled CSF proteome. [file 1559-0275-10-5-S1.pdf]

Additional table 1: Comparison of hippocampal proteome data with literature-compiled CSF proteome.

IPI	GENE	Alzheimer's Hippocampus	Control hippocampus	CSF	Cellular localization	Biological process	Molecular function	Protein Description
IPI00181079.3	METRNL			X	extracellular			Meteorin-like protein
IPI00166002.5	PSD3	X	X			regulation of biological process,response to stimulus,cell communication	protein binding,enzyme regulator activity	Isoform 3 of PH and SEC7 domain-containing protein 3
IPI00219025.3	GLRX	X	X	X	cytoplasm,cytosol	metabolic process,transport,regulation of biological process,cellular homeostasis	protein binding,catalytic activity	Glutaredoxin-1
IPI00025840.2	EFNA1			X	extracellular,membrane	development,cell organization and biogenesis,metabolic process,regulation of biological process,response to stimulus,cellular component movement,cell communication,cell differentiation	protein binding	Isoform 1 of Ephrin-A1
IPI00013749.3	PRKCZ			X	membrane,cytoplasm,organelle lumen,nucleus,endosome	cell death,cell organization and biogenesis,development,metabolic process,transport,regulation of biological process,response to stimulus,cell communication,defense response,cellular homeostasis,cell differentiation,coagulation	protein binding,metal ion binding,nucleotide binding,catalytic activity	Protein kinase C zeta type
IPI00744835.1	PSAP			X		metabolic process		Isoform Sap-mu-9 of Proactivator polypeptide
IPI00102685.1	MYADM	X	X		membrane			Myeloid-associated differentiation marker
IPI00292657.3	PTGR1	X	X	X	cytoplasm	metabolic process,response to stimulus	metal ion binding,nucleotide binding,catalytic activity	Prostaglandin reductase 1
IPI00219673.6	GSTK1	X	X		cytoplasm		antioxidant activity,protein binding,catalytic activity	Isoform 1 of Glutathione S-transferase kappa 1
IPI00300020.5	SLC1A2	X	X	X	membrane	development,transport,regulation of biological process,response to stimulus,cell communication	transporter activity	Isoform 1 of Excitatory amino acid transporter 2
IPI00044743.9	TMEM132B		X	X	membrane			Isoform 1 of Transmembrane protein 132B
IPI00021338.2	DLAT	X	X		mitochondrion,cytoplasm,organelle lumen	metabolic process,regulation of biological process,response to stimulus	protein binding,catalytic activity	Dihydrolipoyllysine-residue acetyltransferase component of pyruvate dehydrogenase complex, mitochondrial
IPI00020319.2	BLOC1S1		X		mitochondrion,membrane,cytoplasm,vacuole,organelle lumen,cytosol	cell organization and biogenesis,metabolic process,transport	protein binding	Isoform 1 of Biogenesis of lysosome-related organelles complex 1 subunit 1
IPI00029629.4	TRIM25	X	X		cytoskeleton,cytoplasm,organelle lumen,cytosol,nucleus	metabolic process,regulation of biological process,response to stimulus,defense response	protein binding,metal ion binding,motor activity,catalytic activity	E3 ubiquitin/ISG15 ligase TRIM25
IPI00009794.2	SDF4			X				Isoform 4 of 45 kDa calcium-binding protein
IPI00643286.4	SDR39U1		X			metabolic process	catalytic activity	Isoform 2 of Epimerase family protein SDR39U1
IPI00220666.3	SCN2A	X	X		membrane	transport	transporter activity	Isoform 2 of Sodium channel protein type 2 subunit alpha
IPI00004367.3	FXYD6	X	X	X	membrane	transport	transporter activity	FXYD domain-containing ion transport regulator 6
IPI00399180.3	SBK1			X	cytoplasm	development,metabolic process	nucleotide binding,catalytic activity	Serine/threonine-protein kinase SBK1
IPI00033573.4	RUFY3	X	X		membrane,cytoplasm	cell organization and biogenesis,development,regulation of biological process,cell differentiation	protein binding	Isoform 1 of Protein RUFY3
IPI00289334.2	FLNB	X	X	X	cytoskeleton,membrane,cytoplasm,nucleus,cytosol	development,cell organization and biogenesis,regulation of biological process,response to stimulus,cell communication,cell differentiation	protein binding	Isoform 1 of Filamin-B
IPI00816155.1	C1QL3			X				Isoform 2 of Complement C1q-like protein 3
IPI00175654.8	MILR1			X	membrane	transport,regulation of biological process,response to stimulus	protein binding	Isoform 1 of Allergin-1
IPI00296907.4	ACOX1	X	X		mitochondrion,membrane,cytoplasm,organelle lumen	metabolic process,reproduction	protein binding,catalytic activity	Isoform 1 of Peroxisomal acyl-coenzyme A oxidase 1
IPI00298281.4	LAMC1	X		X	extracellular,cytoskeleton,organelle lumen,chromosome,nucleus	cell proliferation,development,cell organization and biogenesis,regulation of biological process,response to stimulus,cellular component movement,cell differentiation,reproduction	protein binding,motor activity,structural molecule activity,catalytic activity	Laminin subunit gamma-1
IPI00220292.1	ART3			X		metabolic process	catalytic activity	Isoform 1 of Ecto-ADP-ribosyltransferase 3
IPI00032150.3	CDS2	X	X		mitochondrion,membrane,endoplasmic reticulum,cytoplasm	metabolic process	catalytic activity	Isoform 1 of Phosphatidate cytidylyltransferase 2
IPI00744561.1	IGHA1			X			protein binding	IGHA1 protein
IPI00293679.2	KCNQ4			X	membrane,cytoplasm	development,transport,regulation of biological process,cell communication	transporter activity	Isoform 1 of Potassium voltage-gated channel subfamily KQT member 4

IPI	GENE	Alzheimer's Hippocampus	Control hippocampus	CSF	Cellular localization	Biological process	Molecular function	Protein Description
IPI00304740.2	ITSN1	X	X		cytoskeleton,membrane,cytoplasm,cytosol	cell death,cell organization and biogenesis,metabolic process,transport,regulation of biological process,response to stimulus,cell communication	protein binding,metal ion binding,nucleotide binding,structural molecule activity,catalytic activity,enzyme regulator activity	Isoform 1 of Intersectin-1
IPI00741005.7	MGA			X	nucleus	metabolic process,regulation of biological process	DNA binding	MAX gene-associated protein isoform 2
IPI00008533.3	MMP17			X		metabolic process	metal ion binding,catalytic activity	MMP17 protein
IPI00852648.1	STXBP5L		X		membrane,cytoplasm	transport	protein binding	Isoform 1 of Syntaxin-binding protein 5-like
IPI00062419.2	SDSL	X			mitochondrion,cytoplasm	metabolic process	catalytic activity	Serine dehydratase-like
IPI00027770.1	SYP	X	X		membrane,cytoplasm	cell organization and biogenesis,transport,regulation of biological process,response to stimulus,cell communication	protein binding,transporter activity,metal ion binding	Synaptophysin
IPI00333068.4	ARPC1A	X	X		cytoskeleton,cytoplasm	cell organization and biogenesis,regulation of biological process	protein binding	Actin-related protein 2/3 complex subunit 1A
IPI00027448.3	ATP5L	X	X		membrane,mitochondrion,cytoplasm	metabolic process,transport	transporter activity,catalytic activity	ATP synthase subunit g, mitochondrial
IPI00292218.8	MST1			X				cDNA FLJ53076, highly similar to Hepatocyte growth factor-like protein
IPI00020356.4	MAP1A	X	X	X			catalytic activity	Uncharacterized protein
IPI00797310.1	CLSTN3			X				14 kDa protein
IPI00748891.2	FAM177A1			X			catalytic activity	Isoform 2 of Protein FAM177A1
IPI00013744.1	ITGA2	X	X		cell surface,membrane	development,cell organization and biogenesis,regulation of biological process,response to stimulus,cell communication,cell differentiation,coagulation	protein binding,receptor activity	Integrin alpha-2
IPI00217296.2	PPP2R4	X	X				enzyme regulator activity	Isoform 3 of Serine/threonine-protein phosphatase 2A activator
IPI00289965.4	KCNC3			X	membrane	cell death,cell organization and biogenesis,transport,regulation of biological process,cell communication	protein binding,transporter activity	Potassium voltage-gated channel subfamily C member 3
IPI00001568.1	ATP6V1D	X	X	X	membrane,cytoplasm,vacuole,cytosol	transport,regulation of biological process,response to stimulus,cell communication,cellular homeostasis	protein binding,transporter activity,catalytic activity	V-type proton ATPase subunit D
IPI00303797.3	BRAF		X		membrane,cytoplasm,cytosol,nucleus	cell death,development,metabolic process,regulation of biological process,response to stimulus,cell communication	protein binding,signal transducer activity,metal ion binding,nucleotide binding,catalytic activity	Serine/threonine-protein kinase B-raf
IPI00032830.2	REXO2			X	membrane,mitochondrion,cytoplasm,organelle lumen,nucleus	metabolic process	catalytic activity	Isoform 1 of Oligoribonuclease, mitochondrial (Fragment)
IPI00479669.1	UHRF1BP1L			X				Isoform 1 of UHRF1-binding protein 1-like
IPI00418741.6	FUK	X	X		cytoplasm	metabolic process	nucleotide binding,catalytic activity	Isoform 1 of L-fucose kinase
IPI00013624.4	TTPAL		X			transport	transporter activity	Alpha-tocopherol transfer protein-like
IPI00090720.4	QRSL1		X		mitochondrion,cytoplasm	cell organization and biogenesis,metabolic process	nucleotide binding,catalytic activity	Isoform 1 of Glutamyl-tRNA(Gln) amidotransferase subunit A homolog
IPI00006865.4	SEC22B		X		membrane,endoplasmic reticulum,cytoplasm,Golgi	transport	protein binding	Vesicle-trafficking protein SEC22b
IPI00030059.1	GNG10		X		membrane	metabolic process,regulation of biological process,response to stimulus,cell communication	signal transducer activity,catalytic activity	Guanine nucleotide-binding protein G(I)/G(S)/G(O) subunit gamma-10
IPI00004114.1	RNASE6			X	extracellular	metabolic process,response to stimulus,defense response	catalytic activity	Ribonuclease K6
IPI00155888.8	UBTFL3	X					protein binding,DNA binding	Putative upstream-binding factor 1-like protein 3/5
IPI00219110.7	NCS1	X	X		cytoskeleton,membrane,cytoplasm,Golgi,cytosol	cell organization and biogenesis,development,transport,regulation of biological process,response to stimulus,cell communication,cell differentiation	protein binding,metal ion binding	Neuronal calcium sensor 1
IPI00007765.5	HSPA9	X	X		cell surface,mitochondrion,cytoplasm,organelle lumen,nucleus	cell death,cell organization and biogenesis,metabolic process,transport,regulation of biological process	protein binding,nucleotide binding	Stress-70 protein, mitochondrial
IPI00014592.2	CHAD			X	extracellular	cell organization and biogenesis,regulation of biological process,cell growth	protein binding,structural molecule activity	Chondroadherin
IPI00005657.1	PFDN6	X	X		cytoplasm,cytosol	cell organization and biogenesis,metabolic process	protein binding	Prefoldin subunit 6
IPI00161549.3	SULT4A1	X	X		cytoplasm,cytosol	metabolic process,response to stimulus	catalytic activity	Isoform 1 of Sulfotransferase 4A1

IPI	GENE	Alzheimer's Hippocampus	Control hippocampus	CSF	Cellular localization	Biological process	Molecular function	Protein Description
IPI00024283.2	WDFY1		X		cytoplasm,cytosol,nucleus,endosome		protein binding,metal ion binding	WD repeat and FYVE domain-containing protein 1
IPI00298267.4	PTGS1	X	X			metabolic process,response to stimulus	antioxidant activity,protein binding,metal ion binding,catalytic activity	Cyclooxygenase 1b3
IPI00020012.2	APLP1	X	X	X	extracellular,membrane,cytoplasm,Golgi	cell death,development,transport,metabolic process,regulation of biological process,response to stimulus,cell communication	protein binding,metal ion binding	Isoform 1 of Amyloid-like protein 1
IPI00218637.7	HLA-DQB2			X	membrane	response to stimulus	protein binding	Uncharacterized protein
IPI00452731.6	NDUFA7	X	X		membrane,mitochondrion,cytoplasm	transport,metabolic process	catalytic activity	NADH dehydrogenase [ubiquinone] 1 alpha subcomplex subunit 7
IPI00022697.3	DRG2		X		membrane,mitochondrion,cytoplasm	transport,regulation of biological process,response to stimulus,cell communication	transporter activity,nucleotide binding	Developmentally-regulated GTP-binding protein 2
IPI00220710.5	ACOT9	X	X		mitochondrion,cytoplasm	metabolic process	catalytic activity	Isoform 1 of Acyl-coenzyme A thioesterase 9, mitochondrial
IPI00844498.2	CD99L2	X	X					CD99L2 protein
IPI00031718.1	ENTPD4			X	membrane,cytoplasm,Golgi,vacuole	metabolic process	catalytic activity	Isoform 1 of Ectonucleoside triphosphate diphosphohydrolase 4
IPI00554440.1	FCN2			X	extracellular	transport,metabolic process,regulation of biological process,response to stimulus,defense response,cell communication	protein binding,metal ion binding	ficolin-2 isoform a precursor
IPI00550720.4	C19orf57			X		development	protein binding	Isoform 1 of Uncharacterized protein C19orf57
IPI00219953.5	CMPK1	X	X			metabolic process	nucleotide binding,catalytic activity	UMP-CMP kinase isoform a
IPI00719505.2	RABL2A			X		regulation of biological process,response to stimulus,cell communication	nucleotide binding	Uncharacterized protein
IPI00431738.1	IL1RAPL1			X	cell surface,membrane,cytoplasm	cell organization and biogenesis,development,transport,regulation of biological process,response to stimulus,cell communication,defense response,cell differentiation	protein binding,signal transducer activity,receptor activity	Interleukin-1 receptor accessory protein-like 1
IPI00184821.1	COASY	X	X		membrane,mitochondrion,cytoplasm,organelle lumen,nucleus	metabolic process	nucleotide binding,catalytic activity	Isoform 1 of Bifunctional coenzyme A synthase
IPI00163483.3	BLOC1S2	X						Isoform 2 of Biogenesis of lysosome-related organelles complex 1 subunit 2
IPI00018843.1	DRD3			X	membrane,cytoplasm	cell death,cell proliferation,development,cell organization and biogenesis,metabolic process,transport,regulation of biological process,response to stimulus,defense response,cell communication,cellular homeostasis,cell differentiation	signal transducer activity,protein binding,receptor activity,catalytic activity	Isoform 1 of D(3) dopamine receptor
IPI00156171.3	ENPP2			X	extracellular,membrane	metabolic process,regulation of biological process,response to stimulus,cellular component movement,cell communication	protein binding,metal ion binding,receptor activity,catalytic activity	Isoform 1 of Ectonucleotide pyrophosphatase/phosphodiesterase family member 2
IPI00012912.1	CPT2	X	X		membrane,mitochondrion,cytoplasm,organelle lumen,nucleus	transport,metabolic process,regulation of biological process	catalytic activity	Carnitine O-palmitoyltransferase 2, mitochondrial
IPI00337385.3	PRPF40A			X	organelle lumen,nucleus	cell organization and biogenesis,cell division,metabolic process,regulation of biological process,cellular component movement	protein binding	Isoform 1 of Pre-mRNA-processing factor 40 homolog A
IPI00169383.3	PGK1	X	X	X	cytoplasm,cytosol	metabolic process	nucleotide binding,catalytic activity	Phosphoglycerate kinase 1
IPI00172656.6	FAF2	X	X		membrane,endoplasmic reticulum,cytoplasm	response to stimulus	protein binding	FAS-associated factor 2
IPI00815893.1	CHD2			X	nucleus	metabolic process,regulation of biological process	DNA binding,nucleotide binding,catalytic activity	Isoform 1 of Chromodomain-helicase-DNA-binding protein 2
IPI00032220.3	AGT	X	X	X	extracellular,cytoplasm	cell death,development,cell organization and biogenesis,metabolic process,regulation of biological process,response to stimulus,defense response,reproduction,cell proliferation,transport,cellular component movement,cell communication,cellular homeostasis,cell differentiation,cell growth	protein binding,enzyme regulator activity	Angiotensinogen
IPI00218693.8	APRT	X	X		cytoplasm,organelle lumen,nucleus,cytosol	development,metabolic process,transport,response to stimulus,reproduction	nucleotide binding,catalytic activity	Adenine phosphoribosyltransferase
IPI00479786.5	KHSRP	X	X		cytoplasm,organelle lumen,cytosol,nucleus	transport,metabolic process,regulation of biological process	DNA binding,RNA binding	Isoform 1 of Far upstream element-binding protein 2
IPI00216003.4	CUL5	X	X			metabolic process	protein binding	Cullin-5
IPI00384608.4	FAM123A		X		membrane	regulation of biological process,response to stimulus,cell communication	protein binding	Isoform 1 of Protein FAM123A

IPI	GENE	Alzheimer's Hippocampus	Control hippocampus	CSF	Cellular localization	Biological process	Molecular function	Protein Description
IPI00019581.2	F12			X	extracellular,membrane	metabolic process,regulation of biological process,response to stimulus,defense response,coagulation	protein binding,catalytic activity	Coagulation factor XII
IPI00299155.5	PSMA4	X	X		proteasome,cytoplasm,organelle lumen,nucleus,cytosol	cell death,metabolic process,regulation of biological process,response to stimulus,cell communication	protein binding,catalytic activity	Proteasome subunit alpha type-4
IPI00295832.1	OMG	X	X	X	membrane	development,cell organization and biogenesis,regulation of biological process,response to stimulus,cell communication,cell differentiation,cell growth	protein binding	Oligodendrocyte-myelin glycoprotein
IPI00444371.3	WDR44	X	X		cytoskeleton,membrane,cytoplasm,Golgi,cytosol,endosome	regulation of biological process,cellular component movement	protein binding	Isoform 1 of WD repeat-containing protein 44
IPI00003971.1	RTN1	X	X	X	membrane,endoplasmic reticulum,cytoplasm	development,regulation of biological process,response to stimulus,cell communication,cell differentiation	signal transducer activity,protein binding	Isoform RTN1-A of Reticulon-1
IPI00182938.8	AHCYL1	X	X		endoplasmic reticulum,cytoplasm	metabolic process,transport,regulation of biological process	catalytic activity	Isoform 1 of Putative adenosylhomocysteinase 2
IPI00104074.5	CD163			X	extracellular,membrane	response to stimulus,defense response	protein binding,receptor activity	Isoform 1 of Scavenger receptor cysteine-rich type 1 protein M130
IPI00015973.1	EPB41L2	X	X	X	cytoskeleton,membrane,cytoplasm,organelle lumen,nucleus	cell organization and biogenesis	protein binding,structural molecule activity	Band 4.1-like protein 2
IPI00008575.3	KHDRBS1	X	X		membrane,nucleus	cell proliferation,transport,metabolic process,regulation of biological process,response to stimulus,cell communication	protein binding,DNA binding,RNA binding	Isoform 1 of KH domain-containing, RNA-binding, signal transduction-associated protein 1
IPI00940148.1	GDI2	X	X		cell surface,membrane,cytoplasm,Golgi,cytosol	metabolic process,transport,regulation of biological process,response to stimulus,cell communication	protein binding,enzyme regulator activity	Rab GDP dissociation inhibitor beta
IPI00168479.3	APOA1BP	X	X	X	membrane			cDNA FLJ56357, highly similar to Homo sapiens apolipoprotein A-I binding protein (APOA1BP), mRNA
IPI00025155.1	FSTL3			X	extracellular,cytoplasm,nucleus	development,metabolic process,regulation of biological process,response to stimulus,cell communication,cell differentiation,reproduction	protein binding	Isoform 1 of Follistatin-related protein 3
IPI00005615.1	TIAL1	X			cytoplasm,vacuole,nucleus	cell death,metabolic process,regulation of biological process,response to stimulus,defense response	RNA binding,nucleotide binding	Nucleolysin TIAR
IPI00219798.2	ROBO1		X	X	membrane		protein binding	Uncharacterized protein
IPI00167088.3	NDUFAF6		X			metabolic process	catalytic activity	chromosome 8 open reading frame 38, isoform CRA_d
IPI00148063.1	HEBP1	X	X		extracellular,mitochondrion,cytoplasm,cytosol		metal ion binding	Heme-binding protein 1
IPI00149097.2	SEMA4A			X	membrane	cell organization and biogenesis,development,regulation of biological process,response to stimulus,cellular component movement,cell communication,cell differentiation	protein binding,receptor activity	Semaphorin-4A
IPI00334532.3	L1CAM	X	X	X			protein binding	Isoform 2 of Neural cell adhesion molecule L1
IPI00220487.4	ATP5H	X	X		mitochondrion,membrane,cytoplasm	transport,metabolic process	transporter activity,catalytic activity	Isoform 1 of ATP synthase subunit d, mitochondrial
IPI00220402.3	PPP1R2	X	X			metabolic process,regulation of biological process,response to stimulus,cell communication	protein binding,enzyme regulator activity	Protein phosphatase inhibitor 2
IPI00741608.1	EIF5AP4			X	cytoplasm,ribosome	cell organization and biogenesis,metabolic process,regulation of biological process	RNA binding,structural molecule activity	similar to eukaryotic translation initiation factor 5A
IPI00299086.3	SDCBP	X	X	X	cytoskeleton,membrane,endoplasmic reticulum,cytoplasm,cytosol,nucleus	development,cell organization and biogenesis,transport,metabolic process,regulation of biological process,response to stimulus,cellular component movement,cell communication,cell differentiation	protein binding	Isoform 1 of Syntenin-1
IPI00007752.1	TUBB4B	X	X	X	cytoskeleton,cytoplasm,cytosol	cell death,cell organization and biogenesis,metabolic process,regulation of biological process,response to stimulus,cellular component movement,defense response	protein binding,nucleotide binding,structural molecule activity,catalytic activity	Tubulin beta-2C chain
IPI00464952.2	SRSF11		X		organelle lumen,nucleus	transport,metabolic process	protein binding,RNA binding,nucleotide binding	Serine/arginine-rich splicing factor 11
IPI00002816.1	CTSF			X	cytoplasm,vacuole	metabolic process	catalytic activity	Cathepsin F
IPI00296534.2	FBLN1	X	X	X	extracellular	cell organization and biogenesis,development,metabolic process,regulation of biological process,reproduction	protein binding,metal ion binding,structural molecule activity,enzyme regulator activity	Isoform D of Fibulin-1
IPI00023601.1	HAPLN1	X	X		extracellular		protein binding	Hyaluronan and proteoglycan link protein 1
IPI00002519.1	SHMT1	X			mitochondrion,cytoplasm,cytosol,nucleus	cell organization and biogenesis,metabolic process	protein binding,catalytic activity	Isoform 1 of Serine hydroxymethyltransferase, cytosolic
IPI00552905.3	PRRT3			X	membrane			Isoform 1 of Proline-rich transmembrane protein 3

IPI	GENE	Alzheimer's Hippocampus	Control hippocampus	CSF	Cellular localization	Biological process	Molecular function	Protein Description
IPI00171438.2	TXNDC5	X		X	endoplasmic reticulum,cytoplasm,organelle lumen,vacuole	cell death,cell organization and biogenesis,transport,metabolic process,regulation of biological process,cellular homeostasis	catalytic activity	Thioredoxin domain-containing protein 5
IPI00554488.3	PRKAR1B		X		cytoplasm,cytosol	transport,metabolic process,regulation of biological process,response to stimulus,cell communication,coagulation	nucleotide binding,enzyme regulator activity	cAMP-dependent protein kinase type I-beta regulatory subunit
IPI00642259.6	DST			X	cytoskeleton,membrane,cytoplasm,nucleus	regulation of biological process	protein binding,metal ion binding	Uncharacterized protein
IPI00289851.4	SLC25A25	X	X					Isoform 5 of Calcium-binding mitochondrial carrier protein SCaMC-2
IPI00480056.8	RAB4A	X	X		cytoplasm,nucleus	transport,regulation of biological process,response to stimulus,cell communication	nucleotide binding,catalytic activity	RAB4A, member RAS oncogene family variant
IPI00009619.1	CADM3	X	X	X				Isoform 2 of Cell adhesion molecule 3
IPI00247243.5	VSTM2B			X	membrane		protein binding	V-set and transmembrane domain-containing protein 2B
IPI00301936.4	ELAVL1	X	X				RNA binding,nucleotide binding	cDNA FLJ60076, highly similar to ELAV-like protein 1
IPI00008752.1	MT1G	X	X		cytoplasm,nucleus	development,regulation of biological process,response to stimulus,cell differentiation	protein binding,metal ion binding	Isoform 1 of Metallothionein-1G
IPI00021266.1	RPL23A		X		cytoplasm,ribosome,cytosol,nucleus	cell proliferation,cell organization and biogenesis,transport,metabolic process,reproduction	protein binding,RNA binding,nucleotide binding,structural molecule activity	60S ribosomal protein L23a
IPI00100197.3	NSFL1C	X	X		cytoplasm,Golgi,chromosome,nucleus		protein binding	Isoform 1 of NSFL1 cofactor p47
IPI00216587.9	RPS8	X	X		cytoplasm,ribosome,cytosol	cell organization and biogenesis,metabolic process,transport,reproduction	structural molecule activity	40S ribosomal protein S8
IPI00216318.5	YWHAB			X	cytoskeleton,cytoplasm,organelle lumen,cytosol,nucleus	cell death,development,cell organization and biogenesis,metabolic process,transport,regulation of biological process,response to stimulus,cell communication,cell differentiation	protein binding	Isoform Long of 14-3-3 protein beta/alpha
IPI00020329.6	KCNS2			X	membrane	cell organization and biogenesis,transport,regulation of biological process,cell communication	protein binding,transporter activity	Potassium voltage-gated channel subfamily S member 2
IPI00020672.4	DPP3	X	X	X	membrane,cytoplasm,organelle lumen,nucleus	metabolic process	metal ion binding,catalytic activity	Isoform 1 of Dipeptidyl peptidase 3
IPI00784865.1	IGK@			X			protein binding	IGK@ protein
IPI00395488.2	VASN	X		X	extracellular,membrane		protein binding	Vasorin
IPI00032826.1	ST13	X	X	X	cytoplasm,cytosol	cell organization and biogenesis,metabolic process,regulation of biological process	protein binding,nucleotide binding	Hsc70-interacting protein
IPI00024062.2	EPB41L1	X	X		cytoskeleton,membrane,cytoplasm,cytosol	cell organization and biogenesis,cell communication	protein binding,structural molecule activity	Isoform 1 of Band 4.1-like protein 1
IPI00024825.2	PRG4			X	extracellular	cell proliferation,response to stimulus	receptor activity	Isoform A of Proteoglycan 4
IPI00022447.3	LPGAT1		X		membrane,endoplasmic reticulum,cytoplasm	metabolic process	catalytic activity	Acyl-CoA:lysophosphatidylglycerol acyltransferase 1
IPI00883753.2	NRCAM			X	membrane		protein binding	neuronal cell adhesion molecule isoform E precursor
IPI00307591.5	ZNF609			X	nucleus		metal ion binding	Zinc finger protein 609
IPI00005675.3	NKRF			X	cytoplasm,organelle lumen,nucleus	metabolic process,regulation of biological process	DNA binding,RNA binding	NF-kappa-B-repressing factor
IPI00019906.1	BSG	X	X	X	membrane		protein binding	Isoform 2 of Basigin
IPI00550746.4	NUDC	X	X	X	cytoskeleton,cytoplasm,Golgi,organelle lumen,nucleus,cytosol	cell proliferation,cell organization and biogenesis,development,cell division,response to stimulus		Nuclear migration protein nudC
IPI00219468.4	PFN2			X	cytoskeleton,cytoplasm	cell organization and biogenesis,regulation of biological process	protein binding	Isoform IIa of Profilin-2
IPI00005981.9	TAGLN3	X	X	X		development	protein binding	Transgelin-3
IPI00006252.3	AIMP1		X	X	extracellular,endoplasmic reticulum,cytoplasm,Golgi,cytosol,nucleus	cell death,cell proliferation,development,metabolic process,regulation of biological process,response to stimulus,cellular component movement,defense response,cell communication	protein binding,RNA binding	Aminoacyl tRNA synthase complex-interacting multifunctional protein 1
IPI00007426.1	ARL6IP5	X	X		membrane,endoplasmic reticulum,cytoplasm	transport,regulation of biological process	protein binding	PRA1 family protein 3
IPI00298388.2	PIK3IP1			X	membrane	metabolic process,regulation of biological process		Isoform 1 of Phosphoinositide-3-kinase-interacting protein 1
IPI00413817.3	SERINC1	X	X		membrane,endoplasmic reticulum,cytoplasm	transport,metabolic process,regulation of biological process	protein binding,transporter activity	Serine incorporator 1

IPI	GENE	Alzheimer's Hippocampus	Control hippocampus	CSF	Cellular localization	Biological process	Molecular function	Protein Description
IPI00854667.1	IGHV3-20			X				Single chain Fv
IPI00477468.1	CTR9			X	organelle lumen,chromosome,nucleus	cell organization and biogenesis,development,metabolic process,regulation of biological process,response to stimulus,cell communication,cell differentiation	protein binding	RNA polymerase-associated protein CTR9 homolog
IPI00302641.2	FAT2			X	membrane,nucleus	cellular component movement	protein binding,metal ion binding	Protocadherin Fat 2
IPI00299573.12	RPL7A	X	X		membrane,cytoplasm,ribosome,cytosol	cell organization and biogenesis,transport,metabolic process,reproduction	protein binding,RNA binding,structural molecule activity	60S ribosomal protein L7a
IPI00027875.3	SYT11		X	X	membrane,cytoplasm	transport	protein binding,transporter activity,metal ion binding	Synaptotagmin-11
IPI00003348.3	GNB2	X	X	X	membrane,cytoplasm	metabolic process,regulation of biological process,response to stimulus,cell communication,cellular homeostasis	signal transducer activity,protein binding,catalytic activity	Guanine nucleotide-binding protein G(I)/G(S)/G(T) subunit beta-2
IPI00414320.2	ANXA11			X			metal ion binding	cDNA FLJ55482, highly similar to Annexin A11
IPI00328156.9	MAOB	X	X		mitochondrion,membrane,cytoplasm	transport,metabolic process,regulation of biological process,response to stimulus,cell communication	protein binding,catalytic activity	Amine oxidase [flavin-containing] B
IPI00023576.3	LRRTM2			X	membrane		protein binding	Leucine-rich repeat transmembrane neuronal protein 2
IPI00217766.3	SCARB2	X	X		membrane,cytoplasm,vacuole,organelle lumen	transport	protein binding,receptor activity	Lysosome membrane protein 2
IPI00031747.3	AKT3	X	X		membrane,cytoplasm,Golgi,organelle lumen,nucleus	cell organization and biogenesis,metabolic process,regulation of biological process,response to stimulus,cell communication	protein binding,nucleotide binding,catalytic activity	Isoform 1 of RAC-gamma serine/threonine-protein kinase
IPI00073454.2	COL6A2			X			protein binding	Isoform 2C2A' of Collagen alpha-2(VI) chain
IPI00071185.1	CDK11B			X		metabolic process	nucleotide binding,catalytic activity	Isoform SV1 of Cyclin-dependent kinase 11B
IPI00021728.3	EIF2S2		X		cytoplasm,cytosol	cell proliferation,development,metabolic process,reproduction	protein binding,RNA binding,metal ion binding	Eukaryotic translation initiation factor 2 subunit 2
IPI00291755.6	NUP210	X			membrane,endoplasmic reticulum,cytoplasm,nucleus	transport,metabolic process,regulation of biological process,response to stimulus,cell communication	protein binding	Isoform 1 of Nuclear pore membrane glycoprotein 210
IPI00017596.3	MAPRE1	X	X		cytoskeleton,membrane,cytoplasm,Golgi,cytosol	cell proliferation,cell organization and biogenesis,cell division,regulation of biological process	protein binding	Microtubule-associated protein RP/EB family member 1
IPI00645078.1	UBA1	X	X	X		cell death,metabolic process	protein binding,nucleotide binding,catalytic activity	Ubiquitin-like modifier-activating enzyme 1
IPI00216691.5	PFN1	X	X	X	cytoskeleton,membrane,cytoplasm,nucleus	cell organization and biogenesis,development,transport,metabolic process,regulation of biological process,response to stimulus,reproduction,coagulation	protein binding	Profilin-1
IPI00654875.1	C4B			X	extracellular,membrane	metabolic process,regulation of biological process,response to stimulus,defense response	protein binding,enzyme regulator activity	Complement C4-B
IPI00003392.1	TMEM5			X	membrane			Transmembrane protein 5
IPI00465166.2	ATP8A2		X				metal ion binding,nucleotide binding	probable phospholipid-transporting ATPase IB
IPI00216963.1	CFLAR			X		cell death,regulation of biological process	protein binding	Isoform 9 of CASP8 and FADD-like apoptosis regulator
IPI00014444.6	SERAC1			X	membrane,endoplasmic reticulum,cytoplasm	cell organization and biogenesis,transport,metabolic process	catalytic activity	Isoform 1 of Protein SERAC1
IPI00003519.1	EFTUD2	X	X		spliceosomal complex,cytoplasm,organelle lumen,nucleus	metabolic process	protein binding,nucleotide binding,catalytic activity	116 kDa U5 small nuclear ribonucleoprotein component
IPI00024032.2	TBC1D29			X		metabolic process,regulation of biological process	enzyme regulator activity	Putative TBC1 domain family member 29
IPI00019952.1	GPM6A	X	X		cell surface,membrane	development,cell organization and biogenesis,transport,regulation of biological process,cellular component movement,cell differentiation	transporter activity	Neuronal membrane glycoprotein M6-a
IPI00743766.2	FETUB			X	extracellular		enzyme regulator activity	Fetuin-B
IPI00177940.2	RDH14		X		mitochondrion,endoplasmic reticulum,cytoplasm	metabolic process	nucleotide binding,catalytic activity	Retinol dehydrogenase 14
IPI00157417.1	SEZ6L			X	membrane,endoplasmic reticulum,cytoplasm			Isoform 4 of Seizure 6-like protein
IPI00102096.3	SMAP1	X	X		membrane	development,metabolic process,regulation of biological process,cell differentiation	metal ion binding,enzyme regulator activity	Isoform 1 of Stromal membrane-associated protein 1

IPI	GENE	Alzheimer's Hippocampus	Control hippocampus	CSF	Cellular localization	Biological process	Molecular function	Protein Description
IPI00017334.1	PHB	X	X	X	membrane,mitochondrion,cytoplasm,organelle lumen,nucleus	cell death,cell proliferation,cell organization and biogenesis,metabolic process,regulation of biological process,response to stimulus,cell communication,cell growth	protein binding,DNA binding	Prohibitin
IPI00010182.4	DBI	X	X	X		transport	protein binding	Isoform 1 of Acyl-CoA-binding protein
IPI00607831.2	PRAMEF3			X			protein binding	PRAME family member 3
IPI00005809.7	SDPR			X	membrane,cytoplasm,Golgi,cytosol		protein binding	Serum deprivation-response protein
IPI00000335.1	HINT2	X	X		mitochondrion,cytoplasm	cell death,metabolic process	catalytic activity	Histidine triad nucleotide-binding protein 2, mitochondrial
IPI00157414.3	ENPP6	X	X	X	extracellular,membrane	metabolic process	catalytic activity	Ectonucleotide pyrophosphatase/phosphodiesterase family member 6
IPI00456578.4	C4orf47			X				UPF0602 protein C4orf47
IPI00221305.4	CAMK2B	X	X		cytoskeleton,membrane,endoplasmic reticulum,cytoplasm,organelle lumen,nucleus,cytosol	cell death,development,cell organization and biogenesis,metabolic process,transport,regulation of biological process,response to stimulus,cell communication,defense response,cell differentiation,reproduction	protein binding,nucleotide binding,catalytic activity	Isoform 4 of Calcium/calmodulin-dependent protein kinase type II subunit beta
IPI00329552.2	Mar-02		X		mitochondrion,membrane,cytoplasm	metabolic process,response to stimulus	metal ion binding,catalytic activity	Isoform 1 of MOSC domain-containing protein 2, mitochondrial
IPI00941900.1	CALU			X	extracellular,endoplasmic reticulum,cytoplasm,Golgi,organelle lumen	transport,response to stimulus,coagulation	protein binding,metal ion binding	Isoform 1 of Calumenin
IPI00294879.1	RANGAP1	X	X		cytoskeleton,membrane,cytoplasm,chromosome,nucleus,cytosol	cell organization and biogenesis,transport,metabolic process,regulation of biological process,response to stimulus,cell communication	protein binding,enzyme regulator activity	Ran GTPase-activating protein 1
IPI00025156.4	STUB1	X			cytoplasm,nucleus,cytosol	cell organization and biogenesis,metabolic process,regulation of biological process,response to stimulus,cell communication	protein binding,catalytic activity	Isoform 1 of E3 ubiquitin-protein ligase CHIP
IPI00973335.1	CHFR			X			protein binding,metal ion binding	Uncharacterized protein
IPI00013939.3	RPA2	X	X		organelle lumen,chromosome,nucleus	cell organization and biogenesis,metabolic process,regulation of biological process,response to stimulus	protein binding,DNA binding	Isoform 1 of Replication protein A 32 kDa subunit
IPI00002324.6	MAT2B	X	X		mitochondrion,cytoplasm,cytosol,nucleus	metabolic process,response to stimulus	protein binding,nucleotide binding,catalytic activity,enzyme regulator activity	Isoform 1 of Methionine adenosyltransferase 2 subunit beta
IPI00879915.1	C6			X	extracellular	cell death,development,metabolic process,regulation of biological process		Uncharacterized protein
IPI00883655.2	DPYSL2	X	X		cytoplasm	metabolic process	catalytic activity	dihydropyrimidinase-related protein 2 isoform 1
IPI00291328.3	NDUFV2	X	X		mitochondrion,membrane,cytoplasm	development,metabolic process,transport	metal ion binding,nucleotide binding,catalytic activity	NADH dehydrogenase [ubiquinone] flavoprotein 2, mitochondrial
IPI00939174.1	OTUB1			X	cytoplasm	metabolic process	protein binding,catalytic activity	Isoform 1 of Ubiquitin thioesterase OTUB1
IPI00784810.1	IGHV4-31			X			protein binding	IGHV4-31 protein
IPI00021485.2	LRRN1			X	membrane		protein binding	Leucine-rich repeat neuronal protein 1
IPI00002070.6	LRRC8A		X		membrane	development,cell differentiation	protein binding	Leucine-rich repeat-containing protein 8A
IPI00293817.3	NAPG	X	X		mitochondrion,membrane,cytoplasm	cell organization and biogenesis,metabolic process,transport,regulation of biological process	protein binding	Gamma-soluble NSF attachment protein
IPI00473011.3	HBD	X	X	X	cytoplasm,cytosol	transport,response to stimulus,coagulation	transporter activity,metal ion binding	Hemoglobin subunit delta
IPI00030229.4	GALE			X		metabolic process	nucleotide binding,catalytic activity	Uncharacterized protein
IPI00016335.3	IFT46		X			cell organization and biogenesis,transport,cellular component movement		Isoform 2 of Intraflagellar transport protein 46 homolog
IPI00216983.6	CA3			X	cytoplasm	metabolic process,response to stimulus	metal ion binding,catalytic activity	Carbonic anhydrase 3
IPI00016915.1	IGFBP7			X	extracellular	cell proliferation,cell organization and biogenesis,development,regulation of biological process,cell growth	protein binding	Insulin-like growth factor-binding protein 7
IPI00329753.4	ZC2HC1A	X	X				metal ion binding	Protein FAM164A
IPI00011571.2	PDIA2		X		endoplasmic reticulum,cytoplasm,organelle lumen	cell death,metabolic process,regulation of biological process,response to stimulus,cellular homeostasis	protein binding,catalytic activity	Isoform 1 of Protein disulfide-isomerase A2
IPI00022463.1	TF			X	extracellular	transport,cellular homeostasis	metal ion binding	serotransferrin precursor

IPI	GENE	Alzheimer's Hippocampus	Control hippocampus	CSF	Cellular localization	Biological process	Molecular function	Protein Description
IPI00022429.3	ORM1		X	X	extracellular	transport,regulation of biological process,response to stimulus,defense response	protein binding	Alpha-1-acid glycoprotein 1
IPI00215928.4	CETN2	X	X		cytoskeleton,cytoplasm,nucleus,cytosol	cell organization and biogenesis,cell division,metabolic process,regulation of biological process,response to stimulus,reproduction	protein binding,metal ion binding,nucleotide binding,catalytic activity	Centrin-2
IPI00853525.1	APOA1			X	extracellular	transport,metabolic process		Uncharacterized protein
IPI00023087.1	UBE2T			X	organelle lumen,nucleus	metabolic process,response to stimulus	protein binding,nucleotide binding,catalytic activity	Ubiquitin-conjugating enzyme E2 T
IPI00658152.1	TNS3	X	X		membrane	cell proliferation,development,regulation of biological process,cellular component movement	protein binding	Isoform 1 of Tensin-3
IPI00297288.7	ARHGAP31			X	membrane,cytoplasm,cytosol	metabolic process,regulation of biological process,response to stimulus,cell communication	protein binding,enzyme regulator activity	Rho GTPase-activating protein 31
IPI00554752.3	PRKAR2B	X	X	X	cytoskeleton,mitochondrion,membrane,cytoplasm,cytosol	metabolic process,transport,regulation of biological process,response to stimulus,cell communication,coagulation	protein binding,nucleotide binding,enzyme regulator activity	cAMP-dependent protein kinase type II-beta regulatory subunit
IPI00376237.1	LBX2			X		metabolic process,regulation of biological process	DNA binding	Isoform 2 of Transcription factor LBX2
IPI00060308.3	PDLIM7			X			protein binding	Isoform 6 of PDZ and LIM domain protein 7
IPI00017726.1	HSD17B10	X	X		membrane,mitochondrion,endoplasmic reticulum,cytoplasm,organelle lumen	cell organization and biogenesis,development,metabolic process,reproduction,cell differentiation	protein binding,nucleotide binding,catalytic activity	Isoform 1 of 3-hydroxyacyl-CoA dehydrogenase type-2
IPI00552419.3	PCCA	X	X		extracellular	metabolic process	metal ion binding,nucleotide binding,catalytic activity	propionyl-CoA carboxylase alpha chain, mitochondrial isoform c precursor
IPI00017592.1	LETM1	X	X		membrane,mitochondrion,cytoplasm	cell organization and biogenesis	protein binding,metal ion binding	Isoform 1 of LETM1 and EF-hand domain-containing protein 1, mitochondrial
IPI00008994.2	NDRG2	X	X	X	cytoskeleton,cytoplasm,Golgi,organelle lumen,cytosol,nucleus	cell proliferation,development,regulation of biological process,response to stimulus,cell communication,cell differentiation	protein binding	Isoform 1 of Protein NDRG2
IPI00008219.1	RAD23A	X	X		proteasome,nucleus	metabolic process,regulation of biological process,response to stimulus,reproduction	protein binding,DNA binding	UV excision repair protein RAD23 homolog A
IPI00550876.5	MRO			X	organelle lumen,nucleus			Protein maestro
IPI00000459.6	PRRG1			X	extracellular,membrane		metal ion binding	Transmembrane gamma-carboxyglutamic acid protein 1
IPI00554701.2	UQCR10	X	X		membrane,mitochondrion,cytoplasm	transport,metabolic process	transporter activity,catalytic activity	Isoform 1 of Cytochrome b-c1 complex subunit 9
IPI00010471.6	LCP1	X	X	X	extracellular,cytoskeleton,membrane,cytoplasm,cytosol	cell organization and biogenesis,development,transport,regulation of biological process,response to stimulus,cell communication	protein binding,metal ion binding	Plastin-2
IPI00006128.1	SPOCK2			X	extracellular	development,cell organization and biogenesis,metabolic process,regulation of biological process,response to stimulus,cell communication,cell differentiation	protein binding,metal ion binding	Testican-2
IPI00847271.3	CARNS1	X	X			metabolic process	metal ion binding,nucleotide binding,catalytic activity	Isoform 1 of Carnosine synthase 1
IPI00071824.3	CKAP2			X	cytoskeleton,cytoplasm	cell death,cell organization and biogenesis,cell division,metabolic process,regulation of biological process		Isoform 1 of Cytoskeleton-associated protein 2
IPI00030364.1	TAF10	X			cytoplasm,organelle lumen,nucleus	cell organization and biogenesis,metabolic process,regulation of biological process	protein binding,catalytic activity	Transcription initiation factor TFIID subunit 10
IPI00383680.3	RPN2			X	membrane,endoplasmic reticulum,cytoplasm	metabolic process	catalytic activity	dolichyl-diphosphooligosaccharide--protein glycosyltransferase subunit 2 isoform 2 precursor
IPI00018272.3	PNPO	X	X		cytoplasm,cytosol	metabolic process	nucleotide binding,catalytic activity	Pyridoxine-5'-phosphate oxidase
IPI00007063.5	HDGFRP3	X	X		cytoplasm,nucleus	cell proliferation	protein binding	Hepatoma-derived growth factor-related protein 3
IPI00607744.3	DNAJC11	X					protein binding	Isoform 2 of DnaJ homolog subfamily C member 11
IPI00384722.2	EMC10			X				Isoform 2 of UPF0510 protein INM02
IPI00719600.5	CYFIP2	X	X		cytoplasm			Isoform 2 of Cytoplasmic FMR1-interacting protein 2
IPI00219249.4	CNTNAP1	X	X		membrane	cell organization and biogenesis,development,transport,regulation of biological process,response to stimulus,cellular component movement,cell communication,cellular homeostasis,cell differentiation	protein binding,receptor activity	Contactin-associated protein 1

IPI	GENE	Alzheimer's Hippocampus	Control hippocampus	CSF	Cellular localization	Biological process	Molecular function	Protein Description
IPI00219005.3	FKBP4	X	X	X	cytoskeleton,membrane,cytoplasm,organelle lumen,cytosol,nucleus	development,cell organization and biogenesis,transport,metabolic process,regulation of biological process,response to stimulus,cell communication,reproduction,cell differentiation	protein binding,nucleotide binding,catalytic activity	Peptidyl-prolyl cis-trans isomerase FKBP4
IPI00008085.5	SLC39A10			X	membrane	transport	transporter activity	Zinc transporter ZIP10
IPI00029722.4	KIF5A			X	cytoskeleton,membrane,cytoplasm,cytosol	cell death,response to stimulus,cellular component movement,cell communication,coagulation	motor activity,nucleotide binding,catalytic activity	Kinesin heavy chain isoform 5A
IPI00029079.5	GMPS		X		cytoplasm,cytosol	metabolic process	nucleotide binding,catalytic activity	GMP synthase [glutamine-hydrolyzing]
IPI00029656.1	DFNA5	X	X			cell death,cell proliferation,development,regulation of biological process,cell differentiation		Isoform Long of Non-syndromic hearing impairment protein 5
IPI00871390.1	RBFOX3			X		metabolic process,regulation of biological process	RNA binding,nucleotide binding	39 kDa protein
IPI00215920.8	ARF6	X	X		membrane,cytoplasm,Golgi,nucleus,endosome	cell death,cell organization and biogenesis,development,transport,metabolic process,regulation of biological process,response to stimulus,cellular component movement,cell communication,cell differentiation	protein binding,signal transducer activity,metal ion binding,nucleotide binding,catalytic activity,enzyme regulator activity	ADP-ribosylation factor 6
IPI00465261.2	ERAP2			X	membrane,endoplasmic reticulum,cytoplasm,organelle lumen	metabolic process	metal ion binding,catalytic activity	Isoform 1 of Endoplasmic reticulum aminopeptidase 2
IPI00005978.8	SRSF2	X	X		spliceosomal complex,organelle lumen,nucleus	transport,metabolic process	protein binding,RNA binding,nucleotide binding	Serine/arginine-rich splicing factor 2
IPI00019533.3	CHI3L2			X	extracellular	metabolic process	catalytic activity	Chitinase-3-like protein 2
IPI00022314.1	SOD2	X	X	X	membrane,mitochondrion,cytoplasm,organelle lumen	cell death,cell proliferation,development,cell organization and biogenesis,metabolic process,regulation of biological process,response to stimulus,cell communication,cellular homeostasis,cell differentiation	antioxidant activity,protein binding,DNA binding,metal ion binding,catalytic activity	Superoxide dismutase [Mn], mitochondrial
IPI00059764.4	ZNF428	X	X				metal ion binding	Isoform 1 of Zinc finger protein 428
IPI00554786.5	TXNRD1	X	X	X	cytoplasm	metabolic process,regulation of biological process,cellular homeostasis	catalytic activity	Isoform 5 of Thioredoxin reductase 1, cytoplasmic
IPI00794119.1	ABCC8			X			nucleotide binding,catalytic activity	13 kDa protein
IPI00300567.1	ECI1	X	X		membrane,mitochondrion,cytoplasm,organelle lumen	metabolic process	protein binding,catalytic activity	Isoform 1 of Enoyl-CoA delta isomerase 1, mitochondrial
IPI00307729.3	ADAMTS3			X	extracellular	cell organization and biogenesis,metabolic process	metal ion binding,catalytic activity	A disintegrin and metalloproteinase with thrombospondin motifs 3
IPI00013682.2	ART3			X	membrane	metabolic process	catalytic activity	Isoform 3 of Ecto-ADP-ribosyltransferase 3
IPI00009028.1	CLEC3B			X				C-type lectin domain family 3, member B
IPI00015695.3	C11orf63		X					Isoform 1 of Uncharacterized protein C11orf63
IPI00013485.3	RPS2	X	X		cytoplasm,ribosome,organelle lumen,cytosol,nucleus	cell organization and biogenesis,metabolic process,transport,reproduction	protein binding,RNA binding,structural molecule activity	40S ribosomal protein S2
IPI00420108.6	DLST	X	X		mitochondrion,membrane,cytoplasm,organelle lumen,nucleus	metabolic process	protein binding,catalytic activity	Dihydrolipoyllysine-residue succinyltransferase component of 2-oxoglutarate dehydrogenase complex, mitochondrial
IPI00302592.2	FLNA	X	X	X			protein binding	Isoform 2 of Filamin-A
IPI00003482.1	DECR1	X	X		mitochondrion,cytoplasm,organelle lumen,nucleus	cell organization and biogenesis,metabolic process	nucleotide binding,catalytic activity	2,4-dienoyl-CoA reductase, mitochondrial
IPI00607799.5	BDH2	X	X		mitochondrion,cytoplasm	metabolic process	nucleotide binding,catalytic activity	Isoform 1 of 3-hydroxybutyrate dehydrogenase type 2
IPI00012977.1	BTN3A3	X	X		membrane		protein binding	Butyrophilin subfamily 3 member A3
IPI00021634.3	KLC2	X	X	X	cytoskeleton,cytoplasm,cytosol	transport,response to stimulus,cellular component movement,coagulation	protein binding,motor activity,catalytic activity	Kinesin light chain 2
IPI00022275.6	SACM1L		X		membrane,endoplasmic reticulum,cytoplasm,Golgi	metabolic process	catalytic activity	Phosphatidylinositide phosphatase SAC1
IPI00000027.3	ADCYAP1			X	extracellular,membrane		protein binding	cDNA FLJ56302, highly similar to Pituitary adenylate cyclase-activating polypeptide
IPI00028381.2	DLK2			X	membrane	regulation of biological process,cell differentiation	protein binding,metal ion binding	Isoform 1 of Protein delta homolog 2
IPI00061520.4	B3GNT9			X	membrane,cytoplasm,Golgi	metabolic process	catalytic activity	UDP-GlcNAc:betaGal beta-1,3-N-acetylglucosaminyltransferase 9
IPI00023359.1	MCAT			X	mitochondrion,cytoplasm	metabolic process	protein binding,catalytic activity	Isoform 1 of Malonyl-CoA-acyl carrier protein transacylase, mitochondrial
IPI00410325.4	LDLRAD3			X	membrane		protein binding,receptor activity	Low-density lipoprotein receptor class A domain-containing protein 3

IPI	GENE	Alzheimer's Hippocampus	Control hippocampus	CSF	Cellular localization	Biological process	Molecular function	Protein Description
IPI00294862.2	PCSK5			X	membrane	metabolic process,regulation of biological process,response to stimulus,cell communication	signal transducer activity,nucleotide binding,receptor activity,catalytic activity	Proprotein convertase subtilisin/kexin type 5
IPI00185088.6	IGSF11			X	membrane	regulation of biological process	protein binding,receptor activity	Isoform 1 of Immunoglobulin superfamily member 11
IPI00783665.5	LAMA5	X		X	extracellular,cytoskeleton,membrane,cytoplasm	cell proliferation,development,cell organization and biogenesis,transport,regulation of biological process,response to stimulus,cellular component movement,cell communication,cell differentiation	protein binding,motor activity,structural molecule activity,catalytic activity	Laminin subunit alpha-5
IPI00010697.2	ITGA6	X	X		membrane	cell death,cell organization and biogenesis,metabolic process,regulation of biological process,response to stimulus,cellular component movement,cell communication,coagulation	protein binding,receptor activity	Isoform Alpha-6X1X2B of Integrin alpha-6
IPI00301109.4	PPA2	X	X		mitochondrion,cytoplasm,organelle lumen	metabolic process	metal ion binding,catalytic activity	Isoform 1 of Inorganic pyrophosphatase 2, mitochondrial
IPI00419908.5	GPR179			X	membrane	regulation of biological process,response to stimulus,cell communication	protein binding,signal transducer activity,receptor activity	Probable G-protein coupled receptor 179
IPI00927702.1	SUSD5			X				Protein
IPI00645363.2	IGHG1			X	membrane		protein binding	Putative uncharacterized protein DKFZp686P15220
IPI00034319.2	CUTA	X	X	X		response to stimulus		Isoform A of Protein CutA
IPI00303722.5	FAM136A	X	X		mitochondrion,cytoplasm			Protein FAM136A
IPI00020950.3	PPM1A	X	X		membrane,cytoplasm,cytosol,nucleus	cell organization and biogenesis,metabolic process,regulation of biological process,response to stimulus,cell communication	signal transducer activity,protein binding,metal ion binding,catalytic activity	Isoform Alpha-1 of Protein phosphatase 1A
IPI00298956.6	FSTL4			X				Isoform 2 of Follistatin-related protein 4
IPI00550558.6	POMGNT1			X	membrane,cytoplasm,Golgi	metabolic process	catalytic activity	Protein O-linked-mannose beta-1,2-N-acetylglucosaminyltransferase 1
IPI00002147.4	CHI3L1			X	extracellular,endoplasmic reticulum,cytoplasm	development,transport,metabolic process,regulation of biological process,response to stimulus,defense response,cell communication	structural molecule activity,catalytic activity	Chitinase-3-like protein 1
IPI00033130.3	SAE1	X	X		nucleus	metabolic process,regulation of biological process	protein binding,nucleotide binding,catalytic activity,enzyme regulator activity	SUMO-activating enzyme subunit 1
IPI00177728.3	CNDP2	X	X		cytoplasm,cytosol	metabolic process,response to stimulus	metal ion binding,catalytic activity	Isoform 1 of Cytosolic non-specific dipeptidase
IPI00019242.1	MMP15			X	membrane	metabolic process,regulation of biological process,response to stimulus	protein binding,metal ion binding,catalytic activity,enzyme regulator activity	Matrix metalloproteinase-15
IPI01019005.1	ATXN10	X	X		cytoplasm,cytosol	cell death,cell organization and biogenesis,development,cell differentiation	protein binding	Ataxin-10
IPI00000959.2	VIP			X	extracellular	cell proliferation,metabolic process,transport,regulation of biological process,response to stimulus,cell communication	protein binding	Isoform 1 of VIP peptides
IPI00009920.3	C6			X	extracellular,membrane	cell death,development,metabolic process,regulation of biological process,response to stimulus,defense response	protein binding	complement component C6 precursor
IPI00786926.1	IGHV1-69			X			protein binding	Myosin-reactive immunoglobulin heavy chain variable region (Fragment)
IPI00008034.1	RAB23	X	X		membrane	development,metabolic process,transport,regulation of biological process,response to stimulus,cell communication	nucleotide binding,catalytic activity	Ras-related protein Rab-23
IPI00373872.7	PKD1L2			X	membrane	transport,regulation of biological process,response to stimulus,cell communication	protein binding,transporter activity,metal ion binding	Isoform 1 of Polycystic kidney disease protein 1-like 2
IPI00303071.4	CECR1			X	extracellular,cytoplasm,Golgi,cytosol	development,metabolic process	protein binding,metal ion binding,catalytic activity	Isoform 1 of Adenosine deaminase CECR1
IPI00455739.2	LFNG			X	extracellular,membrane,cytoplasm,Golgi	development,regulation of biological process,response to stimulus,cell communication,reproduction	metal ion binding,catalytic activity	Isoform 1 of Beta-1,3-N-acetylglucosaminyltransferase lunatic fringe
IPI00303568.3	PTGES2	X	X		membrane,mitochondrion,cytoplasm,Golgi,cytosol,nucleus	transport,metabolic process,regulation of biological process,cellular homeostasis	DNA binding,catalytic activity	Prostaglandin E synthase 2
IPI00012726.4	PABPC4		X		cytoplasm,nucleus	metabolic process,response to stimulus,coagulation	protein binding,RNA binding,nucleotide binding	Isoform 1 of Polyadenylate-binding protein 4
IPI00008997.5	WFDC1			X	extracellular	cell proliferation,cell organization and biogenesis,metabolic process,regulation of biological process,response to stimulus,cell growth	enzyme regulator activity	WAP four-disulfide core domain protein 1
IPI00455444.1	POLR2M	X						Isoform 4 of Protein GRINL1A, isoforms 4/5

IPI	GENE	Alzheimer's Hippocampus	Control hippocampus	CSF	Cellular localization	Biological process	Molecular function	Protein Description
IPI00291417.2	DCAKD	X			mitochondrion,cytoplasm	metabolic process	nucleotide binding,catalytic activity	Isoform 1 of Dephospho-CoA kinase domain-containing protein
IPI00060650.3	PCNP	X	X					Isoform 2 of PEST proteolytic signal-containing nuclear protein
IPI00419802.4	HIBCH	X	X		mitochondrion,cytoplasm,organelle lumen	metabolic process	catalytic activity	Isoform 1 of 3-hydroxyisobutyryl-CoA hydrolase, mitochondrial
IPI00792759.1	THSD4			X			catalytic activity	cDNA FLJ53165, weakly similar to Homo sapiens thrombospondin repeat containing 1 (TSRC1), transcript variant 1, mRNA
IPI00479306.1	PSMB5	X	X		proteasome,cytoplasm,organelle lumen,nucleus,cytosol	cell death,metabolic process,regulation of biological process,response to stimulus,cell communication	protein binding,catalytic activity	Isoform 1 of Proteasome subunit beta type-5
IPI00027744.1	NR3C2			X	membrane,endoplasmic reticulum,cytoplasm,organelle lumen,nucleus	metabolic process,regulation of biological process,response to stimulus,cell communication	protein binding,signal transducer activity,DNA binding,metal ion binding,receptor activity	Isoform 1 of Mineralocorticoid receptor
IPI00032405.2	GPR37L1	X	X	X	membrane	regulation of biological process,response to stimulus,cell communication	protein binding,signal transducer activity,receptor activity	Endothelin B receptor-like protein 2
IPI00220834.8	XRCC5	X	X		cytoplasm,organelle lumen,chromosome,nucleus	cell death,cell proliferation,cell organization and biogenesis,development,metabolic process,regulation of biological process,response to stimulus,cell differentiation,reproduction	protein binding,DNA binding,nucleotide binding,catalytic activity	X-ray repair cross-complementing protein 5
IPI00298423.3	PDHX	X	X		mitochondrion,cytoplasm,organelle lumen	metabolic process,regulation of biological process	catalytic activity	Pyruvate dehydrogenase protein X component, mitochondrial
IPI00009688.1	PIP4K2A	X	X		membrane,cytoplasm,nucleus	development,metabolic process,cell differentiation	nucleotide binding,catalytic activity	Phosphatidylinositol-5-phosphate 4-kinase type-2 alpha
IPI00015756.2	PTPRK			X	cell surface,membrane	cell proliferation,cell organization and biogenesis,metabolic process,regulation of biological process,response to stimulus,cellular component movement,cell communication	signal transducer activity,protein binding,receptor activity,catalytic activity	Isoform 1 of Receptor-type tyrosine-protein phosphatase kappa
IPI00025311.2	BCAS1	X	X	X	cytoplasm			Isoform 1 of Breast carcinoma-amplified sequence 1
IPI00171410.1	XXYLT1			X	membrane,endoplasmic reticulum,cytoplasm		catalytic activity	Isoform 1 of Uncharacterized protein C3orf21
IPI00646640.1	CLVS2		X		membrane,cytoplasm,Golgi,endosome	cell organization and biogenesis,transport	transporter activity	Isoform 1 of Clavesin-2
IPI00651653.2	DDX17	X	X				nucleotide binding,catalytic activity	probable ATP-dependent RNA helicase DDX17 isoform 3
IPI00016006.2	GPHN	X	X		cytoskeleton,membrane,cytoplasm	metabolic process	metal ion binding,nucleotide binding,catalytic activity	Isoform 1 of Gephyrin
IPI00174757.3	FBXL20		X		cytoplasm		protein binding	Isoform 1 of F-box/LRR-repeat protein 20
IPI00019903.1	TACO1		X		mitochondrion,cytoplasm	metabolic process,regulation of biological process		Translational activator of cytochrome c oxidase 1
IPI00877084.1	CCDC144C			X	membrane			Coiled-coil domain-containing protein 144C
IPI00030431.1	ANTXR1			X	membrane	cell organization and biogenesis,cell differentiation,reproduction	signal transducer activity,protein binding,metal ion binding,receptor activity	Isoform 1 of Anthrax toxin receptor 1
IPI00032311.4	LBP		X	X	extracellular	metabolic process,transport,regulation of biological process,response to stimulus,cellular component movement,cell communication,defense response	protein binding	Lipopolysaccharide-binding protein
IPI00170508.1	CAMK1D	X	X		cytoplasm,nucleus	cell death,cell organization and biogenesis,development,transport,metabolic process,regulation of biological process,response to stimulus,cellular component movement,defense response,cell differentiation	protein binding,nucleotide binding,catalytic activity	Isoform 1 of Calcium/calmodulin-dependent protein kinase type 1D
IPI00031549.5	DSC3			X	extracellular,membrane,cytoplasm	development,metabolic process,regulation of biological process	protein binding,metal ion binding	Isoform 3A of Desmocollin-3
IPI00030255.1	PLOD3			X	membrane,endoplasmic reticulum,cytoplasm	cell organization and biogenesis,development,metabolic process,response to stimulus,cell differentiation	protein binding,metal ion binding,catalytic activity	Procollagen-lysine,2-oxoglutarate 5-dioxygenase 3
IPI00295777.6	GPD1	X	X		mitochondrion,cytoplasm,cytosol	metabolic process	protein binding,nucleotide binding,catalytic activity	Glycerol-3-phosphate dehydrogenase [NAD+], cytoplasmic
IPI00005780.3	OGT	X	X		cytoskeleton,mitochondrion,membrane,cytoplasm,organelle lumen,cytosol,nucleus	cell death,cell organization and biogenesis,development,metabolic process,regulation of biological process,response to stimulus,cell communication,cell differentiation	protein binding,catalytic activity,enzyme regulator activity	Isoform 3 of UDP-N-acetylglucosamine--peptide N-acetylglucosaminyltransferase 110 kDa subunit
IPI00059164.1	GAL3ST3			X	membrane,cytoplasm,Golgi	metabolic process	nucleotide binding,catalytic activity	Galactose-3-O-sulfotransferase 3
IPI00032179.3	SERPINC1			X	extracellular,membrane	metabolic process,regulation of biological process,response to stimulus,defense response,coagulation	protein binding,enzyme regulator activity	Antithrombin-III
IPI00478890.3	SPOCK3			X	extracellular	regulation of biological process,response to stimulus,cell communication	protein binding,metal ion binding	Isoform 1 of Testican-3

IPI	GENE	Alzheimer's Hippocampus	Control hippocampus	CSF	Cellular localization	Biological process	Molecular function	Protein Description
IPI00002606.5	SCIN	X	X		cytoskeleton,cytoplasm	cell death,cell proliferation,development,cell organization and biogenesis,transport,regulation of biological process,cell differentiation	protein binding,metal ion binding	Isoform 1 of Adseverin
IPI00552671.3	PLXNA1	X	X		membrane	cell organization and biogenesis,development,regulation of biological process,response to stimulus,cell communication,cell differentiation	signal transducer activity,protein binding,receptor activity	Plexin-A1
IPI00162735.3	ATRN			X			protein binding	Isoform 2 of Attractin
IPI00009771.6	LMNB2	X	X	X	cytoskeleton,membrane	metabolic process,regulation of biological process	motor activity,catalytic activity	Lamin-B2
IPI00449920.1	IGHA1			X	membrane		protein binding	cDNA FLJ90170 fis, clone MAMMA1000370, highly similar to Ig alpha-1 chain C region
IPI00398154.2	AFAP1			X	cytoskeleton,membrane,cytoplasm		protein binding	Actin filament-associated protein 1
IPI00847759.2	DENND4B			X	membrane			DENN domain-containing protein 4B
IPI00218924.5	CHP1		X		cytoskeleton,membrane,endoplasmic reticulum,cytoplasm,Golgi,cytosol,nucleus	cell organization and biogenesis,transport,metabolic process,regulation of biological process,response to stimulus,cell communication,cellular homeostasis	protein binding,transporter activity,metal ion binding	Calcium-binding protein p22
IPI00477714.5	IGLV8-61			X				V3-4 protein
IPI00216138.6	TAGLN	X	X	X	cytoplasm	development	protein binding	Transgelin
IPI00102678.2	PCNX			X	membrane			Isoform 1 of Pecanex-like protein 1
IPI00022394.2	C1QC		X	X	extracellular,membrane	development,metabolic process,regulation of biological process,response to stimulus,defense response,cell differentiation	protein binding	Complement C1q subcomponent subunit C
IPI00916253.1	MDH1	X	X		mitochondrion,cytoplasm	metabolic process	nucleotide binding,catalytic activity	Uncharacterized protein
IPI00003327.1	ARL3	X	X		cytoskeleton,membrane,cytoplasm,Golgi,organelle lumen,nucleus	cell organization and biogenesis,development,metabolic process,cell division,transport,regulation of biological process,response to stimulus,cell communication,cell differentiation	protein binding,signal transducer activity,metal ion binding,nucleotide binding,catalytic activity	ADP-ribosylation factor-like protein 3
IPI00413344.3	CFL2	X	X	X	cytoskeleton,cytoplasm,organelle lumen,nucleus		protein binding	Cofilin-2
IPI00001663.1	HTRA2	X	X		membrane,mitochondrion,endoplasmic reticulum,cytoplasm,cytosol,nucleus	cell death,cell organization and biogenesis,development,metabolic process,regulation of biological process,response to stimulus,cell communication,cell differentiation	protein binding,catalytic activity	Isoform 1 of Serine protease HTRA2, mitochondrial
IPI00025331.4	SLC17A7	X	X		membrane,cytoplasm	transport,response to stimulus,cell communication,cellular homeostasis	transporter activity	Vesicular glutamate transporter 1
IPI00550841.1	PDZD11		X		extracellular,membrane,cytoplasm,cytosol		protein binding	Isoform 1 of PDZ domain-containing protein 11
IPI00412492.4	PLXND1			X	membrane	development,cell organization and biogenesis,regulation of biological process,response to stimulus,cellular component movement,cell communication,cell differentiation	protein binding,signal transducer activity,receptor activity	Isoform 1 of Plexin-D1
IPI00328587.4	EDARADD			X	cytoplasm,cytosol	metabolic process	metal ion binding,catalytic activity	EDAR-associated death domain, isoform CRA_a
IPI00006662.1	APOD	X	X	X	extracellular,endoplasmic reticulum,cytoplasm,ribosome,cytosol	cell proliferation,development,cell organization and biogenesis,metabolic process,transport,regulation of biological process,response to stimulus,cellular component movement,defense response,cell communication,cell differentiation	protein binding,transporter activity	Apolipoprotein D
IPI00740545.1	POTEI			X			protein binding,nucleotide binding	POTE ankyrin domain family member I
IPI00007798.1	TRHDE			X	membrane	metabolic process,regulation of biological process,response to stimulus,cell communication	metal ion binding,catalytic activity	Thyrotropin-releasing hormone-degrading ectoenzyme
IPI00003353.1	NREP			X	cytoplasm,nucleus	cell organization and biogenesis,development,regulation of biological process,response to stimulus,cell communication,cell differentiation		Neuronal protein 3.1
IPI00884353.2	ETS1			X	nucleus	metabolic process,regulation of biological process	DNA binding	protein C-ets-1 isoform 3
IPI00855946.1	SNX30	X			cytoplasm	transport,cell communication	protein binding	Sorting nexin-30
IPI00000001.2	STAU1	X	X		cytoskeleton,endoplasmic reticulum,cytoplasm		protein binding,RNA binding	Isoform Long of Double-stranded RNA-binding protein Staufen homolog 1

IPI	GENE	Alzheimer's Hippocampus	Control hippocampus	CSF	Cellular localization	Biological process	Molecular function	Protein Description
IPI00746963.1	IGKC			X			protein binding	IGKC protein
IPI00003590.2	QSOX1			X	extracellular,membrane,cytoplasm,Golgi	metabolic process,regulation of biological process,cellular homeostasis	catalytic activity	Isoform 1 of Sulfhydryl oxidase 1
IPI00334238.1	NPTXR	X	X	X	extracellular,membrane	response to stimulus	protein binding,signal transducer activity,metal ion binding,receptor activity	Neuronal pentraxin receptor
IPI00178894.8	ZBTB20			X	organelle lumen,nucleus	metabolic process,regulation of biological process	protein binding,DNA binding,metal ion binding	Isoform 1 of Zinc finger and BTB domain-containing protein 20
IPI00024107.1	SNCA	X	X	X	extracellular,cytoskeleton,membrane,mitochondrion,cytoplasm,cytosol, nucleus	cell death,cell organization and biogenesis,metabolic process,transport,regulation of biological process,response to stimulus,cell communication,defense response,cellular homeostasis	protein binding,transporter activity,metal ion binding,catalytic activity,enzyme regulator activity	Isoform 1 of Alpha-synuclein
IPI00297655.4	NOTCH2			X	extracellular,cell surface,membrane,endoplasmic reticulum,cytoplasm,Golgi,organelle lumen,nucleus,cytosol	cell death,cell proliferation,development,metabolic process,transport,regulation of biological process,response to stimulus,defense response,cell communication,cell differentiation,cell growth	protein binding,metal ion binding,receptor activity	Neurogenic locus notch homolog protein 2
IPI00296789.2	FBXO3	X	X		nucleus	metabolic process	protein binding,catalytic activity	Isoform 1 of F-box only protein 3
IPI00743716.1	HLA-C			X	membrane	response to stimulus	protein binding,signal transducer activity,receptor activity	HLA class I histocompatibility antigen, Cw-12 alpha chain
IPI00017895.3	GPD2	X	X		mitochondrion,membrane,cytoplasm	metabolic process	metal ion binding,catalytic activity	Isoform 1 of Glycerol-3-phosphate dehydrogenase, mitochondrial
IPI00026285.5	ST8SIA3			X	membrane,cytoplasm,Golgi	metabolic process	catalytic activity	Sia-alpha-2,3-Gal-beta-1,4-GlcNAc-R:alpha 2,8-sialyltransferase
IPI00290094.5	SFSWAP			X	nucleus	cell organization and biogenesis,metabolic process,regulation of biological process	RNA binding	Splicing factor, suppressor of white-apricot homolog
IPI00290292.7	RHBDF1			X	membrane,endoplasmic reticulum,cytoplasm,Golgi	cell proliferation,transport,metabolic process,regulation of biological process,response to stimulus,cellular component movement,cell communication	protein binding,catalytic activity	Inactive rhomboid protein 1
IPI00218463.3	PAM16		X		mitochondrion,membrane,cytoplasm	cell organization and biogenesis,metabolic process,transport		Mitochondrial import inner membrane translocase subunit TIM16
IPI00337335.8	MYH14		X		cytoskeleton,cytoplasm	cell organization and biogenesis,development,regulation of biological process,response to stimulus,cellular component movement,cell differentiation	protein binding,nucleotide binding,motor activity,catalytic activity	Isoform 1 of Myosin-14
IPI00030634.2	GGT7			X	cell surface,membrane	metabolic process	catalytic activity	Isoform 1 of Gamma-glutamyltransferase 7
IPI00789181.3	PLCL1	X	X	X	cytoplasm	metabolic process,regulation of biological process,response to stimulus,cell communication	protein binding,signal transducer activity,metal ion binding,catalytic activity	Isoform 1 of Inactive phospholipase C-like protein 1
IPI00640818.3	PNPLA6			X	membrane	metabolic process		neuropathy target esterase isoform a
IPI00410657.2	RNMT			X		metabolic process		Isoform 2 of mRNA cap guanine-N7 methyltransferase
IPI00032904.1	SNCB	X	X	X	membrane,mitochondrion,cytoplasm,Golgi,organelle lumen,nucleus	cell death,metabolic process,regulation of biological process	metal ion binding,enzyme regulator activity	Beta-synuclein
IPI00016870.1	ZP2			X	extracellular,membrane,endoplasmic reticulum,cytoplasm,Golgi,endosome	transport,regulation of biological process,reproduction	protein binding,signal transducer activity,receptor activity	Zona pellucida sperm-binding protein 2
IPI00059395.3	KIFC2			X	cytoskeleton,cytoplasm	cellular component movement	motor activity,nucleotide binding,catalytic activity	Kinesin-like protein KIFC2
IPI00014398.4	FHL1	X	X	X			metal ion binding	four and a half LIM domains protein 1 isoform 5
IPI00101608.4	CRELD1		X	X			metal ion binding	Isoform 2 of Cysteine-rich with EGF-like domain protein 1
IPI00515021.2	ELN			X	extracellular		structural molecule activity	Isoform 4 of Elastin
IPI00816741.1	C5			X	extracellular	metabolic process,regulation of biological process,response to stimulus,defense response	enzyme regulator activity	Complement component 5 variant (Fragment)
IPI00871535.1	SPTAN1	X	X				protein binding	Isoform 2 of Spectrin alpha chain, brain
IPI00299699.1	NPDC1			X	membrane			Neural proliferation differentiation and control protein 1
IPI00291262.3	CLU	X	X	X	extracellular,membrane,mitochondrion,endoplasmic reticulum,cytoplasm,organelle lumen,cytosol,nucleus	cell death,cell proliferation,development,cell organization and biogenesis,transport,metabolic process,regulation of biological process,response to stimulus,defense response,cell communication,cell differentiation,coagulation	protein binding,catalytic activity	Isoform 1 of Clusterin
IPI00032808.1	RAB3D		X		membrane,mitochondrion,cytoplasm	transport,metabolic process,regulation of biological process,response to stimulus,cell communication	protein binding,nucleotide binding,catalytic activity	Ras-related protein Rab-3D

IPI	GENE	Alzheimer's Hippocampus	Control hippocampus	CSF	Cellular localization	Biological process	Molecular function	Protein Description
IPI00413265.4	SMC5			X	organelle lumen,chromosome,nucleus	cell organization and biogenesis,metabolic process,cell division,regulation of biological process,response to stimulus	protein binding,nucleotide binding	Structural maintenance of chromosomes protein 5
IPI00304692.1	RBMX	X	X		extracellular,spliceosomal complex,organelle lumen,chromosome,nucleus	cell organization and biogenesis,metabolic process,regulation of biological process,response to stimulus	protein binding,DNA binding,RNA binding,nucleotide binding	Heterogeneous nuclear ribonucleoprotein G
IPI00005614.6	SPTBN1	X	X	X	cytoskeleton,membrane,cytoplasm,organelle lumen,nucleus,cytosol	development,cell organization and biogenesis,transport,metabolic process,cell division,regulation of biological process,response to stimulus,cell communication,cell differentiation	protein binding,structural molecule activity	Isoform Long of Spectrin beta chain, brain 1
IPI00025499.3	MAPT	X	X	X			protein binding	Isoform Tau-F of Microtubule-associated protein tau
IPI00442344.4	SLC27A1	X	X		membrane,mitochondrion,endoplasmic reticulum,cytoplasm	transport,metabolic process,regulation of biological process,response to stimulus,cell communication	protein binding,transporter activity,nucleotide binding,catalytic activity	Long-chain fatty acid transport protein 1
IPI00022331.1	LCAT			X	extracellular	cell organization and biogenesis,metabolic process,transport,regulation of biological process,response to stimulus	protein binding,catalytic activity	Phosphatidylcholine-sterol acyltransferase
IPI00470475.4	PLCXD3	X	X			metabolic process,regulation of biological process,response to stimulus,cell communication	catalytic activity	cDNA FLJ45199 fis, clone BRCAN2003814, highly similar to Homo sapiens phosphatidylinositol-specific phospholipase C, X domain containing 3 (PLCXD3), mRNA
IPI00220706.10	HBG1	X	X	X	cytoplasm,cytosol	transport,response to stimulus,coagulation	transporter activity,metal ion binding	Hemoglobin subunit gamma-1
IPI00305442.3	UBAC1		X		membrane,cytoplasm,Golgi	metabolic process	protein binding	Ubiquitin-associated domain-containing protein 1
IPI00012887.1	CTSL1			X	extracellular,cytoplasm,vacuole,organelle lumen,nucleus,endosome	cell death,metabolic process,response to stimulus,defense response	protein binding,catalytic activity	Cathepsin L1
IPI00294519.2	TEP1			X	cytoplasm,organelle lumen,chromosome,nucleus	cell organization and biogenesis,metabolic process	protein binding,RNA binding,nucleotide binding,catalytic activity	Isoform 1 of Telomerase protein component 1
IPI00419263.4	ECI2	X	X			metabolic process	catalytic activity	enoyl-CoA delta isomerase 2, mitochondrial isoform 1
IPI00940451.1	IGHM			X			protein binding	59 kDa protein
IPI00045219.2	SNX18			X	membrane,cytoplasm,endosome	metabolic process,transport,regulation of biological process,cell communication	protein binding	Isoform 1 of Sorting nexin-18
IPI00641172.3	LAMC3			X				172 kDa protein
IPI00017745.1	TIMP4			X	extracellular,cytoplasm	development,metabolic process,regulation of biological process,response to stimulus,reproduction	protein binding,metal ion binding,enzyme regulator activity	Metalloproteinase inhibitor 4
IPI00746681.1	FAM201B			X				Similar to Bcl-2-related ovarian killer protein
IPI00872363.3	PTPRD			X		metabolic process	protein binding,catalytic activity	receptor-type tyrosine-protein phosphatase delta isoform 6 precursor
IPI00032902.5	SMPD3	X	X		membrane,cytoplasm,Golgi	development,transport,metabolic process,cell communication	metal ion binding,catalytic activity	Sphingomyelin phosphodiesterase 3
IPI00030207.1	GMDS	X	X		cytoplasm	metabolic process,regulation of biological process,response to stimulus,cell communication	nucleotide binding,catalytic activity	GDP-mannose 4,6 dehydratase
IPI00220156.1	TGFB2			X	extracellular	cell growth	protein binding	Isoform B of Transforming growth factor beta-2
IPI00290085.2	CDH2	X	X	X	membrane	cell organization and biogenesis,development,regulation of biological process,response to stimulus,cellular component movement,cell communication,cellular homeostasis,cell differentiation	protein binding,metal ion binding	Cadherin-2
IPI00306436.1	STAT3	X	X		nucleus	metabolic process,regulation of biological process,response to stimulus,cell communication	signal transducer activity,protein binding	Isoform Del-701 of Signal transducer and activator of transcription 3
IPI00444272.2	LIFR			X	extracellular,membrane	cell proliferation,cell organization and biogenesis,development,regulation of biological process,response to stimulus,cell communication,cell differentiation	signal transducer activity,protein binding,receptor activity	Leukemia inhibitory factor receptor
IPI00005516.1	LRRC4			X	membrane		protein binding	Leucine-rich repeat-containing protein 4
IPI00302176.5	GAR1	X	X		organelle lumen,nucleus	metabolic process	RNA binding,transporter activity,catalytic activity	Isoform 1 of H/ACA ribonucleoprotein complex subunit 1
IPI00003783.1	MAP2K2	X	X		extracellular,membrane,mitochondrion,cytoplasm,Golgi,cytosol,nucleus,endosome	development,cell organization and biogenesis,transport,metabolic process,regulation of biological process,response to stimulus,cellular component movement,cell communication,defense response,cell differentiation	protein binding,nucleotide binding,structural molecule activity,catalytic activity	Dual specificity mitogen-activated protein kinase kinase 2

IPI	GENE	Alzheimer's Hippocampus	Control hippocampus	CSF	Cellular localization	Biological process	Molecular function	Protein Description
IPI00003102.1	CNTFR	X	X	X	extracellular,membrane	cell death,cell proliferation,development,regulation of biological process,response to stimulus,cell communication,reproduction	signal transducer activity,protein binding,receptor activity	Ciliary neurotrophic factor receptor subunit alpha
IPI00031109.4	NDUFAF2		X		membrane,mitochondrion,cytoplasm		catalytic activity	Mimitin, mitochondrial
IPI00479083.4	C10orf137			X			protein binding	erythroid differentiation-related factor 1 (EDRF1)
IPI00028122.1	PSIP1	X	X		cytoplasm,organelle lumen,chromosome,cytosol,nucleus	cell organization and biogenesis,metabolic process,regulation of biological process,response to stimulus,reproduction	protein binding,DNA binding,metal ion binding	Isoform 1 of PC4 and SFRS1-interacting protein
IPI00013162.4	CD200	X	X	X	membrane	regulation of biological process,response to stimulus	protein binding	Isoform 1 of OX-2 membrane glycoprotein
IPI00019427.4	EXOC1	X	X		membrane,cytoplasm	transport		Isoform 1 of Exocyst complex component 1
IPI00013808.1	ACTN4	X	X		extracellular,cytoskeleton,cytoplasm,organelle lumen,nucleus	cell death,cell organization and biogenesis,transport,regulation of biological process,response to stimulus,cellular component movement,coagulation	protein binding,metal ion binding	Alpha-actinin-4
IPI00002496.2	GMPPB	X	X			metabolic process	catalytic activity	Isoform 2 of Mannose-1-phosphate guanyltransferase beta
IPI00291578.7	PRPSAP1	X	X			metabolic process,regulation of biological process	metal ion binding,catalytic activity,enzyme regulator activity	Isoform 1 of Phosphoribosyl pyrophosphate synthase-associated protein 1
IPI00000024.2	PCDH1			X	membrane	development,cell communication	metal ion binding	Isoform 1 of Protocadherin-1
IPI00854644.3	IGKV3D-20			X				Ig kappa chain V-III region Ti
IPI00643034.3	PLTP			X				cDNA FLJ39690 fis, clone SMINT2010639, highly similar to PHOSPHOLIPID TRANSFER PROTEIN
IPI00001433.1	PCDHB15			X	membrane	development	metal ion binding	Protocadherin beta-15
IPI00930442.1	IGHG4			X	membrane		protein binding	Putative uncharacterized protein DKFZp686M24218
IPI00023505.4	FCGR2A			X	membrane		protein binding,receptor activity	Isoform 1 of Low affinity immunoglobulin gamma Fc region receptor II-a
IPI00297626.4	STXBP3	X	X		membrane,cytoplasm,organelle lumen,cytosol,nucleus	transport,regulation of biological process,response to stimulus,coagulation	protein binding	Syntaxin-binding protein 3
IPI00472676.1	HLA-B			X	membrane,endoplasmic reticulum,cytoplasm,Golgi,endosome	regulation of biological process,response to stimulus,cell communication,defense response	protein binding	HLA class I histocompatibility antigen, B-42 alpha chain
IPI00002245.4	ACSS3		X		mitochondrion,cytoplasm	metabolic process	nucleotide binding,catalytic activity	Isoform 1 of Acyl-CoA synthetase short-chain family member 3, mitochondrial
IPI00023605.1	CDC42EP1	X			cytoskeleton,membrane,cytoplasm,Golgi	cell organization and biogenesis,regulation of biological process,response to stimulus,cell communication	protein binding	Isoform 1 of Cdc42 effector protein 1
IPI00178352.6	FLNC	X	X	X	cytoskeleton,membrane,cytoplasm,cytosol	cell organization and biogenesis	protein binding	Isoform 1 of Filamin-C
IPI00298738.3	POLRMT			X		metabolic process	DNA binding,catalytic activity	Similar to DNA-directed RNA polymerase, mitochondrial precursor
IPI00304840.4	COL6A2			X	extracellular,membrane	development,cell organization and biogenesis,response to stimulus,cell differentiation	protein binding,structural molecule activity	Isoform 2C2 of Collagen alpha-2(VI) chain
IPI00010491.3	RAB27B	X	X		membrane,cytoplasm,Golgi,nucleus,endosome	metabolic process,transport,regulation of biological process,response to stimulus,cell communication	protein binding,nucleotide binding,catalytic activity	Ras-related protein Rab-27B
IPI00003817.3	ARHGDIB	X	X	X	cytoskeleton,cytoplasm,cytosol	cell organization and biogenesis,development,metabolic process,regulation of biological process,response to stimulus,cellular component movement,cell communication	enzyme regulator activity	Rho GDP-dissociation inhibitor 2
IPI00791971.1	NME1		X			metabolic process	nucleotide binding,catalytic activity	9 kDa protein
IPI00219575.5	BLMH			X	cytoplasm,cytosol,nucleus	metabolic process,response to stimulus	protein binding,catalytic activity	Bleomycin hydrolase
IPI00021405.3	LMNA	X	X		cytoskeleton,cytoplasm,organelle lumen,nucleus	cell death,development,cell organization and biogenesis,transport,metabolic process,regulation of biological process,response to stimulus,cellular component movement,cell communication,cell differentiation,reproduction	protein binding,structural molecule activity	Isoform A of Prelamin-A/C
IPI00795055.1	C1RL			X		metabolic process	catalytic activity	cDNA FLJ14022 fis, clone HEMBA1003538, weakly similar to COMPLEMENT C1R COMPONENT
IPI00166071.3	BCL6B			X	nucleus	metabolic process,regulation of biological process	protein binding,DNA binding,metal ion binding	B-cell CLL/lymphoma 6 member B protein
IPI00027139.1	INPP1	X	X			metabolic process,regulation of biological process,response to stimulus,cell communication	metal ion binding,catalytic activity	Inositol polyphosphate 1-phosphatase

IPI	GENE	Alzheimer's Hippocampus	Control hippocampus	CSF	Cellular localization	Biological process	Molecular function	Protein Description
IPI00478809.4	F5			X	extracellular,membrane,cytoplasm,organelle lumen	transport,response to stimulus,coagulation	protein binding,metal ion binding	Coagulation factor V
IPI00027547.2	DCD		X	X	extracellular	metabolic process,response to stimulus,defense response	catalytic activity	Dermcidin
IPI00063130.2	TMEM205	X	X		membrane			Transmembrane protein 205
IPI00105598.3	PSMD11	X	X		proteasome		protein binding	Proteasome 26S non-ATPase subunit 11 variant (Fragment)
IPI00784154.1	HSPD1	X	X	X	extracellular,cell surface,membrane,mitochondrion,cytoplasm,organelle lumen,nucleus,cytosol,endosome	cell death,cell proliferation,cell organization and biogenesis,development,metabolic process,regulation of biological process,response to stimulus,defense response,cell communication	protein binding,DNA binding,nucleotide binding,catalytic activity	60 kDa heat shock protein, mitochondrial
IPI00299435.3	APOF			X	extracellular	transport,metabolic process	protein binding,transporter activity	Apolipoprotein F
IPI00027310.5	MEGF8			X	membrane		protein binding,metal ion binding,structural molecule activity	Isoform 1 of Multiple epidermal growth factor-like domains protein 8
IPI00028091.3	ACTR3	X	X		cytoskeleton,membrane,cytoplasm,Golgi	development,cell organization and biogenesis,cell division,regulation of biological process,response to stimulus,cellular component movement,cell differentiation,reproduction	protein binding,nucleotide binding	Actin-related protein 3
IPI00031169.1	RAB2A	X	X		membrane,endoplasmic reticulum,cytoplasm,Golgi,nucleus	transport,metabolic process,regulation of biological process,response to stimulus,cell communication	nucleotide binding,catalytic activity	Ras-related protein Rab-2A
IPI00374337.1	GRID1	X	X		membrane	transport,regulation of biological process,response to stimulus,cell communication	signal transducer activity,transporter activity,receptor activity	Glutamate receptor delta-1 subunit
IPI00019407.1	NSDHL	X			membrane,endoplasmic reticulum,cytoplasm	development,metabolic process,regulation of biological process,response to stimulus,cell communication	nucleotide binding,catalytic activity	Sterol-4-alpha-carboxylate 3-dehydrogenase, decarboxylating
IPI00374862.1	KLHL5			X	cytoskeleton,cytoplasm		protein binding	Isoform 1 of Kelch-like protein 5
IPI00218343.4	TUBA1C		X		cytoskeleton,cytoplasm	cell organization and biogenesis,cell division,transport,metabolic process,cellular component movement	nucleotide binding,structural molecule activity,catalytic activity	Tubulin alpha-1C chain
IPI00019591.2	C2		X	X		metabolic process	protein binding,catalytic activity	cDNA FLJ55673, highly similar to Complement factor B
IPI00001712.2	CTNNA3			X	cytoskeleton,cytoplasm		protein binding,structural molecule activity	Isoform 1 of Catenin alpha-3
IPI00009329.2	UTRN	X	X		cytoskeleton,membrane,cytoplasm,organelle lumen,nucleus	development,regulation of biological process	protein binding,metal ion binding	Utrophin
IPI00290279.1	ADK	X	X		cytoplasm,cytosol,nucleus	cell proliferation,metabolic process,regulation of biological process	metal ion binding,nucleotide binding,catalytic activity	Isoform Long of Adenosine kinase
IPI00011126.6	PSMC1	X	X		proteasome,cytoplasm,organelle lumen,nucleus,cytosol	cell death,metabolic process,regulation of biological process,response to stimulus,cell communication	protein binding,RNA binding,nucleotide binding,catalytic activity	26S protease regulatory subunit 4
IPI00329536.2	EEA1	X	X		cytoskeleton,membrane,cytoplasm,cytosol,endosome	cell organization and biogenesis,transport,cell communication	protein binding,metal ion binding,motor activity,catalytic activity	Early endosome antigen 1
IPI00215919.5	ARF5	X	X		membrane,cytoplasm,Golgi,nucleus	transport,metabolic process,regulation of biological process,response to stimulus,cell communication	protein binding,signal transducer activity,nucleotide binding,catalytic activity	ADP-ribosylation factor 5
IPI00843765.1	SPTAN1	X	X				protein binding	Isoform 3 of Spectrin alpha chain, brain
IPI00216288.1	L3MBTL1			X	nucleus	metabolic process,regulation of biological process	metal ion binding	Isoform 3 of Lethal(3)malignant brain tumor-like protein 1
IPI00152326.3	GSTM1			X			protein binding	Isoform 2 of Glutathione S-transferase Mu 1
IPI00791493.1	PLXNA2			X	membrane	development,regulation of biological process,response to stimulus,cell communication	protein binding,receptor activity	plexin A2
IPI00973531.1	IGLC2			X			protein binding	Putative uncharacterized protein
IPI00018387.1	FURIN			X	extracellular,cell surface,membrane,endoplasmic reticulum,cytoplasm,Golgi,organelle lumen	cell proliferation,cell organization and biogenesis,metabolic process,transport,regulation of biological process,response to stimulus,cellular component movement,cell communication,reproduction	protein binding,metal ion binding,catalytic activity,enzyme regulator activity	Furin
IPI00013860.3	HIBADH	X	X	X	mitochondrion,cytoplasm,organelle lumen	metabolic process	nucleotide binding,catalytic activity	3-hydroxyisobutyrate dehydrogenase, mitochondrial
IPI00164528.1	SNTA1	X	X		cytoskeleton,membrane,cytoplasm	cell organization and biogenesis,development,cell differentiation	protein binding	Alpha-1-syntrophin
IPI00243451.7	SLCO1B7			X	membrane	transport	protein binding,transporter activity	liver-specific organic anion transporter 3TM12
IPI00301994.6	FAHD2B	X	X			metabolic process	metal ion binding,catalytic activity	Fumarylacetoacetate hydrolase domain-containing protein 2B

IPI	GENE	Alzheimer's Hippocampus	Control hippocampus	CSF	Cellular localization	Biological process	Molecular function	Protein Description
IPI00028055.4	TMED10	X	X		membrane,endoplasmic reticulum,cytoplasm,Golgi	cell organization and biogenesis,metabolic process,transport	protein binding	Transmembrane emp24 domain-containing protein 10
IPI00329582.6	MFSD4		X		membrane	transport		Major facilitator superfamily domain-containing protein 4
IPI00024417.1	HIP1R	X	X		cytoskeleton,mitochondrion,membrane,cytoplasm	cell organization and biogenesis,transport	protein binding,motor activity,catalytic activity	Huntingtin-interacting protein 1-related protein
IPI00375957.3	COMMD6		X		cytoplasm,nucleus	metabolic process,regulation of biological process	protein binding	Isoform 1 of COMM domain-containing protein 6
IPI00399076.2	TNFAIP8L3		X					Tumor necrosis factor alpha-induced protein 8-like protein 3
IPI00412988.1	NTNG1			X			protein binding	Isoform 1 of Netrin-G1
IPI00018027.2	AGGF1			X	extracellular,cytoplasm	cell proliferation,development,metabolic process,regulation of biological process,cell differentiation	protein binding	Isoform 1 of Angiogenic factor with G patch and FHA domains 1
IPI00027258.3	SYNPO	X	X		cytoskeleton,membrane	cell organization and biogenesis	protein binding	Isoform 2 of Synaptopodin
IPI00022145.6	NUCKS1	X	X		nucleus			Isoform 1 of Nuclear ubiquitous casein and cyclin-dependent kinases substrate
IPI00300207.3	C4orf21			X			metal ion binding	prematurely terminated mRNA decay factor-like
IPI00215979.3	BPGM	X	X	X		development,metabolic process,cell differentiation	catalytic activity	Bisphosphoglycerate mutase
IPI00748265.2	IGHV2-26			X				Rheumatoid factor RF-ET13
IPI00064193.3	TMX3		X		membrane,endoplasmic reticulum,cytoplasm	metabolic process,regulation of biological process,cellular homeostasis	metal ion binding,catalytic activity	Isoform 1 of Protein disulfide-isomerase TMX3
IPI00473047.3	PRKAG1	X	X		cytoplasm,organelle lumen,cytosol,nucleus	cell organization and biogenesis,metabolic process,regulation of biological process,response to stimulus,cell communication,reproduction	protein binding,nucleotide binding,catalytic activity,enzyme regulator activity	5'-AMP-activated protein kinase subunit gamma-1
IPI00290308.2	TRIB1			X	cytoplasm,nucleus	cell proliferation,metabolic process,regulation of biological process,response to stimulus,cellular component movement,cell communication	protein binding,nucleotide binding,catalytic activity,enzyme regulator activity	Tribbles homolog 1
IPI00418735.2 2	C17orf97			X				Isoform 2 of Uncharacterized protein C17orf97
IPI00298994.6	TLN1	X	X	X	extracellular,cytoskeleton,membrane,cytoplasm,cytosol	cell organization and biogenesis,development,metabolic process,transport,regulation of biological process,response to stimulus,cellular component movement,cell communication,cell differentiation,coagulation	protein binding,structural molecule activity	Talin-1
IPI00015897.2	CHORDC1	X	X			cell organization and biogenesis,metabolic process,regulation of biological process,response to stimulus	protein binding,metal ion binding	Isoform 1 of Cysteine and histidine-rich domain-containing protein 1
IPI00465123.6	AP5Z1			X	organelle lumen,nucleus	metabolic process,regulation of biological process	DNA binding	KIAA0415 gene product
IPI00006173.3	CETP			X	extracellular	cell organization and biogenesis,metabolic process,transport,regulation of biological process,cell differentiation	transporter activity	Isoform 1 of Cholesteryl ester transfer protein
IPI00045396.3	CALU		X				metal ion binding	Isoform 2 of Calumenin
IPI00215768.3	GCLC	X	X		cytoplasm,cytosol	cell death,metabolic process,regulation of biological process,response to stimulus,cellular homeostasis	protein binding,metal ion binding,nucleotide binding,catalytic activity	Glutamate--cysteine ligase catalytic subunit
IPI00008404.1	DVL1			X	cytoskeleton,membrane,cytoplasm	cell organization and biogenesis,development,metabolic process,transport,regulation of biological process,response to stimulus,cell communication,cell growth,cell differentiation	signal transducer activity,protein binding	Isoform Long of Segment polarity protein dishevelled homolog DVL-1-like
IPI00182438.1	CNTN5			X			protein binding	Isoform 2 of Contactin-5
IPI00294739.1	SAMHD1	X	X		nucleus	metabolic process,regulation of biological process,response to stimulus,defense response	protein binding,metal ion binding,catalytic activity	Isoform 1 of SAM domain and HD domain-containing protein 1
IPI00217948.7	FRMD4B			X	cytoskeleton			Uncharacterized protein
IPI00186295.5	SLC38A3	X	X		membrane	transport	transporter activity	Sodium-coupled neutral amino acid transporter 3

IPI	GENE	Alzheimer's Hippocampus	Control hippocampus	CSF	Cellular localization	Biological process	Molecular function	Protein Description
IPI00027505.2	ITGAV	X	X		cell surface,membrane,cytoplasm	cell death,cell organization and biogenesis,development,metabolic process,regulation of biological process,response to stimulus,reproduction,cell proliferation,transport,cellular component movement,cell communication,cell differentiation,coagulation	protein binding,transporter activity,receptor activity	Isoform 1 of Integrin alpha-V
IPI00028911.2	DAG1	X	X	X	extracellular,cytoskeleton,membrane,cytoplasm,organelle lumen,nucleus	cell organization and biogenesis,transport,metabolic process,regulation of biological process,response to stimulus,cellular component movement,cell communication,reproduction	protein binding,metal ion binding,receptor activity,structural molecule activity	Dystroglycan
IPI00165360.5	MPST	X	X		mitochondrion,membrane,cytoplasm	metabolic process,response to stimulus	catalytic activity	3-mercaptopyruvate sulfurtransferase
IPI00002334.2	NSG1			X	membrane	regulation of biological process,response to stimulus,cell communication	protein binding	DNA segment on chromosome 4 (Unique) 234 expressed sequence, isoform CRA_a
IPI00479523.6	TRIO	X	X		membrane	metabolic process,regulation of biological process,response to stimulus,cell communication	protein binding,nucleotide binding,catalytic activity,enzyme regulator activity	Isoform 2 of Triple functional domain protein
IPI00026665.2	QARS	X	X	X	cytoplasm	metabolic process	nucleotide binding,catalytic activity	glutaminyl-tRNA synthetase, isoform CRA_b
IPI00009943.2	TPT1	X	X	X	extracellular,cytoskeleton,cytoplasm,endosome	cell death,transport,regulation of biological process,cellular homeostasis,cell differentiation	metal ion binding	Tumor protein, translationally-controlled 1
IPI00745056.1	Sep-03	X	X				nucleotide binding,catalytic activity	Isoform 2 of Neuronal-specific septin-3
IPI00056478.1	IGSF8	X	X	X	membrane	cell proliferation,development,cellular component movement,reproduction	protein binding	Isoform 1 of Immunoglobulin superfamily member 8
IPI00022371.1	HRG			X	extracellular,membrane,cytoplasm,organelle lumen	cell death,cell organization and biogenesis,development,metabolic process,regulation of biological process,response to stimulus,defense response,cell proliferation,transport,cellular component movement,cell communication,cell growth,coagulation	protein binding,metal ion binding,enzyme regulator activity	Histidine-rich glycoprotein
IPI00334907.3	PITPNB	X	X		cytoplasm,Golgi	development,metabolic process,transport	transporter activity	Isoform 1 of Phosphatidylinositol transfer protein beta isoform
IPI00289924.4	ST8SIA5			X	membrane,cytoplasm,Golgi	metabolic process	catalytic activity	Alpha-2,8-sialyltransferase 8E
IPI00332106.2	PBXIP1	X	X		cytoskeleton,cytoplasm,cytosol,nucleus	development,metabolic process,regulation of biological process,cell differentiation	protein binding	Isoform 1 of Pre-B-cell leukemia transcription factor-interacting protein 1
IPI00032402.1	ATP8A1	X	X		membrane,endoplasmic reticulum,cytoplasm	cell organization and biogenesis,transport	protein binding,transporter activity,metal ion binding,nucleotide binding,catalytic activity	Isoform 1 of Probable phospholipid-transporting ATPase IA
IPI00003848.1	DNAJB4		X		membrane,cytoplasm,organelle lumen,nucleus	metabolic process,response to stimulus	protein binding	DnaJ homolog subfamily B member 4
IPI00022200.4	COL6A3	X	X	X	extracellular,membrane	cell organization and biogenesis,development,metabolic process,regulation of biological process,response to stimulus,cell differentiation	protein binding,enzyme regulator activity	Isoform 1 of Collagen alpha-3(VI) chain
IPI00304082.8	ISOC1	X	X		cytoplasm	metabolic process	catalytic activity	Isochorismatase domain-containing protein 1
IPI00022640.1	NRGN	X	X	X		development,regulation of biological process,response to stimulus,cell communication	protein binding	Neurogranin
IPI00015972.1	COX6C		X		membrane,mitochondrion,cytoplasm	metabolic process	transporter activity,catalytic activity	Cytochrome c oxidase subunit 6C
IPI00056357.3	C19orf10	X	X	X	extracellular,endoplasmic reticulum,cytoplasm,organelle lumen	metabolic process,regulation of biological process,response to stimulus,cell communication		UPF0556 protein C19orf10
IPI00029400.2	ZRANB2	X	X		nucleus	metabolic process,regulation of biological process	protein binding,RNA binding,metal ion binding	Isoform 1 of Zinc finger Ran-binding domain-containing protein 2
IPI00006166.1	GPR37			X	membrane,endoplasmic reticulum,cytoplasm	regulation of biological process,response to stimulus,cell communication	signal transducer activity,receptor activity	Probable G-protein coupled receptor 37
IPI00019502.3	MYH9	X	X	X	cytoskeleton,membrane,mitochondrion,cytoplasm,nucleus,cytosol	development,cell organization and biogenesis,metabolic process,cell division,transport,regulation of biological process,response to stimulus,cellular component movement,cell communication,cell differentiation,reproduction	protein binding,transporter activity,motor activity,nucleotide binding,catalytic activity	Isoform 1 of Myosin-9
IPI00005128.1	ANGPT2			X	extracellular,membrane,nucleus	development,regulation of biological process,response to stimulus,cellular component movement,cell communication,reproduction,cell differentiation,coagulation	protein binding,metal ion binding,catalytic activity	Isoform 1 of Angiopoietin-2
IPI00337351.4	MDGA2			X	membrane			MAM domain-containing glycosylphosphatidylinositol anchor protein 2 isoform 1

IPI	GENE	Alzheimer's Hippocampus	Control hippocampus	CSF	Cellular localization	Biological process	Molecular function	Protein Description
IPI00410093.2	CCDC69			X				Coiled-coil domain-containing protein 69
IPI00170814.1	PTK7			X		metabolic process	nucleotide binding,catalytic activity	Isoform 2 of Inactive tyrosine-protein kinase 7
IPI00647556.2	GSN	X	X					gelsolin isoform c
IPI00607600.1	APLP1			X			metal ion binding	Isoform 2 of Amyloid-like protein 1
IPI00030871.2	VNN1			X	membrane	cell death,development,metabolic process,regulation of biological process,response to stimulus,cellular component movement,defense response,cell differentiation	catalytic activity	Pantetheinase
IPI00073958.1	CNRIP1	X	X					Isoform 1 of CB1 cannabinoid receptor-interacting protein 1
IPI00013847.4	UQCRC1	X	X	X	membrane,mitochondrion,cytoplasm	transport,metabolic process,response to stimulus	protein binding,transporter activity,metal ion binding,catalytic activity	Cytochrome b-c1 complex subunit 1, mitochondrial
IPI00027799.2	FAM107B	X	X					Isoform 2 of Protein FAM107B
IPI00784842.1	IGHV4-31			X	membrane		protein binding	Putative uncharacterized protein DKFZp686G11190
IPI00289807.4	TRNT1	X	X		cytoskeleton,mitochondrion,cytoplasm	cell organization and biogenesis,transport,metabolic process	RNA binding,nucleotide binding,catalytic activity	Isoform 1 of CCA tRNA nucleotidyltransferase 1, mitochondrial
IPI00250724.4	FAM69C			X			catalytic activity	31 kDa protein
IPI00218075.1	FAM9B			X	organelle lumen,nucleus			Protein FAM9B
IPI00291006.2	MDH2	X	X	X	membrane,mitochondrion,cytoplasm,organelle lumen,nucleus	metabolic process	protein binding,nucleotide binding,catalytic activity	Malate dehydrogenase, mitochondrial
IPI00003856.1	ATP6V1E1	X	X		membrane,mitochondrion,cytoplasm,cytosol,endosome	transport,regulation of biological process,response to stimulus,cell communication,cellular homeostasis	protein binding,transporter activity,catalytic activity	V-type proton ATPase subunit E 1
IPI00031820.3	FARSA	X	X		cytoplasm,cytosol	metabolic process	protein binding,RNA binding,nucleotide binding,catalytic activity	Phenylalanyl-tRNA synthetase alpha chain
IPI00440493.2	ATP5A1	X	X	X	mitochondrion,membrane,cytoplasm,organelle lumen,nucleus	cell proliferation,development,metabolic process,transport,regulation of biological process	protein binding,transporter activity,nucleotide binding,catalytic activity	ATP synthase subunit alpha, mitochondrial
IPI00000138.1	MGAT1			X	membrane,cytoplasm,Golgi	metabolic process	catalytic activity	MGAT1 protein
IPI00910712.1	TPM2	X	X					cDNA FLJ57036, highly similar to Homo sapiens tropomyosin 2 (beta) (TPM2), transcript variant 2, mRNA
IPI00026359.4	CAB39L	X	X		cytoplasm,cytosol	metabolic process,regulation of biological process,response to stimulus,cell communication		Calcium-binding protein 39-like
IPI00217081.1	FUNDC1	X	X		membrane,mitochondrion,cytoplasm	metabolic process,response to stimulus	protein binding	FUN14 domain-containing protein 1
IPI00479654.2	ISLR2			X	cell surface,membrane	cell organization and biogenesis,development,regulation of biological process,cell growth,cell differentiation	protein binding	Immunoglobulin superfamily containing leucine-rich repeat protein 2
IPI00005123.1	EFNA3			X	membrane	regulation of biological process,response to stimulus,cell communication	protein binding,signal transducer activity,receptor activity,catalytic activity	Ephrin-A3
IPI00303963.1	C2			X	extracellular	metabolic process,regulation of biological process,response to stimulus,defense response	protein binding,catalytic activity	Complement C2 (Fragment)
IPI00845229.1	PREX2			X		regulation of biological process,response to stimulus,cell communication	protein binding,enzyme regulator activity	Isoform 2 of Phosphatidylinositol 3,4,5-trisphosphate-dependent Rac exchanger 2 protein
IPI00152050.1	RBFOX1			X				Isoform 5 of RNA binding protein fox-1 homolog 1
IPI00012202.1	WDR77	X			cytoplasm,Golgi,organelle lumen,cytosol,nucleus	cell proliferation,cell organization and biogenesis,development,metabolic process,regulation of biological process,reproduction,cell differentiation	protein binding	Methylosome protein 50
IPI00219622.3	PSMA2	X	X	X	cytoplasm,proteasome,organelle lumen,nucleus,cytosol	cell death,metabolic process,regulation of biological process,response to stimulus,cell communication	protein binding,catalytic activity	Proteasome subunit alpha type-2
IPI00026050.1	CLN5			X	extracellular,membrane,cytoplasm,organelle lumen,vacuole	development,cell organization and biogenesis,metabolic process,response to stimulus,cellular homeostasis,cell differentiation		ceroid-lipofuscinosis neuronal protein 5
IPI00783024.1	LOC652113			X			protein binding	Myosin-reactive immunoglobulin heavy chain variable region (Fragment)
IPI00006971.3	CD248			X	extracellular,membrane,cytoplasm,organelle lumen,nucleus	metabolic process	protein binding,metal ion binding	Isoform 1 of Endosialin
IPI00816555.4	IGLC3			X				V1-4 protein

IPI	GENE	Alzheimer's Hippocampus	Control hippocampus	CSF	Cellular localization	Biological process	Molecular function	Protein Description
IPI00022240.4	ISCU		X		mitochondrion,cytoplasm,nucleus,cytosol	cell organization and biogenesis,metabolic process	protein binding,metal ion binding,structural molecule activity	Isoform 1 of Iron-sulfur cluster assembly enzyme ISCU, mitochondrial
IPI00152426.1	SFXN5	X	X		membrane,mitochondrion,cytoplasm	transport	transporter activity	Sideroflexin-5
IPI00025341.2	BDH1	X	X		membrane,mitochondrion,cytoplasm,organelle lumen,nucleus	development,metabolic process,response to stimulus	nucleotide binding,catalytic activity	D-beta-hydroxybutyrate dehydrogenase, mitochondrial
IPI00023513.1	CHFR			X	organelle lumen,nucleus	cell organization and biogenesis,cell division,metabolic process,regulation of biological process	protein binding,metal ion binding,nucleotide binding,catalytic activity	Isoform 1 of E3 ubiquitin-protein ligase CHFR
IPI00794184.1	CP			X		metabolic process	metal ion binding,catalytic activity	cDNA FLJ37971 fis, clone CTONG2009958, highly similar to CERULOPLASMIN
IPI00165125.6	C14orf37			X				cDNA FLJ59350
IPI00332493.6	PLEKHO2	X	X				protein binding	Isoform 1 of Pleckstrin homology domain-containing family O member 2
IPI00006114.4	SERPINF1			X			enzyme regulator activity	serpin peptidase inhibitor, clade F (alpha-2 antiplasmin, pigment epithelium derived factor), member 1
IPI00420014.2	SNRNP200			X	spliceosomal complex,organelle lumen,nucleus	cell organization and biogenesis,metabolic process	protein binding,nucleotide binding,catalytic activity	Isoform 1 of U5 small nuclear ribonucleoprotein 200 kDa helicase
IPI00418169.3	ANXA2			X			protein binding,metal ion binding,enzyme regulator activity	Isoform 2 of Annexin A2
IPI00013281.1	FKRP			X	extracellular,membrane,endoplasmic reticulum,cytoplasm,Golgi	metabolic process	catalytic activity	Fukutin-related protein
IPI00515034.2	KIFAP3	X	X		cytoskeleton	cell proliferation,regulation of biological process	protein binding	kinesin-associated protein 3 isoform 4
IPI00418240.1	NEBL		X				protein binding,metal ion binding	nebulette isoform 2
IPI00414984.3	SGCE			X	membrane		metal ion binding	epsilon-sarcoglycan isoform 1
IPI00028053.2	GJA9			X	membrane	cell communication		Isoform 1 of Gap junction alpha-9 protein
IPI00009809.1	PEX5L		X		membrane,cytoplasm,cytosol	cell organization and biogenesis,transport,regulation of biological process,response to stimulus,cell communication,cellular homeostasis	protein binding,transporter activity	Isoform 1 of PEX5-related protein
IPI00470766.14	OLFML2B			X	extracellular	cell organization and biogenesis	protein binding	Isoform 1 of Olfactomedin-like protein 2B
IPI00216313.7	VSNL1	X	X				metal ion binding	Visinin-like protein 1
IPI00409669.1	CD163			X	membrane		receptor activity	CD163 molecule
IPI01012577.1	GLB1			X	cytoplasm,organelle lumen,vacuole	metabolic process	protein binding,catalytic activity	Isoform 1 of Beta-galactosidase
IPI00374740.3	CD47	X	X		extracellular,membrane	cell proliferation,cell organization and biogenesis,transport,regulation of biological process,response to stimulus,cellular component movement,defense response,cell communication,coagulation	protein binding,signal transducer activity,receptor activity	Isoform OA3-323 of Leukocyte surface antigen CD47
IPI00419595.2	PODXL2	X		X	membrane	cellular component movement	protein binding	Isoform 1 of Podocalyxin-like protein 2
IPI00016645.3	EPHA7			X	membrane	cell death,cell organization and biogenesis,development,metabolic process,regulation of biological process,response to stimulus,cell communication,cell differentiation	protein binding,signal transducer activity,nucleotide binding,receptor activity,catalytic activity	Isoform 1 of Ephrin type-A receptor 7
IPI00006444.1	SLC24A2			X	membrane	transport,regulation of biological process,response to stimulus,cell communication,cellular homeostasis	protein binding,transporter activity	Isoform 1 of Sodium/potassium/calcium exchanger 2
IPI00217225.7	NOS1		X		cytoskeleton,membrane,endoplasmic reticulum,cytoplasm,cytosol	development,transport,metabolic process,regulation of biological process,response to stimulus,cell communication,cellular homeostasis,cell differentiation,coagulation	protein binding,metal ion binding,nucleotide binding,catalytic activity	Isoform 1 of Nitric oxide synthase, brain
IPI00005102.3	SMS		X		cytoplasm,cytosol	metabolic process	catalytic activity	Isoform 1 of Spermine synthase
IPI00171176.3	PANK2		X		mitochondrion,cytoplasm,organelle lumen,nucleus	cell death,metabolic process	nucleotide binding,catalytic activity	Isoform 1 of Pantothenate kinase 2, mitochondrial
IPI00171459.4	HSDL1	X	X		mitochondrion,cytoplasm	metabolic process	nucleotide binding,catalytic activity	Inactive hydroxysteroid dehydrogenase-like protein 1
IPI00448465.5	ANKRD12			X	cytoplasm,organelle lumen,nucleus		protein binding	Isoform 1 of Ankyrin repeat domain-containing protein 12
IPI00853045.1	IGKC			X			protein binding	Anti-RhD monoclonal T125 kappa light chain
IPI00032498.1	DCTN5	X	X		cytoskeleton,cytoplasm		catalytic activity	Dynactin subunit 5

IPI	GENE	Alzheimer's Hippocampus	Control hippocampus	CSF	Cellular localization	Biological process	Molecular function	Protein Description
IPI00794450.1	LYNX1			X	membrane			9 kDa protein
IPI00306413.3	TPPP3	X	X	X	cytoskeleton,cytoplasm	cell organization and biogenesis	protein binding,metal ion binding	Tubulin polymerization-promoting protein family member 3
IPI00418497.1	TIMM50	X	X				protein binding	Isoform 2 of Mitochondrial import inner membrane translocase subunit TIM50
IPI00018963.4	PARVA	X	X				protein binding	alpha-parvin
IPI00640981.3	UBR4	X	X				metal ion binding,catalytic activity	Isoform 4 of E3 ubiquitin-protein ligase UBR4
IPI00013179.1	PTGDS	X	X	X	extracellular,membrane,endoplasmic reticulum,cytoplasm,Golgi,nucleus	transport,metabolic process,regulation of biological process,response to stimulus	transporter activity,catalytic activity	Prostaglandin-H2 D-isomerase
IPI00221089.5	RPS13	X	X		cytoplasm,ribosome,organelle lumen,nucleus,cytosol	cell organization and biogenesis,transport,metabolic process,regulation of biological process,reproduction	protein binding,RNA binding,structural molecule activity	40S ribosomal protein S13
IPI00012545.2	TGOLN2			X	membrane,cytoplasm,Golgi,organelle lumen,nucleus		protein binding	Isoform TGN51 of Trans-Golgi network integral membrane protein 2
IPI00218465.1 0	PLAA	X	X	X		metabolic process,regulation of biological process,response to stimulus,cell communication,defense response	protein binding,enzyme regulator activity	Phospholipase A-2-activating protein
IPI00396435.3	DHX15	X	X		spliceosomal complex,organelle lumen,nucleus	metabolic process	nucleotide binding,catalytic activity	Putative pre-mRNA-splicing factor ATP-dependent RNA helicase DHX15
IPI00018946.4	PANK4	X	X			metabolic process	nucleotide binding,catalytic activity	cDNA FLJ56439, highly similar to Pantothenate kinase 4
IPI00292071.6	SCG3	X	X	X	extracellular,membrane,cytoplasm	transport,response to stimulus,coagulation		Secretogranin-3
IPI00021329.3	WDR45L	X			cytoplasm	cell organization and biogenesis,metabolic process,response to stimulus,cell communication	protein binding	WD repeat domain phosphoinositide-interacting protein 3
IPI00657687.1	ANLN	X	X		cytoskeleton,cytoplasm,nucleus	cell organization and biogenesis,cell division,regulation of biological process	protein binding	actin-binding protein anillin
IPI00412987.1 0	GMFB	X	X	X			protein binding	Glia maturation factor, beta
IPI00005677.1	GNPAT		X		mitochondrion,membrane,cytoplasm,organelle lumen	cell organization and biogenesis,development,metabolic process,response to stimulus,cell communication,cellular homeostasis	protein binding,catalytic activity	Dihydroxyacetone phosphate acyltransferase
IPI00006713.1	DNAJC3			X	membrane,endoplasmic reticulum,cytoplasm,organelle lumen	metabolic process,regulation of biological process,response to stimulus,cell communication	protein binding,enzyme regulator activity	DnaJ homolog subfamily C member 3
IPI00152900.2	LZIC	X	X				protein binding	cDNA FLJ55829, highly similar to Homo sapiens leucine zipper and CTNNBIP1 domain containing (LZIC), mRNA
IPI00002236.4	MFGE8			X				cDNA FLJ59612, highly similar to Lactadherin
IPI00152733.1	OLFML2A			X			protein binding	FLJ00237 protein (Fragment)
IPI00791228.1	GRIA4			X				glutamate receptor 4 isoform 3 precursor
IPI00455535.1	LOC100652743			X			protein binding	PREDICTED: similar to Ig kappa chain V-III region VH precursor
IPI00021807.3	GBA		X	X	membrane,cytoplasm,vacuole	cell organization and biogenesis,metabolic process	catalytic activity	cDNA FLJ56157, highly similar to Glucosylceramidase
IPI00798272.2	BCAT1		X			metabolic process	catalytic activity	branched-chain-amino-acid aminotransferase, cytosolic isoform 2
IPI00295988.4	PROM2		X		extracellular,membrane			Isoform 1 of Prominin-2
IPI00815926.1	IGHG1			X			protein binding	IGHG1 protein
IPI00020436.4	RAB11B	X	X		membrane,mitochondrion,cytoplasm	transport,metabolic process,regulation of biological process,response to stimulus,cell communication	nucleotide binding,catalytic activity	Ras-related protein Rab-11B
IPI00384861.3	GIT1	X	X	X	membrane,cytoplasm	metabolic process,regulation of biological process,response to stimulus,cell communication	protein binding,metal ion binding,enzyme regulator activity	Isoform 1 of ARF GTPase-activating protein GIT1
IPI00179589.4	MTPN	X	X	X			protein binding	14 kDa protein
IPI00001159.1 1	GCN1L1	X	X		cytoplasm,ribosome	metabolic process,regulation of biological process	protein binding,RNA binding	Translational activator GCN1
IPI00015148.3	RAP1B	X	X	X	membrane,cytoplasm,cytosol	cell proliferation,metabolic process,transport,regulation of biological process,response to stimulus,cell communication,coagulation	protein binding,nucleotide binding,catalytic activity	Ras-related protein Rap-1b
IPI00218570.6	PGAM2	X	X	X	cytoplasm,cytosol,nucleus	metabolic process,response to stimulus,reproduction	catalytic activity	Phosphoglycerate mutase 2
IPI00218046.6	HS6ST3			X	membrane		catalytic activity	Heparan-sulfate 6-O-sulfotransferase 3
IPI00002283.9	PTCHD2			X	membrane	transport	signal transducer activity,transporter activity,receptor activity	Uncharacterized protein

IPI	GENE	Alzheimer's Hippocampus	Control hippocampus	CSF	Cellular localization	Biological process	Molecular function	Protein Description
IPI00017533.2	COX3		X		mitochondrion,membrane,cytoplasm	metabolic process	transporter activity,catalytic activity	Cytochrome c oxidase subunit 3
IPI00177008.1	PGP	X	X			metabolic process	catalytic activity	Phosphoglycolate phosphatase
IPI00025094.3	MYH16			X	cytoskeleton,membrane,organelle lumen,chromosome,nucleus	cell organization and biogenesis,reproduction	motor activity,catalytic activity	cDNA: FLJ22037 fis, clone HEP08868 (Fragment)
IPI00009054.1	BMP1			X	extracellular	development,metabolic process,regulation of biological process,cell differentiation	protein binding,metal ion binding,catalytic activity	Isoform BMP1-3 of Bone morphogenetic protein 1
IPI00217629.3	WDR47		X				protein binding	Isoform 1 of WD repeat-containing protein 47
IPI00026044.3	PIGU		X		membrane,endoplasmic reticulum,cytoplasm	metabolic process,regulation of biological process,response to stimulus,cell communication	catalytic activity	Isoform 1 of Phosphatidylinositol glycan anchor biosynthesis class U protein
IPI00011274.3	HNRPDL	X	X		cytoplasm,nucleus	metabolic process,regulation of biological process	DNA binding,RNA binding,nucleotide binding	Isoform 1 of Heterogeneous nuclear ribonucleoprotein D-like
IPI00028004.2	PSMB3	X	X		cytoplasm,proteasome,organelle lumen,nucleus,cytosol	cell death,metabolic process,regulation of biological process,response to stimulus,cell communication	catalytic activity	Proteasome subunit beta type-3
IPI00216049.1	HNRNPK			X	spliceosomal complex,cytoplasm,organelle lumen,chromosome,nucleus	cell organization and biogenesis,metabolic process,transport,regulation of biological process,response to stimulus,cell communication	protein binding,DNA binding,RNA binding	Isoform 1 of Heterogeneous nuclear ribonucleoprotein K
IPI00333126.1	LRRC56			X			protein binding	Leucine-rich repeat-containing protein 56
IPI00044369.2	PLXDC2			X	membrane		protein binding	Isoform 1 of Plexin domain-containing protein 2
IPI00028369.6	KIAA1715	X	X		membrane	development,response to stimulus,coagulation		Isoform 1 of Protein lunapark
IPI01010912.1	MMP28			X		metabolic process	metal ion binding,catalytic activity	matrix metallopeptidase 28, isoform CRA_b
IPI00220644.8	PKM	X	X	X		metabolic process	metal ion binding,catalytic activity	Isoform M1 of Pyruvate kinase isozymes M1/M2
IPI00645089.2	KCNIP1			X	extracellular	regulation of biological process,response to stimulus,cell communication	transporter activity,metal ion binding	Isoform 4 of Kv channel-interacting protein 1
IPI00553138.4	VAMP2	X	X	X	membrane,cytoplasm	cell organization and biogenesis,metabolic process,transport,regulation of biological process,cell communication	protein binding	Vesicle-associated membrane protein 2
IPI00419575.6	GET4		X					Uncharacterised protein family UPF0363 protein
IPI00216461.5	ACYP2	X	X	X				Acylphosphatase-2
IPI00219077.4	LTA4H	X	X	X	cytoplasm,organelle lumen,cytosol,nucleus	development,metabolic process,response to stimulus,defense response,cell differentiation	metal ion binding,catalytic activity	Isoform 1 of Leukotriene A-4 hydrolase
IPI00413451.3	SERPINB6			X				Serpin B6
IPI00022774.3	VCP	X	X	X	membrane,endoplasmic reticulum,cytoplasm,proteasome,organelle lumen,nucleus,cytosol	cell death,cell organization and biogenesis,transport,metabolic process,regulation of biological process,response to stimulus,cell communication,reproduction	protein binding,RNA binding,nucleotide binding,catalytic activity	Transitional endoplasmic reticulum ATPase
IPI00296830.5	LUZP1	X			nucleus			Isoform 1 of Leucine zipper protein 1
IPI00002511.2	ATF6			X	membrane,endoplasmic reticulum,cytoplasm,Golgi,organelle lumen,nucleus	metabolic process,regulation of biological process,response to stimulus,cell communication	protein binding,DNA binding	Cyclic AMP-dependent transcription factor ATF-6 alpha
IPI00402293.4	ARSG			X	extracellular,endoplasmic reticulum,cytoplasm,vacuole	metabolic process	metal ion binding,catalytic activity	Arylsulfatase G
IPI00337612.2	DCBLD1			X	membrane			Isoform 1 of Discoidin, CUB and LCCL domain-containing protein 1
IPI00297444.4	CD177			X	membrane	response to stimulus,cellular component movement,coagulation		Isoform 1 of CD177 antigen
IPI00830035.1	IGKV1D-13			X				13 kDa protein
IPI00301419.2	COPS7A		X		cytoplasm,organelle lumen,nucleus	metabolic process	protein binding	COP9 signalosome complex subunit 7a
IPI00296635.5	GBE1	X	X			metabolic process	catalytic activity	1,4-alpha-glucan-branching enzyme
IPI00029046.1	MLEC	X	X	X	membrane,endoplasmic reticulum,cytoplasm	metabolic process		Malectin
IPI00218875.1	SPP1			X				Isoform C of Osteopontin
IPI00025809.2	MGAT2			X	membrane,cytoplasm,Golgi	metabolic process	catalytic activity	cDNA FLJ55952, highly similar to Alpha-1,6-mannosyl-glycoprotein2-beta-N- acetylglucosaminyltransferase
IPI00021900.1	TNFSF12			X	extracellular,membrane,cytoplasm	cell death,cell proliferation,development,regulation of biological process,response to stimulus,cellular component movement,cell communication,cell differentiation	protein binding	Tumor necrosis factor ligand superfamily member 12

IPI	GENE	Alzheimer's Hippocampus	Control hippocampus	CSF	Cellular localization	Biological process	Molecular function	Protein Description
IPI00015285.7	PCYT2	X	X	X		metabolic process	catalytic activity	Ethanolamine-phosphate cytidylyltransferase
IPI00000877.1	HYOU1	X	X	X	endoplasmic reticulum,cytoplasm,organelle lumen	metabolic process,regulation of biological process,response to stimulus,cell communication	nucleotide binding	Hypoxia up-regulated protein 1
IPI00025091.3	RPS11	X	X		cytoplasm,ribosome,cytosol	cell organization and biogenesis,metabolic process,transport,reproduction	RNA binding,structural molecule activity	40S ribosomal protein S11
IPI00015351.2	PITHD1	X	X	X	extracellular	metabolic process,regulation of biological process,response to stimulus		Isoform 1 of PITH domain-containing protein 1
IPI00937974.2	ALAD	X	X			metabolic process	metal ion binding,catalytic activity	Delta-aminolevulinic acid dehydratase
IPI00003362.3	HSPA5	X	X	X	cell surface,membrane,endoplasmic reticulum,cytoplasm,organelle lumen,nucleus,cytosol	cell death,cell organization and biogenesis,development,metabolic process,transport,regulation of biological process,response to stimulus,cell communication,coagulation	protein binding,metal ion binding,nucleotide binding,catalytic activity,enzyme regulator activity	78 kDa glucose-regulated protein
IPI00015865.6	ADPRHL2	X	X		cytoplasm,organelle lumen,nucleus		metal ion binding,catalytic activity	Poly(ADP-ribose) glycohydrolase ARH3
IPI00796647.2	HIGD1C			X	membrane			HIG1 domain family member 1C
IPI00167592.3	C19orf66		X					Isoform 2 of UPF0515 protein C19orf66
IPI00291866.5	SERPING1	X	X	X	extracellular,cytoplasm,organelle lumen	transport,metabolic process,regulation of biological process,response to stimulus,defense response,coagulation	protein binding,enzyme regulator activity	Plasma protease C1 inhibitor
IPI00025340.3	PDXP	X	X		cytoskeleton,membrane,cytoplasm,cytosol	cell organization and biogenesis,metabolic process,cell division,regulation of biological process,response to stimulus	protein binding,metal ion binding,catalytic activity	Pyridoxal phosphate phosphatase
IPI00102896.1	RAB2B	X	X		membrane,endoplasmic reticulum,cytoplasm,Golgi,nucleus	transport,regulation of biological process,response to stimulus,cell communication	nucleotide binding	Ras-related protein Rab-2B
IPI00017672.4	PNP	X	X			metabolic process	catalytic activity	cDNA FLJ25678 fis, clone TST04067, highly similar to PURINE NUCLEOSIDE PHOSPHORYLASE
IPI00045841.5	LRP11			X	membrane		protein binding,receptor activity	Isoform 1 of Low-density lipoprotein receptor-related protein 11
IPI00017344.3	RAB5B	X	X		membrane,cytoplasm,nucleus,endosome	metabolic process,transport,regulation of biological process,response to stimulus,cell communication	protein binding,nucleotide binding,catalytic activity	Ras-related protein Rab-5B
IPI00017567.3	ENG			X	extracellular,cell surface,membrane,cytoplasm,organelle lumen,nucleus	cell proliferation,development,cell organization and biogenesis,metabolic process,regulation of biological process,response to stimulus,cellular component movement,cell communication,cell differentiation	signal transducer activity,protein binding,receptor activity,catalytic activity	Isoform Long of Endoglin
IPI00010333.1	MDK			X	extracellular	development,metabolic process,cell division,regulation of biological process,response to stimulus,cell communication,defense response,cell differentiation	protein binding	Midkine
IPI00008504.1	CA14			X	membrane		metal ion binding,catalytic activity	Carbonic anhydrase 14
IPI00296176.2	F9			X	extracellular,membrane,endoplasmic reticulum,cytoplasm,Golgi,organelle lumen	metabolic process,response to stimulus,coagulation	protein binding,metal ion binding,catalytic activity	Coagulation factor IX
IPI00301143.5	PI16	X		X	extracellular,membrane	metabolic process,regulation of biological process	enzyme regulator activity	Isoform 1 of Peptidase inhibitor 16
IPI00104341.4	EPHX2	X	X			metabolic process	catalytic activity	cDNA FLJ59619, highly similar to Epoxide hydrolase 2
IPI00021907.2	MBP			X	membrane	development,response to stimulus,cell communication,cellular homeostasis	structural molecule activity	Isoform 1 of Myelin basic protein
IPI00334715.4	ARHGAP35	X	X		membrane,cytoplasm,cytosol,nucleus	cell organization and biogenesis,development,transport,metabolic process,regulation of biological process,response to stimulus,cell communication,cell differentiation	DNA binding,transporter activity,nucleotide binding,enzyme regulator activity	Isoform 1 of Rho GTPase-activating protein 35
IPI00304409.3	CARHSP1	X	X		cytoplasm,cytosol	metabolic process,regulation of biological process,response to stimulus,cell communication	protein binding,DNA binding,RNA binding	Calcium-regulated heat stable protein 1
IPI00176581.5	FANCM			X	organelle lumen,nucleus	metabolic process,response to stimulus,reproduction	protein binding,DNA binding,nucleotide binding,catalytic activity	Isoform 1 of Fanconi anemia group M protein
IPI00152001.2	TRIM3		X		cytoplasm,nucleus,endosome	development,transport	protein binding,metal ion binding,catalytic activity	Isoform Alpha of Tripartite motif-containing protein 3
IPI00022937.4	F5			X			metal ion binding	252 kDa protein
IPI00018465.1	CCT7	X	X		cytoskeleton,mitochondrion,cytoplasm,cytosol	metabolic process	protein binding,nucleotide binding	T-complex protein 1 subunit eta
IPI00026546.1	PAFAH1B2	X	X	X	cytoplasm	metabolic process,regulation of biological process,response to stimulus,cell communication	catalytic activity	Platelet-activating factor acetylhydrolase IB subunit beta

IPI	GENE	Alzheimer's Hippocampus	Control hippocampus	CSF	Cellular localization	Biological process	Molecular function	Protein Description
IPI00742944.2	FMN2	X	X		membrane	cell organization and biogenesis,transport,regulation of biological process,response to stimulus,cell communication	protein binding	195 kDa protein
IPI00786937.1	LOC731940			X	membrane		receptor activity	similar to deleted in malignant brain tumors 1 isoform b precursor
IPI00644388.2	PNMAL2		X					Isoform 1 of PNMA-like protein 2
IPI00395627.3	CACYBP	X	X		cytoplasm,nucleus	cell death,development,metabolic process,regulation of biological process,response to stimulus,cell differentiation	protein binding	Isoform 1 of Calcyclin-binding protein
IPI00040730.4	CDHR1			X	membrane	cell organization and biogenesis	metal ion binding,receptor activity	Isoform 1 of Cadherin-related family member 1
IPI00030111.1	GDF11			X	extracellular	cell proliferation,development,regulation of biological process,cell differentiation	protein binding	Growth/differentiation factor 11
IPI00171737.6	PIDD			X		regulation of biological process,response to stimulus,cell communication	protein binding	Isoform 2 of p53-induced protein with a death domain
IPI00790021.1	ZNF652			X	nucleus	metabolic process,regulation of biological process	protein binding,DNA binding,metal ion binding	Zinc finger protein 652
IPI00303161.3	ESAM			X	membrane	response to stimulus,cellular component movement,coagulation	protein binding	Endothelial cell-selective adhesion molecule
IPI00008215.1	ME1	X	X	X	mitochondrion,cytoplasm,cytosol	cell organization and biogenesis,metabolic process,response to stimulus	metal ion binding,nucleotide binding,catalytic activity	NADP-dependent malic enzyme
IPI00013917.3	RPS12	X	X		cytoplasm,ribosome,cytosol	cell organization and biogenesis,transport,metabolic process,reproduction	structural molecule activity	40S ribosomal protein S12
IPI00644127.2	IARS		X		cytoplasm,organelle lumen,cytosol,nucleus	metabolic process,regulation of biological process	protein binding,nucleotide binding,catalytic activity	Isoleucyl-tRNA synthetase, cytoplasmic
IPI00376587.8	ANKDD1B			X		regulation of biological process,response to stimulus,cell communication	protein binding	Ankyrin repeat and death domain-containing protein 1B
IPI00019495.1	DYNLT1	X	X		cytoskeleton,cytoplasm,Golgi	development,cell organization and biogenesis,cell division,metabolic process,transport,regulation of biological process,response to stimulus,cell communication,cell differentiation,reproduction	protein binding,motor activity,catalytic activity	Dynein light chain Tctex-type 1
IPI00446874.3	ACAD8	X	X			metabolic process	catalytic activity	cDNA FLJ90159 fis, clone HEMBB1002465, weakly similar to ACYL-COA DEHYDROGENASE
IPI00010790.1	BGN	X	X	X	extracellular,cell surface,membrane,cytoplasm	metabolic process	protein binding,structural molecule activity	Biglycan
IPI00018769.4	THBS2			X	extracellular	cell organization and biogenesis,development,regulation of biological process,cell communication	protein binding,metal ion binding	Thrombospondin-2
IPI00442909.1	IGHV4-31			X			protein binding	CDNA FLJ26301 fis, clone DMC07540
IPI00005776.1	NOD1			X	membrane,cytoplasm,cytosol	cell death,cell organization and biogenesis,metabolic process,regulation of biological process,response to stimulus,defense response,cell communication	protein binding,nucleotide binding,enzyme regulator activity	Nucleotide-binding oligomerization domain-containing protein 1
IPI00219817.2	MAPK8IP3		X		extracellular,cytoskeleton,membrane,mitochondrion,cytoplasm,nucleus	transport	protein binding,motor activity,catalytic activity	C-Jun-amino-terminal kinase-interacting protein 3 isoform 2
IPI00003443.3	IFI16	X			cytoplasm,organelle lumen,nucleus	cell death,cell proliferation,development,metabolic process,regulation of biological process,response to stimulus,cell communication,cell differentiation	protein binding,DNA binding	Isoform 1 of Gamma-interferon-inducible protein 16
IPI00456635.3	UNC13D			X	membrane		protein binding	Isoform 3 of Protein unc-13 homolog D
IPI00383032.1	HAVCR2			X				Isoform 2 of Hepatitis A virus cellular receptor 2
IPI00018274.1	EGFR			X	extracellular,membrane,endoplasmic reticulum,cytoplasm,Golgi,nucleus,endosome	cell death,cell proliferation,cell organization and biogenesis,development,transport,metabolic process,regulation of biological process,response to stimulus,cellular component movement,cell communication,cell differentiation	protein binding,signal transducer activity,DNA binding,nucleotide binding,receptor activity,catalytic activity,enzyme regulator activity	Isoform 1 of Epidermal growth factor receptor
IPI00795481.2	LYPD1			X	membrane			Isoform 1 of Ly6/PLAUR domain-containing protein 1
IPI00015522.2	GDF5			X	extracellular	cell death,cell proliferation,development,regulation of biological process,response to stimulus,cell communication,cell differentiation	protein binding	Growth/differentiation factor 5
IPI00021770.1	HMGCR			X	membrane,endoplasmic reticulum,cytoplasm	cell organization and biogenesis,metabolic process	signal transducer activity,nucleotide binding,receptor activity,catalytic activity	Isoform 1 of 3-hydroxy-3-methylglutaryl-coenzyme A reductase
IPI00008998.3	PTPLAD1	X	X	X	membrane,endoplasmic reticulum,cytoplasm	metabolic process,regulation of biological process,response to stimulus,cell communication	catalytic activity,enzyme regulator activity	3-hydroxyacyl-CoA dehydratase 3
IPI00456604.1	FAM19A1			X	extracellular,endoplasmic reticulum,cytoplasm			Protein FAM19A1
IPI00789477.1	LTF			X	extracellular,cytoplasm	transport,cellular homeostasis	metal ion binding,catalytic activity	lactotransferrin isoform 2

IPI	GENE	Alzheimer's Hippocampus	Control hippocampus	CSF	Cellular localization	Biological process	Molecular function	Protein Description
IPI00000736.1	TSPAN15	X			membrane			Tetraspanin-15
IPI00024317.1	GCDH		X		membrane,mitochondrion,cytoplasm,organelle lumen	metabolic process	catalytic activity	Isoform Long of Glutaryl-CoA dehydrogenase, mitochondrial
IPI00018195.3	MAPK3	X	X		cytoskeleton,membrane,mitochondrion,cytoplasm,Golgi,organelle lumen,cytosol,nucleus,endosome	cell death,cell organization and biogenesis,development,transport,metabolic process,regulation of biological process,response to stimulus,defense response,cell communication,cell differentiation,coagulation	signal transducer activity,protein binding,nucleotide binding,catalytic activity	Mitogen-activated protein kinase 3
IPI00018879.2	IDUA			X	cytoplasm,vacuole	cell organization and biogenesis,development,metabolic process	catalytic activity	Alpha-L-iduronidase
IPI00215997.5	CD9	X	X	X	cell surface,membrane,cytoplasm	cell proliferation,development,cell organization and biogenesis,transport,regulation of biological process,response to stimulus,cellular component movement,cell communication,cellular homeostasis,reproduction,cell differentiation,coagulation	protein binding	CD9 antigen
IPI00006114.5	SERPINF1	X	X	X	extracellular,cytoplasm	cell proliferation,development,metabolic process,regulation of biological process,response to stimulus,defense response,reproduction,cell differentiation	enzyme regulator activity	Pigment epithelium-derived factor
IPI00023343.4	DLG3	X	X		cytoskeleton,membrane,cytoplasm	cell proliferation,cell organization and biogenesis,development,metabolic process,regulation of biological process,response to stimulus,cell communication,cell differentiation	protein binding,catalytic activity	Isoform 1 of Disks large homolog 3
IPI00140420.4	SND1	X	X		mitochondrion,cytoplasm,nucleus	metabolic process,regulation of biological process	catalytic activity	Staphylococcal nuclease domain-containing protein 1
IPI00218918.5	ANXA1	X	X	X	extracellular,cytoskeleton,membrane,mitochondrion,cytoplasm,nucleus	cell death,cell organization and biogenesis,development,metabolic process,regulation of biological process,response to stimulus,defense response,reproduction,cell proliferation,transport,cellular component movement,cell communication,cell differentiation	protein binding,DNA binding,RNA binding,metal ion binding,structural molecule activity,enzyme regulator activity	Annexin A1
IPI00217540.7	KDM1A		X			metabolic process	protein binding,catalytic activity	Isoform 2 of Lysine-specific histone demethylase 1A
IPI00219729.3	SLC25A11	X	X		mitochondrion,membrane,cytoplasm	metabolic process,transport	transporter activity	Mitochondrial 2-oxoglutarate/malate carrier protein
IPI00456623.2	BCAN	X	X	X	extracellular,membrane	development,regulation of biological process,cell communication	protein binding	Isoform 1 of Brevican core protein
IPI00856045.1	AHNAK2	X	X		nucleus		protein binding	Isoform 1 of Protein AHNAK2
IPI00170692.4	VAPA	X	X	X	membrane,endoplasmic reticulum,cytoplasm	cell death,cell organization and biogenesis,development,metabolic process,regulation of biological process,response to stimulus,cell communication,cell differentiation	protein binding,signal transducer activity,structural molecule activity	Isoform 1 of Vesicle-associated membrane protein-associated protein A
IPI00065500.3	BROX	X	X		membrane			BRO1 domain-containing protein BROX
IPI00334627.3	ANXA2P2	X	X		extracellular,membrane,cytoplasm,endosome	development,cell organization and biogenesis,transport,regulation of biological process,response to stimulus,coagulation	protein binding,metal ion binding,enzyme regulator activity	Putative annexin A2-like protein
IPI00398918.6	NCR3LG1			X	membrane		protein binding,structural molecule activity	B7 homolog 6
IPI00031357.1	PPOX		X		mitochondrion,membrane,cytoplasm	metabolic process,response to stimulus	nucleotide binding,catalytic activity	Protoporphyrinogen oxidase
IPI00333067.3	HERC4	X	X		cytoplasm,cytosol,nucleus	metabolic process,cell differentiation,reproduction	catalytic activity	Isoform 1 of Probable E3 ubiquitin-protein ligase HERC4
IPI00398795.4	RTN3	X	X		membrane,endoplasmic reticulum,cytoplasm	cell organization and biogenesis,regulation of biological process		Isoform 2 of Reticulon-3
IPI00297412.4	CADPS	X	X		membrane,cytoplasm,cytosol	cell organization and biogenesis,transport,regulation of biological process,cell communication	protein binding,metal ion binding	Isoform 1 of Calcium-dependent secretion activator 1
IPI00220281.3	GNAO1	X	X	X	membrane	cell organization and biogenesis,development,metabolic process,transport,regulation of biological process,response to stimulus,cell communication,cell differentiation	protein binding,signal transducer activity,metal ion binding,nucleotide binding,catalytic activity	Isoform Alpha-1 of Guanine nucleotide-binding protein G(o) subunit alpha
IPI00010700.3	PRRC2A		X		cytoplasm,nucleus		protein binding	Isoform 1 of Protein PRRC2A
IPI00012902.3	SYT7	X	X		membrane,cytoplasm,vacuole	cell organization and biogenesis,transport,regulation of biological process,response to stimulus,cell communication	protein binding,transporter activity,metal ion binding	Synaptotagmin-7
IPI00029647.3	ZG16			X	extracellular,membrane,cytoplasm,Golgi,organelle lumen	transport		Zymogen granule membrane protein 16

IPI	GENE	Alzheimer's Hippocampus	Control hippocampus	CSF	Cellular localization	Biological process	Molecular function	Protein Description
IPI00006091.3	DMD	X	X		cell surface,cytoskeleton,mitochondrion,membrane,cytoplasm,nucleus,cytosol	development,cell organization and biogenesis,metabolic process,regulation of biological process,cellular component movement,cell communication,cellular homeostasis,cell differentiation	protein binding,metal ion binding,structural molecule activity	Isoform 4 of Dystrophin
IPI00215637.5	DDX3X	X	X		cytoplasm,organelle lumen,nucleus		DNA binding,RNA binding,nucleotide binding,catalytic activity	ATP-dependent RNA helicase DDX3X
IPI00018909.3	TFF3			X	extracellular,cytoplasm	response to stimulus		Trefoil factor 3
IPI00299503.2	GPLD1			X	extracellular,membrane,cytoplasm	cell death,cell proliferation,development,metabolic process,transport,regulation of biological process,response to stimulus,cellular component movement,cell communication,cellular homeostasis,cell differentiation	catalytic activity	Isoform 1 of Phosphatidylinositol-glycan-specific phospholipase D
IPI00218413.2	BTD			X	extracellular,organelle lumen,nucleus	development,metabolic process	catalytic activity	Biotinidase
IPI00299116.3	PODXL	X		X	membrane	regulation of biological process,cellular component movement	catalytic activity	Podocalyxin-like isoform 2 precursor
IPI00031019.1	CST8			X	extracellular,cell surface,cytoplasm	metabolic process,regulation of biological process	enzyme regulator activity	Cystatin-8
IPI00288947.3	GNAQ	X	X		membrane,cytoplasm	development,transport,metabolic process,regulation of biological process,response to stimulus,cell communication,cellular homeostasis,cell differentiation,coagulation	signal transducer activity,protein binding,metal ion binding,nucleotide binding,catalytic activity,enzyme regulator activity	Guanine nucleotide-binding protein G(q) subunit alpha
IPI00219682.6	STOM	X	X	X	cytoskeleton,membrane,cytoplasm	cell organization and biogenesis		Erythrocyte band 7 integral membrane protein
IPI00845432.1	NADKD1	X	X		mitochondrion,cytoplasm	metabolic process,regulation of biological process,response to stimulus,cell communication	catalytic activity	Isoform 1 of NAD kinase domain-containing protein 1
IPI00221234.7	ALDH7A1	X	X		mitochondrion,cytoplasm,organelle lumen,cytosol,nucleus	metabolic process	protein binding,catalytic activity	Isoform 1 of Alpha-aminoadipic semialdehyde dehydrogenase
IPI00060181.1	EFHD2	X	X		membrane		metal ion binding	EF-hand domain-containing protein D2
IPI00419565.4	STAB1			X	membrane	development,transport,regulation of biological process,response to stimulus,cell communication,defense response	protein binding,receptor activity,catalytic activity	Isoform 1 of Stabilin-1
IPI00465128.4	BAG6	X	X		cytoplasm,cytosol,nucleus	cell death,cell proliferation,cell organization and biogenesis,development,transport,metabolic process,regulation of biological process,response to stimulus,cell communication,cell differentiation,reproduction	protein binding	Isoform 1 of Large proline-rich protein BAG6
IPI00221222.7	SUB1	X	X		organelle lumen,nucleus	metabolic process,regulation of biological process	protein binding,DNA binding	Activated RNA polymerase II transcriptional coactivator p15
IPI00640292.4	VWA7			X	extracellular		protein binding	Isoform 1 of Protein G7c
IPI00219512.2	UCHL5		X			metabolic process	catalytic activity	Isoform 2 of Ubiquitin carboxyl-terminal hydrolase isozyme L5
IPI00179109.2	SIRT2	X	X		cytoskeleton,cytoplasm,nucleus	development,cell organization and biogenesis,metabolic process,cell division,regulation of biological process,response to stimulus	protein binding,metal ion binding,nucleotide binding,catalytic activity	Isoform 1 of NAD-dependent deacetylase sirtuin-2
IPI00029623.1	PSMA6	X	X	X	mitochondrion,cytoplasm,proteasome,organelle lumen,cytosol,nucleus	cell death,metabolic process,regulation of biological process,response to stimulus,cell communication,defense response	protein binding,RNA binding,nucleotide binding,catalytic activity	Proteasome subunit alpha type-6
IPI00012066.2	PCBP2	X	X				RNA binding	poly(rC)-binding protein 2 isoform b
IPI00847179.1	APOA4			X	extracellular,cytoplasm	cell organization and biogenesis,transport,metabolic process		apolipoprotein A-IV precursor
IPI00018396.1	CBLN4			X	extracellular	transport	protein binding	Cerebellin-4
IPI00291987.1	IGFBPL1			X	extracellular	cell organization and biogenesis,regulation of biological process,cell growth	protein binding	Insulin-like growth factor-binding protein-like 1
IPI00647650.3	EIF3H	X			cytoplasm	metabolic process	protein binding,RNA binding	cDNA FLJ35809 fis, clone TESTI2006016, highly similar to Eukaryotic translation initiation factor 3 subunit 3
IPI00220473.2	ATP2C1		X			metabolic process	metal ion binding,nucleotide binding,catalytic activity	Isoform 2 of Calcium-transporting ATPase type 2C member 1
IPI00007961.4	ND1	X			membrane,mitochondrion,cytoplasm	transport,metabolic process,response to stimulus	protein binding,catalytic activity	NADH-ubiquinone oxidoreductase chain 1
IPI00011051.1	TLX1			X	nucleus	cell proliferation,development,metabolic process,regulation of biological process,cell differentiation	protein binding,DNA binding	T-cell leukemia homeobox protein 1
IPI00031556.7	U2AF2	X	X		spliceosomal complex,organelle lumen,nucleus	metabolic process,transport,regulation of biological process	protein binding,RNA binding,nucleotide binding	Isoform 1 of Splicing factor U2AF 65 kDa subunit

IPI	GENE	Alzheimer's Hippocampus	Control hippocampus	CSF	Cellular localization	Biological process	Molecular function	Protein Description
IPI00032293.1	CST3		X	X	extracellular	cell organization and biogenesis,development,metabolic process,regulation of biological process,response to stimulus,defense response	protein binding,enzyme regulator activity	Cystatin-C
IPI00001541.1	TIMM9		X		membrane,mitochondrion,cytoplasm	cell organization and biogenesis,metabolic process,transport	protein binding,transporter activity,metal ion binding	Mitochondrial import inner membrane translocase subunit Tim9
IPI00019912.3	HSD17B4	X	X		membrane,cytoplasm,organelle lumen	metabolic process	protein binding,nucleotide binding,catalytic activity	Peroxisomal multifunctional enzyme type 2
IPI00294910.5	PARM1			X	membrane,cytoplasm,Golgi,nucleus,endosome	metabolic process,regulation of biological process		Prostate androgen-regulated mucin-like protein 1
IPI00019600.3	UBE2V2	X	X	X	cytoplasm,nucleus	cell death,cell proliferation,cell organization and biogenesis,development,metabolic process,regulation of biological process,response to stimulus,cell communication,cell differentiation	protein binding,catalytic activity	Ubiquitin-conjugating enzyme E2 variant 2
IPI00028006.1	PSMB2	X	X		cytoplasm,proteasome,organelle lumen,nucleus,cytosol	cell death,metabolic process,regulation of biological process,response to stimulus,cell communication	catalytic activity	Proteasome subunit beta type-2
IPI00063234.1	PRKAR2A	X	X			metabolic process,regulation of biological process,response to stimulus,cell communication	catalytic activity,enzyme regulator activity	Uncharacterized protein
IPI00922456.1	SH3GLB2	X	X		cytoplasm	cell organization and biogenesis,regulation of biological process,response to stimulus,cell communication	protein binding	Uncharacterized protein
IPI00059235.4	PNMA6B	X	X		cytoplasm			Paraneoplastic antigen-like protein 6B
IPI00328361.7	SARS2	X	X	X	cytoskeleton,membrane,mitochondrion,cytoplasm,organelle lumen	cell organization and biogenesis,metabolic process	nucleotide binding,catalytic activity	Seryl-tRNA synthetase, mitochondrial
IPI00289204.3	RTN4R			X	cell surface,membrane,endoplasmic reticulum,cytoplasm	cell organization and biogenesis,development,regulation of biological process,response to stimulus,cell communication,cell differentiation	protein binding,receptor activity	Reticulon-4 receptor
IPI00014581.1	TPM1	X	X		cytoskeleton,membrane,cytoplasm,cytosol	cell organization and biogenesis,development,metabolic process,regulation of biological process,response to stimulus,cellular component movement,cell differentiation	protein binding,structural molecule activity	Isoform 1 of Tropomyosin alpha-1 chain
IPI00784830.1	IGH@			X	membrane		protein binding	cDNA FLJ41981 fis, clone SMINT2011888, highly similar to Protein Tro alpha1 H,myeloma
IPI00218831.4	GSTM1	X	X		cytoplasm,cytosol	metabolic process,response to stimulus	protein binding,catalytic activity	Isoform 1 of Glutathione S-transferase Mu 1
IPI00024661.5	SEC24C	X	X		membrane,endoplasmic reticulum,cytoplasm,Golgi,organelle lumen,cytosol,nucleus	cell organization and biogenesis,transport,metabolic process,regulation of biological process	protein binding,DNA binding,metal ion binding	Protein transport protein Sec24C
IPI00217232.2	SUCLA2	X	X			metabolic process	catalytic activity	Isoform 2 of Succinyl-CoA ligase [ADP-forming] subunit beta, mitochondrial
IPI00302840.2	ATP1A3	X	X	X	membrane,endoplasmic reticulum,cytoplasm,Golgi	metabolic process,transport	transporter activity,metal ion binding,nucleotide binding,catalytic activity	Sodium/potassium-transporting ATPase subunit alpha-3
IPI00018246.5	HK1	X	X	X	membrane,mitochondrion,cytoplasm,organelle lumen,nucleus,cytosol	metabolic process,transport	protein binding,nucleotide binding,catalytic activity	Isoform 1 of Hexokinase-1
IPI00399328.5	KDM4E			X			protein binding	similar to jumonji domain containing 2D
IPI00008087.3	FSTL5			X	extracellular		protein binding,metal ion binding	Follistatin-related protein 5
IPI00023751.1	MSTN			X	extracellular,cytoplasm	development,metabolic process,regulation of biological process,response to stimulus,cell communication,reproduction	protein binding	Growth/differentiation factor 8
IPI00012198.4	C1orf50		X					Uncharacterized protein C1orf50
IPI00022793.5	HADHB	X	X		membrane,mitochondrion,endoplasmic reticulum,cytoplasm,organelle lumen	metabolic process	protein binding,nucleotide binding,catalytic activity	Trifunctional enzyme subunit beta, mitochondrial
IPI00795260.4	PAPLN			X	extracellular	metabolic process,regulation of biological process	protein binding,metal ion binding,catalytic activity,enzyme regulator activity	Isoform 1 of Papilin
IPI00295209.5	SNX5	X	X		membrane,cytoplasm,endosome	transport,cell communication	protein binding	Sorting nexin-5
IPI00639824.1	RIT1		X		membrane	metabolic process,regulation of biological process,response to stimulus,cell communication	nucleotide binding,catalytic activity	Ras-like without CAAX 1
IPI00789008.1	FLOT2	X	X		cell surface,membrane,cytoplasm,endosome	development		Flotillin-2
IPI00000824.2	NTRK3			X	membrane,cytoplasm,Golgi	cell death,development,cell organization and biogenesis,metabolic process,regulation of biological process,response to stimulus,cellular component movement,cell communication,cell growth,reproduction,cell differentiation	protein binding,signal transducer activity,nucleotide binding,receptor activity,catalytic activity	Isoform A of NT-3 growth factor receptor

IPI	GENE	Alzheimer's Hippocampus	Control hippocampus	CSF	Cellular localization	Biological process	Molecular function	Protein Description
IPI00004944.3	SLC4A10	X	X		membrane	transport	transporter activity	Isoform 1 of Sodium-driven chloride bicarbonate exchanger
IPI00457114.2	IQSEC1	X	X		cytoplasm,organelle lumen,nucleus	cell organization and biogenesis,transport,regulation of biological process,response to stimulus,cell communication	protein binding,enzyme regulator activity	Isoform 1 of IQ motif and SEC7 domain-containing protein 1
IPI00455510.4	PTMA	X	X					Isoform 2 of Prothymosin alpha
IPI00945308.2	TF			X	extracellular	transport,cellular homeostasis	protein binding,metal ion binding	Uncharacterized protein
IPI00000889.1	GH1			X	extracellular	cell organization and biogenesis,development,metabolic process,transport,regulation of biological process,response to stimulus,cell communication	protein binding,metal ion binding	Isoform 1 of Somatotropin
IPI00470625.2	NRN1		X	X	membrane			Neuritin
IPI00012645.2	SPTBN2	X	X		cytoskeleton,cytoplasm,cytosol	cell death,development,cell organization and biogenesis,transport,regulation of biological process,response to stimulus,cell differentiation	protein binding,structural molecule activity	Isoform 1 of Spectrin beta chain, brain 2
IPI00182757.10	KIAA1967	X	X		mitochondrion,cytoplasm,organelle lumen,nucleus	cell death,metabolic process,regulation of biological process	protein binding,enzyme regulator activity	Isoform 1 of Protein KIAA1967
IPI00218820.1	TPM2			X		regulation of biological process,response to stimulus,cell communication	structural molecule activity	Isoform 3 of Tropomyosin beta chain
IPI00783271.1	LRPPRC		X		cytoskeleton,mitochondrion,membrane,cytoplasm,organelle lumen,chromosome,nucleus	transport,metabolic process,regulation of biological process,cellular component movement	protein binding,DNA binding,RNA binding	Leucine-rich PPR motif-containing protein, mitochondrial
IPI00165393.1	ANP32E	X	X		cytoplasm,nucleus		protein binding,enzyme regulator activity	Acidic leucine-rich nuclear phosphoprotein 32 family member E
IPI00644409.2	GDA			X			catalytic activity	Uncharacterized protein
IPI00029631.1	ERH	X	X			metabolic process		Enhancer of rudimentary homolog
IPI00165972.3	CFD			X	extracellular	metabolic process,regulation of biological process,response to stimulus,cell communication	catalytic activity	Complement factor D preproprotein
IPI00099670.2	CEL			X	extracellular	metabolic process	catalytic activity	bile salt-activated lipase precursor
IPI00329057.8	FBXL18		X				protein binding	Isoform 1 of F-box/LRR-repeat protein 18
IPI00032597.2	RBMX2			X			RNA binding,nucleotide binding	RNA-binding motif protein, X-linked 2
IPI00299145.9	KRT6C			X	cytoskeleton	cell organization and biogenesis	motor activity,structural molecule activity,catalytic activity	Keratin, type II cytoskeletal 6C
IPI00291463.4	RSAD2			X	mitochondrion,membrane,endoplasmic reticulum,cytoplasm,Golgi	development,transport,metabolic process,regulation of biological process,response to stimulus,defense response,cell communication,cell differentiation,reproduction	protein binding,metal ion binding,catalytic activity	Radical S-adenosyl methionine domain-containing protein 2
IPI00018855.1	ATP6V0C		X		membrane,cytoplasm,vacuole,endosome	transport,regulation of biological process,response to stimulus,cell communication,cellular homeostasis	protein binding,transporter activity,catalytic activity	V-type proton ATPase 16 kDa proteolipid subunit
IPI00004845.4	NIPSNAP3A		X		cytoplasm,cytosol		protein binding	Protein NipSnap homolog 3A
IPI00023162.3	GNE			X	cytoplasm,cytosol	metabolic process	metal ion binding,nucleotide binding,catalytic activity	Isoform 1 of Bifunctional UDP-N-acetylglucosamine 2-epimerase/N-acetylmannosamine kinase
IPI00215948.4	CTNNA1	X	X		cytoskeleton,membrane,cytoplasm,cytosol	cell death,cell proliferation,cell organization and biogenesis,development,regulation of biological process,response to stimulus,cell communication,cell differentiation,reproduction	protein binding,structural molecule activity	Isoform 1 of Catenin alpha-1
IPI00455852.2	ARHGEF15			X	cytoplasm	development,cell organization and biogenesis,metabolic process,regulation of biological process,response to stimulus,cell communication	protein binding,enzyme regulator activity	Rho guanine nucleotide exchange factor 15
IPI00914566.2	FDPS	X	X		mitochondrion,cytoplasm,organelle lumen,cytosol,nucleus	metabolic process	metal ion binding,catalytic activity	Farnesyl pyrophosphate synthase
IPI00021808.3	HARS	X	X		cytoplasm,cytosol	metabolic process	nucleotide binding,catalytic activity	Histidyl-tRNA synthetase, cytoplasmic
IPI00329482.5	LAMA4			X	extracellular,cytoskeleton,membrane,cytoplasm,organelle lumen,chromosome,nucleus	development,cell organization and biogenesis,transport,regulation of biological process,cellular component movement,cell differentiation,reproduction	protein binding,motor activity,structural molecule activity,catalytic activity	Isoform 1 of Laminin subunit alpha-4
IPI00397949.3	GPR56			X	membrane	regulation of biological process,response to stimulus,cell communication	signal transducer activity,receptor activity	Isoform 2 of G-protein coupled receptor 56
IPI00001893.3	PCDH7	X	X	X	membrane		metal ion binding	Isoform A of Protocadherin-7

IPI	GENE	Alzheimer's Hippocampus	Control hippocampus	CSF	Cellular localization	Biological process	Molecular function	Protein Description
IPI00332371.9	PFKL	X	X		cytoplasm,cytosol	cell organization and biogenesis,metabolic process,transport,regulation of biological process,response to stimulus,cell communication	protein binding,metal ion binding,nucleotide binding,catalytic activity	Isoform 1 of 6-phosphofructokinase, liver type
IPI00930124.1	IGHG1	X	X	X	membrane		protein binding	Putative uncharacterized protein DKFZp686C11235
IPI00026241.1	BST2			X	membrane,cytoplasm,Golgi,endosome	cell proliferation,development,transport,regulation of biological process,response to stimulus,defense response,cell communication	protein binding,signal transducer activity,transporter activity	Bone marrow stromal antigen 2
IPI00935408.2	CFI			X	membrane		protein binding,receptor activity	Uncharacterized protein
IPI00006451.7	NSF	X	X	X	membrane,cytoplasm,Golgi,cytosol	cell organization and biogenesis,metabolic process,transport,regulation of biological process,response to stimulus,cell communication	protein binding,RNA binding,metal ion binding,nucleotide binding,catalytic activity	Vesicle-fusing ATPase
IPI00759832.1	YWHAB	X	X				protein binding	Isoform Short of 14-3-3 protein beta/alpha
IPI00015260.1	NELL2		X	X	extracellular	regulation of biological process	protein binding,metal ion binding,structural molecule activity	Protein kinase C-binding protein NELL2
IPI00029133.4	ATP5F1	X	X		membrane,mitochondrion,cytoplasm,organelle lumen	metabolic process,transport	protein binding,transporter activity,catalytic activity	ATP synthase subunit b, mitochondrial
IPI00012750.3	RPS25		X		cytoplasm,ribosome,organelle lumen,cytosol,nucleus	cell organization and biogenesis,transport,metabolic process,reproduction	RNA binding,structural molecule activity	40S ribosomal protein S25
IPI00289535.6	PMPCB	X	X			metabolic process	metal ion binding,catalytic activity	cDNA FLJ78497
IPI00033419.2	FEM1B			X	cytoplasm,organelle lumen,nucleus	cell death,development,metabolic process,regulation of biological process,response to stimulus,cell differentiation,reproduction	protein binding,catalytic activity	Protein fem-1 homolog B
IPI00010737.1	THBD			X	extracellular,cell surface,membrane	development,regulation of biological process,response to stimulus,cellular component movement,reproduction,coagulation	protein binding,signal transducer activity,metal ion binding,receptor activity	Thrombomodulin
IPI00220993.1	CNP	X	X		membrane	metabolic process	nucleotide binding,catalytic activity	Isoform CNPI of 2',3'-cyclic-nucleotide 3'-phosphodiesterase
IPI00470490.2	NCOA1			X	organelle lumen,chromosome,nucleus	cell death,cell organization and biogenesis,development,metabolic process,regulation of biological process,response to stimulus,cell communication,reproduction,cell differentiation	signal transducer activity,protein binding,DNA binding,receptor activity,catalytic activity	Isoform 1 of Nuclear receptor coactivator 1
IPI00003919.1	QPCT			X	extracellular	metabolic process	metal ion binding,catalytic activity	Isoform 1 of Glutaminyl-peptide cyclotransferase
IPI00464968.4	COX1	X			mitochondrion,membrane,cytoplasm	development,metabolic process,transport,response to stimulus	transporter activity,metal ion binding,catalytic activity	Cytochrome c oxidase subunit 1
IPI00300052.2	KRT84			X	cytoskeleton,membrane	development,regulation of biological process,cell differentiation	motor activity,structural molecule activity,catalytic activity	Keratin, type II cuticular Hb4
IPI00012669.2	SMG9		X		cytoplasm,cytosol	metabolic process	protein binding	Isoform 1 of Protein SMG9
IPI00853312.2	POM121L3			X				hCG2019008, isoform CRA_d
IPI00718977.4	GRIA4			X	membrane	transport,regulation of biological process,response to stimulus,cell communication	signal transducer activity,transporter activity,receptor activity	glutamate receptor 4 isoform 2 precursor
IPI00514517.2	IGLV5-37			X				V4-1 protein
IPI00005040.1	ACADM	X	X		mitochondrion,cytoplasm,organelle lumen	metabolic process	protein binding,catalytic activity	Isoform 1 of Medium-chain specific acyl-CoA dehydrogenase, mitochondrial
IPI00216932.4	ACSS1		X		mitochondrion,cytoplasm,organelle lumen	metabolic process,response to stimulus	protein binding,nucleotide binding,catalytic activity	Isoform 1 of Acetyl-coenzyme A synthetase 2-like, mitochondrial
IPI00293867.7	DDT	X	X	X	cytoplasm	metabolic process	catalytic activity	D-dopachrome decarboxylase
IPI00008770.1	GSTM4		X		membrane,endoplasmic reticulum,cytoplasm	metabolic process,response to stimulus	protein binding,catalytic activity	Isoform 1 of Glutathione S-transferase Mu 4
IPI00151710.8	ANO6	X			membrane	cell organization and biogenesis,transport,regulation of biological process,response to stimulus,defense response,coagulation	transporter activity	Anoctamin-6
IPI00296197.2	SIL1			X	endoplasmic reticulum,cytoplasm,organelle lumen	metabolic process,transport	protein binding	Nucleotide exchange factor SIL1
IPI00221235.4	NUP160			X		transport		Isoform 2 of Nuclear pore complex protein Nup160
IPI00009276.2	PROCR			X	membrane	response to stimulus		Endothelial protein C receptor precursor
IPI00025862.2	C4BPB			X	extracellular,membrane	metabolic process,regulation of biological process,response to stimulus,defense response,coagulation		Isoform 1 of C4b-binding protein beta chain
IPI00011770.1	NDUFA4		X		mitochondrion,membrane,cytoplasm	metabolic process,transport	catalytic activity	NADH dehydrogenase [ubiquinone] 1 alpha subcomplex subunit 4

IPI	GENE	Alzheimer's Hippocampus	Control hippocampus	CSF	Cellular localization	Biological process	Molecular function	Protein Description
IPI00306339.4	SPP1			X				osteopontin isoform b precursor
IPI00550991.4	SERPINA3		X	X			enzyme regulator activity	Isoform 2 of Alpha-1-antichymotrypsin
IPI00003391.6	ODZ1			X	membrane	regulation of biological process,response to stimulus,cell communication		teneurin-1 isoform 1
IPI00658112.2	SPEG			X				Uncharacterized protein
IPI00297124.2	IL6ST	X		X	extracellular,cell surface,membrane	cell proliferation,development,metabolic process,regulation of biological process,response to stimulus,cellular component movement,defense response,cell communication,cellular homeostasis,cell differentiation	signal transducer activity,protein binding,receptor activity	Isoform 1 of Interleukin-6 receptor subunit beta
IPI00552937.1	NHLRC3			X	extracellular		protein binding	NHL repeat-containing protein 3
IPI00019932.4	UBE2D2	X	X				nucleotide binding,catalytic activity	ubiquitin-conjugating enzyme E2 D2 isoform 2
IPI00219078.5	ATP2A2	X	X		membrane,endoplasmic reticulum,cytoplasm	development,transport,metabolic process,response to stimulus,coagulation	protein binding,transporter activity,metal ion binding,nucleotide binding,catalytic activity	Isoform 1 of Sarcoplasmic/endoplasmic reticulum calcium ATPase 2
IPI00176427.1	CADM4	X	X	X	membrane		protein binding	Cell adhesion molecule 4
IPI00003833.3	MTCH2	X	X		mitochondrion,membrane,cytoplasm	transport		Mitochondrial carrier homolog 2
IPI00217871.4	ALDH4A1	X	X	X	mitochondrion,cytoplasm,organelle lumen	metabolic process	catalytic activity	Delta-1-pyrroline-5-carboxylate dehydrogenase, mitochondrial
IPI00797851.2	MICAL3			X		metabolic process	catalytic activity	hCG21537
IPI00419273.5	CUL4A		X			cell death,cell proliferation,metabolic process,regulation of biological process,response to stimulus	protein binding	Isoform 1 of Cullin-4A
IPI00334587.1	HNRNPAB	X	X				nucleotide binding	Isoform 2 of Heterogeneous nuclear ribonucleoprotein A/B
IPI00168489.6	NRBP2	X			cytoplasm	cell death,development,metabolic process,regulation of biological process,response to stimulus,cell communication,cell differentiation	nucleotide binding,catalytic activity	Isoform 1 of Nuclear receptor-binding protein 2
IPI00106506.2	ECSIT			X	mitochondrion,cytoplasm,organelle lumen,nucleus	development,metabolic process,regulation of biological process,response to stimulus,cell communication,defense response	protein binding,signal transducer activity,catalytic activity	Isoform 1 of Evolutionarily conserved signaling intermediate in Toll pathway, mitochondrial
IPI00334666.2	PTPRN2			X	membrane,cytoplasm	metabolic process,regulation of biological process	signal transducer activity,receptor activity,catalytic activity	Isoform 1 of Receptor-type tyrosine-protein phosphatase N2
IPI00043201.2	CENPJ			X	cytoskeleton,cytoplasm,cytosol	cell organization and biogenesis,cell division,metabolic process,regulation of biological process	protein binding	Centromere protein J
IPI00015736.3	UBA5	X	X		cytoplasm,nucleus	metabolic process	protein binding,metal ion binding,nucleotide binding,catalytic activity	Isoform 1 of Ubiquitin-like modifier-activating enzyme 5
IPI00640810.1	CTDP1			X				6 kDa protein
IPI00444727.3	DLG2	X	X		cytoskeleton,membrane,cytoplasm	development,cell organization and biogenesis,metabolic process,regulation of biological process,cell communication,cell differentiation	protein binding,catalytic activity	Isoform 1 of Disks large homolog 2
IPI00387168.4	PCSK9			X	extracellular,cell surface,membrane,endoplasmic reticulum,cytoplasm,Golgi,vacuole,endosome	cell death,cell organization and biogenesis,development,metabolic process,transport,regulation of biological process,response to stimulus,cell communication,cell differentiation	protein binding,catalytic activity	Isoform 1 of Proprotein convertase subtilisin/kexin type 9
IPI00021766.5	RTN4	X	X	X	membrane,endoplasmic reticulum,cytoplasm,nucleus	cell death,cell organization and biogenesis,development,transport,regulation of biological process,response to stimulus,cellular component movement,cell communication,cell growth,cell differentiation	protein binding	Isoform 1 of Reticulon-4
IPI00029275.2	MFI2			X	extracellular,membrane	transport,cellular homeostasis	protein binding,metal ion binding	Isoform 1 of Melanotransferrin
IPI00973424.1	IGLV2-14			X			protein binding	Putative uncharacterized protein
IPI00102575.5	ATAD5			X	nucleus	response to stimulus	nucleotide binding,catalytic activity	Isoform 1 of ATPase family AAA domain-containing protein 5
IPI00008556.1	F11			X	extracellular,membrane	metabolic process,regulation of biological process,response to stimulus,coagulation	protein binding,catalytic activity	Isoform 1 of Coagulation factor XI

IPI	GENE	Alzheimer's Hippocampus	Control hippocampus	CSF	Cellular localization	Biological process	Molecular function	Protein Description
IPI00016634.1	GID8		X		nucleus		protein binding	Protein C20orf11
IPI00021727.1	C4BPA			X	extracellular,membrane,cytoplasm	metabolic process,regulation of biological process,response to stimulus,defense response	protein binding	C4b-binding protein alpha chain
IPI00008726.5	IREB2			X	mitochondrion,cytoplasm,cytosol	development,transport,metabolic process,regulation of biological process,cellular homeostasis,cell differentiation	protein binding,RNA binding,metal ion binding	Iron-responsive element-binding protein 2
IPI00298520.4	ARCN1	X	X		membrane,cytoplasm	transport	protein binding	Uncharacterized protein
IPI00942387.2	LOC100130100			X				13 kDa protein
IPI00981317.1	PPIA		X			metabolic process	catalytic activity	cDNA FLJ75025, highly similar to Homo sapiens peptidylprolyl isomerase A (cyclophilin A) (PPIA), transcript variant 2, mRNA
IPI00216048.9	PITPNA	X	X		cytoplasm	development,cell organization and biogenesis,metabolic process,transport,response to stimulus,cell differentiation	transporter activity	Phosphatidylinositol transfer protein alpha isoform
IPI00555744.6	RPL14	X	X		cytoplasm,ribosome	metabolic process	structural molecule activity	Ribosomal protein L14 variant
IPI00000006.1	HRAS		X		membrane,cytoplasm,Golgi,nucleus,cytosol	cell death,cell proliferation,cell organization and biogenesis,development,metabolic process,transport,regulation of biological process,response to stimulus,cellular component movement,cell communication,cell differentiation,coagulation	protein binding,transporter activity,nucleotide binding,catalytic activity	Isoform 1 of GTPase HRas
IPI00965841.1	IGLV1-40			X	extracellular	metabolic process,regulation of biological process,response to stimulus,defense response	protein binding	immunoglobulin lambda light chain variable region
IPI00853224.1	STARD7	X	X		mitochondrion,cytoplasm			StAR-related lipid transfer protein 7, mitochondrial
IPI00291136.4	COL6A1	X		X	extracellular,membrane	cell organization and biogenesis,development,response to stimulus,cell differentiation	protein binding	Collagen alpha-1(VI) chain
IPI00025491.1	EIF4A1	X			cytoplasm,cytosol	development,metabolic process,regulation of biological process,response to stimulus,cell communication	protein binding,RNA binding,nucleotide binding,catalytic activity	Eukaryotic initiation factor 4A-I
IPI00784156.1	AP2B1	X	X	X	membrane,cytoplasm,cytosol	development,cell organization and biogenesis,transport,regulation of biological process,response to stimulus,cell communication,defense response,cell differentiation	protein binding,transporter activity	Isoform 1 of AP-2 complex subunit beta
IPI00180386.5	GYG1	X	X	X	cytoplasm,cytosol	metabolic process	protein binding,metal ion binding,catalytic activity	Isoform GN-1L of Glycogenin-1
IPI00062264.4	SCYL1		X			metabolic process	nucleotide binding,catalytic activity	Isoform 4 of N-terminal kinase-like protein
IPI00028276.5	UNK	X						RING finger protein unkempt homolog
IPI00472058.1	NDUFB11	X	X		membrane,cytoplasm		catalytic activity	Neuronal protein
IPI00016862.1	GSR	X	X	X	mitochondrion,cytoplasm,cytosol	metabolic process,regulation of biological process,cellular homeostasis,reproduction	antioxidant activity,protein binding,nucleotide binding,catalytic activity	Isoform Mitochondrial of Glutathione reductase, mitochondrial
IPI00218606.7	RPS23	X	X		cytoplasm,ribosome,cytosol	cell organization and biogenesis,metabolic process,transport,reproduction	protein binding,structural molecule activity	40S ribosomal protein S23
IPI00793166.3	SPARCL1			X	extracellular	regulation of biological process,response to stimulus,cell communication	metal ion binding	Uncharacterized protein
IPI00410714.5	HBA2	X	X	X	cytoplasm,ribosome,cytosol	cell death,cell organization and biogenesis,transport,metabolic process,regulation of biological process,response to stimulus	antioxidant activity,protein binding,transporter activity,metal ion binding,catalytic activity	Hemoglobin subunit alpha
IPI00295399.4	CDH10			X	membrane	cell organization and biogenesis	metal ion binding	Cadherin-10
IPI00070643.6	FAF1	X			membrane,endoplasmic reticulum,cytoplasm,cytosol,nucleus	cell death,cell organization and biogenesis,metabolic process,transport,regulation of biological process,response to stimulus,cell communication	protein binding,enzyme regulator activity	Isoform Long of FAS-associated factor 1
IPI00219034.3	NDUFA8	X	X		membrane,mitochondrion,cytoplasm	transport,metabolic process	catalytic activity	NADH dehydrogenase [ubiquinone] 1 alpha subcomplex subunit 8
IPI00007675.6	DYNC1LI1	X	X		cytoskeleton,membrane,cytoplasm,chromosome	cell organization and biogenesis,cell division,transport,regulation of biological process	motor activity,nucleotide binding,catalytic activity	Cytoplasmic dynein 1 light intermediate chain 1
IPI00328745.1	RTN4RL1			X	cell surface,membrane	cell organization and biogenesis,development,response to stimulus,cell differentiation	protein binding,receptor activity	Reticulon-4 receptor-like 1
IPI00298984.5	SYN3	X	X		membrane,cytoplasm	metabolic process,transport,cell communication	nucleotide binding,catalytic activity	Synapsin-3
IPI00005573.3	NT5C	X	X		mitochondrion,cytoplasm,nucleus,cytosol	metabolic process	metal ion binding,nucleotide binding,catalytic activity	Isoform 1 of 5'(3')-deoxyribonucleotidase, cytosolic type

IPI	GENE	Alzheimer's Hippocampus	Control hippocampus	CSF	Cellular localization	Biological process	Molecular function	Protein Description
IPI00003031.3	ISOC2			X		metabolic process	catalytic activity	Isoform 2 of Isochorismatase domain-containing protein 2, mitochondrial
IPI00300341.5	TCEB1	X	X		cytoplasm,organelle lumen,cytosol,nucleus	metabolic process,regulation of biological process,reproduction	protein binding	Transcription elongation factor B polypeptide 1
IPI00149044.9	ZNF280D			X			metal ion binding	cDNA FLJ56078, highly similar to Suppressor of hairy wing homolog 4
IPI00877014.1	GDAP1	X	X		membrane,mitochondrion,cytoplasm	cell organization and biogenesis,transport,response to stimulus	protein binding	Isoform 1 of Ganglioside-induced differentiation-associated protein 1
IPI00021428.1	ACTA1			X	cytoskeleton,cytoplasm,cytosol	cell death,development,cell organization and biogenesis,metabolic process,response to stimulus,cellular component movement,cell differentiation,cell growth	protein binding,nucleotide binding,structural molecule activity,catalytic activity	Actin, alpha skeletal muscle
IPI00300074.4	FARSB	X	X		cytoplasm,cytosol	metabolic process	RNA binding,metal ion binding,nucleotide binding,catalytic activity	Phenylalanyl-tRNA synthetase beta chain
IPI00220748.2	ITGA7			X	membrane	regulation of biological process,response to stimulus,cell communication	receptor activity	Isoform Alpha-7X1A of Integrin alpha-7
IPI00019954.4	CST6			X	extracellular,cytoskeleton	development,metabolic process,regulation of biological process	enzyme regulator activity	Cystatin-M
IPI00607591.3	RAP1GDS1	X	X			metabolic process,regulation of biological process	protein binding,enzyme regulator activity	Isoform 1 of Rap1 GTPase-GDP dissociation stimulator 1
IPI00021091.1	LGI1		X	X	extracellular	cell proliferation,cell organization and biogenesis,development,regulation of biological process,response to stimulus,cell communication,cell growth,cell differentiation	protein binding	Isoform 1 of Leucine-rich glioma-inactivated protein 1
IPI00013043.1	TPPP	X	X		cytoskeleton,membrane,cytoplasm,nucleus	cell organization and biogenesis,regulation of biological process	protein binding,metal ion binding	Tubulin polymerization-promoting protein
IPI00016701.1	P2RY14			X	membrane	regulation of biological process,response to stimulus,cell communication	signal transducer activity,receptor activity	P2Y purinoceptor 14
IPI00027264.6	CALB2	X	X	X	cytoplasm		metal ion binding	Calretinin
IPI00218999.5	CFH			X				Uncharacterized protein
IPI00000265.2	FAM171A1			X	membrane			Protein FAM171A1
IPI00027685.1	CCR1			X	cell surface,membrane	development,metabolic process,transport,regulation of biological process,response to stimulus,cellular component movement,cell communication,defense response,cellular homeostasis,cell differentiation	protein binding,signal transducer activity,receptor activity,catalytic activity	C-C chemokine receptor type 1
IPI00749171.1	LOC340184			X				Conserved hypothetical protein
IPI00024523.2	DNAJB6	X	X		cytoplasm,organelle lumen,nucleus	cell death,cell organization and biogenesis,development,metabolic process,regulation of biological process,response to stimulus	protein binding,DNA binding,enzyme regulator activity	Isoform A of DnaJ homolog subfamily B member 6
IPI00887273.1	DNM1	X	X				protein binding,nucleotide binding,catalytic activity	Isoform 2 of Dynamin-1
IPI00384998.4	NFASC	X	X				protein binding	Isoform 7 of Neurofascin
IPI00456683.1	SUPT6H			X				Isoform 3 of Transcription elongation factor SPT6
IPI00034181.1	RBBP9	X	X		cytoplasm,nucleus	cell proliferation,regulation of biological process	catalytic activity	Isoform 1 of Putative hydrolase RBBP9
IPI00299263.5	ARFGAP3			X	membrane,cytoplasm,Golgi,cytosol	transport,metabolic process,regulation of biological process	transporter activity,metal ion binding,enzyme regulator activity	ADP-ribosylation factor GTPase-activating protein 3
IPI00305627.7	C16orf58		X		membrane			Isoform 1 of UPF0420 protein C16orf58
IPI00027463.1	S100A6	X		X	membrane,cytoplasm,cytosol,nucleus	cell proliferation,development,cell organization and biogenesis,transport,regulation of biological process,response to stimulus,cell communication,cell differentiation	protein binding,transporter activity,metal ion binding	Protein S100-A6
IPI00177884.7	SYNGAP1		X		membrane,cytoplasm	metabolic process,regulation of biological process,response to stimulus,cell communication	protein binding,enzyme regulator activity	Isoform 1 of Ras GTPase-activating protein SynGAP
IPI00289159.3	GLS	X	X		mitochondrion,cytoplasm,organelle lumen,cytosol	cell organization and biogenesis,transport,metabolic process,regulation of biological process,response to stimulus,cell communication	protein binding,catalytic activity	Isoform 1 of Glutaminase kidney isoform, mitochondrial
IPI00008438.1	RPS10			X	cytoplasm,ribosome,organelle lumen,nucleus,cytosol	cell organization and biogenesis,transport,metabolic process,reproduction	protein binding	40S ribosomal protein S10
IPI00005605.4	NDRG3	X	X	X	cytoplasm	cell organization and biogenesis,regulation of biological process,cell growth,cell differentiation,reproduction		Isoform 1 of Protein NDRG3

IPI	GENE	Alzheimer's Hippocampus	Control hippocampus	CSF	Cellular localization	Biological process	Molecular function	Protein Description
IPI00294578.1	TGM2		X		mitochondrion,membrane,cytoplasm	cell death,cell proliferation,development,cell organization and biogenesis,transport,metabolic process,regulation of biological process,response to stimulus,cell communication,defense response,cellular homeostasis	protein binding,metal ion binding,nucleotide binding,catalytic activity	Isoform 1 of Protein-glutamine gamma-glutamyltransferase 2
IPI00419997.1	SLC25A23		X		mitochondrion,membrane,cytoplasm	transport	metal ion binding	Isoform 1 of Calcium-binding mitochondrial carrier protein SCaMC-3
IPI00001091.4	AFG3L2		X		mitochondrion,membrane,cytoplasm	cell death,cell organization and biogenesis,development,metabolic process,regulation of biological process,response to stimulus,cell communication,cellular homeostasis,cell differentiation	protein binding,metal ion binding,nucleotide binding,catalytic activity	AFG3-like protein 2
IPI00003933.2	HAGH	X	X	X	mitochondrion,cytoplasm,organelle lumen	metabolic process	metal ion binding,catalytic activity	Isoform 1 of Hydroxyacylglutathione hydrolase, mitochondrial
IPI00395747.4	SCAMP5	X	X		membrane,cytoplasm,Golgi,endosome	cell organization and biogenesis,transport,regulation of biological process,response to stimulus	protein binding	Isoform 1 of Secretory carrier-associated membrane protein 5
IPI00029751.1	CNTN1	X	X	X	membrane	development,cell organization and biogenesis,regulation of biological process,response to stimulus,cell communication,cell differentiation	protein binding	Isoform 1 of Contactin-1
IPI00021129.5	AP3B1	X	X		membrane,cytoplasm,Golgi	cell organization and biogenesis,development,transport,regulation of biological process,response to stimulus,cell differentiation,coagulation	protein binding,transporter activity	Isoform 1 of AP-3 complex subunit beta-1
IPI00395887.4	TMX1	X	X		membrane,endoplasmic reticulum,cytoplasm	cell death,cell proliferation,metabolic process,transport,regulation of biological process,response to stimulus,cell communication,cellular homeostasis	catalytic activity	Thioredoxin-related transmembrane protein 1
IPI00031086.1	IGFBP1			X	extracellular	cell organization and biogenesis,metabolic process,regulation of biological process,response to stimulus,cell communication,cell growth	protein binding	Insulin-like growth factor-binding protein 1
IPI00045109.3	HIST1H2AA	X	X		chromosome,nucleus	cell organization and biogenesis,metabolic process	DNA binding	Histone H2A type 1-A
IPI00022432.1	TTR	X	X	X	extracellular,cytoplasm	transport	protein binding	Transthyretin
IPI00008240.2	MARS	X	X		mitochondrion,cytoplasm,cytosol	metabolic process	RNA binding,nucleotide binding,catalytic activity	Methionyl-tRNA synthetase, cytoplasmic
IPI00015954.1	SAR1A	X	X	X	endoplasmic reticulum,cytoplasm,Golgi	metabolic process,transport,regulation of biological process,response to stimulus,cell communication	signal transducer activity,nucleotide binding,catalytic activity	GTP-binding protein SAR1a
IPI00007052.6	FIS1	X	X		mitochondrion,membrane,cytoplasm	cell death,cell organization and biogenesis,metabolic process,regulation of biological process,response to stimulus,cell communication,cellular homeostasis	protein binding	Mitochondrial fission 1 protein
IPI00002270.3	C6orf211	X	X					UPF0364 protein C6orf211
IPI00295400.1	WARS	X	X	X	cytoplasm,cytosol	cell proliferation,development,metabolic process,regulation of biological process	protein binding,nucleotide binding,catalytic activity	Isoform 1 of Tryptophanyl-tRNA synthetase, cytoplasmic
IPI00785015.3	UBN2			X	organelle lumen,nucleus			Isoform 1 of Ubinuclein-2
IPI00011253.3	RPS3	X	X		membrane,cytoplasm,ribosome,cytosol,nucleus	cell death,cell organization and biogenesis,metabolic process,transport,regulation of biological process,response to stimulus,reproduction	protein binding,DNA binding,RNA binding,structural molecule activity,catalytic activity	40S ribosomal protein S3
IPI00015423.1	MAP1LC3A	X	X		cytoplasm	cell organization and biogenesis,metabolic process,response to stimulus,cell communication		Isoform 2 of Microtubule-associated proteins 1A/1B light chain 3A
IPI00329688.2	YIPF3			X	membrane,cytoplasm,Golgi	cell differentiation		Protein YIPF3
IPI00007611.1	ATP5O	X	X		mitochondrion,membrane,cytoplasm	metabolic process,transport	transporter activity,catalytic activity	ATP synthase subunit O, mitochondrial
IPI00216348.1	DYNC1I2	X	X	X	cytoskeleton,cytoplasm	cellular component movement	protein binding	Isoform 2C of Cytoplasmic dynein 1 intermediate chain 2
IPI00030037.1	ASIP			X	extracellular	metabolic process,regulation of biological process,response to stimulus,cell communication	protein binding	Agouti-signaling protein
IPI00289861.4	ZCCHC11		X	X	cytoplasm,organelle lumen,nucleus	metabolic process,regulation of biological process,response to stimulus,cell communication,cell differentiation	protein binding,metal ion binding,catalytic activity	Isoform 1 of Terminal uridylyltransferase 4
IPI00008223.3	RAD23B	X	X	X	cytoplasm,proteasome,organelle lumen,nucleus	cell organization and biogenesis,metabolic process,regulation of biological process,response to stimulus,reproduction	protein binding,DNA binding	UV excision repair protein RAD23 homolog B
IPI00021327.3	GRB2	X	X	X	membrane,cytoplasm,Golgi,cytosol,nucleus,endosome	development,cell organization and biogenesis,transport,metabolic process,regulation of biological process,response to stimulus,cellular component movement,cell communication,cell differentiation,coagulation	protein binding,receptor activity	Isoform 1 of Growth factor receptor-bound protein 2

IPI	GENE	Alzheimer's Hippocampus	Control hippocampus	CSF	Cellular localization	Biological process	Molecular function	Protein Description
IPI00021048.1	MYOF			X	membrane,cytoplasm,nucleus	cell organization and biogenesis,response to stimulus	protein binding	Isoform 1 of Myoferlin
IPI00217435.4	SCUBE1			X	extracellular,cell surface,membrane	cell organization and biogenesis,development,response to stimulus,defense response,cell differentiation,coagulation	protein binding,metal ion binding	Signal peptide, CUB and EGF-like domain-containing protein 1
IPI00305092.7	WIBG	X			cytoplasm,organelle lumen,nucleus	metabolic process,regulation of biological process	protein binding,RNA binding	Isoform 1 of Partner of Y14 and mago
IPI00480049.2	OCRL		X			regulation of biological process,response to stimulus,cell communication		Isoform B of Inositol polyphosphate 5-phosphatase OCRL-1
IPI00176424.1	NLGN2			X	cell surface,membrane	cell organization and biogenesis,development,transport,metabolic process,regulation of biological process,response to stimulus,cell communication,cellular homeostasis	protein binding,receptor activity,catalytic activity	Neuroligin-2
IPI00027285.1	SNRPB	X			spliceosomal complex,cytoplasm,organelle lumen,cytosol,nucleus	cell organization and biogenesis,metabolic process	protein binding,RNA binding	Isoform SM-B' of Small nuclear ribonucleoprotein-associated proteins B and B'
IPI00419630.1	CNDP1			X		metabolic process	catalytic activity	beta-Ala-His dipeptidase precursor
IPI00788786.4	VWF			X	extracellular		protein binding	cDNA FLJ51654, highly similar to von Willebrand factor
IPI00217759.2	FUT11			X	membrane,cytoplasm,Golgi	metabolic process	catalytic activity	Isoform 1 of Alpha-(1,3)-fucosyltransferase 11
IPI00217264.6	MAP7D3			X			protein binding	Isoform 3 of MAP7 domain-containing protein 3
IPI00385543.1	KIAA0907			X		metabolic process	catalytic activity	Isoform 3 of UPF0469 protein KIAA0907
IPI00107722.6	MCEE	X	X		mitochondrion,cytoplasm,organelle lumen	metabolic process	catalytic activity	Methylmalonyl-CoA epimerase, mitochondrial
IPI00398229.1	USP17L7			X	nucleus	metabolic process	catalytic activity	Inactive ubiquitin carboxyl-terminal hydrolase 17-like protein 7
IPI00177965.5	NT5DC1	X	X				metal ion binding,catalytic activity	5'-nucleotidase domain-containing protein 1
IPI00002535.2	FKBP2	X	X	X	membrane,endoplasmic reticulum,cytoplasm	metabolic process	protein binding,catalytic activity	Peptidyl-prolyl cis-trans isomerase FKBP2
IPI00009362.2	SCG2	X	X	X	extracellular,cytoplasm	cell death,cell proliferation,development,transport,regulation of biological process,response to stimulus,cellular component movement,defense response,cell communication	protein binding	Secretogranin-2
IPI00002732.12	EXTL2			X	membrane,endoplasmic reticulum,cytoplasm		catalytic activity	EXTL2 protein (Fragment)
IPI00013877.2	HNRNPH3	X	X		organelle lumen,nucleus	metabolic process	RNA binding,nucleotide binding	Isoform 1 of Heterogeneous nuclear ribonucleoprotein H3
IPI00021370.1	UBE2K	X	X		cytoplasm	metabolic process	protein binding,nucleotide binding,catalytic activity	Isoform 1 of Ubiquitin-conjugating enzyme E2 K
IPI00008964.3	RAB1B	X	X		membrane,mitochondrion,cytoplasm,Golgi,nucleus	transport,regulation of biological process,response to stimulus,cell communication	nucleotide binding	Ras-related protein Rab-1B
IPI00020977.4	CTGF			X	extracellular,membrane,cytoplasm,Golgi,cytosol	cell death,cell proliferation,cell organization and biogenesis,development,metabolic process,transport,regulation of biological process,response to stimulus,cellular component movement,cell communication,cell differentiation,cell growth	protein binding	Isoform 1 of Connective tissue growth factor
IPI00007067.5	GLIPR2	X	X		extracellular,membrane,cytoplasm,Golgi			Golgi-associated plant pathogenesis-related protein 1
IPI00032425.1	RAMP3			X	cell surface,membrane,cytoplasm,vacuole	cell organization and biogenesis,transport,metabolic process,regulation of biological process,response to stimulus,cell communication	protein binding,signal transducer activity,transporter activity,receptor activity	Receptor activity-modifying protein 3
IPI00004457.3	AOC3			X	cell surface,membrane	metabolic process,response to stimulus,defense response	protein binding,transporter activity,metal ion binding,catalytic activity	Membrane primary amine oxidase
IPI00007040.2	ZNF222			X	nucleus	metabolic process,regulation of biological process	DNA binding,metal ion binding	Zinc finger protein 222
IPI00016385.4	MAP6D1	X			cytoskeleton,cytoplasm,Golgi	cell organization and biogenesis,metabolic process,regulation of biological process	protein binding	MAP6 domain-containing protein 1
IPI00746165.2	WDR1	X	X		extracellular,cytoskeleton,cytoplasm,cytosol	transport,response to stimulus,coagulation	protein binding	Isoform 1 of WD repeat-containing protein 1
IPI00182293.6	GUK1	X	X		cytoplasm,cytosol	metabolic process	protein binding,RNA binding,nucleotide binding,catalytic activity	Guanylate kinase

IPI	GENE	Alzheimer's Hippocampus	Control hippocampus	CSF	Cellular localization	Biological process	Molecular function	Protein Description
IPI00298828.3	APOH			X	extracellular,cell surface	cell death,cell proliferation,development,metabolic process,transport,regulation of biological process,response to stimulus,cellular component movement,coagulation	protein binding,enzyme regulator activity	Beta-2-glycoprotein 1
IPI00554737.3	PPP2R1A	X	X		cytoskeleton,membrane,mitochondrion,cytoplasm,chromosome,nucleus ,cytosol	cell death,cell organization and biogenesis,metabolic process,regulation of biological process,response to stimulus,cell communication,cell growth,cell differentiation	protein binding,catalytic activity,enzyme regulator activity	Serine/threonine-protein phosphatase 2A 65 kDa regulatory subunit A alpha isoform
IPI00442307.1	KCNAB2	X			membrane,cytoplasm	transport	transporter activity	Isoform 3 of Voltage-gated potassium channel subunit beta-2
IPI00220791.6	AMPH			X			protein binding	Uncharacterized protein
IPI00043756.6	SLC39A12	X	X	X	membrane	transport	transporter activity	Isoform 3 of Zinc transporter ZIP12
IPI00785007.2	C4orf48			X				novel protein
IPI00784002.3	SACS		X					Isoform 2 of Sacsin
IPI00477361.3	SDHAP1			X		metabolic process	catalytic activity	10 kDa protein
IPI00827872.2	EPB42			X	cytoskeleton,membrane,cytoplasm	cell organization and biogenesis,development,metabolic process,regulation of biological process,cell differentiation	protein binding,nucleotide binding,structural molecule activity,catalytic activity	Isoform Short of Erythrocyte membrane protein band 4.2
IPI00026991.4	GALNT6			X	membrane,cytoplasm,Golgi	metabolic process	catalytic activity	Polypeptide N-acetylgalactosaminyltransferase 6
IPI00023302.2	SYN2	X	X		cytoplasm	metabolic process,transport,cell communication	nucleotide binding,catalytic activity	Isoform IIa of Synapsin-2
IPI00012463.4	TATDN1		X		nucleus	metabolic process	metal ion binding,catalytic activity	Isoform 1 of Putative deoxyribonuclease TATDN1
IPI00303882.3	PLIN3	X	X	X	membrane,cytoplasm,Golgi,endosome	transport	protein binding	Isoform B of Perilipin-3
IPI00304577.4	AP2A1			X	membrane,cytoplasm,Golgi,cytosol	development,cell organization and biogenesis,transport,regulation of biological process,response to stimulus,defense response,cell communication,cell differentiation	protein binding,transporter activity	Isoform A of AP-2 complex subunit alpha-1
IPI00218795.1	SELL			X				L-selectin precursor
IPI00910602.1	NEFH	X	X		cytoskeleton,mitochondrion,cytoplasm	cell death,development,cell organization and biogenesis		Isoform 1 of Neurofilament heavy polypeptide
IPI00855725.1	TGOLN2			X	membrane			Isoform 4 of Trans-Golgi network integral membrane protein 2
IPI00162199.18	TECPR1		X		membrane,cytoplasm,vacuole	cell organization and biogenesis,metabolic process,response to stimulus,cell communication	protein binding	Isoform 1 of Tectonin beta-propeller repeat-containing protein 1
IPI00296608.6	C7			X	extracellular,membrane	cell death,metabolic process,regulation of biological process,response to stimulus,defense response	protein binding	Complement component C7
IPI00301434.4	BOLA2B	X	X				transcription regulator activity	BolA-like protein 2
IPI00000030.1	PPP2R5D		X		cytoplasm,nucleus	development,regulation of biological process,response to stimulus,cell communication	protein binding,enzyme regulator activity	Isoform Delta-1 of Serine/threonine-protein phosphatase 2A 56 kDa regulatory subunit delta isoform
IPI00004656.3	B2M			X	extracellular,membrane,endoplasmic reticulum,cytoplasm,Golgi,organelle lumen,endosome	regulation of biological process,response to stimulus,cell communication,defense response	protein binding	Beta-2-microglobulin
IPI00218414.5	CA2	X	X	X	extracellular,membrane,cytoplasm,cytosol	development,metabolic process,transport,regulation of biological process,response to stimulus,cellular homeostasis,cell differentiation	metal ion binding,catalytic activity	Carbonic anhydrase 2
IPI00255052.5	NDUFB9	X	X		mitochondrion,membrane,cytoplasm	metabolic process,transport	catalytic activity	NADH dehydrogenase [ubiquinone] 1 beta subcomplex subunit 9
IPI00217989.4	SCYL3			X	cytoplasm,Golgi	metabolic process,cellular component movement	protein binding,nucleotide binding,catalytic activity	Isoform 1 of Protein-associating with the carboxyl-terminal domain of ezrin
IPI00171874.3	RASGRP3			X	membrane,cytoplasm	metabolic process,regulation of biological process,response to stimulus,cell communication	signal transducer activity,protein binding,metal ion binding,enzyme regulator activity	Ras guanyl-releasing protein 3
IPI00009253.2	NAPA	X	X		membrane,cytoplasm,cytosol	development,cell organization and biogenesis,transport,regulation of biological process,cell communication,cell differentiation	protein binding	Alpha-soluble NSF attachment protein
IPI00021833.1	PDGFA			X	extracellular,cell surface,membrane,endoplasmic reticulum,cytoplasm,Golgi,organelle lumen	cell proliferation,development,cell organization and biogenesis,cell division,metabolic process,transport,regulation of biological process,response to stimulus,cellular component movement,cell communication,coagulation	protein binding	Isoform Long of Platelet-derived growth factor subunit A
IPI00008943.3	DDX19B	X	X		membrane,cytoplasm,nucleus	transport	RNA binding,nucleotide binding,catalytic activity	Isoform 1 of ATP-dependent RNA helicase DDX19B

IPI	GENE	Alzheimer's Hippocampus	Control hippocampus	CSF	Cellular localization	Biological process	Molecular function	Protein Description
IPI00160021.2	NFU1	X	X			cell organization and biogenesis	metal ion binding	Isoform 3 of NFU1 iron-sulfur cluster scaffold homolog, mitochondrial
IPI00022388.2	DPYSL4	X	X		cytoplasm,cytosol	cell organization and biogenesis,development,metabolic process,response to stimulus,cell differentiation	catalytic activity	Dihydropyrimidinase-related protein 4
IPI00383871.1	SLC25A27	X	X		mitochondrion,membrane,cytoplasm	transport,metabolic process		Mitochondrial uncoupling protein 4
IPI00002191.5	ERVMER34-1			X	membrane			Uncharacterized protein LP9056
IPI00399053.4	NDUFAF3	X			membrane,mitochondrion,cytoplasm,nucleus	cell organization and biogenesis	protein binding	Isoform a of NADH dehydrogenase [ubiquinone] 1 alpha subcomplex assembly factor 3
IPI00031696.5	FASTKD3			X	mitochondrion,cytoplasm	metabolic process	catalytic activity	FAST kinase domain-containing protein 3
IPI00023530.7	CDK5		X		cytoskeleton,membrane,cytoplasm,cytosol,nucleus	cell death,development,cell organization and biogenesis,metabolic process,regulation of biological process,response to stimulus,cell proliferation,cell division,transport,cellular component movement,cell communication,cellular homeostasis,cell differentiation,cell growth,coagulation	protein binding,nucleotide binding,catalytic activity	Cyclin-dependent kinase 5
IPI00010204.1	SRSF3	X	X		organelle lumen,nucleus	metabolic process,transport	protein binding,RNA binding,nucleotide binding	Serine/arginine-rich splicing factor 3
IPI00025092.1	MYBPC1			X	cytoskeleton,cytoplasm,cytosol	cellular component movement	protein binding,structural molecule activity	Isoform 1 of Myosin-binding protein C, slow-type
IPI00014850.4	PEA15	X	X	X	cytoskeleton,cytoplasm	cell death,transport,regulation of biological process,response to stimulus	protein binding	Astrocytic phosphoprotein PEA-15
IPI00014151.3	PSMD6	X	X		cytoplasm,proteasome,organelle lumen,cytosol,nucleus	cell death,metabolic process,regulation of biological process,response to stimulus,cell communication	protein binding,catalytic activity	26S proteasome non-ATPase regulatory subunit 6
IPI00157734.2	EXOC3		X		membrane,cytoplasm	transport	protein binding	Isoform 1 of Exocyst complex component 3
IPI00010348.1	DNASE2			X	cytoplasm,vacuole	cell death,development,metabolic process,cell differentiation	DNA binding,catalytic activity	Deoxyribonuclease-2-alpha
IPI00219358.7	MPI	X	X		cytoplasm,cytosol	metabolic process	metal ion binding,catalytic activity	Isoform 1 of Mannose-6-phosphate isomerase
IPI00027726.1	KLF3			X	nucleus	development,metabolic process,regulation of biological process	DNA binding,metal ion binding	Isoform 1 of Krueppel-like factor 3
IPI00026271.5	RPS14	X	X		cytoplasm,ribosome,organelle lumen,cytosol,nucleus	development,cell organization and biogenesis,metabolic process,transport,regulation of biological process,cell differentiation,reproduction	RNA binding,translation regulator activity,structural molecule activity	40S ribosomal protein S14
IPI00008485.1	ACO1	X	X		membrane,mitochondrion,endoplasmic reticulum,cytoplasm,Golgi,cytosol	development,metabolic process,regulation of biological process,response to stimulus,cellular homeostasis	protein binding,RNA binding,metal ion binding,catalytic activity	Cytoplasmic aconitate hydratase
IPI00973585.1	IGLC1			X			protein binding	IGL@ protein
IPI00022228.2	HDLBP	X			extracellular,membrane,cytoplasm,nucleus	metabolic process,transport	RNA binding	Vigilin
IPI00298650.1	ADAMTS8			X		metabolic process	metal ion binding,catalytic activity	ADAMTS-8 precursor
IPI00332511.5	PPP2R2A	X	X		cytoplasm,cytosol	metabolic process,regulation of biological process,response to stimulus,cell communication	protein binding,catalytic activity,enzyme regulator activity	Serine/threonine-protein phosphatase 2A 55 kDa regulatory subunit B alpha isoform
IPI00411680.11	PCMT1			X	endoplasmic reticulum,cytoplasm	metabolic process	catalytic activity	Isoform 1 of Protein-L-isoaspartate(D-aspartate) O-methyltransferase
IPI00182180.2	OTUD6B		X		membrane			OTU domain-containing protein 6B
IPI00215917.3	ARF3	X	X		cytoplasm,Golgi,nucleus	metabolic process,transport,regulation of biological process,response to stimulus,cell communication	signal transducer activity,nucleotide binding,catalytic activity	ADP-ribosylation factor 3
IPI00295502.6	WIZ			X	nucleus		metal ion binding	Isoform 1 of Protein Wiz
IPI00218834.9	FCGR3A			X	extracellular,cell surface,membrane	regulation of biological process,response to stimulus	protein binding,receptor activity	Low affinity immunoglobulin gamma Fc region receptor III-A
IPI00030578.2	VAT1L	X	X			metabolic process	metal ion binding,nucleotide binding,catalytic activity	Synaptic vesicle membrane protein VAT-1 homolog-like
IPI00219381.5	NDUFA2	X	X		mitochondrion,membrane,cytoplasm	metabolic process,transport	catalytic activity	NADH dehydrogenase [ubiquinone] 1 alpha subcomplex subunit 2
IPI00007928.4	PRPF8	X	X	X	spliceosomal complex,organelle lumen,nucleus	metabolic process	protein binding,RNA binding	Pre-mRNA-processing-splicing factor 8
IPI00022448.4	CXCL10			X	extracellular,cell surface,membrane	cell proliferation,development,transport,regulation of biological process,response to stimulus,cellular component movement,defense response,cell communication	protein binding,enzyme regulator activity	C-X-C motif chemokine 10
IPI00004416.1	CHMP2A		X		membrane,cytoplasm,cytosol,endosome	cell organization and biogenesis,transport	protein binding	Charged multivesicular body protein 2a

IPI	GENE	Alzheimer's Hippocampus	Control hippocampus	CSF	Cellular localization	Biological process	Molecular function	Protein Description
IPI00000760.1	DDAH2	X	X	X	cytoplasm	cell death,metabolic process,regulation of biological process,response to stimulus,cell communication	protein binding,catalytic activity	N(G),N(G)-dimethylarginine dimethylaminohydrolase 2
IPI00003377.1	SRSF7	X	X		organelle lumen,nucleus	metabolic process,transport,regulation of biological process	protein binding,RNA binding,metal ion binding,nucleotide binding	Isoform 1 of Serine/arginine-rich splicing factor 7
IPI00030058.1	GNG4	X	X		membrane	cell organization and biogenesis,metabolic process,regulation of biological process,response to stimulus,cell communication,cell growth	signal transducer activity	Guanine nucleotide-binding protein G(I)/G(S)/G(O) subunit gamma-4
IPI00005142.1	FGFR1			X	extracellular,membrane,cytoplasm,cytosol,nucleus	cell proliferation,development,cell organization and biogenesis,metabolic process,regulation of biological process,response to stimulus,cellular component movement,cell communication,cell differentiation	protein binding,signal transducer activity,nucleotide binding,receptor activity,catalytic activity	Isoform 1 of Basic fibroblast growth factor receptor 1
IPI00003813.5	CADM1	X	X	X	membrane,cytoplasm	cell death,cell proliferation,development,cell organization and biogenesis,transport,regulation of biological process,response to stimulus,defense response,cell growth,cell differentiation,reproduction	protein binding	Isoform 1 of Cell adhesion molecule 1
IPI00064377.2	RELT			X	membrane,cytoplasm		protein binding,receptor activity	Tumor necrosis factor receptor superfamily member 19L
IPI00005531.1	APOBEC3B			X		metabolic process,regulation of biological process	RNA binding,metal ion binding,catalytic activity	Isoform 1 of Probable DNA dC->dU-editing enzyme APOBEC-3B
IPI00294650.5	FRZB			X	extracellular,membrane,cytoplasm	cell death,cell proliferation,cell organization and biogenesis,development,regulation of biological process,response to stimulus,cell communication,cell growth,reproduction,cell differentiation	signal transducer activity,protein binding,receptor activity	Secreted frizzled-related protein 3
IPI00016949.1	SLC4A4			X	membrane	transport	transporter activity	Isoform 4 of Electrogenic sodium bicarbonate cotransporter 1
IPI00739099.2	COL5A2			X	extracellular	cell organization and biogenesis,development,response to stimulus,cell differentiation	protein binding,structural molecule activity	Collagen alpha-2(V) chain
IPI00166553.3	FAM19A2			X	cytoplasm			Isoform 1 of Protein FAM19A2
IPI00304596.3	NONO	X	X	X	organelle lumen,nucleus	metabolic process,regulation of biological process,response to stimulus	protein binding,DNA binding,RNA binding,nucleotide binding	Non-POU domain-containing octamer-binding protein
IPI00375441.2	FUBP1		X		organelle lumen,nucleus	metabolic process,regulation of biological process	DNA binding,RNA binding	Isoform 1 of Far upstream element-binding protein 1
IPI01009172.1	SPTAN1			X			protein binding,metal ion binding	Spectrin alpha chain, brain
IPI00746030.2	PLCD1	X	X		cytoplasm	metabolic process,regulation of biological process,response to stimulus,cell communication	protein binding,signal transducer activity,metal ion binding,catalytic activity	1-phosphatidylinositol-4,5-bisphosphate phosphodiesterase delta-1
IPI00165936.1	CLIC6		X	X				Isoform A of Chloride intracellular channel protein 6
IPI00187143.1	RAB4B	X	X	X	cytoplasm,nucleus	regulation of biological process,response to stimulus,cell communication	nucleotide binding,catalytic activity	Isoform 2 of Ras-related protein Rab-4B
IPI00167515.1	PTGR2	X	X		cytoplasm	metabolic process	metal ion binding,nucleotide binding,catalytic activity	Isoform 1 of Prostaglandin reductase 2
IPI00550069.3	RNH1	X	X		extracellular,cytoplasm	development,metabolic process,regulation of biological process	protein binding,enzyme regulator activity	Ribonuclease inhibitor
IPI00009793.4	C1RL			X	extracellular	metabolic process,regulation of biological process,response to stimulus,defense response	catalytic activity	Complement C1r subcomponent-like protein
IPI00002149.1	SAR1B	X	X		membrane,endoplasmic reticulum,cytoplasm,Golgi,cytosol	cell organization and biogenesis,transport,metabolic process,regulation of biological process,response to stimulus,cell communication	signal transducer activity,metal ion binding,nucleotide binding,catalytic activity	GTP-binding protein SAR1b
IPI00012011.6	CFL1	X	X	X	cytoskeleton,membrane,cytoplasm,organelle lumen,nucleus	cell death,cell organization and biogenesis,development,transport,regulation of biological process,response to stimulus,cell communication,cell differentiation,coagulation	protein binding	Cofilin-1
IPI00006482.1	ATP1A1	X	X	X	membrane,endoplasmic reticulum,cytoplasm,Golgi	metabolic process,transport,regulation of biological process,response to stimulus	protein binding,transporter activity,metal ion binding,nucleotide binding,catalytic activity	Isoform Long of Sodium/potassium-transporting ATPase subunit alpha-1
IPI00219153.4	RPL22	X	X		cytoplasm,ribosome,cytosol	development,cell organization and biogenesis,metabolic process,transport,cell differentiation,reproduction	RNA binding,structural molecule activity	60S ribosomal protein L22
IPI00419253.5	NCKAP5			X				Isoform 1 of Nck-associated protein 5
IPI00029175.5	KIAA0196	X	X	X	cytoplasm	cell death		WASH complex subunit strumpellin
IPI00019733.1	RAE1		X		cytoskeleton,membrane,cytoplasm,nucleus	metabolic process,transport,regulation of biological process,response to stimulus,cell communication	protein binding,RNA binding	mRNA export factor
IPI00386630.1	TCN2			X		transport		transcobalamin-2 isoform 2 precursor

IPI	GENE	Alzheimer's Hippocampus	Control hippocampus	CSF	Cellular localization	Biological process	Molecular function	Protein Description
IPI00219563.1	PLCB1	X	X		membrane,cytoplasm,organelle lumen,chromosome,cytosol,nucleus	development,metabolic process,transport,regulation of biological process,response to stimulus,cellular component movement,cell communication,reproduction,cell differentiation	signal transducer activity,protein binding,metal ion binding,catalytic activity,enzyme regulator activity	Isoform A of 1-phosphatidylinositol-4,5-bisphosphate phosphodiesterase beta-1
IPI00003363.2	PPP1R1B	X	X	X	cytoplasm,cytosol,nucleus	metabolic process,regulation of biological process,response to stimulus,cell communication,reproduction	protein binding,enzyme regulator activity	Isoform 1 of Protein phosphatase 1 regulatory subunit 1B
IPI00009904.1	PDIA4	X	X	X	endoplasmic reticulum,cytoplasm,organelle lumen	metabolic process,transport,regulation of biological process,cellular homeostasis	metal ion binding,catalytic activity	Protein disulfide-isomerase A4
IPI00171407.4	STXBP5	X	X		membrane,cytoplasm	transport,regulation of biological process	protein binding	Isoform 1 of Syntaxin-binding protein 5
IPI00166130.1	DESI1	X			cytoplasm,nucleus	metabolic process	catalytic activity	PPPDE peptidase domain-containing protein 2
IPI00376798.3	RPL11	X	X		cytoplasm,ribosome,organelle lumen,cytosol,nucleus	cell organization and biogenesis,transport,metabolic process,reproduction	protein binding,RNA binding,structural molecule activity	Isoform 1 of 60S ribosomal protein L11
IPI00018980.1	SCN1B	X		X	extracellular,membrane	development,cell organization and biogenesis,transport,regulation of biological process,response to stimulus,cell communication,cellular homeostasis,cell differentiation	transporter activity	Sodium channel subunit beta-1
IPI00032147.1	ATP6V1G2		X		membrane,cytoplasm,vacuole,cytosol	transport,regulation of biological process,response to stimulus,cell communication,cellular homeostasis	transporter activity,catalytic activity	V-type proton ATPase subunit G 2
IPI00019385.4	SSR4	X	X		membrane,endoplasmic reticulum,cytoplasm	transport,metabolic process	metal ion binding	Translocon-associated protein subunit delta
IPI00179298.5	HUWE1	X	X			metabolic process	protein binding,catalytic activity	Uncharacterized protein
IPI00021033.2	COL3A1			X	extracellular	development,cell organization and biogenesis,metabolic process,regulation of biological process,response to stimulus,cell communication,cell differentiation,coagulation	protein binding,structural molecule activity	Isoform 1 of Collagen alpha-1(III) chain
IPI00399252.2	PHF17			X	mitochondrion,cytoplasm,organelle lumen,nucleus	cell death,cell organization and biogenesis,metabolic process,regulation of biological process,response to stimulus,cell growth	protein binding,metal ion binding	Isoform 1 of Protein Jade-1
IPI00016513.5	RAB10	X	X		membrane,cytoplasm,Golgi,nucleus	transport,regulation of biological process,response to stimulus,cell communication	nucleotide binding	Ras-related protein Rab-10
IPI00001710.3	VPS51		X		membrane,cytoplasm,Golgi	transport		Isoform 1 of Protein fat-free homolog
IPI00064652.6	VEGFA			X	membrane		protein binding	vascular endothelial growth factor A isoform e
IPI00029699.1	RNASE4			X	extracellular	metabolic process	catalytic activity	Ribonuclease 4
IPI00182138.4	GRN			X	extracellular,cytoplasm	cell proliferation,development,metabolic process,regulation of biological process,reproduction	catalytic activity	Isoform 2 of Granulins
IPI00442121.4	ALAD			X		metabolic process	metal ion binding,catalytic activity	Isoform 2 of Delta-aminolevulinic acid dehydratase
IPI00026268.3	GNB1	X	X		membrane	cell proliferation,development,metabolic process,regulation of biological process,response to stimulus,cell communication,cellular homeostasis,coagulation	signal transducer activity,protein binding,catalytic activity	Guanine nucleotide-binding protein G(I)/G(S)/G(T) subunit beta-1
IPI00456758.4	RPL27A	X	X		cytoplasm,ribosome,cytosol	cell organization and biogenesis,metabolic process,transport,reproduction	protein binding,RNA binding,structural molecule activity	60S ribosomal protein L27a
IPI00030009.4	PAPSS2			X	cytoplasm,cytosol	development,metabolic process,response to stimulus	nucleotide binding,catalytic activity	Isoform A of Bifunctional 3'-phosphoadenosine 5'-phosphosulfate synthase 2
IPI00022890.1	IGLV7-43			X	extracellular,membrane	metabolic process,regulation of biological process,response to stimulus,defense response	protein binding	Ig lambda chain V region 4A
IPI00003467.3	GABRB3		X		membrane	transport,regulation of biological process,response to stimulus,cell communication	signal transducer activity,transporter activity,receptor activity	Isoform 1 of Gamma-aminobutyric acid receptor subunit beta-3
IPI00300241.2	LRRC4B	X	X	X	membrane	development,cell organization and biogenesis,regulation of biological process,cell communication	protein binding	Leucine-rich repeat-containing protein 4B
IPI00167254.3	PLD5			X				Isoform 4 of Inactive phospholipase D5
IPI00386284.5	OR2AK2			X	membrane	regulation of biological process,response to stimulus,cell communication	signal transducer activity,receptor activity	Olfactory receptor 2AK2
IPI00328748.5	MANF	X	X		extracellular,endoplasmic reticulum,cytoplasm			cDNA FLJ77177, highly similar to Homo sapiens arginine-rich, mutated in early stage tumors (ARMET), mRNA
IPI00219018.7	GAPDH	X	X	X	cytoskeleton,membrane,cytoplasm,cytosol,nucleus	cell death,cell organization and biogenesis,metabolic process,regulation of biological process	protein binding,nucleotide binding,catalytic activity	Glyceraldehyde-3-phosphate dehydrogenase
IPI00006533.2	WIPI2		X					Isoform 3 of WD repeat domain phosphoinositide-interacting protein 2

IPI	GENE	Alzheimer's Hippocampus	Control hippocampus	CSF	Cellular localization	Biological process	Molecular function	Protein Description
IPI00644712.4	XRCC6	X	X		membrane,cytoplasm,organelle lumen,chromosome,nucleus	cell organization and biogenesis,development,metabolic process,regulation of biological process,response to stimulus,cell differentiation,reproduction	protein binding,DNA binding,nucleotide binding,catalytic activity	X-ray repair cross-complementing protein 6
IPI00010402.2	SH3BGRL3	X	X	X		regulation of biological process,cellular homeostasis	catalytic activity	Putative uncharacterized protein
IPI00305978.4	AKR7A2	X	X		cytoplasm,Golgi	metabolic process	catalytic activity	Aflatoxin B1 aldehyde reductase member 2
IPI00005474.5	LHPP	X	X	X	cytoplasm,organelle lumen,cytosol,nucleus	metabolic process	protein binding,metal ion binding,catalytic activity	Isoform 1 of Phospholysine phosphohistidine inorganic pyrophosphate phosphatase
IPI00011302.1	CD59	X	X	X	extracellular,cell surface,membrane	regulation of biological process,response to stimulus,cell communication,coagulation	protein binding	CD59 glycoprotein
IPI00031506.3	HCN1	X	X	X	membrane	development,transport,regulation of biological process,cell communication,cell differentiation	transporter activity,nucleotide binding	Potassium/sodium hyperpolarization-activated cyclic nucleotide-gated channel 1
IPI00220150.4	IDH3G	X	X		mitochondrion,cytoplasm,organelle lumen,nucleus	metabolic process,regulation of biological process	metal ion binding,nucleotide binding,catalytic activity	Isocitrate dehydrogenase [NAD] subunit gamma, mitochondrial
IPI00013930.1	STX6		X		membrane,cytoplasm,Golgi,endosome	cell organization and biogenesis,transport	protein binding	Syntaxin-6
IPI00025366.4	CS	X	X		mitochondrion,cytoplasm,organelle lumen	metabolic process	catalytic activity	Citrate synthase, mitochondrial
IPI00016112.6	PXDN			X	extracellular,endoplasmic reticulum,cytoplasm	cell organization and biogenesis,metabolic process,regulation of biological process,response to stimulus,cell communication	antioxidant activity,protein binding,metal ion binding,structural molecule activity,catalytic activity	Isoform 1 of Peroxidasin homolog
IPI00296485.6	MAP1S	X	X		cytoskeleton,cytoplasm,nucleus,cytosol	cell death,cell organization and biogenesis,development,transport,cellular component movement,cell differentiation	protein binding,DNA binding,catalytic activity	Microtubule-associated protein 1S
IPI00008787.4	NAGLU			X	cytoplasm,vacuole	cell organization and biogenesis,development,metabolic process,response to stimulus,cell differentiation	catalytic activity	Alpha-N-acetylglucosaminidase
IPI00026670.3	TCEB2	X	X		cytoplasm,organelle lumen,cytosol,nucleus	cell organization and biogenesis,metabolic process,regulation of biological process,reproduction	protein binding	Transcription elongation factor B polypeptide 2
IPI00334743.4	CCNY	X	X			metabolic process,regulation of biological process	protein binding	Isoform 2 of Cyclin-Y
IPI00019372.2	SRGN			X	extracellular,membrane,cytoplasm,Golgi,vacuole,organelle lumen	cell death,cell organization and biogenesis,development,metabolic process,transport,regulation of biological process,response to stimulus,coagulation	protein binding	Serglycin
IPI00217950.5	HMGN2	X	X		cytoplasm,chromosome,nucleus	cell organization and biogenesis,metabolic process,regulation of biological process	DNA binding	Non-histone chromosomal protein HMG-17
IPI00044388.3	INPP4A		X					Isoform 3 of Type I inositol-3,4-bisphosphate 4-phosphatase
IPI00744811.2	LRP5			X	membrane,mitochondrion,endoplasmic reticulum,cytoplasm	cell death,cell proliferation,development,cell organization and biogenesis,transport,metabolic process,regulation of biological process,response to stimulus,cellular component movement,cell communication,cellular homeostasis,cell differentiation	signal transducer activity,protein binding,transporter activity,receptor activity	Low-density lipoprotein receptor-related protein 5
IPI00291643.4	SPRYD4		X		mitochondrion,cytoplasm,nucleus		protein binding	SPRY domain-containing protein 4
IPI00296992.7	AXL			X	extracellular,cell surface,membrane	cell death,cell organization and biogenesis,development,metabolic process,regulation of biological process,response to stimulus,defense response,reproduction,transport,cellular component movement,cell communication,cell differentiation,coagulation	protein binding,signal transducer activity,nucleotide binding,receptor activity,catalytic activity	Isoform Long of Tyrosine-protein kinase receptor UFO
IPI00298793.4	MANBA			X	cytoplasm,vacuole	metabolic process	catalytic activity	Beta-mannosidase
IPI00160369.5	PICK1			X	cytoskeleton,membrane,mitochondrion,cytoplasm,Golgi	cell organization and biogenesis,development,metabolic process,transport,regulation of biological process,response to stimulus,cell communication,cell differentiation,reproduction	protein binding,metal ion binding,catalytic activity	PRKCA-binding protein
IPI00026314.1	GSN			X	extracellular,cytoskeleton,cytoplasm,cytosol	cell death,development,cell organization and biogenesis,transport,regulation of biological process,response to stimulus,cell communication,cell differentiation	protein binding,metal ion binding	Isoform 1 of Gelsolin
IPI00013508.5	ACTN1			X	extracellular,cytoskeleton,membrane,cytoplasm,organelle lumen,cytosol	cell death,cell organization and biogenesis,transport,regulation of biological process,response to stimulus,cellular component movement,coagulation	protein binding,metal ion binding	Isoform 1 of Alpha-actinin-1

IPI	GENE	Alzheimer's Hippocampus	Control hippocampus	CSF	Cellular localization	Biological process	Molecular function	Protein Description
IPI00185146.5	IPO9	X	X	X	cytoplasm,nucleus	transport	protein binding,transporter activity	Importin-9
IPI00027032.1	LYPLA2		X		cytoplasm	metabolic process	catalytic activity	Acyl-protein thioesterase 2
IPI00550533.2	C1orf56			X	extracellular			Isoform 1 of Uncharacterized protein C1orf56
IPI00168112.2	PDDC1	X	X		extracellular			Isoform 1 of Parkinson disease 7 domain-containing protein 1
IPI00028095.3	FHIT	X	X		membrane,cytoplasm,cytosol,nucleus	metabolic process	protein binding,metal ion binding,catalytic activity	Bis(5'-adenosyl)-triphosphatase
IPI00021926.4	PSMC6	X	X		cytoplasm,proteasome,organelle lumen,nucleus,cytosol	cell death,metabolic process,regulation of biological process,response to stimulus,cell communication	protein binding,RNA binding,nucleotide binding,catalytic activity	26S protease regulatory subunit 10B
IPI00465325.5	LINGO1	X	X	X	membrane	cell organization and biogenesis,development,regulation of biological process,response to stimulus,cell communication,cell differentiation	protein binding	Isoform 1 of Leucine-rich repeat and immunoglobulin-like domain-containing nogo receptor-interacting protein 1
IPI00012575.1	PIR	X	X		cytoplasm,nucleus	development,metabolic process,cell differentiation	protein binding,metal ion binding,catalytic activity	Pirin
IPI00180954.4	CIRBP	X	X		cytoplasm,organelle lumen,nucleus	cell organization and biogenesis,metabolic process,regulation of biological process,response to stimulus	protein binding,RNA binding,nucleotide binding,translation regulator activity	cold-inducible RNA-binding protein
IPI00430842.3	IGHA1			X			protein binding	IGHA1 protein
IPI00871227.1	HMCN1			X	extracellular,cell surface,membrane,cytoplasm	response to stimulus	protein binding,metal ion binding	Isoform 1 of Hemicentin-1
IPI00216882.4	MASP1			X			metal ion binding	Isoform 3 of Mannan-binding lectin serine protease 1
IPI00167089.1	TNK2			X		metabolic process	protein binding,nucleotide binding,catalytic activity	Isoform 2 of Activated CDC42 kinase 1
IPI00013096.4	PTPRT			X	membrane	metabolic process	protein binding,catalytic activity	Isoform 1 of Receptor-type tyrosine-protein phosphatase T
IPI00375364.1	CHIT1			X	extracellular	metabolic process	catalytic activity	Isoform 3 of Chitotriosidase-1
IPI00400986.7	KIAA1731			X	cytoskeleton,cytoplasm			Isoform 1 of Protein KIAA1731
IPI00220030.2	PXN	X					metal ion binding	Isoform Alpha of Paxillin
IPI00550021.4	RPL3	X	X		cytoplasm,ribosome,organelle lumen,cytosol,nucleus	cell organization and biogenesis,metabolic process,transport,reproduction	RNA binding,structural molecule activity	60S ribosomal protein L3
IPI00034049.1	UPF1	X	X		cytoplasm,chromosome,nucleus,cytosol	cell organization and biogenesis,metabolic process,transport,regulation of biological process,response to stimulus	protein binding,DNA binding,RNA binding,metal ion binding,nucleotide binding,catalytic activity	Isoform 1 of Regulator of nonsense transcripts 1
IPI00177878.9	ANO4			X				Isoform 3 of Anoctamin-4
IPI00001699.1	PYCARD	X			cytoplasm,cytosol	cell death,metabolic process,transport,regulation of biological process,response to stimulus,cell communication,defense response	protein binding,catalytic activity,enzyme regulator activity	Isoform 1 of Apoptosis-associated speck-like protein containing a CARD
IPI00402144.4	ZNF555			X	nucleus	metabolic process,regulation of biological process	DNA binding,metal ion binding	Isoform 1 of Zinc finger protein 555
IPI00000775.3	LRIG1			X	membrane		protein binding	Isoform 1 of Leucine-rich repeats and immunoglobulin-like domains protein 1
IPI00166060.1	LGI4		X		extracellular	development,response to stimulus,cell communication,cellular homeostasis,cell differentiation	protein binding	Isoform 1 of Leucine-rich repeat LGI family member 4
IPI01012111.1	RPL29P11			X	cytoplasm,ribosome	metabolic process	structural molecule activity	Similar to 60S ribosomal protein L29
IPI00747268.1	PPP2R4			X			enzyme regulator activity	serine/threonine-protein phosphatase 2A activator isoform b
IPI00027107.5	TUFM		X			metabolic process	RNA binding,nucleotide binding,catalytic activity	elongation factor Tu, mitochondrial precursor
IPI00022431.2	AHSG	X	X	X	extracellular,membrane		enzyme regulator activity	cDNA FLJ55606, highly similar to Alpha-2-HS-glycoprotein
IPI00815727.4	LOC642131			X				putative V-set and immunoglobulin domain-containing protein 6-like
IPI00027984.4	DNAJC11			X		metabolic process	protein binding	Uncharacterized protein
IPI00293857.1	ARRB1	X	X		membrane,cytoplasm,Golgi,vacuole,chromosome,cytosol,nucleus	cell organization and biogenesis,metabolic process,transport,regulation of biological process,response to stimulus,cell communication,coagulation	protein binding,DNA binding,catalytic activity,enzyme regulator activity	Isoform 1A of Beta-arrestin-1

IPI	GENE	Alzheimer's Hippocampus	Control hippocampus	CSF	Cellular localization	Biological process	Molecular function	Protein Description
IPI00002824.7	CSRP2	X			nucleus	development,cell differentiation	metal ion binding	Cysteine and glycine-rich protein 2
IPI00328746.3	RTN4RL2			X	cell surface,membrane	cell organization and biogenesis,development,response to stimulus,cell differentiation	receptor activity	Reticulon-4 receptor-like 2
IPI00000051.4	PFDN1		X		cytoplasm,cytosol	cell organization and biogenesis,development,metabolic process,regulation of biological process	protein binding	Prefoldin subunit 1
IPI00293757.4	UNC5C			X	membrane	cell death,cell organization and biogenesis,development,regulation of biological process,response to stimulus,cellular component movement,cell communication,cell differentiation	signal transducer activity,protein binding,receptor activity	Isoform 1 of Netrin receptor UNC5C
IPI00017292.1	CTNNB1	X	X		cytoskeleton,membrane,cytoplasm,organelle lumen,cytosol,nucleus	cell death,cell proliferation,development,cell organization and biogenesis,transport,metabolic process,regulation of biological process,response to stimulus,cell communication,cellular homeostasis,cell differentiation,reproduction	protein binding,signal transducer activity,DNA binding,structural molecule activity	Isoform 1 of Catenin beta-1
IPI00007402.3	IPO7	X	X	X	membrane,cytoplasm,Golgi,nucleus	transport,regulation of biological process,response to stimulus,cell communication	protein binding,transporter activity,enzyme regulator activity	Importin-7
IPI00034159.1	ATP6V0D1	X	X		membrane,cytoplasm,vacuole,endosome	development,metabolic process,transport,regulation of biological process,response to stimulus,cell communication,cellular homeostasis	protein binding,transporter activity,catalytic activity	V-type proton ATPase subunit d 1
IPI00183274.3	SNX1	X	X			transport,cell communication	protein binding,transporter activity	Uncharacterized protein
IPI00024802.1	BTAF1			X	nucleus	metabolic process,regulation of biological process	DNA binding,nucleotide binding,catalytic activity	TATA-binding protein-associated factor 172
IPI00000144.1	OXT			X	extracellular,cytoplasm	development,cell organization and biogenesis,transport,regulation of biological process,response to stimulus,cell communication,cellular homeostasis,reproduction	protein binding	Oxytocin-neurophysin 1
IPI00747200.2	C6orf203		X		mitochondrion,cytoplasm			Uncharacterized protein C6orf203
IPI00060715.1	KCTD12	X	X	X	membrane	cell organization and biogenesis	protein binding	BTB/POZ domain-containing protein KCTD12
IPI00641829.5	DDX39B	X	X				nucleotide binding,catalytic activity	Isoform 2 of Spliceosome RNA helicase DDX39B
IPI00018188.3	NECAP2	X			membrane,cytoplasm	transport		Isoform 1 of Adaptin ear-binding coat-associated protein 2
IPI00152769.4	TRPC4AP			X		metabolic process	protein binding	Isoform 1 of Short transient receptor potential channel 4-associated protein
IPI00184533.1	USP11		X		cytoplasm,nucleus	metabolic process	protein binding,catalytic activity	Ubiquitin carboxyl-terminal hydrolase 11
IPI00015688.2	GPC1		X	X	extracellular,membrane,cytoplasm,endosome	cell organization and biogenesis,development,metabolic process,regulation of biological process,response to stimulus,cell communication,cellular homeostasis,cell differentiation	protein binding,metal ion binding	Glypican-1
IPI00024580.4	MCCC1		X	X	mitochondrion,membrane,cytoplasm,organelle lumen	metabolic process	metal ion binding,nucleotide binding,catalytic activity	Methylcrotonoyl-CoA carboxylase subunit alpha, mitochondrial
IPI00010779.4	TPM4	X	X		cytoskeleton,cytoplasm,cytosol	response to stimulus,cellular component movement	protein binding,metal ion binding,structural molecule activity	Isoform 1 of Tropomyosin alpha-4 chain
IPI00640524.4	CAMSAP3	X	X				protein binding	Isoform 2 of Calmodulin-regulated spectrin-associated protein 3
IPI00011107.2	IDH2	X	X		membrane,mitochondrion,cytoplasm,organelle lumen	metabolic process	metal ion binding,nucleotide binding,catalytic activity	Isocitrate dehydrogenase [NADP], mitochondrial
IPI00029009.4	PIP5K1C		X			metabolic process	catalytic activity	Uncharacterized protein
IPI00009737.1	RRAGD	X			cytoplasm,vacuole,nucleus	regulation of biological process,response to stimulus,cell communication	protein binding,nucleotide binding	Isoform 1 of Ras-related GTP-binding protein D
IPI00296461.4	SMPD1			X	extracellular,cytoplasm,vacuole,organelle lumen	cell death,development,metabolic process,regulation of biological process,response to stimulus,cell communication	catalytic activity	Isoform 1 of Sphingomyelin phosphodiesterase
IPI00102670.7	FNBP1	X	X				protein binding	Isoform 2 of Formin-binding protein 1
IPI00032939.13	WIPF3	X	X		cytoplasm,organelle lumen,nucleus	development,cell differentiation,reproduction	protein binding	WAS/WASL-interacting protein family member 3
IPI00442171.4	PREPL	X	X		cytoplasm,cytosol	metabolic process	catalytic activity	Isoform 1 of Prolyl endopeptidase-like
IPI00386314.1	CNDP2			X		metabolic process	catalytic activity	FLJ00064 protein (Fragment)
IPI00061376.5	ELP4	X	X		cytoplasm,organelle lumen,nucleus	cell organization and biogenesis,metabolic process,regulation of biological process	protein binding,catalytic activity,enzyme regulator activity	Isoform 1 of Elongator complex protein 4

IPI	GENE	Alzheimer's Hippocampus	Control hippocampus	CSF	Cellular localization	Biological process	Molecular function	Protein Description
IPI00220637.5	SARS	X	X		mitochondrion,cytoplasm,cytosol	metabolic process	RNA binding,nucleotide binding,catalytic activity	Seryl-tRNA synthetase, cytoplasmic
IPI00451624.1	CRTAC1			X	extracellular		metal ion binding	Isoform 1 of Cartilage acidic protein 1
IPI00301631.5	TOR3A			X	endoplasmic reticulum,cytoplasm,organelle lumen	metabolic process	nucleotide binding	Isoform 1 of Torsin-3A
IPI00658025.1	SZT2			X				Uncharacterized protein
IPI00795918.1	NCAM1	X	X	X	extracellular,cell surface,membrane,cytoplasm,Golgi	cell organization and biogenesis,development,regulation of biological process,response to stimulus,defense response,cell communication,cell differentiation	protein binding	Isoform 1 of Neural cell adhesion molecule 1
IPI00016014.1	ITM2C		X	X	membrane,cytoplasm,Golgi,vacuole	cell death,cell organization and biogenesis,development,regulation of biological process,cell differentiation	protein binding,nucleotide binding	Isoform 1 of Integral membrane protein 2C
IPI00012069.1	NQO1	X			cytoplasm,cytosol	cell death,metabolic process,regulation of biological process,response to stimulus,cell communication	protein binding,catalytic activity	NAD(P)H dehydrogenase [quinone] 1
IPI00385918.1	ZNF627			X				cDNA FLJ90582 fis, clone PLACE1000442, moderately similar to ZINC FINGER PROTEIN ZFP-36
IPI00032103.3	GATM	X	X		membrane,mitochondrion,cytoplasm	development,metabolic process,response to stimulus	catalytic activity	Isoform 1 of Glycine amidinotransferase, mitochondrial
IPI00023958.1	MPV17	X	X		mitochondrion,membrane,cytoplasm	development,cell organization and biogenesis,metabolic process,regulation of biological process,response to stimulus		Protein Mpv17
IPI00016666.1	MT3	X	X	X	cytoplasm	cell death,cell proliferation,development,cell organization and biogenesis,metabolic process,transport,regulation of biological process,response to stimulus,cell communication,cellular homeostasis,cell differentiation,cell growth	antioxidant activity,protein binding,metal ion binding,catalytic activity,enzyme regulator activity	Metallothionein-3
IPI00166729.4	AZGP1			X	extracellular,membrane	cell proliferation,metabolic process,regulation of biological process,response to stimulus	protein binding,transporter activity,catalytic activity	Zinc-alpha-2-glycoprotein
IPI00021794.8	CTSA			X	mitochondrion,endoplasmic reticulum,cytoplasm,organelle lumen,vacuole,nucleus	transport,metabolic process,regulation of biological process	catalytic activity,enzyme regulator activity	Lysosomal protective protein
IPI00925804.1	AIP	X			membrane,cytoplasm,cytosol	metabolic process,regulation of biological process,response to stimulus,cell communication	protein binding,receptor activity	Non-functional aryl hydrocarbon receptor interacting protein (Fragment)
IPI00748037.4	NAPB	X	X		membrane	cell organization and biogenesis,transport,regulation of biological process,cell communication	protein binding	cDNA FLJ54102, highly similar to Beta-soluble NSF attachment protein
IPI00384600.3	EFR3B		X					Isoform 2 of Protein EFR3 homolog B
IPI00028516.1	KIAA0513		X		cytoplasm			Isoform 1 of Uncharacterized protein KIAA0513
IPI00103175.1	CANT1			X	membrane,endoplasmic reticulum,cytoplasm,Golgi	regulation of biological process,response to stimulus,cell communication	signal transducer activity,metal ion binding,catalytic activity	Isoform 1 of Soluble calcium-activated nucleotidase 1
IPI00328826.8	CADPS2			X			protein binding	Isoform 3 of Calcium-dependent secretion activator 2
IPI00001399.1	AJAP1			X	membrane	metabolic process	catalytic activity	Adherens junction-associated protein 1
IPI00647400.1	RNPEP	X	X		extracellular	metabolic process	metal ion binding,catalytic activity	Uncharacterized protein
IPI00018671.1	DUSP3	X	X		membrane,cytoplasm,organelle lumen,cytosol,nucleus	development,metabolic process,regulation of biological process,response to stimulus,defense response,cell communication	catalytic activity	Dual specificity protein phosphatase 3
IPI00305166.2	SDHA	X	X			metabolic process	catalytic activity	cDNA FLJ61478, highly similar to Succinate dehydrogenase (ubiquinone) flavoprotein subunit, mitochondrial
IPI00306322.2	COL4A2			X	extracellular	development,cell organization and biogenesis,metabolic process,regulation of biological process,response to stimulus,cell differentiation	protein binding,structural molecule activity	Collagen alpha-2(IV) chain
IPI00220301.5	PRDX6	X	X	X	cytoplasm,vacuole,cytosol	metabolic process,response to stimulus	antioxidant activity,protein binding,catalytic activity	Peroxiredoxin-6
IPI00298476.3	GREM1			X	extracellular,cell surface	cell death,cell proliferation,cell organization and biogenesis,development,transport,metabolic process,regulation of biological process,response to stimulus,cellular component movement,cell communication,cell growth,cell differentiation	protein binding	Isoform 1 of Gremlin-1
IPI00748312.2	PTPRZ1	X	X	X	extracellular,membrane	cell organization and biogenesis,development,metabolic process,cell differentiation	protein binding,signal transducer activity,receptor activity,catalytic activity	Isoform Long of Receptor-type tyrosine-protein phosphatase zeta
IPI00647027.1	CHGB			X	cytoplasm			32 kDa protein

IPI	GENE	Alzheimer's Hippocampus	Control hippocampus	CSF	Cellular localization	Biological process	Molecular function	Protein Description
IPI00163185.9	AGAP3	X	X			metabolic process,regulation of biological process,response to stimulus,cell communication	protein binding,metal ion binding,nucleotide binding,enzyme regulator activity	Isoform 2 of Arf-GAP with GTPase, ANK repeat and PH domain-containing protein 3
IPI00297550.8	F13A1		X	X	extracellular,cytoplasm,organelle lumen	metabolic process,transport,response to stimulus,coagulation	metal ion binding,catalytic activity	Coagulation factor XIII A chain
IPI00005153.3	OBP2A			X	extracellular	transport,response to stimulus		Isoform Aa of Odorant-binding protein 2a
IPI00219129.10	NQO2	X	X	X	cytoplasm,organelle lumen,nucleus	metabolic process	metal ion binding,catalytic activity	Ribosyldihydronicotinamide dehydrogenase [quinone]
IPI00100199.4	CYBRD1	X			membrane	transport,metabolic process,response to stimulus,cellular homeostasis	metal ion binding,catalytic activity	Cytochrome b reductase 1
IPI00029733.1	AKR1C1	X	X		cytoplasm,cytosol	cell organization and biogenesis,metabolic process,transport,response to stimulus	catalytic activity	Aldo-keto reductase family 1 member C1
IPI00029012.1	EIF3A	X	X	X	cytoplasm,organelle lumen,nucleus,cytosol	cell organization and biogenesis,metabolic process	protein binding,RNA binding,structural molecule activity	Eukaryotic translation initiation factor 3 subunit A
IPI00012441.4	SYNJ1	X	X	X	membrane,cytoplasm	development,cell organization and biogenesis,transport,metabolic process,regulation of biological process,response to stimulus,cell communication,cell differentiation	protein binding,RNA binding,nucleotide binding,catalytic activity	Isoform 1 of Synaptojanin-1
IPI00785084.2	IGHV4-31			X	membrane		protein binding	IGH@ protein
IPI00332271.7	PTPRS			X	membrane	metabolic process	protein binding,catalytic activity	Isoform PTPS-MEA of Receptor-type tyrosine-protein phosphatase S
IPI00215901.1	AK2	X	X		mitochondrion,membrane,cytoplasm	metabolic process	nucleotide binding,catalytic activity	Isoform 1 of Adenylate kinase 2, mitochondrial
IPI00219757.13	GSTP1	X	X	X	membrane,mitochondrion,cytoplasm,cytosol,nucleus	cell death,cell proliferation,development,metabolic process,regulation of biological process,response to stimulus,cell communication,defense response,cell differentiation	protein binding,catalytic activity,enzyme regulator activity	Glutathione S-transferase P
IPI00013475.1	TUBB2A	X	X	X	cytoskeleton,cytoplasm	development,cell organization and biogenesis,metabolic process,cellular component movement,cell differentiation	nucleotide binding,structural molecule activity,catalytic activity	Tubulin beta-2A chain
IPI00555830.1	MST1			X	extracellular	metabolic process	catalytic activity	Hepatocyte growth factor-like protein
IPI00419237.3	LAP3	X	X	X	mitochondrion,cytoplasm,organelle lumen,nucleus	metabolic process	metal ion binding,catalytic activity	Isoform 1 of Cytosol aminopeptidase
IPI00419979.3	PAK2	X	X			metabolic process	protein binding,nucleotide binding,catalytic activity	Serine/threonine-protein kinase PAK 2
IPI00249656.1	MURC	X			cytoplasm	development,metabolic process,regulation of biological process,cell differentiation		Muscle-related coiled-coil protein
IPI00179463.8	ATCAY		X			transport	protein binding	Isoform 1 of Caytaxin
IPI00641920.1	NECAB2	X	X		cytoplasm		protein binding,metal ion binding	N-terminal EF-hand calcium-binding protein 2
IPI00152418.2	CD55			X	membrane			Decay-accelerating factor splicing variant 4
IPI00297284.1	IGFBP2			X	extracellular	cell organization and biogenesis,regulation of biological process,cell growth	protein binding	insulin-like growth factor-binding protein 2 precursor
IPI00007199.5	SERPINA10			X	extracellular	transport,metabolic process,regulation of biological process,response to stimulus,reproduction	enzyme regulator activity	Protein Z-dependent protease inhibitor
IPI00220362.5	HSPE1	X	X	X	mitochondrion,cytoplasm,organelle lumen	cell death,metabolic process,regulation of biological process,response to stimulus	protein binding,nucleotide binding	10 kDa heat shock protein, mitochondrial
IPI00001593.1	PRCP	X	X	X	membrane,cytoplasm,vacuole	metabolic process,response to stimulus,coagulation	protein binding,catalytic activity	Lysosomal Pro-X carboxypeptidase
IPI00015913.1	TYRP1			X	membrane,cytoplasm,endosome	cell organization and biogenesis,metabolic process,cell differentiation	protein binding,metal ion binding,catalytic activity	5,6-dihydroxyindole-2-carboxylic acid oxidase
IPI00002478.1	ECE1			X	cell surface,membrane,cytoplasm,endosome	cell death,development,metabolic process,regulation of biological process,response to stimulus,cell communication	protein binding,metal ion binding,catalytic activity	Isoform B of Endothelin-converting enzyme 1
IPI00289758.7	CAPN2	X	X		membrane,cytoplasm,chromosome,nucleus	development,metabolic process,response to stimulus,cell differentiation	protein binding,metal ion binding,catalytic activity	Calpain-2 catalytic subunit
IPI00180154.4	ATXN2		X		cytoplasm,Golgi,organelle lumen,nucleus	cell death,cell organization and biogenesis,development,metabolic process,transport,regulation of biological process,cell differentiation	protein binding,RNA binding	Isoform 1 of Ataxin-2
IPI00028714.4	MGP			X	extracellular,endoplasmic reticulum,cytoplasm	development,regulation of biological process,response to stimulus,cell differentiation	protein binding,metal ion binding,structural molecule activity	Matrix Gla protein
IPI00220766.5	GLO1	X	X	X	cytoplasm	cell death,metabolic process,regulation of biological process	metal ion binding,catalytic activity	Isoform 1 of Lactoylglutathione lyase
IPI00792677.1	TUBA1B			X	cytoskeleton	cell organization and biogenesis,metabolic process,cellular component movement	nucleotide binding,structural molecule activity,catalytic activity	alpha tubulin, isoform CRA_b

IPI	GENE	Alzheimer's Hippocampus	Control hippocampus	CSF	Cellular localization	Biological process	Molecular function	Protein Description
IPI00292858.4	SCO2		X	X	cytoplasm,cytosol	cell organization and biogenesis,development,metabolic process,response to stimulus,cell differentiation	protein binding,catalytic activity	Thymidine phosphorylase
IPI00005914.5	SHPK	X	X		cytoplasm	metabolic process	nucleotide binding,catalytic activity	Sedoheptulokinase
IPI00479125.3	SRGAP2	X	X	X	cytoskeleton,membrane,cytoplasm,cytosol	cell death,cell proliferation,cell organization and biogenesis,development,metabolic process,regulation of biological process,response to stimulus,cellular component movement,cell communication,cell differentiation	protein binding,enzyme regulator activity	SLIT-ROBO Rho GTPase-activating protein 2
IPI00023640.3	PDCD5	X	X		cytoplasm,nucleus	cell death,regulation of biological process	DNA binding	Programmed cell death protein 5
IPI00023591.1	PURA	X	X		cytoplasm,organelle lumen,chromosome,nucleus	cell proliferation,development,metabolic process,regulation of biological process	protein binding,DNA binding,translation regulator activity	Transcriptional activator protein Pur-alpha
IPI00023330.3	KIAA1468		X					Isoform 2 of LisH domain and HEAT repeat-containing protein KIAA1468
IPI00479185.1	TPM3	X	X			response to stimulus,cell communication	structural molecule activity	tropomyosin alpha-3 chain isoform 4
IPI00005707.7	MRC2			X	membrane	transport	receptor activity	C-type mannose receptor 2
IPI00002756.1	GABBR1	X	X		extracellular,membrane,mitochondrion,endoplasmic reticulum,cytoplasm	cell proliferation,transport,metabolic process,regulation of biological process,response to stimulus,cell communication,cell differentiation	protein binding,signal transducer activity,receptor activity	Isoform 1A of Gamma-aminobutyric acid type B receptor subunit 1
IPI00020986.2	LUM		X	X	extracellular	development,cell organization and biogenesis,response to stimulus	protein binding,structural molecule activity	Lumican
IPI00010381.2	SORCS3			X	membrane	regulation of biological process,response to stimulus,cell communication	signal transducer activity,receptor activity	VPS10 domain-containing receptor SorCS3
IPI00024157.1	FKBP3	X	X		membrane,nucleus	metabolic process	receptor activity,catalytic activity	Peptidyl-prolyl cis-trans isomerase FKBP3
IPI00748955.4	GP1BA			X	cell surface,membrane	cell organization and biogenesis	protein binding	Platelet glycoprotein Ib alpha polypeptide (Fragment)
IPI00031641.4	TMEM38A		X		membrane,endoplasmic reticulum,cytoplasm,nucleus	transport	transporter activity	Trimeric intracellular cation channel type A
IPI00027996.2	CORO7	X	X		cytoskeleton,membrane,cytoplasm,Golgi,cytosol	cell organization and biogenesis	protein binding	Isoform 1 of Coronin-7
IPI00411453.3	AP3D1	X	X		membrane,cytoplasm,Golgi,endosome	development,cell organization and biogenesis,metabolic process,transport,regulation of biological process,cellular homeostasis,cell differentiation	transporter activity	Isoform 1 of AP-3 complex subunit delta-1
IPI00553006.1	PARS2		X		mitochondrion,cytoplasm,organelle lumen	metabolic process	nucleotide binding,catalytic activity	Probable prolyl-tRNA synthetase, mitochondrial
IPI00550115.3	SMPDL3B			X	extracellular	metabolic process	catalytic activity	Isoform 1 of Acid sphingomyelinase-like phosphodiesterase 3b
IPI00301180.4	SLC12A5	X	X	X	membrane	transport	transporter activity	Isoform 2 of Solute carrier family 12 member 5
IPI00217563.4	ITGB1			X	extracellular,cell surface,membrane,cytoplasm	cell death,development,cell organization and biogenesis,metabolic process,regulation of biological process,response to stimulus,defense response,reproduction,cell proliferation,transport,cellular component movement,cell communication,cellular homeostasis,cell differentiation,coagulation	protein binding,receptor activity	Isoform Beta-1A of Integrin beta-1
IPI00007709.3	ADAM28			X	extracellular,membrane	metabolic process,reproduction	protein binding,metal ion binding,catalytic activity	Isoform 1 of Disintegrin and metalloproteinase domain-containing protein 28
IPI00645206.2	PCDH17			X	membrane		protein binding,metal ion binding	Isoform 1 of Protocadherin-17
IPI00300623.2	PMCH			X	extracellular,nucleus	development,transport,regulation of biological process,response to stimulus,cell communication,cellular homeostasis,cell differentiation,reproduction	protein binding	Pro-MCH
IPI00009032.1	SSB	X			nucleus	metabolic process	RNA binding,nucleotide binding	Lupus La protein
IPI00290461.3	EIF3J	X	X		cytoplasm,cytosol	metabolic process	protein binding,RNA binding	Eukaryotic translation initiation factor 3 subunit J
IPI00294627.3	SF1	X	X				RNA binding,metal ion binding	Isoform 2 of Splicing factor 1
IPI00299571.5	PDIA6	X	X	X		regulation of biological process,cellular homeostasis		Isoform 2 of Protein disulfide-isomerase A6

IPI	GENE	Alzheimer's Hippocampus	Control hippocampus	CSF	Cellular localization	Biological process	Molecular function	Protein Description
IPI00982478.1	PTK2	X	X		cytoskeleton,membrane,cytoplasm,nucleus,cytosol	cell death,cell proliferation,cell organization and biogenesis,development,metabolic process,regulation of biological process,response to stimulus,cellular component movement,cell communication,cell differentiation,coagulation	signal transducer activity,protein binding,nucleotide binding,catalytic activity	Isoform 1 of Focal adhesion kinase 1
IPI00013991.1	TPM2			X	cytoskeleton,cytoplasm,cytosol	metabolic process,regulation of biological process,cellular component movement	protein binding,structural molecule activity	Isoform 1 of Tropomyosin beta chain
IPI00872352.1	HDDC2	X	X		mitochondrion,cytoplasm	metabolic process	metal ion binding,catalytic activity	Isoform 1 of HD domain-containing protein 2
IPI01011005.1	NTM			X				Isoform 2 of Neurotrimin
IPI00643733.2	MTMR1		X		membrane	metabolic process	catalytic activity	Uncharacterized protein
IPI00815786.1	HK1			X		metabolic process	catalytic activity	Hexokinase 1 (Fragment)
IPI00001539.8	ACAA2	X	X		membrane,mitochondrion,cytoplasm	cell death,metabolic process,regulation of biological process	protein binding,catalytic activity	3-ketoacyl-CoA thiolase, mitochondrial
IPI00237011.5	STK38L		X		cytoskeleton,membrane,cytoplasm,organelle lumen,nucleus	cell organization and biogenesis,metabolic process,regulation of biological process,response to stimulus,cell communication	protein binding,metal ion binding,nucleotide binding,catalytic activity	Serine/threonine-protein kinase 38-like
IPI00100668.3	GBA2		X		membrane	metabolic process	catalytic activity	Non-lysosomal glucosylceramidase
IPI00166394.1	ARMC10		X		membrane,mitochondrion,endoplasmic reticulum,cytoplasm	regulation of biological process		Isoform 1 of Armadillo repeat-containing protein 10
IPI00924657.1	CHL1	X	X	X	membrane		protein binding	Protein
IPI00645805.4	IVD	X	X		membrane,mitochondrion,cytoplasm,organelle lumen	metabolic process	catalytic activity	Isovaleryl-CoA dehydrogenase, mitochondrial
IPI00409659.2	UBQLN2	X	X		membrane,cytoplasm,nucleus	cell death	protein binding	Ubiquilin-2
IPI00025796.3	NDUFS3	X	X		mitochondrion,membrane,cytoplasm	cell death,cell organization and biogenesis,metabolic process,transport,regulation of biological process,cell growth	protein binding,catalytic activity	NADH dehydrogenase [ubiquinone] iron-sulfur protein 3, mitochondrial
IPI00018081.8	FLJ22184		X			metabolic process	structural molecule activity,catalytic activity	Putative uncharacterized protein FLJ22184
IPI00168520.6	MATN2			X			protein binding	Isoform 2 of Matrilin-2
IPI00028931.2	DSG2			X	membrane	cell death,cell organization and biogenesis	metal ion binding	Desmoglein-2
IPI00016736.1	PLCG1	X	X		membrane,cytoplasm,cytosol	cell organization and biogenesis,development,metabolic process,regulation of biological process,response to stimulus,cellular component movement,cell communication,cell differentiation,coagulation	signal transducer activity,protein binding,metal ion binding,catalytic activity	Isoform 1 of 1-phosphatidylinositol-4,5-bisphosphate phosphodiesterase gamma-1
IPI00022433.5	HSPB6	X	X					Heat shock protein beta-6
IPI00216651.3	IL28RA			X	membrane	cell proliferation,regulation of biological process,response to stimulus,defense response,cell communication	signal transducer activity,protein binding,receptor activity	Isoform 1 of Interleukin-28 receptor subunit alpha
IPI00747494.2	GRID2			X	cytoskeleton,membrane	cell death,transport,regulation of biological process,response to stimulus,cell communication,cellular homeostasis	signal transducer activity,protein binding,transporter activity,receptor activity	Glutamate receptor delta-2 subunit
IPI00030182.1	GAMT	X	X		cytoplasm,cytosol	metabolic process	catalytic activity	Guanidinoacetate N-methyltransferase
IPI00296053.3	FH	X	X		mitochondrion,cytoplasm,organelle lumen	metabolic process	catalytic activity	Isoform Mitochondrial of Fumarate hydratase, mitochondrial
IPI00015315.1	ECM2			X	extracellular	cell organization and biogenesis,regulation of biological process	protein binding	Isoform 1 of Extracellular matrix protein 2
IPI00295469.5	CPNE6	X	X	X	membrane		protein binding	cDNA FLJ55997, highly similar to Copine-6
IPI00183445.1	LPHN1		X	X	cytoskeleton,membrane	development,regulation of biological process,response to stimulus,cell communication	protein binding,signal transducer activity,receptor activity	Isoform 1 of Latrophilin-1
IPI00217652.5	GXYLT1			X	membrane	metabolic process	catalytic activity	Isoform 1 of Glucoside xylosyltransferase 1
IPI00015836.1	APPL1	X	X		membrane,cytoplasm,organelle lumen,cytosol,nucleus,endosome	cell death,cell proliferation,cell organization and biogenesis,transport,regulation of biological process,response to stimulus,cell communication	protein binding	DCC-interacting protein 13-alpha
IPI00022827.1	SLK		X		membrane,cytoplasm	cell death,metabolic process	protein binding,nucleotide binding,catalytic activity	Isoform 1 of STE20-like serine/threonine-protein kinase
IPI00023152.4	NAALADL1			X	membrane	metabolic process	metal ion binding,catalytic activity	Isoform 1 of N-acetylated-alpha-linked acidic dipeptidase-like protein

IPI	GENE	Alzheimer's Hippocampus	Control hippocampus	CSF	Cellular localization	Biological process	Molecular function	Protein Description
IPI00295172.5	NINJ1			X	membrane	development,response to stimulus		Ninjurin-1
IPI00245940.3	IGSF5			X	cell surface,membrane		protein binding	Immunoglobulin superfamily member 5
IPI01022265.1	MAPT	X	X		cytoskeleton		protein binding	Microtubule-associated protein
IPI00176104.1	SLITRK2			X	membrane	development,cell organization and biogenesis,cell differentiation	protein binding	Isoform 1 of SLIT and NTRK-like protein 2
IPI00020747.1	SCN3B		X	X	membrane,cytoplasm	development,cell organization and biogenesis,transport,regulation of biological process,response to stimulus,cell communication,cellular homeostasis,cell differentiation	protein binding,transporter activity	Sodium channel subunit beta-3
IPI00790503.3	MYH10	X	X		cytoskeleton,mitochondrion,membrane,cytoplasm	cell organization and biogenesis,metabolic process,transport,regulation of biological process	protein binding,transporter activity,motor activity,nucleotide binding,catalytic activity	Isoform 3 of Myosin-10
IPI00655702.3	NFASC			X			protein binding	Isoform 5 of Neurofascin
IPI00019884.1	ACTN2	X	X		extracellular,cytoskeleton,membrane,cytoplasm,organelle lumen,cytosol	cell death,cell organization and biogenesis,transport,regulation of biological process,response to stimulus,cellular component movement,cell communication,coagulation	protein binding,metal ion binding,structural molecule activity	Alpha-actinin-2
IPI00783559.1	EXOC2		X		membrane,cytoplasm	transport	protein binding	Exocyst complex component 2
IPI00099838.6	ACP6			X	extracellular,mitochondrion,cytoplasm	metabolic process	catalytic activity	Isoform 1 of Lysophosphatidic acid phosphatase type 6
IPI00646748.1	TPM2	X	X		membrane	transport,cell communication	transporter activity,structural molecule activity	Uncharacterized protein
IPI00009865.4	KRT10			X	cytoskeleton,cytoplasm	development,response to stimulus,cell differentiation	structural molecule activity	Keratin, type I cytoskeletal 10
IPI00032291.2	C5			X	extracellular,membrane	cell death,development,metabolic process,transport,regulation of biological process,response to stimulus,cellular component movement,cell communication,defense response	protein binding,enzyme regulator activity	Complement C5
IPI00220271.3	AKR1A1	X	X	X	membrane,cytoplasm,cytosol	metabolic process	catalytic activity	Alcohol dehydrogenase [NADP+]
IPI00219585.4	PFKM	X	X		cytoplasm,cytosol	metabolic process	catalytic activity	Isoform 2 of 6-phosphofructokinase, muscle type
IPI00025818.1	GALNT1			X	extracellular,membrane,cytoplasm,Golgi	metabolic process	metal ion binding,catalytic activity	Isoform 1 of Polypeptide N-acetylgalactosaminyltransferase 1
IPI00018314.1	SEC14L2	X	X		membrane,cytoplasm,cytosol,nucleus	transport,metabolic process,regulation of biological process	transporter activity,catalytic activity,enzyme regulator activity	SEC14-like protein 2
IPI00221178.1	TPD52L2	X	X	X		cell proliferation		Isoform 2 of Tumor protein D54
IPI00246058.10	PDCD6IP	X	X	X	cytoskeleton,cytoplasm,cytosol	cell death,cell division,transport	protein binding	Programmed cell death 6-interacting protein
IPI00305438.3	VPS16	X	X		cytoskeleton,membrane,cytoplasm,vacuole,endosome	transport	protein binding	Isoform 1 of Vacuolar protein sorting-associated protein 16 homolog
IPI00007797.3	FABP5	X	X	X	cytoplasm	development,transport,metabolic process	protein binding,transporter activity	Fatty acid-binding protein, epidermal
IPI00027733.1	GABARAPL1	X			cytoskeleton,membrane,endoplasmic reticulum,cytoplasm,Golgi,vacuole	cell organization and biogenesis,metabolic process,response to stimulus,cell communication	protein binding	Gamma-aminobutyric acid receptor-associated protein-like 1
IPI00025846.3	DSC2			X	membrane		metal ion binding	Isoform 2A of Desmocollin-2
IPI00294398.2	HADH	X	X		membrane,mitochondrion,cytoplasm,organelle lumen,nucleus	transport,metabolic process,regulation of biological process,response to stimulus,cell communication	nucleotide binding,catalytic activity	Isoform 1 of Hydroxyacyl-coenzyme A dehydrogenase, mitochondrial
IPI00303280.4	MPP6	X	X		membrane	cell organization and biogenesis	protein binding	MAGUK p55 subfamily member 6
IPI00017342.1	RHOG	X	X		membrane,cytoplasm,cytosol,nucleus	cell proliferation,development,cell organization and biogenesis,metabolic process,regulation of biological process,response to stimulus,cellular component movement,cell communication,cell differentiation,coagulation	protein binding,nucleotide binding,catalytic activity	Rho-related GTP-binding protein RhoG
IPI00333619.4	ALDH3A2	X	X		membrane,endoplasmic reticulum,cytoplasm	development,metabolic process	catalytic activity	Isoform 1 of Fatty aldehyde dehydrogenase
IPI00020539.1	PGPEP1	X			cytoplasm,cytosol	metabolic process	catalytic activity	Pyroglutamyl-peptidase 1
IPI00384395.2	IGHV6-1			X				Myosin-reactive immunoglobulin heavy chain variable region
IPI00152344.1	PHOSPHO2	X		X		metabolic process	metal ion binding,catalytic activity	Pyridoxal phosphate phosphatase PHOSPHO2

IPI	GENE	Alzheimer's Hippocampus	Control hippocampus	CSF	Cellular localization	Biological process	Molecular function	Protein Description
IPI00003370.1	STX1A	X	X		extracellular,cytoskeleton,membrane,cytoplasm	transport,metabolic process,regulation of biological process,response to stimulus,cell communication	protein binding	Isoform 1 of Syntaxin-1A
IPI00295741.4	CTSB	X	X	X	extracellular,cell surface,membrane,mitochondrion,cytoplasm,vacuole,organelle lumen,endosome	cell death,development,metabolic process,regulation of biological process,response to stimulus,defense response	protein binding,catalytic activity	Cathepsin B
IPI00030319.1	FOXF2			X	organelle lumen,nucleus	cell organization and biogenesis,development,metabolic process,regulation of biological process,cell differentiation,reproduction	protein binding,DNA binding	Forkhead box protein F2
IPI00012283.2	SEMA3B			X	extracellular,membrane,endoplasmic reticulum,cytoplasm	development,cell organization and biogenesis,response to stimulus,cell communication,cell differentiation	protein binding,receptor activity	Isoform 1 of Semaphorin-3B
IPI00064667.5	CNDP1	X		X	extracellular	metabolic process	metal ion binding,catalytic activity	Beta-Ala-His dipeptidase
IPI00911076.1	DCC			X			protein binding,receptor activity	cDNA FLJ51404, highly similar to Netrin receptor DCC
IPI00021733.1	NDST4			X	membrane,cytoplasm,Golgi	metabolic process	catalytic activity	Bifunctional heparan sulfate N-deacetylase/N-sulfotransferase 4
IPI00219065.2	AGL	X	X			metabolic process	catalytic activity	Isoform 5 of Glycogen debranching enzyme
IPI00221006.1	TCF7L2			X			protein binding	Isoform 4 of Transcription factor 7-like 2
IPI00791343.3	MEGF8			X	membrane			Uncharacterized protein
IPI00217831.4	ANKRD13A			X			protein binding	Ankyrin repeat domain-containing protein 13A
IPI00291419.6	ACAT2	X	X		membrane,mitochondrion,cytoplasm,organelle lumen,nucleus	metabolic process	catalytic activity	cDNA FLJ53975, highly similar to Acetyl-CoA acetyltransferase, cytosolic
IPI00022418.1	FN1			X	extracellular	metabolic process	protein binding,catalytic activity	Isoform 1 of Fibronectin
IPI00935200.1	LOC100293144			X				hypothetical protein XP_002343308
IPI00385143.1	PTF1A			X				Microfibrillar protein 2 (Fragment)
IPI00470716.1	SCG5			X	cytoplasm	regulation of biological process,response to stimulus,cell communication		Isoform 2 of Neuroendocrine protein 7B2
IPI00025239.2	NDUFS2	X	X		membrane,mitochondrion,cytoplasm	transport,metabolic process,response to stimulus	protein binding,metal ion binding,nucleotide binding,catalytic activity	NADH dehydrogenase [ubiquinone] iron-sulfur protein 2, mitochondrial
IPI00002523.2	CCBL1	X	X		cytoplasm,organelle lumen,nucleus	metabolic process	catalytic activity	cDNA FLJ56468, highly similar to Kynurenine--oxoglutarate transaminase 1
IPI00022300.5	METTL7A	X	X		membrane,endoplasmic reticulum,cytoplasm	metabolic process	catalytic activity	Methyltransferase-like protein 7A
IPI00010490.1	ARVCF	X			membrane,cytoplasm,nucleus	development	protein binding	Isoform Long of Armadillo repeat protein deleted in velo-cardio-facial syndrome
IPI00024466.2	UGGT1	X	X	X	endoplasmic reticulum,cytoplasm,organelle lumen	metabolic process	protein binding,catalytic activity	Isoform 1 of UDP-glucose:glycoprotein glucosyltransferase 1
IPI00026530.4	LMAN1			X	membrane,endoplasmic reticulum,cytoplasm,Golgi	cell organization and biogenesis,metabolic process,transport,regulation of biological process,response to stimulus,coagulation	protein binding,metal ion binding	Protein ERGIC-53
IPI00217519.3	RALA	X	X		cell surface,membrane,cytoplasm	cell organization and biogenesis,cell division,transport,metabolic process,regulation of biological process,response to stimulus,cell communication	protein binding,nucleotide binding,catalytic activity	Ras-related protein Ral-A
IPI00175989.7	MCF2L2			X		regulation of biological process,response to stimulus,cell communication	protein binding,enzyme regulator activity	Isoform 1 of Probable guanine nucleotide exchange factor MCF2L2
IPI00301204.2	RDH13		X		mitochondrion,cytoplasm	metabolic process	nucleotide binding,catalytic activity	Isoform 1 of Retinol dehydrogenase 13
IPI00872684.2	EZR			X	cytoskeleton,membrane,cytoplasm		protein binding	cDNA FLJ54141, highly similar to Ezrin
IPI00374301.5	LOC100128918			X				hypothetical protein
IPI00001586.1	ENTPD2	X			extracellular,membrane	metabolic process,regulation of biological process,response to stimulus,cell communication,coagulation	nucleotide binding,catalytic activity	Isoform Long of Ectonucleoside triphosphate diphosphohydrolase 2
IPI00328319.8	RBBP4	X	X		organelle lumen,chromosome,nucleus	cell proliferation,cell organization and biogenesis,metabolic process,regulation of biological process	protein binding,catalytic activity	Isoform 1 of Histone-binding protein RBBP4
IPI00399272.1	PDE1A	X	X			regulation of biological process,response to stimulus,cell communication	catalytic activity	Isoform 6 of Calcium/calmodulin-dependent 3',5'-cyclic nucleotide phosphodiesterase 1A
IPI00048230.1	NXPH1			X	extracellular		protein binding	Neurexophilin-1

IPI	GENE	Alzheimer's Hippocampus	Control hippocampus	CSF	Cellular localization	Biological process	Molecular function	Protein Description
IPI00301579.4	NPC2	X	X	X	membrane			cDNA FLJ59142, highly similar to Epididymal secretory protein E1
IPI00010105.1	EIF6	X	X		cytoskeleton,cytoplasm,organelle lumen,nucleus	cell organization and biogenesis,metabolic process	protein binding,RNA binding	Eukaryotic translation initiation factor 6
IPI00442564.1	LOC645342			X			catalytic activity	cDNA FLJ26948 fis, clone RCT08241
IPI00027487.3	CKM			X	cytoplasm,cytosol	metabolic process	nucleotide binding,catalytic activity	Creatine kinase M-type
IPI00454858.6	COL6A4P1			X			protein binding	Dual Intracellular Von Willebrand factor domain A
IPI00008554.1	ANG			X	extracellular,organelle lumen,nucleus	cell death,cell proliferation,cell organization and biogenesis,development,transport,metabolic process,regulation of biological process,response to stimulus,cellular component movement,cell communication,reproduction,cell differentiation	protein binding,DNA binding,RNA binding,metal ion binding,catalytic activity	Angiogenin
IPI00031397.3	ACSL3	X	X		membrane,mitochondrion,endoplasmic reticulum,cytoplasm,Golgi	development,transport,metabolic process,regulation of biological process,response to stimulus	protein binding,nucleotide binding,catalytic activity	Long-chain-fatty-acid--CoA ligase 3
IPI00328522.5	POGLUT1			X	extracellular,cytoplasm			KTEL (Lys-Tyr-Glu-Leu) containing 1
IPI00790473.1	SERPINF1			X			enzyme regulator activity	Pigment epithelium-derived factor
IPI00003588.1	EEF1E1		X		cytoplasm,organelle lumen,nucleus,cytosol	cell death,cell proliferation,metabolic process,regulation of biological process,response to stimulus,cell communication		Eukaryotic translation elongation factor 1 epsilon-1
IPI00024913.2	C21orf33	X	X		mitochondrion,cytoplasm			Isoform Long of ES1 protein homolog, mitochondrial
IPI00059264.3	VPS26B	X	X		membrane,cytoplasm,cytosol	transport		Vacuolar protein sorting-associated protein 26B
IPI00027350.3	PRDX2	X	X	X	mitochondrion,cytoplasm	cell death,cell proliferation,development,metabolic process,regulation of biological process,response to stimulus,cell communication,defense response,cell differentiation,coagulation	antioxidant activity,catalytic activity	Peroxiredoxin-2
IPI00296863.3	DCTD		X		cytoplasm,cytosol	metabolic process	metal ion binding,catalytic activity	Isoform 1 of Deoxycytidylate deaminase
IPI00297188.6	BAI2			X	membrane	regulation of biological process,response to stimulus,cell communication	signal transducer activity,receptor activity	Isoform 3 of Brain-specific angiogenesis inhibitor 2
IPI00553043.3	LIMCH1	X	X			cell organization and biogenesis	protein binding,metal ion binding	Isoform 1 of LIM and calponin homology domains-containing protein 1
IPI00013789.5	SMYD5		X				protein binding,metal ion binding	SET and MYND domain-containing protein 5
IPI00022055.2	KAT2B			X	cytoskeleton,cytoplasm,organelle lumen,chromosome,nucleus	cell proliferation,cell organization and biogenesis,metabolic process,regulation of biological process,response to stimulus,cell communication	protein binding,catalytic activity,enzyme regulator activity	Histone acetyltransferase KAT2B
IPI00021812.2	AHNAK	X	X	X	nucleus	development	protein binding	Neuroblast differentiation-associated protein AHNAK
IPI00016925.1	C12orf57		X					Protein C10
IPI00644191.1	ZNF90			X		metabolic process,regulation of biological process	metal ion binding	70 kDa protein
IPI00021174.1	GGA3		X		membrane,cytoplasm,Golgi,endosome	transport	protein binding	Isoform Long of ADP-ribosylation factor-binding protein GGA3
IPI00306960.3	NARS	X	X		mitochondrion,cytoplasm,cytosol	metabolic process	nucleotide binding,catalytic activity	Asparaginyl-tRNA synthetase, cytoplasmic
IPI00171411.4	GOLM1			X				Golgi membrane protein 1
IPI00022606.2	PSTPIP1			X	cytoskeleton,cytoplasm,cytosol	metabolic process,regulation of biological process,response to stimulus,cell communication,defense response	protein binding,catalytic activity	Isoform 1 of Proline-serine-threonine phosphatase-interacting protein 1
IPI00738920.3	LOC642574			X			protein binding	Putative IQ motif and ankyrin repeat domain-containing protein LOC642574
IPI00789954.1	TF			X	extracellular	transport,cellular homeostasis	metal ion binding	7 kDa protein
IPI00789337.4	YWHAZ		X		cytoskeleton,mitochondrion,cytoplasm,nucleus	cell organization and biogenesis,transport,response to stimulus,cell communication,defense response	protein binding	cDNA, FLJ79516, highly similar to 14-3-3 protein zeta/delta
IPI00155466.7	SUCLG2	X				metabolic process	nucleotide binding,catalytic activity	Uncharacterized protein
IPI00292150.5	LTBP2			X	extracellular,cell surface,membrane	transport,regulation of biological process,response to stimulus,cell communication	protein binding,metal ion binding	Latent-transforming growth factor beta-binding protein 2
IPI00784257.1	FOLR2			X	cytoskeleton,membrane,cytoplasm,vacuole,cytosol	development,cell organization and biogenesis,transport,response to stimulus,cellular component movement,cell differentiation	protein binding,signal transducer activity,nucleotide binding,motor activity,catalytic activity	Folate receptor beta precursor

IPI	GENE	Alzheimer's Hippocampus	Control hippocampus	CSF	Cellular localization	Biological process	Molecular function	Protein Description
IPI00409671.3	DDX42		X		cytoplasm,organelle lumen,nucleus	regulation of biological process	protein binding,RNA binding,nucleotide binding,catalytic activity	Isoform 1 of ATP-dependent RNA helicase DDX42
IPI00019269.3	WDR61	X	X		cytoplasm,organelle lumen,chromosome,nucleus	cell organization and biogenesis,development,metabolic process,regulation of biological process,response to stimulus,cell communication,cell differentiation	protein binding	WD repeat-containing protein 61
IPI00219664.4	MOG			X				Isoform 2 of Myelin-oligodendrocyte glycoprotein
IPI00741710.4	SNED1			X			protein binding,metal ion binding	Isoform 3 of Sushi, nidogen and EGF-like domain-containing protein 1
IPI00010118.7	PTOV1			X	membrane,cytoplasm,nucleus	metabolic process,regulation of biological process		Isoform 1 of Prostate tumor-overexpressed gene 1 protein
IPI00375577.2	TMEM65	X	X		membrane,mitochondrion,cytoplasm			Transmembrane protein 65
IPI00796279.1	SERPINF1			X			enzyme regulator activity	25 kDa protein
IPI00916111.3	MDH1	X	X	X	cytoskeleton,cytoplasm,cytosol	metabolic process	nucleotide binding,catalytic activity	Malate dehydrogenase, cytoplasmic
IPI00953028.1	RPL7P32	X	X		cytoplasm,ribosome	metabolic process	structural molecule activity	similar to 60S ribosomal protein L7; similar to P18124 (PID:d133021)
IPI00178767.1	SMPDL3A			X	extracellular	metabolic process	protein binding,catalytic activity	Acid sphingomyelinase-like phosphodiesterase 3a
IPI00299059.6	CHL1			X			protein binding	cell adhesion molecule with homology to L1CAM (close homolog of L1), isoform CRA_b
IPI00645702.1	CTPS2	X	X		mitochondrion,cytoplasm,cytosol	metabolic process	nucleotide binding,catalytic activity	CTP synthase 2
IPI00033143.1	EIF3K	X	X		cytoplasm,organelle lumen,nucleus,cytosol	metabolic process,regulation of biological process	RNA binding	Eukaryotic translation initiation factor 3 subunit K
IPI00001786.5	USP36			X		metabolic process	catalytic activity	Isoform 2 of Ubiquitin carboxyl-terminal hydrolase 36
IPI00013122.1	CDC37	X	X		cytoplasm,cytosol	metabolic process,transport,regulation of biological process,response to stimulus,cell communication,defense response	protein binding	Hsp90 co-chaperone Cdc37
IPI00023648.6	ISLR			X	extracellular		protein binding	Immunoglobulin superfamily containing leucine-rich repeat protein
IPI00184019.6	PILRA			X				Isoform 3 of Paired immunoglobulin-like type 2 receptor alpha
IPI00304761.12	DDHD2		X		cytoskeleton,cytoplasm		protein binding,metal ion binding	cDNA FLJ45096 fis, clone BRAWH3030910, highly similar to Homo sapiens DDHD domain containing 2 (DDHD2), mRNA
IPI00410122.2	PLXDC1			X	extracellular,membrane,cytoplasm	development	protein binding	Isoform 1 of Plexin domain-containing protein 1
IPI00006510.1	TUBB1			X	cytoskeleton,cytoplasm	cell organization and biogenesis,metabolic process,cellular component movement	nucleotide binding,structural molecule activity,catalytic activity	Tubulin beta-1 chain
IPI00099986.5	FN3KRP	X	X			metabolic process	catalytic activity	Ketosamine-3-kinase
IPI00293655.3	DDX1	X	X		cytoplasm,organelle lumen,nucleus	cell organization and biogenesis,development,metabolic process,regulation of biological process,response to stimulus	protein binding,DNA binding,RNA binding,nucleotide binding,catalytic activity	ATP-dependent RNA helicase DDX1
IPI00014340.2	PPP1R12C			X	cytoplasm		protein binding	Isoform 1 of Protein phosphatase 1 regulatory subunit 12C
IPI00644210.3	GLYR1		X			metabolic process	catalytic activity	Isoform 4 of Putative oxidoreductase GLYR1
IPI00008580.1	SLPI			X	extracellular	metabolic process,regulation of biological process,reproduction	protein binding,enzyme regulator activity	Antileukoproteinase
IPI00018219.1	TGFBI			X	extracellular,membrane	cell proliferation,cell organization and biogenesis,development,regulation of biological process,response to stimulus,cell differentiation	protein binding	Transforming growth factor-beta-induced protein ig-h3
IPI00304803.3	GMPR	X	X		cytoplasm,cytosol	metabolic process,response to stimulus	metal ion binding,catalytic activity	GMP reductase 1
IPI00215899.1	SRPX			X				Isoform 2 of Sushi repeat-containing protein SRPX
IPI00299003.2	WASF3	X	X		cytoskeleton,cytoplasm	cell organization and biogenesis,regulation of biological process	protein binding	Wiskott-Aldrich syndrome protein family member 3
IPI00018275.2	PRND			X	membrane	cell organization and biogenesis,cellular homeostasis	metal ion binding	Prion-like protein doppel
IPI00021903.1	ADAM23	X	X	X	extracellular,membrane	development,metabolic process	protein binding,metal ion binding,catalytic activity	Isoform Alpha of Disintegrin and metalloproteinase domain-containing protein 23
IPI00009946.4	TOMM34		X		membrane,mitochondrion,cytoplasm	cell organization and biogenesis,transport	protein binding	Mitochondrial import receptor subunit TOM34

IPI	GENE	Alzheimer's Hippocampus	Control hippocampus	CSF	Cellular localization	Biological process	Molecular function	Protein Description
IPI00005158.1	LONP1	X	X	X	mitochondrion,cytoplasm,organelle lumen	cell organization and biogenesis,metabolic process,response to stimulus	protein binding,DNA binding,RNA binding,nucleotide binding,catalytic activity	Lon protease homolog, mitochondrial
IPI00411486.1	OPALIN	X	X		membrane,cytoplasm,Golgi			Opalin
IPI00395783.4	LTBP4			X			protein binding,metal ion binding	Isoform 4 of Latent-transforming growth factor beta-binding protein 4
IPI00005774.6	LRP8			X	extracellular,cell surface,membrane	cell organization and biogenesis,development,transport,metabolic process,regulation of biological process,response to stimulus,cellular component movement,cell communication,cell differentiation,coagulation	protein binding,signal transducer activity,metal ion binding,receptor activity	Isoform 1 of Low-density lipoprotein receptor-related protein 8
IPI00171206.6	NECAB1		X		cytoplasm,organelle lumen,nucleus		metal ion binding	Isoform 1 of N-terminal EF-hand calcium-binding protein 1
IPI00329104.7	LILRA3			X	extracellular,membrane	regulation of biological process,response to stimulus,cell communication,defense response	protein binding,receptor activity	Leukocyte immunoglobulin-like receptor subfamily A member 3
IPI00017551.2	RGN	X	X		cytoplasm,nucleus	metabolic process,regulation of biological process,response to stimulus,cell communication,cellular homeostasis	metal ion binding,catalytic activity,enzyme regulator activity	Isoform 1 of Regucalcin
IPI00296141.4	DPP7	X	X	X	extracellular,cytoplasm,vacuole,cytosol	metabolic process	catalytic activity	Dipeptidyl peptidase 2
IPI00909984.3	ANK3	X	X		membrane		protein binding	Uncharacterized protein
IPI00382894.2	TPM3	X	X		cytoskeleton,cytoplasm			Uncharacterized protein
IPI00643583.2	DYNLRB1	X	X		cytoskeleton,cytoplasm	transport,response to stimulus,cellular component movement	protein binding,motor activity,catalytic activity	Isoform 1 of Dynein light chain roadblock-type 1
IPI00011605.1	CBLN1			X	extracellular,membrane	development,cell organization and biogenesis,transport,regulation of biological process,cell communication	protein binding	Cerebellin-1
IPI00156282.2	GPS1	X	X		cytoplasm,nucleus	metabolic process,regulation of biological process,response to stimulus,cell communication	protein binding,enzyme regulator activity	Isoform 1 of COP9 signalosome complex subunit 1
IPI00003935.6	HIST2H2BE	X	X	X	chromosome,nucleus	cell organization and biogenesis,metabolic process,response to stimulus,defense response	DNA binding	Histone H2B type 2-E
IPI00455521.3	SHISA8			X	membrane			Putative protein shisa-8
IPI00465170.1	NT5DC3		X		mitochondrion,cytoplasm,cytosol	metabolic process	metal ion binding,catalytic activity	Isoform 1 of 5'-nucleotidase domain-containing protein 3
IPI00028457.2	RGS7		X		membrane,cytoplasm	metabolic process,regulation of biological process,response to stimulus,cell communication	signal transducer activity,enzyme regulator activity	Isoform 1 of Regulator of G-protein signaling 7
IPI00005908.2	ADAMTS1			X	extracellular,cytoplasm	cell proliferation,development,metabolic process,regulation of biological process,response to stimulus,cell communication,reproduction	metal ion binding,catalytic activity	A disintegrin and metalloproteinase with thrombospondin motifs 1
IPI00745568.2	TIPRL	X	X		cytoplasm	metabolic process,regulation of biological process,response to stimulus	protein binding	Isoform 1 of TIP41-like protein
IPI00023807.3	SEMA4D			X	extracellular,membrane	cell death,development,cell organization and biogenesis,metabolic process,regulation of biological process,response to stimulus,cellular component movement,cell communication,cell differentiation,cell growth	protein binding,signal transducer activity,receptor activity	Isoform 1 of Semaphorin-4D
IPI00024853.3	PRX			X	membrane,cytoplasm,nucleus	development,metabolic process,cell communication,cellular homeostasis	protein binding	Isoform 1 of Periaxin
IPI00025849.1	ANP32A	X	X		endoplasmic reticulum,cytoplasm,organelle lumen,nucleus	transport,metabolic process,regulation of biological process,response to stimulus,cell communication	protein binding	Acidic leucine-rich nuclear phosphoprotein 32 family member A
IPI00186621.3	OFCC1			X				Isoform 2 of Orofacial cleft 1 candidate gene 1 protein
IPI00022758.3	GAN	X			cytoskeleton,cytoplasm	cell death,metabolic process	protein binding	Gigaxonin
IPI00167619.2	LRTM2			X	membrane		protein binding	Leucine-rich repeat and transmembrane domain-containing protein 2
IPI00025992.1	HAMP			X	extracellular,cytoplasm	response to stimulus,defense response,cellular homeostasis	protein binding	Hepcidin
IPI00432707.5	CASP12			X		cell death,metabolic process,regulation of biological process	protein binding,catalytic activity	Isoform 1 of Inactive caspase-12
IPI00874020.2	PCBD2	X	X		mitochondrion,cytoplasm,nucleus	cell organization and biogenesis,metabolic process,regulation of biological process	protein binding,catalytic activity	Pterin-4-alpha-carbinolamine dehydratase 2
IPI00221091.9	RPS15A	X	X		mitochondrion,cytoplasm,ribosome,cytosol	cell proliferation,cell organization and biogenesis,metabolic process,transport,regulation of biological process,response to stimulus,reproduction	RNA binding,structural molecule activity	40S ribosomal protein S15a

IPI	GENE	Alzheimer's Hippocampus	Control hippocampus	CSF	Cellular localization	Biological process	Molecular function	Protein Description
IPI00015881.2	CSF1			X	extracellular,membrane,cytoplasm	cell proliferation,development,metabolic process,regulation of biological process,response to stimulus,cellular component movement,cell communication,defense response,cell differentiation,reproduction	protein binding	Isoform 1 of Macrophage colony-stimulating factor 1
IPI00873344.1	TPD52			X				Isoform 3 of Tumor protein D52
IPI00014048.1	RNASE1			X	extracellular	metabolic process	protein binding,catalytic activity	Ribonuclease pancreatic
IPI00165261.6	SCFD1	X	X		membrane,endoplasmic reticulum,cytoplasm,Golgi	transport,regulation of biological process,response to stimulus	protein binding	Sec1 family domain-containing protein 1
IPI00552735.3	IGLV3-12			X				V2-8 protein
IPI00063827.1	ABHD14B	X	X	X	cytoplasm,organelle lumen,nucleus		catalytic activity	Isoform 1 of Abhydrolase domain-containing protein 14B
IPI00465256.4	AK3	X	X		mitochondrion,cytoplasm,organelle lumen	metabolic process,response to stimulus,coagulation	protein binding,nucleotide binding,catalytic activity	GTP:AMP phosphotransferase, mitochondrial
IPI00166010.6	CNOT1			X	membrane,cytoplasm,cytosol	metabolic process,regulation of biological process	protein binding	Isoform 1 of CCR4-NOT transcription complex subunit 1
IPI00141938.4	H2AFV			X	chromosome,nucleus	cell organization and biogenesis,metabolic process	DNA binding	histone H2A.V isoform 2
IPI00006121.1	IDS		X			metabolic process	catalytic activity	Isoform 2 of Iduronate 2-sulfatase
IPI00024539.1	PRKCE	X	X		cytoskeleton,mitochondrion,membrane,endoplasmic reticulum,cytoplasm,Golgi,cytosol,nucleus	cell death,cell organization and biogenesis,metabolic process,cell division,transport,regulation of biological process,response to stimulus,cellular component movement,defense response,cell communication,cellular homeostasis,coagulation	signal transducer activity,protein binding,metal ion binding,nucleotide binding,catalytic activity,enzyme regulator activity	Protein kinase C epsilon type
IPI00413578.4	TPRG1L	X	X		cytoplasm		protein binding	Isoform 1 of Tumor protein p63-regulated gene 1-like protein
IPI00022082.7	Sep-08	X	X				nucleotide binding	Isoform 2 of Septin-8
IPI00022649.3	SLC12A2	X	X	X	membrane	development,transport,response to stimulus	transporter activity	Isoform 1 of Solute carrier family 12 member 2
IPI00796392.3	RASAL1		X			metabolic process,regulation of biological process,response to stimulus,cell communication	protein binding,enzyme regulator activity	RASAL1 protein
IPI00044842.1	RAB3IP			X	cytoskeleton,cytoplasm,nucleus,cytosol	cell organization and biogenesis,transport	protein binding,motor activity,catalytic activity,enzyme regulator activity	Isoform 2 of Rab-3A-interacting protein
IPI00852954.1	KIAA1045	X	X				metal ion binding	Protein KIAA1045
IPI00386687.3	LRRFIP1		X				nucleotide binding	leucine-rich repeat flightless-interacting protein 1 isoform 1
IPI00010400.1	PLCB3	X	X		cytoskeleton,membrane,cytoplasm,cytosol	transport,metabolic process,regulation of biological process,response to stimulus,cell communication	protein binding,signal transducer activity,metal ion binding,motor activity,catalytic activity	1-phosphatidylinositol-4,5-bisphosphate phosphodiesterase beta-3
IPI00186008.2	STARD10	X	X					PCTP-like protein
IPI00171833.2	CCDC104		X					Isoform 1 of Coiled-coil domain-containing protein 104
IPI00830132.1	IGHG4			X			protein binding	Putative uncharacterized protein IGHG4 (Fragment)
IPI00220249.6	LTBP1			X	cell surface,membrane		protein binding,metal ion binding	Uncharacterized protein
IPI00027166.1	TIMP2			X	extracellular,cell surface	cell proliferation,development,cell organization and biogenesis,metabolic process,regulation of biological process,response to stimulus,cell communication,cell differentiation	protein binding,metal ion binding,enzyme regulator activity	Metalloproteinase inhibitor 2
IPI00926625.1	ZYX	X			cytoskeleton,membrane,cytoplasm,nucleus	regulation of biological process,response to stimulus,cell communication	protein binding,metal ion binding	Zyxin
IPI00025880.2	MYH7			X	cytoskeleton,membrane,cytoplasm,organelle lumen,chromosome,nucleus	development,cell organization and biogenesis,metabolic process,regulation of biological process,response to stimulus,cellular component movement,reproduction	protein binding,nucleotide binding,motor activity,structural molecule activity,catalytic activity	Myosin-7
IPI00186004.9	ANKRD36B			X			protein binding	Isoform 3 of Ankyrin repeat domain-containing protein 36B
IPI00168514.2	MORN4		X					Isoform 1 of MORN repeat-containing protein 4
IPI00151462.3	MAP1LC3B2	X	X		cytoskeleton,membrane,cytoplasm,vacuole	cell organization and biogenesis,metabolic process,response to stimulus,cell communication		Microtubule-associated proteins 1A/1B light chain 3 beta 2
IPI00025464.1	MT1F	X	X		cytoplasm,nucleus	regulation of biological process,response to stimulus	metal ion binding	Metallothionein-1F

IPI	GENE	Alzheimer's Hippocampus	Control hippocampus	CSF	Cellular localization	Biological process	Molecular function	Protein Description
IPI00025084.3	CAPNS1	X	X	X	membrane,cytoplasm	cell proliferation,metabolic process,regulation of biological process	protein binding,metal ion binding,catalytic activity	Calpain small subunit 1
IPI00022021.3	PHYHIP	X	X				protein binding	Phytanoyl-CoA hydroxylase-interacting protein
IPI00178926.2	IGJ			X	extracellular	response to stimulus		Immunoglobulin J chain
IPI00005537.3	SLC25A10	X	X		membrane,mitochondrion,cytoplasm,ribosome,organelle lumen,nucleus	transport,metabolic process	structural molecule activity	cDNA FLJ60124, highly similar to Mitochondrial dicarboxylate carrier
IPI00027497.5	GPI	X	X	X	extracellular,membrane,cytoplasm,organelle lumen,nucleus,cytosol	development,metabolic process,response to stimulus	protein binding,catalytic activity	Glucose-6-phosphate isomerase
IPI00218728.4	PAFAH1B1	X	X		cytoskeleton,membrane,cytoplasm,organelle lumen,chromosome,cytosol,nucleus	cell organization and biogenesis,development,metabolic process,regulation of biological process,response to stimulus,reproduction,cell proliferation,cell division,transport,cellular component movement,cell communication,cell differentiation,cell growth	protein binding	Isoform 1 of Platelet-activating factor acetylhydrolase IB subunit alpha
IPI00409607.4	CAMSAP2	X	X		cytoskeleton,cytoplasm		protein binding	Isoform 1 of Calmodulin-regulated spectrin-associated protein 2
IPI00032227.2	RPH3A	X	X	X	membrane,cytoplasm	transport	protein binding,transporter activity,metal ion binding	Isoform 1 of Rabphilin-3A
IPI00396378.3	HNRNPA2B1	X	X	X	spliceosomal complex,cytoplasm,organelle lumen,nucleus	transport,metabolic process	protein binding,DNA binding,RNA binding,nucleotide binding	Isoform B1 of Heterogeneous nuclear ribonucleoproteins A2/B1
IPI00028908.4	NID2	X		X	extracellular,cell surface,membrane		protein binding,metal ion binding	Isoform 1 of Nidogen-2
IPI00025100.1	BCKDHA	X	X		mitochondrion,cytoplasm,organelle lumen	metabolic process,response to stimulus	protein binding,metal ion binding,catalytic activity	2-oxoisovalerate dehydrogenase subunit alpha, mitochondrial
IPI00021891.5	FGG	X	X	X	extracellular,cell surface,membrane,cytoplasm,organelle lumen	cell organization and biogenesis,transport,regulation of biological process,response to stimulus,cell communication,coagulation	protein binding,catalytic activity	Isoform Gamma-B of Fibrinogen gamma chain
IPI00008475.1	HMGCS1		X		cytoplasm,cytosol	development,metabolic process,response to stimulus,reproduction	protein binding,catalytic activity	Hydroxymethylglutaryl-CoA synthase, cytoplasmic
IPI00299399.7	S100B	X	X	X	extracellular,cytoplasm,nucleus	cell proliferation,development,cell organization and biogenesis,regulation of biological process,response to stimulus,defense response,cell communication,cell differentiation	protein binding,metal ion binding	Protein S100-B
IPI00183508.3	TWF1	X	X				protein binding	44 kDa protein
IPI00220007.6	APOL2	X	X		extracellular	transport,metabolic process	catalytic activity	50 kDa protein
IPI00024971.1	OSBP	X	X		membrane,cytoplasm,Golgi	transport	protein binding	Oxysterol-binding protein 1
IPI00184119.6	DNAJC6	X	X				protein binding	Isoform 2 of Putative tyrosine-protein phosphatase auxilin
IPI00743623.2	AK5	X				metabolic process	nucleotide binding,catalytic activity	Adenylate kinase isoenzyme 5
IPI00478003.3	A2M	X	X	X	extracellular,cytoplasm,organelle lumen,cytosol	metabolic process,transport,regulation of biological process,response to stimulus,defense response,cell communication,cell differentiation,coagulation	protein binding,enzyme regulator activity	Alpha-2-macroglobulin
IPI00478892.2	LRIG2			X	membrane,cytoplasm		protein binding	Leucine-rich repeats and immunoglobulin-like domains protein 2
IPI00017557.8	SFRP4			X	extracellular,cell surface,membrane,cytoplasm,nucleus	cell death,cell proliferation,development,cell organization and biogenesis,metabolic process,transport,regulation of biological process,response to stimulus,cell communication,reproduction,cell differentiation	protein binding,signal transducer activity,receptor activity	Secreted frizzled-related protein 4
IPI00103925.2	IRGQ	X	X					Immunity-related GTPase family Q protein
IPI00376427.4	NCAM2	X	X	X	membrane		protein binding	Neural cell adhesion molecule 2
IPI00296922.4	LAMB2	X	X	X	extracellular,cytoskeleton,membrane,cytoplasm,nucleus	cell organization and biogenesis,development,metabolic process,transport,response to stimulus,cell growth,cell differentiation	protein binding,transporter activity,motor activity,nucleotide binding,structural molecule activity,catalytic activity	Laminin subunit beta-2
IPI00335541.5	TIMELESS			X	organelle lumen,chromosome,nucleus	cell proliferation,development,cell organization and biogenesis,cell division,metabolic process,regulation of biological process,response to stimulus	protein binding	Isoform 1 of Protein timeless homolog
IPI00015047.1	CD320			X				8D6 antigen (Fragment)
IPI00013683.2	TUBB3	X	X		cytoskeleton,cytoplasm	development,cell organization and biogenesis,metabolic process,response to stimulus,cellular component movement,cell differentiation	nucleotide binding,structural molecule activity,catalytic activity	Tubulin beta-3 chain

IPI	GENE	Alzheimer's Hippocampus	Control hippocampus	CSF	Cellular localization	Biological process	Molecular function	Protein Description
IPI00783378.3	UBE2O	X	X			metabolic process	nucleotide binding,catalytic activity	Ubiquitin-conjugating enzyme E2 O
IPI00794679.1	HLA-B			X	membrane	response to stimulus		Major histocompatibility complex, class I, B
IPI00306531.3	NCK2		X		membrane,endoplasmic reticulum,cytoplasm,cytosol	cell proliferation,cell organization and biogenesis,development,metabolic process,regulation of biological process,response to stimulus,cellular component movement,cell communication,cell differentiation	protein binding,structural molecule activity	Cytoplasmic protein NCK2
IPI00169307.6	ARHGAP21		X			regulation of biological process,response to stimulus,cell communication	protein binding	rho GTPase-activating protein 21
IPI00830025.1	IGHV4-28			X				13 kDa protein
IPI00219217.3	LDHB	X	X	X	mitochondrion,cytoplasm,cytosol	metabolic process	protein binding,nucleotide binding,catalytic activity	L-lactate dehydrogenase B chain
IPI00024915.3	PRDX5	X	X		mitochondrion,cytoplasm,organelle lumen,nucleus,cytosol	cell death,metabolic process,regulation of biological process,response to stimulus,defense response	antioxidant activity,protein binding,DNA binding,catalytic activity,enzyme regulator activity	Isoform Mitochondrial of Peroxiredoxin-5, mitochondrial
IPI00030023.3	HNMT	X	X		membrane,cytoplasm,organelle lumen,nucleus	development,metabolic process,response to stimulus	catalytic activity	Histamine N-methyltransferase
IPI00000792.1	CRYZ	X	X	X	cytoplasm,Golgi,cytosol	cell organization and biogenesis,metabolic process,response to stimulus	RNA binding,metal ion binding,nucleotide binding,catalytic activity	Quinone oxidoreductase
IPI00021856.3	APOC2			X	extracellular	cell organization and biogenesis,metabolic process,transport,regulation of biological process,response to stimulus	protein binding,enzyme regulator activity	Apolipoprotein C-II
IPI00024253.1	FGF14			X	nucleus	cell death,development,regulation of biological process,response to stimulus,cell communication	protein binding	Isoform 1 of Fibroblast growth factor 14
IPI00059279.5	EXOC4		X		cytoskeleton,membrane,cytoplasm,Golgi,endosome	development,transport,regulation of biological process,response to stimulus,cell communication,cell differentiation	protein binding	Exocyst complex component 4
IPI00180675.4	TUBA1A	X	X		cytoskeleton,cytoplasm,cytosol	cell organization and biogenesis,metabolic process,cell division,transport,cellular component movement	protein binding,nucleotide binding,structural molecule activity,catalytic activity	Tubulin alpha-1A chain
IPI00154774.4	OSCP1	X	X		membrane	transport		Isoform 1 of Protein OSCP1
IPI00604599.2	TMED3			X	membrane,endoplasmic reticulum,cytoplasm,Golgi	transport		Transmembrane emp24 domain-containing protein 3
IPI00377077.2	ASTN2			X				Isoform 3 of Astrotactin-2
IPI00017373.1	RPA3	X	X		cytoplasm,organelle lumen,chromosome,nucleus	cell organization and biogenesis,metabolic process,regulation of biological process,response to stimulus	protein binding,DNA binding	Replication protein A 14 kDa subunit
IPI00005721.1	DEFA1B			X	extracellular,cytoplasm,Golgi,organelle lumen,vacuole	response to stimulus,defense response		Neutrophil defensin 1
IPI00853454.1	LAMB1			X	extracellular,cytoskeleton,mitochondrion,membrane,cytoplasm,nucleus	development,transport,metabolic process,regulation of biological process,response to stimulus,cellular component movement,cell communication,cell differentiation	signal transducer activity,protein binding,nucleotide binding,motor activity,catalytic activity	200 kDa protein
IPI00028481.1	RAB8A	X	X	X	cytoskeleton,membrane,cytoplasm,Golgi,endosome	cell organization and biogenesis,transport,regulation of biological process,response to stimulus,cell communication	protein binding,nucleotide binding	Ras-related protein Rab-8A
IPI00006657.1	FAM20B			X	membrane,cytoplasm,Golgi	metabolic process	nucleotide binding,catalytic activity	Glycosaminoglycan xylosylkinase
IPI00001543.1	TIMM10	X			mitochondrion,membrane,cytoplasm	cell organization and biogenesis,metabolic process,transport	protein binding,transporter activity,metal ion binding	Mitochondrial import inner membrane translocase subunit Tim10
IPI00008756.8	DST	X	X	X	membrane	regulation of biological process	protein binding,metal ion binding	Uncharacterized protein
IPI00018276.1	SEZ6L2		X	X	membrane		signal transducer activity	Isoform 3 of Seizure 6-like protein 2
IPI00515041.5	CFH			X				Uncharacterized protein
IPI00012858.1	KCNQ2		X		membrane	cell organization and biogenesis,development,transport,regulation of biological process,response to stimulus,cell communication,cell differentiation	protein binding,transporter activity	Isoform 1 of Potassium voltage-gated channel subfamily KQT member 2
IPI00307794.2	AKAP5		X		membrane,cytoplasm,cytosol	transport,metabolic process,regulation of biological process,response to stimulus,cell communication	protein binding	A-kinase anchor protein 5
IPI00448925.6	IGHG1			X	membrane			44 kDa protein
IPI00012895.3	CA12			X	membrane	metabolic process	metal ion binding,catalytic activity	Isoform 1 of Carbonic anhydrase 12

IPI	GENE	Alzheimer's Hippocampus	Control hippocampus	CSF	Cellular localization	Biological process	Molecular function	Protein Description
IPI00221080.1	PTHLH			X	extracellular,cytoplasm,Golgi,organelle lumen,nucleus	cell proliferation,development,metabolic process,regulation of biological process,response to stimulus,cell communication,cell differentiation	protein binding	Isoform 2 of Parathyroid hormone-related protein
IPI00218087.1	SRGAP3	X	X		cytoplasm,cytosol	metabolic process,regulation of biological process,response to stimulus,cell communication	protein binding,enzyme regulator activity	Isoform 1 of SLIT-ROBO Rho GTPase-activating protein 3
IPI00021347.1	UBE2L3	X	X	X	cytoplasm,nucleus	cell proliferation,metabolic process,regulation of biological process,response to stimulus	protein binding,nucleotide binding,catalytic activity	Ubiquitin-conjugating enzyme E2 L3
IPI00019381.4	TMEM30A	X	X		membrane,endoplasmic reticulum,cytoplasm	transport,regulation of biological process	protein binding	Isoform 1 of Cell cycle control protein 50A
IPI00218658.1	ITPR1	X	X		membrane	transport	transporter activity	Isoform 2 of Inositol 1,4,5-trisphosphate receptor type 1
IPI00657936.2	COL28A1			X	extracellular	metabolic process,regulation of biological process	protein binding,enzyme regulator activity	Isoform 1 of Collagen alpha-1(XXVIII) chain
IPI00100656.3	TECR	X	X		membrane,endoplasmic reticulum,cytoplasm	metabolic process	catalytic activity	Isoform 1 of Trans-2,3-enoyl-CoA reductase
IPI00027482.1	SERPINA6			X	extracellular	transport,metabolic process,regulation of biological process	enzyme regulator activity	Corticosteroid-binding globulin
IPI00060265.4	ZNF775			X	nucleus	metabolic process,regulation of biological process	DNA binding,metal ion binding	Zinc finger protein 775
IPI00059762.5	LYPLAL1	X	X		cytoplasm		catalytic activity	Isoform 1 of Lysophospholipase-like protein 1
IPI00023014.3	VWF			X	extracellular,cell surface,membrane,endoplasmic reticulum,cytoplasm,organelle lumen	cell organization and biogenesis,development,metabolic process,transport,response to stimulus,coagulation	protein binding	von Willebrand factor
IPI00010120.4	CTBP2	X	X		organelle lumen,nucleus	cell proliferation,metabolic process,regulation of biological process,reproduction,cell differentiation	protein binding,nucleotide binding,catalytic activity	Isoform 1 of C-terminal-binding protein 2
IPI00183208.3	FBXO22		X		cytoplasm,nucleus	development,metabolic process,transport,regulation of biological process,response to stimulus,cell communication,cell differentiation	protein binding,catalytic activity	Isoform 1 of F-box only protein 22
IPI00218568.7	PCBD1	X	X		cytoplasm,organelle lumen,nucleus,cytosol	cell organization and biogenesis,metabolic process,regulation of biological process	protein binding,catalytic activity	Pterin-4-alpha-carbinolamine dehydratase
IPI00784985.1	IGK@			X			protein binding	IGK@ protein
IPI00856012.1	COL6A6			X	extracellular		protein binding	Isoform 1 of Collagen alpha-6(VI) chain
IPI00069750.2	PUF60	X	X		organelle lumen,nucleus	cell death,metabolic process,regulation of biological process,cellular homeostasis	protein binding,DNA binding,RNA binding,nucleotide binding,catalytic activity	Isoform 1 of Poly(U)-binding-splicing factor PUF60
IPI00921205.1	FAM171A2			X	membrane			Protein FAM171A2
IPI00151036.1	RNF13			X	membrane,endoplasmic reticulum,cytoplasm,Golgi,vacuole,nucleus,endosome	metabolic process	protein binding,metal ion binding,catalytic activity	E3 ubiquitin-protein ligase RNF13
IPI00019771.1	CX3CL1			X	extracellular,cell surface,membrane,cytoplasm	cell death,cell proliferation,development,transport,regulation of biological process,response to stimulus,cellular component movement,cell communication,defense response,cellular homeostasis,coagulation	protein binding	Fractalkine
IPI00300244.3	ZCWPW1			X			metal ion binding	Isoform 1 of Zinc finger CW-type PWWP domain protein 1
IPI00024284.5	HSPG2			X	membrane		protein binding	Basement membrane-specific heparan sulfate proteoglycan core protein
IPI00006176.3	HGS	X	X		membrane,cytoplasm,cytosol,endosome	cell proliferation,cell organization and biogenesis,metabolic process,transport,regulation of biological process,response to stimulus,cell communication	protein binding,metal ion binding	Isoform 1 of Hepatocyte growth factor-regulated tyrosine kinase substrate
IPI00373911.1	BTBD17		X		extracellular		protein binding	BTB/POZ domain-containing protein 17
IPI00009922.3	SLIRP		X		mitochondrion,cytoplasm,nucleus	metabolic process,regulation of biological process	RNA binding,nucleotide binding	SRA stem-loop-interacting RNA-binding protein, mitochondrial
IPI00216704.9	SPTB		X	X			protein binding	Isoform 2 of Spectrin beta chain, erythrocyte
IPI00792115.1	CLEC3B			X	extracellular	development		Uncharacterized protein
IPI00788612.2	LIMS1	X	X		membrane,cytoplasm,cytosol	cell organization and biogenesis,metabolic process,regulation of biological process,response to stimulus	protein binding,metal ion binding	LIM and senescent cell antigen-like-containing domain protein 1
IPI00303476.1	ATP5B	X	X	X	cell surface,membrane,mitochondrion,cytoplasm,organelle lumen	development,metabolic process,transport,regulation of biological process,cellular component movement,cellular homeostasis	protein binding,transporter activity,metal ion binding,nucleotide binding,receptor activity,catalytic activity	ATP synthase subunit beta, mitochondrial
IPI00872579.2	PCDH1	X	X	X	membrane		metal ion binding	Isoform 2 of Protocadherin-1

IPI	GENE	Alzheimer's Hippocampus	Control hippocampus	CSF	Cellular localization	Biological process	Molecular function	Protein Description
IPI00011416.2	ECH1	X	X	X	mitochondrion,cytoplasm	metabolic process	protein binding,catalytic activity	Delta(3,5)-Delta(2,4)-dienoyl-CoA isomerase, mitochondrial
IPI00021274.2	EPHA8			X	membrane,cytoplasm,endosome	development,cell organization and biogenesis,metabolic process,regulation of biological process,response to stimulus,cellular component movement,cell communication,cell differentiation	protein binding,signal transducer activity,nucleotide binding,receptor activity,catalytic activity	Ephrin type-A receptor 8
IPI00302458.10	XPO7	X	X		membrane,cytoplasm,nucleus	transport	transporter activity	Exportin-7
IPI00816555.3	LOC100293440			X	membrane		protein binding	similar to Ig lambda chain
IPI00217507.6	NEFM	X	X		cytoskeleton,membrane,cytoplasm	development,cell organization and biogenesis,metabolic process,transport,regulation of biological process,cellular component movement,cell differentiation	protein binding,structural molecule activity	Neurofilament medium polypeptide
IPI00013466.4	ASNA1	X	X	X	endoplasmic reticulum,cytoplasm,organelle lumen,nucleus	metabolic process,transport,response to stimulus	transporter activity,metal ion binding,nucleotide binding,catalytic activity	ATPase ASNA1
IPI00014235.3	RAB3GAP1	X	X		cytoskeleton,cytoplasm,organelle lumen,nucleus	development,metabolic process,regulation of biological process	protein binding,enzyme regulator activity	Isoform 1 of Rab3 GTPase-activating protein catalytic subunit
IPI00018771.3	RIC8B			X				Isoform 1 of Synembryn-B
IPI00074962.3	ANK2	X	X		cytoskeleton,membrane,cytoplasm,nucleus,cytosol	development,cell organization and biogenesis,transport,metabolic process,regulation of biological process,response to stimulus,cellular component movement,cell communication,cellular homeostasis,reproduction,cell differentiation	protein binding	Isoform 3 of Ankyrin-2
IPI00015833.1	CHCHD3	X	X		mitochondrion,membrane,cytoplasm	cell organization and biogenesis	protein binding,structural molecule activity	Coiled-coil-helix-coiled-coil-helix domain-containing protein 3, mitochondrial
IPI00030880.4	GRIA1		X		cell surface,cytoskeleton,membrane,endoplasmic reticulum,cytoplasm	metabolic process,transport,regulation of biological process,response to stimulus,cell communication	protein binding,signal transducer activity,transporter activity,receptor activity	Isoform Flop of Glutamate receptor 1
IPI00005162.3	ARPC3	X	X		cytoskeleton,cytoplasm	cell organization and biogenesis,regulation of biological process,cellular component movement	protein binding,structural molecule activity	Actin-related protein 2/3 complex subunit 3
IPI00179415.4	PPP3CA	X	X		mitochondrion,membrane,cytoplasm,organelle lumen,nucleus,cytosol	development,metabolic process,transport,regulation of biological process,response to stimulus,cell communication,cellular homeostasis,cell differentiation	protein binding,metal ion binding,catalytic activity	Isoform 1 of Serine/threonine-protein phosphatase 2B catalytic subunit alpha isoform
IPI00215743.3	RRBP1	X	X		membrane,endoplasmic reticulum,cytoplasm,ribosome,organelle lumen,nucleus	transport,metabolic process,regulation of biological process,response to stimulus,cell communication	receptor activity	Isoform 3 of Ribosome-binding protein 1
IPI00411706.1	ESD	X	X	X	cytoplasm,Golgi,nucleus	metabolic process	catalytic activity	S-formylglutathione hydrolase
IPI00642211.3	RNPEP	X			extracellular,cell surface,membrane,cytoplasm,Golgi	development,metabolic process	metal ion binding,catalytic activity	Aminopeptidase B
IPI00032202.3	WDR37	X	X				protein binding	cDNA FLJ76685, highly similar to Homo sapiens WD repeat domain 37 (WDR37), mRNA
IPI00796990.3	CFI			X	membrane	metabolic process	protein binding,receptor activity,catalytic activity	unnamed protein product
IPI00815938.4	IGLV3-21			X				Similar to V2-14 protein
IPI00005715.5	UBE4B		X		cytoplasm,nucleus	cell death,development,cell organization and biogenesis,metabolic process,response to stimulus,cell differentiation	protein binding,catalytic activity	Isoform 1 of Ubiquitin conjugation factor E4 B
IPI00550689.3	C22orf28	X	X		cytoplasm	cell organization and biogenesis,metabolic process,cell differentiation	protein binding,metal ion binding,nucleotide binding,catalytic activity	tRNA-splicing ligase RtcB homolog
IPI00719051.4	EXOC5		X		cytoplasm,cytosol	transport	protein binding	Exocyst complex component 5
IPI00020191.1	PTP4A2	X	X		membrane,cytoplasm,endosome	metabolic process	catalytic activity	Isoform 1 of Protein tyrosine phosphatase type IVA 2
IPI00291351.1	RPS6KC1		X		membrane,cytoplasm,endosome	metabolic process,regulation of biological process,response to stimulus,cell communication	protein binding,nucleotide binding,catalytic activity	Ribosomal protein S6 kinase delta-1
IPI00166518.6	MPRIP	X	X				protein binding	Isoform 3 of Myosin phosphatase Rho-interacting protein
IPI00013297.1	PDAP1	X	X			cell proliferation,regulation of biological process,response to stimulus,cell communication		28 kDa heat- and acid-stable phosphoprotein
IPI00423462.5	IGHA1			X	membrane		protein binding	Putative uncharacterized protein DKFZp686K18196 (Fragment)
IPI00922222.2	ND6		X		membrane,mitochondrion,cytoplasm	metabolic process,response to stimulus	catalytic activity	NADH-ubiquinone oxidoreductase chain 6
IPI00300838.2	CHST8			X	membrane,cytoplasm,Golgi	development,metabolic process	catalytic activity	Carbohydrate sulfotransferase 8

IPI	GENE	Alzheimer's Hippocampus	Control hippocampus	CSF	Cellular localization	Biological process	Molecular function	Protein Description
IPI00386812.1	DKK3			X	extracellular	development,regulation of biological process,response to stimulus,cell communication		RIG-like 7-1
IPI00024105.1	C1QTNF5			X	extracellular		protein binding	Complement C1q tumor necrosis factor-related protein 5
IPI00554590.1	RAB3GAP2	X			membrane,cytoplasm	transport,metabolic process,regulation of biological process	protein binding,catalytic activity,enzyme regulator activity	Isoform 1 of Rab3 GTPase-activating protein non-catalytic subunit
IPI00002352.4	MYLPF			X	cytoskeleton,cytoplasm	development,response to stimulus	metal ion binding,structural molecule activity	Myosin regulatory light chain 2, skeletal muscle isoform
IPI00031708.1	FAH			X	cytoplasm,cytosol	metabolic process	metal ion binding,catalytic activity	Fumarylacetoacetase
IPI00045928.1	SLC9A7			X	membrane,cytoplasm,Golgi,endosome	transport	protein binding,transporter activity	Sodium/hydrogen exchanger 7
IPI00014849.3	PDK3	X	X		mitochondrion,cytoplasm,organelle lumen	metabolic process,regulation of biological process,response to stimulus,cell communication	protein binding,signal transducer activity,nucleotide binding,receptor activity,catalytic activity	[Pyruvate dehydrogenase [lipoamide]] kinase isozyme 3, mitochondrial
IPI00032338.5	KLHL20			X	cell surface,cytoskeleton,cytoplasm,Golgi,organelle lumen,nucleus	cell death,cell organization and biogenesis,metabolic process,regulation of biological process,response to stimulus	protein binding,catalytic activity	Kelch-like protein 20
IPI00023942.1	SDC3			X	membrane		protein binding	Syndecan
IPI00003814.1	MAP2K6			X	cytoskeleton,cytoplasm,organelle lumen,nucleus,cytosol	cell death,metabolic process,regulation of biological process,response to stimulus,defense response,cell communication,cell differentiation	protein binding,nucleotide binding,catalytic activity	Isoform 1 of Dual specificity mitogen-activated protein kinase kinase 6
IPI00395507.4	ELAVL4	X	X				RNA binding,nucleotide binding	Isoform 2 of ELAV-like protein 4
IPI00018311.1	NPTN			X	membrane	development,cell organization and biogenesis,metabolic process,regulation of biological process,response to stimulus,cell communication,cellular homeostasis,cell differentiation	protein binding	Isoform 2 of Neuroplastin
IPI00016679.5	SLITRK5			X	membrane	development,cell organization and biogenesis,cell differentiation	protein binding	SLIT and NTRK-like protein 5
IPI00878181.4	TBC1D10B	X	X			metabolic process,regulation of biological process	catalytic activity,enzyme regulator activity	Isoform 2 of TBC1 domain family member 10B
IPI00010295.1	CPN1			X	extracellular	metabolic process,response to stimulus	metal ion binding,catalytic activity	Carboxypeptidase N catalytic chain
IPI00026833.4	ADSS	X	X		membrane,cytoplasm,cytosol	metabolic process	metal ion binding,nucleotide binding,catalytic activity	Adenylosuccinate synthetase isozyme 2
IPI00333420.6	SRPK2	X	X		cytoplasm,organelle lumen,nucleus	cell death,cell proliferation,cell organization and biogenesis,development,metabolic process,regulation of biological process,response to stimulus,defense response,cell communication,reproduction,cell differentiation	protein binding,metal ion binding,nucleotide binding,catalytic activity	Isoform 1 of Serine/threonine-protein kinase SRPK2
IPI00221224.6	ANPEP			X	membrane,cytoplasm,organelle lumen,cytosol	development,transport,metabolic process,regulation of biological process,cell differentiation	metal ion binding,receptor activity,catalytic activity	Aminopeptidase N
IPI00002243.5	GGT5			X	cell surface,membrane	metabolic process	catalytic activity	Isoform 1 of Gamma-glutamyltransferase 5
IPI00935516.1	HSBP1	X	X		cytoskeleton,nucleus	metabolic process,regulation of biological process		Heat shock factor-binding protein 1
IPI00646291.1	GPR180			X	membrane			Integral membrane protein GPR180
IPI00896449.1	SMIM1			X	membrane			Uncharacterized protein LOC388588
IPI00071180.1	UBQLN1	X	X				protein binding	Isoform 2 of Ubiquilin-1
IPI00328170.9	MOGS		X		membrane,endoplasmic reticulum,cytoplasm	metabolic process	catalytic activity	Mannosyl-oligosaccharide glucosidase
IPI00217778.1	PLTP			X				Isoform 2 of Phospholipid transfer protein
IPI00022229.1	APOB			X		transport	transporter activity	lipoprotein B100
IPI00022891.3	SLC25A4	X	X	X	membrane,mitochondrion,cytoplasm	cell death,cell organization and biogenesis,transport,metabolic process,regulation of biological process,cell communication	protein binding,transporter activity	ADP/ATP translocase 1
IPI00003842.3	MAP2	X	X		cytoskeleton,membrane,cytoplasm,organelle lumen,nucleus	cell organization and biogenesis,development,metabolic process,response to stimulus,cell differentiation	protein binding,structural molecule activity	Isoform 1 of Microtubule-associated protein 2
IPI00020984.2	CANX	X	X	X	endoplasmic reticulum,cytoplasm	metabolic process	protein binding,metal ion binding	cDNA FLJ55574, highly similar to Calnexin

IPI	GENE	Alzheimer's Hippocampus	Control hippocampus	CSF	Cellular localization	Biological process	Molecular function	Protein Description
IPI00298547.3	PARK7	X	X	X	mitochondrion,cytoplasm,cytosol,nucleus	cell death,cell organization and biogenesis,metabolic process,transport,regulation of biological process,response to stimulus,cell communication,defense response,cellular homeostasis,reproduction	antioxidant activity,protein binding,RNA binding,catalytic activity	Protein DJ-1
IPI00020966.1	PIGA			X	membrane,endoplasmic reticulum,cytoplasm	metabolic process,regulation of biological process	protein binding,catalytic activity	Isoform 1 of Phosphatidylinositol N-acetylglucosaminyltransferase subunit A
IPI00399296.6	ACCSL			X		metabolic process	catalytic activity	1-aminocyclopropane-1-carboxylate synthase-like protein 2
IPI00329572.5	PACSIN3	X	X		cytoplasm	cell organization and biogenesis,metabolic process,transport,regulation of biological process	protein binding,nucleotide binding,catalytic activity,enzyme regulator activity	Protein kinase C and casein kinase substrate in neurons 3, isoform CRA_b
IPI00299485.5	CD93			X	cell surface,membrane,cytoplasm	transport	protein binding,metal ion binding,receptor activity	Complement component C1q receptor
IPI00410436.5	KIAA1211L	X	X					Uncharacterized protein C2orf55
IPI00445315.2	FAM47C			X	membrane	regulation of biological process,response to stimulus,cell communication	signal transducer activity,receptor activity,catalytic activity	Putative protein FAM47C
IPI00009960.6	IMMT	X	X		membrane,mitochondrion,cytoplasm		protein binding	Isoform 1 of Mitochondrial inner membrane protein
IPI00290416.3	OLA1	X	X		membrane,cytoplasm	transport,metabolic process	transporter activity,nucleotide binding,catalytic activity	Isoform 1 of Obg-like ATPase 1
IPI00184376.5	SCAI		X		membrane			Isoform 2 of Protein SCAI
IPI00061507.2	C11orf54	X	X	X	nucleus			Isoform 3 of Ester hydrolase C11orf54
IPI00298497.3	FGB	X	X	X	extracellular,cell surface,membrane,cytoplasm,organelle lumen	cell organization and biogenesis,transport,regulation of biological process,response to stimulus,cell communication,reproduction,coagulation	protein binding	Fibrinogen beta chain
IPI00157790.7	KIAA0368		X					proteasome-associated protein ECM29 homolog
IPI00328154.4	UBA3	X	X		nucleus	transport,metabolic process,regulation of biological process	protein binding,transporter activity,nucleotide binding,catalytic activity	Isoform 1 of NEDD8-activating enzyme E1 catalytic subunit
IPI00022333.1	BAI1			X	membrane	cell proliferation,cell organization and biogenesis,development,regulation of biological process,response to stimulus,cell communication,cell differentiation	signal transducer activity,receptor activity	Brain-specific angiogenesis inhibitor 1
IPI00026237.1	MAG	X	X	X	membrane	development,cell organization and biogenesis,regulation of biological process,response to stimulus,cellular component movement,cell communication,cell differentiation,coagulation	protein binding	Myelin-associated glycoprotein
IPI00238209.3	NUDCD3	X	X					NudC domain-containing protein 3
IPI00149375.3	FAM160A2			X				Isoform 2 of FTS and Hook-interacting protein
IPI00167006.3	TEX26			X				Uncharacterized protein C13orf26
IPI00013681.2	TRPC3			X	membrane	transport	transporter activity	short transient receptor potential channel 3 isoform a
IPI00220267.7	ASL		X		cytoplasm,cytosol	metabolic process	catalytic activity	Argininosuccinate lyase
IPI00443534.1	NSFL1C		X				protein binding	cDNA FLJ46889 fis, clone UTERU3017995, highly similar to Homo sapiens likely ortholog of rat p47
IPI00012119.1	DCN			X	extracellular	development,metabolic process,response to stimulus	protein binding	Isoform A of Decorin
IPI00009342.1	IQGAP1	X	X		membrane	regulation of biological process,response to stimulus,cell communication	protein binding,enzyme regulator activity	Ras GTPase-activating-like protein IQGAP1
IPI00555956.2	PSMB4	X	X		cytoplasm,proteasome,organelle lumen,cytosol,nucleus	cell death,metabolic process,regulation of biological process,response to stimulus,cell communication,defense response	catalytic activity	Proteasome subunit beta type-4
IPI00014230.1	C1QBP	X	X		extracellular,membrane,mitochondrion,cytoplasm,organelle lumen,nucleus	metabolic process,response to stimulus,coagulation	protein binding	Complement component 1 Q subcomponent-binding protein, mitochondrial
IPI00465436.4	CAT	X	X	X	membrane,mitochondrion,endoplasmic reticulum,cytoplasm,Golgi,organelle lumen,vacuole,cytosol	cell death,cell organization and biogenesis,development,metabolic process,cell division,regulation of biological process,response to stimulus,cell communication,reproduction	antioxidant activity,protein binding,metal ion binding,nucleotide binding,catalytic activity	Catalase
IPI00022608.1	SORL1			X	membrane	metabolic process,response to stimulus	protein binding,metal ion binding,nucleotide binding,catalytic activity	sortilin-related receptor preproprotein
IPI00011937.1	PRDX4		X		extracellular,mitochondrion,cytoplasm	metabolic process,regulation of biological process,response to stimulus,cell communication	antioxidant activity,protein binding,catalytic activity	Peroxiredoxin-4

IPI	GENE	Alzheimer's Hippocampus	Control hippocampus	CSF	Cellular localization	Biological process	Molecular function	Protein Description
IPI00221233.6	BCAP29	X			mitochondrion,membrane,endoplasmic reticulum,cytoplasm	cell death,transport	receptor activity	B-cell receptor-associated protein 29
IPI00009841.6	EWSR1		X				metal ion binding	RNA-binding protein EWS isoform 1
IPI00021805.1	MGST1	X	X		mitochondrion,membrane,endoplasmic reticulum,cytoplasm,nucleus	cell organization and biogenesis,development,metabolic process,response to stimulus,reproduction,cell differentiation	antioxidant activity,protein binding,catalytic activity	Microsomal glutathione S-transferase 1
IPI00018452.3	CPNE1	X	X			transport,metabolic process	protein binding,transporter activity	Copine-1
IPI00004573.2	PIGR			X	extracellular,membrane,cytoplasm,endosome		protein binding	Polymeric immunoglobulin receptor
IPI00033494.3	MYL12B	X	X		cytoskeleton,cytoplasm,cytosol	development,cell organization and biogenesis,regulation of biological process,response to stimulus,cell differentiation	metal ion binding	Myosin regulatory light chain 12B
IPI00007632.2	GRIA4		X	X	cytoskeleton,membrane,cytoplasm	transport,regulation of biological process,response to stimulus,cell communication	signal transducer activity,transporter activity,receptor activity	Glutamate receptor 4
IPI00185661.5	USP32			X	membrane,cytoplasm,Golgi	metabolic process	metal ion binding,catalytic activity	Ubiquitin carboxyl-terminal hydrolase 32
IPI00020501.1	MYH11			X	cytoskeleton,membrane,cytoplasm,cytosol	development,cell organization and biogenesis,metabolic process,response to stimulus,cell differentiation	protein binding,nucleotide binding,motor activity,structural molecule activity,catalytic activity	Myosin-11
IPI00412785.3	MPC1	X			membrane,mitochondrion,cytoplasm	transport,metabolic process	catalytic activity	Brain protein 44-like protein
IPI00303258.3	LMCD1		X		extracellular,cytoplasm,nucleus	metabolic process,regulation of biological process,response to stimulus,cell communication	metal ion binding	LIM and cysteine-rich domains protein 1
IPI00303158.3	CMAS	X	X		nucleus	metabolic process	catalytic activity	Isoform 1 of N-acylneuraminate cytidylyltransferase
IPI00423466.1	IGHG1			X	membrane		protein binding	Putative uncharacterized protein DKFZp686H20196
IPI00852623.2	PLXNB2	X	X			development,cell organization and biogenesis,regulation of biological process,cell differentiation		16 kDa protein
IPI00026625.1	NUP155	X			membrane,nucleus	cell organization and biogenesis,metabolic process,transport,regulation of biological process,response to stimulus,cell communication	transporter activity,structural molecule activity	Isoform 1 of Nuclear pore complex protein Nup155
IPI00019599.2	UBE2V1	X	X				catalytic activity	Isoform 1 of Ubiquitin-conjugating enzyme E2 variant 1
IPI00004436.1	LSM1	X	X		cytoplasm,cytosol,nucleus	metabolic process	protein binding,RNA binding	U6 snRNA-associated Sm-like protein LSm1
IPI00032473.1	PPIL3	X	X			metabolic process	catalytic activity	Isoform 2 of Peptidyl-prolyl cis-trans isomerase-like 3
IPI00022959.1	PVRL3			X	membrane	cell organization and biogenesis,development,reproduction	protein binding	Isoform 1 of Poliovirus receptor-related protein 3
IPI00018708.1	CEP63			X		regulation of biological process,response to stimulus,cell communication		Isoform 2 of Centrosomal protein of 63 kDa
IPI00027464.8	PPP3R1	X	X	X	cytoplasm,cytosol	cell death,regulation of biological process	protein binding,metal ion binding,catalytic activity	Calcineurin subunit B type 1
IPI00216728.2	NRXN3	X	X	X			protein binding	Uncharacterized protein
IPI00220808.1	PFKFB2	X	X			metabolic process	nucleotide binding,catalytic activity	Isoform 2 of 6-phosphofructo-2-kinase/fructose-2,6-biphosphatase 2
IPI00164776.2	TMEM198			X	membrane			Transmembrane protein 198
IPI00872359.2	DCTN1		X		cytoskeleton,membrane,cytoplasm,chromosome,cytosol	cell death,cell organization and biogenesis,development,transport,metabolic process,regulation of biological process,response to stimulus,cell communication	protein binding,motor activity,catalytic activity	Isoform p150 of Dynactin subunit 1
IPI00024976.5	TOMM22	X	X	X	mitochondrion,membrane,cytoplasm	cell organization and biogenesis,metabolic process,transport	protein binding,transporter activity,receptor activity	Mitochondrial import receptor subunit TOM22 homolog
IPI00011140.3	NOV			X	extracellular	cell organization and biogenesis,regulation of biological process,cell growth	protein binding	Protein NOV homolog
IPI00004902.1	ETFB	X	X		mitochondrion,cytoplasm,organelle lumen	metabolic process,transport		Isoform 1 of Electron transfer flavoprotein subunit beta
IPI00294536.2	STRAP	X	X		mitochondrion,membrane,cytoplasm,nucleus	metabolic process	protein binding,receptor activity,catalytic activity	cDNA FLJ51909, highly similar to Serine-threonine kinase receptor-associatedprotein
IPI00909904.1	STK24		X			metabolic process	nucleotide binding,catalytic activity	cDNA FLJ61383, highly similar to Serine/threonine-protein kinase 24
IPI00300371.5	SF3B3	X	X		spliceosomal complex,organelle lumen,nucleus	cell organization and biogenesis,metabolic process	protein binding	Isoform 1 of Splicing factor 3B subunit 3
IPI00432755.2	FAM124A			X				PPRR6495

IPI	GENE	Alzheimer's Hippocampus	Control hippocampus	CSF	Cellular localization	Biological process	Molecular function	Protein Description
IPI00002320.1	FLRT3			X	extracellular,membrane		protein binding,signal transducer activity	Leucine-rich repeat transmembrane protein FLRT3
IPI00027485.3	EIF4E		X		cytoplasm,cytosol	metabolic process,transport,regulation of biological process,response to stimulus,cell communication	protein binding,RNA binding	Eukaryotic translation initiation factor 4E
IPI00178302.6	SEMA6D			X	membrane,cytoplasm,Golgi	cell organization and biogenesis,development,response to stimulus,cell differentiation	protein binding,receptor activity	Isoform 4 of Semaphorin-6D
IPI00016467.2	SLITRK3			X	membrane	cell organization and biogenesis,development,cell differentiation	protein binding	SLIT and NTRK-like protein 3
IPI00103471.3	SELM		X	X	cytoplasm,Golgi	metabolic process	metal ion binding,catalytic activity	Selenoprotein M
IPI00032406.1	DNAJA2	X	X		membrane	cell proliferation,metabolic process,regulation of biological process,response to stimulus	protein binding,metal ion binding,nucleotide binding	DnaJ homolog subfamily A member 2
IPI00185374.4	PSMD12	X	X		proteasome,cytoplasm,organelle lumen,cytosol,nucleus	cell death,metabolic process,regulation of biological process,response to stimulus,cell communication	protein binding	26S proteasome non-ATPase regulatory subunit 12
IPI00747657.1	RNPS1P1			X				Conserved hypothetical protein
IPI00016077.1	GBAS	X	X		mitochondrion,membrane,cytoplasm		protein binding	Protein NipSnap homolog 2
IPI00218131.3	S100A12			X	extracellular,cytoplasm,cytosol,nucleus	metabolic process,regulation of biological process,response to stimulus,cell communication,defense response	protein binding,metal ion binding	Protein S100-A12
IPI00305152.6	SEC31A	X	X		organelle lumen,nucleus	metabolic process,regulation of biological process		Isoform 3 of Protein transport protein Sec31A
IPI00394879.6	LRRC9			X				Isoform 2 of Leucine-rich repeat-containing protein 9
IPI00401852.6	DKFZP434L187			X				Putative uncharacterized protein DKFZp434L187
IPI00012474.3	TMEM35		X		membrane,cytoplasm			Transmembrane protein 35
IPI00297646.4	COL1A1			X	extracellular		protein binding,structural molecule activity	collagen alpha-1(I) chain preproprotein
IPI00739106.3	RPL5P1			X	cytoplasm,ribosome	metabolic process	RNA binding,structural molecule activity	similar to MSTP030 isoform 1
IPI00549467.3	NIT2	X	X		cytoskeleton,mitochondrion,cytoplasm	metabolic process	catalytic activity	Omega-amidase NIT2
IPI00152695.2	WDR82	X			organelle lumen,chromosome,nucleus	cell organization and biogenesis,metabolic process	protein binding,catalytic activity	WD repeat-containing protein 82
IPI00413778.7	FKBP1A			X		metabolic process	catalytic activity	FKBP1A protein
IPI00171678.4	DBH			X	extracellular,membrane,cytoplasm,organelle lumen	cell death,cell proliferation,development,metabolic process,regulation of biological process,response to stimulus,cellular component movement,cell communication,reproduction	metal ion binding,catalytic activity	Dopamine beta-hydroxylase
IPI00026259.2	AGA			X	endoplasmic reticulum,cytoplasm,vacuole	metabolic process	protein binding,catalytic activity	N(4)-(beta-N-acetylglucosaminyl)-L-asparaginase
IPI00000728.3	USP15	X	X			metabolic process	catalytic activity	Isoform 1 of Ubiquitin carboxyl-terminal hydrolase 15
IPI00301216.6	TBC1D13	X				metabolic process,regulation of biological process	enzyme regulator activity	Isoform 2 of TBC1 domain family member 13
IPI00549672.2	PSMD13	X	X				protein binding	HSPC027
IPI00291316.5	ARHGEF2		X		cytoskeleton,membrane,cytoplasm,Golgi,cytosol	cell death,cell proliferation,cell organization and biogenesis,development,cell division,transport,metabolic process,regulation of biological process,response to stimulus,cell communication,cell differentiation	protein binding,metal ion binding,enzyme regulator activity	Isoform 1 of Rho guanine nucleotide exchange factor 2
IPI00945153.2	NDUFA6	X	X		mitochondrion,membrane,cytoplasm	metabolic process,transport,response to stimulus	catalytic activity	NADH dehydrogenase [ubiquinone] 1 alpha subcomplex subunit 6
IPI00556643.2	SEMA3F			X	membrane	development	protein binding,receptor activity	Uncharacterized protein
IPI00154734.3	SEZ6			X	extracellular,membrane	cell organization and biogenesis,development,regulation of biological process,cell communication,cellular homeostasis,cell differentiation		Isoform 1 of Seizure protein 6 homolog
IPI00084828.1	STXBP1	X	X		membrane,mitochondrion,cytoplasm,cytosol	development,cell organization and biogenesis,transport,metabolic process,regulation of biological process,response to stimulus,cell communication,cell differentiation,coagulation	protein binding	Isoform 1 of Syntaxin-binding protein 1
IPI00333770.6	DOCK10		X				protein binding,nucleotide binding,enzyme regulator activity	Isoform 1 of Dedicator of cytokinesis protein 10

IPI	GENE	Alzheimer's Hippocampus	Control hippocampus	CSF	Cellular localization	Biological process	Molecular function	Protein Description
IPI00295240.6	NFS1		X		mitochondrion,cytoplasm,organelle lumen,cytosol,nucleus	cell organization and biogenesis,metabolic process	protein binding,catalytic activity	Isoform Mitochondrial of Cysteine desulfurase, mitochondrial
IPI00009477.4	ICAM2			X	membrane	regulation of biological process,response to stimulus	protein binding	Intercellular adhesion molecule 2
IPI00015018.1	PPA1	X	X		cytoplasm,cytosol	metabolic process	metal ion binding,catalytic activity	Inorganic pyrophosphatase
IPI00059242.3	SYAP1	X	X		nucleus			Synapse-associated protein 1
IPI00644818.1	TTLL9			X		metabolic process	catalytic activity	27 kDa protein
IPI00059135.1	PPP1R14A	X	X		cytoplasm	metabolic process,regulation of biological process	enzyme regulator activity	Isoform 1 of Protein phosphatase 1 regulatory subunit 14A
IPI00165931.7	PLXNA4	X	X		membrane	cell organization and biogenesis,development,regulation of biological process,response to stimulus,cellular component movement,cell communication,cell growth,cell differentiation	protein binding,receptor activity	Isoform 1 of Plexin-A4
IPI00305692.5	TXNL1	X	X		cytoplasm	transport,metabolic process,regulation of biological process,cellular homeostasis	catalytic activity	Thioredoxin-like protein 1
IPI00453473.6	HIST2H4B	X	X	X	extracellular,organelle lumen,chromosome,nucleus	development,cell organization and biogenesis,metabolic process,regulation of biological process,response to stimulus,cell communication,cell differentiation	protein binding,DNA binding	Histone H4
IPI00247583.5	RPL21	X	X		cytoplasm,ribosome,cytosol	cell organization and biogenesis,metabolic process,transport,reproduction	RNA binding,structural molecule activity	60S ribosomal protein L21
IPI00006608.1	APP	X	X	X	extracellular,cytoskeleton,cell surface,membrane,cytoplasm,Golgi,organelle lumen,cytosol	cell death,development,cell organization and biogenesis,metabolic process,regulation of biological process,response to stimulus,defense response,reproduction,transport,cellular component movement,cell communication,cellular homeostasis,cell growth,cell differentiation,coagulation	protein binding,DNA binding,metal ion binding,enzyme regulator activity	Isoform APP770 of Amyloid beta A4 protein (Fragment)
IPI00011643.1	SPINT1			X			protein binding,enzyme regulator activity	Isoform 2 of Kunitz-type protease inhibitor 1
IPI00257932.1	TCEAL5		X		nucleus	metabolic process,regulation of biological process	protein binding	Transcription elongation factor A protein-like 5
IPI00398874.4	ACBD7	X	X					Acyl-CoA-binding domain-containing protein 7
IPI00290928.2	GNA13	X	X		membrane,cytoplasm	development,cell organization and biogenesis,metabolic process,regulation of biological process,response to stimulus,cellular component movement,cell communication,cell differentiation,coagulation	signal transducer activity,protein binding,metal ion binding,nucleotide binding,catalytic activity	Guanine nucleotide-binding protein subunit alpha-13
IPI00026272.2	HIST1H2AB			X	chromosome,nucleus	cell organization and biogenesis,metabolic process	DNA binding	Histone H2A type 1-B/E
IPI00554681.2	NDUFA5	X	X		membrane,mitochondrion,cytoplasm	metabolic process,transport	catalytic activity	NADH dehydrogenase [ubiquinone] 1 alpha subcomplex subunit 5
IPI00743104.2	ITGA1	X			cell surface,membrane,cytoplasm	cell death,cell organization and biogenesis,development,metabolic process,regulation of biological process,response to stimulus,cellular component movement,cell communication,cell differentiation	protein binding,receptor activity	Integrin alpha-1
IPI00005132.3	GNL3L			X	membrane,mitochondrion,cytoplasm,organelle lumen,nucleus	transport,metabolic process	protein binding,transporter activity,nucleotide binding,catalytic activity	Guanine nucleotide-binding protein-like 3-like protein
IPI00097856.6	SLC4A8		X		membrane	transport	transporter activity	Isoform 2 of Electroneutral sodium bicarbonate exchanger 1
IPI00441473.3	PRMT5	X	X		cytoplasm,cytosol,nucleus	cell proliferation,cell organization and biogenesis,metabolic process,regulation of biological process	protein binding,catalytic activity	Protein arginine N-methyltransferase 5
IPI00604763.2	TMEM66			X	membrane,endoplasmic reticulum,cytoplasm	transport,regulation of biological process	protein binding	Transmembrane protein 66
IPI00024307.1	EFNB1			X	membrane,cytoplasm,nucleus	cell proliferation,development,cell organization and biogenesis,regulation of biological process,response to stimulus,cellular component movement,cell communication,cell differentiation	protein binding	Ephrin-B1
IPI00013890.2	SFN	X			extracellular,cytoplasm,nucleus	cell death,cell proliferation,development,cell organization and biogenesis,metabolic process,transport,regulation of biological process,response to stimulus,cell communication,cell growth,cell differentiation	protein binding,enzyme regulator activity	Isoform 1 of 14-3-3 protein sigma

IPI	GENE	Alzheimer's Hippocampus	Control hippocampus	CSF	Cellular localization	Biological process	Molecular function	Protein Description
IPI00006054.1	SNPH	X	X	X	cytoplasm	regulation of biological process,response to stimulus,cell communication	protein binding	Isoform 2 of Syntaphilin
IPI00027782.1	MMP3			X	extracellular,nucleus	cell organization and biogenesis,metabolic process,regulation of biological process,response to stimulus,cellular component movement,cell communication,reproduction	protein binding,metal ion binding,catalytic activity	Stromelysin-1
IPI00386930.6	RAP1GAP	X	X			regulation of biological process,response to stimulus,cell communication	enzyme regulator activity	Isoform 2 of Rap1 GTPase-activating protein 1
IPI00306290.5	XPOT	X	X		membrane,cytoplasm,organelle lumen,nucleus	transport	transporter activity,RNA binding	Exportin-T
IPI00024346.1	SNAPC3			X	organelle lumen,nucleus	metabolic process,regulation of biological process	DNA binding	snRNA-activating protein complex subunit 3
IPI00152253.3	TMEM163		X		membrane,cytoplasm,endosome	transport	transporter activity,metal ion binding	Isoform 1 of Transmembrane protein 163
IPI00554718.1	EPDR1			X	extracellular		metal ion binding	mammalian ependymin-related protein 1 isoform 1 precursor
IPI00329373.3	C19orf70		X		mitochondrion,cytoplasm			Protein QIL1
IPI00305423.6	CHMP6		X		membrane,cytoplasm,cytosol,endosome	cell organization and biogenesis,transport	protein binding	Charged multivesicular body protein 6
IPI00021000.1	SPP1	X	X	X	extracellular,cytoplasm	cell organization and biogenesis,development,regulation of biological process,response to stimulus,cellular component movement,defense response,cell differentiation,cell growth,reproduction	protein binding	Isoform A of Osteopontin
IPI00218407.6	ALDOB			X	cytoskeleton,membrane,endoplasmic reticulum,cytoplasm,vacuole,nucleus,cytosol	development,cell organization and biogenesis,metabolic process,regulation of biological process,response to stimulus,cell communication	protein binding,catalytic activity	Fructose-bisphosphate aldolase B
IPI00414819.3	SKIV2L		X		cytoplasm,nucleus		RNA binding,nucleotide binding,catalytic activity	Helicase SKI2W
IPI00182194.8	ODZ2			X	cytoskeleton,membrane	regulation of biological process,response to stimulus,cell communication	protein binding,catalytic activity	Teneurin-2
IPI00028946.2	RTN3	X	X		endoplasmic reticulum,cytoplasm			Isoform 3 of Reticulon-3
IPI00217512.4	GPR115		X		membrane	regulation of biological process,response to stimulus,cell communication	signal transducer activity,receptor activity	Probable G-protein coupled receptor 115
IPI00329633.5	TARS	X	X		cytoskeleton,cytoplasm,cytosol	metabolic process	protein binding,nucleotide binding,catalytic activity	Threonyl-tRNA synthetase, cytoplasmic
IPI00024282.1	RAB8B	X	X		membrane,mitochondrion,cytoplasm,nucleus	cell organization and biogenesis,metabolic process,transport,regulation of biological process,response to stimulus,cell communication	protein binding,nucleotide binding,catalytic activity	Ras-related protein Rab-8B
IPI00013976.4	LAMB1			X	extracellular,cytoskeleton,membrane,mitochondrion,cytoplasm,nucleus	cell proliferation,development,cell organization and biogenesis,transport,metabolic process,regulation of biological process,response to stimulus,cellular component movement,cell communication,reproduction,cell differentiation	protein binding,signal transducer activity,nucleotide binding,motor activity,structural molecule activity,catalytic activity	Laminin subunit beta-1
IPI00013701.1	PNOC			X	extracellular	regulation of biological process,response to stimulus,cell communication	protein binding	Prepronociceptin
IPI00005202.2	PGRMC2	X	X				metal ion binding	membrane-associated progesterone receptor component 2
IPI00018402.1	TBCE	X	X		cytoskeleton,cytoplasm	development,cell organization and biogenesis,metabolic process,response to stimulus,cell differentiation	protein binding	Tubulin-specific chaperone E
IPI00016786.1	CDC42	X	X		cytoskeleton,membrane,cytoplasm,Golgi,cytosol	development,cell organization and biogenesis,transport,cell division,metabolic process,regulation of biological process,response to stimulus,cellular component movement,cell communication,cell differentiation,coagulation	protein binding,nucleotide binding,catalytic activity	Isoform 2 of Cell division control protein 42 homolog
IPI00844090.1	COL5A1			X	extracellular	cell organization and biogenesis,development,metabolic process,regulation of biological process,response to stimulus,cellular component movement,cell differentiation	protein binding,structural molecule activity	Collagen alpha-1(V) chain
IPI00252731.4	DPP6	X	X	X	membrane	metabolic process	catalytic activity	Isoform DPPX-S of Dipeptidyl aminopeptidase-like protein 6
IPI00221332.8	DNM3	X	X	X	cytoskeleton,cytoplasm	development,cell organization and biogenesis,transport,metabolic process	protein binding,nucleotide binding,catalytic activity	Isoform 1 of Dynamin-3
IPI00011913.1	HNRNPA0		X		organelle lumen,nucleus	metabolic process,regulation of biological process,response to stimulus,defense response	protein binding,RNA binding,nucleotide binding	Heterogeneous nuclear ribonucleoprotein A0
IPI00926254.1	MAP2	X						Uncharacterized protein

IPI	GENE	Alzheimer's Hippocampus	Control hippocampus	CSF	Cellular localization	Biological process	Molecular function	Protein Description
IPI00031836.3	DRG1		X		cytoskeleton,membrane,cytoplasm,nucleus	development,metabolic process,transport	protein binding,transporter activity,nucleotide binding,catalytic activity	Developmentally-regulated GTP-binding protein 1
IPI00017841.3	OLFM1	X	X	X	cytoskeleton,mitochondrion,membrane,endoplasmic reticulum,cytoplasm,organelle lumen,chromosome,nucleus	development,cell organization and biogenesis,reproduction	protein binding,motor activity,catalytic activity	Isoform 1 of Noelin
IPI00027848.1	MRC1			X	membrane,cytoplasm,endosome	transport	receptor activity	Isoform 1 of Macrophage mannose receptor 1
IPI00045511.1	CLCC1			X	membrane,endoplasmic reticulum,cytoplasm,Golgi,nucleus	transport	transporter activity	Isoform 1 of Chloride channel CLIC-like protein 1
IPI00940960.1	NPC2			X	extracellular,cytoplasm,vacuole	transport,metabolic process,regulation of biological process,response to stimulus	protein binding	Epididymal secretory protein E1
IPI00215777.1	SLC25A3	X	X	X	mitochondrion,membrane,cytoplasm			Isoform B of Phosphate carrier protein, mitochondrial
IPI00012074.3	HNRNPR	X	X		spliceosomal complex,cytoplasm,organelle lumen,nucleus	metabolic process	protein binding,RNA binding,nucleotide binding	Isoform 1 of Heterogeneous nuclear ribonucleoprotein R
IPI00930214.1	PAK3	X	X			metabolic process	protein binding,nucleotide binding,catalytic activity	PAK2
IPI00789455.3	KATNAL1	X	X		cytoskeleton,cytoplasm	metabolic process	nucleotide binding,catalytic activity	Katanin p60 ATPase-containing subunit A-like 1
IPI00024166.3	MRAS	X	X		membrane	development,cell organization and biogenesis,metabolic process,regulation of biological process,response to stimulus,cell communication	protein binding,nucleotide binding,catalytic activity	Ras-related protein M-Ras
IPI00304710.6	SPHKAP		X		cytoplasm		protein binding	Isoform 1 of A-kinase anchor protein SPHKAP
IPI00010080.2	OXSR1	X	X		cytoplasm	metabolic process,regulation of biological process,response to stimulus,cell communication	metal ion binding,nucleotide binding,catalytic activity	Serine/threonine-protein kinase OSR1
IPI00024757.1	UQCR11	X	X		membrane,mitochondrion,cytoplasm	metabolic process,transport	transporter activity,catalytic activity	Cytochrome b-c1 complex subunit 10
IPI00220797.1	ENSA	X	X					Isoform 2 of Alpha-endosulfine
IPI00737429.3	ODZ4			X	membrane	development,regulation of biological process,response to stimulus,cell communication	protein binding	Teneurin-4
IPI00014177.3	Sep-02	X	X		cytoskeleton,cell surface,membrane,cytoplasm,organelle lumen,chromosome,nucleus	cell organization and biogenesis,development,transport,metabolic process,cell division,regulation of biological process,response to stimulus,cell communication,cell differentiation	protein binding,nucleotide binding,structural molecule activity,enzyme regulator activity	Isoform 1 of Septin-2
IPI00005089.1	TMOD2	X	X		cytoskeleton,cytoplasm	development,regulation of biological process,response to stimulus,cell communication	protein binding	Isoform 1 of Tropomodulin-2
IPI00023780.3	DNAJC5	X	X	X		metabolic process	protein binding	Isoform 2 of DnaJ homolog subfamily C member 5
IPI00170935.1	LRRC47	X	X				protein binding,RNA binding,catalytic activity	Leucine-rich repeat-containing protein 47
IPI00075248.1 1	CALM3	X	X	X	extracellular,cytoskeleton,membrane,cytoplasm,organelle lumen,cytosol,nucleus	transport,metabolic process,cell division,regulation of biological process,response to stimulus,cell communication,cellular homeostasis,coagulation	protein binding,metal ion binding,catalytic activity	Calmodulin
IPI00003949.1	UBE2N	X	X		cytoplasm,cytosol,nucleus	cell organization and biogenesis,metabolic process,regulation of biological process,response to stimulus,cell communication,defense response	protein binding,nucleotide binding,catalytic activity	Ubiquitin-conjugating enzyme E2 N
IPI00153011.1	TRIM2	X	X		cytoplasm	metabolic process	protein binding,metal ion binding,catalytic activity	Tripartite motif-containing protein 2
IPI00829947.3	IGKV1-5			X				Similar to Ig kappa chain V-II region GM607 precursor
IPI00300407.4	SDC2			X	membrane	cell organization and biogenesis,development,response to stimulus,cell differentiation	protein binding	Syndecan-2
IPI00296526.6	NAGK	X	X			metabolic process	catalytic activity	N-acetyl-D-glucosamine kinase
IPI00009931.3	HDHD3	X	X		mitochondrion,cytoplasm	metabolic process	protein binding,catalytic activity	Haloacid dehalogenase-like hydrolase domain-containing protein 3
IPI00479296.2	ABCA8	X			membrane	transport,metabolic process	transporter activity,nucleotide binding,catalytic activity	Isoform 1 of ATP-binding cassette sub-family A member 8
IPI00550949.3	BMP7			X	extracellular	cell death,cell proliferation,development,cell organization and biogenesis,transport,metabolic process,regulation of biological process,response to stimulus,cell communication,cell differentiation,reproduction	protein binding	Bone morphogenetic protein 7
IPI00550263.1	CDC42BPA			X	extracellular,cytoskeleton,membrane,cytoplasm,Golgi,organelle lumen,chromosome,nucleus	cell organization and biogenesis,transport,metabolic process,regulation of biological process,response to stimulus,cell communication,reproduction	protein binding,nucleotide binding,motor activity,catalytic activity,enzyme regulator activity	Isoform 5 of Serine/threonine-protein kinase MRCK alpha

IPI	GENE	Alzheimer's Hippocampus	Control hippocampus	CSF	Cellular localization	Biological process	Molecular function	Protein Description
IPI00012989.2	MAN2B1			X	cytoplasm,vacuole	metabolic process,response to stimulus	metal ion binding,catalytic activity	Lysosomal alpha-mannosidase
IPI00853400.1	FKBP15			X	extracellular,cytoskeleton,mitochondrion,membrane,cytoplasm,Golgi,organelle lumen,chromosome,nucleus,endosome	development,cell organization and biogenesis,transport,metabolic process,regulation of biological process,cellular component movement,cell differentiation,reproduction	protein binding,transporter activity,motor activity,catalytic activity	Isoform 1 of FK506-binding protein 15
IPI00455967.9	LOC644717			X	membrane			Putative SAGE1-like protein
IPI00065931.4	AKAP13			X		regulation of biological process,response to stimulus,cell communication	protein binding,enzyme regulator activity	Isoform 2 of A-kinase anchor protein 13
IPI00024704.1	UST			X	membrane,cytoplasm,Golgi	metabolic process	catalytic activity	Uronyl 2-sulfotransferase
IPI00902997.1	FAM134A		X					cDNA FLJ33692 fis, clone BRAWH2003000
IPI00307611.6	MAST4			X		metabolic process	protein binding,metal ion binding,nucleotide binding,catalytic activity	Isoform 1 of Microtubule-associated serine/threonine-protein kinase 4
IPI00024129.1	PPIC			X	cytoplasm	metabolic process,regulation of biological process,response to stimulus,cell communication	protein binding,catalytic activity	Peptidyl-prolyl cis-trans isomerase C
IPI00009028.2	CLEC3B		X	X	extracellular,cytoplasm,organelle lumen,nucleus	development,metabolic process,regulation of biological process,response to stimulus	protein binding,metal ion binding	Tetranectin
IPI00180707.8	FREM2			X	extracellular,membrane	development,cell communication	metal ion binding	Isoform 1 of FRAS1-related extracellular matrix protein 2
IPI00009791.1	ATP2B2	X	X		membrane,endoplasmic reticulum,cytoplasm	cell organization and biogenesis,development,metabolic process,transport,regulation of biological process,response to stimulus,cell communication,cellular homeostasis,cell differentiation,reproduction,coagulation	protein binding,transporter activity,metal ion binding,nucleotide binding,catalytic activity	Isoform WB of Plasma membrane calcium-transporting ATPase 2
IPI00305486.3	AMPH	X	X		cytoskeleton,membrane,cytoplasm	cell organization and biogenesis,transport,metabolic process,regulation of biological process,response to stimulus,cell communication	protein binding	Isoform 1 of Amphiphysin
IPI00024572.3	ASPH			X	membrane			Isoform Junctin-1 of Aspartyl/asparaginyl beta-hydroxylase
IPI00060569.1	ABHD12		X			metabolic process	catalytic activity	Isoform 2 of Monoacylglycerol lipase ABHD12
IPI00844000.1	UFL1		X		endoplasmic reticulum,cytoplasm,organelle lumen,nucleus	metabolic process,regulation of biological process	protein binding,catalytic activity	Isoform 1 of E3 UFM1-protein ligase 1
IPI00000041.1	RHOB	X	X		membrane,cytoplasm,cytosol,nucleus,endosome	cell death,cell organization and biogenesis,development,transport,metabolic process,cell division,regulation of biological process,response to stimulus,cell communication,cell differentiation,coagulation	protein binding,nucleotide binding,catalytic activity	Rho-related GTP-binding protein RhoB
IPI00021840.1	RPS6	X	X		cytoplasm,ribosome,organelle lumen,nucleus,cytosol	cell death,cell organization and biogenesis,metabolic process,transport,regulation of biological process,response to stimulus,cell communication,reproduction	protein binding,structural molecule activity	40S ribosomal protein S6
IPI00002491.4	SORBS1	X	X	X			protein binding	Isoform 9 of Sorbin and SH3 domain-containing protein 1
IPI00016576.3	GRHL2			X	cytoplasm,organelle lumen,nucleus	metabolic process,regulation of biological process	DNA binding	Isoform 1 of Grainyhead-like protein 2 homolog
IPI00030205.3	IGKV3-20			X	extracellular,membrane	metabolic process,regulation of biological process,response to stimulus,defense response	protein binding	Ig kappa chain V-III region HAH
IPI00550894.4	GNPDA2	X	X		cytoplasm	metabolic process	catalytic activity	Isoform 1 of Glucosamine-6-phosphate isomerase 2
IPI00012386.1	COCH			X	extracellular	cell organization and biogenesis,regulation of biological process	protein binding	Cochlin
IPI00877929.2	CASK	X	X			metabolic process	protein binding,nucleotide binding,catalytic activity	Isoform 3 of Peripheral plasma membrane protein CASK
IPI00221240.3	LNPEP		X				metal ion binding,catalytic activity	Isoform 2 of Leucyl-cystinyl aminopeptidase
IPI00401939.6	RAP1GAP2		X			regulation of biological process,response to stimulus,cell communication	enzyme regulator activity	Isoform 3 of Rap1 GTPase-activating protein 2
IPI00167967.3	HEATR7A		X		membrane			Isoform 7 of HEAT repeat-containing protein 7A
IPI00022892.2	THY1	X	X	X	cell surface,membrane,endoplasmic reticulum,cytoplasm,cytosol	development,cell organization and biogenesis,metabolic process,transport,regulation of biological process,response to stimulus,cellular component movement,cell communication,cellular homeostasis,cell differentiation	protein binding,enzyme regulator activity	Thy-1 membrane glycoprotein
IPI00216085.3	COX6B1	X	X		mitochondrion,membrane,cytoplasm	metabolic process	transporter activity,catalytic activity	cytochrome c oxidase subunit 6B1

IPI	GENE	Alzheimer's Hippocampus	Control hippocampus	CSF	Cellular localization	Biological process	Molecular function	Protein Description
IPI00012490.1	ATP2B4	X	X		membrane	development,transport,metabolic process,response to stimulus,coagulation	protein binding,transporter activity,metal ion binding,nucleotide binding,catalytic activity	Isoform XD of Plasma membrane calcium-transporting ATPase 4
IPI00299679.1	RGL1			X		regulation of biological process,response to stimulus,cell communication	enzyme regulator activity	Isoform B of Ral guanine nucleotide dissociation stimulator-like 1
IPI00001120.1	LINGO2			X	membrane		protein binding	Leucine-rich repeat and immunoglobulin-like domain-containing nogo receptor-interacting protein 2
IPI00028015.2	LAIR1			X	membrane			Isoform 2 of Leukocyte-associated immunoglobulin-like receptor 1
IPI00793221.1	RPP14		X		cytoplasm,cytosol	metabolic process	catalytic activity	16 kDa protein
IPI00181047.4	PNPLA8		X			metabolic process		Isoform 2 of Calcium-independent phospholipase A2-gamma
IPI00152981.1	ACAD9	X	X		mitochondrion,cytoplasm,nucleus	metabolic process	catalytic activity	Acyl-CoA dehydrogenase family member 9, mitochondrial
IPI00294911.1	SDHB	X	X		mitochondrion,membrane,cytoplasm	metabolic process,transport	protein binding,metal ion binding,catalytic activity	Succinate dehydrogenase [ubiquinone] iron-sulfur subunit, mitochondrial
IPI00021364.1	CFP			X	extracellular	metabolic process,regulation of biological process,response to stimulus,defense response		Properdin
IPI00947296.1	ROBO1	X						Protein
IPI00011578.1	NPTN			X	membrane		protein binding,receptor activity	Isoform 1 of Neuroplastin
IPI00930680.1	ABR	X	X		membrane,cytoplasm,cytosol	development,cell organization and biogenesis,transport,metabolic process,regulation of biological process,response to stimulus,cellular component movement,defense response,cell communication	protein binding,enzyme regulator activity	active breakpoint cluster region-related protein isoform c
IPI00807418.1	HELLS			X			catalytic activity	Isoform 9 of Lymphoid-specific helicase
IPI00165949.2	ERAP1	X	X	X			metal ion binding,catalytic activity	Isoform 2 of Endoplasmic reticulum aminopeptidase 1
IPI00909594.1	C7			X	membrane	response to stimulus	protein binding	cDNA FLJ58413, highly similar to Complement component C7
IPI00100154.1	TOLLIP	X	X	X	membrane,cytoplasm,cytosol	metabolic process,regulation of biological process,response to stimulus,cell communication,defense response	protein binding,signal transducer activity	Toll-interacting protein
IPI00783753.2	C15orf40			X				hypothetical protein LOC123207 isoform a
IPI00303292.2	KPNA1	X			membrane,cytoplasm,organelle lumen,cytosol,nucleus	cell death,cell organization and biogenesis,metabolic process,transport,regulation of biological process,response to stimulus,cell communication,reproduction	protein binding,transporter activity	Importin subunit alpha-1
IPI00217223.1	PAICS	X	X			metabolic process	catalytic activity	Multifunctional protein ADE2
IPI00644472.2	HDHD2			X		metabolic process	catalytic activity	Isoform 2 of Haloacid dehalogenase-like hydrolase domain-containing protein 2
IPI00376119.1	PRKACB	X	X			metabolic process	nucleotide binding,catalytic activity	Isoform 2 of cAMP-dependent protein kinase catalytic subunit beta
IPI00023845.1	KLK6			X	extracellular,membrane,mitochondrion,endoplasmic reticulum,cytoplasm,organelle lumen,nucleus	development,metabolic process,regulation of biological process,response to stimulus,cell communication,cellular homeostasis,cell differentiation	protein binding,catalytic activity	Isoform 1 of Kallikrein-6
IPI00022977.1	CKB	X	X	X	mitochondrion,cytoplasm,cytosol	development,metabolic process,cellular homeostasis	protein binding,nucleotide binding,catalytic activity	Creatine kinase B-type
IPI00301812.3	SMOC1			X	extracellular	development,regulation of biological process,response to stimulus,cell communication,cell differentiation	protein binding,metal ion binding	Isoform 1 of SPARC-related modular calcium-binding protein 1
IPI00008711.4	WFS1	X	X		membrane,endoplasmic reticulum,cytoplasm	cell death,development,transport,metabolic process,regulation of biological process,response to stimulus,cell communication,cellular homeostasis	protein binding,transporter activity	Wolframin
IPI00301317.2	CTNND2	X	X		cytoskeleton,membrane,cytoplasm,nucleus	development,metabolic process,regulation of biological process,response to stimulus,cell communication	protein binding	Isoform 1 of Catenin delta-2
IPI00003968.1	NDUFA9	X	X		membrane,mitochondrion,cytoplasm,organelle lumen	metabolic process,transport	protein binding,nucleotide binding,catalytic activity	NADH dehydrogenase [ubiquinone] 1 alpha subcomplex subunit 9, mitochondrial
IPI00306884.5	ST3GAL4			X	membrane,cytoplasm,Golgi	metabolic process	catalytic activity	cDNA FLJ11867 fis, clone HEMBA1006976, weakly similar to H.sapiens Gal-beta(1-3/1-4)GlcNAc alpha-2.3-sialyltransferase
IPI00102808.1	DUSP19			X	cytoplasm	metabolic process,regulation of biological process,response to stimulus,cell communication	protein binding,structural molecule activity,catalytic activity,enzyme regulator activity	Isoform 1 of Dual specificity protein phosphatase 19

IPI	GENE	Alzheimer's Hippocampus	Control hippocampus	CSF	Cellular localization	Biological process	Molecular function	Protein Description
IPI00829711.2	IGHA2			X	membrane		protein binding	20 kDa protein
IPI00176221.7	NEGR1	X	X	X	membrane		protein binding	Neuronal growth regulator 1
IPI00784368.1	NDST1			X	membrane,cytoplasm,Golgi	development,metabolic process,regulation of biological process,response to stimulus,defense response,cell communication	catalytic activity	Isoform 1 of Bifunctional heparan sulfate N-deacetylase/N-sulfotransferase 1
IPI00006433.6	DNAJC16	X	X		membrane	metabolic process,regulation of biological process,cellular homeostasis	protein binding	Isoform 1 of DnaJ homolog subfamily C member 16
IPI00446859.3	HEPACAM	X						Isoform 2 of Hepatocyte cell adhesion molecule
IPI00166161.2	C14orf39			X		development,metabolic process,regulation of biological process		Protein SIX6OS1
IPI00827584.1	KIF27			X	mitochondrion,membrane,cytoplasm,organelle lumen,chromosome,nucleus	cell organization and biogenesis,cellular component movement,reproduction	nucleotide binding,motor activity,catalytic activity	similar to kinesin family member 27
IPI00008603.1	ACTA2	X	X	X	cytoskeleton,cytoplasm,cytosol	development,response to stimulus,cell differentiation	nucleotide binding	Actin, aortic smooth muscle
IPI00030702.1	IDH3A	X	X		mitochondrion,cytoplasm,organelle lumen	metabolic process	metal ion binding,nucleotide binding,catalytic activity	Isoform 1 of Isocitrate dehydrogenase [NAD] subunit alpha, mitochondrial
IPI00179700.3	HMGA1	X	X		cytoplasm,organelle lumen,chromosome,cytosol,nucleus	cell proliferation,cell organization and biogenesis,metabolic process,regulation of biological process,response to stimulus,reproduction	protein binding,DNA binding,catalytic activity	Isoform HMG-I of High mobility group protein HMG-I/HMG-Y
IPI00329538.3	PRSS8			X	extracellular,membrane	metabolic process,response to stimulus	protein binding,catalytic activity	Prostasin
IPI00296537.4	FBLN1			X	extracellular		metal ion binding	Isoform C of Fibulin-1
IPI00394992.1	PGLYRP2			X		metabolic process	catalytic activity	Isoform 2 of N-acetylmuramoyl-L-alanine amidase
IPI00293303.1	LGMN			X	cytoplasm,organelle lumen,vacuole,endosome	cell death,metabolic process,regulation of biological process,response to stimulus,defense response	catalytic activity	Legumain
IPI00000005.1	NRAS	X	X		membrane,cytoplasm,Golgi	cell proliferation,cell organization and biogenesis,development,metabolic process,transport,regulation of biological process,response to stimulus,cellular component movement,cell communication,cell differentiation,coagulation	transporter activity,nucleotide binding,catalytic activity	GTPase NRas
IPI00255145.6	C16orf96			X	membrane			Putative uncharacterized protein LOC342346
IPI00030882.2	GRIA2	X	X	X	membrane,endoplasmic reticulum,cytoplasm	transport,regulation of biological process,response to stimulus,cell communication	signal transducer activity,transporter activity,receptor activity	Isoform Flop of Glutamate receptor 2
IPI00294619.2	TFG	X	X	X	cytoplasm	regulation of biological process,response to stimulus,cell communication	protein binding,signal transducer activity	Protein TFG
IPI00022462.2	TFRC			X	extracellular,cell surface,membrane,mitochondrion,cytoplasm,endosome	development,transport,metabolic process,regulation of biological process,response to stimulus,defense response,cellular homeostasis,cell differentiation	protein binding,receptor activity,catalytic activity	Transferrin receptor protein 1
IPI00384051.6	PSME2	X	X	X	proteasome			Uncharacterized protein
IPI00399254.3	OTUD4			X				Isoform 1 of OTU domain-containing protein 4
IPI00024929.2	CLMP			X	membrane		protein binding	CXADR-like membrane protein
IPI00292914.4	ANKIB1	X					protein binding,metal ion binding	Ankyrin repeat and IBR domain-containing protein 1
IPI00029235.1	IGFBP6			X	extracellular,cytoplasm,Golgi	cell proliferation,cell organization and biogenesis,regulation of biological process,response to stimulus,cell communication,cell growth	protein binding	Insulin-like growth factor-binding protein 6
IPI00984125.1	GPX3			X		metabolic process,response to stimulus	antioxidant activity,catalytic activity	Glutathione peroxidase
IPI00016608.1	TMED2	X	X		membrane,endoplasmic reticulum,cytoplasm,Golgi	development,cell organization and biogenesis,metabolic process,transport,regulation of biological process	protein binding	Transmembrane emp24 domain-containing protein 2
IPI00022989.2	RARB			X	cytoplasm,organelle lumen,nucleus	development,metabolic process,regulation of biological process,response to stimulus,cell communication	signal transducer activity,DNA binding,metal ion binding,receptor activity	Isoform Beta-1 of Retinoic acid receptor beta
IPI00004440.1	PTPRN		X	X	membrane	metabolic process,regulation of biological process,response to stimulus,cell communication	protein binding,signal transducer activity,receptor activity,catalytic activity	Receptor-type tyrosine-protein phosphatase-like N
IPI00742725.1	DHTKD1			X		metabolic process		Conserved hypothetical protein
IPI00026299.1	GYPC			X	cytoskeleton,membrane,cytoplasm		protein binding	Isoform Glycophorin-C of Glycophorin-C

IPI	GENE	Alzheimer's Hippocampus	Control hippocampus	CSF	Cellular localization	Biological process	Molecular function	Protein Description
IPI00013303.2	LSAMP	X	X	X	membrane	development	protein binding	Limbic system-associated membrane protein
IPI00784758.1	IGH@			X	membrane		protein binding	Putative uncharacterized protein DKFZp686M08189
IPI00337415.9	GNAI1	X	X		cytoskeleton,membrane,cytoplasm,nucleus	cell division,metabolic process,regulation of biological process,response to stimulus,cell communication,coagulation	protein binding,signal transducer activity,metal ion binding,nucleotide binding,catalytic activity	Guanine nucleotide-binding protein G(i) subunit alpha-1
IPI00479877.4	ALDH9A1	X	X		membrane,mitochondrion,cytoplasm,cytosol	development,metabolic process	protein binding,nucleotide binding,catalytic activity	4-trimethylaminobutyraldehyde dehydrogenase
IPI00854743.1	IGHV3-49			X				Immunglobulin heavy chain variable region
IPI00009997.1	B3GNT1	X	X	X	membrane,cytoplasm,Golgi	metabolic process	catalytic activity	N-acetyllactosaminide beta-1,3-N-acetylglucosaminyltransferase
IPI00418471.6	VIM	X	X	X	cytoskeleton,membrane,cytoplasm,cytosol	cell death,cell organization and biogenesis,metabolic process,regulation of biological process,cellular component movement	protein binding,DNA binding,motor activity,structural molecule activity,catalytic activity	Vimentin
IPI00008318.1	EPHA4	X	X	X	cytoskeleton,membrane,cytoplasm,endosome	development,cell organization and biogenesis,metabolic process,regulation of biological process,response to stimulus,cell communication,cell differentiation	protein binding,signal transducer activity,nucleotide binding,receptor activity,catalytic activity	Ephrin type-A receptor 4
IPI00029819.4	NOTCH3			X	extracellular,membrane,endoplasmic reticulum,cytoplasm,Golgi,organelle lumen,nucleus,cytosol	cell proliferation,development,metabolic process,regulation of biological process,response to stimulus,cell communication,cell differentiation	protein binding,metal ion binding,receptor activity	Neurogenic locus notch homolog protein 3
IPI00299724.3	SIRPB1			X	membrane	regulation of biological process,response to stimulus,cell communication	protein binding	Isoform 1 of Signal-regulatory protein beta-1
IPI00028509.1	GNG7	X	X		membrane	metabolic process,regulation of biological process,response to stimulus,defense response,cell communication	signal transducer activity	Guanine nucleotide-binding protein G(I)/G(S)/G(O) subunit gamma-7
IPI00293143.2	PHOX2B	X			organelle lumen,chromosome,nucleus	cell proliferation,development,cell organization and biogenesis,metabolic process,regulation of biological process,response to stimulus,cellular component movement,cell differentiation	DNA binding	Paired mesoderm homeobox protein 2B
IPI00022442.2	NDUFAB1	X	X		mitochondrion,membrane,cytoplasm,organelle lumen	transport,metabolic process	metal ion binding,catalytic activity	Acyl carrier protein, mitochondrial
IPI00017510.3	COX2	X	X		mitochondrion,membrane,cytoplasm	development,metabolic process,transport,response to stimulus,reproduction	transporter activity,metal ion binding,catalytic activity	Cytochrome c oxidase subunit 2
IPI00029700.1	DSCAM			X	extracellular,membrane	development,cell organization and biogenesis,metabolic process,regulation of biological process,response to stimulus,cellular component movement,cell growth,cell differentiation	protein binding	Isoform Long of Down syndrome cell adhesion molecule
IPI00793874.1	SFXN3	X	X		mitochondrion,membrane,cytoplasm	transport	transporter activity	sideroflexin-3
IPI00017963.1	SNRPD2	X	X		spliceosomal complex,cytoplasm,organelle lumen,cytosol,nucleus	cell organization and biogenesis,metabolic process	protein binding	Small nuclear ribonucleoprotein Sm D2
IPI00028083.1	EIF2B2		X		cytoplasm,cytosol	development,metabolic process,regulation of biological process,response to stimulus,cell communication,cellular homeostasis,reproduction,cell differentiation	protein binding,RNA binding,nucleotide binding,catalytic activity,enzyme regulator activity	Translation initiation factor eIF-2B subunit beta
IPI00180426.3	GRK4			X		metabolic process,regulation of biological process,response to stimulus,cell communication	nucleotide binding,catalytic activity	Isoform 3 of G protein-coupled receptor kinase 4
IPI00017569.5	FAIM2	X	X	X	membrane	cell death,development,regulation of biological process,cell differentiation		Fas apoptotic inhibitory molecule 2
IPI00215715.5	CAMK2A	X	X	X	membrane,cytoplasm,organelle lumen,cytosol,nucleus	metabolic process,regulation of biological process,response to stimulus,defense response,cell communication	protein binding,nucleotide binding,catalytic activity	Isoform A of Calcium/calmodulin-dependent protein kinase type II subunit alpha
IPI00793375.2	XPNPEP1	X	X		cytoplasm	metabolic process	protein binding,metal ion binding,catalytic activity	Isoform 1 of Xaa-Pro aminopeptidase 1
IPI00002134.4	PSMD5	X	X		proteasome,cytoplasm,organelle lumen,cytosol,nucleus	cell death,cell organization and biogenesis,metabolic process,regulation of biological process,response to stimulus,cell communication	protein binding	26S proteasome non-ATPase regulatory subunit 5
IPI00465028.7	TPI1	X	X	X	cytoplasm,cytosol,nucleus	development,metabolic process	catalytic activity	triosephosphate isomerase isoform 2
IPI00219525.10	PGD	X	X	X	cytoplasm,cytosol	metabolic process	nucleotide binding,catalytic activity	6-phosphogluconate dehydrogenase, decarboxylating
IPI00787827.1	PITRM1	X	X			metabolic process	metal ion binding,catalytic activity	Isoform 2 of Presequence protease, mitochondrial
IPI00396370.6	EIF3B	X	X		cytoplasm,cytosol	metabolic process,regulation of biological process	protein binding,RNA binding,nucleotide binding,structural molecule activity	Isoform 1 of Eukaryotic translation initiation factor 3 subunit B
IPI00555693.2	SPOCK3			X	extracellular	regulation of biological process,response to stimulus,cell communication	protein binding,metal ion binding	cDNA FLJ55965, highly similar to Testican-3

IPI	GENE	Alzheimer's Hippocampus	Control hippocampus	CSF	Cellular localization	Biological process	Molecular function	Protein Description
IPI00514897.3	KIAA1244		X		membrane	metabolic process,transport,regulation of biological process,response to stimulus,cell communication	enzyme regulator activity	Brefeldin A-inhibited guanine nucleotide-exchange protein 3
IPI00386755.2	ERO1L	X	X		membrane,endoplasmic reticulum,cytoplasm,organelle lumen	metabolic process,transport,regulation of biological process,response to stimulus,cell communication,cell differentiation	catalytic activity	ERO1-like protein alpha
IPI00307592.7	ABCA2			X	membrane		nucleotide binding,catalytic activity	Isoform 2 of ATP-binding cassette sub-family A member 2
IPI00033560.2	PTPRR			X	cell surface,membrane,cytoplasm,Golgi,nucleus	development,metabolic process,cell differentiation	signal transducer activity,protein binding,receptor activity,catalytic activity	Isoform Alpha of Receptor-type tyrosine-protein phosphatase R
IPI00022488.1	HPX	X	X	X	extracellular	metabolic process,transport,regulation of biological process,response to stimulus,defense response,cell communication,cellular homeostasis	protein binding,transporter activity,metal ion binding	Hemopexin
IPI00219385.3	NDUFB6		X		mitochondrion,membrane,cytoplasm,organelle lumen,nucleus	metabolic process,transport	catalytic activity	NADH dehydrogenase [ubiquinone] 1 beta subcomplex subunit 6
IPI00165665.1	XXYLT1			X			catalytic activity	Isoform 2 of Uncharacterized protein C3orf21
IPI00645667.1	MBOAT7	X	X		membrane			Isoform 3 of Lysophospholipid acyltransferase 7
IPI00027436.1	NGFR			X	extracellular,cell surface,membrane,cytoplasm,organelle lumen,cytosol,nucleus,endosome	cell death,cell proliferation,development,cell organization and biogenesis,metabolic process,regulation of biological process,response to stimulus,cell communication,cell differentiation	protein binding,signal transducer activity,receptor activity	Tumor necrosis factor receptor superfamily member 16
IPI00031485.1	MRP63			X	mitochondrion,cytoplasm,ribosome,organelle lumen	metabolic process	structural molecule activity	Ribosomal protein 63, mitochondrial
IPI00003441.1	SUCO			X	membrane,endoplasmic reticulum,cytoplasm	development		Isoform 1 of Protein osteopotentia homolog
IPI00010303.1	SERPINB4			X	extracellular,cytoplasm	metabolic process,regulation of biological process,response to stimulus	enzyme regulator activity	Serpin B4
IPI00478414.5	CHRDL1			X	extracellular	development,regulation of biological process,response to stimulus,cell communication,cell differentiation	protein binding	Isoform 1 of Chordin-like protein 1
IPI00642861.1	CXorf36			X	extracellular			Isoform 1 of UPF0672 protein CXorf36
IPI00022461.1	DYNC1I1	X	X		cytoskeleton,cytoplasm,chromosome	transport,cellular component movement	protein binding,motor activity,catalytic activity	Isoform 1 of Cytoplasmic dynein 1 intermediate chain 1
IPI00307155.8	ROCK2	X	X		cytoskeleton,mitochondrion,membrane,cytoplasm,cytosol,nucleus	cell organization and biogenesis,development,metabolic process,cell division,regulation of biological process,response to stimulus,cellular component movement,cell communication,cell differentiation	protein binding,metal ion binding,motor activity,nucleotide binding,structural molecule activity,catalytic activity	Rho-associated protein kinase 2
IPI00018342.5	AK1	X	X	X	membrane,cytoplasm,cytosol	metabolic process,regulation of biological process	nucleotide binding,catalytic activity	Adenylate kinase isoenzyme 1
IPI00021834.1	TFPI			X	extracellular,membrane	metabolic process,regulation of biological process,response to stimulus,coagulation	enzyme regulator activity	Isoform Alpha of Tissue factor pathway inhibitor
IPI00001796.2	TNFRSF18			X	membrane		protein binding,receptor activity	Tumor necrosis factor receptor superfamily, member 18 (Fragment)
IPI00103630.3	PPM1E	X	X	X			catalytic activity	Isoform 2 of Protein phosphatase 1E
IPI00384369.5	TPM1	X	X		cytoskeleton,cytoplasm	development,regulation of biological process,response to stimulus,cell communication	structural molecule activity,enzyme regulator activity	Tropomyosin 1 alpha variant 6
IPI00023001.2	FAM162A	X	X		membrane,mitochondrion,cytoplasm	cell death,cell organization and biogenesis,metabolic process,regulation of biological process,response to stimulus,cell communication	protein binding	Protein FAM162A
IPI00007764.4	HN1	X	X		nucleus			Isoform 1 of Hematological and neurological expressed 1 protein
IPI00879309.1	NRXN2			X			protein binding	Protein
IPI00152377.1	STT3B	X	X		membrane,endoplasmic reticulum,cytoplasm	metabolic process	catalytic activity	Dolichyl-diphosphooligosaccharide--protein glycosyltransferase subunit STT3B
IPI00020557.2	LRP1	X	X	X	membrane,cytoplasm,nucleus,endosome	cell proliferation,cell organization and biogenesis,development,transport,metabolic process,regulation of biological process,response to stimulus,cellular component movement,cell communication	protein binding,transporter activity,metal ion binding,receptor activity	Prolow-density lipoprotein receptor-related protein 1
IPI00166048.3	CADM3			X	membrane	cell organization and biogenesis	protein binding	Isoform 1 of Cell adhesion molecule 3
IPI00412243.5	KIAA1609	X	X				protein binding	TLD domain-containing protein KIAA1609
IPI00217146.1	SLITRK4			X	membrane		protein binding	SLIT and NTRK-like protein 4

IPI	GENE	Alzheimer's Hippocampus	Control hippocampus	CSF	Cellular localization	Biological process	Molecular function	Protein Description
IPI00329775.8	CPB2			X	extracellular	metabolic process,regulation of biological process,response to stimulus,coagulation	metal ion binding,catalytic activity	Isoform 1 of Carboxypeptidase B2
IPI00031616.6	MIEN1	X				regulation of biological process,cellular homeostasis		chromosome 17 open reading frame 37, isoform CRA_a
IPI00044607.1	PPP1R2P1			X		metabolic process,regulation of biological process,response to stimulus,cell communication	enzyme regulator activity	Putative protein phosphatase inhibitor 2-like protein 1
IPI00329593.3	ADPGK			X		metabolic process	catalytic activity	Isoform 2 of ADP-dependent glucokinase
IPI00791593.1	GLYCAM1			X				8 kDa protein
IPI00302850.4	SNRPD1	X	X	X	spliceosomal complex,cytoplasm,organelle lumen,cytosol,nucleus	cell organization and biogenesis,metabolic process	protein binding,RNA binding	Small nuclear ribonucleoprotein Sm D1
IPI00290857.3	KRT3			X	cytoskeleton	cell organization and biogenesis,cell differentiation	structural molecule activity	Keratin, type II cytoskeletal 3
IPI00027703.2	MAN2A2			X	membrane	metabolic process	metal ion binding,catalytic activity	Isoform 1 of Alpha-mannosidase 2x
IPI00013949.1	SGTA	X	X		cytoplasm		protein binding	Small glutamine-rich tetratricopeptide repeat-containing protein alpha
IPI00797699.1	PSCA			X				20 kDa protein
IPI00010903.2	DOPEY1			X	membrane,cytoplasm,Golgi	cell organization and biogenesis,transport		Uncharacterized protein
IPI01019113.1	TUBB			X	cytoskeleton,cytoplasm,cytosol,nucleus	cell death,development,cell organization and biogenesis,cell division,transport,metabolic process,regulation of biological process,response to stimulus,cellular component movement,defense response,cell differentiation	protein binding,nucleotide binding,structural molecule activity,catalytic activity	Tubulin beta chain
IPI00216298.6	TXN	X	X	X	extracellular,mitochondrion,cytoplasm,organelle lumen,cytosol,nucleus	cell proliferation,transport,metabolic process,regulation of biological process,response to stimulus,cellular component movement,defense response,cell communication,cellular homeostasis	protein binding,catalytic activity	Thioredoxin
IPI00412541.2	GPR158		X	X	membrane	regulation of biological process,response to stimulus,cell communication	signal transducer activity,receptor activity	Probable G-protein coupled receptor 158
IPI00243995.5	NEK5			X		metabolic process	metal ion binding,nucleotide binding,catalytic activity	Serine/threonine-protein kinase Nek5
IPI00013945.1	UMOD			X	extracellular,cytoskeleton,membrane,cytoplasm,Golgi	cell proliferation,development,regulation of biological process,response to stimulus,defense response	protein binding,metal ion binding	Isoform 1 of Uromodulin
IPI00894384.2	IGH@			X	membrane		protein binding	hypothetical protein
IPI00414481.7	GTF3C1			X	membrane,organelle lumen,nucleus	metabolic process	transcription regulator activity	GTF3C1 protein
IPI00903001.2	NLGN1			X		metabolic process	catalytic activity	Isoform 2 of Neuroligin-1
IPI00376229.1	PACS1		X		cytoplasm,Golgi,cytosol	cell organization and biogenesis,transport,regulation of biological process,response to stimulus,defense response	protein binding	Isoform 1 of Phosphofurin acidic cluster sorting protein 1
IPI00025363.1	GFAP	X	X	X	cytoskeleton,membrane,cytoplasm,cytosol	cell proliferation,cell organization and biogenesis,development,transport,regulation of biological process,response to stimulus,cell communication,cell differentiation	protein binding,motor activity,structural molecule activity,catalytic activity	Isoform 1 of Glial fibrillary acidic protein
IPI00219299.4	TLN2	X	X		cytoskeleton,membrane,cytoplasm	cell organization and biogenesis	protein binding,structural molecule activity	Talin-2
IPI00002186.5	ARFGEF2	X	X		membrane,cytoplasm,Golgi,cytosol	transport,regulation of biological process,response to stimulus,cell communication	protein binding,enzyme regulator activity	Brefeldin A-inhibited guanine nucleotide-exchange protein 2
IPI00294779.1	VDAC3	X	X		membrane,mitochondrion,cytoplasm	transport,regulation of biological process	transporter activity	Isoform 2 of Voltage-dependent anion-selective channel protein 3
IPI00031522.2	HADHA	X	X		mitochondrion,membrane,cytoplasm,organelle lumen,nucleus	metabolic process,response to stimulus	protein binding,nucleotide binding,catalytic activity	Trifunctional enzyme subunit alpha, mitochondrial
IPI00514806.1	S100A2	X	X				metal ion binding	Uncharacterized protein
IPI00410585.3	CRB2			X	extracellular,membrane		protein binding,metal ion binding	Isoform 1 of Crumbs homolog 2
IPI00219533.3	PMP2	X	X		cytoplasm	transport	transporter activity	Myelin P2 protein
IPI00307612.4	CDH20			X	membrane		metal ion binding	Cadherin-20
IPI00748145.2	GNAI2	X	X		cytoskeleton,membrane,cytoplasm,cytosol,nucleus	cell proliferation,metabolic process,cell division,transport,regulation of biological process,response to stimulus,cell communication,coagulation	signal transducer activity,protein binding,metal ion binding,nucleotide binding,catalytic activity	Isoform 1 of Guanine nucleotide-binding protein G(i) subunit alpha-2

IPI	GENE	Alzheimer's Hippocampus	Control hippocampus	CSF	Cellular localization	Biological process	Molecular function	Protein Description
IPI00302690.5	ACTR10	X	X		cytoskeleton,cytoplasm	cellular component movement		Actin-related protein 10
IPI00027230.3	HSP90B1	X	X	X	membrane,endoplasmic reticulum,cytoplasm,organelle lumen,cytosol	cell death,cell organization and biogenesis,transport,metabolic process,regulation of biological process,response to stimulus,defense response,cell communication,cellular homeostasis	protein binding,RNA binding,metal ion binding,nucleotide binding	Endoplasmin
IPI00008780.3	STC2			X	extracellular	development,regulation of biological process,response to stimulus,cell communication,reproduction	protein binding	Stanniocalcin-2
IPI00872550.1	PRDM2			X	nucleus	metabolic process,regulation of biological process	protein binding,DNA binding,metal ion binding	Putative uncharacterized protein PRDM2
IPI00470606.3	TRMT11		X		cytoplasm	metabolic process,regulation of biological process	DNA binding,catalytic activity	Isoform 1 of tRNA guanosine-2'-O-methyltransferase TRM11 homolog
IPI00741097.2	GSTT1	X	X		cytoplasm,cytosol	metabolic process	antioxidant activity,protein binding,catalytic activity	Glutathione S-transferase theta-1
IPI00013068.1	EIF3E		X		cytoplasm,organelle lumen,chromosome,nucleus,cytosol	metabolic process,regulation of biological process	protein binding,RNA binding	Eukaryotic translation initiation factor 3 subunit E
IPI00791134.2	CLSTN2			X	membrane		metal ion binding	unnamed protein product
IPI00062037.1	DYNLL2	X	X	X	cytoskeleton,membrane,cytoplasm,cytosol	cell death,development,transport,regulation of biological process,cell differentiation	protein binding,motor activity,catalytic activity	Dynein light chain 2, cytoplasmic
IPI00073713.3	MSI2	X			cytoplasm	cell differentiation	RNA binding,nucleotide binding	Isoform 1 of RNA-binding protein Musashi homolog 2
IPI00018434.1	TSG101	X	X		cytoskeleton,membrane,cytoplasm,organelle lumen,nucleus,endosome	cell proliferation,cell organization and biogenesis,development,transport,cell division,metabolic process,regulation of biological process,cell growth,reproduction,cell differentiation	protein binding,DNA binding	Isoform 1 of Tumor susceptibility gene 101 protein
IPI00456628.5	RLTPR	X	X			metabolic process	protein binding,catalytic activity	Leucine-rich repeat-containing protein 16C
IPI00184851.1	ST3GAL6			X	membrane,cytoplasm,Golgi	metabolic process,response to stimulus	catalytic activity	Type 2 lactosamine alpha-2,3-sialyltransferase
IPI00456670.2	PAOX			X		metabolic process		Isoform 13 of Peroxisomal N(1)-acetyl-spermine/spermidine oxidase
IPI00478997.3	IGLV4-69			X				V5-6 protein
IPI00152847.4	WFIKKN2			X	extracellular	metabolic process,regulation of biological process,response to stimulus,cell communication	protein binding,enzyme regulator activity	WAP, kazal, immunoglobulin, kunitz and NTR domain-containing protein 2
IPI00179964.5	PTBP1	X	X		organelle lumen,nucleus	metabolic process,regulation of biological process,cell differentiation	protein binding,RNA binding,nucleotide binding	Isoform 1 of Polypyrimidine tract-binding protein 1
IPI00296259.4	TMED4			X	membrane,endoplasmic reticulum,cytoplasm	transport,regulation of biological process,response to stimulus,cell communication	signal transducer activity	Isoform 1 of Transmembrane emp24 domain-containing protein 4
IPI00217465.5	HIST1H1C		X	X	chromosome,nucleus	cell organization and biogenesis,metabolic process	DNA binding	Histone H1.2
IPI00414909.1	NAGA			X	cytoplasm,vacuole	metabolic process	protein binding,catalytic activity	Alpha-N-acetylgalactosaminidase
IPI00023647.4	UBA6	X	X		cytoplasm	metabolic process	protein binding,nucleotide binding,catalytic activity	Isoform 1 of Ubiquitin-like modifier-activating enzyme 6
IPI00013272.1	GOLGA4			X	mitochondrion,membrane,cytoplasm,Golgi	transport	protein binding	Isoform 1 of Golgin subfamily A member 4
IPI00374039.5	C1orf189			X				Uncharacterized protein C1orf189
IPI00005087.1	TMOD3		X		cytoskeleton,cytoplasm		protein binding	Tropomodulin-3
IPI00004346.2	CCR10			X	membrane	regulation of biological process,response to stimulus,cell communication,cellular homeostasis	signal transducer activity,receptor activity	C-C chemokine receptor type 10
IPI00017283.2	IARS2		X		mitochondrion,cytoplasm,organelle lumen	metabolic process,regulation of biological process	nucleotide binding,catalytic activity	Isoleucyl-tRNA synthetase, mitochondrial
IPI00413324.6	RPL17	X	X		cytoplasm,ribosome,cytosol	cell organization and biogenesis,transport,metabolic process,reproduction	structural molecule activity	60S ribosomal protein L17
IPI00016610.2	PCBP1	X	X		cytoplasm,organelle lumen,nucleus	metabolic process	protein binding,DNA binding,RNA binding	Poly(rC)-binding protein 1
IPI00004047.1	EXT2			X	membrane,endoplasmic reticulum,cytoplasm,Golgi	development,metabolic process,regulation of biological process,response to stimulus,cell communication,cell differentiation	protein binding,catalytic activity	Isoform 1 of Exostosin-2
IPI00012578.1	KPNA4	X	X		cytoplasm,organelle lumen,cytosol,nucleus	transport,regulation of biological process,response to stimulus,cell communication	protein binding,transporter activity	Importin subunit alpha-4
IPI00301294.3	FAM134A			X	membrane,endoplasmic reticulum,cytoplasm			Protein FAM134A
IPI00029665.8	MMAB		X		mitochondrion,cytoplasm	metabolic process	nucleotide binding,catalytic activity	Cob(I)yrinic acid a,c-diamide adenosyltransferase, mitochondrial

IPI	GENE	Alzheimer's Hippocampus	Control hippocampus	CSF	Cellular localization	Biological process	Molecular function	Protein Description
IPI00013895.1	S100A11	X	X	X	cytoplasm,nucleus	cell proliferation,metabolic process,regulation of biological process,response to stimulus,cell communication	protein binding,metal ion binding	Protein S100-A11
IPI00289271.6	PPFIA2			X	membrane,cytoplasm	cell organization and biogenesis	protein binding	Liprin-alpha-2
IPI00045839.3	LEPRE1			X				Isoform 3 of Prolyl 3-hydroxylase 1
IPI00219315.2	GRIA3		X		membrane	transport	signal transducer activity,transporter activity,receptor activity	Isoform Flip of Glutamate receptor 3
IPI00852577.3	IGLC1			X			protein binding	Putative uncharacterized protein
IPI00010153.5	RPL23	X	X		cytoplasm,ribosome,organelle lumen,cytosol,nucleus	cell organization and biogenesis,transport,metabolic process,reproduction	structural molecule activity	60S ribosomal protein L23
IPI00744780.2	BCAS1	X	X					Isoform 2 of Breast carcinoma-amplified sequence 1
IPI00394745.3	HOMER2		X		cytoskeleton,membrane,cytoplasm	regulation of biological process,response to stimulus,cell communication	protein binding,structural molecule activity	Isoform 1 of Homer protein homolog 2
IPI00060801.1	RAB39B		X		membrane,cytoplasm,Golgi	cell organization and biogenesis,transport,regulation of biological process,response to stimulus,cell communication	nucleotide binding	Ras-related protein Rab-39B
IPI00187158.2	GPM6B	X	X		membrane			neuronal membrane glycoprotein M6-b isoform 1
IPI00168626.5	GALNTL4			X	membrane,cytoplasm,Golgi	metabolic process	catalytic activity	Isoform 1 of Putative polypeptide N-acetylgalactosaminyltransferase-like protein 4
IPI00798430.2	TF			X	extracellular	transport,cellular homeostasis	metal ion binding	17 kDa protein
IPI00642645.1	MTHFR			X		metabolic process	catalytic activity	Methylenetetrahydrofolate reductase
IPI00026795.4	ATAT1	X	X				catalytic activity	Isoform 4 of Alpha-tubulin N-acetyltransferase
IPI00059186.1	CPNE4	X	X			metabolic process,regulation of biological process	protein binding	Isoform 1 of Copine-4
IPI00479357.5	BSCL2	X	X		membrane,endoplasmic reticulum,cytoplasm			Seipin
IPI00025019.3	PSMB1	X	X	X	cytoplasm,proteasome,organelle lumen,nucleus,cytosol	cell death,metabolic process,regulation of biological process,response to stimulus,cell communication	protein binding,catalytic activity	Proteasome subunit beta type-1
IPI00935729.2	HBXIP	X	X		cytoplasm	cell death,metabolic process,regulation of biological process,response to stimulus,reproduction,cell differentiation		hepatitis B virus X-interacting protein
IPI00021290.5	ACLY	X	X		membrane,mitochondrion,cytoplasm,organelle lumen,cytosol,nucleus	metabolic process,regulation of biological process	metal ion binding,nucleotide binding,catalytic activity	ATP-citrate synthase
IPI00783313.2	PYGL	X	X	X	membrane,cytoplasm,cytosol	metabolic process	protein binding,nucleotide binding,catalytic activity	Glycogen phosphorylase, liver form
IPI00304189.6	OPTN	X			cytoplasm,Golgi	cell death,cell organization and biogenesis,transport,regulation of biological process,response to stimulus,cell communication	protein binding	Isoform 1 of Optineurin
IPI00783114.3	FAM198A			X	extracellular			Protein FAM198A
IPI00166807.4	OXR1			X		metabolic process		Isoform 3 of Oxidation resistance protein 1
IPI00328520.1	PRRT2	X	X	X	membrane	response to stimulus		Isoform 2 of Proline-rich transmembrane protein 2
IPI00019004.1	SEC62		X		cytoskeleton,membrane,endoplasmic reticulum,cytoplasm	transport	transporter activity,receptor activity	Translocation protein SEC62
IPI00009899.4	FAM13B			X	cytoplasm,cytosol	metabolic process,regulation of biological process,response to stimulus,cell communication	enzyme regulator activity	Protein FAM13B
IPI00936846.2	IGLV3-27			X				V2-19 protein
IPI00553177.1	SERPINA1	X	X	X	extracellular,cytoplasm,organelle lumen	transport,metabolic process,regulation of biological process,response to stimulus,defense response,coagulation	protein binding,enzyme regulator activity	Isoform 1 of Alpha-1-antitrypsin
IPI00006052.3	PFDN2	X	X		mitochondrion,cytoplasm,cytosol,nucleus	metabolic process	protein binding	Prefoldin subunit 2
IPI00018278.3	H2AFV	X	X		chromosome,nucleus	cell organization and biogenesis,metabolic process	DNA binding	Histone H2A.V
IPI00012315.2	NME3		X	X	mitochondrion,cytoplasm	cell death,metabolic process,regulation of biological process	metal ion binding,nucleotide binding,catalytic activity	Nucleoside diphosphate kinase 3
IPI00218429.2	PREX1	X	X					Isoform 2 of Phosphatidylinositol 3,4,5-trisphosphate-dependent Rac exchanger 1 protein
IPI00217458.3	GPT		X		cytoplasm,cytosol	metabolic process	catalytic activity	Alanine aminotransferase 1

IPI	GENE	Alzheimer's Hippocampus	Control hippocampus	CSF	Cellular localization	Biological process	Molecular function	Protein Description
IPI00478816.3	SPINK5			X	extracellular,membrane,endoplasmic reticulum,cytoplasm,cytosol	cell organization and biogenesis,development,metabolic process,regulation of biological process,response to stimulus,cell differentiation	protein binding,enzyme regulator activity	Isoform f-l of Serine protease inhibitor Kazal-type 5
IPI00830045.2	LILRB4			X				Isoform 2 of Leukocyte immunoglobulin-like receptor subfamily B member 4
IPI00020965.1	UBE2H	X				metabolic process	protein binding,nucleotide binding,catalytic activity	Ubiquitin-conjugating enzyme E2 H
IPI00220770.2	DCTN6	X	X		cytoskeleton,cytoplasm		protein binding,catalytic activity	Dynactin subunit 6
IPI00419258.4	HMGB1	X	X		extracellular,cell surface,cytoplasm,organelle lumen,chromosome,nucleus	cell death,cell proliferation,cell organization and biogenesis,development,metabolic process,regulation of biological process,response to stimulus,cellular component movement,defense response,cell differentiation	protein binding,DNA binding,RNA binding	High mobility group protein B1
IPI00000832.1	PDYN			X	extracellular,membrane	cell death,regulation of biological process,response to stimulus,cell communication	protein binding	Proenkephalin-B
IPI00011676.2	ASB15	X	X		cytoskeleton,membrane,cytoplasm,Golgi,organelle lumen,cytosol,nucleus	development,cell organization and biogenesis,transport,metabolic process,regulation of biological process,response to stimulus,cellular component movement,cell differentiation	protein binding,enzyme regulator activity	Neural Wiskott-Aldrich syndrome protein
IPI00644231.3	CYFIP1		X	X	membrane,cytoplasm	cell organization and biogenesis,development,regulation of biological process,cell growth,cell differentiation	protein binding	Isoform 1 of Cytoplasmic FMR1-interacting protein 1
IPI00009268.2	ABHD14A-ACY1	X	X		cytoplasm	metabolic process	catalytic activity	cDNA FLJ60317, highly similar to Aminoacylase-1
IPI00784969.1	LOC100126583			X			protein binding	LOC100126583 protein
IPI00014852.2	PGM5	X	X		cytoskeleton,membrane,cytoplasm,cytosol	metabolic process	metal ion binding,structural molecule activity,catalytic activity	Isoform 1 of Phosphoglucomutase-like protein 5
IPI00010271.3	RAC1	X	X		membrane,cytoplasm,cytosol	cell death,cell organization and biogenesis,development,transport,metabolic process,regulation of biological process,response to stimulus,cellular component movement,defense response,cell communication,cell differentiation,coagulation	protein binding,signal transducer activity,nucleotide binding,receptor activity,catalytic activity	Isoform A of Ras-related C3 botulinum toxin substrate 1
IPI00013881.6	HNRNPH1	X	X		spliceosomal complex,cytoskeleton,cytoplasm,organelle lumen,nucleus	metabolic process,regulation of biological process	protein binding,RNA binding,nucleotide binding	Heterogeneous nuclear ribonucleoprotein H
IPI00021440.1	ACTG1	X	X		cytoskeleton,cytoplasm,cytosol	cell organization and biogenesis,development,response to stimulus,cellular component movement,cell differentiation,coagulation	protein binding,nucleotide binding,structural molecule activity	Actin, cytoplasmic 2
IPI00911047.1	CHGB		X	X	cytoplasm			cDNA FLJ58131, highly similar to Secretogranin-1
IPI00300562.2	RAB3B	X	X		membrane,cytoplasm,cytosol	metabolic process,transport,regulation of biological process,response to stimulus,cell communication	nucleotide binding,catalytic activity	Ras-related protein Rab-3B
IPI00395866.2	SCUBE1			X			protein binding,metal ion binding	Uncharacterized protein
IPI00215719.6	RPL18	X	X		cytoplasm,ribosome,cytosol	cell organization and biogenesis,transport,metabolic process,reproduction	RNA binding,structural molecule activity	60S ribosomal protein L18
IPI00027701.3	ACADS	X	X		mitochondrion,cytoplasm,organelle lumen	cell organization and biogenesis,metabolic process,response to stimulus	catalytic activity	Short-chain specific acyl-CoA dehydrogenase, mitochondrial
IPI00607580.2	MEGF8			X			metal ion binding	Isoform 2 of Multiple epidermal growth factor-like domains protein 8
IPI00008422.6	SMARCAD1			X			DNA binding,nucleotide binding,catalytic activity	Isoform 2 of SWI/SNF-related matrix-associated actin-dependent regulator of chromatin subfamily A containing DEAD/H box 1
IPI00004968.1	PRPF19	X	X		spliceosomal complex,cytoskeleton,cytoplasm,organelle lumen,nucleus	cell proliferation,development,cell organization and biogenesis,metabolic process,regulation of biological process,response to stimulus,cell differentiation	protein binding,DNA binding,catalytic activity	Pre-mRNA-processing factor 19
IPI00026216.4	NPEPPS	X	X	X	cytoplasm,cytosol,nucleus	metabolic process,response to stimulus	metal ion binding,catalytic activity	Puromycin-sensitive aminopeptidase
IPI00479361.4	B3GNT4			X	membrane,cytoplasm,Golgi	metabolic process	catalytic activity	Isoform 1 of UDP-GlcNAc:betaGal beta-1,3-N-acetylglucosaminyltransferase 4
IPI00218829.9	GSPT1	X	X			metabolic process	protein binding,RNA binding,nucleotide binding,catalytic activity	Eukaryotic peptide chain release factor GTP-binding subunit ERF3A
IPI00295857.7	COPA	X	X		extracellular,membrane,cytoplasm,Golgi,cytosol	cell organization and biogenesis,transport	protein binding,structural molecule activity	Isoform 1 of Coatomer subunit alpha
IPI00009949.2	PSMF1	X	X		cytoplasm,proteasome,organelle lumen,cytosol,nucleus	cell death,metabolic process,regulation of biological process,response to stimulus,cell communication	protein binding,enzyme regulator activity	Proteasome inhibitor PI31 subunit

IPI	GENE	Alzheimer's Hippocampus	Control hippocampus	CSF	Cellular localization	Biological process	Molecular function	Protein Description
IPI00413912.2	TMEM132E			X	membrane			Transmembrane protein 132E
IPI00003925.6	PDHB	X	X		mitochondrion,cytoplasm,organelle lumen	metabolic process,regulation of biological process	catalytic activity	Isoform 1 of Pyruvate dehydrogenase E1 component subunit beta, mitochondrial
IPI00854841.1	IGHV3-48			X				Myosin-reactive immunoglobulin heavy chain variable region
IPI00003870.1	CLPP	X	X		mitochondrion,cytoplasm,organelle lumen	metabolic process	protein binding,nucleotide binding,catalytic activity	Putative ATP-dependent Clp protease proteolytic subunit, mitochondrial
IPI00478521.5	RILPL1		X	X	membrane,cytoplasm,organelle lumen,cytosol,nucleus			Isoform 1 of RILP-like protein 1
IPI00465361.4	RPL13	X	X		cytoplasm,ribosome,cytosol	cell organization and biogenesis,metabolic process,transport,reproduction	protein binding,RNA binding,structural molecule activity	60S ribosomal protein L13
IPI00745872.2	ALB	X	X	X	extracellular,cytoplasm,organelle lumen	cell death,metabolic process,transport,regulation of biological process,response to stimulus,cell communication,coagulation	antioxidant activity,protein binding,DNA binding,metal ion binding,catalytic activity	Isoform 1 of Serum albumin
IPI00396171.4	MAP4	X	X		cytoskeleton,membrane,cytoplasm		protein binding,structural molecule activity	Isoform 1 of Microtubule-associated protein 4
IPI00018026.3	BSDC1		X					Isoform 3 of BSD domain-containing protein 1
IPI00029730.1	STX4		X		cell surface,membrane,cytoplasm,vacuole,cytosol	cell organization and biogenesis,transport,response to stimulus,cell communication,coagulation	protein binding,transporter activity	Syntaxin-4
IPI00006579.1	COX4I1	X	X		membrane,mitochondrion,cytoplasm,nucleus	metabolic process,response to stimulus	transporter activity,catalytic activity	Cytochrome c oxidase subunit 4 isoform 1, mitochondrial
IPI00009123.2	NUCB2			X	extracellular,membrane,cytoplasm,Golgi,cytosol		protein binding,DNA binding,metal ion binding	Isoform 1 of Nucleobindin-2
IPI00028030.4	COMP			X	extracellular	cell death,development,regulation of biological process	protein binding,metal ion binding,structural molecule activity	Cartilage oligomeric matrix protein
IPI00293460.5	ABCA1			X	membrane,cytoplasm,Golgi	cell organization and biogenesis,transport,metabolic process,regulation of biological process,response to stimulus,cell communication,cell differentiation	signal transducer activity,protein binding,transporter activity,nucleotide binding,receptor activity,catalytic activity	ATP-binding cassette sub-family A member 1
IPI00017375.2	SEC23A	X	X		membrane,endoplasmic reticulum,cytoplasm,Golgi,cytosol	cell organization and biogenesis,transport,metabolic process	protein binding,metal ion binding	Protein transport protein Sec23A
IPI00741780.2	LOC652559			X				similar to CG4845-PA
IPI00006196.3	NUMA1	X	X				nucleotide binding	Isoform 2 of Nuclear mitotic apparatus protein 1
IPI00031564.1	GGCT	X	X	X	cytoplasm,cytosol	cell death,cell organization and biogenesis,metabolic process,regulation of biological process,response to stimulus,cell communication	protein binding,catalytic activity	Isoform 1 of Gamma-glutamylcyclotransferase
IPI00019907.1	GPC3			X	extracellular,membrane	cell proliferation,development,metabolic process,regulation of biological process	protein binding,enzyme regulator activity	Glypican-3
IPI00029403.1	SNX4		X		cytoskeleton,membrane,cytoplasm,endosome	transport,cell communication	protein binding	Sorting nexin-4
IPI00010368.6	KIF2A		X		cytoskeleton	cellular component movement	motor activity,nucleotide binding,catalytic activity	Uncharacterized protein
IPI00018522.5	PRMT1	X	X		cytoplasm,cytosol,nucleus	cell organization and biogenesis,development,metabolic process,regulation of biological process,response to stimulus,cell communication,cell differentiation	protein binding,catalytic activity	Protein arginine N-methyltransferase 1
IPI00797766.1	MPRIP		X		membrane	transport		KIAA0864 protein
IPI00014307.4	MTMR2	X	X		cytoskeleton,membrane,cytoplasm,vacuole,nucleus,cytosol,endosome	cell organization and biogenesis,transport,metabolic process,regulation of biological process,cell communication,cellular homeostasis	protein binding,catalytic activity	Myotubularin-related protein 2
IPI00031812.3	YBX1	X	X		extracellular,spliceosomal complex,cytoplasm,organelle lumen,nucleus	development,cell division,metabolic process,regulation of biological process,cell differentiation	protein binding,DNA binding,RNA binding	Nuclease-sensitive element-binding protein 1
IPI00032875.2	ETFDH	X	X		mitochondrion,membrane,cytoplasm,organelle lumen	metabolic process,transport,response to stimulus	metal ion binding,catalytic activity	Electron transfer flavoprotein-ubiquinone oxidoreductase, mitochondrial
IPI00152849.2	CCNB3			X	organelle lumen,nucleus	cell division,metabolic process,regulation of biological process,reproduction	protein binding	Isoform 1 of G2/mitotic-specific cyclin-B3
IPI00003807.7	ACP2		X	X	membrane,cytoplasm,organelle lumen,vacuole	cell death,development,cell organization and biogenesis,response to stimulus	protein binding,catalytic activity	Lysosomal acid phosphatase
IPI00456827.5	FAM22G			X	extracellular			Isoform 1 of Protein FAM22G
IPI00179026.2	MCTS1	X	X		cytoplasm	cell proliferation,metabolic process,regulation of biological process,response to stimulus	RNA binding	Isoform 1 of Malignant T cell-amplified sequence 1
IPI00300584.1	EPN3		X		cytoplasm,nucleus			Isoform 1 of Epsin-3
IPI00012795.3	EIF3I	X	X		cytoplasm,cytosol	metabolic process	protein binding,RNA binding	Eukaryotic translation initiation factor 3 subunit I

IPI	GENE	Alzheimer's Hippocampus	Control hippocampus	CSF	Cellular localization	Biological process	Molecular function	Protein Description
IPI00873766.2	IMPG2			X	extracellular,cell surface,membrane		protein binding,receptor activity,structural molecule activity	Interphotoreceptor matrix proteoglycan 2
IPI00289058.3	LYNX1		X	X	membrane			Ly-6/neurotoxin-like protein 1
IPI00294955.3	LSM4	X			cytoplasm,cytosol,nucleus	metabolic process	protein binding,RNA binding	U6 snRNA-associated Sm-like protein LSm4
IPI00024273.1	VLDLR			X	extracellular,cell surface,membrane,cytoplasm,nucleus	development,cell organization and biogenesis,metabolic process,transport,regulation of biological process,response to stimulus,cell communication,cell differentiation	protein binding,signal transducer activity,transporter activity,metal ion binding,receptor activity	Isoform Long of Very low-density lipoprotein receptor
IPI00009950.1	LMAN2	X		X	membrane,endoplasmic reticulum,cytoplasm,Golgi	transport	metal ion binding	Vesicular integral-membrane protein VIP36
IPI00010845.3	NDUFS8	X	X		membrane,mitochondrion,cytoplasm	cell organization and biogenesis,transport,metabolic process,response to stimulus	metal ion binding,catalytic activity	NADH dehydrogenase [ubiquinone] iron-sulfur protein 8, mitochondrial
IPI00829767.1	IGHG2			X			protein binding	Protein
IPI00791534.2	SLC4A1	X	X		cytoskeleton,membrane,cytoplasm	transport	protein binding,transporter activity	Solute carrier family 4, anion exchanger, member 1
IPI00011732.2	GFRA2			X	membrane	regulation of biological process,response to stimulus,cell communication	signal transducer activity,receptor activity	Isoform 1 of GDNF family receptor alpha-2
IPI00046828.4	CCDC58		X		mitochondrion,cytoplasm			Coiled-coil domain-containing protein 58
IPI00008454.1	DNAJB11		X		endoplasmic reticulum,cytoplasm,organelle lumen,nucleus	metabolic process,regulation of biological process,response to stimulus,cell communication	protein binding	DnaJ homolog subfamily B member 11
IPI00063048.6	ST6GAL2			X	membrane,cytoplasm,Golgi	metabolic process	catalytic activity	Isoform 2 of Beta-galactoside alpha-2,6-sialyltransferase 2
IPI00215651.1	GUCY1B3	X	X			metabolic process,regulation of biological process,response to stimulus,cell communication	metal ion binding,catalytic activity	Isoform HSGC-2 of Guanylate cyclase soluble subunit beta-1
IPI00550162.4	IGLV3-25			X				V2-17 protein
IPI00015944.5	EML2	X	X		cytoskeleton,cytoplasm	metabolic process	protein binding,catalytic activity	Echinoderm microtubule-associated protein-like 2
IPI00556376.2	CRMP1	X	X				catalytic activity	dihydropyrimidinase-related protein 1 isoform 1
IPI00020567.3	ARHGAP1	X	X		membrane,cytoplasm,cytosol	metabolic process,regulation of biological process,response to stimulus,cell communication	protein binding,enzyme regulator activity	Rho GTPase-activating protein 1
IPI00029605.1	GALNS			X	cytoplasm,vacuole	metabolic process	metal ion binding,catalytic activity	N-acetylgalactosamine-6-sulfatase
IPI00107731.5	OSCAR			X	membrane		signal transducer activity	Isoform 6 of Osteoclast-associated immunoglobulin-like receptor
IPI00007750.1	TUBA4A	X	X	X	extracellular,cytoskeleton,cytoplasm,cytosol	cell organization and biogenesis,transport,metabolic process,response to stimulus,cellular component movement,coagulation	nucleotide binding,structural molecule activity,catalytic activity	Tubulin alpha-4A chain
IPI00020508.1	TRMT1	X	X			metabolic process	RNA binding,metal ion binding,catalytic activity	Isoform 1 of N(2),N(2)-dimethylguanosine tRNA methyltransferase
IPI00642256.2	CAPZB	X	X		cytoskeleton,cytoplasm	cell organization and biogenesis	protein binding	Isoform 2 of F-actin-capping protein subunit beta
IPI00024094.4	RHAG			X	membrane	transport,cellular homeostasis	protein binding,transporter activity	Ammonium transporter Rh type A
IPI00217987.8	ITGAM			X	membrane	regulation of biological process,response to stimulus,cellular component movement,cell communication,coagulation	protein binding,receptor activity	Integrin alpha-M
IPI00243338.4	KRT23			X			structural molecule activity	cDNA, FLJ95443, highly similar to Homo sapiens keratin 23 (histone deacetylase inducible) (KRT23), transcript variant 2, mRNA
IPI00290553.2	ALDH1L1	X	X		mitochondrion,cytoplasm	metabolic process	catalytic activity	Aldehyde dehydrogenase family 1 member L1
IPI00217920.7	ALDH16A1	X	X			metabolic process	catalytic activity	Isoform 1 of Aldehyde dehydrogenase family 16 member A1
IPI00465363.3	HIST1H2BA			X	membrane,chromosome,nucleus	cell organization and biogenesis,metabolic process,response to stimulus,cellular component movement,defense response	DNA binding	Histone H2B type 1-A
IPI00030075.1	FGL2			X	extracellular,membrane	regulation of biological process,response to stimulus,cell communication	protein binding	Fibroleukin
IPI00031121.2	CPE	X	X	X		metabolic process	metal ion binding,catalytic activity	Carboxypeptidase E precursor
IPI00303318.2	FAM49B	X	X	X				Protein FAM49B
IPI00004533.1	KIF3B	X	X	X	cytoskeleton,cytoplasm,cytosol	development,cell organization and biogenesis,transport,response to stimulus,cellular component movement,coagulation	protein binding,motor activity,nucleotide binding,catalytic activity	Kinesin-like protein KIF3B

IPI	GENE	Alzheimer's Hippocampus	Control hippocampus	CSF	Cellular localization	Biological process	Molecular function	Protein Description
IPI00293849.4	PTPRM			X	membrane,cytoplasm	cell proliferation,development,cell organization and biogenesis,metabolic process,regulation of biological process,response to stimulus,cellular component movement,cell communication,cell differentiation	protein binding,signal transducer activity,receptor activity,catalytic activity	Receptor-type tyrosine-protein phosphatase mu
IPI00003448.1	MIA			X	extracellular	cell proliferation,cell organization and biogenesis	protein binding	Melanoma-derived growth regulatory protein
IPI00005491.4	CLSTN2			X	membrane,endoplasmic reticulum,cytoplasm,Golgi		metal ion binding	Calsyntenin-2
IPI00220002.3	PALM	X			membrane	cell organization and biogenesis,regulation of biological process		Isoform 2 of Paralemmin-1
IPI00216921.1	STMN4			X		regulation of biological process,response to stimulus,cell communication		Isoform 2 of Stathmin-4
IPI00152303.8	PIP4K2C	X			membrane,cytoplasm,organelle lumen,nucleus	metabolic process	protein binding,nucleotide binding,catalytic activity	Phosphatidylinositol-5-phosphate 4-kinase type-2 gamma
IPI00018873.1	NAMPT	X	X		cytoplasm,cytosol	cell proliferation,development,metabolic process,regulation of biological process,response to stimulus,cell communication	protein binding,catalytic activity	Nicotinamide phosphoribosyltransferase
IPI00386754.4	CRELD2			X				Isoform 2 of Cysteine-rich with EGF-like domain protein 2
IPI00299254.4	EIF5B		X		cytoplasm,cytosol	metabolic process,regulation of biological process	protein binding,RNA binding,nucleotide binding,catalytic activity	Eukaryotic translation initiation factor 5B
IPI00939270.1	CYAT1			X			protein binding	V2-14 protein
IPI00549330.7	CDKL4			X			protein binding	Similar to Ig kappa chain V-IV region JI precursor
IPI00438286.4	ERBB2IP	X	X	X	extracellular,membrane,cytoplasm,nucleus	cell organization and biogenesis,regulation of biological process,response to stimulus,cell communication,cell growth	protein binding,structural molecule activity	Isoform 1 of Protein LAP2
IPI00022822.5	COL18A1	X	X	X			structural molecule activity	Isoform 2 of Collagen alpha-1(XVIII) chain
IPI00029015.1	KDSR	X	X		extracellular,membrane,endoplasmic reticulum,cytoplasm	metabolic process	nucleotide binding,catalytic activity	3-ketodihydrosphingosine reductase
IPI00000783.2	PSMB8		X		cytoplasm,proteasome,organelle lumen,nucleus,cytosol	cell death,metabolic process,regulation of biological process,response to stimulus,cell communication,defense response,cell differentiation	catalytic activity	Isoform 1 of Proteasome subunit beta type-8
IPI00024990.6	ALDH6A1	X	X		mitochondrion,cytoplasm,organelle lumen,nucleus	metabolic process	catalytic activity	Methylmalonate-semialdehyde dehydrogenase [acylating], mitochondrial
IPI00215894.1	KNG1			X			enzyme regulator activity	Isoform LMW of Kininogen-1
IPI00002412.1	PPT1	X	X	X	extracellular,membrane,cytoplasm,Golgi,vacuole,cytosol,nucleus	cell death,cell organization and biogenesis,development,transport,metabolic process,regulation of biological process,response to stimulus,cell communication,cellular homeostasis,cell differentiation,cell growth	catalytic activity	Palmitoyl-protein thioesterase 1
IPI00023625.1	GNG3	X	X		membrane	metabolic process,regulation of biological process,response to stimulus,cell communication,cellular homeostasis	signal transducer activity,protein binding,catalytic activity	Guanine nucleotide-binding protein G(I)/G(S)/G(O) subunit gamma-3
IPI00295940.4	SUN2	X	X		nucleus			cDNA FLJ55508, highly similar to Sad1/unc-84-like protein 2
IPI00012497.2	ADRBK1	X	X		membrane,cytoplasm,cytosol	development,transport,metabolic process,regulation of biological process,response to stimulus,cell communication,reproduction	protein binding,nucleotide binding,catalytic activity	Beta-adrenergic receptor kinase 1
IPI00023184.6	PARP3			X	cytoskeleton,cytoplasm,nucleus	metabolic process,response to stimulus	catalytic activity	Isoform 1 of Poly [ADP-ribose] polymerase 3
IPI00022958.1	C16orf72			X	membrane,mitochondrion,cytoplasm	metabolic process	metal ion binding,catalytic activity	PRO0149
IPI00337548.6	CGREF1			X	extracellular	cell proliferation,regulation of biological process,response to stimulus	metal ion binding	Cell growth regulator with EF hand domain protein 1
IPI00871533.2	PP2D1			X		metabolic process	catalytic activity	Isoform 1 of Protein phosphatase 2C-like domain-containing protein 1
IPI00909283.1	CDH11			X	membrane		metal ion binding	cDNA FLJ58514, highly similar to Cadherin-11
IPI00879084.2	CP			X			metal ion binding	Uncharacterized protein
IPI00216308.5	VDAC1	X	X		mitochondrion,membrane,cytoplasm,organelle lumen	cell death,transport,regulation of biological process,response to stimulus,cell communication,defense response	protein binding,transporter activity	Voltage-dependent anion-selective channel protein 1
IPI00736885.1	LOC440786			X	extracellular,membrane	metabolic process,regulation of biological process,response to stimulus,defense response	protein binding	Ig kappa chain V-II region TEW

IPI	GENE	Alzheimer's Hippocampus	Control hippocampus	CSF	Cellular localization	Biological process	Molecular function	Protein Description
IPI00032460.3	LSM2	X	X		spliceosomal complex,cytoplasm,organelle lumen,cytosol,nucleus	metabolic process	protein binding,RNA binding	U6 snRNA-associated Sm-like protein LSm2
IPI00328298.6	SMC4			X	chromosome	cell organization and biogenesis	protein binding,nucleotide binding	Isoform 2 of Structural maintenance of chromosomes protein 4
IPI00943305.2	PTPRB			X	membrane	development,metabolic process	signal transducer activity,protein binding,receptor activity,catalytic activity	Isoform 1 of Receptor-type tyrosine-protein phosphatase beta
IPI00005966.6	NDUFA12	X	X		membrane		catalytic activity	13kDa differentiation-associated protein variant (Fragment)
IPI00176398.2	SLITRK6			X	membrane	cell organization and biogenesis,development,cell differentiation	protein binding	SLIT and NTRK-like protein 6
IPI00001611.1	IGF2			X	extracellular	cell proliferation,development,cell organization and biogenesis,metabolic process,cell division,regulation of biological process,response to stimulus,cell communication	protein binding,enzyme regulator activity	Isoform 1 of Insulin-like growth factor II
IPI00292228.1	GSK3A	X	X		cytoplasm,cytosol	development,cell organization and biogenesis,transport,metabolic process,regulation of biological process,response to stimulus,cellular component movement,cell communication,cell differentiation,cell growth	protein binding,nucleotide binding,catalytic activity	Glycogen synthase kinase-3 alpha
IPI00026358.3	GABARAPL2	X	X	X	membrane,cytoplasm,Golgi,vacuole,cytosol	cell organization and biogenesis,transport,metabolic process,regulation of biological process,response to stimulus,cell communication	protein binding,receptor activity	Gamma-aminobutyric acid receptor-associated protein-like 2
IPI00328753.1	KTN1	X	X		membrane,endoplasmic reticulum,cytoplasm	cellular component movement	protein binding	Isoform 1 of Kinectin
IPI01015859.1	CACNA2D1			X			protein binding	Isoform 2 of Voltage-dependent calcium channel subunit alpha-2/delta-1
IPI00026942.5	ERLIN2	X	X		membrane,endoplasmic reticulum,cytoplasm	cell death,metabolic process	protein binding	Isoform 1 of Erlin-2
IPI00607655.4	EPHA7			X		metabolic process,regulation of biological process,response to stimulus,cell communication	protein binding,nucleotide binding,catalytic activity	Isoform 2 of Ephrin type-A receptor 7
IPI00005724.1	LANCL1	X	X		cytoskeleton,membrane,cytoplasm,nucleus	metabolic process,regulation of biological process,response to stimulus,cell communication	signal transducer activity,protein binding,metal ion binding,receptor activity,catalytic activity	LanC-like protein 1
IPI00008214.4	NDUFV3	X	X		membrane,mitochondrion,cytoplasm,organelle lumen,nucleus	transport,metabolic process	catalytic activity	Isoform 1 of NADH dehydrogenase [ubiquinone] flavoprotein 3, mitochondrial
IPI00020906.1	IMPA1	X	X	X	mitochondrion,cytoplasm,organelle lumen,nucleus	metabolic process,regulation of biological process,response to stimulus,cell communication	protein binding,metal ion binding,catalytic activity	Inositol monophosphatase 1
IPI00872762.2	SUCLG1	X	X		mitochondrion,membrane,cytoplasm,organelle lumen	metabolic process	protein binding,nucleotide binding,catalytic activity	Succinyl-CoA ligase [GDP-forming] subunit alpha, mitochondrial
IPI00220219.6	COPB2	X	X		cytoskeleton,membrane,cytoplasm,Golgi,cytosol	cell organization and biogenesis,transport	protein binding,structural molecule activity	Coatomer subunit beta'
IPI00021857.1	APOC3		X	X	extracellular	cell organization and biogenesis,metabolic process,transport,regulation of biological process,response to stimulus,cell communication	protein binding,enzyme regulator activity	Apolipoprotein C-III
IPI00790856.2	NME2	X				metabolic process	nucleotide binding,catalytic activity	Nucleoside diphosphate kinase
IPI00014240.8	MLC1	X	X		membrane,endoplasmic reticulum,cytoplasm,ribosome,vacuole,endosome	cell organization and biogenesis,transport,metabolic process,regulation of biological process,response to stimulus	protein binding,transporter activity,structural molecule activity	Membrane protein MLC1
IPI00024966.1	CNTN2	X	X	X	cell surface,membrane	development,cell organization and biogenesis,transport,metabolic process,regulation of biological process,response to stimulus,cellular component movement,cell communication,cellular homeostasis,cell differentiation	protein binding	Contactin-2
IPI00017451.1	SF3A1	X	X		spliceosomal complex,organelle lumen,nucleus	cell organization and biogenesis,metabolic process	protein binding,RNA binding	Splicing factor 3A subunit 1
IPI00017964.1	SNRPD3	X	X	X	spliceosomal complex,cytoplasm,organelle lumen,nucleus,cytosol	cell organization and biogenesis,metabolic process	protein binding,RNA binding	Small nuclear ribonucleoprotein Sm D3
IPI00306332.4	RPL24		X	X	cytoplasm,ribosome,cytosol	cell organization and biogenesis,metabolic process,transport,reproduction	RNA binding,structural molecule activity	60S ribosomal protein L24
IPI00015475.3	SLC1A1		X		membrane	cell organization and biogenesis,transport,cell communication	transporter activity	Excitatory amino acid transporter 3
IPI00019276.1	UBXN6	X	X		cytoskeleton,cytoplasm,organelle lumen,nucleus		protein binding	Isoform 1 of UBX domain-containing protein 6
IPI00787020.1	DNM1P31			X			nucleotide binding,catalytic activity	similar to hCG1743199
IPI00178854.1	CNTN4			X	extracellular,membrane	development,cell organization and biogenesis,regulation of biological process,response to stimulus,cell communication,cell differentiation	protein binding	Isoform 1 of Contactin-4
IPI00296558.2	CPXM2			X	extracellular	metabolic process	metal ion binding,catalytic activity	Inactive carboxypeptidase-like protein X2

IPI	GENE	Alzheimer's Hippocampus	Control hippocampus	CSF	Cellular localization	Biological process	Molecular function	Protein Description
IPI00385007.5	AP1B1			X	membrane,cytoplasm	transport		Putative uncharacterized protein DKFZp686A01208
IPI00385562.1	QKI	X	X				RNA binding	Isoform 4 of Protein quaking
IPI00006211.4	VAPB	X	X		membrane,endoplasmic reticulum,cytoplasm,Golgi	cell death,metabolic process,regulation of biological process,response to stimulus,cell communication,cellular homeostasis,reproduction	protein binding,structural molecule activity	Isoform 1 of Vesicle-associated membrane protein-associated protein B/C
IPI00012503.1	PSAP	X	X	X	extracellular,membrane,mitochondrion,cytoplasm,Golgi,vacuole,organelle lumen	development,transport,metabolic process,regulation of biological process,response to stimulus,cell communication,reproduction,cell differentiation,coagulation	enzyme regulator activity	Isoform Sap-mu-0 of Proactivator polypeptide
IPI00217258.6	CEP120			X				Isoform 3 of Centrosomal protein of 120 kDa
IPI00398700.3	GNAO1	X	X			regulation of biological process,response to stimulus,cell communication	signal transducer activity,nucleotide binding	Isoform Alpha-2 of Guanine nucleotide-binding protein G(o) subunit alpha
IPI00028438.4	CMIP		X		cytoplasm,nucleus		protein binding	C-Maf-inducing protein isoform C-mip
IPI00305626.5	ASPHD1		X		membrane,endoplasmic reticulum,cytoplasm	metabolic process	catalytic activity	Aspartate beta-hydroxylase domain-containing protein 1
IPI00020042.2	PSMC4	X	X		mitochondrion,proteasome,cytoplasm,organelle lumen,cytosol,nucleus	cell death,development,metabolic process,regulation of biological process,response to stimulus,cell communication,reproduction	protein binding,RNA binding,nucleotide binding,catalytic activity	Isoform 1 of 26S protease regulatory subunit 6B
IPI00220906.6	ACOT2	X	X		mitochondrion,cytoplasm	metabolic process	protein binding,catalytic activity	Isoform 1 of Acyl-coenzyme A thioesterase 2, mitochondrial
IPI00012268.3	PSMD2	X	X		cytoplasm,proteasome,organelle lumen,cytosol,nucleus	cell death,metabolic process,regulation of biological process,response to stimulus,cell communication	protein binding,enzyme regulator activity	26S proteasome non-ATPase regulatory subunit 2
IPI00644766.5	TOR1AIP1			X				Torsin A interacting protein 1
IPI00554521.2	FTH1			X	mitochondrion,cytoplasm,cytosol	cell death,cell proliferation,cell organization and biogenesis,metabolic process,transport,regulation of biological process,response to stimulus,cellular homeostasis	protein binding,metal ion binding,catalytic activity	Ferritin heavy chain
IPI00024623.3	ACADSB	X	X		mitochondrion,cytoplasm,organelle lumen	metabolic process	catalytic activity	Short/branched chain specific acyl-CoA dehydrogenase, mitochondrial
IPI00465431.8	LGALS3	X	X		extracellular,spliceosomal complex,membrane,mitochondrion,cytoplasm,nucleus	cell organization and biogenesis,development,metabolic process,response to stimulus,defense response,cell differentiation	protein binding	Galectin-3
IPI00400935.5	COL16A1			X	extracellular	regulation of biological process,response to stimulus,cell communication,reproduction	protein binding	Isoform 1 of Collagen alpha-1(XVI) chain
IPI00029693.2	NRP2		X	X	membrane	cell proliferation,development,cell organization and biogenesis,regulation of biological process,response to stimulus,cellular component movement,cell communication,cell differentiation	protein binding,signal transducer activity,metal ion binding,receptor activity,catalytic activity	Isoform A22 of Neuropilin-2
IPI00646689.1	TXNDC17	X	X	X	cytoplasm,cytosol	regulation of biological process,response to stimulus,cell communication	antioxidant activity,protein binding,catalytic activity	Thioredoxin domain-containing protein 17
IPI00513845.2	FSD1L		X				protein binding	Isoform 2 of FSD1-like protein
IPI00018534.4	HIST1H2BL			X	chromosome,nucleus	cell organization and biogenesis,metabolic process	DNA binding	Histone H2B type 1-L
IPI00025622.3	ZFAND5			X	cytoplasm	development,regulation of biological process,response to stimulus,cellular component movement,cell communication	DNA binding,metal ion binding	AN1-type zinc finger protein 5
IPI00748998.1	SCFV			X			protein binding	Single-chain Fv (Fragment)
IPI00027466.1	CA4			X	cell surface,membrane,endoplasmic reticulum,cytoplasm,Golgi	development,transport,metabolic process,response to stimulus	protein binding,metal ion binding,catalytic activity	Carbonic anhydrase 4
IPI00250297.3	AASDHPPT	X	X		cytoplasm,cytosol	metabolic process	protein binding,metal ion binding,catalytic activity	L-aminoadipate-semialdehyde dehydrogenase-phosphopantetheinyl transferase
IPI00025346.3	PEX14	X			membrane,cytoplasm,nucleus	cell organization and biogenesis,transport,metabolic process,regulation of biological process,cellular component movement	protein binding	Isoform 1 of Peroxisomal membrane protein PEX14
IPI00016249.4	FXR1		X		cytoplasm,organelle lumen,nucleus	cell death,development,metabolic process,regulation of biological process,cell differentiation	RNA binding	Isoform 1 of Fragile X mental retardation syndrome-related protein 1
IPI00145260.3	IBA57	X	X		mitochondrion,cytoplasm	metabolic process	catalytic activity	Putative transferase CAF17, mitochondrial
IPI00784880.2	LOC440934			X				Cancer/testis antigen 75
IPI00215965.3	HNRNPA1	X	X		spliceosomal complex,cytoplasm,organelle lumen,nucleus	transport,metabolic process	DNA binding,RNA binding,nucleotide binding	Isoform A1-B of Heterogeneous nuclear ribonucleoprotein A1
IPI00009680.3	MRPL44		X		mitochondrion,cytoplasm,ribosome	metabolic process	RNA binding,catalytic activity	39S ribosomal protein L44, mitochondrial

IPI	GENE	Alzheimer's Hippocampus	Control hippocampus	CSF	Cellular localization	Biological process	Molecular function	Protein Description
IPI00409679.1	SHC3	X	X		cytoplasm,cytosol	development,regulation of biological process,response to stimulus,cell communication	protein binding,signal transducer activity	Isoform p64 of SHC-transforming protein 3
IPI00012535.1	DNAJA1	X	X		membrane	metabolic process,regulation of biological process,response to stimulus,cellular component movement,cell communication,reproduction	protein binding,metal ion binding,nucleotide binding	DnaJ homolog subfamily A member 1
IPI00302436.6	HDHD1	X	X			metabolic process	metal ion binding,catalytic activity	Isoform 1 of Pseudouridine-5'-monophosphatase
IPI00016670.3	LAMTOR1	X	X		membrane,cytoplasm,Golgi,vacuole,endosome	cell organization and biogenesis,transport,metabolic process,regulation of biological process,response to stimulus,cell communication,cell growth	protein binding	Ragulator complex protein LAMTOR1
IPI00293276.1 0	MIF	X	X	X	extracellular,cell surface,cytoplasm	cell death,cell proliferation,cell organization and biogenesis,transport,metabolic process,regulation of biological process,response to stimulus,cell communication,defense response	protein binding,catalytic activity	Macrophage migration inhibitory factor
IPI00442911.1	IGHV4-31			X			protein binding	CDNA FLJ26266 fis, clone DMC05613
IPI00074489.1	NDUFB10	X	X		membrane,mitochondrion,cytoplasm	metabolic process	catalytic activity	Uncharacterized protein
IPI00007277.2	LRRFIP2			X	cytoskeleton	regulation of biological process,response to stimulus,cell communication	protein binding,motor activity,catalytic activity	Isoform 1 of Leucine-rich repeat flightless-interacting protein 2
IPI00024766.1	PLXNC1			X	membrane	development,cell organization and biogenesis,regulation of biological process,response to stimulus,cell communication,cell differentiation	protein binding,receptor activity	Plexin-C1
IPI00007926.1	C6orf108	X	X		cytoplasm,organelle lumen,nucleus	cell proliferation,cell organization and biogenesis,metabolic process,regulation of biological process,cell growth	catalytic activity	Isoform 1 of Deoxyribonucleoside 5'-monophosphate N-glycosidase
IPI00219456.5	P4HTM		X		membrane,endoplasmic reticulum,cytoplasm	metabolic process	metal ion binding,catalytic activity	Isoform 1 of Transmembrane prolyl 4-hydroxylase
IPI00023748.3	NACA	X	X		cytoplasm,nucleus	transport,metabolic process	DNA binding	Nascent polypeptide-associated complex subunit alpha
IPI00016968.1	SDHC		X		membrane,mitochondrion,cytoplasm	transport,metabolic process	metal ion binding,catalytic activity	Isoform 1 of Succinate dehydrogenase cytochrome b560 subunit, mitochondrial
IPI00395769.2	ATP5C1	X	X		membrane	metabolic process,transport	transporter activity,catalytic activity	Isoform Heart of ATP synthase subunit gamma, mitochondrial
IPI00289815.2	WDR7	X	X				protein binding	Isoform 2 of WD repeat-containing protein 7
IPI00291510.3	IMPDH2	X	X		membrane,cytoplasm,cytosol,nucleus	cell proliferation,metabolic process	protein binding,DNA binding,RNA binding,metal ion binding,nucleotide binding,catalytic activity	Inosine-5'-monophosphate dehydrogenase 2
IPI00260090.3	ELMO2	X	X		cytoskeleton	transport	protein binding	cDNA FLJ60834, highly similar to Engulfment and cell motility protein 2
IPI00550792.4	BIN2			X	cytoplasm		protein binding	Isoform 1 of Bridging integrator 2
IPI00872861.1	RNFT1			X			protein binding,metal ion binding	PTD016 protein
IPI00166079.4	VKORC1L1	X	X		membrane			Vitamin K epoxide reductase complex subunit 1-like protein 1
IPI00550852.4	DCTN4	X	X		cytoskeleton,cytoplasm,nucleus		protein binding	Isoform 1 of Dynactin subunit 4
IPI00014165.1	HIST1H2APS4	X			chromosome,nucleus	cell organization and biogenesis,metabolic process	DNA binding	Histone H2A
IPI00718806.4	AHRR			X		metabolic process,regulation of biological process		Isoform 2 of Aryl hydrocarbon receptor repressor
IPI00746623.2	HABP2			X	extracellular	metabolic process	protein binding,catalytic activity	Hyaluronan-binding protein 2
IPI00880120.1	ABHD14A			X	membrane,cytoplasm		catalytic activity	Abhydrolase domain-containing protein 14A
IPI00785067.1	IGH@			X	membrane		protein binding	IGH@ protein
IPI00181743.4	BAIAP3			X		transport,regulation of biological process,response to stimulus,cell communication	protein binding	Isoform 1 of BAI1-associated protein 3
IPI00301907.3	HLCS		X		mitochondrion,cytoplasm,organelle lumen,chromosome,cytosol,nucleus	cell proliferation,cell organization and biogenesis,metabolic process,response to stimulus	protein binding,nucleotide binding,catalytic activity	Biotin--protein ligase
IPI00032328.2	KNG1			X	extracellular,membrane,cytoplasm,organelle lumen	cell death,metabolic process,transport,regulation of biological process,response to stimulus,defense response,cellular homeostasis,coagulation	protein binding,metal ion binding,enzyme regulator activity	Isoform HMW of Kininogen-1
IPI00020418.1	RRAS	X	X		membrane	development,cell organization and biogenesis,metabolic process,regulation of biological process,response to stimulus,cellular component movement,cell communication,cell differentiation	protein binding,nucleotide binding,catalytic activity	Ras-related protein R-Ras

IPI	GENE	Alzheimer's Hippocampus	Control hippocampus	CSF	Cellular localization	Biological process	Molecular function	Protein Description
IPI00100247.3	TMX4	X	X		membrane	metabolic process,transport,regulation of biological process,cellular homeostasis		Thioredoxin-related transmembrane protein 4
IPI00002280.1	PCSK1N	X	X	X	extracellular,cytoplasm,Golgi	development,metabolic process,regulation of biological process,response to stimulus,cell communication	protein binding,enzyme regulator activity	ProSAAS
IPI00947307.1	CP			X		metabolic process	metal ion binding,catalytic activity	cDNA FLJ58075, highly similar to Ceruloplasmin
IPI00736251.2	PPM1H	X	X			metabolic process	catalytic activity	Protein phosphatase 1H
IPI00554579.4	MAP7D2	X	X					Isoform 2 of MAP7 domain-containing protein 2
IPI00295485.4	HSPA4L	X	X		cytoplasm,nucleus	metabolic process,response to stimulus	nucleotide binding	Heat shock 70 kDa protein 4L
IPI00783390.3	CHL1			X	extracellular,membrane	cell organization and biogenesis,development,regulation of biological process,response to stimulus,cell communication,cell differentiation	protein binding	Isoform 1 of Neural cell adhesion molecule L1-like protein
IPI00289876.6	STX7	X	X	X	membrane,cytoplasm,vacuole,endosome	cell organization and biogenesis,transport	protein binding	Isoform 1 of Syntaxin-7
IPI00027780.1	MMP2			X	extracellular,membrane,cytoplasm,nucleus	development,cell organization and biogenesis,metabolic process,response to stimulus,reproduction	metal ion binding,catalytic activity	72 kDa type IV collagenase
IPI00784258.3	LTBP1			X	extracellular,cell surface,membrane	regulation of biological process,response to stimulus,cell communication	signal transducer activity,protein binding,metal ion binding,receptor activity,catalytic activity	Isoform Long of Latent-transforming growth factor beta-binding protein 1
IPI00215610.2	MPP1			X	cytoskeleton,membrane,cytoplasm	regulation of biological process,response to stimulus,cellular component movement,cell communication	protein binding,catalytic activity	55 kDa erythrocyte membrane protein
IPI00034006.1	PTPN23	X	X	X	cytoskeleton,cytoplasm,nucleus,endosome	cell organization and biogenesis,metabolic process	protein binding,catalytic activity	Tyrosine-protein phosphatase non-receptor type 23
IPI00396387.3	GNL1	X	X		extracellular,membrane,cytoplasm,cytosol	transport,metabolic process,regulation of biological process,response to stimulus,cell communication	transporter activity,nucleotide binding,structural molecule activity,catalytic activity	Isoform 1 of Guanine nucleotide-binding protein-like 1
IPI00216293.6	TST	X	X		mitochondrion,membrane,cytoplasm,organelle lumen	metabolic process,transport	RNA binding,catalytic activity	Thiosulfate sulfurtransferase
IPI00217740.6	DZANK1			X	membrane	regulation of biological process,response to stimulus,cell communication		C20orf12 protein
IPI00032465.4	KCNN3	X	X		cytoskeleton,membrane,cytoplasm,cytosol	metabolic process,transport,cell communication	protein binding,transporter activity	Isoform 1 of Small conductance calcium-activated potassium channel protein 3
IPI00008290.3	EPHA5		X	X	membrane	metabolic process,regulation of biological process,response to stimulus,cell communication	protein binding,signal transducer activity,nucleotide binding,receptor activity,catalytic activity	EPHA5 protein
IPI00030275.5	TRAP1	X	X	X	mitochondrion,cytoplasm	metabolic process,response to stimulus	protein binding,nucleotide binding	Heat shock protein 75 kDa, mitochondrial
IPI00030355.1	PPP1R11		X				enzyme regulator activity	Protein phosphatase 1 regulatory subunit 11
IPI00180292.5	BAIAP2	X	X			cell organization and biogenesis,regulation of biological process,response to stimulus,cell communication	protein binding	Isoform 5 of Brain-specific angiogenesis inhibitor 1-associated protein 2
IPI00375881.1	PKD1L3			X	cell surface,membrane	transport,regulation of biological process,response to stimulus,cell communication	signal transducer activity,protein binding,transporter activity,receptor activity	Polycystic kidney disease protein 1-like 3
IPI00003366.1	NTRK2		X	X	membrane,cytoplasm,endosome	cell death,cell proliferation,cell organization and biogenesis,development,metabolic process,regulation of biological process,response to stimulus,cellular component movement,cell communication,cell differentiation	protein binding,signal transducer activity,nucleotide binding,receptor activity,catalytic activity	Isoform TrkB of BDNF/NT-3 growth factors receptor
IPI00300725.7	KRT6A			X	cytoskeleton	cell proliferation,regulation of biological process,cell differentiation	protein binding,motor activity,structural molecule activity,catalytic activity	Keratin, type II cytoskeletal 6A
IPI00013219.1	ILK			X	cytoskeleton,membrane,cytoplasm,cytosol	cell death,cell proliferation,development,cell organization and biogenesis,metabolic process,regulation of biological process,response to stimulus,cellular component movement,cell communication,cellular homeostasis,cell differentiation,cell growth	signal transducer activity,protein binding,nucleotide binding,catalytic activity	Integrin-linked protein kinase
IPI00178415.4	PGAP1		X		membrane,endoplasmic reticulum,cytoplasm	development,transport,metabolic process	catalytic activity	Isoform 1 of GPI inositol-deacylase
IPI00221117.3	ACYP1		X	X		metabolic process	catalytic activity	Acylphosphatase-1
IPI00030179.3	RPL7		X		cytoplasm,ribosome,cytosol	cell organization and biogenesis,metabolic process,transport,reproduction	protein binding,DNA binding,RNA binding,structural molecule activity	60S ribosomal protein L7

IPI	GENE	Alzheimer's Hippocampus	Control hippocampus	CSF	Cellular localization	Biological process	Molecular function	Protein Description
IPI00018783.1	ITPA	X	X		cytoplasm	cell organization and biogenesis,metabolic process	metal ion binding,nucleotide binding,catalytic activity	Inosine triphosphate pyrophosphatase
IPI00328113.4	FBN1			X	extracellular,cell surface,membrane	development,regulation of biological process,response to stimulus,cell communication	protein binding,metal ion binding,structural molecule activity	Fibrillin-1
IPI00021119.1	CHST1			X	membrane,cytoplasm,Golgi	metabolic process,response to stimulus,defense response	catalytic activity	Carbohydrate sulfotransferase 1
IPI00011285.1	CAPN1	X	X		membrane,cytoplasm	cell proliferation,metabolic process,regulation of biological process	protein binding,metal ion binding,catalytic activity	Calpain-1 catalytic subunit
IPI00021187.4	RUVBL1	X	X		cytoskeleton,membrane,cytoplasm,Golgi,organelle lumen,chromosome,nucleus	cell organization and biogenesis,cell division,metabolic process,regulation of biological process,response to stimulus,reproduction	protein binding,nucleotide binding,catalytic activity	Isoform 1 of RuvB-like 1
IPI00293336.6	MBLAC2	X	X				metal ion binding,catalytic activity	Isoform 1 of Metallo-beta-lactamase domain-containing protein 2
IPI00414554.5	ARPC5L	X	X		cytoskeleton,cytoplasm	cell organization and biogenesis,regulation of biological process	protein binding	Actin-related protein 2/3 complex subunit 5-like protein
IPI00216508.3	SNX3		X	X		cell communication	protein binding	Isoform 2 of Sorting nexin-3
IPI00410600.3	CACNA2D2		X	X			protein binding	Isoform 3 of Voltage-dependent calcium channel subunit alpha-2/delta-2
IPI00025864.5	BCHE			X	extracellular,membrane,endoplasmic reticulum,cytoplasm,organelle lumen,nucleus	metabolic process,regulation of biological process,response to stimulus,cell communication	catalytic activity	Butyrylcholinesterase, isoform CRA_b
IPI00005607.2	DBC1			X	cytoskeleton,cytoplasm	cell death,regulation of biological process	protein binding	Isoform 1 of Deleted in bladder cancer protein 1
IPI00240812.6	PDS5B	X	X				DNA binding	Uncharacterized protein
IPI00021841.1	APOA1	X	X	X	extracellular,membrane,endoplasmic reticulum,cytoplasm,organelle lumen	cell proliferation,development,cell organization and biogenesis,metabolic process,transport,regulation of biological process,response to stimulus,cellular component movement,defense response,cell communication,coagulation	protein binding,transporter activity,enzyme regulator activity	Apolipoprotein A-I
IPI00215716.1	EPB41L3	X	X		cytoskeleton	cell organization and biogenesis	protein binding,structural molecule activity	Isoform B of Band 4.1-like protein 3
IPI00003865.1	HSPA8	X	X	X	cell surface,membrane,cytoplasm,cytosol	cell organization and biogenesis,transport,metabolic process,regulation of biological process,response to stimulus,cell communication	protein binding,nucleotide binding,catalytic activity	Isoform 1 of Heat shock cognate 71 kDa protein
IPI00743023.1	LOC650412			X	cytoskeleton,cytoplasm,ribosome	metabolic process,cellular component movement	nucleotide binding,motor activity,structural molecule activity,catalytic activity	PREDICTED: similar to dynein, axonemal, heavy polypeptide 1
IPI00783399.4	TMEM132D			X	membrane	metabolic process,regulation of biological process	protein binding	Isoform 1 of Transmembrane protein 132D
IPI00022334.1	OAT	X	X		mitochondrion,cytoplasm,organelle lumen	metabolic process	catalytic activity	Ornithine aminotransferase, mitochondrial
IPI00164949.4	TH1L			X	nucleus	metabolic process,regulation of biological process		TH1-like (Drosophila), isoform CRA_b
IPI00290566.1	TCP1	X	X		cytoskeleton,membrane,cytoplasm,Golgi,organelle lumen,chromosome,cytosol,nucleus	cell organization and biogenesis,metabolic process	protein binding,nucleotide binding	T-complex protein 1 subunit alpha
IPI00005161.3	ARPC2	X	X		cytoskeleton,membrane,cytoplasm,Golgi	cell organization and biogenesis,regulation of biological process,cellular component movement	protein binding,structural molecule activity	Actin-related protein 2/3 complex subunit 2
IPI00299608.3	PSMD1	X	X		cytoplasm,proteasome,organelle lumen,cytosol,nucleus	cell death,metabolic process,regulation of biological process,response to stimulus,cell communication	enzyme regulator activity	Isoform 1 of 26S proteasome non-ATPase regulatory subunit 1
IPI00942214.1	MAP2K4	X	X		cytoplasm,cytosol,nucleus	cell death,metabolic process,regulation of biological process,response to stimulus,defense response,cell communication	protein binding,nucleotide binding,catalytic activity	Isoform 1 of Dual specificity mitogen-activated protein kinase kinase 4
IPI00026015.1	SLC32A1	X	X		membrane,cytoplasm	transport,cell communication	transporter activity	Vesicular inhibitory amino acid transporter
IPI00012828.3	ACAA1	X	X		cytoplasm,organelle lumen	metabolic process	protein binding,catalytic activity	3-ketoacyl-CoA thiolase, peroxisomal
IPI00413587.2	BID	X		X	mitochondrion,membrane,cytoplasm,cytosol	cell death,cell proliferation,cell organization and biogenesis,development,transport,regulation of biological process,response to stimulus,cell communication	protein binding	Isoform 1 of BH3-interacting domain death agonist
IPI00555812.4	GC			X	extracellular,cytoplasm,organelle lumen,vacuole,cytosol	metabolic process,transport	protein binding,transporter activity	Vitamin D-binding protein
IPI00871556.1	ZFYVE28			X			metal ion binding	107 kDa protein
IPI00009148.1	NUDT3	X	X	X	cytoplasm	metabolic process,cell communication	metal ion binding,catalytic activity	Diphosphoinositol polyphosphate phosphohydrolase 1
IPI00647102.5	ATP1A2	X	X		membrane	transport,metabolic process	transporter activity,metal ion binding,nucleotide binding,catalytic activity	Uncharacterized protein
IPI00008944.2	SCG5	X		X	extracellular,cytoplasm	metabolic process,transport,regulation of biological process,response to stimulus,cell communication	protein binding,nucleotide binding,enzyme regulator activity	Isoform 1 of Neuroendocrine protein 7B2

IPI	GENE	Alzheimer's Hippocampus	Control hippocampus	CSF	Cellular localization	Biological process	Molecular function	Protein Description
IPI00296370.2	LCMT1		X				catalytic activity	Uncharacterized protein
IPI00829752.1	IGHV3-43			X				Myosin-reactive immunoglobulin heavy chain variable region
IPI00845366.1	SBF1	X	X		membrane,nucleus	metabolic process	protein binding,catalytic activity	Myotubularin-related protein 5
IPI00027434.1	RHOC	X	X		membrane,cytoplasm,cytosol,nucleus	cell death,cell organization and biogenesis,development,transport,metabolic process,cell division,regulation of biological process,response to stimulus,cellular component movement,cell communication,cell differentiation,cell growth	protein binding,signal transducer activity,nucleotide binding	Rho-related GTP-binding protein RhoC
IPI00830107.1	LOC651536			X				V4-2 protein
IPI00472961.2	IGKC			X			protein binding	IGKC protein
IPI00299778.2	PON3			X	extracellular,membrane	metabolic process,response to stimulus	protein binding,metal ion binding,catalytic activity	Serum paraoxonase/lactonase 3
IPI00426060.3	IGHA1			X	membrane		protein binding	Putative uncharacterized protein DKFZp686J11235 (Fragment)
IPI00020719.2	MAVS	X			mitochondrion,membrane,cytoplasm	cell organization and biogenesis,transport,metabolic process,regulation of biological process,response to stimulus,defense response,cell communication,reproduction	protein binding,signal transducer activity,transporter activity	Isoform 1 of Mitochondrial antiviral-signaling protein
IPI00044600.3	SORCS2			X	membrane	regulation of biological process,response to stimulus,cell communication	signal transducer activity,receptor activity	VPS10 domain-containing receptor SorCS2
IPI00026813.2	FNTA	X	X			metabolic process	catalytic activity	Farnesyltransferase, CAAX box, alpha, isoform CRA_a
IPI00023504.1	RAB3A	X	X		membrane,cytoplasm	development,cell organization and biogenesis,transport,metabolic process,regulation of biological process,response to stimulus,cell communication,cell differentiation	protein binding,nucleotide binding,catalytic activity,enzyme regulator activity	Ras-related protein Rab-3A
IPI00009958.6	COPS5	X	X		cytoplasm,organelle lumen,nucleus	metabolic process,regulation of biological process,response to stimulus,cell communication	protein binding,RNA binding,metal ion binding,catalytic activity	COP9 signalosome complex subunit 5
IPI00016669.1	RHEB	X	X		spliceosomal complex,membrane,cytoplasm,cytosol,nucleus	metabolic process,regulation of biological process,response to stimulus,cell communication	protein binding,metal ion binding,nucleotide binding,catalytic activity	GTP-binding protein Rheb
IPI00011603.2	PSMD3	X	X		cytoplasm,proteasome,organelle lumen,cytosol,nucleus	cell death,metabolic process,regulation of biological process,response to stimulus,cell communication	protein binding,enzyme regulator activity	26S proteasome non-ATPase regulatory subunit 3
IPI00005631.3	GCC2			X	membrane,cytoplasm,Golgi,nucleus	cell organization and biogenesis,transport,regulation of biological process	protein binding	Isoform 1 of GRIP and coiled-coil domain-containing protein 2
IPI00759776.1	ACTN1	X	X				protein binding	alpha-actinin-1 isoform a
IPI00001633.1	FLRT2			X	extracellular,membrane		protein binding,signal transducer activity	Leucine-rich repeat transmembrane protein FLRT2
IPI00023729.1	FN3K	X	X			metabolic process	catalytic activity	Fructosamine-3-kinase
IPI00028387.3	DDRGK1	X		X	endoplasmic reticulum,cytoplasm		protein binding	Isoform 1 of DDRGK domain-containing protein 1
IPI00295601.1	CEND1	X	X		membrane			Cell cycle exit and neuronal differentiation protein 1
IPI00465105.4	DNAJA4	X	X			metabolic process	protein binding	Isoform 2 of DnaJ homolog subfamily A member 4
IPI00329801.12	ANXA5	X	X	X	extracellular,cell surface,membrane,cytoplasm	cell death,cell organization and biogenesis,regulation of biological process,response to stimulus,cell communication,coagulation	protein binding,metal ion binding,enzyme regulator activity	Annexin A5
IPI00152145.1	OSR1			X	organelle lumen,nucleus	cell death,cell proliferation,development,metabolic process,regulation of biological process,response to stimulus,cell communication,cell differentiation,reproduction	metal ion binding	Protein odd-skipped-related 1
IPI00043731.2	RUNDC3B			X				Isoform 1 of RUN domain-containing protein 3B
IPI01018111.1	FKBP1A	X	X			metabolic process,regulation of biological process,response to stimulus,cell communication	catalytic activity	FK506 binding protein12
IPI00419250.6	GSTT2	X	X		cytoplasm		protein binding,catalytic activity	Glutathione S-transferase theta-2
IPI00011284.1	COMT	X	X		membrane,mitochondrion,cytoplasm,cytosol	cell proliferation,metabolic process,regulation of biological process,response to stimulus,cell communication,reproduction	metal ion binding,catalytic activity	Isoform Membrane-bound of Catechol O-methyltransferase

IPI	GENE	Alzheimer's Hippocampus	Control hippocampus	CSF	Cellular localization	Biological process	Molecular function	Protein Description
IPI00016422.2	DCC			X	membrane,cytoplasm,cytosol	cell death,development,cell organization and biogenesis,regulation of biological process,response to stimulus,cellular component movement,cell communication,cell growth,cell differentiation	protein binding,signal transducer activity,receptor activity	Netrin receptor DCC
IPI00141318.2	CKAP4	X	X		cytoskeleton,membrane,endoplasmic reticulum,cytoplasm		protein binding	Cytoskeleton-associated protein 4
IPI00940245.1	IGHA2			X	membrane		protein binding	Immunoglobulin heavy chain variant (Fragment)
IPI00297040.2	SPINK6			X	extracellular	metabolic process,regulation of biological process	protein binding,enzyme regulator activity	Serine protease inhibitor Kazal-type 6
IPI00008483.1	MAOA	X	X		membrane,mitochondrion,cytoplasm	transport,metabolic process,response to stimulus,cell communication	catalytic activity	Amine oxidase [flavin-containing] A
IPI00003921.2	EPB41			X	cytoskeleton,membrane,cytoplasm,Golgi,nucleus	cell organization and biogenesis	protein binding,structural molecule activity	Isoform 1 of Protein 4.1
IPI00002884.4	C17orf101			X	membrane	metabolic process	metal ion binding,catalytic activity	Isoform 1 of PKHD domain-containing transmembrane protein C17orf101
IPI00018429.1	PRRX2			X	organelle lumen,chromosome,nucleus	cell proliferation,development,metabolic process,regulation of biological process,response to stimulus,cell communication	DNA binding	Paired mesoderm homeobox protein 2
IPI00030877.2	Sep-15	X	X	X	endoplasmic reticulum,cytoplasm,organelle lumen	metabolic process		15 kDa selenoprotein isoform 1 precursor
IPI00218914.5	ALDH1A1	X	X	X	cytoplasm,cytosol	metabolic process,regulation of biological process,response to stimulus	catalytic activity,enzyme regulator activity	Retinal dehydrogenase 1
IPI00168885.5	DHX57			X			protein binding,metal ion binding,nucleotide binding,catalytic activity	Isoform 1 of Putative ATP-dependent RNA helicase DHX57
IPI00382412.6	BCAT1	X	X		cytoplasm,cytosol	cell proliferation,metabolic process	catalytic activity	Branched-chain-amino-acid aminotransferase, cytosolic
IPI00180384.5	DNAH7			X	cytoskeleton,cytoplasm	cellular component movement	motor activity,nucleotide binding,catalytic activity	Isoform 1 of Dynein heavy chain 7, axonemal
IPI00163601.2	EBLN2			X				Endogenous Bornavirus-like nucleoprotein 2
IPI00001477.1	DDR1			X	extracellular,membrane	cell proliferation,cell organization and biogenesis,development,metabolic process,transport,regulation of biological process,response to stimulus,cellular component movement,cell communication,cell growth,reproduction	protein binding,signal transducer activity,nucleotide binding,receptor activity,catalytic activity	Isoform 1 of Epithelial discoidin domain-containing receptor 1
IPI00943257.1	CD99L2			X	membrane			Isoform 1 of CD99 antigen-like protein 2
IPI00396961.4	LRFN5			X	membrane		protein binding	Leucine-rich repeat and fibronectin type-III domain-containing protein 5
IPI00027794.6	C11orf68		X					Isoform 2 of UPF0696 protein C11orf68
IPI00375370.1	SEC13		X				protein binding	SEC13-like 1 isoform b variant
IPI00016150.1	SERPINI1	X	X	X	extracellular	development,metabolic process,regulation of biological process	enzyme regulator activity	Neuroserpin
IPI00427330.3	SBDS	X	X		cytoskeleton,cytoplasm,organelle lumen,nucleus	cell proliferation,cell organization and biogenesis,development,metabolic process,regulation of biological process,response to stimulus,cellular component movement	protein binding,RNA binding	Ribosome maturation protein SBDS
IPI00014808.1	PAFAH1B3	X			cytoplasm	development,metabolic process	protein binding,catalytic activity	Platelet-activating factor acetylhydrolase IB subunit gamma
IPI00170551.1	SEMA6D			X	membrane		protein binding	Isoform 2 of Semaphorin-6D
IPI00001985.2	VPS18		X		cytoskeleton,membrane,cytoplasm,vacuole,endosome	cell organization and biogenesis,transport	protein binding,metal ion binding	Vacuolar protein sorting-associated protein 18 homolog
IPI00749245.1	SFRP1			X	extracellular,cell surface,membrane,cytoplasm,cytosol	cell death,development,cell organization and biogenesis,metabolic process,regulation of biological process,response to stimulus,reproduction,cell proliferation,transport,cellular component movement,cell communication,cell growth,cell differentiation	protein binding,signal transducer activity,receptor activity,catalytic activity	Secreted frizzled-related protein 1
IPI00107831.4	PTPRF		X	X	membrane,cytoplasm,endosome	cell death,cell proliferation,cell organization and biogenesis,development,metabolic process,regulation of biological process,response to stimulus,cell communication,cell differentiation	protein binding,signal transducer activity,receptor activity,catalytic activity	Isoform 1 of Receptor-type tyrosine-protein phosphatase F
IPI00174976.5	MPP5			X	membrane,cytoplasm	development,cell organization and biogenesis,cell communication,cellular homeostasis,cell differentiation	protein binding	Isoform 1 of MAGUK p55 subfamily member 5

IPI	GENE	Alzheimer's Hippocampus	Control hippocampus	CSF	Cellular localization	Biological process	Molecular function	Protein Description
IPI00383832.7	DVL3			X		development,metabolic process,regulation of biological process,response to stimulus,cell communication	signal transducer activity,protein binding,catalytic activity	Protein kinase C-binding protein RACK8 (Fragment)
IPI00394820.4	OLFML1			X	extracellular		protein binding	Olfactomedin-like protein 1
IPI00164930.5	KIAA1033	X	X		cytoplasm	metabolic process,transport,regulation of biological process	RNA binding	Isoform 2 of WASH complex subunit 7
IPI00940952.1	IGKC			X			protein binding	26 kDa protein
IPI00028450.1	SLC8A1			X	cytoskeleton,mitochondrion,membrane,cytoplasm	development,transport,regulation of biological process,response to stimulus,cell communication,cellular homeostasis,cell differentiation,coagulation	protein binding,transporter activity,metal ion binding	Isoform 1 of Sodium/calcium exchanger 1
IPI00855821.1	NRXN1			X				Isoform 3a of Neurexin-1-alpha
IPI00152199.7	FAM213B		X		cytoplasm	metabolic process	nucleotide binding,catalytic activity	prostamide/prostaglandin F synthase isoform b
IPI00003420.1	MAPRE2	X	X		cytoskeleton,cytoplasm	cell proliferation,cell organization and biogenesis,cell division,regulation of biological process,response to stimulus,cell communication	protein binding	Isoform 1 of Microtubule-associated protein RP/EB family member 2
IPI00217616.2	FAM126B		X					Protein FAM126B
IPI00550364.8	PGM2	X	X	X	cytoplasm,cytosol	metabolic process	metal ion binding,catalytic activity	Phosphoglucomutase-2
IPI00166075.1	LGI3	X		X	extracellular,membrane,cytoplasm	transport,regulation of biological process	protein binding	Leucine-rich repeat LGI family member 3
IPI00216780.3	CILP2			X				Cartilage intermediate layer protein 2 precursor
IPI00008569.1	YKT6	X			mitochondrion,membrane,endoplasmic reticulum,cytoplasm,Golgi,cytosol,endosome	transport	protein binding,catalytic activity	Synaptobrevin homolog YKT6
IPI00014311.6	CUL2	X	X			cell death,cell proliferation,metabolic process,regulation of biological process	protein binding	Cullin-2
IPI00008380.1	PPP2CA	X	X		cytoskeleton,membrane,mitochondrion,cytoplasm,chromosome,nucleus ,cytosol	cell death,cell organization and biogenesis,development,metabolic process,regulation of biological process,response to stimulus,cell communication,reproduction,cell differentiation,cell growth	protein binding,metal ion binding,catalytic activity	Serine/threonine-protein phosphatase 2A catalytic subunit alpha isoform
IPI00010130.3	GLUL	X	X		mitochondrion,cytoplasm,Golgi,cytosol	cell proliferation,metabolic process,transport,response to stimulus,cell communication	protein binding,nucleotide binding,catalytic activity	Glutamine synthetase
IPI00306710.2	CHRD			X	extracellular	cell proliferation,development,regulation of biological process,response to stimulus,cellular component movement,cell communication,cell differentiation	protein binding	Isoform 1 of Chordin
IPI00333410.1	UBE2Q1			X		metabolic process	protein binding,nucleotide binding,catalytic activity	Isoform 1 of Ubiquitin-conjugating enzyme E2 Q1
IPI00294705.2	PAPLN			X			catalytic activity,enzyme regulator activity	papilin, proteoglycan-like sulfated glycoprotein, isoform CRA_b
IPI00439446.3	MAN1A1			X	membrane		metal ion binding,catalytic activity	Uncharacterized protein
IPI00025086.4	COX5A	X	X		mitochondrion,membrane,cytoplasm	metabolic process	transporter activity,metal ion binding,catalytic activity	Cytochrome c oxidase subunit 5A, mitochondrial
IPI00396321.1	LRRC59	X	X		mitochondrion,membrane,endoplasmic reticulum,cytoplasm,organelle lumen		protein binding	Leucine-rich repeat-containing protein 59
IPI00382682.1	IGH@			X			protein binding	Putative matrix cell adhesion molecule-3
IPI00176193.6	COL14A1			X	extracellular	cell organization and biogenesis	protein binding,structural molecule activity	Isoform 1 of Collagen alpha-1(XIV) chain
IPI00847335.1	HLA-L			X	membrane	response to stimulus		FLJ45422 protein
IPI00024284.6	HSPG2	X	X	X	extracellular,membrane	cell organization and biogenesis,development,metabolic process,cell differentiation	protein binding	Basement membrane-specific heparan sulfate proteoglycan core protein
IPI00029260.2	CD14	X		X	extracellular,cell surface,membrane	cell death,cell organization and biogenesis,transport,regulation of biological process,response to stimulus,defense response,cell communication	protein binding,signal transducer activity,receptor activity	Monocyte differentiation antigen CD14
IPI00438923.3	CRYZL1		X		cytoplasm,cytosol	metabolic process	metal ion binding,nucleotide binding,catalytic activity	Quinone oxidoreductase-like protein 1
IPI00304331.2	B3GAT3			X	membrane,cytoplasm,Golgi	metabolic process	metal ion binding,catalytic activity	Galactosylgalactosylxylosylprotein 3-beta-glucuronosyltransferase 3

IPI	GENE	Alzheimer's Hippocampus	Control hippocampus	CSF	Cellular localization	Biological process	Molecular function	Protein Description
IPI00296165.7	C1R			X	extracellular	metabolic process,regulation of biological process,response to stimulus,defense response	metal ion binding,catalytic activity	Complement C1r subcomponent
IPI00168920.4	COL24A1			X	extracellular		structural molecule activity	Isoform 1 of Collagen alpha-1(XXIV) chain
IPI00025869.1	GLA			X	extracellular,cytoplasm,Golgi,vacuole,organelle lumen	metabolic process,regulation of biological process	protein binding,catalytic activity	Alpha-galactosidase A
IPI00221092.8	RPS16	X	X		cytoplasm,ribosome,cytosol	cell organization and biogenesis,metabolic process,transport,reproduction	RNA binding,structural molecule activity	40S ribosomal protein S16
IPI00291807.8	CPAMD8			X	extracellular,membrane		enzyme regulator activity	Isoform 2 of C3 and PZP-like alpha-2-macroglobulin domain-containing protein 8
IPI00026612.1	PPM1B	X	X	X	cytoplasm,cytosol	metabolic process,regulation of biological process,response to stimulus,cell communication	protein binding,metal ion binding,catalytic activity	Isoform Beta-1 of Protein phosphatase 1B
IPI00001755.1	GPC6			X	extracellular,membrane		protein binding	Glypican-6
IPI00217781.3	C11orf87			X	membrane			Uncharacterized protein C11orf87
IPI00412408.1	BRCA2			X	cytoskeleton,cytoplasm,organelle lumen,nucleus	cell death,cell proliferation,development,cell organization and biogenesis,cell division,metabolic process,regulation of biological process,response to stimulus,cell communication,reproduction,cell differentiation	protein binding,DNA binding,catalytic activity	Breast cancer type 2 susceptibility protein
IPI00219131.8	ICOSLG			X	cell surface,membrane	cell proliferation,metabolic process,regulation of biological process,response to stimulus,cell communication,defense response	protein binding	Isoform 1 of ICOS ligand
IPI00007814.3	ATP6V1C1	X	X		membrane,cytoplasm,cytosol	transport,regulation of biological process,response to stimulus,cell communication,cellular homeostasis	protein binding,transporter activity,catalytic activity	V-type proton ATPase subunit C 1
IPI00032292.1	TIMP1			X	extracellular,cytoplasm,organelle lumen	cell death,cell proliferation,development,cell organization and biogenesis,transport,metabolic process,regulation of biological process,response to stimulus,cell differentiation,coagulation	protein binding,metal ion binding,enzyme regulator activity	Metalloproteinase inhibitor 1
IPI00022039.2	CD84			X				Isoform 3 of SLAM family member 5
IPI00873863.2	BDNF			X			protein binding	29 kDa protein
IPI00293088.7	GAA	X	X	X	membrane,cytoplasm,vacuole	cell organization and biogenesis,development,metabolic process,regulation of biological process,response to stimulus,cellular homeostasis	catalytic activity	Lysosomal alpha-glucosidase
IPI00604551.3	CDCA7			X	nucleus	cell proliferation,metabolic process,regulation of biological process		Isoform 1 of Cell division cycle-associated protein 7
IPI00186966.3	BIN1	X	X	X	cytoskeleton,cytoplasm,nucleus	cell proliferation,development,cell organization and biogenesis,metabolic process,transport,regulation of biological process,cell differentiation	protein binding	Isoform IIA of Myc box-dependent-interacting protein 1
IPI00219806.7	S100A7			X	extracellular,membrane,endoplasmic reticulum,cytoplasm,nucleus,cytosol	development,regulation of biological process,response to stimulus,cellular component movement,defense response,cell communication,cell differentiation	protein binding,metal ion binding	Protein S100-A7
IPI00023673.1	LGALS3BP	X	X	X	extracellular,membrane	regulation of biological process,response to stimulus,cell communication,defense response	protein binding,receptor activity	Galectin-3-binding protein
IPI00011651.3	PTPRG			X	membrane	metabolic process,regulation of biological process,response to stimulus,cell communication	signal transducer activity,protein binding,receptor activity,catalytic activity	Isoform 1 of Receptor-type tyrosine-protein phosphatase gamma
IPI00604664.5	NDUFS1	X	X			metabolic process	catalytic activity	NADH-ubiquinone oxidoreductase 75 kDa subunit, mitochondrial isoform 5
IPI00102435.4	COL21A1			X	extracellular,cytoplasm		protein binding	Isoform 1 of Collagen alpha-1(XXI) chain
IPI00290283.6	MASP1			X		metabolic process	metal ion binding,catalytic activity	Isoform 2 of Mannan-binding lectin serine protease 1
IPI00014589.1	CLTB	X	X		membrane,cytoplasm,Golgi	transport	protein binding,structural molecule activity	Isoform Brain of Clathrin light chain B
IPI00026519.1	PPIF	X	X		mitochondrion,membrane,cytoplasm,organelle lumen	cell death,cell organization and biogenesis,metabolic process,transport,regulation of biological process	catalytic activity	Peptidyl-prolyl cis-trans isomerase F, mitochondrial
IPI00853376.1	ASXL3			X	nucleus	metabolic process,regulation of biological process	metal ion binding	Isoform 1 of Putative Polycomb group protein ASXL3
IPI00556579.3	GSTZ1	X	X				protein binding	maleylacetoacetate isomerase isoform 1
IPI00029131.1	PCSK2		X	X	extracellular,membrane,cytoplasm	development,metabolic process	protein binding,catalytic activity	Neuroendocrine convertase 2
IPI00784950.1	IGH@			X	membrane		protein binding	Putative uncharacterized protein DKFZp686L19235
IPI00221226.7	ANXA6	X	X		membrane,cytoplasm	transport,regulation of biological process	protein binding,metal ion binding	Annexin A6

IPI	GENE	Alzheimer's Hippocampus	Control hippocampus	CSF	Cellular localization	Biological process	Molecular function	Protein Description
IPI00005794.2	CPQ			X		metabolic process	catalytic activity	Uncharacterized protein
IPI00153049.3	MXRA8			X				Isoform 2 of Matrix-remodeling-associated protein 8
IPI00020131.1	SOS1			X	membrane,cytoplasm,cytosol	cell death,development,cell organization and biogenesis,metabolic process,regulation of biological process,response to stimulus,cellular component movement,cell communication,cell differentiation,coagulation	protein binding,DNA binding,enzyme regulator activity	Son of sevenless homolog 1
IPI00396485.3	EEF1A1	X	X		cytoplasm,cytosol,nucleus	metabolic process,regulation of biological process	protein binding,RNA binding,nucleotide binding,catalytic activity	Elongation factor 1-alpha 1
IPI00328293.3	SRRM1	X	X		membrane	metabolic process		cDNA FLJ61739, highly similar to Serine/arginine repetitive matrix protein 1
IPI00852758.6	ANKRD18B			X			protein binding	Ankyrin repeat domain-containing protein 18B
IPI00027443.5	CARS	X	X		cytoplasm	metabolic process	nucleotide binding,catalytic activity	cysteinyl-tRNA synthetase, cytoplasmic isoform c
IPI00022421.2	COX7A2L	X	X		mitochondrion,membrane,cytoplasm	metabolic process	transporter activity,catalytic activity	Cytochrome c oxidase subunit 7A-related protein, mitochondrial
IPI00042580.4	APOO	X	X		extracellular,membrane	transport		Isoform 1 of Apolipoprotein O
IPI00430291.6	CAMK2D			X	membrane,endoplasmic reticulum,cytoplasm,organelle lumen,cytosol,nucleus	cell organization and biogenesis,transport,metabolic process,regulation of biological process,response to stimulus,defense response,cell communication,cellular homeostasis,cell growth	protein binding,nucleotide binding,catalytic activity	Isoform Delta 2 of Calcium/calmodulin-dependent protein kinase type II subunit delta
IPI00292393.5	SCN4A			X	membrane	transport,regulation of biological process	protein binding,transporter activity	Sodium channel protein type 4 subunit alpha
IPI00385437.1	OGFRL1	X	X		membrane		receptor activity	Opioid growth factor receptor-like protein 1
IPI00171769.1	FUNDC2	X			mitochondrion,cytoplasm			FUN14 domain-containing protein 2
IPI00293836.5	CADM2	X	X	X			protein binding	Isoform 3 of Cell adhesion molecule 2
IPI00299547.4	LCN2			X	extracellular,cytoplasm,cytosol	cell death,cell organization and biogenesis,transport,metabolic process,regulation of biological process,response to stimulus,defense response,cell communication,cellular homeostasis	protein binding,transporter activity,metal ion binding	Isoform 1 of Neutrophil gelatinase-associated lipocalin
IPI00029591.3	SELPLG			X	membrane	response to stimulus,cellular component movement,coagulation	protein binding	P-selectin glycoprotein ligand 1
IPI00329332.1	STX12	X	X	X	membrane,cytoplasm,Golgi,endosome	metabolic process,transport,regulation of biological process	protein binding	Syntaxin-12
IPI00012493.1	RPS20	X	X		cytoplasm,ribosome,cytosol	cell organization and biogenesis,metabolic process,transport,reproduction	RNA binding,structural molecule activity	40S ribosomal protein S20
IPI00444331.3	WHSC1L1			X			protein binding	Isoform 4 of Histone-lysine N-methyltransferase NSD3
IPI00004534.5	PFAS	X	X	X	cytoplasm,cytosol	metabolic process	nucleotide binding,catalytic activity	Phosphoribosylformylglycinamidine synthase
IPI00221124.2	KCNAB1	X	X		membrane,cytoplasm	transport	transporter activity	Isoform KvB1.1 of Voltage-gated potassium channel subunit beta-1
IPI00019943.1	AFM			X	extracellular	transport		Afamin
IPI00479997.4	STMN1	X	X	X	cytoskeleton,membrane,cytoplasm	development,cell organization and biogenesis,regulation of biological process,response to stimulus,cell communication,cell differentiation	protein binding,signal transducer activity	Isoform 1 of Stathmin
IPI00004409.2	DDR2			X	membrane	cell proliferation,development,cell organization and biogenesis,metabolic process,regulation of biological process,response to stimulus,cellular component movement,cell communication,cell differentiation	protein binding,signal transducer activity,nucleotide binding,receptor activity,catalytic activity	Discoidin domain-containing receptor 2
IPI00829834.2	IGKV3-7			X	extracellular,membrane	metabolic process,regulation of biological process,response to stimulus,defense response	protein binding	Ig kappa chain V-III region VH (Fragment)
IPI00032825.2	TMED7			X	membrane,endoplasmic reticulum,cytoplasm,Golgi	transport		Transmembrane emp24 domain-containing protein 7
IPI00215888.4	SRP72	X	X		membrane,cytoplasm,organelle lumen,cytosol,nucleus	transport,metabolic process,response to stimulus	protein binding,RNA binding	Signal recognition particle 72 kDa protein
IPI00023779.1	NIT1	X	X		mitochondrion,cytoplasm	metabolic process	catalytic activity	Isoform 2 of Nitrilase homolog 1
IPI00305212.5	COMMD9	X	X					Isoform 1 of COMM domain-containing protein 9
IPI00006612.2	SNAP91	X	X		membrane,cytoplasm	cell organization and biogenesis,transport	protein binding	Isoform 1 of Clathrin coat assembly protein AP180

IPI	GENE	Alzheimer's Hippocampus	Control hippocampus	CSF	Cellular localization	Biological process	Molecular function	Protein Description
IPI00030774.3	TBCD	X	X		membrane			Isoform 4 of Tubulin-specific chaperone D
IPI00183781.4	AP2S1	X	X					Isoform 2 of AP-2 complex subunit sigma
IPI00328680.2	MCFD2		X	X	extracellular,membrane,endoplasmic reticulum,cytoplasm,Golgi	metabolic process,transport	metal ion binding	Multiple coagulation factor deficiency protein 2
IPI00219483.1	SNRNP70	X					nucleotide binding	Isoform 2 of U1 small nuclear ribonucleoprotein 70 kDa
IPI00026302.3	RPL31		X		cytoplasm,ribosome,cytosol	cell organization and biogenesis,metabolic process,transport,reproduction	RNA binding,structural molecule activity	60S ribosomal protein L31
IPI00019448.3	CEP41	X	X		cytoskeleton,cytoplasm,cytosol	cell organization and biogenesis,metabolic process,transport	protein binding	Isoform 1 of Centrosomal protein of 41 kDa
IPI00384016.1	DLST			X		metabolic process	catalytic activity	Full-length cDNA 5-PRIME end of clone CS0DJ009YL13 of T cells (Jurkat cell line) of Homo sapiens
IPI00384938.1	IGHG1			X	membrane		protein binding	Putative uncharacterized protein DKFZp686N02209
IPI00000070.1	LDLR			X	extracellular,cell surface,membrane,cytoplasm,Golgi,vacuole,endosome	transport,metabolic process,regulation of biological process	protein binding,metal ion binding,receptor activity	Low-density lipoprotein receptor
IPI00478231.2	RHOA	X	X		cytoskeleton,membrane,mitochondrion,cytoplasm,cytosol,nucleus	cell death,cell organization and biogenesis,development,metabolic process,regulation of biological process,response to stimulus,cell proliferation,cell division,transport,cellular component movement,cell communication,cell differentiation,coagulation	protein binding,nucleotide binding,catalytic activity	Transforming protein RhoA
IPI00472249.2	PTPRN2			X		metabolic process	catalytic activity	receptor-type tyrosine-protein phosphatase N2 isoform 2 precursor
IPI00007731.1	BAG5	X	X		cytoplasm	cell death,cell organization and biogenesis,metabolic process,regulation of biological process	protein binding	Isoform 1 of BAG family molecular chaperone regulator 5
IPI00921125.2	GATSL1	X	X					GATS-like protein 1
IPI00645016.3	S100A1	X	X		endoplasmic reticulum,cytoplasm,nucleus	metabolic process,regulation of biological process,response to stimulus,cell communication	protein binding,metal ion binding	Protein S100-A1
IPI00026104.1	IDS			X	cytoplasm,vacuole	metabolic process	metal ion binding,catalytic activity	Isoform 1 of Iduronate 2-sulfatase
IPI00060146.6	SMCR7			X	mitochondrion,membrane,cytoplasm	cell organization and biogenesis	protein binding	Isoform 1 of Smith-Magenis syndrome chromosomal region candidate gene 7 protein
IPI00335168.9	MYL6	X	X	X	cytoskeleton,cytoplasm,cytosol	cell organization and biogenesis,development,response to stimulus,cellular component movement,cell differentiation	protein binding,metal ion binding,motor activity,structural molecule activity,catalytic activity	Isoform Non-muscle of Myosin light polypeptide 6
IPI00017448.1	RPS21	X			cytoplasm,ribosome,cytosol	cell organization and biogenesis,transport,metabolic process,reproduction	protein binding,structural molecule activity	40S ribosomal protein S21
IPI00024587.1	CALY			X	membrane,cytoplasm	cell organization and biogenesis,transport,regulation of biological process,response to stimulus,cell communication	protein binding	Isoform 1 of Neuron-specific vesicular protein calcyon
IPI00797893.1	GDAP1		X			response to stimulus		unnamed protein product
IPI00004363.1	STK39	X	X			metabolic process	nucleotide binding,catalytic activity	STE20/SPS1-related proline-alanine-rich protein kinase
IPI00029863.4	SERPINF2			X			enzyme regulator activity	55 kDa protein
IPI00019530.1	TIE1			X	membrane	development,cell organization and biogenesis,metabolic process,regulation of biological process,response to stimulus,cellular component movement,cell communication	protein binding,signal transducer activity,nucleotide binding,receptor activity,catalytic activity	Tyrosine-protein kinase receptor Tie-1
IPI00217943.3	RAB6A	X	X		membrane,cytoplasm,nucleus	transport,regulation of biological process,response to stimulus,cell communication	transporter activity,nucleotide binding	Isoform 2 of Ras-related protein Rab-6A
IPI00251596.2	COL23A1			X	extracellular,cell surface,membrane	cell organization and biogenesis	protein binding	Isoform 1 of Collagen alpha-1(XXIII) chain
IPI00289746.2	PAK1	X	X	X		metabolic process	protein binding,nucleotide binding,catalytic activity	Isoform 2 of Serine/threonine-protein kinase PAK 1
IPI00020075.4	ABHD10	X	X		mitochondrion,cytoplasm,cytosol	metabolic process	catalytic activity	Abhydrolase domain-containing protein 10, mitochondrial
IPI00181706.4	PCP4L1	X	X					Purkinje cell protein 4-like protein 1
IPI00550239.4	H1F0	X	X		cytoplasm,Golgi,organelle lumen,chromosome,nucleus	cell death,cell organization and biogenesis,metabolic process	DNA binding	Histone H1.0
IPI00021537.5	OGFR	X	X		extracellular,membrane,cytoplasm,nucleus	cell organization and biogenesis,regulation of biological process,response to stimulus,cell communication,cell growth	signal transducer activity,receptor activity	Isoform 1 of Opioid growth factor receptor
IPI00239077.5	HINT1	X	X	X	cytoskeleton,membrane,cytoplasm,nucleus	metabolic process,regulation of biological process,response to stimulus,cell communication	protein binding,catalytic activity	Histidine triad nucleotide-binding protein 1

IPI	GENE	Alzheimer's Hippocampus	Control hippocampus	CSF	Cellular localization	Biological process	Molecular function	Protein Description
IPI00001434.1	PCDHB14			X	membrane	cell organization and biogenesis,development,cell communication	metal ion binding	Protocadherin beta-14
IPI00013452.1 1	EPRS	X	X		cytoplasm,cytosol	cell organization and biogenesis,metabolic process	protein binding,RNA binding,nucleotide binding,catalytic activity	Bifunctional aminoacyl-tRNA synthetase
IPI00018206.4	GOT2	X	X	X	mitochondrion,membrane,cytoplasm,organelle lumen	transport,metabolic process,response to stimulus	catalytic activity	Aspartate aminotransferase, mitochondrial
IPI00011592.3	DYNC1LI2	X	X	X	cytoskeleton,cytoplasm	transport	motor activity,nucleotide binding,catalytic activity	Cytoplasmic dynein 1 light intermediate chain 2
IPI00554538.6	TPP1			X		metabolic process	catalytic activity	Uncharacterized protein
IPI00477747.3	FSTL4			X	extracellular		protein binding,metal ion binding	Isoform 1 of Follistatin-related protein 4
IPI00009111.1	TPBG			X	membrane,endoplasmic reticulum,cytoplasm		protein binding	Trophoblast glycoprotein
IPI00016604.1	PDE4B	X	X		cytoplasm,cytosol,nucleus	metabolic process,regulation of biological process,response to stimulus,cell communication	metal ion binding,catalytic activity	Isoform PDE4B1 of cAMP-specific 3',5'-cyclic phosphodiesterase 4B
IPI00004373.1	MBL2			X	extracellular	cell organization and biogenesis,metabolic process,transport,regulation of biological process,response to stimulus,defense response	protein binding	Mannose-binding protein C
IPI00008403.2	CHST7			X	membrane,cytoplasm,Golgi	metabolic process	catalytic activity	Carbohydrate sulfotransferase 7
IPI00018208.2	TTC33			X			protein binding	Tetratricopeptide repeat protein 33
IPI00186299.7	SYN2	X	X		cytoplasm	transport,cell communication		Isoform IIb of Synapsin-2
IPI00030363.1	ACAT1	X	X		mitochondrion,membrane,cytoplasm,organelle lumen	cell organization and biogenesis,development,metabolic process,response to stimulus	protein binding,metal ion binding,catalytic activity	Acetyl-CoA acetyltransferase, mitochondrial
IPI00021842.1	APOE	X	X	X	extracellular,cytoskeleton,cell surface,membrane,cytoplasm,Golgi,endosome	cell death,development,cell organization and biogenesis,metabolic process,regulation of biological process,response to stimulus,defense response,cell proliferation,transport,cellular component movement,cell communication,cellular homeostasis,cell differentiation,cell growth,coagulation	antioxidant activity,protein binding,transporter activity,metal ion binding,catalytic activity,enzyme regulator activity	Apolipoprotein E
IPI00000160.2	POMC			X	extracellular,cytoplasm		protein binding	Proopiomelanocortin preproprotein
IPI00027235.1	ATRN			X	extracellular,membrane,cytoplasm	regulation of biological process,response to stimulus,defense response	protein binding,receptor activity	Isoform 1 of Attractin
IPI00294495.5	UFC1		X			metabolic process	protein binding,catalytic activity	Ubiquitin-fold modifier-conjugating enzyme 1
IPI00028134.1	KCNA2	X	X		membrane	cell organization and biogenesis,transport,regulation of biological process,cell communication	protein binding,transporter activity	Potassium voltage-gated channel subfamily A member 2
IPI00328829.4	ITIH5			X		metabolic process	protein binding,enzyme regulator activity	Similar to Inter-alpha trypsin inhibitor heavy chain precursor 5 isoform 3
IPI00413293.6	TOR1A	X	X		endoplasmic reticulum,cytoplasm,nucleus	metabolic process	nucleotide binding	cDNA FLJ56343, highly similar to Torsin A
IPI00021301.1	ASPA	X	X		cytoplasm,nucleus	development,metabolic process,regulation of biological process,cell communication,cellular homeostasis,cell differentiation	metal ion binding,catalytic activity	Aspartoacylase
IPI00470467.5	POR	X	X		mitochondrion,membrane,endoplasmic reticulum,cytoplasm	cell death,cell proliferation,development,metabolic process,regulation of biological process,response to stimulus,cell communication,cell differentiation	protein binding,metal ion binding,nucleotide binding,catalytic activity	NADPH--cytochrome P450 reductase
IPI00029750.1	RPS24		X		cytoplasm,ribosome,organelle lumen,cytosol,nucleus	cell organization and biogenesis,metabolic process,transport,reproduction	protein binding,nucleotide binding,structural molecule activity	Isoform 1 of 40S ribosomal protein S24
IPI00026994.3	PRAF2		X		membrane,cytoplasm,endosome	transport		PRA1 family protein 2
IPI00333763.7	GLRX5		X		mitochondrion,cytoplasm,nucleus	development,regulation of biological process,cellular homeostasis	metal ion binding,catalytic activity	Glutaredoxin-related protein 5, mitochondrial
IPI00334190.4	STOML2	X	X		cytoskeleton,mitochondrion,membrane,cytoplasm,nucleus		protein binding	Stomatin-like protein 2
IPI00333140.8	DNER			X	cell surface,membrane,cytoplasm,endosome	development,cell organization and biogenesis,metabolic process,transport,regulation of biological process,response to stimulus,cellular component movement,cell communication,cell differentiation	protein binding,signal transducer activity,metal ion binding,receptor activity	Delta and Notch-like epidermal growth factor-related receptor
IPI00027192.6	PLOD1			X	extracellular,endoplasmic reticulum,cytoplasm	metabolic process	metal ion binding,catalytic activity	cDNA, FLJ79184, highly similar to Procollagen-lysine, 2-oxoglutarate 5-dioxygenase 1
IPI00017160.3	VTA1	X	X		membrane,cytoplasm,cytosol,endosome	cell organization and biogenesis,transport		Vacuolar protein sorting-associated protein VTA1 homolog

IPI	GENE	Alzheimer's Hippocampus	Control hippocampus	CSF	Cellular localization	Biological process	Molecular function	Protein Description
IPI00005719.1	RAB1A	X	X	X	endoplasmic reticulum,cytoplasm,Golgi,nucleus	metabolic process,transport,regulation of biological process,response to stimulus,cell communication	nucleotide binding,catalytic activity	Isoform 1 of Ras-related protein Rab-1A
IPI00893541.1	PDIA3			X		metabolic process,regulation of biological process,cellular homeostasis	catalytic activity	14 kDa protein
IPI00291488.3	WFDC2			X	extracellular	metabolic process,regulation of biological process,reproduction	enzyme regulator activity	Isoform 1 of WAP four-disulfide core domain protein 2
IPI00008040.4	PADI1	X			cytoplasm	metabolic process	metal ion binding,catalytic activity	Protein-arginine deiminase type-1
IPI00011515.1	PACSIN1	X	X	X	cytoplasm,Golgi	cell organization and biogenesis,metabolic process,transport,regulation of biological process	protein binding,catalytic activity	Protein kinase C and casein kinase substrate in neurons protein 1
IPI00303207.3	ABCE1		X		mitochondrion,cytoplasm	metabolic process,response to stimulus	nucleotide binding,catalytic activity,enzyme regulator activity	ATP-binding cassette sub-family E member 1
IPI00938364.1	IGHV1-24			X			protein binding	hCG2038918
IPI00377045.4	LAMA3			X	extracellular,cytoskeleton	development,metabolic process,regulation of biological process,cellular component movement	protein binding,motor activity,catalytic activity	Laminin alpha-3 chain variant 1
IPI00031821.1	ITM2B	X	X	X	extracellular,membrane,cytoplasm,Golgi,nucleus,endosome	cell death,development,metabolic process,regulation of biological process	protein binding,nucleotide binding	Integral membrane protein 2B
IPI00008148.2	GFRA1			X	membrane	development,cell organization and biogenesis,regulation of biological process,response to stimulus,cell communication,cell differentiation	signal transducer activity,protein binding,receptor activity	Isoform 1 of GDNF family receptor alpha-1
IPI00008586.3	CSPG5		X	X	membrane,endoplasmic reticulum,cytoplasm,Golgi	development,transport,regulation of biological process,cell communication,cell differentiation	protein binding	Isoform 1 of Chondroitin sulfate proteoglycan 5
IPI00101664.3	NARS2		X		mitochondrion,cytoplasm,organelle lumen	metabolic process	nucleotide binding,catalytic activity	Probable asparaginyl-tRNA synthetase, mitochondrial
IPI00045550.4	PPP1R9B	X	X				protein binding	neurabin-2
IPI00260755.8	ARHGAP40			X	cytoplasm,cytosol	metabolic process,regulation of biological process,response to stimulus,cell communication	enzyme regulator activity	Rho GTPase-activating protein 40
IPI00793443.2	IPO5	X	X		membrane,cytoplasm,organelle lumen,nucleus	transport	protein binding,transporter activity,enzyme regulator activity	Isoform 1 of Importin-5
IPI00064241.4	IKZF4			X	nucleus	metabolic process,regulation of biological process	protein binding,DNA binding,metal ion binding	Isoform 1 of Zinc finger protein Eos
IPI00307749.2	NDUFS7	X	X			metabolic process	catalytic activity	33 kDa protein
IPI00009505.1	SNTB2		X		cytoskeleton,membrane,cytoplasm		protein binding	Isoform 1 of Beta-2-syntrophin
IPI00217617.4	MPP7			X	membrane	cell organization and biogenesis,regulation of biological process,response to stimulus,cell communication	protein binding,structural molecule activity	MAGUK p55 subfamily member 7
IPI00218946.2	HCN2			X	membrane	development,transport,regulation of biological process,response to stimulus,cell communication,cellular homeostasis	protein binding,transporter activity,nucleotide binding,structural molecule activity	Potassium/sodium hyperpolarization-activated cyclic nucleotide-gated channel 2
IPI00009456.1	NT5E	X	X		membrane,cytoplasm	metabolic process,regulation of biological process,response to stimulus,defense response	metal ion binding,nucleotide binding,catalytic activity	5'-nucleotidase
IPI00301465.4	HJURP			X	cytoplasm,organelle lumen,chromosome,nucleus	cell organization and biogenesis,metabolic process,regulation of biological process	protein binding,DNA binding	Isoform 1 of Holliday junction recognition protein
IPI00020956.1	HDGF	X	X		extracellular,cytoplasm,organelle lumen,nucleus	cell proliferation,metabolic process,regulation of biological process,response to stimulus,cell communication	protein binding,DNA binding,nucleotide binding	Hepatoma-derived growth factor
IPI00026958.4	FDXR	X	X		mitochondrion,membrane,cytoplasm,organelle lumen	transport,metabolic process	protein binding,nucleotide binding,catalytic activity	Isoform Short of NADPH:adrenodoxin oxidoreductase, mitochondrial
IPI00220327.4	KRT1	X	X	X	cytoskeleton,membrane	development,metabolic process,transport,regulation of biological process,response to stimulus,defense response,coagulation	protein binding,transporter activity,motor activity,receptor activity,structural molecule activity,catalytic activity	Keratin, type II cytoskeletal 1
IPI00026125.1	DNASE1L1	X	X	X	endoplasmic reticulum,cytoplasm	metabolic process	DNA binding,catalytic activity	Deoxyribonuclease-1-like 1
IPI00410675.1	STX1B	X	X	X	membrane	transport,regulation of biological process,cell communication	protein binding,transporter activity	Syntaxin-1B
IPI00887169.2	IGLV1-44			X			protein binding	Putative uncharacterized protein
IPI00828189.2	PCMT1	X	X		membrane,cytoplasm	metabolic process	catalytic activity	Isoform 2 of Protein-L-isoaspartate(D-aspartate) O-methyltransferase
IPI00022443.1	AFP			X	extracellular,cytoplasm	development,metabolic process,transport,regulation of biological process,response to stimulus,cell communication,reproduction	metal ion binding	Alpha-fetoprotein
IPI00022694.3	PSMD4	X	X		cytoplasm,proteasome,organelle lumen,nucleus,cytosol	cell death,metabolic process,regulation of biological process,response to stimulus,cell communication	protein binding,metal ion binding	Isoform Rpn10A of 26S proteasome non-ATPase regulatory subunit 4
IPI00030847.3	TM9SF3			X	membrane			Transmembrane 9 superfamily member 3

IPI	GENE	Alzheimer's Hippocampus	Control hippocampus	CSF	Cellular localization	Biological process	Molecular function	Protein Description
IPI00784807.1	IGHG2			X			protein binding	IGHG2 protein
IPI00418382.1	OPLAH	X			cytoplasm,cytosol	metabolic process,response to stimulus	nucleotide binding,catalytic activity	5-oxoprolinase
IPI00005859.5	KRT75			X	cytoskeleton		motor activity,structural molecule activity,catalytic activity	Keratin, type II cytoskeletal 75
IPI00003269.1	ACTBL2	X	X	X	cytoskeleton,cytoplasm		nucleotide binding	Beta-actin-like protein 2
IPI00008905.3	UGT2B15			X	membrane,endoplasmic reticulum,cytoplasm	metabolic process,response to stimulus	catalytic activity	UDP-glucuronosyltransferase 2B15
IPI00021924.1	H1FX	X	X		chromosome,nucleus	cell organization and biogenesis,metabolic process	DNA binding	Histone H1x
IPI00384225.4	METRN			X	extracellular	development,cell differentiation		Meteorin
IPI00022630.1	AIF1	X	X		cytoskeleton,membrane,cytoplasm,nucleus,cytosol	cell death,cell proliferation,cell organization and biogenesis,metabolic process,transport,regulation of biological process,response to stimulus,cellular component movement,cell communication,defense response	protein binding,metal ion binding	Allograft inflammatory factor 1
IPI00956602.1	IGLV1-44			X			protein binding	Anti-streptococcal/anti-myosin immunoglobulin lambda light chain variable region (Fragment)
IPI00442073.5	CSRP1	X	X		nucleus		metal ion binding	Cysteine and glycine-rich protein 1
IPI00100796.4	CHMP5	X	X		membrane,cytoplasm,cytosol,nucleus,endosome	cell organization and biogenesis,metabolic process,transport,regulation of biological process	protein binding	Charged multivesicular body protein 5
IPI00007249.3	ENPP4			X	membrane	metabolic process	catalytic activity	Ectonucleotide pyrophosphatase/phosphodiesterase family member 4
IPI00426051.3	LOC100133739			X	membrane		protein binding	Putative uncharacterized protein DKFZp686C15213
IPI00103536.4	MADD	X	X		membrane,cytoplasm	cell death,metabolic process,regulation of biological process,response to stimulus,cell communication	protein binding,enzyme regulator activity	Isoform 1 of MAP kinase-activating death domain protein
IPI00916757.1	DCTN1	X	X		cytoskeleton,membrane,cytoplasm	cell organization and biogenesis	protein binding	dynactin subunit 1 isoform 5
IPI00023020.1	SEMG1		X		extracellular,cytoplasm	reproduction	structural molecule activity	Isoform 1 of Semenogelin-1
IPI00017601.1	CP	X	X	X	extracellular	transport,metabolic process,cellular homeostasis	protein binding,metal ion binding,catalytic activity	Ceruloplasmin
IPI00455667.7	IGLON5		X	X	extracellular		protein binding	IgLON family member 5
IPI00549543.1	NCDN	X	X		cytoplasm,cytosol,nucleus	development,cell organization and biogenesis,regulation of biological process,cell communication,cell differentiation		Isoform 1 of Neurochondrin
IPI00020194.1	TAF15		X				metal ion binding	Isoform Short of TATA-binding protein-associated factor 2N
IPI00026240.2	BST1			X	membrane	development,response to stimulus	nucleotide binding,catalytic activity	ADP-ribosyl cyclase 2
IPI00183603.3	OSTC	X	X		membrane,endoplasmic reticulum,cytoplasm	metabolic process	catalytic activity	Oligosaccharyltransferase complex subunit OSTC
IPI00788189.1	FCGBP			X			protein binding	similar to Fc fragment of IgG binding protein
IPI00027826.6	SLC25A18		X		mitochondrion,membrane,cytoplasm	transport	transporter activity	Mitochondrial glutamate carrier 2
IPI00012075.1	NPPC			X	extracellular	cell proliferation,development,metabolic process,regulation of biological process,response to stimulus,cell communication,cell differentiation	protein binding	C-type natriuretic peptide
IPI00646909.2	TUBA8	X	X		cytoskeleton,cytoplasm	cell organization and biogenesis,metabolic process,cellular component movement	nucleotide binding,structural molecule activity,catalytic activity	Tubulin alpha-8 chain
IPI00783943.2	SUN1	X	X		membrane,nucleus			Uncharacterized protein
IPI00013455.8	CLIP1	X	X	X	cytoskeleton,membrane,mitochondrion,cytoplasm,Golgi,organelle lumen,chromosome,cytosol,nucleus,endosome	cell organization and biogenesis,transport,regulation of biological process,cell differentiation,reproduction	protein binding,transporter activity,metal ion binding,motor activity,catalytic activity	Isoform 1 of CAP-Gly domain-containing linker protein 1
IPI00791901.1	C1R			X		metabolic process	catalytic activity	26 kDa protein
IPI00003648.2	PVRL1	X	X	X	extracellular,membrane	development,cell organization and biogenesis,regulation of biological process,response to stimulus,cell communication,reproduction,cell differentiation	protein binding,signal transducer activity,receptor activity	Isoform Delta of Poliovirus receptor-related protein 1
IPI00155729.6	PLXNB3			X	membrane	development,cell organization and biogenesis,regulation of biological process,response to stimulus,cell communication,cell differentiation	protein binding,receptor activity	Plexin-B3

IPI	GENE	Alzheimer's Hippocampus	Control hippocampus	CSF	Cellular localization	Biological process	Molecular function	Protein Description
IPI00419722.4	SEZ6L2			X	membrane			cDNA FLJ61340, highly similar to Homo sapiens seizure related 6 homolog-like 2 (SEZ6L2), transcript variant 2, mRNA
IPI00018860.1	ULBP2			X	extracellular,cell surface,membrane	regulation of biological process,response to stimulus	signal transducer activity,receptor activity	NKG2D ligand 2
IPI00021439.1	ACTB	X	X	X	cytoskeleton,cytoplasm,organelle lumen,nucleus,cytosol	development,cell organization and biogenesis,metabolic process,response to stimulus,cellular component movement,cell differentiation,coagulation	protein binding,nucleotide binding,structural molecule activity	Actin, cytoplasmic 1
IPI00217975.4	LMNB1	X			cytoskeleton,mitochondrion,membrane,cytoplasm,organelle lumen,nucleus	cell death,cell organization and biogenesis,metabolic process,regulation of biological process	protein binding,motor activity,structural molecule activity,catalytic activity	Lamin-B1
IPI00025277.5	PDCD6	X	X		membrane,endoplasmic reticulum,cytoplasm,nucleus	cell death,metabolic process,transport,regulation of biological process,response to stimulus	protein binding,metal ion binding	Programmed cell death protein 6
IPI00027377.1 4	ACAN	X		X	extracellular	cell organization and biogenesis,development,metabolic process,regulation of biological process,response to stimulus,cellular component movement,cell differentiation	protein binding,metal ion binding	aggrecan core protein isoform 2 precursor
IPI00009826.2	CPB1			X	extracellular	metabolic process	metal ion binding,catalytic activity	Carboxypeptidase B
IPI00328526.6	ARPP19	X	X		cytoplasm	cell organization and biogenesis,transport,metabolic process,cell division,regulation of biological process	protein binding,enzyme regulator activity	Isoform ARPP-19 of cAMP-regulated phosphoprotein 19
IPI00827650.2	CD44			X	membrane			Isoform 3 of CD44 antigen
IPI00101969.9	ARHGAP23	X	X			metabolic process,regulation of biological process,response to stimulus,cell communication	protein binding,enzyme regulator activity	Isoform 1 of Rho GTPase-activating protein 23
IPI00008433.4	RPS5	X	X	X	cytoplasm,ribosome,cytosol	cell organization and biogenesis,transport,metabolic process,regulation of biological process,reproduction	RNA binding,structural molecule activity	40S ribosomal protein S5
IPI00299627.3	DUOX2			X	membrane	development,metabolic process,regulation of biological process,response to stimulus,cell communication,reproduction	antioxidant activity,metal ion binding,catalytic activity	Dual oxidase 2
IPI00258833.1	SNX6	X	X		cytoplasm	cell communication	protein binding	sorting nexin-6 isoform a
IPI00029803.5	MTG1			X	membrane,mitochondrion,cytoplasm,organelle lumen,nucleus	transport,metabolic process	transporter activity,nucleotide binding,catalytic activity	Isoform 1 of Mitochondrial GTPase 1
IPI00216683.6	CDC25C			X		metabolic process	catalytic activity	M-phase inducer phosphatase 3
IPI00012792.2	CDH5			X	membrane	cell proliferation,cell organization and biogenesis,development,regulation of biological process	protein binding,metal ion binding	Cadherin-5
IPI00000130.3	SST			X	extracellular	cell death,cell proliferation,regulation of biological process,response to stimulus,cellular component movement,cell communication	protein binding	Somatostatin
IPI00555812.5	GC	X	X	X	extracellular			vitamin D-binding protein isoform 1 precursor
IPI00044326.3	CHST14			X	membrane,cytoplasm,Golgi	metabolic process	catalytic activity	Carbohydrate sulfotransferase 14
IPI00470805.2	MDC1			X	membrane	reproduction	protein binding	Isoform 2 of Mediator of DNA damage checkpoint protein 1
IPI00412579.6	RPL10A	X	X		cytoplasm,ribosome,cytosol	cell organization and biogenesis,metabolic process,transport,reproduction	RNA binding,structural molecule activity	60S ribosomal protein L10a
IPI00183487.1	XYLT1			X	extracellular,membrane,endoplasmic reticulum,cytoplasm,Golgi	cell organization and biogenesis,development,metabolic process,regulation of biological process,response to stimulus,cell differentiation	catalytic activity	Xylosyltransferase 1
IPI00004358.4	PYGB	X	X		cytoplasm	metabolic process	protein binding,catalytic activity	Glycogen phosphorylase, brain form
IPI00021435.3	PSMC2	X	X		mitochondrion,cytoplasm,proteasome,organelle lumen,cytosol,nucleus	cell death,metabolic process,regulation of biological process,response to stimulus,cell communication	protein binding,nucleotide binding,catalytic activity	26S protease regulatory subunit 7
IPI00218732.4	PON1			X	extracellular,membrane	transport,metabolic process,regulation of biological process,response to stimulus	protein binding,metal ion binding,catalytic activity	Serum paraoxonase/arylesterase 1
IPI00028296.1	CAMK1	X	X		cytoplasm,nucleus	development,cell organization and biogenesis,transport,metabolic process,regulation of biological process,response to stimulus,cell communication,cell differentiation	protein binding,nucleotide binding,catalytic activity	Calcium/calmodulin-dependent protein kinase type 1
IPI00448751.4	KIAA1598	X	X		membrane	regulation of biological process,response to stimulus,cell communication	signal transducer activity,receptor activity	Isoform 3 of Shootin-1
IPI00002525.3	NENF			X	extracellular	regulation of biological process,response to stimulus,cell communication	protein binding,metal ion binding	Neudesin
IPI00218108.1	NDRG2		X					Isoform 2 of Protein NDRG2

IPI	GENE	Alzheimer's Hippocampus	Control hippocampus	CSF	Cellular localization	Biological process	Molecular function	Protein Description
IPI00643720.3	OGDHL		X		mitochondrion,cytoplasm,organelle lumen	metabolic process	protein binding,catalytic activity	Isoform 1 of 2-oxoglutarate dehydrogenase-like, mitochondrial
IPI00290397.8	PPP1R14C		X		membrane,cytoplasm	metabolic process,regulation of biological process	enzyme regulator activity	Protein phosphatase 1 regulatory subunit 14C
IPI00002459.4	ANXA6	X	X	X			metal ion binding	Uncharacterized protein
IPI00643667.1	C1QTNF3			X				complement C1q tumor necrosis factor-related protein 3 isoform b
IPI00235647.9	FBLL1			X	organelle lumen,nucleus	metabolic process	RNA binding,catalytic activity	rRNA/tRNA 2'-O-methyltransferase fibrillarin-like protein 1
IPI00643041.3	RAN	X	X		membrane,cytoplasm,organelle lumen,chromosome,cytosol,nucleus	cell organization and biogenesis,cell division,transport,metabolic process,regulation of biological process,response to stimulus,cell communication,reproduction	protein binding,nucleotide binding,catalytic activity	GTP-binding nuclear protein Ran
IPI00009822.1	SRP54	X	X		cytoplasm,organelle lumen,cytosol,nucleus	cell organization and biogenesis,transport,metabolic process,response to stimulus	RNA binding,nucleotide binding,catalytic activity	Signal recognition particle 54 kDa protein
IPI00024818.3	USPL1			X		metabolic process	catalytic activity	Isoform 1 of Ubiquitin-specific peptidase-like protein 1
IPI00876888.1	LOC100290146			X	membrane		protein binding	cDNA FLJ78387
IPI00382515.1	LOC100129447			X	membrane	metabolic process,response to stimulus	RNA binding,nucleotide binding,catalytic activity	Similar to SubName: Full=Predicted protein;
IPI00289083.5	CACHD1			X	membrane	transport	protein binding	Isoform 1 of VWFA and cache domain-containing protein 1
IPI00022395.1	C9			X	extracellular,membrane	cell death,metabolic process,regulation of biological process,response to stimulus,defense response,coagulation	protein binding	Complement component C9
IPI00171611.7	HIST2H3D			X	extracellular,organelle lumen,chromosome,nucleus	cell organization and biogenesis,metabolic process,response to stimulus,coagulation	protein binding,DNA binding	Histone H3.2
IPI00027009.2	PACSIN2	X	X	X	cytoplasm,cytosol	cell organization and biogenesis,transport,regulation of biological process	protein binding,transporter activity	Isoform 1 of Protein kinase C and casein kinase substrate in neurons protein 2
IPI00107886.7	SEMA6B			X	membrane	development	protein binding,receptor activity	Semaphorin 6B isoform 2
IPI00924639.1	AASS		X			metabolic process	nucleotide binding,catalytic activity	102 kDa protein
IPI00044751.6	KIF20B			X	cytoskeleton,membrane	cellular component movement	motor activity,nucleotide binding,catalytic activity	Uncharacterized protein
IPI00032187.6	NISCH	X	X	X	membrane,cytoplasm,cytosol,endosome	cell death,cell organization and biogenesis,transport,metabolic process,regulation of biological process,response to stimulus,cellular component movement,cell communication	protein binding,signal transducer activity,receptor activity	Isoform 1 of Nischarin
IPI00742682.2	TPR	X	X		cytoskeleton,membrane,cytoplasm,organelle lumen,chromosome,nucleus	cell organization and biogenesis,metabolic process,transport,regulation of biological process,response to stimulus,cell communication	protein binding,transporter activity,nucleotide binding,motor activity,catalytic activity	Nucleoprotein TPR
IPI00916823.1	TUBA4A	X	X		cytoskeleton	cell organization and biogenesis,cellular component movement	nucleotide binding,structural molecule activity	Uncharacterized protein
IPI00000581.6	OTUB1	X	X			metabolic process	catalytic activity	cDNA FLJ56307, highly similar to Ubiquitin thioesterase protein OTUB1
IPI00010810.1	ETFA	X	X	X	mitochondrion,cytoplasm,organelle lumen	metabolic process,transport	catalytic activity	Electron transfer flavoprotein subunit alpha, mitochondrial
IPI00335355.3	SLC6A17	X	X		membrane,cytoplasm	transport	transporter activity	Sodium-dependent neutral amino acid transporter SLC6A17
IPI00007189.1	CDC42	X	X			regulation of biological process,response to stimulus,cell communication	nucleotide binding	Isoform 1 of Cell division control protein 42 homolog
IPI00480159.1	INPP5F			X			catalytic activity	Isoform 1 of Phosphatidylinositide phosphatase SAC2
IPI00029050.2	LARGE			X	membrane,cytoplasm,Golgi	metabolic process,cellular homeostasis	catalytic activity	Isoform 1 of Glycosyltransferase-like protein LARGE1
IPI00010405.4	ROR1			X	membrane,cytoplasm	metabolic process,regulation of biological process,response to stimulus,cell communication	protein binding,signal transducer activity,nucleotide binding,receptor activity,catalytic activity	Isoform Long of Tyrosine-protein kinase transmembrane receptor ROR1
IPI00019794.1<br>0	SHANK3		X		cytoskeleton,membrane	development,cell organization and biogenesis,transport,regulation of biological process,response to stimulus,defense response,cell communication,cellular homeostasis,cell differentiation	protein binding,metal ion binding	SH3 and multiple ankyrin repeat domains protein 3
IPI00032230.2	EPB41L3	X	X		cytoskeleton,membrane,cytoplasm	cell organization and biogenesis	protein binding,structural molecule activity	Isoform A of Band 4.1-like protein 3
IPI00478986.3	RPS4XP21			X	cytoplasm,ribosome	metabolic process	structural molecule activity	30 kDa protein
IPI00289837.4	CCDC85A			X				Coiled-coil domain-containing protein 85A

IPI	GENE	Alzheimer's Hippocampus	Control hippocampus	CSF	Cellular localization	Biological process	Molecular function	Protein Description
IPI00658045.3	SLC2A13		X		membrane	transport	transporter activity	Proton myo-inositol cotransporter
IPI00246975.8	GSTM3	X	X		cytoplasm	development,metabolic process,response to stimulus,cell differentiation	protein binding,catalytic activity	Glutathione S-transferase Mu 3
IPI00017855.1	ACO2	X	X		mitochondrion,cytoplasm,organelle lumen,nucleus	cell death,metabolic process	metal ion binding,catalytic activity	Aconitate hydratase, mitochondrial
IPI00015346.1	CELSR2			X	membrane,cytoplasm	development,cell organization and biogenesis,metabolic process,regulation of biological process,response to stimulus,cellular component movement,cell communication,cell differentiation,reproduction	protein binding,signal transducer activity,metal ion binding,receptor activity	Cadherin EGF LAG seven-pass G-type receptor 2
IPI00021855.1	APOC1		X	X	extracellular,endoplasmic reticulum,cytoplasm	cell organization and biogenesis,metabolic process,transport,regulation of biological process	enzyme regulator activity	Apolipoprotein C-I
IPI00294004.1	PROS1			X	extracellular,cell surface,membrane,endoplasmic reticulum,cytoplasm,Golgi,organelle lumen	metabolic process,transport,regulation of biological process,response to stimulus,cellular component movement,defense response,coagulation	protein binding,metal ion binding,enzyme regulator activity	Vitamin K-dependent protein S
IPI00386258.1	MTCH1	X			membrane,mitochondrion,cytoplasm	cell death,development,cell organization and biogenesis,metabolic process,transport,regulation of biological process,response to stimulus,cell communication,cell differentiation	protein binding	Isoform 1 of Mitochondrial carrier homolog 1
IPI00010346.1	NLN	X		X	mitochondrion,cytoplasm	metabolic process	metal ion binding,catalytic activity	Neurolysin, mitochondrial
IPI00604624.1	NIF3L1	X	X		mitochondrion,cytoplasm	metabolic process,regulation of biological process	protein binding	Isoform 1 of NIF3-like protein 1
IPI00413826.2	H3F3AP6			X	chromosome	cell organization and biogenesis,metabolic process	DNA binding	similar to H3 histone, family 3B
IPI00018098.4	PRPF38B			X	spliceosomal complex,nucleus	metabolic process		Isoform 1 of Pre-mRNA-splicing factor 38B
IPI00410079.3	FAM82A2	X	X	X	cytoskeleton,membrane,mitochondrion,cytoplasm,nucleus	cell death,cellular homeostasis,cell differentiation	protein binding	Isoform 1 of Regulator of microtubule dynamics protein 3
IPI00011899.1	BAMBI			X	membrane,cytoplasm	cell proliferation,cell organization and biogenesis,development,transport,metabolic process,regulation of biological process,response to stimulus,cellular component movement,cell communication,cell differentiation	protein binding	BMP and activin membrane-bound inhibitor homolog
IPI00301051.3	NHLRC2	X	X			metabolic process	antioxidant activity,protein binding,catalytic activity	Isoform 1 of NHL repeat-containing protein 2
IPI00018871.2	ARL8B	X	X		cytoplasm,nucleus	regulation of biological process,response to stimulus,cell communication	nucleotide binding	cDNA FLJ56285, highly similar to ADP-ribosylation factor-like protein 8B
IPI00328715.4	MTDH	X	X		membrane,endoplasmic reticulum,cytoplasm,organelle lumen,nucleus	cell death,cell organization and biogenesis,development,metabolic process,regulation of biological process,response to stimulus,cell communication	protein binding	Protein LYRIC
IPI00005517.1	EFNA5			X	cell surface,membrane	cell death,cell organization and biogenesis,development,transport,metabolic process,regulation of biological process,response to stimulus,cell communication,cellular homeostasis,cell differentiation	protein binding	Ephrin-A5
IPI00004838.2	CRK	X	X	X	membrane,cytoplasm,cytosol,nucleus,endosome	cell organization and biogenesis,metabolic process,regulation of biological process,response to stimulus,cell communication,coagulation	protein binding	Isoform Crk-II of Adapter molecule crk
IPI00300096.4	RAB35	X	X		extracellular,membrane,mitochondrion,cytoplasm,nucleus,endosome	cell division,metabolic process,transport,regulation of biological process,response to stimulus,cell communication	nucleotide binding,catalytic activity	Ras-related protein Rab-35
IPI00221034.9	RELB			X	cytoskeleton,cytoplasm,cytosol,nucleus	development,metabolic process,regulation of biological process,response to stimulus,cell differentiation	protein binding,DNA binding	Transcription factor RelB
IPI00979245.1	CYFIP2		X		membrane			unnamed protein product
IPI00329352.4	NOMO1	X	X	X	membrane		catalytic activity	Nodal modulator 1
IPI00012391.3	APC			X	cytoskeleton,membrane,cytoplasm,chromosome,nucleus,cytosol	cell death,development,cell organization and biogenesis,metabolic process,regulation of biological process,response to stimulus,reproduction,cell proliferation,cell division,cellular component movement,cell communication,cellular homeostasis,cell differentiation	protein binding,enzyme regulator activity	Isoform Long of Adenomatous polyposis coli protein
IPI00217600.6	PNPLA6	X	X			metabolic process		Isoform 2 of Neuropathy target esterase

IPI	GENE	Alzheimer's Hippocampus	Control hippocampus	CSF	Cellular localization	Biological process	Molecular function	Protein Description
IPI00007792.1	FGF1	X	X		extracellular,cytoplasm,organelle lumen,cytosol,nucleus	cell proliferation,development,cell division,metabolic process,regulation of biological process,response to stimulus,cellular component movement,cell communication,cell differentiation	protein binding	Isoform 1 of Heparin-binding growth factor 1
IPI00005107.2	NPC1			X	membrane,endoplasmic reticulum,cytoplasm,Golgi,vacuole,nucleus,endosome	metabolic process,transport,regulation of biological process,response to stimulus,cell communication	protein binding,signal transducer activity,transporter activity,receptor activity	Niemann-Pick C1 protein
IPI00024934.5	MUT		X		mitochondrion,cytoplasm,organelle lumen	development,metabolic process	metal ion binding,catalytic activity	Methylmalonyl-CoA mutase, mitochondrial
IPI00409640.3	LSR			X	extracellular,membrane	development,metabolic process,regulation of biological process	protein binding,receptor activity	Isoform 1 of Lipolysis-stimulated lipoprotein receptor
IPI00414249.5	NRXN3			X	membrane	development,cell organization and biogenesis,response to stimulus,cell differentiation	protein binding,metal ion binding,receptor activity	Isoform 1a of Neurexin-3-alpha
IPI00002993.1	TAF9			X	organelle lumen,nucleus	cell death,cell organization and biogenesis,metabolic process,regulation of biological process,response to stimulus,cell growth	protein binding,DNA binding,catalytic activity	Transcription initiation factor TFIID subunit 9
IPI00027769.1	ELANE			X	extracellular,cell surface,cytoplasm	cell proliferation,cell organization and biogenesis,transport,metabolic process,regulation of biological process,response to stimulus,cellular component movement,defense response,cell communication,cellular homeostasis	protein binding,catalytic activity	Neutrophil elastase
IPI00374590.4	CASC4			X	membrane			protein CASC4 isoform a
IPI00009328.4	EIF4A3		X		spliceosomal complex,cytoplasm,organelle lumen,nucleus,cytosol	metabolic process,transport,regulation of biological process,response to stimulus,cell communication	protein binding,RNA binding,nucleotide binding,catalytic activity	Eukaryotic initiation factor 4A-III
IPI00922914.2	EPB41L3		X		cytoskeleton,membrane,cytoplasm	cell organization and biogenesis	protein binding,structural molecule activity	Uncharacterized protein
IPI00386393.5	EXOC6B			X	cytoplasm	transport		unnamed protein product
IPI00007221.2	SERPINA5			X	extracellular,cell surface,membrane,cytoplasm	cell organization and biogenesis,metabolic process,transport,regulation of biological process,reproduction	protein binding,enzyme regulator activity	Plasma serine protease inhibitor
IPI00007673.4	CHCHD2	X	X		mitochondrion,cytoplasm			Coiled-coil-helix-coiled-coil-helix domain-containing protein 2, mitochondrial
IPI00298285.1	ERBB3			X	extracellular,membrane,nucleus	cell death,cell proliferation,cell organization and biogenesis,development,metabolic process,transport,regulation of biological process,response to stimulus,cell communication,cell differentiation	protein binding,signal transducer activity,nucleotide binding,receptor activity,catalytic activity,enzyme regulator activity	Isoform 1 of Receptor tyrosine-protein kinase erbB-3
IPI00216356.2	RASAL1	X	X		membrane	regulation of biological process,response to stimulus,cell communication	protein binding,enzyme regulator activity	Isoform 2 of RasGAP-activating-like protein 1
IPI00290770.3	CCT3	X	X			metabolic process	protein binding,nucleotide binding	Uncharacterized protein
IPI00220741.8	SPTA1	X	X	X	cytoskeleton,membrane,cytoplasm,cytosol	cell proliferation,cell organization and biogenesis,development,metabolic process,regulation of biological process,response to stimulus,cell differentiation	protein binding,metal ion binding,structural molecule activity	Isoform 1 of Spectrin alpha chain, erythrocyte
IPI00008234.3	CYB5R2	X	X		membrane,mitochondrion,cytoplasm	metabolic process	catalytic activity	Isoform 1 of NADH-cytochrome b5 reductase 2
IPI00007145.4	RAB11FIP2		X		organelle lumen,nucleus		protein binding	Uncharacterized protein
IPI00013569.4	PAPPA2			X	extracellular,membrane	cell organization and biogenesis,metabolic process,regulation of biological process,cell growth,cell differentiation	protein binding,metal ion binding,catalytic activity	Isoform 1 of Pappalysin-2
IPI00465234.2	CSF2RB			X	membrane	regulation of biological process,response to stimulus,cell communication	signal transducer activity,protein binding,receptor activity	Isoform 1 of Cytokine receptor common subunit beta
IPI00329685.9	ARSA	X	X	X		metabolic process	catalytic activity	arylsulfatase A, isoform CRA_a
IPI00025252.1	PDIA3	X	X	X	endoplasmic reticulum,cytoplasm,organelle lumen	cell death,transport,metabolic process,regulation of biological process,response to stimulus,cell communication,cellular homeostasis	protein binding,catalytic activity	Protein disulfide-isomerase A3
IPI00219219.3	LGALS1	X	X	X	extracellular,cell surface,cytoplasm,nucleus	cell death,cell organization and biogenesis,development,regulation of biological process,response to stimulus,cell communication,cellular homeostasis,cell differentiation	signal transducer activity,protein binding	Galectin-1
IPI00007856.1	MYH2			X	cytoskeleton,membrane,cytoplasm,Golgi	cell organization and biogenesis,metabolic process,response to stimulus,cellular component movement	protein binding,motor activity,nucleotide binding,structural molecule activity,catalytic activity	Myosin-2
IPI00257882.7	PEPD	X	X	X		metabolic process	metal ion binding,catalytic activity	Xaa-Pro dipeptidase

IPI	GENE	Alzheimer's Hippocampus	Control hippocampus	CSF	Cellular localization	Biological process	Molecular function	Protein Description
IPI00888280.3	LOC100133944			X	membrane			IgGFc-binding protein-like
IPI00297252.6	SULF2			X	extracellular,cell surface,membrane,endoplasmic reticulum,cytoplasm,Golgi	cell organization and biogenesis,development,metabolic process,regulation of biological process,response to stimulus,cell communication,cell differentiation	metal ion binding,catalytic activity	Isoform 1 of Extracellular sulfatase Sulf-2
IPI00018914.2	PTPN14			X	cytoskeleton,cytoplasm	development,metabolic process	protein binding,catalytic activity	Tyrosine-protein phosphatase non-receptor type 14
IPI00829980.2	MSTN			X			protein binding	myosin-reactive immunoglobulin light chain variable region
IPI00479708.6	IGHM	X	X	X			protein binding	Full-length cDNA clone CS0DD006YL02 of Neuroblastoma of Homo sapiens
IPI00556079.3	MOG	X	X		membrane	development,regulation of biological process,response to stimulus,cell communication	protein binding	Isoform 1 of Myelin-oligodendrocyte glycoprotein
IPI00152182.1	KLHDC4			X			protein binding	Isoform 1 of Kelch domain-containing protein 4
IPI00006644.4	PLXNB1	X	X	X	membrane		protein binding	Isoform 2 of Plexin-B1
IPI00000779.1	ADAM22	X	X	X	membrane	development,metabolic process,regulation of biological process,response to stimulus,cell communication,cellular homeostasis,cell differentiation	protein binding,metal ion binding,receptor activity,catalytic activity	disintegrin and metalloproteinase domain-containing protein 22 isoform 1 preproprotein
IPI00549972.3	LIMD2			X			metal ion binding	LIM domain-containing protein 2
IPI00646304.4	PPIB	X	X	X	endoplasmic reticulum,cytoplasm,organelle lumen	metabolic process	protein binding,catalytic activity	Peptidyl-prolyl cis-trans isomerase B
IPI00025318.1	SH3BGRL	X	X	X	cytoplasm,nucleus	regulation of biological process,cellular homeostasis	protein binding,catalytic activity	SH3 domain-binding glutamic acid-rich-like protein
IPI00005038.1	HRSP12	X	X	X	mitochondrion,cytoplasm,organelle lumen,nucleus	cell organization and biogenesis,metabolic process,regulation of biological process	catalytic activity	Ribonuclease UK114
IPI00647217.2	SKIV2L2			X	spliceosomal complex,organelle lumen,nucleus	metabolic process	protein binding,DNA binding,nucleotide binding,catalytic activity	Superkiller viralicidic activity 2-like 2
IPI00025285.3	ATP6V1G1	X	X		membrane,cytoplasm,vacuole,cytosol	transport,metabolic process,regulation of biological process,response to stimulus,cell communication,cellular homeostasis	protein binding,transporter activity,catalytic activity	V-type proton ATPase subunit G 1
IPI00012044.2	NRG3			X	extracellular,membrane	cell organization and biogenesis,metabolic process,regulation of biological process,response to stimulus,cell communication,cell growth	protein binding,enzyme regulator activity	Isoform 1 of Pro-neuregulin-3, membrane-bound isoform
IPI00168866.6	MDGA1			X	membrane	development,cellular component movement,cell differentiation	protein binding	Isoform 1 of MAM domain-containing glycosylphosphatidylinositol anchor protein 1
IPI00001869.3	PAPPA			X	extracellular,membrane	metabolic process,reproduction,cell differentiation	metal ion binding,catalytic activity	Pappalysin-1
IPI00385631.7	ZZEF1	X	X				metal ion binding	Isoform 1 of Zinc finger ZZ-type and EF-hand domain-containing protein 1
IPI00012585.1	HEXB	X	X	X	membrane,cytoplasm,vacuole,organelle lumen	cell death,cell organization and biogenesis,development,metabolic process,regulation of biological process,response to stimulus,cellular component movement,cell communication,cellular homeostasis,reproduction,cell differentiation	protein binding,catalytic activity	Beta-hexosaminidase subunit beta
IPI00747142.2	AGAP9			X		metabolic process,regulation of biological process	protein binding,metal ion binding,nucleotide binding,catalytic activity,enzyme regulator activity	Arf-GAP with GTPase, ANK repeat and PH domain-containing protein 9
IPI00890829.2	MYEOV2	X	X					Isoform 1 of Myeloma-overexpressed gene 2 protein
IPI00298558.2	PDCD10		X		membrane,cytoplasm,Golgi,cytosol	cell death,cell proliferation,development,metabolic process,regulation of biological process,response to stimulus,cell communication	protein binding	Programmed cell death protein 10
IPI00299010.4	SPG7		X		mitochondrion,membrane,cytoplasm	cell death,cell organization and biogenesis,development,metabolic process,transport,response to stimulus,cellular component movement	protein binding,metal ion binding,nucleotide binding,catalytic activity	Isoform 1 of Paraplegin
IPI00163724.2	HCN3			X	membrane	transport,regulation of biological process,cell communication	transporter activity,nucleotide binding	Potassium/sodium hyperpolarization-activated cyclic nucleotide-gated channel 3
IPI00162329.2	TMEM25			X	extracellular,membrane		protein binding	Isoform 1 of Transmembrane protein 25
IPI00019157.3	CSPG4			X	cell surface,membrane	cell proliferation,development,metabolic process,regulation of biological process,response to stimulus,cellular component movement,cell communication,cell differentiation	protein binding,signal transducer activity	Chondroitin sulfate proteoglycan 4

IPI	GENE	Alzheimer's Hippocampus	Control hippocampus	CSF	Cellular localization	Biological process	Molecular function	Protein Description
IPI00293748.3	MINPP1			X	endoplasmic reticulum,cytoplasm,organelle lumen	development,metabolic process	catalytic activity	Isoform 1 of Multiple inositol polyphosphate phosphatase 1
IPI00147874.1	NANS	X	X		cytoplasm,cytosol	metabolic process	catalytic activity	Sialic acid synthase
IPI00465373.2	CCBL2		X		mitochondrion,cytoplasm	metabolic process	protein binding,catalytic activity	Isoform 1 of Kynurenine--oxoglutarate transaminase 3
IPI00012058.1	BDNF			X	extracellular,cytoplasm	cell death,cell proliferation,cell organization and biogenesis,development,metabolic process,transport,regulation of biological process,response to stimulus,cell communication,cell differentiation	protein binding	Isoform 1 of Brain-derived neurotrophic factor
IPI00789245.1	SLC22A23			X	membrane	transport	transporter activity	Isoform 2 of Solute carrier family 22 member 23
IPI00183695.9	S100A10	X			membrane	regulation of biological process,response to stimulus,cell communication	protein binding,metal ion binding	Protein S100-A10
IPI00334410.1	MYO18A	X	X		cytoskeleton,mitochondrion,membrane,cytoplasm,organelle lumen,chromosome,nucleus	cell organization and biogenesis,metabolic process,transport,regulation of biological process,cell differentiation,reproduction	protein binding,transporter activity,nucleotide binding,motor activity,catalytic activity	Isoform 2 of Myosin-XVIIIa
IPI00163446.4	IGHD		X	X				Isoform 2 of Ig delta chain C region
IPI00789234.3	VSTM2A			X				Isoform 2 of V-set and transmembrane domain-containing protein 2A
IPI00101645.3	AHCYL2	X	X			transport,metabolic process	catalytic activity	Isoform 1 of Putative adenosylhomocysteinase 3
IPI00792375.1	ALDOC	X	X			metabolic process	catalytic activity	Fructose-bisphosphate aldolase
IPI00829699.2	IGKC			X	extracellular,membrane	metabolic process,regulation of biological process,response to stimulus,defense response	protein binding	Ig kappa chain V-I region HK101 (Fragment)
IPI00002790.3	SEL1L		X	X	membrane,endoplasmic reticulum,cytoplasm	regulation of biological process,response to stimulus,cell communication	protein binding	Isoform 1 of Protein sel-1 homolog 1
IPI00005824.1	LIN7A		X		cytoskeleton,membrane	cell organization and biogenesis,transport	protein binding	Protein lin-7 homolog A
IPI00385791.1	MRPS26			X			metal ion binding	Serologically defined breast cancer antigen NY-BR-87 (Fragment)
IPI00384646.7	C22orf25	X	X					Isoform 3 of Uncharacterized protein C22orf25
IPI00031666.6	SELO	X			extracellular	metabolic process	catalytic activity	Selenoprotein O
IPI00298946.1	DSCR3		X		membrane,nucleus	transport		Down syndrome critical region protein 3
IPI00219097.4	HMGB2	X			extracellular,cytoplasm,organelle lumen,chromosome,nucleus	cell death,cell proliferation,development,cell organization and biogenesis,metabolic process,regulation of biological process,response to stimulus,cellular component movement,cell communication,reproduction,cell differentiation	protein binding,DNA binding	High mobility group protein B2
IPI00783604.3	EPHA6			X	membrane	metabolic process,regulation of biological process,response to stimulus,cell communication	signal transducer activity,nucleotide binding,receptor activity,catalytic activity	EPA6
IPI00328260.4	NSMAF			X	cytoplasm	metabolic process,regulation of biological process,response to stimulus,cell communication	signal transducer activity,protein binding	Protein FAN
IPI00003176.1	HTRA1			X	extracellular,cytoplasm,cytosol	cell organization and biogenesis,metabolic process,regulation of biological process,response to stimulus,cell communication,cell growth	protein binding,catalytic activity	Serine protease HTRA1
IPI00172450.2	CAMK2G			X		metabolic process	protein binding,nucleotide binding,catalytic activity	Isoform 4 of Calcium/calmodulin-dependent protein kinase type II subunit gamma
IPI00444842.1	LOC100131101			X				cDNA FLJ45125 fis, clone BRAWH3036561
IPI00009294.1	CRIM1			X	extracellular,membrane	development,cell organization and biogenesis,metabolic process,regulation of biological process,response to stimulus,cell communication,cell growth	protein binding,signal transducer activity,receptor activity,catalytic activity,enzyme regulator activity	Cysteine-rich motor neuron 1 protein
IPI00002375.3	TMOD1	X	X		cytoskeleton,membrane,cytoplasm,cytosol,nucleus	cell organization and biogenesis,cellular component movement,cell differentiation	protein binding	Tropomodulin-1
IPI00219042.1	PAM			X		metabolic process	protein binding,metal ion binding,catalytic activity	Isoform 3 of Peptidyl-glycine alpha-amidating monooxygenase
IPI00013940.4	SYNGR1	X	X		membrane,cytoplasm	transport,regulation of biological process,cell communication		Isoform 1A of Synaptogyrin-1
IPI00791316.1	GAP43	X	X			regulation of biological process	protein binding	neuromodulin isoform 1
IPI00290358.2	C16orf91			X	membrane			Protein CCSMST1

IPI	GENE	Alzheimer's Hippocampus	Control hippocampus	CSF	Cellular localization	Biological process	Molecular function	Protein Description
IPI00219913.10	USP14	X	X		cell surface,membrane,cytoplasm,proteasome	metabolic process,regulation of biological process,response to stimulus,cell communication	protein binding,catalytic activity,enzyme regulator activity	Ubiquitin carboxyl-terminal hydrolase 14
IPI00217466.3	HIST1H1D			X	organelle lumen,chromosome,nucleus	cell organization and biogenesis,metabolic process	DNA binding	Histone H1.3
IPI00029737.1	ACSL4		X		membrane,mitochondrion,endoplasmic reticulum,cytoplasm	cell organization and biogenesis,development,metabolic process,regulation of biological process,response to stimulus,reproduction,cell differentiation,cell growth	nucleotide binding,catalytic activity	Isoform Long of Long-chain-fatty-acid--CoA ligase 4
IPI00217561.2	ITGB1	X	X		membrane	regulation of biological process,response to stimulus,cell communication	protein binding,receptor activity	Isoform Beta-1C of Integrin beta-1
IPI00184363.5	GLTP	X	X		membrane,cytoplasm	transport	transporter activity	Glycolipid transfer protein
IPI00374732.4	PPIAP19			X		metabolic process	catalytic activity	similar to peptidylprolyl isomerase A-like
IPI00031534.3	ST6GALNAC1			X	membrane,cytoplasm,Golgi	metabolic process	catalytic activity	Alpha-N-acetylgalactosaminide alpha-2,6-sialyltransferase 1
IPI00790775.1	CACNA2D3			X			protein binding	Isoform 3 of Voltage-dependent calcium channel subunit alpha-2/delta-3
IPI00249982.4	DIDO1			X		metabolic process	protein binding	Isoform 1 of Death-inducer obliterator 1
IPI00005793.2	AP3B2	X	X		membrane,cytoplasm,Golgi,organelle lumen,nucleus	transport	transporter activity	AP-3 complex subunit beta-2
IPI00031557.2	CTH	X	X		cytoplasm,cytosol,nucleus	cell death,cell proliferation,cell organization and biogenesis,metabolic process,regulation of biological process,response to stimulus,cell communication,cell growth	protein binding,catalytic activity	Isoform 1 of Cystathionine gamma-lyase
IPI00019997.1	LIN7C	X	X		cytoskeleton,membrane	transport	protein binding	Protein lin-7 homolog C
IPI00472345.1	IGHG3			X			protein binding	IGHG3 protein
IPI00021447.1	AMY2B			X	extracellular	metabolic process	metal ion binding,catalytic activity	Alpha-amylase 2B
IPI00220102.1	DNAJB2	X	X		proteasome	cell proliferation,cell organization and biogenesis,metabolic process,regulation of biological process,response to stimulus,cell growth	protein binding	Isoform 3 of DnaJ homolog subfamily B member 2
IPI00941398.2	ITSN2		X		membrane,cytoplasm	regulation of biological process,response to stimulus,cell communication	protein binding,enzyme regulator activity	Isoform 2 of Intersectin-2
IPI00028082.1	RECK			X	membrane	cell organization and biogenesis,development,metabolic process,transport,regulation of biological process,reproduction	protein binding,transporter activity,enzyme regulator activity	Reversion-inducing cysteine-rich protein with Kazal motifs
IPI00026944.3	NID1		X	X	extracellular,cell surface,membrane	cell organization and biogenesis,development,regulation of biological process	protein binding,metal ion binding	Isoform 1 of Nidogen-1
IPI00328488.7	MAN2B2			X	extracellular	metabolic process	metal ion binding,catalytic activity	Isoform 1 of Epididymis-specific alpha-mannosidase
IPI00017704.3	COTL1	X	X	X	cytoskeleton,cytoplasm	response to stimulus,defense response	protein binding	Coactosin-like protein
IPI00937278.2	PSMD8	X	X		proteasome,cytoplasm,organelle lumen,cytosol,nucleus	cell death,metabolic process,regulation of biological process,response to stimulus,cell communication		26S proteasome non-ATPase regulatory subunit 8
IPI00006034.1	CRIP2	X	X	X		cell proliferation,development,regulation of biological process	metal ion binding	Cysteine-rich protein 2
IPI00011200.5	PHGDH	X	X		cytoplasm,cytosol	development,cell organization and biogenesis,metabolic process,regulation of biological process,cell differentiation	nucleotide binding,catalytic activity	D-3-phosphoglycerate dehydrogenase
IPI00017256.6	RSU1	X	X	X	cytoplasm,cytosol	cell organization and biogenesis,regulation of biological process,response to stimulus,cell communication	protein binding	Ras suppressor protein 1
IPI00032258.4	C4A			X	extracellular,membrane	metabolic process,regulation of biological process,response to stimulus,defense response	protein binding,enzyme regulator activity	Complement C4-A
IPI00026570.2	COX7A2	X	X	X	membrane,mitochondrion,cytoplasm		transporter activity,catalytic activity	cytochrome c oxidase subunit 7A2, mitochondrial precursor
IPI00000044.1	PDGFB			X	extracellular,cell surface,membrane,endoplasmic reticulum,cytoplasm,Golgi,organelle lumen	cell organization and biogenesis,development,metabolic process,regulation of biological process,response to stimulus,cell proliferation,transport,cell division,cellular component movement,cell communication,cell differentiation,cell growth,coagulation	protein binding,enzyme regulator activity	Platelet-derived growth factor subunit B
IPI00553067.4	CCDC132		X					Isoform 1 of Coiled-coil domain-containing protein 132

IPI	GENE	Alzheimer's Hippocampus	Control hippocampus	CSF	Cellular localization	Biological process	Molecular function	Protein Description
IPI00013302.12	ADAM15			X		metabolic process	metal ion binding,catalytic activity	Isoform 2 of Disintegrin and metalloproteinase domain-containing protein 15
IPI00296190.4	FAM213A	X	X		mitochondrion,cytoplasm	development,metabolic process,regulation of biological process,cell differentiation	antioxidant activity,catalytic activity	UPF0765 protein C10orf58
IPI00973474.2	IGHG3			X	membrane		protein binding	Putative uncharacterized protein
IPI00016589.5	USP19	X	X		membrane,endoplasmic reticulum,cytoplasm	development,metabolic process,regulation of biological process,response to stimulus	protein binding,metal ion binding,catalytic activity	Isoform 1 of Ubiquitin carboxyl-terminal hydrolase 19
IPI00006663.1	ALDH2	X	X		mitochondrion,cytoplasm,organelle lumen	metabolic process,response to stimulus,cell communication	catalytic activity	Aldehyde dehydrogenase, mitochondrial
IPI00854745.2	LOC100130811			X			protein binding	similar to hCG2038921
IPI00939159.6	CAP1	X	X			cell organization and biogenesis	protein binding	Adenylyl cyclase-associated protein
IPI00103994.4	LARS		X		cytoplasm,cytosol	metabolic process,regulation of biological process	protein binding,nucleotide binding,catalytic activity	Leucyl-tRNA synthetase, cytoplasmic
IPI00016621.7	AP2A2			X	membrane,cytoplasm	transport		Isoform 2 of AP-2 complex subunit alpha-2
IPI00289147.5	HHATL		X		membrane,endoplasmic reticulum,cytoplasm	metabolic process,regulation of biological process		Protein-cysteine N-palmitoyltransferase HHAT-like protein
IPI00292791.8	CNTN3			X	membrane	development	protein binding	Contactin-3
IPI00337556.1	MDP1	X				metabolic process	protein binding,metal ion binding,catalytic activity	Isoform 1 of Magnesium-dependent phosphatase 1
IPI00163849.2	EPS15L1	X	X		cytoskeleton,membrane,cytoplasm,Golgi,organelle lumen,chromosome,nucleus	cell organization and biogenesis,metabolic process,transport,regulation of biological process,cell differentiation,reproduction	protein binding,transporter activity,nucleotide binding,motor activity,catalytic activity	cDNA FLJ60624, highly similar to Epidermal growth factor receptor substrate 15-like 1
IPI00043810.4	PRRT1			X	membrane	response to stimulus		Isoform 1 of Proline-rich transmembrane protein 1
IPI00020060.2	SLC9A1		X		membrane,endoplasmic reticulum,cytoplasm	cell organization and biogenesis,transport,regulation of biological process,response to stimulus,cell communication,cellular homeostasis,cell differentiation,cell growth	protein binding,transporter activity	Isoform 1 of Sodium/hydrogen exchanger 1
IPI00029468.1	ACTR1A	X	X		cytoskeleton,cytoplasm,cytosol	transport	nucleotide binding	Alpha-centractin
IPI00440221.3	CDK13			X			catalytic activity	Putative uncharacterized protein (Fragment)
IPI00551024.5	DAK	X	X		cytoplasm,cytosol	metabolic process,response to stimulus,defense response	metal ion binding,nucleotide binding,catalytic activity	Bifunctional ATP-dependent dihydroxyacetone kinase/FAD-AMP lyase (cyclizing)
IPI00855846.1	ABRACL	X	X	X				Costars family protein C6orf115
IPI00056314.1	TSR2			X		metabolic process	protein binding	Pre-rRNA-processing protein TSR2 homolog
IPI00329629.6	DNAJC7	X	X		cytoskeleton,cytoplasm,organelle lumen,nucleus	metabolic process	protein binding	DnaJ homolog subfamily C member 7
IPI00171903.2	HNRNPM	X	X		spliceosomal complex,membrane,organelle lumen,nucleus	metabolic process	protein binding,RNA binding,nucleotide binding	Isoform 1 of Heterogeneous nuclear ribonucleoprotein M
IPI00294187.1	PADI2	X	X		cytoplasm	metabolic process	metal ion binding,catalytic activity	Protein-arginine deiminase type-2
IPI01017935.1	IGK@			X			protein binding	IGK@ protein
IPI00291930.7	CLINT1	X	X		membrane,cytoplasm,Golgi,cytosol	cell organization and biogenesis,transport	protein binding	Isoform 1 of Clathrin interactor 1
IPI00186903.4	APOL1			X	extracellular	metabolic process,transport		Isoform 2 of Apolipoprotein L1
IPI00299063.2	STIM1	X	X		cytoskeleton,membrane,endoplasmic reticulum,cytoplasm	cell organization and biogenesis,transport,metabolic process,regulation of biological process,response to stimulus,coagulation	protein binding,metal ion binding	Stromal interaction molecule 1
IPI00830044.1	IGKV6D-41			X				13 kDa protein
IPI00103869.1	CTTNBP2		X		cytoskeleton,membrane,cytoplasm	development	protein binding,motor activity,catalytic activity	Cortactin-binding protein 2
IPI00012759.1	CPLX2	X	X		membrane,cytoplasm,cytosol	development,transport,regulation of biological process,response to stimulus,cell communication,cell differentiation	protein binding	Complexin-2
IPI00830122.2	IGKV1-17			X				A30
IPI00646281.1	L1CAM			X			protein binding	neural cell adhesion molecule L1 isoform 3 precursor
IPI00005837.1	ANGPTL1			X	extracellular	regulation of biological process,response to stimulus,cell communication	protein binding	Angiopoietin-related protein 1

IPI	GENE	Alzheimer's Hippocampus	Control hippocampus	CSF	Cellular localization	Biological process	Molecular function	Protein Description
IPI00002307.1	NLGN3	X	X	X	cell surface,membrane,cytoplasm	development,cell organization and biogenesis,transport,metabolic process,regulation of biological process,response to stimulus,cell communication,cellular homeostasis,cell differentiation,cell growth	protein binding,receptor activity,catalytic activity	Isoform 1 of Neuroligin-3
IPI00183002.6	PPP1R12A	X	X	X	cytoskeleton,cytoplasm,chromosome	cell organization and biogenesis,metabolic process,transport,regulation of biological process,response to stimulus,cell communication	signal transducer activity,protein binding,catalytic activity,enzyme regulator activity	Isoform 1 of Protein phosphatase 1 regulatory subunit 12A
IPI00307328.1	NLGN1			X	cytoskeleton,cell surface,membrane	development,cell organization and biogenesis,metabolic process,transport,regulation of biological process,response to stimulus,cell communication,cellular homeostasis,cell differentiation	protein binding,receptor activity,catalytic activity	Neuroligin-1
IPI00014572.2	SPARC	X		X	extracellular,cytoplasm,nucleus	cell proliferation,development,regulation of biological process,response to stimulus,cell communication	protein binding,metal ion binding	Secreted protein, acidic, cysteine-rich (Osteonectin), isoform CRA_a
IPI00414836.6	OSTF1		X		cytoplasm	regulation of biological process,response to stimulus,cell communication	protein binding	Osteoclast-stimulating factor 1
IPI00643937.3	MTHFD1L			X		metabolic process	nucleotide binding,catalytic activity	Methylenetetrahydrofolate dehydrogenase (NADP+ dependent) 1-like
IPI01022820.1	IGLV6-57			X			protein binding	Amyloid lambda 6 light chain variable region SAR (Fragment)
IPI00297224.6	SUSD5			X	membrane			Sushi domain-containing protein 5
IPI00166546.2	C7orf41	X	X					Isoform 1 of UPF0452 protein C7orf41
IPI00017257.1	CTSO			X	cytoplasm,vacuole	metabolic process	catalytic activity	Cathepsin O
IPI00937304.1	CASK		X			metabolic process	protein binding,nucleotide binding,catalytic activity	Isoform 4 of Peripheral plasma membrane protein CASK
IPI00010800.2	NES	X			cytoskeleton,cytoplasm	cell death,cell proliferation,development,cell organization and biogenesis,regulation of biological process,response to stimulus	protein binding,structural molecule activity	Nestin
IPI00006601.6	CHGB	X	X	X	extracellular,cytoplasm		protein binding	Secretogranin-1
IPI00022745.1	MVD	X	X		cytoplasm,cytosol	cell proliferation,metabolic process,regulation of biological process,response to stimulus	protein binding,nucleotide binding,catalytic activity	Diphosphomevalonate decarboxylase
IPI00375684.6	TRAPPC11		X			metabolic process	catalytic activity	Isoform 3 of UPF0636 protein C4orf41
IPI00007074.5	YARS	X	X		extracellular,cytoplasm,cytosol,nucleus	cell death,metabolic process,regulation of biological process,response to stimulus,cell communication	signal transducer activity,protein binding,RNA binding,nucleotide binding,catalytic activity	Tyrosyl-tRNA synthetase, cytoplasmic
IPI00418446.4	ASAH1	X	X	X			catalytic activity	Isoform 2 of Acid ceramidase
IPI00514594.1	FAM5B			X	extracellular,cytoplasm	development,regulation of biological process	protein binding	Isoform 1 of Protein FAM5B
IPI00333908.2	CCDC91		X		membrane,cytoplasm,Golgi,organelle lumen,nucleus	transport		Isoform 1 of Coiled-coil domain-containing protein 91
IPI00848226.1	GNB2L1	X	X		cytoskeleton,membrane,cytoplasm,ribosome,nucleus	cell death,development,cell organization and biogenesis,cell division,metabolic process,transport,regulation of biological process,response to stimulus,cellular component movement,cell communication,cell growth	protein binding,enzyme regulator activity	Guanine nucleotide-binding protein subunit beta-2-like 1
IPI00550731.2	IGKC	X	X	X			protein binding	Putative uncharacterized protein
IPI00554649.2	ATG4B	X	X		cytoplasm	cell organization and biogenesis,transport,metabolic process,response to stimulus,cell communication	protein binding,catalytic activity	Isoform 1 of Cysteine protease ATG4B
IPI00032959.3	GPD1L	X	X		cytoplasm,cytosol	metabolic process	protein binding,nucleotide binding,catalytic activity	Glycerol-3-phosphate dehydrogenase 1-like protein
IPI00402008.4	PPP6R1		X			metabolic process,response to stimulus,reproduction	antioxidant activity,metal ion binding,catalytic activity	103 kDa protein
IPI00008986.1	SLC7A5	X	X		membrane,cytoplasm,cytosol	development,transport,metabolic process,response to stimulus,cellular component movement,cell differentiation,coagulation	transporter activity	Large neutral amino acids transporter small subunit 1
IPI00004657.1	HLA-B	X	X		membrane,endoplasmic reticulum,cytoplasm,Golgi,endosome	development,regulation of biological process,response to stimulus,cell communication,defense response,cell differentiation	signal transducer activity,protein binding,receptor activity	HLA class I histocompatibility antigen, B-7 alpha chain
IPI00607861.2	H6PD			X	membrane,endoplasmic reticulum,cytoplasm,organelle lumen	metabolic process	nucleotide binding,catalytic activity	GDH/6PGL endoplasmic bifunctional protein
IPI00090764.2	TLR1			X	membrane,cytoplasm	metabolic process,regulation of biological process,response to stimulus,defense response,cell communication	protein binding,signal transducer activity,receptor activity	Toll-like receptor 1

IPI	GENE	Alzheimer's Hippocampus	Control hippocampus	CSF	Cellular localization	Biological process	Molecular function	Protein Description
IPI00005347.3	ZRANB1			X	cytoplasm,nucleus	cell organization and biogenesis,metabolic process,regulation of biological process,response to stimulus,cellular component movement,cell communication	protein binding,metal ion binding,catalytic activity	Ubiquitin thioesterase ZRANB1
IPI00428967.2	TMED7-TICAM2			X	membrane	transport,regulation of biological process,response to stimulus,cell communication	protein binding	TRAM adaptor with GOLD domain isoform 1
IPI00387004.3	MCM7			X		metabolic process	DNA binding,catalytic activity	PNAS-146
IPI00022434.4	ALB			X	extracellular	transport		Uncharacterized protein
IPI00554799.1	SPRN			X	membrane			Shadow of prion protein
IPI00012007.6	AHCY	X	X	X	cytoplasm,cytosol	metabolic process,response to stimulus	catalytic activity	Adenosylhomocysteinase
IPI00220063.5	NDUFS5	X	X		membrane,mitochondrion,cytoplasm	cell organization and biogenesis,transport,metabolic process	catalytic activity	NADH dehydrogenase [ubiquinone] iron-sulfur protein 5
IPI00017659.3	RCSD1			X	cytoskeleton	response to stimulus	protein binding	Isoform 1 of CapZ-interacting protein
IPI00302133.2	TRPV5			X	membrane	cell organization and biogenesis,transport	protein binding,transporter activity	Transient receptor potential cation channel subfamily V member 5
IPI00470528.5	RPL15	X	X		cytoplasm,ribosome,cytosol	cell organization and biogenesis,metabolic process,transport,reproduction	RNA binding,structural molecule activity	60S ribosomal protein L15
IPI00641533.5	PRUNE2	X	X		membrane	regulation of biological process		Prune homolog 2
IPI00215980.1	PVRL2			X				Isoform Alpha of Poliovirus receptor-related protein 2
IPI00218319.3	TPM3			X		response to stimulus,cell communication	structural molecule activity	Isoform 2 of Tropomyosin alpha-3 chain
IPI00000837.4	GRM3		X		cytoskeleton,membrane	metabolic process,regulation of biological process,response to stimulus,cell communication	signal transducer activity,receptor activity	Metabotropic glutamate receptor 3
IPI00219352.4	CBS	X	X		cytoplasm,organelle lumen,cytosol,nucleus	development,metabolic process,regulation of biological process,response to stimulus,cell communication,reproduction	protein binding,metal ion binding,catalytic activity	Isoform 1 of Cystathionine beta-synthase
IPI00028883.1	NDUFB8	X	X		mitochondrion,membrane,endoplasmic reticulum,cytoplasm	metabolic process,transport	catalytic activity	NADH dehydrogenase [ubiquinone] 1 beta subcomplex subunit 8, mitochondrial
IPI00396527.4	PPP1R21	X	X				protein binding	Isoform 1 of KLRAQ motif-containing protein 1
IPI00000184.3	SCOC	X	X		cytoplasm,Golgi,nucleus,endosome	metabolic process,regulation of biological process,response to stimulus,cell communication	protein binding	Isoform 1 of Short coiled-coil protein
IPI00219067.5	GSTM2	X	X	X		metabolic process	protein binding,catalytic activity	Uncharacterized protein
IPI00844287.2	NES	X						Similar to Nestin
IPI00019755.3	GSTO1	X	X	X	cytoplasm,cytosol	metabolic process,regulation of biological process,response to stimulus,cellular homeostasis	antioxidant activity,protein binding,catalytic activity	Glutathione S-transferase omega-1
IPI00220175.5	MAPT	X	X				protein binding	Isoform Tau-E of Microtubule-associated protein tau
IPI00007247.4	PCCB	X	X		mitochondrion,cytoplasm,organelle lumen	metabolic process	nucleotide binding,catalytic activity	Propionyl-CoA carboxylase beta chain, mitochondrial
IPI00292550.2	GALNT13			X	membrane,cytoplasm,Golgi	metabolic process	catalytic activity	Isoform 1 of Polypeptide N-acetylgalactosaminyltransferase 13
IPI00025974.3	CHMP4B		X		membrane,cytoplasm,cytosol,endosome	cell organization and biogenesis,transport	protein binding	Charged multivesicular body protein 4b
IPI00305380.3	IGFBP4			X	extracellular	cell proliferation,development,cell organization and biogenesis,metabolic process,regulation of biological process,response to stimulus,cell communication,defense response,cell growth	protein binding	Insulin-like growth factor-binding protein 4
IPI00001793.3	RFNG			X	extracellular,membrane,cytoplasm,Golgi	development,regulation of biological process,response to stimulus,cell communication,cell differentiation	metal ion binding,catalytic activity	Beta-1,3-N-acetylglucosaminyltransferase radical fringe
IPI00028413.8	ITIH3			X	extracellular	metabolic process,regulation of biological process	protein binding,enzyme regulator activity	Isoform 1 of Inter-alpha-trypsin inhibitor heavy chain H3
IPI00004946.8	CXCL16			X	extracellular,membrane	cell organization and biogenesis,transport,response to stimulus,cellular component movement,defense response	signal transducer activity,protein binding,receptor activity	C-X-C motif chemokine 16
IPI00017597.3	MAPRE3	X	X		cytoskeleton,cytoplasm	cell organization and biogenesis,cell division,metabolic process,regulation of biological process	protein binding,enzyme regulator activity	Isoform 1 of Microtubule-associated protein RP/EB family member 3
IPI00426727.1	MBD4			X	cytoplasm,organelle lumen,chromosome,nucleus	cell death,metabolic process,regulation of biological process,response to stimulus,cell communication	protein binding,DNA binding,catalytic activity	Isoform 1 of Methyl-CpG-binding domain protein 4

IPI	GENE	Alzheimer's Hippocampus	Control hippocampus	CSF	Cellular localization	Biological process	Molecular function	Protein Description
IPI00103874.7	ZFYVE1			X	endoplasmic reticulum,cytoplasm,Golgi	metabolic process,regulation of biological process	protein binding,metal ion binding,nucleotide binding,catalytic activity	Isoform 1 of Zinc finger FYVE domain-containing protein 1
IPI00219718.3	RBP1	X	X					retinol-binding protein 1 isoform a
IPI00384643.2	TP53I3	X				cell death,metabolic process,regulation of biological process	protein binding,metal ion binding,nucleotide binding,catalytic activity	Isoform 1 of Quinone oxidoreductase PIG3
IPI00045051.3	PURB		X		organelle lumen,chromosome,nucleus	development,metabolic process,regulation of biological process,cell differentiation	protein binding,DNA binding,RNA binding,translation regulator activity	Transcriptional activator protein Pur-beta
IPI00744692.1	TALDO1	X	X	X	membrane,cytoplasm,cytosol	metabolic process	catalytic activity	Transaldolase
IPI00165438.3	NRP1			X				Uncharacterized protein
IPI00289499.3	ATIC	X	X		mitochondrion,cytoplasm,cytosol	development,metabolic process	protein binding,catalytic activity	Bifunctional purine biosynthesis protein PURH
IPI01009998.1	IGKV3-20			X			protein binding	anti-(ED-B) scFV
IPI00012835.1	CTBP1	X	X		cytoplasm,organelle lumen,nucleus	cell proliferation,cell organization and biogenesis,metabolic process,regulation of biological process,reproduction,cell differentiation	protein binding,nucleotide binding,catalytic activity	C-terminal-binding protein 1
IPI00003111.7	IGKV1D-33			X				Ig kappa chain V-I region AU
IPI00448095.3	DCXR	X	X		membrane	cell organization and biogenesis,metabolic process	nucleotide binding,catalytic activity	L-xylulose reductase
IPI00024670.5	REEP5	X	X		membrane		protein binding	Receptor expression-enhancing protein 5
IPI00102275.3	GDPD2	X	X		cytoskeleton,membrane,cytoplasm	metabolic process	metal ion binding,catalytic activity	Glycerophosphoinositol inositolphosphodiesterase GDPD2
IPI00644025.1	SV2A	X	X	X	membrane,endoplasmic reticulum,cytoplasm	transport,cellular homeostasis	transporter activity,receptor activity	Isoform 1 of Synaptic vesicle glycoprotein 2A
IPI00002745.1	CTSZ			X	extracellular,endoplasmic reticulum,cytoplasm,vacuole	development,metabolic process	catalytic activity	Cathepsin Z
IPI00006746.3	ERMN	X	X	X	cytoskeleton,membrane,cytoplasm	cell organization and biogenesis,regulation of biological process	protein binding	Isoform 1 of Ermin
IPI00031982.1	NCKAP1	X	X		membrane	cell death,development	protein binding	Isoform 1 of Nck-associated protein 1
IPI00386975.3	DSC1			X	membrane		metal ion binding	Isoform 1B of Desmocollin-1
IPI00024067.4	CLTC	X	X	X	cytoskeleton,mitochondrion,membrane,cytoplasm,Golgi,cytosol	development,cell organization and biogenesis,transport,metabolic process,regulation of biological process,response to stimulus,cell communication,cell differentiation	protein binding,structural molecule activity	Isoform 1 of Clathrin heavy chain 1
IPI00219029.3	GOT1	X	X	X	cytoplasm,vacuole,cytosol	metabolic process,response to stimulus	catalytic activity	Aspartate aminotransferase, cytoplasmic
IPI00456736.5	RGMB			X	membrane			RGM domain family member B
IPI00412878.1	NUDT14	X	X		cytoplasm		protein binding,metal ion binding,catalytic activity	Uridine diphosphate glucose pyrophosphatase
IPI00003565.1	PSMD10	X	X		cytoskeleton,cytoplasm,proteasome,organelle lumen,nucleus,cytosol	cell death,cell organization and biogenesis,transport,metabolic process,regulation of biological process,response to stimulus,cell communication,cell growth	protein binding	26S proteasome non-ATPase regulatory subunit 10
IPI00007702.1	HSPA2	X	X	X	cell surface,membrane	cell organization and biogenesis,response to stimulus,reproduction,cell differentiation	protein binding,nucleotide binding	Heat shock-related 70 kDa protein 2
IPI00026627.4	RP2		X		membrane,cytoplasm	cell organization and biogenesis,transport,metabolic process,regulation of biological process	protein binding,nucleotide binding,catalytic activity,enzyme regulator activity	Protein XRP2
IPI00419724.2	SEMA4B			X	membrane		protein binding	semaphorin-4B precursor
IPI00646161.2	RENBP	X	X			metabolic process	catalytic activity	Uncharacterized protein
IPI00014223.2	LRRC4C			X	membrane	cell organization and biogenesis,development,regulation of biological process,cell differentiation	protein binding	Leucine-rich repeat-containing protein 4C
IPI00014439.4	QDPR	X	X	X	membrane,mitochondrion,cytoplasm,cytosol	development,metabolic process,response to stimulus	protein binding,nucleotide binding,catalytic activity	Dihydropteridine reductase
IPI00306382.2	SCAMP3		X		membrane,cytoplasm,Golgi	transport,response to stimulus		Isoform 1 of Secretory carrier-associated membrane protein 3
IPI00005126.1	EFNB2		X	X	membrane	development,regulation of biological process,response to stimulus,cellular component movement,cell communication,cell differentiation	protein binding	Ephrin-B2

IPI	GENE	Alzheimer's Hippocampus	Control hippocampus	CSF	Cellular localization	Biological process	Molecular function	Protein Description
IPI00013912.1	C1orf198	X	X		cytoplasm			Uncharacterized protein C1orf198
IPI00852806.1	EXOC6B		X		cytoplasm	transport		Isoform 1 of Exocyst complex component 6B
IPI00430411.1	CAMK4		X		cytoplasm,organelle lumen,cytosol,nucleus	development,metabolic process,regulation of biological process,response to stimulus,defense response,cell communication,cell differentiation	protein binding,nucleotide binding,catalytic activity	Calcium/calmodulin-dependent protein kinase type IV
IPI00154567.6	SPATA20		X					Isoform 2 of Spermatogenesis-associated protein 20
IPI00845263.3	FN1			X	extracellular		protein binding	fibronectin isoform 4 preproprotein
IPI00816626.1	PLXNB2			X			protein binding	PLXNB2 protein
IPI00744825.1	QTRTD1			X			catalytic activity	Similar to SubName: Full=SJCHGC03017 protein;
IPI00003479.3	MAPK1	X	X		cytoskeleton,membrane,mitochondrion,cytoplasm,Golgi,organelle lumen,nucleus,cytosol,endosome	cell death,cell organization and biogenesis,development,metabolic process,regulation of biological process,response to stimulus,defense response,cell proliferation,transport,cellular component movement,cell communication,cell differentiation,coagulation	protein binding,signal transducer activity,DNA binding,nucleotide binding,catalytic activity	Mitogen-activated protein kinase 1
IPI00019862.4	BTN2A1			X				butyrophilin subfamily 2 member A1 isoform 2 precursor
IPI00001662.2	OPCML	X	X	X			protein binding	Uncharacterized protein
IPI00297208.7	FAM184B			X	cytoskeleton,mitochondrion,membrane,cytoplasm	transport	motor activity,catalytic activity	Protein FAM184B
IPI00515119.3	HSP90AB1		X			metabolic process,response to stimulus	protein binding,nucleotide binding	Uncharacterized protein
IPI00007756.1	RAB22A		X		membrane,cytoplasm,nucleus,endosome	cell organization and biogenesis,transport,metabolic process,regulation of biological process,response to stimulus,cell communication	protein binding,nucleotide binding,catalytic activity	Ras-related protein Rab-22A
IPI00375205.1	GALNT10			X	membrane,cytoplasm,Golgi	metabolic process	metal ion binding,catalytic activity	Isoform 1 of Polypeptide N-acetylgalactosaminyltransferase 10
IPI00027248.4	TUSC2			X		cell proliferation,cell communication		Tumor suppressor candidate 2
IPI00220558.3	RYR1		X		membrane	transport,cellular homeostasis	protein binding,transporter activity	Isoform 2 of Ryanodine receptor 1
IPI00737638.2	FAM169A	X	X					Isoform 1 of Protein FAM169A
IPI00550363.3	TAGLN2	X	X	X	membrane,nucleus	development	protein binding	Transgelin-2
IPI00299594.2	NRP1			X	extracellular,cell surface,cytoskeleton,membrane,cytoplasm,cytosol	cell proliferation,development,cell organization and biogenesis,transport,metabolic process,regulation of biological process,response to stimulus,cellular component movement,cell communication,cell growth,cell differentiation	protein binding,signal transducer activity,metal ion binding,receptor activity,catalytic activity	Isoform 1 of Neuropilin-1
IPI00023814.2	NEO1	X	X	X	membrane	development,cell organization and biogenesis,regulation of biological process,response to stimulus,cell differentiation	protein binding	Isoform 1 of Neogenin
IPI00061280.7	MTMR12	X	X		cytoplasm	metabolic process	catalytic activity	Isoform 1 of Myotubularin-related protein 12
IPI00009203.4	SNX7			X		cell communication	protein binding	Isoform 3 of Sorting nexin-7
IPI00411426.3	VPS26A	X	X		membrane,cytoplasm,cytosol,endosome	transport	protein binding,transporter activity	Vacuolar protein sorting-associated protein 26A
IPI00011454.1	GANAB			X		metabolic process	catalytic activity	Isoform 2 of Neutral alpha-glucosidase AB
IPI00018398.4	PSMC3	X	X		proteasome,cytoplasm,organelle lumen,cytosol,nucleus	cell death,development,metabolic process,regulation of biological process,response to stimulus,cell communication	protein binding,nucleotide binding,catalytic activity	26S protease regulatory subunit 6A
IPI00376131.5	LINGO3			X				Leucine rich repeat and Ig domain containing 3
IPI00167065.3	ADSSL1		X		cytoplasm	metabolic process	metal ion binding,nucleotide binding,catalytic activity	Isoform 2 of Adenylosuccinate synthetase isozyme 1
IPI00019148.1	IGBP1	X	X		cytoplasm	cell death,metabolic process,regulation of biological process,response to stimulus,cellular component movement,cell communication	protein binding,enzyme regulator activity	Immunoglobulin-binding protein 1
IPI00787936.2	CTSL1P3			X			catalytic activity	similar to Cathepsin L1

IPI	GENE	Alzheimer's Hippocampus	Control hippocampus	CSF	Cellular localization	Biological process	Molecular function	Protein Description
IPI00011217.3	NDUFS4	X	X		mitochondrion,membrane,cytoplasm	cell proliferation,cell organization and biogenesis,development,transport,metabolic process,regulation of biological process,response to stimulus,cell communication	catalytic activity	NADH dehydrogenase [ubiquinone] iron-sulfur protein 4, mitochondrial
IPI00896380.1	IGHM			X				Isoform 2 of Ig mu chain C region
IPI00643525.1	C4A			X	extracellular	metabolic process,regulation of biological process,response to stimulus,defense response	protein binding,enzyme regulator activity	Uncharacterized protein
IPI00029556.1	C1orf105			X				Uncharacterized protein C1orf105
IPI00219616.7	PRPS1	X	X		cytoplasm,cytosol	development,metabolic process	protein binding,metal ion binding,nucleotide binding,catalytic activity	Ribose-phosphate pyrophosphokinase 1
IPI00220740.1	NPM1	X	X					Isoform 2 of Nucleophosmin
IPI00171412.2	SUMF2	X		X				sulfatase-modifying factor 2 isoform b precursor
IPI00788835.1	PENK			X		regulation of biological process,response to stimulus,cell communication		25 kDa protein
IPI00007461.4	DPY19L1		X		membrane			Isoform 1 of Protein dpy-19 homolog 1
IPI00220299.1	FLAD1	X	X		cytoplasm,cytosol	metabolic process	nucleotide binding,catalytic activity	Isoform 1 of FAD synthase
IPI00847652.3	LOC400891			X			protein binding	Isoform 1 of Leucine-rich repeat-containing protein LOC400891
IPI00414467.7	COLEC12			X	extracellular,membrane	cell organization and biogenesis,transport,regulation of biological process,response to stimulus,cell communication,defense response	metal ion binding,receptor activity	Isoform 1 of Collectin-12
IPI00026872.2	ADORA1	X			membrane	regulation of biological process,response to stimulus,cell communication	signal transducer activity,receptor activity	cDNA FLJ58571, highly similar to Adenosine A1 receptor
IPI00000690.1	AIFM1	X	X		membrane,mitochondrion,cytoplasm,cytosol,nucleus	cell death,cell organization and biogenesis,development,metabolic process,regulation of biological process,response to stimulus,cell communication,cellular homeostasis,cell differentiation	protein binding,DNA binding,catalytic activity	Isoform 1 of Apoptosis-inducing factor 1, mitochondrial
IPI00019901.1	ADD1	X	X	X	cytoskeleton,membrane,cytoplasm,cytosol,nucleus	cell death,cell organization and biogenesis,metabolic process,regulation of biological process,response to stimulus,cell communication	protein binding,metal ion binding	Isoform 1 of Alpha-adducin
IPI00300299.6	SPCS3	X	X		membrane,endoplasmic reticulum,cytoplasm	metabolic process,transport,regulation of biological process,cell communication	catalytic activity	Signal peptidase complex subunit 3
IPI00019146.6	CXADR			X	extracellular,membrane,cytoplasm,nucleus	cell organization and biogenesis,development,transport,regulation of biological process,response to stimulus,cellular component movement,defense response,reproduction,cell differentiation,coagulation	protein binding,receptor activity	Isoform 1 of Coxsackievirus and adenovirus receptor
IPI00171626.3	LPCAT1		X		membrane,endoplasmic reticulum,cytoplasm,Golgi	development,metabolic process,regulation of biological process	metal ion binding,catalytic activity	Lysophosphatidylcholine acyltransferase 1
IPI00607642.1	MBP	X	X				structural molecule activity	Isoform 4 of Myelin basic protein
IPI01018354.1	LINC00470			X				Uncharacterized protein
IPI00029111.3	DPYSL3	X	X		cytoplasm	metabolic process	catalytic activity	dihydropyrimidinase-related protein 3 isoform 1
IPI00015856.6	DNPEP	X				metabolic process	metal ion binding,catalytic activity	aspartyl aminopeptidase
IPI00412216.2	VPS13C	X	X	X				Isoform 2 of Vacuolar protein sorting-associated protein 13C
IPI00440577.3	IGKV2-24			X			protein binding	IGKV2-24 protein
IPI00218874.1	SPP1			X				Isoform B of Osteopontin
IPI00005222.6	EPHB6			X	membrane	metabolic process	protein binding,nucleotide binding,catalytic activity	Isoform 2 of Ephrin type-B receptor 6
IPI00022391.1	APCS			X	extracellular	cell organization and biogenesis,metabolic process,response to stimulus,defense response	protein binding,metal ion binding	Serum amyloid P-component
IPI00216592.2	HNRNPC	X	X	X			nucleotide binding	Isoform C1 of Heterogeneous nuclear ribonucleoproteins C1/C2
IPI00020495.1	MRPS36		X		mitochondrion,cytoplasm,ribosome,organelle lumen	metabolic process	structural molecule activity	28S ribosomal protein S36, mitochondrial
IPI00396423.3	CLSTN3			X	membrane		metal ion binding	Uncharacterized protein

IPI	GENE	Alzheimer's Hippocampus	Control hippocampus	CSF	Cellular localization	Biological process	Molecular function	Protein Description
IPI00015894.1	CDC42EP4	X	X		cytoskeleton,membrane,cytoplasm	cell organization and biogenesis,regulation of biological process,response to stimulus,cell communication	protein binding	Cdc42 effector protein 4
IPI00025473.1	B4GALNT1			X	membrane,cytoplasm,Golgi	metabolic process,reproduction	catalytic activity	Beta-1,4 N-acetylgalactosaminyltransferase 1
IPI00791479.2	ROBO2			X			protein binding	Uncharacterized protein
IPI00064086.5	EEF1D	X	X		cytoplasm	metabolic process	RNA binding	elongation factor 1-delta isoform 4
IPI00170635.1	SECTM1			X	extracellular,membrane,cytoplasm,Golgi	regulation of biological process,response to stimulus,cell communication	protein binding,signal transducer activity	Secreted and transmembrane protein 1
IPI00845508.3	BAHCC1			X			DNA binding	BAH and coiled-coil domain-containing protein 1
IPI00002957.2	TBC1D17		X			metabolic process,regulation of biological process	enzyme regulator activity	TBC1 domain family member 17
IPI00027174.1	FGFR3			X	membrane,endoplasmic reticulum,cytoplasm	cell death,cell proliferation,development,metabolic process,regulation of biological process,response to stimulus,cell communication,cell differentiation	protein binding,signal transducer activity,nucleotide binding,receptor activity,catalytic activity	Isoform 1 of Fibroblast growth factor receptor 3
IPI00008207.7	MAN1B1			X	membrane,endoplasmic reticulum,cytoplasm,nucleus	metabolic process,regulation of biological process,response to stimulus,cell communication	signal transducer activity,DNA binding,metal ion binding,receptor activity,catalytic activity	Endoplasmic reticulum mannosyl-oligosaccharide 1,2-alpha-mannosidase
IPI00383581.4	GANAB	X	X		membrane	transport,metabolic process,regulation of biological process,response to stimulus,cell communication	transporter activity,catalytic activity	cDNA FLJ61290, highly similar to Neutral alpha-glucosidase AB
IPI00470913.8	RGPD1			X	membrane	transport	protein binding	RANBP2-like and GRIP domain-containing protein 1/2
IPI00029658.1	EFEMP1	X		X	extracellular,cell surface,membrane	development,metabolic process,regulation of biological process,response to stimulus,cell communication,cell differentiation	protein binding,signal transducer activity,metal ion binding,receptor activity,catalytic activity	Isoform 1 of EGF-containing fibulin-like extracellular matrix protein 1
IPI00830018.4	IGKC			X				13 kDa protein
IPI00008552.6	GLRX3		X		spliceosomal complex,cytoplasm,nucleus	cell organization and biogenesis,metabolic process,regulation of biological process,cellular homeostasis	protein binding,metal ion binding,catalytic activity	Glutaredoxin-3
IPI00003815.3	ARHGDIA	X	X	X	cytoskeleton,membrane,cytoplasm,cytosol	cell death,cell organization and biogenesis,development,metabolic process,regulation of biological process,response to stimulus,cellular component movement,cell communication,cell differentiation	protein binding,enzyme regulator activity	Rho GDP-dissociation inhibitor 1
IPI00015911.2	DLD	X	X	X	mitochondrion,cytoplasm,vacuole,organelle lumen	development,metabolic process,regulation of biological process,cellular homeostasis,reproduction,cell differentiation	nucleotide binding,catalytic activity	Dihydrolipoyl dehydrogenase, mitochondrial
IPI00385042.4	GTPBP4			X	membrane,cytoplasm,Golgi,organelle lumen,nucleus	cell proliferation,transport,metabolic process,regulation of biological process,response to stimulus,cellular component movement,cell communication	protein binding,transporter activity,nucleotide binding,catalytic activity	Nucleolar GTP-binding protein 1
IPI00215914.5	ARF1		X	X	membrane,cytoplasm,Golgi,cytosol,nucleus	cell organization and biogenesis,transport,metabolic process,regulation of biological process,response to stimulus,defense response,cell communication,cellular homeostasis	protein binding,signal transducer activity,metal ion binding,nucleotide binding,catalytic activity,enzyme regulator activity	ADP-ribosylation factor 1
IPI00017696.1	C1S			X	extracellular	development,metabolic process,regulation of biological process,response to stimulus,defense response,cell differentiation	metal ion binding,catalytic activity	Complement C1s subcomponent
IPI00301865.5	TMEM132A		X	X	membrane,endoplasmic reticulum,cytoplasm,Golgi			Isoform 1 of Transmembrane protein 132A
IPI00005668.4	AKR1C2			X	cytoplasm	cell proliferation,metabolic process,regulation of biological process,response to stimulus,cell communication	signal transducer activity,receptor activity,catalytic activity	Aldo-keto reductase family 1 member C2
IPI00218971.4	PDP1		X		mitochondrion,cytoplasm,organelle lumen	metabolic process,regulation of biological process	protein binding,metal ion binding,catalytic activity	[Pyruvate dehydrogenase [acetyl-transferring]]-phosphatase 1, mitochondrial
IPI00030351.2	DLG1	X	X		cytoskeleton,membrane,endoplasmic reticulum,cytoplasm,nucleus,cytosol	cell proliferation,development,cell organization and biogenesis,regulation of biological process,response to stimulus,cell communication,cell differentiation	protein binding,catalytic activity	Isoform 1 of Disks large homolog 1
IPI00552939.1	C1QL3			X	extracellular	cell organization and biogenesis,regulation of biological process,cell communication	protein binding	Isoform 1 of Complement C1q-like protein 3
IPI00000138.2	MGAT1			X	membrane,cytoplasm,Golgi	development,metabolic process	metal ion binding,catalytic activity	Alpha-1,3-mannosyl-glycoprotein 2-beta-N-acetylglucosaminyltransferase
IPI00010949.3	SIAE			X	extracellular,cytoplasm,vacuole		catalytic activity	Isoform 1 of Sialate O-acetylesterase
IPI00829845.1	IGHV3-35			X				Immunglobulin heavy chain variable region

IPI	GENE	Alzheimer's Hippocampus	Control hippocampus	CSF	Cellular localization	Biological process	Molecular function	Protein Description
IPI00329719.1	MYO1D		X		cytoskeleton,membrane,endoplasmic reticulum,cytoplasm	metabolic process,regulation of biological process	protein binding,motor activity,nucleotide binding,catalytic activity	Myosin-Id
IPI00744226.1	FAM9B			X				Conserved hypothetical protein
IPI00020884.2	PLXNA3			X	membrane	cell organization and biogenesis,development,regulation of biological process,response to stimulus,cell communication,cell differentiation	protein binding,signal transducer activity,receptor activity	Plexin-A3
IPI00006980.1	C14orf166	X	X		cytoskeleton,cytoplasm,organelle lumen,nucleus	metabolic process,regulation of biological process	protein binding	UPF0568 protein C14orf166
IPI00743931.3	GORASP2	X	X		cytoplasm,Golgi		protein binding	cDNA FLJ59712, highly similar to Golgi reassembly-stacking protein 2
IPI00009867.3	KRT5			X	cytoskeleton,mitochondrion,membrane,cytoplasm,cytosol	cell organization and biogenesis,development	protein binding,motor activity,structural molecule activity,catalytic activity	Keratin, type II cytoskeletal 5
IPI00216057.6	SORD	X	X		extracellular,mitochondrion,membrane,cytoplasm	metabolic process,cellular component movement	metal ion binding,nucleotide binding,catalytic activity	Sorbitol dehydrogenase
IPI00186826.1	EPHB4			X	membrane	metabolic process,regulation of biological process,response to stimulus,cell communication	protein binding,signal transducer activity,nucleotide binding,receptor activity,catalytic activity	Uncharacterized protein
IPI00220642.7	YWHAG	X	X	X	cytoplasm,cytosol	development,transport,metabolic process,regulation of biological process,response to stimulus,cell communication,cell differentiation	protein binding,enzyme regulator activity	14-3-3 protein gamma
IPI00020996.5	IGFALS			X	extracellular,organelle lumen,nucleus	regulation of biological process,response to stimulus,cell communication	protein binding	Insulin-like growth factor-binding protein complex acid labile subunit
IPI00396077.4	TOPORS			X	cytoskeleton,cytoplasm,organelle lumen,nucleus	cell death,cell proliferation,cell organization and biogenesis,development,metabolic process,regulation of biological process,response to stimulus,cell communication,cell differentiation	protein binding,DNA binding,metal ion binding,catalytic activity	Isoform 1 of E3 ubiquitin-protein ligase Topors
IPI00423463.1	IGHG1			X	membrane		protein binding	Putative uncharacterized protein DKFZp686O01196
IPI00441498.1	FOLR1			X	extracellular,membrane	cell death,metabolic process,transport	receptor activity	Folate receptor alpha
IPI00301961.4	PCSK1			X	extracellular,endoplasmic reticulum,cytoplasm,Golgi	development,metabolic process,transport,regulation of biological process,response to stimulus,cell communication,cell differentiation	protein binding,catalytic activity	Neuroendocrine convertase 1
IPI00182933.5	CYB5A	X	X				metal ion binding	Isoform 2 of Cytochrome b5
IPI00784119.1	ATP6AP1			X	membrane,cytoplasm,vacuole	cell death,transport	transporter activity,nucleotide binding,catalytic activity	V-type proton ATPase subunit S1
IPI00478640.2	SCIMP			X	membrane	regulation of biological process,response to stimulus,cell communication	protein binding	Isoform 1 of Transmembrane protein C17orf87
IPI00387159.3	ING3			X	organelle lumen,nucleus	cell death,cell organization and biogenesis,metabolic process,regulation of biological process	protein binding,metal ion binding,catalytic activity	Isoform 1 of Inhibitor of growth protein 3
IPI00021828.1	CSTB	X	X	X	cytoplasm,organelle lumen,nucleus	cell death,metabolic process,regulation of biological process,response to stimulus	protein binding,enzyme regulator activity	Cystatin-B
IPI00302688.7	ECHDC1	X	X		cytoplasm,cytosol	metabolic process	catalytic activity	Isoform 1 of Enoyl-CoA hydratase domain-containing protein 1
IPI00647205.1	SOGA3		X		membrane	metabolic process,regulation of biological process		Uncharacterized protein C6orf174
IPI00830051.2	IGHV5-78			X				Similar to Immunolgoobulin heavy chain
IPI00376383.2	CNTRL			X	membrane,cytoplasm,Golgi	transport	transporter activity,nucleotide binding	Isoform 2 of Centriolin
IPI00165975.6	ISLR2			X			protein binding	84 kDa protein
IPI00027776.6	FECH	X	X		mitochondrion,membrane,cytoplasm,organelle lumen	metabolic process,response to stimulus	protein binding,metal ion binding,catalytic activity	Isoform 1 of Ferrochelatase, mitochondrial
IPI00514561.1	HNRNPK	X	X		organelle lumen,nucleus		RNA binding	cDNA FLJ54552, highly similar to Heterogeneous nuclear ribonucleoprotein K
IPI00477868.1	LAMA5			X	extracellular,membrane	development,regulation of biological process,cellular component movement,cell differentiation	protein binding,signal transducer activity	LAMA5 protein
IPI00007921.1	NRXN2			X	extracellular,membrane	cell organization and biogenesis,development	protein binding,metal ion binding	Isoform 1a of Neurexin-2-alpha
IPI00409635.2	ESYT2	X	X				protein binding	Isoform 2 of Extended synaptotagmin-2
IPI00009802.1	VCAN	X	X	X	extracellular	cell organization and biogenesis,development,response to stimulus,cellular component movement,cell differentiation	protein binding,metal ion binding	Isoform V0 of Versican core protein

IPI	GENE	Alzheimer's Hippocampus	Control hippocampus	CSF	Cellular localization	Biological process	Molecular function	Protein Description
IPI00294215.3	KIAA0232			X			nucleotide binding	Uncharacterized protein KIAA0232
IPI00007188.6	SLC25A5	X	X		cytoskeleton,mitochondrion,membrane,cytoplasm,organelle lumen	metabolic process,transport,regulation of biological process,cell communication	protein binding,transporter activity	ADP/ATP translocase 2
IPI00020990.1	OMD			X	extracellular		protein binding	Osteomodulin
IPI00019355.1	TSC22D1		X			metabolic process,regulation of biological process		Isoform 2 of TSC22 domain family protein 1
IPI00009480.1	COPS8	X	X		cytoplasm,organelle lumen,nucleus	metabolic process		COP9 signalosome complex subunit 8
IPI00003799.2	HEBP2			X				Isoform 2 of Heme-binding protein 2
IPI00005585.5	TAX1BP3	X	X		membrane,cytoplasm,nucleus	cell proliferation,metabolic process,regulation of biological process,response to stimulus,cell communication	protein binding	Tax1-binding protein 3
IPI00024012.4	FZD7			X	membrane,cytoplasm	cell proliferation,development,cell organization and biogenesis,transport,cell division,metabolic process,regulation of biological process,response to stimulus,cell communication,cell differentiation,reproduction	protein binding,signal transducer activity,receptor activity	Frizzled-7
IPI00178440.3	EEF1B2	X	X		cytoplasm,cytosol	metabolic process	protein binding,RNA binding	Elongation factor 1-beta
IPI00027847.3	LPL			X	extracellular,cell surface,membrane	cell organization and biogenesis,metabolic process,regulation of biological process,response to stimulus,cell differentiation	protein binding,catalytic activity	Lipoprotein lipase
IPI00103510.1	RXFP2			X	membrane	cell death,cell proliferation,development,metabolic process,regulation of biological process,response to stimulus,cell communication,reproduction,cell differentiation	protein binding,signal transducer activity,receptor activity	Relaxin receptor 2
IPI00015988.9	HLA-G			X	membrane,endoplasmic reticulum,cytoplasm,Golgi,endosome	cell proliferation,development,regulation of biological process,response to stimulus,defense response,cell communication,cell differentiation	signal transducer activity,protein binding,receptor activity	HLA class I histocompatibility antigen, alpha chain G
IPI00184180.4	PCYOX1L		X		extracellular	metabolic process	catalytic activity	Isoform 1 of Prenylcysteine oxidase-like
IPI00009368.4	SFXN1	X	X		mitochondrion,membrane,cytoplasm	development,transport,cell differentiation	transporter activity	Sideroflexin-1
IPI00297646.5	COL1A1			X	extracellular,cytoplasm	cell organization and biogenesis,development,metabolic process,transport,regulation of biological process,response to stimulus,cellular component movement,cell communication,cell differentiation,coagulation	protein binding,structural molecule activity	Collagen alpha-1(I) chain
IPI00103530.1	ATL1	X	X		membrane,endoplasmic reticulum,cytoplasm,Golgi	cell death,development,cell organization and biogenesis,metabolic process,cell differentiation	protein binding,nucleotide binding,catalytic activity	Atlastin-1
IPI00220562.3	NPTX1	X	X	X	cytoplasm	development,cell organization and biogenesis,transport,cell communication,cell differentiation	metal ion binding	Neuronal pentraxin-1
IPI00435020.3	NCAM1			X			protein binding	Isoform 2 of Neural cell adhesion molecule 1
IPI00216470.1	PIP4K2B	X	X	X	membrane,endoplasmic reticulum,cytoplasm,nucleus	metabolic process,regulation of biological process,response to stimulus,cell communication	signal transducer activity,nucleotide binding,catalytic activity	Isoform 1 of Phosphatidylinositol-5-phosphate 4-kinase type-2 beta
IPI00152748.1	UAP1L1	X				metabolic process	catalytic activity	UDP-N-acteylglucosamine pyrophosphorylase 1-like 1, isoform CRA_a (Fragment)
IPI00011218.1	CSF1R			X	cell surface,membrane	cell proliferation,cell organization and biogenesis,development,metabolic process,transport,regulation of biological process,response to stimulus,cellular component movement,cell communication,defense response,cell differentiation	protein binding,signal transducer activity,nucleotide binding,receptor activity,catalytic activity	Macrophage colony-stimulating factor 1 receptor
IPI00024266.3	MGST3	X	X		membrane,endoplasmic reticulum,cytoplasm	metabolic process,regulation of biological process,response to stimulus,cell communication	antioxidant activity,catalytic activity	Microsomal glutathione S-transferase 3
IPI00914938.1	AP2A2	X	X		membrane,cytoplasm,cytosol	development,cell organization and biogenesis,transport,regulation of biological process,response to stimulus,cell communication,defense response,cell differentiation	protein binding,transporter activity	Isoform 1 of AP-2 complex subunit alpha-2
IPI00382606.1	F7			X	extracellular	metabolic process	protein binding,metal ion binding,catalytic activity	factor VII active site mutant immunoconjugate
IPI00940393.3	EEF1A1			X		metabolic process	nucleotide binding,catalytic activity	Uncharacterized protein
IPI00299738.1	PCOLCE			X	extracellular	development,metabolic process,regulation of biological process	protein binding,enzyme regulator activity	Procollagen C-endopeptidase enhancer 1

IPI	GENE	Alzheimer's Hippocampus	Control hippocampus	CSF	Cellular localization	Biological process	Molecular function	Protein Description
IPI00014371.1	CDH18			X	membrane	cell organization and biogenesis	metal ion binding	Cadherin-18
IPI00009365.3	COX16			X	membrane,mitochondrion,cytoplasm			Cytochrome c oxidase assembly protein COX16 homolog, mitochondrial
IPI00297277.4	RNF150			X	membrane		protein binding,metal ion binding	Isoform 1 of RING finger protein 150
IPI00168336.1	LEMD2		X		membrane,nucleus	development,regulation of biological process,response to stimulus,cell communication,cell differentiation		LEM domain-containing protein 2
IPI00012837.1	KIF5B	X	X		cytoskeleton,membrane,cytoplasm,organelle lumen,chromosome,nucleus	cell organization and biogenesis,metabolic process,transport,cellular component movement,cell differentiation,reproduction	protein binding,motor activity,nucleotide binding,catalytic activity	Kinesin-1 heavy chain
IPI00005564.1	STC1			X	extracellular,membrane,cytoplasm	development,regulation of biological process,response to stimulus,cell communication,cellular homeostasis,reproduction	protein binding	Stanniocalcin-1
IPI00010133.3	CORO1A	X	X	X	cytoskeleton,membrane,cytoplasm,nucleus	cell proliferation,cell organization and biogenesis,transport,regulation of biological process,response to stimulus,cellular component movement,defense response	protein binding	Coronin-1A
IPI00852621.1	FAM116B		X		membrane			Family with sequence similarity 116, member B
IPI00021997.1	CREG1	X		X	extracellular,organelle lumen,nucleus	cell proliferation,development,metabolic process,regulation of biological process	protein binding,nucleotide binding,catalytic activity	Protein CREG1
IPI00026053.1	CLDN11	X	X		membrane	development,cell communication,cellular homeostasis,reproduction	protein binding,structural molecule activity	Claudin-11
IPI00001676.9	NPLOC4	X	X			metabolic process	protein binding	Isoform 2 of Nuclear protein localization protein 4 homolog
IPI00298971.1	VTN		X	X	extracellular,membrane	cell organization and biogenesis,metabolic process,transport,regulation of biological process,response to stimulus,cellular component movement,cell communication,coagulation	protein binding,receptor activity	Vitronectin
IPI00298817.7	RUNDC3A		X			regulation of biological process,response to stimulus,cell communication	enzyme regulator activity	Isoform 3 of RUN domain-containing protein 3A
IPI00658109.1	CKMT1A	X	X		membrane,mitochondrion,cytoplasm	metabolic process	nucleotide binding,catalytic activity	Isoform 1 of Creatine kinase U-type, mitochondrial
IPI00328082.3	TARSL2	X	X		cytoplasm	metabolic process	nucleotide binding,catalytic activity	Isoform 1 of Probable threonyl-tRNA synthetase 2, cytoplasmic
IPI00021263.3	YWHAZ	X	X	X	cytoskeleton,mitochondrion,cytoplasm,organelle lumen,cytosol,nucleus	cell death,cell organization and biogenesis,metabolic process,transport,regulation of biological process,response to stimulus,defense response,cell communication,coagulation	protein binding,catalytic activity	14-3-3 protein zeta/delta
IPI00943670.1	LOC727947	X	X			metabolic process	transporter activity,catalytic activity	similar to ubiquinol-cytochrome c reductase binding protein
IPI00409608.3	CERS6	X			membrane,endoplasmic reticulum,cytoplasm,nucleus	metabolic process,regulation of biological process	DNA binding,catalytic activity	Ceramide synthase 6
IPI00007321.2	LYPLA1	X		X	mitochondrion,cytoplasm		catalytic activity	cDNA FLJ60607, highly similar to Acyl-protein thioesterase 1
IPI00384542.4	NID1			X			protein binding	Isoform 2 of Nidogen-1
IPI00019329.1	DYNLL1	X	X		cytoskeleton,mitochondrion,membrane,cytoplasm,cytosol,nucleus	cell death,cell organization and biogenesis,transport,metabolic process,regulation of biological process,reproduction	protein binding,motor activity,catalytic activity,enzyme regulator activity	Dynein light chain 1, cytoplasmic
IPI00218292.2	UFD1L	X	X	X	cytoplasm,cytosol,nucleus	development,metabolic process	protein binding,catalytic activity	Isoform Short of Ubiquitin fusion degradation protein 1 homolog
IPI00216219.3	TJP1	X	X		membrane,cytoplasm,Golgi,nucleus,cytosol	cell death,cell organization and biogenesis,development,regulation of biological process,response to stimulus,cell communication	protein binding	Isoform Long of Tight junction protein ZO-1
IPI00000137.1	GNPTG			X	extracellular,cytoplasm,Golgi			N-acetylglucosamine-1-phosphotransferase subunit gamma
IPI00480183.3	PTPRF			X		metabolic process	protein binding,catalytic activity	LAR splice variant 1
IPI00002230.6	NCEH1	X	X			metabolic process	catalytic activity	neutral cholesterol ester hydrolase 1 isoform b
IPI00925923.1	FAM3C			X				Uncharacterized protein
IPI00006648.4	RABGAP1L		X		cytoskeleton	cell organization and biogenesis,metabolic process,regulation of biological process	enzyme regulator activity	Isoform 7 of Rab GTPase-activating protein 1-like
IPI00219330.2	ILF3	X	X				RNA binding	Isoform 5 of Interleukin enhancer-binding factor 3
IPI00011564.1	SDC4	X		X	extracellular,cell surface,membrane,cytoplasm	development,cell organization and biogenesis,metabolic process,regulation of biological process	signal transducer activity,protein binding,receptor activity	Syndecan-4

IPI	GENE	Alzheimer's Hippocampus	Control hippocampus	CSF	Cellular localization	Biological process	Molecular function	Protein Description
IPI00940343.1	TPM1	X	X		cytoskeleton,cytoplasm			cDNA FLJ16459 fis, clone BRCAN2002473, moderately similar to Tropomyosin, fibroblast isoform 2
IPI00219941.1	OSBPL1A	X	X			transport		Isoform A of Oxysterol-binding protein-related protein 1
IPI00025721.3	COPS3	X	X		cytoplasm,nucleus	development,metabolic process,regulation of biological process,response to stimulus,cell communication	protein binding	COP9 signalosome complex subunit 3
IPI00947496.1	CFB			X		metabolic process	protein binding,catalytic activity	Uncharacterized protein
IPI00166749.3	PMPCA		X		mitochondrion,membrane,cytoplasm,organelle lumen	cell organization and biogenesis,transport,metabolic process	metal ion binding,catalytic activity	Mitochondrial-processing peptidase subunit alpha
IPI00027809.2	PPP3CB	X	X				catalytic activity	serine/threonine-protein phosphatase 2B catalytic subunit beta isoform isoform a
IPI00006967.3	PCDH9	X	X	X	membrane		metal ion binding	Isoform 2 of Protocadherin-9
IPI00029469.1	ACTR1B	X	X		cytoskeleton,cytoplasm		protein binding,nucleotide binding	Beta-centractin
IPI00938347.1	LOC100287684	X			membrane			hypothetical protein XP_002342945
IPI00216798.5	MYL2			X	cytoskeleton,cytoplasm,cytosol	cell organization and biogenesis,development,regulation of biological process,cellular component movement,cell differentiation,cell growth	protein binding,metal ion binding,structural molecule activity	Myosin regulatory light chain 2, ventricular/cardiac muscle isoform
IPI00171145.7	SPIRE1		X		cytoskeleton,cytoplasm,Golgi	transport	protein binding	Isoform 1 of Protein spire homolog 1
IPI00013698.3	ASAH1			X			catalytic activity	N-acylsphingosine amidohydrolase (Acid ceramidase) 1, isoform CRA_c
IPI00018260.1	ARMC1		X		mitochondrion,cytoplasm	transport	protein binding,metal ion binding	Armadillo repeat-containing protein 1
IPI00470576.4	LRRC57	X	X				protein binding	Leucine-rich repeat-containing protein 57
IPI00437751.1	ACE			X	extracellular,cell surface,membrane,cytoplasm,endosome	cell death,cell proliferation,development,metabolic process,transport,regulation of biological process,response to stimulus,cellular component movement,defense response,cell differentiation	protein binding,metal ion binding,catalytic activity	Isoform Somatic-1 of Angiotensin-converting enzyme
IPI00023542.6	TMED9	X	X	X	membrane,endoplasmic reticulum,cytoplasm,Golgi	cell organization and biogenesis,transport,regulation of biological process	protein binding	Transmembrane emp24 domain-containing protein 9
IPI00299059.7	CHL1	X	X	X			protein binding	Isoform 2 of Neural cell adhesion molecule L1-like protein
IPI00020599.1	CALR	X	X	X	extracellular,cell surface,membrane,endoplasmic reticulum,cytoplasm,Golgi,organelle lumen,nucleus,cytosol	cell death,cell proliferation,development,cell organization and biogenesis,metabolic process,transport,regulation of biological process,response to stimulus,cell communication,cellular homeostasis,cell differentiation,reproduction	protein binding,DNA binding,RNA binding,metal ion binding	Calreticulin
IPI00926276.1	NAPEPLD	X	X				metal ion binding,catalytic activity	cDNA FLJ59821, highly similar to Homo sapiens N-acyl-phosphatidylethanolamine-hydrolyzing phospholipase D (NAPE-PLD), mRNA
IPI00442299.1	NRXN1	X	X		cell surface,membrane	cell organization and biogenesis,development,regulation of biological process,response to stimulus,cell communication,cellular homeostasis,cell differentiation	protein binding,metal ion binding,receptor activity	Isoform 1a of Neurexin-1-alpha
IPI00020396.1	PCSK6			X	extracellular,cell surface,membrane,endoplasmic reticulum,cytoplasm,Golgi,organelle lumen	development,metabolic process,transport,regulation of biological process,response to stimulus,cell communication	protein binding,signal transducer activity,nucleotide binding,receptor activity,catalytic activity	Isoform PACE4A-I of Proprotein convertase subtilisin/kexin type 6
IPI00181352.2	DNM2	X				metabolic process	protein binding,nucleotide binding,catalytic activity	dynamin-2 isoform 4
IPI00843819.1	CLK2P			X		metabolic process	nucleotide binding,catalytic activity	Similar to Dual specificity protein kinase CLK2
IPI00019158.3	ADAM8			X	cytoskeleton,cell surface,membrane,cytoplasm,vacuole	cell death,cell organization and biogenesis,development,transport,metabolic process,regulation of biological process,response to stimulus,cellular component movement,defense response,cell communication,cell differentiation	protein binding,metal ion binding,catalytic activity	Disintegrin and metalloproteinase domain-containing protein 8
IPI00760602.3	LYRM5		X					LYR motif-containing protein 5
IPI00218200.8	BCAP31	X	X		membrane,endoplasmic reticulum,cytoplasm,Golgi,cytosol	cell death,cell organization and biogenesis,transport,metabolic process,regulation of biological process,response to stimulus,cell communication,cellular homeostasis	protein binding	B-cell receptor-associated protein 31

IPI	GENE	Alzheimer's Hippocampus	Control hippocampus	CSF	Cellular localization	Biological process	Molecular function	Protein Description
IPI00033075.3	ABHD16A		X		membrane		catalytic activity	Abhydrolase domain-containing protein 16A
IPI00294242.2	MRPS31		X		mitochondrion,cytoplasm,ribosome		protein binding	28S ribosomal protein S31, mitochondrial
IPI00020692.4	SCN3A			X	membrane	transport,regulation of biological process	protein binding,transporter activity	Isoform 1 of Sodium channel protein type 3 subunit alpha
IPI00472855.1	HLA-A			X	membrane	response to stimulus	signal transducer activity,protein binding,receptor activity	HLA class I histocompatibility antigen, A-30 alpha chain
IPI00028193.4	KNDC1			X		regulation of biological process,response to stimulus,cell communication	enzyme regulator activity	Uncharacterized protein
IPI00006556.6	TRIL			X	membrane	regulation of biological process,response to stimulus,cell communication,defense response	protein binding	TLR4 interactor with leucine rich repeats
IPI00428511.2	NRXN1			X	cell surface,membrane,cytoplasm	development,cell organization and biogenesis,transport,regulation of biological process,response to stimulus,cell communication,cellular homeostasis	protein binding,metal ion binding	Neurexin-1-beta
IPI00102936.3	SRP68		X					Isoform 2 of Signal recognition particle 68 kDa protein
IPI00027481.2	ABCB1		X		cell surface,membrane,cytoplasm,Golgi	cell proliferation,transport,metabolic process,response to stimulus	protein binding,transporter activity,nucleotide binding,catalytic activity	Multidrug resistance protein 1
IPI00419847.4	SORBS3	X	X		cytoskeleton,membrane,cytoplasm,cytosol,nucleus	cell organization and biogenesis,metabolic process,regulation of biological process,response to stimulus,cell communication	protein binding,structural molecule activity	Isoform Alpha of Vinexin
IPI00009792.1	IGHV1OR15-1			X	extracellular	response to stimulus	protein binding	Ig heavy chain V-I region V35
IPI00783497.2	NUDT16	X	X		organelle lumen,nucleus		RNA binding,metal ion binding,catalytic activity	U8 snoRNA-decapping enzyme
IPI00184884.6	NSMCE1			X	chromosome,nucleus	metabolic process,regulation of biological process,response to stimulus,cell communication	protein binding,metal ion binding,catalytic activity	Non-structural maintenance of chromosomes element 1 homolog
IPI00023217.2	RYR2		X	X	membrane,endoplasmic reticulum,cytoplasm	cell death,development,transport,regulation of biological process,response to stimulus,cell communication,cellular homeostasis	protein binding,transporter activity,metal ion binding,receptor activity	Isoform 1 of Ryanodine receptor 2
IPI00008494.4	ICAM1	X	X	X	extracellular,cell surface,membrane	development,cell organization and biogenesis,metabolic process,transport,regulation of biological process,response to stimulus,cellular component movement,defense response,cell communication,cellular homeostasis,reproduction	signal transducer activity,protein binding,receptor activity	Intercellular adhesion molecule 1
IPI00796045.5	ATP6V0A1			X	membrane,cytoplasm,Golgi,organelle lumen,vacuole,nucleus,endosome	transport,regulation of biological process,response to stimulus,cell communication,cellular homeostasis	protein binding,transporter activity	Isoform 1 of V-type proton ATPase 116 kDa subunit a isoform 1
IPI00070943.4	PI4KA	X	X		cytoplasm,Golgi	metabolic process,regulation of biological process,response to stimulus,cell communication	protein binding,nucleotide binding,catalytic activity	Isoform 1 of Phosphatidylinositol 4-kinase alpha
IPI00011694.1	PRSS1	X			extracellular	cell organization and biogenesis,metabolic process	metal ion binding,catalytic activity	Trypsin-1
IPI00784739.1	C14orf43			X	organelle lumen,nucleus	metabolic process,regulation of biological process	DNA binding	Uncharacterized protein C14orf43
IPI00004315.1	SIGLEC9			X	membrane	regulation of biological process,response to stimulus,cell communication	protein binding	Sialic acid-binding Ig-like lectin 9
IPI00930452.1	IGKC			X			protein binding	A30
IPI00012102.1	GNS	X	X	X	cytoplasm,vacuole	metabolic process	protein binding,metal ion binding,catalytic activity	N-acetylglucosamine-6-sulfatase
IPI00375600.6	B3GALTL		X		membrane,endoplasmic reticulum,cytoplasm	metabolic process	catalytic activity	Beta-1,3-glucosyltransferase
IPI00297084.7	DDOST		X		membrane,endoplasmic reticulum,cytoplasm	metabolic process,transport,response to stimulus,defense response	catalytic activity	Dolichyl-diphosphooligosaccharide--protein glycosyltransferase 48 kDa subunit
IPI00304273.2	APOA4			X	extracellular,cytoskeleton,cell surface,endoplasmic reticulum,cytoplasm,organelle lumen	cell organization and biogenesis,transport,metabolic process,regulation of biological process,response to stimulus,defense response	antioxidant activity,protein binding,transporter activity,metal ion binding,catalytic activity,enzyme regulator activity	Apolipoprotein A-IV
IPI00216346.2	NOL3	X	X			cell death,regulation of biological process	protein binding	cDNA FLJ58768, highly similar to Homo sapiens nucleolar protein 3 (apoptosis repressor with CARD domain) (NOL3), mRNA
IPI00005722.5	FLT3			X	membrane	metabolic process,regulation of biological process,response to stimulus,cell communication	protein binding,signal transducer activity,nucleotide binding,receptor activity,catalytic activity	Uncharacterized protein
IPI00297982.7	EIF2S3	X	X		cytoplasm,cytosol	metabolic process	protein binding,RNA binding,nucleotide binding,catalytic activity	Eukaryotic translation initiation factor 2 subunit 3
IPI00026199.2	GPX3			X		metabolic process,response to stimulus	antioxidant activity,catalytic activity	Glutathione peroxidase 3
IPI00023728.1	GGH			X	extracellular,cytoplasm,vacuole,cytosol	metabolic process,response to stimulus	catalytic activity	Gamma-glutamyl hydrolase

IPI	GENE	Alzheimer's Hippocampus	Control hippocampus	CSF	Cellular localization	Biological process	Molecular function	Protein Description
IPI00026174.1	CCK			X	extracellular	cell death,cell proliferation,cell organization and biogenesis,development,metabolic process,regulation of biological process,response to stimulus,cellular component movement,cell communication,defense response,cellular homeostasis,cell differentiation	protein binding	Cholecystokinin
IPI00006362.1	EDF1	X	X				DNA binding	Isoform 2 of Endothelial differentiation-related factor 1
IPI00021389.1	CCS		X		mitochondrion,membrane,cytoplasm,cytosol,nucleus	transport,metabolic process,response to stimulus	antioxidant activity,protein binding,transporter activity,metal ion binding,catalytic activity	Copper chaperone for superoxide dismutase
IPI00007061.1	GOLT1B		X		membrane,endoplasmic reticulum,cytoplasm,Golgi	transport,regulation of biological process,response to stimulus,cell communication	signal transducer activity	Vesicle transport protein GOT1B
IPI00001453.2	INA	X	X		cytoskeleton	development,cell organization and biogenesis,response to stimulus,cell differentiation	structural molecule activity	Alpha-internexin
IPI00465044.2	RCC2			X	cytoskeleton,cytoplasm,organelle lumen,chromosome,cytosol,nucleus	cell organization and biogenesis,cell division		Protein RCC2
IPI00007280.10	ARPC5	X	X		cytoskeleton	cell organization and biogenesis,regulation of biological process		Isoform 2 of Actin-related protein 2/3 complex subunit 5
IPI00031627.4	POLR2A			X	organelle lumen,nucleus	metabolic process,regulation of biological process,response to stimulus,reproduction	protein binding,DNA binding,metal ion binding,catalytic activity	DNA-directed RNA polymerase II subunit RPB1
IPI00144243.2	HIVEP2			X	nucleus	metabolic process,regulation of biological process	DNA binding,metal ion binding	Transcription factor HIVEP2
IPI00293350.3	TSNAX	X	X		cytoplasm,nucleus	development,transport,reproduction,cell differentiation	DNA binding,transporter activity	Translin-associated protein X
IPI00515123.2	COMMD3		X			regulation of biological process,response to stimulus,cell communication	signal transducer activity,receptor activity,catalytic activity	COMM domain containing 3
IPI00179057.6	CUL4B	X	X			metabolic process	protein binding	Isoform 2 of Cullin-4B
IPI00419442.5	IGLV6-57			X			protein binding	Amyloid lambda 6 light chain variable region PIP
IPI00022033.6	RAB11FIP5		X		cytoskeleton,membrane,mitochondrion,cytoplasm,endosome	transport	protein binding	Rab11 family-interacting protein 5
IPI00027014.1	DCTN3	X	X		cytoskeleton,cytoplasm,chromosome,cytosol	cell organization and biogenesis,cell division	protein binding,structural molecule activity	Isoform 1 of Dynactin subunit 3
IPI00292530.1	ITIH1			X	extracellular	metabolic process,regulation of biological process	protein binding,metal ion binding,enzyme regulator activity	Inter-alpha-trypsin inhibitor heavy chain H1
IPI00829590.1	IGHV3-72			X				13 kDa protein
IPI00146935.4	DNM1L	X	X		cytoskeleton,membrane,mitochondrion,cytoplasm,Golgi,cytosol	cell death,cell organization and biogenesis,metabolic process,transport,regulation of biological process,response to stimulus,cell communication	protein binding,nucleotide binding,catalytic activity	Isoform 1 of Dynamin-1-like protein
IPI00855918.1	MUC5B			X			protein binding	Uncharacterized protein
IPI00399007.7	IGHG2	X	X	X	membrane		protein binding	Putative uncharacterized protein DKFZp686I04196 (Fragment)
IPI00064935.6	ALPK3			X	nucleus	development,metabolic process	protein binding,nucleotide binding,catalytic activity	Alpha-protein kinase 3
IPI00294834.6	ASPH	X	X	X	membrane,endoplasmic reticulum,cytoplasm	cell proliferation,development,transport,metabolic process,regulation of biological process,response to stimulus,cell communication,cellular homeostasis	protein binding,metal ion binding,structural molecule activity,catalytic activity	Isoform 1 of Aspartyl/asparaginyl beta-hydroxylase
IPI00477611.1	COL5A1			X	extracellular		structural molecule activity	Collagen type V alpha 1
IPI00021594.3	GPAA1			X	membrane,endoplasmic reticulum,cytoplasm	cell organization and biogenesis,metabolic process	protein binding,catalytic activity	Isoform 1 of Glycosylphosphatidylinositol anchor attachment 1 protein
IPI00442544.1	LOC100507079			X				CDNA FLJ27034 fis, clone SLV07984
IPI00011250.3	UCHL3	X	X		mitochondrion,cytoplasm,nucleus	metabolic process	protein binding,catalytic activity	Ubiquitin carboxyl-terminal hydrolase isozyme L3
IPI00010720.1	CCT5	X	X		cytoskeleton,cytoplasm,organelle lumen,nucleus,cytosol	metabolic process,response to stimulus	protein binding,nucleotide binding	T-complex protein 1 subunit epsilon
IPI00019141.1	AGPAT1	X	X		membrane,endoplasmic reticulum,cytoplasm	metabolic process,regulation of biological process,response to stimulus,cell communication	catalytic activity	1-acyl-sn-glycerol-3-phosphate acyltransferase alpha
IPI00007800.1	ANGPTL2			X	extracellular	development,regulation of biological process,response to stimulus,cell communication	protein binding	Angiopoietin-related protein 2
IPI00398002.6	PLEC	X	X		cytoskeleton,membrane		protein binding	Isoform 3 of Plectin
IPI00010154.3	GDI1	X	X	X	cytoplasm,cytosol	transport,metabolic process,regulation of biological process,response to stimulus,cell communication	protein binding,enzyme regulator activity	Rab GDP dissociation inhibitor alpha
IPI00000494.6	RPL5	X	X		cytoplasm,ribosome,organelle lumen,cytosol,nucleus	cell organization and biogenesis,transport,metabolic process,reproduction	protein binding,RNA binding,structural molecule activity	60S ribosomal protein L5

IPI	GENE	Alzheimer's Hippocampus	Control hippocampus	CSF	Cellular localization	Biological process	Molecular function	Protein Description
IPI00022296.1	KIT			X	extracellular,cell surface,membrane,cytoplasm,nucleus	cell death,development,cell organization and biogenesis,metabolic process,regulation of biological process,response to stimulus,defense response,reproduction,cell proliferation,transport,cellular component movement,cell communication,cell differentiation	signal transducer activity,protein binding,metal ion binding,nucleotide binding,receptor activity,catalytic activity	Isoform 1 of Mast/stem cell growth factor receptor
IPI00784044.1	MCCC2	X	X	X	mitochondrion,membrane,cytoplasm,organelle lumen	metabolic process	nucleotide binding,catalytic activity	Isoform 1 of Methylcrotonoyl-CoA carboxylase beta chain, mitochondrial
IPI00168862.3	PXT1			X	cytoplasm			Peroxisomal testis-specific protein 1
IPI00007068.1	ACTR3B	X	X	X	cytoskeleton,cytoplasm	cell organization and biogenesis,regulation of biological process	protein binding,nucleotide binding	Isoform 1 of Actin-related protein 3B
IPI00023461.2	MLLT4	X	X		membrane,cytoplasm,organelle lumen,nucleus,cytosol	cell organization and biogenesis,regulation of biological process,response to stimulus,cell communication	protein binding	Isoform 4 of Afadin
IPI00796830.1	A2M			X				13 kDa protein
IPI00064296.1	LAMTOR3			X				PRO0633
IPI00020017.1	C10orf116	X	X					Adipose most abundant gene transcript 2 protein
IPI00019581.1	F12			X	extracellular	metabolic process	protein binding,catalytic activity	coagulation factor XII (Hageman factor), isoform CRA_b
IPI00009145.1	MAN1A2			X	membrane,cytoplasm,Golgi,organelle lumen,nucleus	development,metabolic process	metal ion binding,catalytic activity	Mannosyl-oligosaccharide 1,2-alpha-mannosidase IB
IPI00101095.3	C20orf27	X	X					Isoform 1 of UPF0687 protein C20orf27
IPI00032416.7	JAG2			X	membrane	cell death,cell proliferation,development,metabolic process,regulation of biological process,response to stimulus,cellular component movement,cell communication,reproduction,cell differentiation	protein binding,metal ion binding	Isoform Long of Protein jagged-2
IPI00329600.3	SCCPDH	X	X		mitochondrion,cytoplasm	metabolic process	nucleotide binding,catalytic activity	Probable saccharopine dehydrogenase
IPI00005969.3	CAPZA1	X	X	X	extracellular,cytoskeleton,cytoplasm,cytosol	cell organization and biogenesis,regulation of biological process,response to stimulus,cellular component movement,defense response,coagulation	protein binding	F-actin-capping protein subunit alpha-1
IPI00431025.1	ABI1	X	X		cytoskeleton,membrane,endoplasmic reticulum,cytoplasm,nucleus,cytosol	cell proliferation,development,cell organization and biogenesis,metabolic process,regulation of biological process,response to stimulus,cellular component movement,cell communication	protein binding,enzyme regulator activity	Isoform 1 of Abl interactor 1
IPI00027429.1	FABP7	X	X	X		transport	transporter activity	cDNA FLJ75346
IPI00011996.5	UBE2Z	X	X		cytoplasm,organelle lumen,nucleus	cell death,metabolic process	nucleotide binding,catalytic activity	Isoform 1 of Ubiquitin-conjugating enzyme E2 Z
IPI00440580.3	MANEAL			X	membrane,cytoplasm,Golgi		catalytic activity	Isoform 1 of Glycoprotein endo-alpha-1,2-mannosidase-like protein
IPI00217966.9	LDHA	X	X	X	mitochondrion,cytoplasm,cytosol	metabolic process,response to stimulus,cell communication	nucleotide binding,catalytic activity	Isoform 1 of L-lactate dehydrogenase A chain
IPI00029702.1	PTK2B		X		cytoskeleton,membrane,cytoplasm,organelle lumen,cytosol,nucleus	cell death,cell organization and biogenesis,development,metabolic process,regulation of biological process,response to stimulus,reproduction,cell proliferation,transport,cellular component movement,cell communication,cellular homeostasis,cell differentiation,cell growth	protein binding,signal transducer activity,nucleotide binding,catalytic activity	Isoform 1 of Protein-tyrosine kinase 2-beta
IPI00073763.4	SEMA4C	X		X	cytoskeleton,membrane,cytoplasm	development,regulation of biological process,response to stimulus,cellular component movement,cell communication,cell differentiation	protein binding,receptor activity	Semaphorin-4C
IPI00937615.2	EEF1G	X	X		cytoplasm,cytosol	metabolic process,response to stimulus	protein binding,RNA binding	Elongation factor 1-gamma
IPI00844422.1	IGKC			X			protein binding	immunoblobulin light chain
IPI00219425.3	PVR			X				Isoform Beta of Poliovirus receptor
IPI00007736.5	EIF2C1	X			cytoplasm,cytosol	metabolic process,regulation of biological process,response to stimulus,cell communication	protein binding,RNA binding	Protein argonaute-1
IPI00382470.3	HSP90AA1	X	X			metabolic process,response to stimulus	protein binding,nucleotide binding	Isoform 2 of Heat shock protein HSP 90-alpha
IPI00027378.5	UBXN1	X			membrane,endoplasmic reticulum,cytoplasm,proteasome	metabolic process,regulation of biological process	protein binding	Isoform 1 of UBX domain-containing protein 1
IPI00854624.3	IGHV2-70			X				Ig heavy chain V-II region COR

IPI	GENE	Alzheimer's Hippocampus	Control hippocampus	CSF	Cellular localization	Biological process	Molecular function	Protein Description
IPI00024911.1	ERP29	X	X		endoplasmic reticulum,cytoplasm,organelle lumen	metabolic process,transport	catalytic activity	Endoplasmic reticulum resident protein 29
IPI01012723.1	UCKL1			X		metabolic process	nucleotide binding,catalytic activity	Uncharacterized protein
IPI00217536.3	RHOT1	X	X			regulation of biological process,response to stimulus,cell communication	nucleotide binding	Isoform 3 of Mitochondrial Rho GTPase 1
IPI00885081.1	HDGFRP2	X	X			metabolic process,regulation of biological process		hepatoma-derived growth factor-related protein 2 isoform 2
IPI00027038.1	VSIG4			X	membrane	cell proliferation,metabolic process,regulation of biological process,response to stimulus,defense response	protein binding	Isoform 1 of V-set and immunoglobulin domain-containing protein 4
IPI00291922.2	PSMA5	X	X	X	proteasome,cytoplasm,organelle lumen,nucleus,cytosol	cell death,metabolic process,regulation of biological process,response to stimulus,cell communication	protein binding,catalytic activity	Proteasome subunit alpha type-5
IPI00305833.3	SMU1			X	cytoplasm,nucleus		protein binding	WD40 repeat-containing protein SMU1
IPI00171199.5	PSMA3	X	X	X	proteasome	metabolic process	catalytic activity	Isoform 2 of Proteasome subunit alpha type-3
IPI00435925.1	IGFBP3			X		cell death,cell organization and biogenesis,development,regulation of biological process,response to stimulus,cell communication,cell differentiation,cell growth	protein binding,enzyme regulator activity	PP14214
IPI00008868.4	MAP1B	X	X		cytoskeleton,membrane,cytoplasm,cytosol	development,cell organization and biogenesis,transport,regulation of biological process,response to stimulus,cellular component movement,cell communication,cell differentiation,cell growth	protein binding,structural molecule activity	Microtubule-associated protein 1B
IPI00292946.1	SERPINA7			X	extracellular	development,metabolic process,regulation of biological process,response to stimulus	enzyme regulator activity	Thyroxine-binding globulin
IPI00012451.3	GNB4	X			membrane	metabolic process,regulation of biological process,response to stimulus,cell communication	signal transducer activity,protein binding	Guanine nucleotide-binding protein subunit beta-4
IPI00978163.2	DIAPH1			X	cytoskeleton,membrane,cytoplasm	cell organization and biogenesis	protein binding,motor activity,catalytic activity	Uncharacterized protein
IPI00784430.5	IGKV3-11			X	extracellular,membrane	metabolic process,regulation of biological process,response to stimulus,defense response	protein binding	Ig kappa chain V-III region VG (Fragment)
IPI00470674.5	CYB5R1		X		membrane,mitochondrion,cytoplasm	metabolic process	catalytic activity	NADH-cytochrome b5 reductase 1
IPI00910552.1	DTNA	X	X					dystrobrevin alpha isoform 16
IPI00002311.3	SCRN3	X	X			metabolic process	catalytic activity	SCRN3 protein
IPI00019213.1	DLG4		X				protein binding	Isoform 2 of Disks large homolog 4
IPI00012107.2	FAAH	X	X		cytoskeleton,membrane,cytoplasm	metabolic process	protein binding,catalytic activity	Fatty-acid amide hydrolase 1
IPI00220194.6	SLC2A1	X	X		membrane,cytoplasm,nucleus	transport,metabolic process,regulation of biological process,response to stimulus,cell communication	protein binding,transporter activity	Solute carrier family 2, facilitated glucose transporter member 1
IPI00031131.4	APMAP	X	X	X	cell surface,membrane	metabolic process	catalytic activity	Isoform 1 of Adipocyte plasma membrane-associated protein
IPI00217960.1	PRKACA	X	X			metabolic process	nucleotide binding,catalytic activity	Isoform 2 of cAMP-dependent protein kinase catalytic subunit alpha
IPI00017763.8	NAP1L4	X	X		nucleus	cell organization and biogenesis,metabolic process		Uncharacterized protein
IPI00412498.2	NAPRT1	X			cytoplasm,Golgi,nucleus	metabolic process	catalytic activity	Uncharacterized protein
IPI00299083.5	JAM2			X	membrane	response to stimulus,cellular component movement,coagulation	protein binding	Junctional adhesion molecule B
IPI00304925.5	HSPA1B	X	X	X	mitochondrion,endoplasmic reticulum,cytoplasm,organelle lumen,cytosol,nucleus	cell death,cell proliferation,cell organization and biogenesis,metabolic process,regulation of biological process,response to stimulus,cell growth	protein binding,nucleotide binding,receptor activity	Heat shock 70 kDa protein 1A/1B
IPI00451132.2	PDE2A	X	X		membrane,mitochondrion,endoplasmic reticulum,cytoplasm,Golgi,organelle lumen,cytosol,nucleus	cell organization and biogenesis,development,metabolic process,transport,regulation of biological process,response to stimulus,defense response,cell communication,cell differentiation,coagulation	protein binding,transporter activity,metal ion binding,nucleotide binding,catalytic activity	cGMP-dependent 3',5'-cyclic phosphodiesterase
IPI00413641.7	AKR1B1	X	X	X	extracellular,cytoplasm,organelle lumen,nucleus,cytosol	metabolic process,response to stimulus	catalytic activity	Aldose reductase
IPI00910886.1	SPARCL1			X	extracellular	regulation of biological process,response to stimulus,cell communication	protein binding,metal ion binding	cDNA FLJ54387, moderately similar to SPARC-like protein 1
IPI00025700.2	CD6			X	cell surface,membrane		receptor activity	Isoform CD6A of T-cell differentiation antigen CD6
IPI00807609.1	CLEC11A			X	extracellular			Aberrant LSLCL

IPI	GENE	Alzheimer's Hippocampus	Control hippocampus	CSF	Cellular localization	Biological process	Molecular function	Protein Description
IPI00018755.1	HMGB1P10			X			protein binding	Putative high mobility group protein 1-like 10
IPI00219678.3	EIF2S1	X	X		cytoplasm,cytosol,nucleus	metabolic process,regulation of biological process,response to stimulus,cell communication	protein binding,RNA binding	Eukaryotic translation initiation factor 2 subunit 1
IPI00010777.3	FGFR2			X	extracellular,cell surface,membrane,cytoplasm,Golgi,nucleus	cell death,cell proliferation,development,cell organization and biogenesis,cell division,metabolic process,regulation of biological process,response to stimulus,cell communication,reproduction,cell differentiation	protein binding,signal transducer activity,nucleotide binding,receptor activity,catalytic activity	fibroblast growth factor receptor 2 isoform 1 precursor
IPI00645192.4	MECP2	X	X		nucleus		DNA binding	Mutant methyl CpG binding protein 2 variant 1
IPI00847609.3	SVEP1			X	extracellular,cell surface,membrane,cytoplasm		protein binding,metal ion binding	sushi, von Willebrand factor type A, EGF and pentraxin domain-containing protein 1 precursor
IPI00221088.5	RPS9	X			cytoplasm,ribosome,organelle lumen,cytosol,nucleus	cell proliferation,cell organization and biogenesis,metabolic process,transport,regulation of biological process,reproduction	protein binding,RNA binding,translation regulator activity,structural molecule activity	40S ribosomal protein S9
IPI00008091.2	INO80			X	cytoskeleton,organelle lumen,chromosome,nucleus	cell organization and biogenesis,cell division,metabolic process,regulation of biological process,response to stimulus,cell growth	protein binding,DNA binding,nucleotide binding,catalytic activity	DNA helicase INO80
IPI00026846.2	SLC6A1	X	X		cell surface,membrane	cell organization and biogenesis,transport,regulation of biological process,response to stimulus,cell communication	transporter activity	Sodium- and chloride-dependent GABA transporter 1
IPI00413959.2	CLSTN1			X	extracellular,membrane,endoplasmic reticulum,cytoplasm,Golgi,nucleus		metal ion binding	Isoform 1 of Calsyntenin-1 (Fragment)
IPI00829640.1	IGL@			X			protein binding	IGL@ protein
IPI00011264.2	CFHR1			X	extracellular	metabolic process,regulation of biological process,response to stimulus		Complement factor H-related protein 1
IPI00220117.2	CD99			X		regulation of biological process,response to stimulus,cell communication	signal transducer activity,receptor activity	Uncharacterized protein
IPI00306280.4	DENR	X	X			metabolic process	protein binding,RNA binding	Density-regulated protein
IPI00014310.3	CUL1	X	X		cytoplasm,organelle lumen,cytosol,nucleus	cell death,cell proliferation,development,metabolic process,regulation of biological process,response to stimulus,cell communication	protein binding	Cullin-1
IPI00001734.3	PSAT1	X	X	X		metabolic process	catalytic activity	Phosphoserine aminotransferase
IPI00072377.1	SET	X	X		endoplasmic reticulum,cytoplasm,organelle lumen,nucleus,cytosol	cell death,cell organization and biogenesis,transport,metabolic process,regulation of biological process	protein binding,DNA binding,enzyme regulator activity	Isoform 1 of Protein SET
IPI00218628.4	ITGA2B			X				Isoform 2 of Integrin alpha-IIb
IPI00549189.4	THOP1	X	X		cytoplasm	metabolic process,regulation of biological process,response to stimulus,cell communication	metal ion binding,catalytic activity	Thimet oligopeptidase
IPI00304600.5	CAMKV	X	X		membrane	metabolic process,reproduction	nucleotide binding,catalytic activity	Isoform 3 of CaM kinase-like vesicle-associated protein
IPI00295767.4	OLFM2			X	extracellular		protein binding	Noelin-2
IPI00791573.1	SUGT1	X	X				protein binding	Isoform 2 of Suppressor of G2 allele of SKP1 homolog
IPI00014978.3	PPP2R5A	X	X		membrane,cytoplasm,chromosome,nucleus	cell organization and biogenesis,metabolic process,regulation of biological process,response to stimulus,cell communication	protein binding,enzyme regulator activity	Serine/threonine-protein phosphatase 2A 56 kDa regulatory subunit alpha isoform
IPI00874023.2	ZNF818P			X	nucleus	metabolic process,regulation of biological process	DNA binding,metal ion binding	Putative zinc finger protein 818
IPI00102069.5	EIF3M	X	X		cytoplasm	metabolic process	protein binding,RNA binding	Eukaryotic translation initiation factor 3 subunit M
IPI00024272.1	DGCR2			X	membrane	development	protein binding,receptor activity	Integral membrane protein DGCR2/IDD
IPI00007943.1	STAMBP	X	X		membrane,cytoplasm,organelle lumen,nucleus,endosome	cell death,cell proliferation,metabolic process,regulation of biological process,response to stimulus,cell communication	protein binding,metal ion binding,catalytic activity	STAM-binding protein
IPI00843975.1	EZR	X	X		cytoskeleton,membrane,cytoplasm,organelle lumen,cytosol,nucleus	development,cell organization and biogenesis,regulation of biological process,response to stimulus,cell differentiation	protein binding	Ezrin
IPI00005737.1	SURF4	X	X		membrane,endoplasmic reticulum,cytoplasm,Golgi	cell organization and biogenesis,transport,regulation of biological process	protein binding	Isoform 1 of Surfeit locus protein 4
IPI00031769.1	C2orf40			X	extracellular,cytoplasm			Augurin
IPI00060310.5	PLD4			X		metabolic process	catalytic activity	cDNA FLJ56630, highly similar to Homo sapiens phospholipase D family, member 4 (PLD4), mRNA

IPI	GENE	Alzheimer's Hippocampus	Control hippocampus	CSF	Cellular localization	Biological process	Molecular function	Protein Description
IPI00293396.5	AP1G1	X	X	X	membrane,cytoplasm,Golgi	transport	transporter activity	Isoform 2 of AP-1 complex subunit gamma-1
IPI00783464.3	DNAH2			X	cytoskeleton,cytoplasm	metabolic process,cellular component movement	nucleotide binding,motor activity,catalytic activity	Isoform 1 of Dynein heavy chain 2, axonemal
IPI00023549.5	LGALSL	X	X					Galectin-related protein
IPI00218725.3	LAMA2	X		X	extracellular,cytoskeleton,membrane,mitochondrion,cytoplasm,organelle lumen,chromosome,nucleus,cytosol	development,cell organization and biogenesis,transport,metabolic process,regulation of biological process,response to stimulus,cellular component movement,cell communication,reproduction	signal transducer activity,protein binding,motor activity,catalytic activity	laminin subunit alpha-2 isoform b precursor
IPI00005728.2	RER1	X	X		membrane			Uncharacterized protein
IPI00014137.3	OSBPL2	X	X			transport		Isoform 1 of Oxysterol-binding protein-related protein 2
IPI00300568.4	SYN1	X	X		membrane,cytoplasm,Golgi,organelle lumen,chromosome,nucleus	metabolic process,transport,cell communication	protein binding,transporter activity,nucleotide binding,catalytic activity	Isoform IA of Synapsin-1
IPI00217005.7	ANKRD18A			X			protein binding	Ankyrin repeat domain-containing protein 18A
IPI00761159.1	IGHM			X			protein binding	IGHM protein
IPI00419194.2	IAH1	X	X			metabolic process	catalytic activity	Isoamyl acetate-hydrolyzing esterase 1 homolog
IPI00103467.5	ALDH1B1	X	X		mitochondrion,cytoplasm,organelle lumen,nucleus	metabolic process	catalytic activity	Aldehyde dehydrogenase X, mitochondrial
IPI00171928.1	ANGPTL7			X	extracellular	regulation of biological process,response to stimulus,cell communication	protein binding	Angiopoietin-related protein 7
IPI00783982.1	COPG1		X		membrane,cytoplasm,Golgi,nucleus,cytosol	cell organization and biogenesis,transport,cellular component movement	structural molecule activity	Coatomer subunit gamma
IPI00470619.4	GNG2	X	X		membrane	cell proliferation,metabolic process,regulation of biological process,response to stimulus,cell communication,coagulation	signal transducer activity	Guanine nucleotide-binding protein G(I)/G(S)/G(O) subunit gamma-2
IPI00304417.7	IDH3B	X	X			metabolic process	catalytic activity	Isocitrate dehydrogenase [NAD] subunit beta, mitochondrial precursor
IPI00419585.9	PPIA	X	X	X	extracellular,cytoplasm,cytosol,nucleus	metabolic process,transport,regulation of biological process,response to stimulus,cellular component movement,reproduction,coagulation	protein binding,catalytic activity	Peptidyl-prolyl cis-trans isomerase A
IPI00293009.4	RABEP1	X	X		membrane,cytoplasm,endosome	cell death,cell organization and biogenesis,metabolic process,transport,regulation of biological process	protein binding,transporter activity,enzyme regulator activity	Isoform 1 of Rab GTPase-binding effector protein 1
IPI00026946.2	NPTX2		X	X	extracellular	cell communication	metal ion binding	Neuronal pentraxin-2
IPI00152216.2	RIC3			X	membrane,endoplasmic reticulum,cytoplasm,Golgi	cell organization and biogenesis,metabolic process	protein binding	Isoform 1 of Protein RIC-3
IPI00008860.1	C1QTNF3			X	extracellular	metabolic process,regulation of biological process	protein binding	Isoform 1 of Complement C1q tumor necrosis factor-related protein 3
IPI00027342.1	CAP2	X	X		membrane	development,cell organization and biogenesis,metabolic process,regulation of biological process,response to stimulus,cell communication,cell differentiation	protein binding	Adenylyl cyclase-associated protein 2
IPI00552943.3	IGLV1-36			X				V1-11 protein
IPI00001589.1	TIMM13	X	X		mitochondrion,membrane,cytoplasm	cell organization and biogenesis,metabolic process,transport	metal ion binding	Mitochondrial import inner membrane translocase subunit Tim13
IPI00892713.1	ADAP1		X			metabolic process,regulation of biological process	protein binding,metal ion binding,enzyme regulator activity	cDNA FLJ77789
IPI00217791.7	CCDC105			X	cytoskeleton	cell organization and biogenesis		Coiled-coil domain-containing protein 105
IPI00023095.1	MLF2		X		cytoplasm,nucleus	response to stimulus,defense response		Myeloid leukemia factor 2
IPI00009057.2	G3BP2	X	X		cytoplasm,cytosol	transport,regulation of biological process,response to stimulus,cell communication	protein binding,RNA binding,nucleotide binding,structural molecule activity	Isoform A of Ras GTPase-activating protein-binding protein 2
IPI00305975.5	SPON2			X	extracellular	development,cell organization and biogenesis,response to stimulus,defense response,cell differentiation	protein binding,metal ion binding	Spondin-2
IPI00400967.5	KIAA1843			X	membrane			Isoform 2 of Uncharacterized protein KIAA1843
IPI00876962.2	INF2	X	X			cell organization and biogenesis	protein binding	Isoform 2 of Inverted formin-2
IPI00034205.1	AIF1L	X	X		cytoskeleton,membrane,cytoplasm		protein binding,metal ion binding	Isoform 1 of Allograft inflammatory factor 1-like

IPI	GENE	Alzheimer's Hippocampus	Control hippocampus	CSF	Cellular localization	Biological process	Molecular function	Protein Description
IPI00021695.1	ATP2B1	X	X	X	membrane	metabolic process,transport,response to stimulus,coagulation	protein binding,transporter activity,metal ion binding,nucleotide binding,catalytic activity	Isoform D of Plasma membrane calcium-transporting ATPase 1
IPI00477992.1	C1QB			X	extracellular,membrane	development,metabolic process,regulation of biological process,response to stimulus,defense response	protein binding	Complement C1q subcomponent subunit B
IPI00014312.1	CUL3	X	X		cytoskeleton,cytoplasm,Golgi,nucleus	cell death,cell proliferation,development,cell organization and biogenesis,cell division,metabolic process,transport,regulation of biological process,response to stimulus,cellular component movement,cell communication,cell differentiation	protein binding,catalytic activity	Isoform 1 of Cullin-3
IPI00001952.5	ENDOD1	X		X	extracellular	metabolic process	metal ion binding,catalytic activity	Endonuclease domain-containing 1 protein
IPI00004497.2	BCR		X		membrane,cytoplasm,Golgi,cytosol	development,cell organization and biogenesis,transport,metabolic process,regulation of biological process,response to stimulus,cellular component movement,defense response,cell communication	protein binding,nucleotide binding,structural molecule activity,catalytic activity,enzyme regulator activity	Isoform 1 of Breakpoint cluster region protein
IPI00020885.1	DYNLT3	X	X		cytoskeleton,membrane,cytoplasm,chromosome,nucleus	cell organization and biogenesis,cell division,transport,regulation of biological process	protein binding,motor activity,catalytic activity	Dynein light chain Tctex-type 3
IPI00013933.2	DSP			X	extracellular,cytoskeleton,membrane,mitochondrion,cytoplasm,organelle lumen,chromosome,nucleus	cell death,development,cell organization and biogenesis,metabolic process,regulation of biological process,response to stimulus,cellular component movement,cell differentiation,reproduction	protein binding,motor activity,structural molecule activity,catalytic activity	Isoform DPI of Desmoplakin
IPI00218398.6	MMP14			X	membrane,cytoplasm,Golgi,organelle lumen	cell proliferation,development,cell organization and biogenesis,metabolic process,regulation of biological process,response to stimulus,cellular component movement,reproduction,cell differentiation	protein binding,metal ion binding,catalytic activity,enzyme regulator activity	Matrix metalloproteinase-14
IPI00024621.3	OLFML3			X	extracellular	development	protein binding	Isoform 1 of Olfactomedin-like protein 3
IPI00006470.3	HMP19			X	membrane,cytoplasm,Golgi,endosome	regulation of biological process,response to stimulus,cell communication	protein binding	Neuron-specific protein family member 2
IPI00328415.13	CYB5R3	X	X		mitochondrion,membrane,endoplasmic reticulum,cytoplasm,cytosol	metabolic process	catalytic activity	Isoform 1 of NADH-cytochrome b5 reductase 3
IPI00657742.1	HLA-F			X	membrane	response to stimulus	protein binding	Major histocompatibility complex, class I, F
IPI00184670.4	AMPD2	X	X			metabolic process	catalytic activity	Isoform Ex1A-2-3 of AMP deaminase 2
IPI00642632.2	IGLC7			X	extracellular,membrane	metabolic process,regulation of biological process,response to stimulus,defense response	protein binding	Ig lambda-7 chain C region
IPI00006987.1	DDX24			X	cytoplasm,organelle lumen,nucleus	metabolic process	RNA binding,nucleotide binding,catalytic activity	ATP-dependent RNA helicase DDX24
IPI00010148.4	PCP4	X	X	X	cytoplasm,cytosol,nucleus	development	protein binding	Purkinje cell protein 4
IPI00032995.1	LANCL2	X	X		cytoskeleton,membrane,cytoplasm,cytosol,nucleus	metabolic process,regulation of biological process,response to stimulus,cell communication	nucleotide binding,catalytic activity	LanC-like protein 2
IPI00453458.3	NCOA7	X	X		nucleus	metabolic process,regulation of biological process	protein binding	Isoform 1 of Nuclear receptor coactivator 7
IPI00878576.5	ENPP2			X	membrane	response to stimulus	metal ion binding,receptor activity,catalytic activity	Isoform 3 of Ectonucleotide pyrophosphatase/phosphodiesterase family member 2
IPI00183171.5	SIRT3		X		membrane	metabolic process	metal ion binding,nucleotide binding,catalytic activity	cDNA FLJ60939, highly similar to NAD-dependent deacetylase sirtuin-3, mitochondrial
IPI00414676.6	HSP90AB1	X	X	X	mitochondrion,membrane,cytoplasm,cytosol,nucleus	development,cell organization and biogenesis,metabolic process,regulation of biological process,response to stimulus,defense response,cell communication,cell differentiation	protein binding,nucleotide binding,enzyme regulator activity	Heat shock protein HSP 90-beta
IPI00902969.2	GFER		X		extracellular,mitochondrion,cytoplasm	cell proliferation,cell organization and biogenesis,metabolic process,transport,reproduction	protein binding,catalytic activity	Isoform 1 of FAD-linked sulfhydryl oxidase ALR
IPI00031030.1	APLP2	X	X	X	membrane,nucleus	development,metabolic process,regulation of biological process,response to stimulus,cell communication,cellular homeostasis,reproduction	protein binding,DNA binding,metal ion binding,enzyme regulator activity	Isoform 1 of Amyloid-like protein 2
IPI00551022.3	ISOC2	X	X		mitochondrion,cytoplasm,nucleus	metabolic process,regulation of biological process	protein binding,catalytic activity	Isoform 1 of Isochorismatase domain-containing protein 2, mitochondrial
IPI00291483.4	AKR1C3	X	X		cytoplasm,nucleus	cell death,cell proliferation,development,transport,metabolic process,regulation of biological process,response to stimulus,cell communication,cell differentiation,reproduction	signal transducer activity,receptor activity,catalytic activity	Aldo-keto reductase family 1 member C3
IPI00296374.3	ZFPL1			X	membrane,cytoplasm,Golgi,nucleus	transport,metabolic process,regulation of biological process	protein binding,DNA binding,metal ion binding	Zinc finger protein-like 1

IPI	GENE	Alzheimer's Hippocampus	Control hippocampus	CSF	Cellular localization	Biological process	Molecular function	Protein Description
IPI00179473.9	SQSTM1			X	endoplasmic reticulum,cytoplasm,organelle lumen,nucleus,cytosol,endosome	cell death,metabolic process,transport,regulation of biological process,response to stimulus,cell communication,cell differentiation	protein binding,metal ion binding,catalytic activity	Isoform 1 of Sequestosome-1
IPI00025204.1	CD5L			X	extracellular,membrane	cell death,response to stimulus,defense response	receptor activity	CD5 antigen-like
IPI00292020.3	SRM	X	X		cytoplasm,cytosol	metabolic process	protein binding,catalytic activity	Spermidine synthase
IPI00019176.3	RARRES2			X	extracellular	development,metabolic process,regulation of biological process,response to stimulus,cellular component movement,cell differentiation	protein binding,receptor activity	Retinoic acid receptor responder protein 2
IPI00013723.3	PIN1	X	X		cytoplasm,organelle lumen,nucleus	cell proliferation,cell organization and biogenesis,metabolic process,cell division,regulation of biological process,response to stimulus,cellular component movement,defense response,cell communication	protein binding,DNA binding,catalytic activity	Peptidyl-prolyl cis-trans isomerase NIMA-interacting 1
IPI00216008.4	G6PD	X	X			metabolic process	nucleotide binding,catalytic activity	Isoform Long of Glucose-6-phosphate 1-dehydrogenase
IPI00302673.4	ATPAF1		X		mitochondrion,cytoplasm	cell organization and biogenesis		ATP synthase mitochondrial F1 complex assembly factor 1 isoform 1 precursor
IPI00472614.3	ERC2		X		cytoskeleton,membrane,cytoplasm	transport	protein binding,transporter activity	ERC protein 2
IPI00940069.1	IGKC			X			protein binding	25 kDa protein
IPI00166642.3	D2HGDH	X	X		mitochondrion,cytoplasm,organelle lumen	metabolic process,response to stimulus	catalytic activity	Isoform 1 of D-2-hydroxyglutarate dehydrogenase, mitochondrial
IPI00952583.2	MDH1	X	X		cytoskeleton,membrane,mitochondrion,cytoplasm	metabolic process	nucleotide binding,catalytic activity	malate dehydrogenase, cytoplasmic isoform 1
IPI00219012.4	FYN	X	X		membrane,cytoplasm,cytosol,endosome	development,cell organization and biogenesis,transport,metabolic process,regulation of biological process,response to stimulus,cellular component movement,defense response,cell communication,cell differentiation,coagulation	protein binding,metal ion binding,nucleotide binding,catalytic activity	Isoform 1 of Tyrosine-protein kinase Fyn
IPI00479385.4	ASMTL	X	X		cytoplasm	metabolic process	catalytic activity	Isoform 1 of N-acetylserotonin O-methyltransferase-like protein
IPI00009901.1	NUTF2	X	X	X	cytoskeleton,membrane,cytoplasm,cytosol,nucleus	transport	protein binding,transporter activity	Nuclear transport factor 2
IPI00290305.3	TP53RK		X		membrane,nucleus	metabolic process	protein binding,nucleotide binding,catalytic activity	TP53-regulating kinase
IPI00739827.1	LAMP2			X	membrane			Isoform LAMP-2B of Lysosome-associated membrane glycoprotein 2
IPI00008495.5	ND4L	X	X		mitochondrion,membrane,cytoplasm	development,metabolic process,transport,response to stimulus	catalytic activity	NADH-ubiquinone oxidoreductase chain 4
IPI00010951.2	EPPK1	X	X		cytoskeleton,cytoplasm		structural molecule activity	Epiplakin
IPI00328257.5	AP1B1	X	X	X	membrane,cytoplasm,Golgi,vacuole,cytosol	cell organization and biogenesis,transport,regulation of biological process,response to stimulus,defense response	protein binding,transporter activity	Isoform A of AP-1 complex subunit beta-1
IPI00011094.2	C1QTNF4			X	extracellular		protein binding	Complement C1q tumor necrosis factor-related protein 4
IPI00306576.1	ARSB			X	mitochondrion,endoplasmic reticulum,cytoplasm,Golgi,vacuole	development,cell organization and biogenesis,transport,metabolic process,response to stimulus	metal ion binding,catalytic activity	Arylsulfatase B
IPI00298883.8	AZI1	X	X		cytoskeleton,membrane,cytoplasm,cytosol	development,cell differentiation,reproduction	motor activity,catalytic activity	Isoform 1 of 5-azacytidine-induced protein 1
IPI00006979.1	LYRM4	X	X		mitochondrion,cytoplasm,nucleus			LYR motif-containing protein 4
IPI00027493.1	SLC3A2	X	X	X		metabolic process	catalytic activity	Isoform 2 of 4F2 cell-surface antigen heavy chain
IPI00095891.2	GNAS	X	X		membrane,cytoplasm,cytosol	development,metabolic process,regulation of biological process,response to stimulus,cell communication,cell differentiation	protein binding,signal transducer activity,metal ion binding,nucleotide binding,catalytic activity	Isoform XLas-1 of Guanine nucleotide-binding protein G(s) subunit alpha isoforms XLas
IPI00852725.1	PRLR			X			protein binding	Isoform 7 of Prolactin receptor
IPI00187164.11	ABLIM2	X	X			cell organization and biogenesis	protein binding,metal ion binding	Isoform 3 of Actin-binding LIM protein 2
IPI00299024.9	BASP1	X	X	X	cytoskeleton,membrane,cytoplasm,organelle lumen,nucleus	cell organization and biogenesis,development,metabolic process,regulation of biological process,reproduction,cell differentiation	protein binding,DNA binding	Isoform 1 of Brain acid soluble protein 1
IPI00001610.1	IGF1			X	extracellular		protein binding	Isoform 2 of Insulin-like growth factor I

IPI	GENE	Alzheimer's Hippocampus	Control hippocampus	CSF	Cellular localization	Biological process	Molecular function	Protein Description
IPI00024662.1	CBX5	X	X	X	cytoplasm,organelle lumen,chromosome,nucleus	metabolic process,regulation of biological process,response to stimulus,coagulation	protein binding	Chromobox protein homolog 5
IPI00024145.2	VDAC2	X	X		membrane,mitochondrion,cytoplasm	transport,regulation of biological process	transporter activity	Isoform 2 of Voltage-dependent anion-selective channel protein 2
IPI00216475.3	MBP	X	X				structural molecule activity	Isoform 3 of Myelin basic protein
IPI00009844.1	GMPR2	X	X		cytoplasm,cytosol	metabolic process	metal ion binding,catalytic activity	Isoform 1 of GMP reductase 2
IPI00028383.3	FAM173A			X	membrane			Protein FAM173A
IPI00032876.1	CYTL1			X	extracellular	development,metabolic process,regulation of biological process,response to stimulus,cell communication,cell differentiation	protein binding	Cytokine-like protein 1
IPI00973315.1	CYAT1			X			protein binding	IGL@ protein
IPI00423461.3	IGHA2			X	membrane		protein binding	Putative uncharacterized protein DKFZp686C02220 (Fragment)
IPI00020199.1	ST8SIA2			X	membrane,cytoplasm,Golgi	development,cell organization and biogenesis,metabolic process,response to stimulus,cell differentiation	catalytic activity	Alpha-2,8-sialyltransferase 8B
IPI00022883.1	AP3M2	X	X		membrane,cytoplasm,Golgi	transport	protein binding	AP-3 complex subunit mu-2
IPI00015102.2	ALCAM	X	X	X	cell surface,membrane	cell organization and biogenesis,development,regulation of biological process,response to stimulus,cell communication,cell differentiation	protein binding	Isoform 1 of CD166 antigen
IPI00218646.3	CYBB		X		mitochondrion,membrane,cytoplasm	metabolic process,transport,regulation of biological process,response to stimulus,defense response	protein binding,transporter activity,metal ion binding,catalytic activity	Cytochrome b-245 heavy chain
IPI00015140.2	CYTB		X		mitochondrion,membrane,cytoplasm	metabolic process,transport	metal ion binding,catalytic activity	Cytochrome b
IPI00014964.5	LY6H	X	X	X	membrane	development		lymphocyte antigen 6H isoform b
IPI00009532.5	ABAT	X	X	X		metabolic process	catalytic activity	cDNA FLJ56034, highly similar to 4-aminobutyrate aminotransferase, mitochondrial
IPI00291867.4	CFI			X	extracellular,membrane	metabolic process,regulation of biological process,response to stimulus,defense response	protein binding,receptor activity,catalytic activity	Complement factor I
IPI00294962.4	SH3KBP1		X		cytoskeleton,membrane,cytoplasm,organelle lumen,nucleus,cytosol	cell death,cell organization and biogenesis,metabolic process,transport,regulation of biological process,response to stimulus,cellular component movement,cell communication	protein binding,catalytic activity	Isoform 1 of SH3 domain-containing kinase-binding protein 1
IPI00025344.1	NDUFS6	X	X		membrane,mitochondrion,cytoplasm	transport,metabolic process	catalytic activity	NADH dehydrogenase [ubiquinone] iron-sulfur protein 6, mitochondrial
IPI00019209.2	SEMA3C			X	membrane	development	protein binding,receptor activity	cDNA FLJ55486, highly similar to Semaphorin-3C
IPI00216337.1	GGA1	X	X		membrane,cytoplasm	transport		Isoform 2 of ADP-ribosylation factor-binding protein GGA1
IPI00163207.1	PGLYRP2			X	extracellular,membrane	metabolic process,response to stimulus,defense response	metal ion binding,receptor activity,catalytic activity	Isoform 1 of N-acetylmuramoyl-L-alanine amidase
IPI00473033.3	ZNF69			X	nucleus	metabolic process,regulation of biological process	DNA binding,metal ion binding	Isoform 1 of Zinc finger protein 69
IPI00005160.3	ARPC1B	X	X		cytoskeleton,cytoplasm	cell organization and biogenesis,regulation of biological process,cellular component movement	protein binding,structural molecule activity	Actin-related protein 2/3 complex subunit 1B
IPI00020632.4	ASS1	X	X		cytoplasm	metabolic process	nucleotide binding,catalytic activity	Argininosuccinate synthase
IPI00007193.7	ANKRD26			X	cytoskeleton,membrane,cytoplasm	cell organization and biogenesis	protein binding,motor activity,catalytic activity	Isoform 2 of Ankyrin repeat domain-containing protein 26
IPI00641251.1	CD320			X	membrane,endoplasmic reticulum,cytoplasm	cell organization and biogenesis,regulation of biological process,cell growth	protein binding	CD320 antigen
IPI00289819.5	IGF2R			X	cell surface,membrane,cytoplasm,Golgi,vacuole,nucleus,endosome	transport,regulation of biological process,response to stimulus,cell communication	signal transducer activity,protein binding,transporter activity,receptor activity,catalytic activity	Cation-independent mannose-6-phosphate receptor
IPI00017469.1	SPR	X	X		cytoplasm,organelle lumen,cytosol,nucleus	metabolic process,regulation of biological process	nucleotide binding,catalytic activity	Sepiapterin reductase
IPI00301459.2	PLA2G15			X	extracellular,mitochondrion,cytoplasm,vacuole	metabolic process	catalytic activity	Group XV phospholipase A2
IPI00028553.1	MINPP1			X			catalytic activity	Isoform 2 of Multiple inositol polyphosphate phosphatase 1
IPI00008894.2	CPA4			X	extracellular	cell organization and biogenesis,metabolic process	metal ion binding,catalytic activity	Carboxypeptidase A4

IPI	GENE	Alzheimer's Hippocampus	Control hippocampus	CSF	Cellular localization	Biological process	Molecular function	Protein Description
IPI00385034.6	SLC9A3R2	X			membrane,cytoplasm,nucleus	development,cell organization and biogenesis,regulation of biological process,response to stimulus,cell communication,cellular homeostasis	protein binding	Isoform 1 of Na(+)/H(+) exchange regulatory cofactor NHE-RF2
IPI00005600.1	NDST2			X	membrane,cytoplasm,Golgi	metabolic process,regulation of biological process	catalytic activity	Isoform 1 of Bifunctional heparan sulfate N-deacetylase/N-sulfotransferase 2
IPI00168921.4	WBSCR17			X	membrane,cytoplasm,Golgi	metabolic process	catalytic activity	Putative polypeptide N-acetylgalactosaminyltransferase-like protein 3
IPI00420096.4	PLEC	X			cytoskeleton		protein binding	Isoform 8 of Plectin
IPI00375547.7	PTPRD			X		metabolic process	protein binding,receptor activity,catalytic activity	213 kDa protein
IPI00465377.2	MXRA7			X	membrane			Isoform 1 of Matrix-remodeling-associated protein 7
IPI00000811.2	PSMB6		X	X	cytoplasm,proteasome,organelle lumen,nucleus,cytosol	cell death,metabolic process,regulation of biological process,response to stimulus,cell communication	catalytic activity	Proteasome subunit beta type-6
IPI00011932.8	HSPA12A	X	X				nucleotide binding	Heat shock 70 kDa protein 12A
IPI00550677.4	WSCD1			X	membrane		catalytic activity	WSC domain-containing protein 1
IPI00303894.7	FAM3A			X	extracellular			Protein FAM3A
IPI00290328.3	PTPRJ			X		metabolic process	protein binding,catalytic activity	Receptor-type tyrosine-protein phosphatase eta precursor
IPI00454695.4	HIST2H2BC			X	chromosome,nucleus	cell organization and biogenesis,metabolic process	DNA binding	Putative histone H2B type 2-C
IPI00029019.7	UBAP2L	X	X					Isoform 2 of Ubiquitin-associated protein 2-like
IPI00025329.1	RPL19	X	X		cytoplasm,ribosome,cytosol	cell organization and biogenesis,metabolic process,transport,reproduction	RNA binding,structural molecule activity	60S ribosomal protein L19
IPI00220059.5	NDUFB4	X	X		mitochondrion,membrane,cytoplasm	transport,metabolic process,response to stimulus	catalytic activity	NADH dehydrogenase [ubiquinone] 1 beta subcomplex subunit 4
IPI00295542.5	NUCB1	X	X	X	extracellular,cytoskeleton,membrane,cytoplasm,Golgi		DNA binding,metal ion binding	Nucleobindin-1
IPI00783987.2	C3	X	X	X	extracellular,membrane	cell organization and biogenesis,development,transport,metabolic process,regulation of biological process,response to stimulus,cell communication,defense response	protein binding,enzyme regulator activity	Complement C3 (Fragment)
IPI00418238.1	AGFG2	X				metabolic process,regulation of biological process	metal ion binding,enzyme regulator activity	Isoform 1 of Arf-GAP domain and FG repeats-containing protein 2
IPI00029061.3	SEPP1			X				Selenoprotein P
IPI00295503.2	DDX58			X			DNA binding,nucleotide binding,catalytic activity	Isoform 2 of Probable ATP-dependent RNA helicase DDX58
IPI00745313.1	AEBP1			X	extracellular,cytoplasm,nucleus	development,metabolic process,regulation of biological process	protein binding,DNA binding,metal ion binding,catalytic activity	Isoform 1 of Adipocyte enhancer-binding protein 1
IPI00335581.5	UBR3		X		membrane	development,metabolic process,response to stimulus	metal ion binding,catalytic activity	Isoform 1 of E3 ubiquitin-protein ligase UBR3
IPI00328328.3	EIF4A2	X	X		cytoplasm,cytosol	metabolic process,regulation of biological process,response to stimulus,cell communication	protein binding,RNA binding,nucleotide binding,catalytic activity	Isoform 1 of Eukaryotic initiation factor 4A-II
IPI00479186.7	PKM	X	X		membrane,cytoplasm,cytosol,nucleus	cell death,metabolic process	protein binding,metal ion binding,nucleotide binding,catalytic activity	Isoform M2 of Pyruvate kinase isozymes M1/M2
IPI00927720.3	FBLN2			X	extracellular,membrane		protein binding,metal ion binding	cDNA FLJ56912, highly similar to Fibulin-2
IPI00306516.1	TIMM44		X		membrane,mitochondrion,cytoplasm,organelle lumen	cell organization and biogenesis,metabolic process,transport	protein binding,transporter activity,nucleotide binding	Mitochondrial import inner membrane translocase subunit TIM44
IPI00218667.2	STMN2			X	membrane,cytoplasm,Golgi,endosome	development,cell organization and biogenesis,regulation of biological process,response to stimulus,cell communication,cell differentiation	protein binding	Stathmin-2
IPI00103064.4	EXOC7		X		cytoskeleton,membrane,cytoplasm,cytosol	transport	protein binding	Isoform 3 of Exocyst complex component 7
IPI00827557.1	MACROD2		X			metabolic process	catalytic activity	Isoform 1 of MACRO domain-containing protein 2
IPI00004397.2	RALB	X	X		membrane	metabolic process,regulation of biological process,response to stimulus,cell communication	nucleotide binding,catalytic activity	cDNA FLJ55177, highly similar to Ras-related protein Ral-B
IPI00942498.1	H3F3AP6	X			chromosome,nucleus	cell organization and biogenesis,metabolic process	DNA binding	H3L-like histone
IPI00642971.3	EEF1D	X	X		cytoplasm	metabolic process	RNA binding	Isoform 2 of Elongation factor 1-delta
IPI00025476.1	AMY2A			X	extracellular	metabolic process	metal ion binding,catalytic activity	Pancreatic alpha-amylase

IPI	GENE	Alzheimer's Hippocampus	Control hippocampus	CSF	Cellular localization	Biological process	Molecular function	Protein Description
IPI00337641.6	TTC7B		X				protein binding	Isoform 1 of Tetratricopeptide repeat protein 7B
IPI00000660.2	TUB	X	X		extracellular,membrane,cytoplasm,nucleus	cell organization and biogenesis,transport,regulation of biological process,response to stimulus		Isoform 1 of Tubby protein homolog
IPI00478003.2	A2M			X	extracellular		enzyme regulator activity	alpha-2-macroglobulin precursor
IPI00477231.3	MGEA5	X	X			metabolic process	catalytic activity	Bifunctional protein NCOAT
IPI00414717.1	GLG1	X	X	X	membrane			Isoform 2 of Golgi apparatus protein 1
IPI00071826.3	FAM54B		X		cytoplasm			cDNA FLJ55391
IPI00167222.4	FAM123A	X	X					Isoform 2 of Protein FAM123A
IPI00007676.3	HSD17B12	X	X		membrane,endoplasmic reticulum,cytoplasm	cell organization and biogenesis,metabolic process,regulation of biological process	protein binding,nucleotide binding,catalytic activity	Estradiol 17-beta-dehydrogenase 12
IPI00299301.1	SYNM	X			cytoskeleton,membrane,cytoplasm	cell organization and biogenesis	protein binding,structural molecule activity	Isoform 1 of Synemin
IPI00033036.1	METAP2	X	X		cytoplasm	metabolic process	metal ion binding,catalytic activity	Methionine aminopeptidase 2
IPI00000873.3	VARS	X	X		mitochondrion,cytoplasm,cytosol	metabolic process,regulation of biological process	protein binding,nucleotide binding,catalytic activity	Valyl-tRNA synthetase
IPI00033541.1	A4GALT			X	membrane,cytoplasm,Golgi	cell organization and biogenesis,metabolic process	catalytic activity	Lactosylceramide 4-alpha-galactosyltransferase
IPI00216774.1	CBLN2			X	extracellular,membrane	development,cell organization and biogenesis,regulation of biological process,cell communication	protein binding	Cerebellin-2
IPI00022305.4	BZW2		X			development,metabolic process,cell differentiation	protein binding	Basic leucine zipper and W2 domain-containing protein 2
IPI00604590.3	NME1-NME2	X	X		cytoplasm	metabolic process	nucleotide binding,catalytic activity	Nucleoside diphosphate kinase
IPI00023322.2	DPF2			X			protein binding,metal ion binding	46 kDa protein
IPI00908788.1	TNC	X	X				protein binding	cDNA FLJ60213, highly similar to Tenascin
IPI00178727.5	WDR96			X				Novel protein
IPI00291395.2	FLRT1			X			protein binding	leucine-rich repeat transmembrane protein FLRT1
IPI00240793.1	ATP11B			X	membrane,endoplasmic reticulum,cytoplasm,nucleus,endosome	cell organization and biogenesis,metabolic process,transport	protein binding,transporter activity,metal ion binding,nucleotide binding,catalytic activity	Probable phospholipid-transporting ATPase IF
IPI00871951.1	CCDC6	X	X		cytoskeleton,cytoplasm		protein binding,structural molecule activity	coiled-coil domain-containing protein 6
IPI00007512.3	GSTO2			X	cytoplasm,cytosol	metabolic process,regulation of biological process,response to stimulus,cellular homeostasis	antioxidant activity,protein binding,catalytic activity	Glutathione S-transferase omega-2
IPI00719622.1	RPS28	X	X		cytoplasm,ribosome,cytosol	cell organization and biogenesis,transport,metabolic process,reproduction	structural molecule activity	40S ribosomal protein S28
IPI00328835.1	STK32C	X	X			metabolic process	metal ion binding,nucleotide binding,catalytic activity	Isoform 1 of Serine/threonine-protein kinase 32C
IPI00003802.2	MAN2A1			X	membrane,cytoplasm,Golgi	development,cell organization and biogenesis,metabolic process	metal ion binding,catalytic activity	Alpha-mannosidase 2
IPI00301255.2	IGSF21	X	X	X	extracellular		protein binding	Immunoglobulin superfamily member 21
IPI00396025.8	SNX32		X			transport,cell communication	protein binding	Isoform 1 of Sorting nexin-32
IPI00254162.2	PTBP2	X			nucleus	metabolic process	RNA binding,nucleotide binding	cDNA FLJ55936, highly similar to Polypyrimidine tract-binding protein 2
IPI00028601.1	MT1P3			X				Similar to Metallothionein-1E
IPI00027806.1	CRISPLD1			X	extracellular			Cysteine-rich secretory protein LCCL domain-containing 1
IPI00007853.2	IFI30			X	extracellular,membrane,cytoplasm,organelle lumen,vacuole	cell proliferation,metabolic process,regulation of biological process,response to stimulus,defense response,cell communication	catalytic activity	Gamma-interferon-inducible lysosomal thiol reductase
IPI00007682.2	ATP6V1A	X	X	X	membrane,mitochondrion,cytoplasm,cytosol	transport,metabolic process,regulation of biological process,response to stimulus,cell communication,cellular homeostasis	transporter activity,nucleotide binding,catalytic activity	V-type proton ATPase catalytic subunit A
IPI00241562.7	RELN			X				Isoform 2 of Reelin
IPI00061623.1	SGTB	X	X		membrane,mitochondrion,cytoplasm	cell organization and biogenesis,transport	protein binding	Small glutamine-rich tetratricopeptide repeat-containing protein beta

IPI	GENE	Alzheimer's Hippocampus	Control hippocampus	CSF	Cellular localization	Biological process	Molecular function	Protein Description
IPI00745122.1	MAP2K4			X				Conserved hypothetical protein
IPI00029739.5	CFH			X	extracellular	metabolic process,regulation of biological process,response to stimulus,defense response		Isoform 1 of Complement factor H
IPI00744366.1	NCAM2			X	extracellular			Similar to DEAH-box RNA helicase
IPI00217345.1	B3GNT2			X	membrane	metabolic process	catalytic activity	Isoform 2 of UDP-GlcNAc:betaGal beta-1,3-N-acetylglucosaminyltransferase 2
IPI00002521.1	ATP5J	X	X		membrane,mitochondrion,cytoplasm	transport,metabolic process	transporter activity,catalytic activity	ATP synthase-coupling factor 6, mitochondrial
IPI00383164.1	IGHA1	X	X		membrane		protein binding	SNC66 protein
IPI00009305.1	GNPDA1	X	X		cytoplasm	metabolic process,reproduction	catalytic activity	Glucosamine-6-phosphate isomerase 1
IPI00553092.2	IGLV7-46			X				V3-3 protein
IPI00010320.1	CBX1	X			cytoskeleton,organelle lumen,chromosome,nucleus	metabolic process,regulation of biological process	protein binding	chromobox protein homolog 1
IPI00942117.2	CRISP3			X				cysteine-rich secretory protein 3 isoform 1 precursor
IPI00386271.4	SLC25A12	X	X		mitochondrion,membrane,cytoplasm	metabolic process,transport,response to stimulus	transporter activity,metal ion binding	Calcium-binding mitochondrial carrier protein Aralar1
IPI00022417.4	LRG1			X	extracellular,membrane	cell differentiation	protein binding	Leucine-rich alpha-2-glycoprotein
IPI01025882.1	IGKV3-20			X			protein binding	Myosin-reactive immunoglobulin light chain variable region (Fragment)
IPI00218539.4	COL11A1			X	extracellular		structural molecule activity	Isoform B of Collagen alpha-1(XI) chain
IPI00179804.2	RBP7	X			cytoplasm	transport	transporter activity	Retinoid-binding protein 7
IPI00019904.2	ADD2	X	X	X	cytoskeleton,membrane,cytoplasm	cell organization and biogenesis,development,transport,regulation of biological process	protein binding,metal ion binding,structural molecule activity	Isoform 1 of Beta-adducin
IPI00000663.1	MLYCD	X	X		mitochondrion,cytoplasm,cytosol	metabolic process,regulation of biological process	protein binding,catalytic activity	Isoform Mitochondrial of Malonyl-CoA decarboxylase, mitochondrial
IPI00376087.3	MACC1			X	cytoplasm,nucleus	cell division,metabolic process,regulation of biological process	protein binding	Metastasis-associated in colon cancer protein 1
IPI00167137.4	SLAMF7			X				Isoform 3 of SLAM family member 7
IPI00887739.3	LOC100133511			X	extracellular		enzyme regulator activity	complement C3-like, partial
IPI00298237.8	TPP1	X	X	X		metabolic process	catalytic activity	cDNA FLJ56402, highly similar to Tripeptidyl-peptidase 1
IPI00060546.3	C10orf35		X	X	membrane			Uncharacterized protein C10orf35
IPI00065475.6	C16orf13	X	X					Isoform 2 of UPF0585 protein C16orf13
IPI00008530.1	RPLP0	X	X		cytoplasm,ribosome,cytosol,nucleus	cell organization and biogenesis,metabolic process,transport,reproduction	protein binding,RNA binding,structural molecule activity	60S acidic ribosomal protein P0
IPI00221354.1	FUS	X	X				metal ion binding	Isoform Short of RNA-binding protein FUS
IPI00794221.1	DBN1	X	X				protein binding	Uncharacterized protein
IPI00401773.5	VPS37C	X	X		cytoskeleton,membrane,cytoplasm,endosome	cell organization and biogenesis,transport,reproduction	protein binding,motor activity,catalytic activity	Vacuolar protein sorting-associated protein 37C
IPI00171844.3	COPS4	X	X		cytoplasm,nucleus	metabolic process	protein binding	COP9 signalosome complex subunit 4
IPI00026781.3	FASN	X	X		mitochondrion,membrane,cytoplasm,Golgi,cytosol	metabolic process,regulation of biological process	protein binding,metal ion binding,nucleotide binding,catalytic activity	Fatty acid synthase
IPI00031411.4	FAT1			X	cell surface,membrane		protein binding,metal ion binding	506 kDa protein
IPI00746177.3	LOC339505			X	cytoskeleton	cell organization and biogenesis,metabolic process,cellular component movement	nucleotide binding,structural molecule activity,catalytic activity	Similar to Tubulin alpha-8 chain
IPI00006094.4	RIMS3			X		transport	protein binding	Regulating synaptic membrane exocytosis protein 3
IPI00220416.3	UQCRB	X	X		membrane,mitochondrion,cytoplasm	transport,metabolic process	transporter activity,catalytic activity	Cytochrome b-c1 complex subunit 7
IPI00220827.5	TMSB10	X	X	X	cytoskeleton,cytoplasm	cell organization and biogenesis,regulation of biological process	protein binding	Thymosin beta-10
IPI00644674.1	NUBP2		X		cytoskeleton,cytoplasm,nucleus	metabolic process	protein binding,metal ion binding,nucleotide binding,catalytic activity	Cytosolic Fe-S cluster assembly factor NUBP2

IPI	GENE	Alzheimer's Hippocampus	Control hippocampus	CSF	Cellular localization	Biological process	Molecular function	Protein Description
IPI00019171.1	SH3GL2	X	X		membrane,cytoplasm,Golgi,cytosol	development,cell organization and biogenesis,metabolic process,transport,regulation of biological process,response to stimulus,cell communication,cell differentiation	protein binding	Endophilin-A1
IPI00872350.2	GALC	X	X		mitochondrion,cytoplasm,organelle lumen,vacuole	metabolic process	catalytic activity	Isoform 1 of Galactocerebrosidase
IPI00000296.2	BRK1		X		cytoskeleton,cytoplasm	cell proliferation,development,cell organization and biogenesis,regulation of biological process,cellular component movement		protein BRICK1
IPI00216697.3	ANK1	X	X	X	cytoskeleton,membrane,endoplasmic reticulum,cytoplasm,cytosol	cell organization and biogenesis,development,transport,regulation of biological process,response to stimulus,cell communication,cell differentiation	protein binding,structural molecule activity	Isoform Er1 of Ankyrin-1
IPI00305827.4	FSD1		X		cytoskeleton,cytoplasm,nucleus	cell organization and biogenesis,cell division	protein binding	Fibronectin type III and SPRY domain-containing protein 1
IPI00418531.4	GLDN			X	extracellular,membrane	development,cell differentiation	protein binding	Isoform 1 of Gliomedin
IPI00169426.5	NT5C1B			X	cytoplasm	metabolic process	metal ion binding,nucleotide binding,catalytic activity	Isoform 2 of Cytosolic 5'-nucleotidase 1B
IPI00298347.1	PTPN11	X	X			metabolic process	protein binding,catalytic activity	Isoform 2 of Tyrosine-protein phosphatase non-receptor type 11
IPI00175019.6	CCDC168			X	membrane			similar to Temporarily Assigned Gene name family member
IPI00554742.3	API5	X	X					Isoform 2 of Apoptosis inhibitor 5
IPI00015980.5	MPDZ			X	membrane		protein binding	Uncharacterized protein
IPI00179330.6	RPS27A	X	X	X	membrane,cytoplasm,ribosome,organelle lumen,nucleus,cytosol,endosome	cell death,cell organization and biogenesis,metabolic process,transport,regulation of biological process,response to stimulus,defense response,cell communication,reproduction	protein binding,metal ion binding,structural molecule activity	Ubiquitin-40S ribosomal protein S27a
IPI00020416.8	TPP2	X	X		cytoplasm,cytosol,nucleus	metabolic process	catalytic activity	Tripeptidyl-peptidase 2
IPI00293530.2	C3AR1			X	membrane	development,regulation of biological process,response to stimulus,cellular component movement,defense response,cell communication,cellular homeostasis	signal transducer activity,protein binding,receptor activity,catalytic activity	C3a anaphylatoxin chemotactic receptor
IPI00792229.1	TATDN1			X			catalytic activity	20 kDa protein
IPI00296441.5	ADA	X	X	X	extracellular,cell surface,membrane,cytoplasm,vacuole,organelle lumen,cytosol	cell death,cell proliferation,development,transport,metabolic process,regulation of biological process,response to stimulus,cellular component movement,cell communication,defense response,reproduction,cell differentiation	protein binding,metal ion binding,catalytic activity	Adenosine deaminase
IPI00011916.1	AIMP2		X		cytoplasm,cytosol,nucleus	cell death,cell proliferation,development,metabolic process,regulation of biological process,cell differentiation	protein binding	Aminoacyl tRNA synthase complex-interacting multifunctional protein 2
IPI00016372.1	RAB9A	X	X		membrane,endoplasmic reticulum,cytoplasm,Golgi,vacuole,nucleus,endosome	metabolic process,transport,regulation of biological process,response to stimulus,cell communication	protein binding,nucleotide binding,catalytic activity	Ras-related protein Rab-9A
IPI00302181.4	CACNA2D3	X	X	X	membrane	transport,regulation of biological process	protein binding,transporter activity,metal ion binding	Isoform 1 of Voltage-dependent calcium channel subunit alpha-2/delta-3
IPI00018451.6	CIB1		X		membrane,endoplasmic reticulum,cytoplasm,organelle lumen,nucleus	cell death,cell proliferation,metabolic process,regulation of biological process,response to stimulus	protein binding,metal ion binding	Calcium and integrin-binding protein 1
IPI00019930.1	UBE2D1		X		cytoplasm,organelle lumen,cytosol,nucleus	cell organization and biogenesis,metabolic process,regulation of biological process,response to stimulus,cell communication	protein binding,nucleotide binding,catalytic activity	Ubiquitin-conjugating enzyme E2 D1
IPI00413500.4	COMMD4	X	X		cytoplasm		protein binding	Isoform 1 of COMM domain-containing protein 4
IPI00306046.1	EDIL3			X	extracellular	development,regulation of biological process	protein binding,metal ion binding	Isoform 1 of EGF-like repeat and discoidin I-like domain-containing protein 3
IPI00746666.6	BET1L			X			protein binding	Uncharacterized protein
IPI00217537.3	ASXL1			X	organelle lumen,chromosome,nucleus	cell organization and biogenesis,metabolic process,regulation of biological process,response to stimulus,cell communication,cell differentiation	protein binding,metal ion binding	Isoform 1 of Putative Polycomb group protein ASXL1
IPI00028912.2	VEZF1			X	organelle lumen,nucleus	development,metabolic process,regulation of biological process,response to stimulus,defense response,cell differentiation	DNA binding,metal ion binding	Vascular endothelial zinc finger 1
IPI00020701.5	ERI3	X	X			metabolic process	metal ion binding,catalytic activity	Isoform 1 of ERI1 exoribonuclease 3

IPI	GENE	Alzheimer's Hippocampus	Control hippocampus	CSF	Cellular localization	Biological process	Molecular function	Protein Description
IPI00414640.1	ISYNA1	X	X			metabolic process	nucleotide binding,catalytic activity	58 kDa protein
IPI00004488.2	ATP6V1F	X	X	X	membrane,cytoplasm,vacuole,cytosol	transport,regulation of biological process,response to stimulus,cell communication,cellular homeostasis	protein binding,transporter activity,catalytic activity	V-type proton ATPase subunit F
IPI00103749.2	TREM2			X	membrane			Isoform 2 of Triggering receptor expressed on myeloid cells 2
IPI00425404.3	KIF21A	X	X		cytoskeleton,mitochondrion,membrane,cytoplasm,organelle lumen,chromosome,nucleus,cytosol	cell organization and biogenesis,metabolic process,transport,cellular component movement,reproduction	protein binding,motor activity,nucleotide binding,catalytic activity	Isoform 1 of Kinesin-like protein KIF21A
IPI00871139.1	MASP1			X		metabolic process	metal ion binding,catalytic activity	92 kDa protein
IPI00024035.1	CDH6			X	membrane,cytoplasm,organelle lumen,nucleus	cell organization and biogenesis	metal ion binding	Isoform 1 of Cadherin-6
IPI00001592.1	GPNMB			X	membrane,cytoplasm	development,metabolic process,regulation of biological process,response to stimulus,cell communication,cell differentiation	protein binding	Isoform 2 of Transmembrane glycoprotein NMB
IPI00514285.2	PTGDS			X	extracellular	transport,metabolic process	transporter activity	Uncharacterized protein
IPI00181733.4	FBXL16	X	X				protein binding	F-box/LRR-repeat protein 16
IPI00032262.1	INPP5A		X		membrane	metabolic process,cell communication	protein binding,catalytic activity	Type I inositol-1,4,5-trisphosphate 5-phosphatase
IPI00031907.3	TMEM108			X	membrane			Isoform 1 of Transmembrane protein 108
IPI00295387.5	CRLF3	X	X		cytoplasm	cell organization and biogenesis,metabolic process,regulation of biological process,response to stimulus,cell communication,cell growth	protein binding	Cytokine receptor-like factor 3
IPI00332095.2	BCAS3		X		cytoplasm,nucleus		protein binding	Isoform 2 of Breast carcinoma-amplified sequence 3
IPI00015077.1	EIF1	X	X		cytoplasm	metabolic process,regulation of biological process,response to stimulus	RNA binding	Eukaryotic translation initiation factor 1
IPI00020153.4	BSN	X	X		cytoskeleton,membrane,cytoplasm,organelle lumen,nucleus	cell communication	metal ion binding	Protein bassoon
IPI00010706.1	GSS		X	X	cytoplasm,cytosol	development,metabolic process,response to stimulus	protein binding,metal ion binding,nucleotide binding,catalytic activity	Glutathione synthetase
IPI00290078.5	KRT4			X	cytoskeleton		motor activity,catalytic activity	cDNA FLJ58275, highly similar to Keratin, type II cytoskeletal 4
IPI00163187.10	FSCN1	X	X	X	cytoskeleton,cytoplasm	cell proliferation,cell organization and biogenesis,cellular component movement	protein binding	Fascin
IPI00019399.2	SAA4			X	extracellular	response to stimulus,defense response		Serum amyloid A-4 protein
IPI00004582.3	EML1	X			membrane		protein binding,catalytic activity	Uncharacterized protein
IPI00829812.3	IGHV3-13			X				13 kDa protein
IPI00019038.1	LYZ			X	extracellular	cell death,metabolic process,response to stimulus,defense response	catalytic activity	Lysozyme C
IPI00021466.5	PAIP1	X	X		cytoplasm,cytosol	metabolic process,regulation of biological process	protein binding,DNA binding,RNA binding,translation regulator activity	Isoform 1 of Polyadenylate-binding protein-interacting protein 1
IPI00443909.1	CNPY2	X	X	X	membrane,endoplasmic reticulum,cytoplasm	metabolic process,regulation of biological process	protein binding	Isoform 1 of Protein canopy homolog 2
IPI00010338.1	F3	X	X		extracellular,cell surface,membrane,cytoplasm	cell death,cell proliferation,development,metabolic process,regulation of biological process,response to stimulus,cellular component movement,cell communication,defense response,coagulation	protein binding	Tissue factor
IPI00410214.1	BPNT1	X	X		cytoplasm,cytosol	development,metabolic process,response to stimulus	metal ion binding,catalytic activity	Isoform 1 of 3'(2'),5'-bisphosphate nucleotidase 1
IPI00878436.1	SLC2A11			X	membrane	transport	transporter activity	Uncharacterized protein
IPI00219365.3	MSN	X	X	X	cell surface,cytoskeleton,membrane,cytoplasm,organelle lumen,nucleus	regulation of biological process,cellular component movement	protein binding,structural molecule activity	Moesin
IPI00434711.1	FLJ38576			X				Putative uncharacterized protein FP6679
IPI00000914.2	CALCA			X	extracellular,cytoplasm,nucleus	development,metabolic process,regulation of biological process,response to stimulus,cellular component movement,defense response,cell communication,cellular homeostasis,reproduction	protein binding	Isoform 1 of Calcitonin
IPI00329495.4	ABLIM1	X	X		cytoskeleton,cytoplasm	cell organization and biogenesis,development,metabolic process,regulation of biological process,response to stimulus,cell differentiation	protein binding,metal ion binding	Isoform 1 of Actin-binding LIM protein 1

IPI	GENE	Alzheimer's Hippocampus	Control hippocampus	CSF	Cellular localization	Biological process	Molecular function	Protein Description
IPI00216682.5	CNN3	X	X			cell organization and biogenesis	protein binding	Calponin-3
IPI00027462.1	S100A9			X	extracellular,cytoskeleton,membrane,cytoplasm,organelle lumen,nucleus	cell organization and biogenesis,metabolic process,regulation of biological process,response to stimulus,cellular component movement,defense response,cell communication	protein binding,signal transducer activity,metal ion binding	Protein S100-A9
IPI00844156.2	SERPINC1			X			enzyme regulator activity	Uncharacterized protein
IPI00002538.1	PDPK1		X		membrane,cytoplasm,organelle lumen,cytosol,nucleus	cell death,development,cell organization and biogenesis,transport,metabolic process,regulation of biological process,response to stimulus,cellular component movement,cell communication,cell differentiation,coagulation	protein binding,nucleotide binding,catalytic activity	Isoform 1 of 3-phosphoinositide-dependent protein kinase 1
IPI00328703.1	OAF			X				Out at first protein homolog
IPI00012079.12	EIF4B	X	X		cytoplasm,cytosol	metabolic process,regulation of biological process,response to stimulus,cell communication	protein binding,RNA binding,nucleotide binding	Eukaryotic translation initiation factor 4B
IPI00031547.2	DSG3			X	membrane,cytoplasm,cytosol	cell death,cell organization and biogenesis	metal ion binding	Desmoglein-3
IPI00854806.1	IGKV1-5			X			protein binding	IGKV1-5 protein
IPI00024821.1	PSMD14	X	X		proteasome,cytoplasm,organelle lumen,nucleus,cytosol	cell death,metabolic process,regulation of biological process,response to stimulus,cell communication	protein binding,metal ion binding,catalytic activity,enzyme regulator activity	26S proteasome non-ATPase regulatory subunit 14
IPI00028031.2	ACADVL	X	X			metabolic process	catalytic activity	cDNA FLJ56425, highly similar to Very-long-chain specific acyl-CoAdehydrogenase, mitochondrial
IPI00100980.9	EHD2		X	X	membrane,cytoplasm,nucleus,endosome	cell organization and biogenesis,metabolic process,transport,response to stimulus,coagulation	protein binding,metal ion binding,nucleotide binding,catalytic activity	EH domain-containing protein 2
IPI00029817.1	NEU1			X	membrane,cytoplasm,vacuole,organelle lumen	metabolic process	catalytic activity	Sialidase-1
IPI00299299.3	HSPA13			X	membrane,endoplasmic reticulum,cytoplasm	cell organization and biogenesis	nucleotide binding	Heat shock 70 kDa protein 13
IPI00032561.1	CAB39	X	X		cytoplasm,cytosol	cell organization and biogenesis,metabolic process,regulation of biological process,response to stimulus,cell communication	protein binding,catalytic activity,enzyme regulator activity	Calcium-binding protein 39
IPI00162547.5	LPHN3			X	membrane	regulation of biological process,response to stimulus,cell communication	protein binding,signal transducer activity,receptor activity	Isoform 2 of Latrophilin-3
IPI00550232.2	XIRP2			X		cell organization and biogenesis	protein binding	xin actin-binding repeat-containing protein 2 isoform 1
IPI00783434.1	MAN2C1	X	X			metabolic process	metal ion binding,catalytic activity	Alpha-mannosidase 2C1
IPI00328391.3	GALNT7			X	membrane,cytoplasm,Golgi	metabolic process	catalytic activity	N-acetylgalactosaminyltransferase 7
IPI00294615.3	FBLN5			X	extracellular,cytoplasm	cell organization and biogenesis,metabolic process,regulation of biological process,response to stimulus	protein binding,metal ion binding	Fibulin-5
IPI00218993.1	HSPH1	X	X					Isoform Beta of Heat shock protein 105 kDa
IPI00456969.1	DYNC1H1	X	X	X	cytoskeleton,cytoplasm,Golgi,cytosol	cell death,cell organization and biogenesis,metabolic process,transport,cellular component movement	protein binding,motor activity,nucleotide binding,catalytic activity	Cytoplasmic dynein 1 heavy chain 1
IPI00304865.3	TGFBR3			X	extracellular,cell surface,membrane	cell proliferation,development,cell organization and biogenesis,metabolic process,regulation of biological process,response to stimulus,cellular component movement,cell communication,cell differentiation,cell growth	protein binding,signal transducer activity,receptor activity,catalytic activity	Isoform 1 of Transforming growth factor beta receptor type 3
IPI00472521.5	FAM21B			X				Family with sequence similarity 21, member B
IPI00293095.2	CCDC83			X			structural molecule activity,catalytic activity	Isoform 1 of Coiled-coil domain-containing protein 83
IPI00335946.4	FAM120B			X		metabolic process	catalytic activity	cDNA FLJ56631
IPI00216602.3	FGFR2			X		metabolic process	nucleotide binding,catalytic activity	Isoform 5 of Fibroblast growth factor receptor 2
IPI00555734.3	ASRGL1	X	X		cytoskeleton,cytoplasm,organelle lumen,nucleus	metabolic process	catalytic activity	Isoform 1 of L-asparaginase
IPI00026197.8	IGKV4-1			X			protein binding	Ig kappa chain V-IV region
IPI00410487.3	TWSG1			X	extracellular	development,regulation of biological process,response to stimulus,cell communication,cell differentiation		Isoform 1 of Twisted gastrulation protein homolog 1

IPI	GENE	Alzheimer's Hippocampus	Control hippocampus	CSF	Cellular localization	Biological process	Molecular function	Protein Description
IPI00010863.7	ATOX1	X	X	X	cytoplasm,cytosol	transport,response to stimulus,cellular homeostasis	protein binding,metal ion binding	Copper transport protein ATOX1
IPI00219446.5	PEBP1	X	X	X	extracellular,cell surface,mitochondrion,membrane,endoplasmic reticulum,cytoplasm,Golgi	cell organization and biogenesis,development,metabolic process,regulation of biological process,response to stimulus,cell communication,reproduction,cell differentiation	protein binding,nucleotide binding,enzyme regulator activity	Phosphatidylethanolamine-binding protein 1
IPI00029606.1	ADAM17			X		metabolic process	metal ion binding,catalytic activity	Isoform B of Disintegrin and metalloproteinase domain-containing protein 17
IPI00006009.3	PLEKHB1	X	X	X			protein binding	Isoform 2 of Pleckstrin homology domain-containing family B member 1
IPI00011730.3	EMILIN3			X	extracellular,cytoskeleton,membrane,cytoplasm	cell organization and biogenesis	protein binding	EMILIN-3
IPI00025512.2	HSPB1	X	X		cytoskeleton,cell surface,membrane,cytoplasm,proteasome,nucleus,cytosol	cell death,development,metabolic process,regulation of biological process,response to stimulus,cellular component movement,cell communication	protein binding,enzyme regulator activity	Heat shock protein beta-1
IPI00032050.4	WBP2	X	X	X			protein binding	WW domain-binding protein 2
IPI00217405.1	UBR1			X	cytoplasm,proteasome,cytosol	metabolic process,regulation of biological process,response to stimulus,cell communication	metal ion binding,catalytic activity	Isoform 1 of E3 ubiquitin-protein ligase UBR1
IPI00022810.2	CTSC			X	endoplasmic reticulum,cytoplasm,Golgi,vacuole	cell death,metabolic process,regulation of biological process,response to stimulus	protein binding,catalytic activity	Isoform 1 of Dipeptidyl peptidase 1
IPI00604494.2	CCDC92	X	X		membrane,cytoplasm,Golgi	cell organization and biogenesis	transporter activity	Coiled-coil domain-containing protein 92
IPI00005218.1	MOCS2	X			cytoplasm,cytosol,nucleus	metabolic process	protein binding,catalytic activity	Molybdopterin synthase catalytic subunit
IPI00027175.1	SRI	X	X		membrane,endoplasmic reticulum,cytoplasm	development,transport,regulation of biological process,response to stimulus,cell communication,cellular homeostasis	protein binding,metal ion binding	Sorcin
IPI00008164.2	PREP	X	X	X	cytoplasm,cytosol,nucleus	metabolic process	catalytic activity	Prolyl endopeptidase
IPI00335385.4	DCPS	X			mitochondrion,cytoplasm,organelle lumen,nucleus,cytosol	metabolic process	protein binding,catalytic activity	Scavenger mRNA-decapping enzyme DcpS
IPI00004494.1	SEMA3E			X	extracellular,membrane	development,cell organization and biogenesis,regulation of biological process,response to stimulus,cell communication,cell differentiation	protein binding,receptor activity	Semaphorin-3E
IPI00302944.3	COL12A1			X			protein binding	Isoform 4 of Collagen alpha-1(XII) chain
IPI00017731.1	Sep-05	X	X		cytoskeleton,membrane,cytoplasm	metabolic process,cell division,transport,regulation of biological process,cell communication	protein binding,nucleotide binding,structural molecule activity,catalytic activity	Septin-5
IPI00029343.2	CNTNAP2	X	X	X	extracellular,cell surface,membrane,cytoplasm,Golgi,endosome	cell organization and biogenesis,development,regulation of biological process,response to stimulus,cell communication,cell differentiation	protein binding,structural molecule activity	Isoform 1 of Contactin-associated protein-like 2
IPI00061114.1	RAB3C	X	X		membrane,cytoplasm,cytosol	transport,regulation of biological process,response to stimulus,cell communication	nucleotide binding	Ras-related protein Rab-3C
IPI00007058.1	CORO1B	X	X		cytoskeleton,cytoplasm	cell organization and biogenesis	protein binding	Coronin-1B
IPI00789155.1	CALU	X	X					calumenin isoform c precurosr
IPI00021753.5	KIF13B			X	cytoskeleton,cytoplasm	cell organization and biogenesis,development,transport,regulation of biological process,response to stimulus,cellular component movement,cell communication,cell differentiation	protein binding,nucleotide binding,motor activity,catalytic activity	Kinesin-like protein KIF13B
IPI00018381.1	TLL1			X	extracellular	development,metabolic process,cell differentiation	protein binding,metal ion binding,catalytic activity	Isoform 1 of Tolloid-like protein 1
IPI00647635.3	GIGYF2	X					protein binding	Isoform 2 of PERQ amino acid-rich with GYF domain-containing protein 2
IPI00102291.2	FAM126A	X			membrane,cytoplasm	regulation of biological process,response to stimulus,cell communication	signal transducer activity	Isoform 1 of Hyccin
IPI00884105.2	LAMP1	X		X	cell surface,membrane,cytoplasm,vacuole,endosome	cell death,metabolic process		Lysosome-associated membrane glycoprotein 1
IPI00441867.1	PEX19	X	X		membrane,cytoplasm,nucleus,cytosol	cell organization and biogenesis,metabolic process,transport,regulation of biological process	protein binding	Isoform 1 of Peroxisomal biogenesis factor 19
IPI00029811.5	WDR54		X				protein binding	WD repeat-containing protein 54
IPI00018364.2	RAP2B	X	X		membrane,cytoplasm,cytosol,endosome	metabolic process,transport,regulation of biological process,response to stimulus,cellular component movement,cell communication	protein binding,transporter activity,nucleotide binding,catalytic activity	Ras-related protein Rap-2b

IPI	GENE	Alzheimer's Hippocampus	Control hippocampus	CSF	Cellular localization	Biological process	Molecular function	Protein Description
IPI00101927.2	LZTS2			X	cytoskeleton,membrane,cytoplasm	cell death,cell organization and biogenesis,cell division,regulation of biological process,response to stimulus,cell communication		Leucine zipper putative tumor suppressor 2
IPI00295414.7	COL15A1			X	extracellular,membrane	development,regulation of biological process,response to stimulus,cell communication,cell differentiation	structural molecule activity	Collagen alpha-1(XV) chain
IPI00743284.2	MTR			X	cytoplasm,cytosol	development,metabolic process,response to stimulus	metal ion binding,catalytic activity	Methionine synthase
IPI00031617.3	MNF1		X		mitochondrion,cytoplasm,organelle lumen			Uncharacterized protein C6orf125
IPI00418414.4	COQ3		X		mitochondrion,cytoplasm,organelle lumen	metabolic process	catalytic activity	Hexaprenyldihydroxybenzoate methyltransferase, mitochondrial
IPI00022229.2	APOB	X	X	X	extracellular,membrane,endoplasmic reticulum,cytoplasm,organelle lumen,endosome	cell organization and biogenesis,development,transport,metabolic process,regulation of biological process,response to stimulus,cellular component movement,cell differentiation,reproduction,coagulation	protein binding,transporter activity	Apolipoprotein B-100
IPI00397645.7	MXRA7		X	X				Isoform 2 of Matrix-remodeling-associated protein 7
IPI00006024.5	DOCK4	X	X		membrane		protein binding,nucleotide binding,enzyme regulator activity	Isoform 2 of Dedicator of cytokinesis protein 4
IPI00019888.1	ALDH5A1	X	X	X	mitochondrion,cytoplasm,organelle lumen	cell organization and biogenesis,development,transport,metabolic process,cell communication	protein binding,catalytic activity	Succinate-semialdehyde dehydrogenase, mitochondrial
IPI00470484.2	EGFLAM			X	extracellular	cell organization and biogenesis,metabolic process,regulation of biological process	protein binding	Isoform 1 of Pikachurin
IPI00299147.8	SUMO3			X	cytoplasm,chromosome	metabolic process	protein binding,catalytic activity	Small ubiquitin-related modifier 3
IPI00030532.1	CPNE5		X				protein binding	Copine-5
IPI00152470.1	PROKR1			X	membrane	cell death,regulation of biological process,response to stimulus,cell communication	signal transducer activity,receptor activity	Prokineticin receptor 1
IPI00217012.4	PSD			X	membrane	regulation of biological process,response to stimulus,cell communication	protein binding,enzyme regulator activity	Isoform 1 of PH and SEC7 domain-containing protein 1
IPI00024601.1	CA10			X		development		Carbonic anhydrase-related protein 10
IPI00657699.1	MUC19			X		metabolic process	nucleotide binding,catalytic activity,enzyme regulator activity	Protein
IPI00395510.5	NCALD	X	X		membrane,cytoplasm,Golgi,cytosol	transport,cell communication	protein binding,metal ion binding	Neurocalcin-delta
IPI00029054.1	NT5C2	X	X		cytoplasm,cytosol	metabolic process	protein binding,metal ion binding,nucleotide binding,catalytic activity	Cytosolic purine 5'-nucleotidase
IPI00004461.2	DGUOK	X	X		mitochondrion,cytoplasm,organelle lumen,cytosol	cell organization and biogenesis,development,metabolic process,regulation of biological process,cell differentiation	nucleotide binding,catalytic activity	Isoform 1 of Deoxyguanosine kinase, mitochondrial
IPI00103853.7	RBFA			X	mitochondrion,cytoplasm	metabolic process	catalytic activity	Isoform 1 of Putative ribosome-binding factor A, mitochondrial
IPI00289501.2	VGF	X	X	X	extracellular,membrane,cytoplasm	development,transport,metabolic process,regulation of biological process,response to stimulus,cell communication,reproduction	protein binding	Neurosecretory protein VGF
IPI00011604.2	GCSH	X	X		mitochondrion,cytoplasm	metabolic process	protein binding,catalytic activity	Glycine cleavage system H protein, mitochondrial
IPI00019172.3	SH3GL3	X	X		membrane,cytoplasm,endosome	development,transport,regulation of biological process,response to stimulus,cell communication	protein binding	Isoform 1 of Endophilin-A3
IPI00328867.6	SRC		X			metabolic process	protein binding,nucleotide binding,catalytic activity	Isoform 2 of Proto-oncogene tyrosine-protein kinase Src
IPI00024248.1	SLC5A5			X	membrane,nucleus	transport,metabolic process,response to stimulus	transporter activity	Sodium/iodide cotransporter
IPI00215911.3	APEX1	X	X		cytoskeleton,mitochondrion,endoplasmic reticulum,cytoplasm,ribosome,organelle lumen,nucleus	metabolic process,regulation of biological process,response to stimulus,cellular homeostasis	protein binding,DNA binding,RNA binding,metal ion binding,catalytic activity	DNA-(apurinic or apyrimidinic site) lyase
IPI00915412.1	EPHB6			X	membrane	metabolic process,regulation of biological process,response to stimulus,cell communication	signal transducer activity,protein binding,nucleotide binding,receptor activity,catalytic activity	EPH receptor B6
IPI00015473.3	SLC1A3	X	X		extracellular,cell surface,mitochondrion,membrane,cytoplasm	cell organization and biogenesis,development,metabolic process,transport,regulation of biological process,response to stimulus,cell communication,cell differentiation	transporter activity	Excitatory amino acid transporter 1

IPI	GENE	Alzheimer's Hippocampus	Control hippocampus	CSF	Cellular localization	Biological process	Molecular function	Protein Description
IPI00061448.5	CCDC126			X			catalytic activity	unknown
IPI00941154.3	TPD52	X	X					tumor protein D52 isoform 2
IPI00477526.2	CCZ1	X						Similar to Radial spoke head 10 homolog B
IPI00385128.3	LPCAT4		X		membrane,endoplasmic reticulum,cytoplasm	metabolic process	metal ion binding,catalytic activity	Isoform 1 of Lysophospholipid acyltransferase LPCAT4
IPI00021275.4	EPHB2			X	membrane	development,cell organization and biogenesis,metabolic process,regulation of biological process,response to stimulus,cell communication,cell differentiation	protein binding,signal transducer activity,nucleotide binding,receptor activity,catalytic activity	Isoform 1 of Ephrin type-B receptor 2
IPI00829841.1	IGHV3-73			X				13 kDa protein
IPI00174390.3	PDE12	X	X		mitochondrion,cytoplasm		catalytic activity	Isoform 1 of 2',5'-phosphodiesterase 12
IPI00168165.5	CLASP2	X	X		membrane			Uncharacterized protein
IPI00016912.1	TTC1	X	X		membrane,cytoplasm	metabolic process	protein binding	Tetratricopeptide repeat protein 1
IPI00743576.1	ATP6V0A1	X	X		membrane,cytoplasm,vacuole	transport	transporter activity	Isoform 2 of V-type proton ATPase 116 kDa subunit a isoform 1
IPI00217030.1<br>0	RPS4X	X	X		cytoplasm,ribosome,cytosol	cell proliferation,cell organization and biogenesis,development,metabolic process,transport,regulation of biological process,reproduction	RNA binding,structural molecule activity	40S ribosomal protein S4, X isoform
IPI00397578.3	PPFIA4		X	X	extracellular,mitochondrion,membrane,cytoplasm	cell organization and biogenesis,transport,metabolic process	protein binding	Protein tyrosine phosphatase, receptor type, f polypeptide (PTPRF), interacting protein (Liprin), alpha 4
IPI00025257.1	SEMA7A	X		X	cell surface,membrane	cell organization and biogenesis,development,regulation of biological process,response to stimulus,cell communication,defense response,cell growth,cell differentiation	protein binding,receptor activity	Semaphorin-7A
IPI00472754.3	KIAA0319L			X	membrane,cytoplasm		protein binding	Isoform 1 of Dyslexia-associated protein KIAA0319-like protein
IPI00290854.2	AKAP3			X	cytoplasm	transport,regulation of biological process,response to stimulus,cellular component movement,cell communication,reproduction	protein binding	A-kinase anchor protein 3
IPI00031410.1	MTOR	X	X		membrane,mitochondrion,endoplasmic reticulum,cytoplasm,Golgi,vacuole,organelle lumen,cytosol,nucleus	cell proliferation,cell organization and biogenesis,transport,metabolic process,regulation of biological process,response to stimulus,cell communication,reproduction,cell growth,cell differentiation	protein binding,DNA binding,nucleotide binding,catalytic activity	Serine/threonine-protein kinase mTOR
IPI00154528.3	SMC6			X	cytoskeleton,membrane,cytoplasm,organelle lumen,chromosome,nucleus	cell organization and biogenesis,metabolic process,response to stimulus	protein binding,nucleotide binding	Isoform 1 of Structural maintenance of chromosomes protein 6
IPI00969427.1	IGL@			X			protein binding	IGL@ protein
IPI00029168.1	LPA			X	extracellular	metabolic process,transport,regulation of biological process	protein binding,catalytic activity,enzyme regulator activity	Apolipoprotein(a)
IPI00019812.1	PPP5C	X	X	X	cytoplasm,Golgi,cytosol,nucleus	cell organization and biogenesis,metabolic process,regulation of biological process,response to stimulus,cell communication	protein binding,signal transducer activity,metal ion binding,catalytic activity	Serine/threonine-protein phosphatase 5
IPI00150057.6	SMARCC2		X				protein binding	Isoform 2 of SWI/SNF complex subunit SMARCC2
IPI00440153.2	XRRA1			X				Isoform 2 of X-ray radiation resistance-associated protein 1
IPI00004669.1	GALNT2			X	extracellular,membrane,cytoplasm,Golgi	metabolic process	metal ion binding,catalytic activity	Polypeptide N-acetylgalactosaminyltransferase 2
IPI00022674.2	OSMR			X	membrane	cell proliferation,regulation of biological process,response to stimulus,defense response,cell communication	signal transducer activity,protein binding,receptor activity	Isoform 1 of Oncostatin-M-specific receptor subunit beta
IPI00021885.1	FGA	X	X	X	extracellular,cell surface,membrane,cytoplasm,organelle lumen	cell organization and biogenesis,transport,regulation of biological process,response to stimulus,cell communication,coagulation	protein binding	Isoform 1 of Fibrinogen alpha chain
IPI00297181.2	CDH7			X	membrane	cell organization and biogenesis	metal ion binding	Cadherin-7
IPI00016832.1	PSMA1	X	X		cytoskeleton,cytoplasm,proteasome,organelle lumen,cytosol,nucleus	cell death,metabolic process,regulation of biological process,response to stimulus,cell communication	protein binding,RNA binding,catalytic activity	Isoform Short of Proteasome subunit alpha type-1
IPI00293971.3	ATP1B2	X	X	X	membrane,cytoplasm	metabolic process,transport,response to stimulus,cellular component movement,coagulation	protein binding,transporter activity,catalytic activity	Sodium/potassium-transporting ATPase subunit beta-2
IPI00025110.5	MSLN			X				Isoform 2 of Mesothelin

IPI	GENE	Alzheimer's Hippocampus	Control hippocampus	CSF	Cellular localization	Biological process	Molecular function	Protein Description
IPI00446294.3	TOM1L2	X	X			transport		Isoform 1 of TOM1-like protein 2
IPI00024502.2	UBQLN4			X	membrane,endoplasmic reticulum,proteasome,cytoplasm,organelle lumen,cytosol,nucleus	metabolic process,regulation of biological process	protein binding	Isoform 1 of Ubiquilin-4
IPI00020127.1	RPA1		X		cytoskeleton,cytoplasm,organelle lumen,chromosome,nucleus	cell proliferation,cell organization and biogenesis,development,metabolic process,regulation of biological process,response to stimulus,reproduction	protein binding,DNA binding,metal ion binding	Replication protein A 70 kDa DNA-binding subunit
IPI00027834.3	HNRNPL	X	X	X	cytoplasm,organelle lumen,nucleus	metabolic process	protein binding,DNA binding,RNA binding,nucleotide binding	Heterogeneous nuclear ribonucleoprotein L
IPI00411656.5	PCLO		X	X	cytoskeleton,membrane,cytoplasm	cell organization and biogenesis,transport,cell communication	protein binding,transporter activity,metal ion binding	Isoform 1 of Protein piccolo
IPI00793841.1	BCAS1	X			chromosome	cell organization and biogenesis,metabolic process		53 kDa protein
IPI00740191.2	LOC651986			X		metabolic process,regulation of biological process	DNA binding	similar to Forkhead box protein L1
IPI00218730.6	PDE6A			X	membrane,cytoplasm,cytosol	development,metabolic process,regulation of biological process,response to stimulus,cell communication,cellular homeostasis,coagulation	protein binding,metal ion binding,catalytic activity	Rod cGMP-specific 3',5'-cyclic phosphodiesterase subunit alpha
IPI00011781.3	ANKRD60			X			protein binding	Ankyrin repeat domain-containing protein 60
IPI00746851.2	ITGB2	X	X		membrane	regulation of biological process,response to stimulus,cell communication	protein binding,receptor activity	Uncharacterized protein
IPI00024689.6	AQP1	X	X	X	extracellular,membrane,cytoplasm,nucleus	cell death,cell proliferation,cell organization and biogenesis,development,metabolic process,transport,regulation of biological process,response to stimulus,cell communication,cellular homeostasis	protein binding,transporter activity	Aquaporin-1
IPI00294210.3	ID2			X	cytoplasm,organelle lumen,chromosome,nucleus,cytosol	cell proliferation,development,metabolic process,regulation of biological process,response to stimulus,cell differentiation	protein binding	DNA-binding protein inhibitor ID-2
IPI00216914.2	VMO1			X	extracellular	cell organization and biogenesis,reproduction		Vitelline membrane outer layer protein 1 homolog
IPI00783872.2	CAPRIN1	X	X		membrane,cytoplasm,cytosol	development,cell organization and biogenesis,metabolic process,regulation of biological process,cell differentiation	RNA binding	Isoform 1 of Caprin-1
IPI00394870.3	VWC2			X	extracellular	development,regulation of biological process,response to stimulus,cell communication,cell differentiation	protein binding	Brorin
IPI00642816.2	SRP9P1	X	X		membrane,endoplasmic reticulum,cytoplasm,cytosol	metabolic process,transport,regulation of biological process	RNA binding	Isoform 1 of Signal recognition particle 9 kDa protein
IPI00478586.3	VPS13A		X					Isoform 2 of Vacuolar protein sorting-associated protein 13A
IPI00012009.1	CSF2RA			X	extracellular,membrane	regulation of biological process,response to stimulus,cell communication	protein binding,signal transducer activity,receptor activity	Isoform 1 of Granulocyte-macrophage colony-stimulating factor receptor subunit alpha
IPI00021304.1	KRT2			X				Keratin, type II cytoskeletal 2 epidermal
IPI00257508.4	DPYSL2	X	X	X	membrane,mitochondrion,cytoplasm,cytosol	cell organization and biogenesis,development,transport,metabolic process,regulation of biological process,response to stimulus,cell communication,cell differentiation	protein binding,catalytic activity	Dihydropyrimidinase-related protein 2
IPI00219156.7	RPL30	X	X		cytoplasm,ribosome,cytosol	cell organization and biogenesis,transport,metabolic process,reproduction	RNA binding,structural molecule activity	60S ribosomal protein L30
IPI00465016.7	QSOX1			X		metabolic process,regulation of biological process,cellular homeostasis	catalytic activity	Isoform 2 of Sulfhydryl oxidase 1
IPI00009896.1	EPHX1	X	X		membrane,endoplasmic reticulum,cytoplasm	development,metabolic process,response to stimulus	protein binding,catalytic activity	Epoxide hydrolase 1
IPI00304962.4	COL1A2			X	extracellular	development,cell organization and biogenesis,regulation of biological process,response to stimulus,cellular component movement,cell communication,cell differentiation,coagulation	protein binding,structural molecule activity	Collagen alpha-2(I) chain
IPI00015902.3	PDGFRB			X	membrane,cytoplasm,vacuole,organelle lumen,nucleus	cell proliferation,development,cell organization and biogenesis,metabolic process,transport,regulation of biological process,response to stimulus,cellular component movement,cell communication,cell differentiation	protein binding,signal transducer activity,nucleotide binding,receptor activity,catalytic activity	Beta-type platelet-derived growth factor receptor
IPI00745232.1	GNB5	X	X				protein binding	Isoform 2 of Guanine nucleotide-binding protein subunit beta-5
IPI00026089.4	SF3B1		X		spliceosomal complex,organelle lumen,nucleus	metabolic process,regulation of biological process,response to stimulus,cell communication,reproduction	signal transducer activity,DNA binding,receptor activity	Splicing factor 3B subunit 1

IPI	GENE	Alzheimer's Hippocampus	Control hippocampus	CSF	Cellular localization	Biological process	Molecular function	Protein Description
IPI00014843.5	LRRC16A	X	X		cytoplasm,cytosol,nucleus	cell organization and biogenesis,metabolic process,response to stimulus,cellular component movement,coagulation	protein binding	Isoform 1 of Leucine-rich repeat-containing protein 16A
IPI00167154.9	MAPKBP1			X			protein binding	Uncharacterized protein
IPI00382756.1	PLRG1			X			protein binding	Isoform 2 of Pleiotropic regulator 1
IPI00410091.3	C11orf73		X		cytoplasm,cytosol,nucleus	development,cell organization and biogenesis,transport,response to stimulus	protein binding,transporter activity	Uncharacterized protein C11orf73
IPI00008524.1	PABPC1	X	X		spliceosomal complex,cytoplasm,nucleus,cytosol	metabolic process,regulation of biological process	protein binding,RNA binding,nucleotide binding,translation regulator activity	Isoform 1 of Polyadenylate-binding protein 1
IPI00644697.2	HEBP2	X	X		cytoplasm			HEBP2 protein (Fragment)
IPI00020058.3	ATP7B			X	mitochondrion,membrane,cytoplasm,Golgi,endosome	development,metabolic process,transport,response to stimulus,cellular homeostasis,reproduction	protein binding,transporter activity,metal ion binding,nucleotide binding,catalytic activity	Isoform 1 of Copper-transporting ATPase 2
IPI00339384.5	RDH11	X			membrane,endoplasmic reticulum,cytoplasm	metabolic process	nucleotide binding,catalytic activity	Isoform 1 of Retinol dehydrogenase 11
IPI00020602.1	CSNK2A2	X	X		cytoplasm,cytosol,nucleus	cell death,cell organization and biogenesis,development,metabolic process,regulation of biological process,response to stimulus,cell communication,cell differentiation	protein binding,nucleotide binding,catalytic activity	Casein kinase II subunit alpha'
IPI00438170.3	SNX12	X		X	membrane	transport,cell communication	protein binding	Isoform 1 of Sorting nexin-12
IPI00030131.3	TMPO	X	X		membrane,nucleus		protein binding,DNA binding	Isoform Beta of Lamina-associated polypeptide 2, isoforms beta/gamma
IPI00003970.2	ME3	X	X		mitochondrion,cytoplasm,organelle lumen	metabolic process	metal ion binding,nucleotide binding,catalytic activity	NADP-dependent malic enzyme, mitochondrial
IPI00014899.3	BRPF3			X		metabolic process,regulation of biological process		cDNA FLJ20744 fis, clone HEP06585
IPI00018140.3	SYNCRIP	X	X		spliceosomal complex,membrane,endoplasmic reticulum,cytoplasm,organelle lumen,nucleus	metabolic process,regulation of biological process	protein binding,RNA binding,nucleotide binding	Isoform 1 of Heterogeneous nuclear ribonucleoprotein Q
IPI00296191.1	ATP6V1H	X	X		membrane,cytoplasm,vacuole,cytosol	metabolic process,transport,regulation of biological process,response to stimulus,defense response,cell communication,cellular homeostasis	protein binding,transporter activity,catalytic activity,enzyme regulator activity	Isoform 1 of V-type proton ATPase subunit H
IPI00021817.1	PROC			X	extracellular,membrane,endoplasmic reticulum,cytoplasm,Golgi,organelle lumen	cell death,metabolic process,regulation of biological process,response to stimulus,cellular component movement,coagulation	protein binding,metal ion binding,catalytic activity	Vitamin K-dependent protein C
IPI00024048.1	CDH15			X	membrane	cell organization and biogenesis,regulation of biological process,cell differentiation	metal ion binding	Cadherin-15
IPI00028932.7	MAST3			X		metabolic process	protein binding,metal ion binding,nucleotide binding,catalytic activity	Microtubule-associated serine/threonine-protein kinase 3
IPI00003907.1	PCDHGC5			X	membrane		metal ion binding	Isoform 1 of Protocadherin gamma-C5
IPI00103755.1	UNC5D			X		regulation of biological process,response to stimulus,cell communication	protein binding	Isoform 2 of Netrin receptor UNC5D
IPI00001639.2	KPNB1	X	X		membrane,cytoplasm,organelle lumen,cytosol,nucleus	cell death,cell organization and biogenesis,metabolic process,transport,regulation of biological process,response to stimulus,cell communication,reproduction	protein binding,transporter activity,metal ion binding	Importin subunit beta-1
IPI00104907.3	MIS18BP1			X	organelle lumen,chromosome,nucleus	cell organization and biogenesis,metabolic process,cell division	DNA binding	Isoform 1 of Mis18-binding protein 1
IPI00300886.5	SLC25A32	X			membrane,mitochondrion,cytoplasm	transport,metabolic process	transporter activity	Mitochondrial folate transporter/carrier
IPI00428741.1	LINC00094			X				LP2477
IPI00216951.2	DARS	X	X		cytoplasm,cytosol	cell organization and biogenesis,metabolic process	protein binding,nucleotide binding,catalytic activity	Aspartyl-tRNA synthetase, cytoplasmic
IPI00159049.3	SETBP1			X	nucleus		DNA binding	Isoform 1 of SET-binding protein
IPI00301023.5	PALM	X	X		membrane,cytoplasm	cell organization and biogenesis,development,transport,metabolic process,regulation of biological process,response to stimulus,cellular component movement,cell communication,cell differentiation	protein binding	Isoform 1 of Paralemmin-1
IPI00014232.1	ARL6IP1	X			membrane,endoplasmic reticulum,cytoplasm		protein binding	ADP-ribosylation factor-like protein 6-interacting protein 1
IPI00294158.1	BLVRA	X	X		cytoplasm,cytosol	metabolic process	metal ion binding,nucleotide binding,catalytic activity	Biliverdin reductase A

IPI	GENE	Alzheimer's Hippocampus	Control hippocampus	CSF	Cellular localization	Biological process	Molecular function	Protein Description
IPI00045939.4	ADO			X	mitochondrion,cytoplasm	metabolic process	metal ion binding,catalytic activity	2-aminoethanethiol dioxygenase
IPI00746756.3	ARHGAP44		X		cytoplasm,cytosol	metabolic process,regulation of biological process,response to stimulus,cell communication	protein binding,enzyme regulator activity	Rho GTPase-activating protein 44
IPI00334282.2	WNT16			X	extracellular,cytoplasm	development	protein binding	Protein FAM3C
IPI00745906.2	SUOX	X	X		mitochondrion,cytoplasm,organelle lumen,cytosol	metabolic process,response to stimulus	metal ion binding,catalytic activity	Sulfite oxidase, mitochondrial
IPI00306959.12	KRT7			X	cytoskeleton,cytoplasm,Golgi,organelle lumen,nucleus	cell organization and biogenesis,metabolic process,regulation of biological process	protein binding,structural molecule activity	Keratin, type II cytoskeletal 7
IPI00386122.4	MOB4		X		membrane,cytoplasm,Golgi	transport	protein binding,metal ion binding	Isoform 1 of Mps one binder kinase activator-like 3
IPI00219813.1	RTN1	X	X		endoplasmic reticulum,cytoplasm			Isoform RTN1-C of Reticulon-1
IPI00030530.6	TMEM55B		X		membrane,cytoplasm,vacuole,endosome		catalytic activity	Isoform 1 of Transmembrane protein 55B
IPI00916402.1	AAK1	X	X		membrane,cytoplasm	cell organization and biogenesis,transport,metabolic process,regulation of biological process,response to stimulus,cell communication	protein binding,nucleotide binding,catalytic activity	Isoform 1 of AP2-associated protein kinase 1
IPI00456925.3	DBNL	X	X		cytoskeleton,cytoplasm,cytosol	cell death,cell organization and biogenesis,transport,metabolic process,regulation of biological process,response to stimulus,cell communication	protein binding,enzyme regulator activity	Isoform 1 of Drebrin-like protein
IPI00013004.1	PDXK	X	X	X	cytoplasm,organelle lumen,cytosol,nucleus	cell proliferation,metabolic process	protein binding,metal ion binding,nucleotide binding,catalytic activity	Isoform 1 of Pyridoxal kinase
IPI00604711.3	KIF1A	X	X		cytoskeleton,cytoplasm,nucleus	cell death,transport,cellular component movement	protein binding,motor activity,nucleotide binding,catalytic activity	Isoform 1 of Kinesin-like protein KIF1A
IPI00221067.1	PTPRA		X			metabolic process	catalytic activity	Isoform 2 of Receptor-type tyrosine-protein phosphatase alpha
IPI00020431.2	TGFBR2			X	cell surface,membrane	cell death,cell proliferation,development,metabolic process,regulation of biological process,response to stimulus,cell communication,cell differentiation	protein binding,signal transducer activity,metal ion binding,nucleotide binding,receptor activity,catalytic activity	Isoform 1 of TGF-beta receptor type-2
IPI00028264.3	EXOC8		X		membrane,cytoplasm,nucleus	transport	protein binding	Exocyst complex component 8
IPI00299149.2	SUMO2	X	X	X			protein binding	Small ubiquitin-related modifier 2
IPI00008274.8	CAP1	X	X	X	cytoskeleton,membrane,cytoplasm	cell organization and biogenesis,development,metabolic process,transport,regulation of biological process,response to stimulus,cellular component movement,cell communication,cell differentiation,coagulation	protein binding	Isoform 1 of Adenylyl cyclase-associated protein 1
IPI00031370.3	TUBB2B	X	X		cytoskeleton,cytoplasm	cell organization and biogenesis,development,metabolic process,cellular component movement,cell differentiation	protein binding,nucleotide binding,structural molecule activity,catalytic activity	Tubulin beta-2B chain
IPI00396130.6	SRCIN1	X	X					Isoform 4 of SRC kinase signaling inhibitor 1
IPI00465352.2	CAPS	X	X		membrane,cytoplasm,nucleus	regulation of biological process,response to stimulus,cell communication	metal ion binding	Calcyphosin
IPI00022284.3	PRNP			X	membrane,endoplasmic reticulum,cytoplasm,Golgi,organelle lumen,nucleus	cell death,cell proliferation,development,cell organization and biogenesis,metabolic process,regulation of biological process,response to stimulus,cell communication,cellular homeostasis,cell differentiation	protein binding,metal ion binding	Isoform 1 of Major prion protein
IPI00217784.1	NDRG4	X	X					Isoform 4 of Protein NDRG4
IPI00022002.6	MRPS27		X		cytoplasm,ribosome			cDNA FLJ54536, highly similar to Mitochondrial 28S ribosomal protein S27
IPI00218775.2	FKBP5		X		membrane,cytoplasm,organelle lumen,nucleus	metabolic process	protein binding,catalytic activity	Peptidyl-prolyl cis-trans isomerase FKBP5
IPI00293564.5	HMGCL		X		mitochondrion,membrane,cytoplasm,organelle lumen	development,cell organization and biogenesis,metabolic process,response to stimulus	protein binding,metal ion binding,catalytic activity	Hydroxymethylglutaryl-CoA lyase, mitochondrial
IPI00018235.3	PEF1	X	X		membrane,cytoplasm	response to stimulus	protein binding,metal ion binding	Peflin
IPI00005159.3	ACTR2	X	X	X	cytoskeleton,cytoplasm	cellular component movement	protein binding,nucleotide binding	Actin-related protein 2
IPI00619924.2	RAP1GDS1		X				protein binding	rap1 GTPase-GDP dissociation stimulator 1 isoform 4

IPI	GENE	Alzheimer's Hippocampus	Control hippocampus	CSF	Cellular localization	Biological process	Molecular function	Protein Description
IPI00180240.2	TMSB4XP8	X	X	X	cytoplasm	cell organization and biogenesis	protein binding	Thymosin beta-4-like protein 3
IPI00028888.1	HNRNPD	X	X		cytoplasm,organelle lumen,cytosol,nucleus	metabolic process,regulation of biological process	protein binding,DNA binding,RNA binding,nucleotide binding	Isoform 1 of Heterogeneous nuclear ribonucleoprotein D0
IPI00413108.4	RPSA	X	X		cytoplasm,ribosome	metabolic process	structural molecule activity	33 kDa protein
IPI00004839.1	CRKL	X	X		cytoplasm,cytosol	development,regulation of biological process,response to stimulus,cell communication	signal transducer activity,protein binding	Crk-like protein
IPI00046057.1	STXBP1	X	X	X		transport		Isoform 2 of Syntaxin-binding protein 1
IPI00023510.1	RAB5A	X	X		membrane,cytoplasm,nucleus,endosome	development,cell organization and biogenesis,transport,metabolic process,regulation of biological process,response to stimulus,cell communication,cell differentiation,coagulation	protein binding,nucleotide binding,catalytic activity	Ras-related protein Rab-5A
IPI00291175.7	VCL	X	X	X	cytoskeleton		structural molecule activity,catalytic activity	Isoform 1 of Vinculin
IPI00218474.6	ENO3			X	membrane,cytoplasm,cytosol	metabolic process,response to stimulus	protein binding,metal ion binding,catalytic activity	Isoform 1 of Beta-enolase
IPI00020226.2	ACOX3		X		mitochondrion,cytoplasm,organelle lumen	metabolic process	protein binding,catalytic activity	Isoform 1 of Peroxisomal acyl-coenzyme A oxidase 3
IPI00642238.4	HP1BP3	X	X		chromosome,nucleus	cell organization and biogenesis,metabolic process	DNA binding	Isoform 1 of Heterochromatin protein 1-binding protein 3
IPI00154858.2	PEAR1			X	membrane	transport	protein binding	Platelet endothelial aggregation receptor 1
IPI00784201.3	CEP290	X			cytoskeleton,membrane,cytoplasm	development,cell organization and biogenesis,transport,cell differentiation	protein binding,transcription regulator activity	291 kDa protein
IPI00465248.5	ENO1	X	X	X	membrane,cytoplasm,cytosol,nucleus	cell organization and biogenesis,metabolic process,regulation of biological process,response to stimulus,cell growth	protein binding,DNA binding,metal ion binding,catalytic activity	Isoform alpha-enolase of Alpha-enolase
IPI00301224.2	TMLHE	X	X		mitochondrion,cytoplasm,organelle lumen	metabolic process,regulation of biological process	metal ion binding,catalytic activity	Isoform A of Trimethyllysine dioxygenase, mitochondrial
IPI00169331.1	SGMS2			X	membrane,cytoplasm,Golgi	metabolic process	catalytic activity	Phosphatidylcholine:ceramide cholinephosphotransferase 2
IPI00550451.1	PPP1CA		X		cytoplasm,organelle lumen,nucleus,cytosol	cell division,metabolic process	protein binding,metal ion binding,catalytic activity	Serine/threonine-protein phosphatase PP1-alpha catalytic subunit
IPI00301489.3	UROD	X	X		cytoskeleton,cytoplasm,organelle lumen,cytosol,nucleus	development,metabolic process,response to stimulus	metal ion binding,catalytic activity	Uroporphyrinogen decarboxylase
IPI00030968.4	C9orf142	X						Isoform 1 of Uncharacterized protein C9orf142
IPI00156005.3	NOVA2		X		nucleus		RNA binding	RNA-binding protein Nova-2
IPI00410270.5	SLC4A4	X	X		membrane	transport	protein binding,transporter activity	Isoform 1 of Electrogenic sodium bicarbonate cotransporter 1
IPI00658210.1	WDR48	X	X		cytoplasm,vacuole,nucleus	metabolic process	protein binding	Isoform 1 of WD repeat-containing protein 48
IPI00007404.1	ATG7	X	X		cytoplasm	cell death,cell organization and biogenesis,development,transport,metabolic process,regulation of biological process,response to stimulus,cell communication,cell differentiation	protein binding,nucleotide binding,catalytic activity	Isoform 1 of Ubiquitin-like modifier-activating enzyme ATG7
IPI00003351.2	ECM1			X	extracellular	cell proliferation,development,metabolic process,regulation of biological process,response to stimulus,cell communication	protein binding,signal transducer activity	Isoform 1 of Extracellular matrix protein 1
IPI00442865.1	SLC12A1			X	membrane	transport		CDNA FLJ26488 fis, clone KDN05770, highly similar to Bumetanide-sensitive sodium-(potassium)-chloride cotransporter 2
IPI00016685.3	ENAM			X	extracellular	development	structural molecule activity	Enamelin
IPI00018136.1	VCAM1			X	extracellular,cell surface,cytoskeleton,membrane	cell proliferation,development,regulation of biological process,response to stimulus,cellular component movement,cell communication,defense response,cellular homeostasis,cell differentiation	protein binding	Isoform 1 of Vascular cell adhesion protein 1
IPI00002211.4	SEMA6A			X	membrane		protein binding	Isoform 2 of Semaphorin-6A
IPI00643166.1	PGM3	X	X			metabolic process	metal ion binding,catalytic activity	phosphoglucomutase 3
IPI00026230.1	HNRNPH2		X	X	cytoskeleton,cytoplasm,organelle lumen,nucleus	metabolic process	RNA binding,nucleotide binding	Heterogeneous nuclear ribonucleoprotein H2
IPI00301518.6	MOB1A	X			cytoplasm,cytosol	regulation of biological process,response to stimulus,cell communication	protein binding,metal ion binding	Isoform 1 of Mps one binder kinase activator-like 1B
IPI00014361.1	TSTA3		X		cytoplasm	metabolic process	nucleotide binding,catalytic activity	GDP-L-fucose synthase

IPI	GENE	Alzheimer's Hippocampus	Control hippocampus	CSF	Cellular localization	Biological process	Molecular function	Protein Description
IPI00442745.1	LOC100507610			X				CDNA FLJ26780 fis, clone PRS03837
IPI00377214.1	NLRX1		X			response to stimulus	nucleotide binding,catalytic activity	Isoform 2 of NLR family member X1
IPI00003909.1	SLC2A3	X	X		membrane	transport,metabolic process	transporter activity	Solute carrier family 2, facilitated glucose transporter member 3
IPI00030919.3	LAMTOR3	X	X	X	membrane,cytoplasm,endosome	regulation of biological process,response to stimulus,cell communication	protein binding	Ragulator complex protein LAMTOR3
IPI00514893.3	DAAM2		X	X		cell organization and biogenesis	protein binding	Disheveled-associated activator of morphogenesis 2
IPI00299033.1	KPNA3	X	X		membrane,cytoplasm,organelle lumen,cytosol,nucleus	cell organization and biogenesis,transport,regulation of biological process,response to stimulus,cell communication	protein binding,transporter activity	Importin subunit alpha-3
IPI00398992.7	CHD8			X	organelle lumen,nucleus	cell death,development,cell organization and biogenesis,metabolic process,regulation of biological process,response to stimulus,cell communication	protein binding,DNA binding,nucleotide binding,catalytic activity	Isoform 1 of Chromodomain-helicase-DNA-binding protein 8
IPI00019501.1	EFNB3			X	membrane	development,cell organization and biogenesis,regulation of biological process,response to stimulus,cell communication,cell differentiation	protein binding,signal transducer activity,receptor activity,catalytic activity	Ephrin-B3
IPI00878517.1	ALB			X	extracellular	transport		Uncharacterized protein
IPI00412713.4	SAMM50	X	X		mitochondrion,membrane,cytoplasm	cell organization and biogenesis,transport,metabolic process	protein binding	Sorting and assembly machinery component 50 homolog
IPI00307276.2	ADAMTS4			X	extracellular	development,metabolic process	protein binding,metal ion binding,catalytic activity	A disintegrin and metalloproteinase with thrombospondin motifs 4
IPI00027078.3	CPD			X	membrane,nucleus	metabolic process,response to stimulus	metal ion binding,catalytic activity	Carboxypeptidase D
IPI00007960.4	POSTN			X	extracellular	cell organization and biogenesis,development,regulation of biological process,response to stimulus,cell communication	protein binding	Isoform 1 of Periostin
IPI00020530.1	ACOT13	X	X		mitochondrion,cytoplasm	cell organization and biogenesis	catalytic activity	Acyl-coenzyme A thioesterase 13
IPI00329331.6	UGP2	X	X		cytoplasm,cytosol	metabolic process,response to stimulus	protein binding,metal ion binding,nucleotide binding,catalytic activity	Isoform 1 of UTP--glucose-1-phosphate uridylyltransferase
IPI00444605.1	ACTA2			X	cytoskeleton		structural molecule activity	cDNA FLJ45296 fis, clone BRHIP3003340, moderately similar to Actin, alpha skeletal muscle 2
IPI00000087.1	SCN2B		X	X	membrane	development,transport,regulation of biological process,response to stimulus,cell communication,cellular homeostasis	protein binding,transporter activity	Sodium channel subunit beta-2
IPI00022664.3	RABGGTA		X			metabolic process	protein binding,metal ion binding,catalytic activity	Geranylgeranyl transferase type-2 subunit alpha
IPI00477355.3	KIAA1279		X		mitochondrion,cytoplasm	development,transport,cell differentiation	protein binding	KIF1-binding protein
IPI00107625.1	SNAP25		X				protein binding	Isoform SNAP-25a of Synaptosomal-associated protein 25
IPI00148020.3	BRSK1	X			cytoskeleton,membrane,cytoplasm,organelle lumen,nucleus	cell organization and biogenesis,development,transport,metabolic process,regulation of biological process,response to stimulus,cell communication,cell differentiation	protein binding,metal ion binding,nucleotide binding,catalytic activity	Isoform 1 of Serine/threonine-protein kinase BRSK1
IPI00171527.10	MAPK1IP1L	X	X		membrane	metabolic process,regulation of biological process,response to stimulus,cell communication	signal transducer activity,DNA binding,transcription regulator activity,receptor activity	MAPK-interacting and spindle-stabilizing protein-like
IPI00062730.5	C16orf45		X	X				Uncharacterized protein C16orf45
IPI00010575.4	ERV3-2			X		metabolic process	catalytic activity	KIAA1466 protein
IPI00796777.1	CRYAA			X			structural molecule activity	Uncharacterized protein
IPI00005652.6	WSCD2			X	membrane		catalytic activity	Isoform 1 of WSC domain-containing protein 2
IPI00010470.3	SNAP25	X	X	X	cytoskeleton,membrane,cytoplasm,endosome	development,cell organization and biogenesis,metabolic process,transport,regulation of biological process,response to stimulus,cell communication,cell differentiation	protein binding,transporter activity	Isoform SNAP-25b of Synaptosomal-associated protein 25
IPI00026817.3	FNTB	X			cytoskeleton	cell proliferation,metabolic process,regulation of biological process,response to stimulus	protein binding,metal ion binding,catalytic activity	Protein farnesyltransferase subunit beta
IPI00873027.2	CARKD	X	X		mitochondrion,cytoplasm		nucleotide binding,catalytic activity	Isoform 1 of Carbohydrate kinase domain-containing protein
IPI00024570.1	SEMA3G			X	extracellular,membrane	development	protein binding,receptor activity	Semaphorin-3G

IPI	GENE	Alzheimer's Hippocampus	Control hippocampus	CSF	Cellular localization	Biological process	Molecular function	Protein Description
IPI00020510.1	CISD1	X	X		mitochondrion,membrane,cytoplasm	metabolic process,regulation of biological process	metal ion binding	CDGSH iron-sulfur domain-containing protein 1
IPI00023340.2	KAT6A			X	organelle lumen,chromosome,nucleus	development,cell organization and biogenesis,metabolic process,regulation of biological process,cell differentiation	protein binding,DNA binding,metal ion binding,catalytic activity	Histone acetyltransferase MYST3
IPI00159927.2	NCAN	X	X	X	extracellular	development,cell organization and biogenesis,regulation of biological process,response to stimulus,cell communication,reproduction,cell differentiation	protein binding,metal ion binding	Neurocan core protein
IPI00401283.4	MEGF9			X	membrane		protein binding,metal ion binding	Multiple epidermal growth factor-like domains protein 9
IPI00945830.2	CADPS	X	X		cytoplasm	cell organization and biogenesis,transport,regulation of biological process,cell communication		CADPS protein
IPI00787265.1	LOC729034			X		metabolic process	metal ion binding,catalytic activity	similar to puromycin sensitive aminopeptidase
IPI00465233.1	EIF3L	X	X			metabolic process	protein binding,RNA binding	Uncharacterized protein
IPI00024773.1	FARP1	X	X		cytoskeleton,membrane,cytoplasm	metabolic process,regulation of biological process,response to stimulus,cell communication	protein binding,enzyme regulator activity	Isoform 1 of FERM, RhoGEF and pleckstrin domain-containing protein 1
IPI00017297.1	MATR3	X	X		membrane,organelle lumen,nucleus		protein binding,RNA binding,metal ion binding,nucleotide binding,structural molecule activity	Matrin-3
IPI00952909.1	Sep-11	X	X		cytoskeleton,cytoplasm	cell organization and biogenesis,cell division	protein binding,nucleotide binding	Isoform 1 of Septin-11
IPI00168404.5	ZBTB34			X			protein binding,metal ion binding	Zinc finger and BTB domain-containing protein 34
IPI00216319.3	YWHAH	X	X	X	cytoplasm	cell organization and biogenesis,development,metabolic process,transport,regulation of biological process,response to stimulus,cell communication,cell differentiation	protein binding	14-3-3 protein eta
IPI00184094.2	B3GNT8			X	membrane,cytoplasm,Golgi	metabolic process	catalytic activity	UDP-GlcNAc:betaGal beta-1,3-N-acetylglucosaminyltransferase 8
IPI00005565.2	DGKQ		X		cytoskeleton,membrane,cytoplasm,organelle lumen,cytosol,nucleus	metabolic process,regulation of biological process,response to stimulus,cell communication,coagulation	protein binding,metal ion binding,nucleotide binding,catalytic activity	Diacylglycerol kinase theta
IPI00028570.2	GSK3B	X	X		cytoskeleton,membrane,cytoplasm,organelle lumen,nucleus,cytosol	cell death,development,cell organization and biogenesis,transport,metabolic process,regulation of biological process,response to stimulus,cell communication,cell differentiation	protein binding,nucleotide binding,catalytic activity	Isoform 1 of Glycogen synthase kinase-3 beta
IPI00219860.3	PTPRD	X	X			metabolic process	protein binding,catalytic activity	Isoform 3 of Receptor-type tyrosine-protein phosphatase delta
IPI00787932.3	ZNF891			X	nucleus	metabolic process,regulation of biological process	DNA binding,metal ion binding	Zinc finger protein 891
IPI01013214.2	IGHV3-74			X				Rheumatoid factor RF-IP14
IPI00009104.7	RUVBL2	X	X		membrane,cytoplasm,organelle lumen,chromosome,nucleus	cell organization and biogenesis,metabolic process,regulation of biological process,response to stimulus	protein binding,DNA binding,nucleotide binding,catalytic activity	RuvB-like 2
IPI00019580.1	PLG		X	X	extracellular,cell surface,membrane,cytoplasm,organelle lumen	cell death,cell proliferation,development,cell organization and biogenesis,metabolic process,transport,regulation of biological process,response to stimulus,cellular component movement,cellular homeostasis,cell differentiation,coagulation	protein binding,metal ion binding,catalytic activity	Plasminogen
IPI00329605.6	MSH3			X	organelle lumen,chromosome,nucleus	cell organization and biogenesis,development,metabolic process,regulation of biological process,response to stimulus,reproduction	protein binding,DNA binding,nucleotide binding,catalytic activity	DNA mismatch repair protein Msh3
IPI00456589.1	GALNT11			X	membrane,cytoplasm,Golgi	metabolic process	catalytic activity	Isoform 1 of Polypeptide N-acetylgalactosaminyltransferase 11
IPI00028275.2	CKAP5	X	X		cytoskeleton,cytoplasm,cytosol	cell organization and biogenesis,cell division,transport	protein binding	Isoform 1 of Cytoskeleton-associated protein 5
IPI00219103.6	HPCA	X	X			development	protein binding,metal ion binding	Neuron-specific calcium-binding protein hippocalcin
IPI00031107.1	HSDL2	X	X					Isoform 2 of Hydroxysteroid dehydrogenase-like protein 2
IPI00152540.3	CD109	X	X	X	extracellular,membrane	metabolic process,regulation of biological process	enzyme regulator activity	Isoform 1 of CD109 antigen
IPI00022643.3	TRIM36		X		cytoskeleton,membrane,cytoplasm	transport,metabolic process,reproduction	protein binding,transporter activity,metal ion binding,catalytic activity	E3 ubiquitin-protein ligase TRIM36

IPI	GENE	Alzheimer's Hippocampus	Control hippocampus	CSF	Cellular localization	Biological process	Molecular function	Protein Description
IPI00303210.4	ENPP2			X		response to stimulus	metal ion binding,receptor activity,catalytic activity	Isoform 2 of Ectonucleotide pyrophosphatase/phosphodiesterase family member 2
IPI00747764.3	KPNA6		X		cytoplasm,nucleus	transport	protein binding,transporter activity	cDNA FLJ53229, highly similar to Importin alpha-7 subunit
IPI00289831.5	PTPRS	X	X	X	membrane	development,metabolic process	protein binding,signal transducer activity,receptor activity,catalytic activity	Isoform 1 of Receptor-type tyrosine-protein phosphatase S
IPI00186290.6	EEF2	X	X		cytoplasm,cytosol	metabolic process	RNA binding,nucleotide binding,catalytic activity	Elongation factor 2
IPI00795013.1	LPHN3			X	membrane	regulation of biological process,response to stimulus,cell communication	protein binding,signal transducer activity,receptor activity,catalytic activity	149 kDa protein
IPI00298258.4	UNC13B			X		regulation of biological process,response to stimulus,cell communication	protein binding,metal ion binding	Uncharacterized protein
IPI00432723.3	XYLT2			X	membrane,endoplasmic reticulum,cytoplasm,Golgi	metabolic process	catalytic activity	Isoform 1 of Xylosyltransferase 2
IPI00031087.7	SGIP1	X	X		membrane,cytoplasm	cell organization and biogenesis,transport,regulation of biological process,response to stimulus	protein binding	Isoform 1 of SH3-containing GRB2-like protein 3-interacting protein 1
IPI00793576.1	PRB4			X	membrane	regulation of biological process,response to stimulus,cell communication		7 kDa protein
IPI00748303.3	LOC730732	X			organelle lumen,nucleus		metal ion binding	Uncharacterized protein
IPI00022445.1	PPBP			X	extracellular,cytoplasm,organelle lumen	transport,cell division,regulation of biological process,response to stimulus,defense response,coagulation	protein binding,transporter activity	Platelet basic protein
IPI00289776.4	MYCBP2		X		membrane			probable E3 ubiquitin-protein ligase MYCBP2
IPI00291412.1	PPM1F	X	X		cytoplasm,cytosol	cell death,cell organization and biogenesis,metabolic process,regulation of biological process,response to stimulus,cellular component movement,cell communication	protein binding,metal ion binding,catalytic activity	Protein phosphatase 1F
IPI00020091.1	ORM2			X	extracellular	transport,regulation of biological process,response to stimulus,defense response		Alpha-1-acid glycoprotein 2
IPI00941161.1	USO1	X	X		membrane,cytoplasm,Golgi,cytosol	cell organization and biogenesis,metabolic process,transport	protein binding,transporter activity,nucleotide binding,catalytic activity	Isoform 1 of General vesicular transport factor p115
IPI00387117.5	IGKV3D-20			X	extracellular,membrane	metabolic process,regulation of biological process,response to stimulus,defense response	protein binding	Ig kappa chain V-III region Ti
IPI00550571.3	C5orf15			X	membrane			Keratinocyte-associated transmembrane protein 2
IPI00790899.1	NUCB1			X			metal ion binding	nucleobindin 1, isoform CRA_b
IPI00024660.1	IST1	X	X		cytoplasm	cell division	protein binding	Isoform 1 of IST1 homolog
IPI00013319.8	RAPSN			X	cytoskeleton,membrane	cell communication	protein binding,metal ion binding	Isoform 2 of 43 kDa receptor-associated protein of the synapse
IPI00019927.2	PSMD7	X	X		cytoplasm,proteasome,organelle lumen,cytosol,nucleus	cell death,metabolic process,regulation of biological process,response to stimulus,cell communication	protein binding	26S proteasome non-ATPase regulatory subunit 7
IPI00844511.1	MAN1A1			X	membrane,endoplasmic reticulum,cytoplasm,Golgi	metabolic process	metal ion binding,catalytic activity	Mannosyl-oligosaccharide 1,2-alpha-mannosidase IA
IPI00301395.4	CPVL		X	X		metabolic process	catalytic activity	Probable serine carboxypeptidase CPVL
IPI00374970.4	Sep-10	X	X		cytoskeleton,cytoplasm	cell division	nucleotide binding	Isoform 1 of Septin-10
IPI00419373.1	HNRNPA3	X	X		spliceosomal complex,organelle lumen,nucleus	metabolic process	RNA binding,nucleotide binding	Isoform 1 of Heterogeneous nuclear ribonucleoprotein A3
IPI00013679.2	DUT	X	X			metabolic process	catalytic activity	27 kDa protein
IPI00289396.6	PHYHIPL	X	X		mitochondrion,cytoplasm		protein binding	Isoform 1 of Phytanoyl-CoA hydroxylase-interacting protein-like
IPI00299076.1	EBAG9			X	membrane,cytoplasm,Golgi	cell death,cell organization and biogenesis,regulation of biological process,cell growth	enzyme regulator activity	Receptor-binding cancer antigen expressed on SiSo cells (Fragment)
IPI00155601.1	MACROD1	X	X		membrane,mitochondrion,cytoplasm	metabolic process	protein binding,catalytic activity	MACRO domain-containing protein 1
IPI00025276.2	TNXB			X	extracellular,cytoskeleton	cell organization and biogenesis,metabolic process,regulation of biological process,response to stimulus,cell communication	protein binding	Isoform XB of Tenascin-X
IPI00010349.1	AGPS		X		membrane,mitochondrion,cytoplasm,organelle lumen,nucleus	metabolic process,response to stimulus	catalytic activity	Alkyldihydroxyacetonephosphate synthase, peroxisomal

IPI	GENE	Alzheimer's Hippocampus	Control hippocampus	CSF	Cellular localization	Biological process	Molecular function	Protein Description
IPI00054042.1	GTF2I	X			cytoplasm,organelle lumen,nucleus	development,metabolic process,regulation of biological process,response to stimulus,cell communication	protein binding,DNA binding	Isoform 1 of General transcription factor II-I
IPI00552323.4	IGLV4-60			X				Similar to V5-4 protein
IPI00032516.5	AP1M1	X	X		membrane,cytoplasm,Golgi,vacuole,cytosol	cell organization and biogenesis,transport,regulation of biological process,response to stimulus,defense response	protein binding	AP-1 complex subunit mu-1
IPI00031005.3	SGK196			X	membrane	metabolic process	nucleotide binding,catalytic activity	Protein kinase-like protein SgK196
IPI00654584.7	NPTN	X	X				protein binding	Isoform 4 of Neuroplastin
IPI00021854.1	APOA2	X	X	X	extracellular,endoplasmic reticulum,cytoplasm,organelle lumen	development,cell organization and biogenesis,metabolic process,transport,regulation of biological process,response to stimulus,defense response	protein binding,transporter activity,enzyme regulator activity	Apolipoprotein A-II
IPI00604756.2	NRBP1		X		membrane,cytoplasm,organelle lumen,nucleus	transport,metabolic process	protein binding,nucleotide binding,catalytic activity	Nuclear receptor-binding protein
IPI00430622.1	SPG20	X	X		cytoplasm	cell death	protein binding	Spartin
IPI00007617.2	OR52A1			X	membrane	regulation of biological process,response to stimulus,cell communication	signal transducer activity,receptor activity	Olfactory receptor 52A1
IPI00006475.3	UBE2D4	X	X			metabolic process	protein binding,nucleotide binding,catalytic activity	Ubiquitin-conjugating enzyme E2 D4
IPI00018931.6	VPS35	X	X		membrane,cytoplasm,cytosol,endosome	cell death,transport	protein binding	Vacuolar protein sorting-associated protein 35
IPI00167710.5	FBLN7			X	extracellular		protein binding,metal ion binding	Isoform 1 of Fibulin-7
IPI00170766.4	CASC5			X	membrane			Isoform 2 of Protein CASC5
IPI00015614.4	PRSS3			X	extracellular	metabolic process,response to stimulus,cellular component movement,defense response	protein binding,metal ion binding,catalytic activity	Isoform A of Trypsin-3
IPI00027827.2	SOD3		X	X	extracellular,mitochondrion,cytoplasm,nucleus,cytosol	metabolic process,response to stimulus	antioxidant activity,protein binding,metal ion binding,catalytic activity	Extracellular superoxide dismutase [Cu-Zn]
IPI00654888.4	KLKB1			X	extracellular,membrane	cell organization and biogenesis,metabolic process,regulation of biological process,response to stimulus,defense response,coagulation	protein binding,catalytic activity	Plasma kallikrein
IPI00032532.1	GAS6			X	extracellular,cell surface,membrane		metal ion binding	Isoform 2 of Growth arrest-specific protein 6
IPI00013897.1	ADAM10			X	cell surface,cytoskeleton,membrane,cytoplasm,Golgi,nucleus	cell proliferation,cell organization and biogenesis,development,metabolic process,regulation of biological process,response to stimulus,cellular component movement,cell communication,cell growth	protein binding,metal ion binding,catalytic activity	Disintegrin and metalloproteinase domain-containing protein 10
IPI00008455.1	MYO6	X	X		cytoskeleton		motor activity,nucleotide binding,catalytic activity	Isoform 2 of Myosin-VI
IPI00024993.4	ECHS1	X	X		mitochondrion,cytoplasm,organelle lumen	metabolic process	protein binding,catalytic activity	Enoyl-CoA hydratase, mitochondrial
IPI00219532.1	ELMO1	X	X		cytoskeleton,membrane,cytoplasm,cytosol	cell death,cell organization and biogenesis,transport,regulation of biological process,response to stimulus,cellular component movement,cell communication,defense response	protein binding	Isoform 1 of Engulfment and cell motility protein 1
IPI00158296.6	VWA8		X		membrane,mitochondrion,cytoplasm	metabolic process	protein binding,nucleotide binding,catalytic activity	Uncharacterized protein
IPI00291440.2	KCTD4		X			cell organization and biogenesis	protein binding	BTB/POZ domain-containing protein KCTD4
IPI00029236.1	IGFBP5			X	extracellular	cell proliferation,development,cell organization and biogenesis,metabolic process,regulation of biological process,response to stimulus,cellular component movement,cell communication,cell differentiation,cell growth	protein binding	Insulin-like growth factor-binding protein 5
IPI00168847.3	HYAL1			X		metabolic process	catalytic activity	Isoform 2 of Hyaluronidase-1
IPI00022078.3	NDRG1	X	X	X	cytoskeleton,membrane,cytoplasm,nucleus,cytosol,endosome	cell organization and biogenesis,development,regulation of biological process,response to stimulus,cell communication,cellular homeostasis,cell differentiation	protein binding	Protein NDRG1
IPI00220361.3	CALB1	X	X	X	cytoplasm,cytosol,nucleus	development,regulation of biological process,response to stimulus,cell communication	protein binding,metal ion binding	Calbindin

IPI	GENE	Alzheimer's Hippocampus	Control hippocampus	CSF	Cellular localization	Biological process	Molecular function	Protein Description
IPI00015891.1	PFDN4	X	X		mitochondrion,cytoplasm,nucleus,cytosol	metabolic process	protein binding	Prefoldin subunit 4
IPI00307257.5	TBC1D9B	X	X		membrane	metabolic process,regulation of biological process	metal ion binding,enzyme regulator activity	Isoform 1 of TBC1 domain family member 9B
IPI00383798.3	PRODH	X	X		mitochondrion,membrane,cytoplasm,organelle lumen	cell death,metabolic process,regulation of biological process	catalytic activity	Isoform 1 of Proline dehydrogenase 1, mitochondrial
IPI00009790.1	PFKP	X	X		cytoplasm,cytosol	cell organization and biogenesis,metabolic process	metal ion binding,nucleotide binding,catalytic activity	6-phosphofructokinase type C
IPI00171705.1	NLGN4Y		X	X	cytoskeleton,cell surface,membrane	development,cell organization and biogenesis,metabolic process	protein binding,receptor activity,catalytic activity	Isoform 1 of Neuroligin-4, Y-linked
IPI00783097.4	GARS	X	X		mitochondrion,cytoplasm,organelle lumen,cytosol	cell death,metabolic process	protein binding,nucleotide binding,catalytic activity	Glycyl-tRNA synthetase
IPI00171473.2	SPON1		X	X	extracellular		protein binding	Spondin-1
IPI00024920.1	ATP5D	X	X	X	membrane,mitochondrion,cytoplasm,organelle lumen	transport,metabolic process,response to stimulus	protein binding,transporter activity,nucleotide binding,catalytic activity	ATP synthase subunit delta, mitochondrial
IPI00016371.1	ERBB4			X	extracellular,cytoskeleton,membrane,mitochondrion,cytoplasm,organelle lumen,cytosol,nucleus	cell death,cell proliferation,cell organization and biogenesis,development,transport,metabolic process,regulation of biological process,response to stimulus,cellular component movement,cell communication,reproduction,cell differentiation	protein binding,signal transducer activity,DNA binding,nucleotide binding,receptor activity,catalytic activity	Isoform JM-A CYT-1 of Receptor tyrosine-protein kinase erbB-4
IPI00032288.3	MANSC1			X	membrane			MANSC domain-containing protein 1
IPI00038378.5	ENOPH1	X	X	X	cytoplasm,cytosol,nucleus	metabolic process	metal ion binding,catalytic activity	Isoform 1 of Enolase-phosphatase E1
IPI00334159.6	VBP1	X	X		cytoplasm,cytosol	metabolic process	protein binding	von Hippel-Lindau binding protein 1, isoform CRA_b
IPI00787781.1	TIMP2			X		metabolic process	protein binding,enzyme regulator activity	similar to Metalloproteinase inhibitor 2 precursor
IPI00296099.6	THBS1			X	extracellular,cell surface,membrane,cytoplasm,organelle lumen	cell death,cell proliferation,cell organization and biogenesis,development,metabolic process,transport,regulation of biological process,response to stimulus,cellular component movement,defense response,cell communication,coagulation	protein binding,metal ion binding	Thrombospondin-1
IPI00470607.3	FAM20C			X	extracellular	development,metabolic process	metal ion binding,catalytic activity	Isoform 1 of Dentin matrix protein 4
IPI00300086.4	QPRT	X			cytoplasm,cytosol	cell organization and biogenesis,metabolic process	protein binding,catalytic activity	Nicotinate-nucleotide pyrophosphorylase [carboxylating]
IPI00026182.5	CAPZA2	X	X		extracellular,cytoskeleton,membrane,cytoplasm,cytosol	cell organization and biogenesis,regulation of biological process,response to stimulus,cellular component movement,defense response,coagulation	protein binding	F-actin-capping protein subunit alpha-2
IPI00791938.1	LLGL1	X	X				protein binding	LLGL1 protein
IPI00292753.7	GAPVD1		X			regulation of biological process,response to stimulus,cell communication	enzyme regulator activity	Isoform 6 of GTPase-activating protein and VPS9 domain-containing protein 1
IPI00021785.2	COX5B		X		membrane,mitochondrion,cytoplasm	metabolic process	transporter activity,metal ion binding,catalytic activity	Cytochrome c oxidase subunit 5B, mitochondrial
IPI00646779.2	TUBB6	X	X		cytoskeleton,cytoplasm	cell organization and biogenesis,metabolic process,cellular component movement	nucleotide binding,structural molecule activity,catalytic activity	TUBB6 protein
IPI00003004.1	SLC25A22	X	X		membrane,mitochondrion,cytoplasm,organelle lumen,nucleus	transport	transporter activity	Mitochondrial glutamate carrier 1
IPI00030738.5	SPECC1	X	X		cytoskeleton,membrane,cytoplasm,Golgi	cell organization and biogenesis,transport	transporter activity	Isoform 2 of Cytospin-B
IPI00218192.3	TMEM110	X	X	X		metabolic process	protein binding,enzyme regulator activity	Isoform 2 of Inter-alpha-trypsin inhibitor heavy chain H4
IPI00878716.1	C1S			X	extracellular	development,response to stimulus,cell differentiation		Uncharacterized protein
IPI00289329.2	EPHB3			X	membrane	cell organization and biogenesis,development,metabolic process,regulation of biological process,response to stimulus,cellular component movement,cell communication,cell differentiation	protein binding,signal transducer activity,nucleotide binding,receptor activity,catalytic activity	Ephrin type-B receptor 3
IPI00398364.3	PPFIA3		X		cell surface,cytoplasm	transport,regulation of biological process,cell communication	protein binding	Isoform 1 of Liprin-alpha-3
IPI00306853.2	CHST3			X	membrane,cytoplasm,Golgi	development,cell organization and biogenesis,metabolic process,regulation of biological process,response to stimulus,cellular component movement,cell differentiation	catalytic activity	Carbohydrate sulfotransferase 3
IPI00304814.4	GPX4	X	X			metabolic process,response to stimulus	antioxidant activity,catalytic activity	Isoform Mitochondrial of Phospholipid hydroperoxide glutathione peroxidase, mitochondrial

IPI	GENE	Alzheimer's Hippocampus	Control hippocampus	CSF	Cellular localization	Biological process	Molecular function	Protein Description
IPI00184650.1	BHLHE23			X	nucleus	cell death,development,metabolic process,regulation of biological process,cell differentiation	DNA binding	Class E basic helix-loop-helix protein 23
IPI00297180.4	CDH9			X	membrane	cell organization and biogenesis	metal ion binding	Cadherin-9
IPI00412264.1	PTN	X		X	extracellular,endoplasmic reticulum,cytoplasm	cell proliferation,development,cell division,regulation of biological process,response to stimulus,cell communication	protein binding,enzyme regulator activity	Pleiotrophin
IPI00418960.2	NDRG4			X				Isoform 3 of Protein NDRG4
IPI00019568.1	F2			X	extracellular,membrane,endoplasmic reticulum,cytoplasm,Golgi,organelle lumen	development,cell organization and biogenesis,metabolic process,regulation of biological process,response to stimulus,defense response,cell proliferation,transport,cellular component movement,cell communication,cellular homeostasis,cell differentiation,cell growth,coagulation	signal transducer activity,protein binding,metal ion binding,receptor activity,catalytic activity	Prothrombin (Fragment)
IPI00004471.2	PDLIM3	X	X		cytoskeleton,cytoplasm	development,cell organization and biogenesis	protein binding,metal ion binding,structural molecule activity	Isoform 1 of PDZ and LIM domain protein 3
IPI00432766.2	NTNG2			X	extracellular			Isoform 2 of Netrin-G2
IPI00027457.1	C1QL1			X	extracellular,cytoplasm	response to stimulus	protein binding	C1q-related factor
IPI00375879.6	KIAA1467			X	membrane			Uncharacterized protein KIAA1467
IPI00016457.5	CRAT		X		mitochondrion,membrane,endoplasmic reticulum,cytoplasm,organelle lumen	transport,metabolic process	protein binding,catalytic activity	Isoform 1 of Carnitine O-acetyltransferase
IPI00414294.3	SHISA7			X				53 kDa protein
IPI00031789.1	IL1RAP			X	extracellular,membrane	cell organization and biogenesis,regulation of biological process,response to stimulus,cell communication,defense response	signal transducer activity,protein binding,receptor activity	Isoform 1 of Interleukin-1 receptor accessory protein
IPI00027341.2	CAPG	X	X	X	cytoskeleton,membrane,cytoplasm,organelle lumen,nucleus	cell organization and biogenesis,regulation of biological process	protein binding	Macrophage-capping protein
IPI00396383.1	VWA1			X	extracellular	cell organization and biogenesis	protein binding	Isoform 1 of von Willebrand factor A domain-containing protein 1
IPI00158992.2	SNAPC4			X	organelle lumen,nucleus	metabolic process,regulation of biological process	DNA binding	snRNA-activating protein complex subunit 4
IPI00410588.4	ADAMTSL3			X	extracellular		protein binding,metal ion binding,catalytic activity	Isoform 1 of ADAMTS-like protein 3
IPI00010193.1	IFNAR2			X	extracellular,membrane	cell proliferation,metabolic process,regulation of biological process,response to stimulus,cell communication,defense response	protein binding,signal transducer activity,receptor activity	Isoform 1 of Interferon alpha/beta receptor 2
IPI00828205.1	IGHM			X			protein binding	IGHM protein
IPI00006130.3	KIAA0494			X			metal ion binding	EF-hand domain-containing protein KIAA0494
IPI00298337.6	SLC14A1	X	X	X	membrane	transport	transporter activity	Isoform 1 of Urea transporter 1
IPI00878240.2	SLC8A1	X	X		membrane	transport,cell communication	protein binding,transporter activity	Uncharacterized protein
IPI00745251.2	MAN1C1			X	membrane,cytoplasm,Golgi	metabolic process	metal ion binding,catalytic activity	Mannosyl-oligosaccharide 1,2-alpha-mannosidase IC
IPI00216699.1	FERMT3		X	X			protein binding	Isoform 2 of Fermitin family homolog 3
IPI00472605.1	HLA-C			X	membrane	response to stimulus	signal transducer activity,protein binding,receptor activity	HLA class I histocompatibility antigen, Cw-2 alpha chain
IPI00647704.1	IGHA1			X	membrane		protein binding	cDNA FLJ41552 fis, clone COLON2004478, highly similar to Protein Tro alpha1 H,myeloma
IPI00216457.7	HIST2H2AA4			X	chromosome,nucleus	cell organization and biogenesis,metabolic process	DNA binding	Histone H2A type 2-A
IPI00296022.1	UQCRH	X	X		membrane,mitochondrion,cytoplasm	cell organization and biogenesis,transport,metabolic process	protein binding,transporter activity,catalytic activity	Cytochrome b-c1 complex subunit 6, mitochondrial
IPI00430803.3	PTRHD1	X	X			metabolic process	catalytic activity	Putative peptidyl-tRNA hydrolase PTRHD1
IPI00215767.1	B4GALT1			X	extracellular,cell surface,membrane,cytoplasm,Golgi	cell death,cell organization and biogenesis,development,metabolic process,regulation of biological process,response to stimulus,defense response,reproduction,cell proliferation,transport,cellular component movement,cell communication,cell differentiation	protein binding,metal ion binding,catalytic activity	Isoform Long of Beta-1,4-galactosyltransferase 1
IPI00242956.5	FCGBP		X	X	extracellular,cytoplasm		protein binding	IgGFc-binding protein

IPI	GENE	Alzheimer's Hippocampus	Control hippocampus	CSF	Cellular localization	Biological process	Molecular function	Protein Description
IPI00402088.3	C1orf95	X	X		membrane			Uncharacterized membrane protein C1orf95
IPI00014263.1	EIF4H	X	X		cytoplasm,cytosol	metabolic process,regulation of biological process	protein binding,RNA binding,nucleotide binding	Isoform Long of Eukaryotic translation initiation factor 4H
IPI00429191.3	ETF1	X	X	X	cytoplasm,cytosol	cell organization and biogenesis,metabolic process,regulation of biological process	protein binding,RNA binding	Eukaryotic peptide chain release factor subunit 1
IPI00335509.3	DPYSL5	X	X		cytoplasm,cytosol	development,cell organization and biogenesis,metabolic process,regulation of biological process,response to stimulus,cell communication,cell differentiation	catalytic activity	Dihydropyrimidinase-related protein 5
IPI00030757.2	ADAMTS2			X	extracellular	cell organization and biogenesis,development,metabolic process,reproduction	metal ion binding,catalytic activity	Isoform LpNPI of A disintegrin and metalloproteinase with thrombospondin motifs 2
IPI00786893.1	LOC730296			X				similar to LYRIC/3D3
IPI00011229.1	CTSD	X	X	X	extracellular,cytoplasm,vacuole	cell death,metabolic process	catalytic activity	Cathepsin D
IPI00646917.1	NUDT21	X	X		cytoskeleton,cytoplasm,organelle lumen,nucleus	cell organization and biogenesis,metabolic process	protein binding,RNA binding,catalytic activity	Cleavage and polyadenylation specificity factor subunit 5
IPI00921496.2	C22orf46			X				cDNA FLJ16614 fis, clone TESTI4013365
IPI00008305.7	HPCAL4	X	X			development	protein binding,metal ion binding	Hippocalcin-like protein 4
IPI00883857.2	HNRNPU	X	X		spliceosomal complex,cell surface,cytoplasm,organelle lumen,nucleus	metabolic process,regulation of biological process	protein binding,DNA binding,RNA binding,nucleotide binding	Isoform Long of Heterogeneous nuclear ribonucleoprotein U
IPI00029628.1	RCN2	X	X		endoplasmic reticulum,cytoplasm,organelle lumen		metal ion binding	Reticulocalbin-2
IPI00009010.3	TRMT112		X			metabolic process	protein binding,catalytic activity	tRNA methyltransferase 112 homolog
IPI00217261.3	TEX2		X					Isoform 2 of Testis-expressed sequence 2 protein
IPI00163230.5	COPS6	X	X		cytoplasm,nucleus	metabolic process	protein binding	COP9 signalosome complex subunit 6
IPI00065312.2	B4GALNT4			X	membrane,cytoplasm,Golgi		catalytic activity	N-acetyl-beta-glucosaminyl-glycoprotein 4-beta-N-acetylgalactosaminyltransferase 1
IPI00554474.4	SSC5D			X	membrane		receptor activity	Scavenger receptor cysteine-rich domain-containing protein LOC284297
IPI00456887.2	HNRNPUL2	X	X		nucleus		protein binding	Heterogeneous nuclear ribonucleoprotein U-like protein 2
IPI00335437.7	ANKZF1			X			protein binding,metal ion binding	Ankyrin repeat and zinc finger domain-containing protein 1
IPI00419880.6	RPS3A	X	X		cytoplasm,ribosome,organelle lumen,cytosol,nucleus	cell death,cell organization and biogenesis,transport,metabolic process,regulation of biological process,cell differentiation,reproduction	protein binding,RNA binding,structural molecule activity	40S ribosomal protein S3a
IPI00103597.4	SORCS1			X	membrane	regulation of biological process,response to stimulus,cell communication	signal transducer activity,protein binding,receptor activity	Isoform 1 of VPS10 domain-containing receptor SorCS1
IPI00166892.3	DPP10	X	X	X	membrane	metabolic process	catalytic activity	inactive dipeptidyl peptidase 10 isoform c
IPI00478860.4	MANEA			X	membrane,cytoplasm,Golgi	metabolic process	catalytic activity	Glycoprotein endo-alpha-1,2-mannosidase
IPI00220754.1	ADD3	X	X				metal ion binding	Isoform 1 of Gamma-adducin
IPI00012440.8	FUCA2			X	extracellular	metabolic process	catalytic activity	Plasma alpha-L-fucosidase
IPI00004962.2	GOLIM4			X	membrane,cytoplasm,Golgi,organelle lumen,nucleus,endosome	transport		Golgi integral membrane protein 4
IPI00220739.3	PGRMC1	X	X	X	cell surface,membrane,endoplasmic reticulum,cytoplasm,organelle lumen,nucleus		metal ion binding,receptor activity	Membrane-associated progesterone receptor component 1
IPI00014958.1	PON2	X	X				catalytic activity	Isoform 1 of Serum paraoxonase/arylesterase 2
IPI00019576.1	F10			X	extracellular,cell surface,membrane,endoplasmic reticulum,cytoplasm,Golgi,organelle lumen	metabolic process,regulation of biological process,response to stimulus,cellular component movement,cell communication,coagulation	protein binding,metal ion binding,catalytic activity	Coagulation factor X
IPI00007047.1	S100A8			X	extracellular,cytoskeleton,membrane,cytoplasm	response to stimulus,defense response	protein binding,metal ion binding	Protein S100-A8
IPI00020096.2	KLC1	X	X		cytoskeleton,cytoplasm,cytosol	cell organization and biogenesis,response to stimulus,cellular component movement,coagulation	protein binding,motor activity,catalytic activity	Isoform A of Kinesin light chain 1
IPI00029997.1	PGLS	X	X	X	cytoplasm,cytosol	metabolic process	catalytic activity	6-phosphogluconolactonase

IPI	GENE	Alzheimer's Hippocampus	Control hippocampus	CSF	Cellular localization	Biological process	Molecular function	Protein Description
IPI00021199.2	STMN3			X	membrane,cytoplasm,Golgi	development,cell organization and biogenesis,metabolic process,regulation of biological process,response to stimulus,cell communication,cell differentiation	protein binding	Stathmin-3
IPI00298928.1	SHOC2	X	X		cytoplasm,nucleus	regulation of biological process,response to stimulus,cell communication	protein binding,enzyme regulator activity	Isoform 1 of Leucine-rich repeat protein SHOC-2
IPI00644346.2	ADAMTSL2			X	extracellular	regulation of biological process,response to stimulus,cell communication	protein binding,metal ion binding,catalytic activity	ADAMTS-like protein 2
IPI00221093.7	RPS17L	X	X		cytoplasm,ribosome,cytosol	cell organization and biogenesis,transport,metabolic process,reproduction	structural molecule activity	40S ribosomal protein S17
IPI00641737.2	HP	X	X	X	extracellular	cell death,metabolic process,regulation of biological process,response to stimulus,defense response,cellular homeostasis	protein binding,catalytic activity	Haptoglobin
IPI00002853.1	PPP2R5E	X	X		cytoplasm	regulation of biological process,response to stimulus,cell communication	protein binding,enzyme regulator activity	Serine/threonine-protein phosphatase 2A 56 kDa regulatory subunit epsilon isoform
IPI00888695.2	IGHV1-69			X			protein binding	immunoglobulin heavy chain variable region
IPI00445278.1	RCC2			X				cDNA FLJ44033 fis, clone TESTI4028062
IPI00016702.4	RABGAP1	X	X		cytoskeleton,cytoplasm,cytosol	metabolic process,regulation of biological process	protein binding,enzyme regulator activity	Isoform 1 of Rab GTPase-activating protein 1
IPI00006721.4	OPA1	X	X		mitochondrion,membrane,cytoplasm	cell death,cell organization and biogenesis,metabolic process,transport,regulation of biological process,response to stimulus,cellular component movement,cell communication	protein binding,metal ion binding,nucleotide binding,catalytic activity	Isoform 1 of Dynamin-like 120 kDa protein, mitochondrial
IPI00297779.7	CCT2	X	X	X	cytoskeleton,cytoplasm,organelle lumen,nucleus,cytosol	cell organization and biogenesis,metabolic process	protein binding,nucleotide binding	T-complex protein 1 subunit beta
IPI00016605.3	C1orf123	X	X	X				UPF0587 protein C1orf123
IPI00745363.4	LOC652113			X			protein binding	immunglobulin heavy chain variable region
IPI00217236.4	TBCA	X	X	X	cytoskeleton,cytoplasm,organelle lumen,nucleus	cell organization and biogenesis,metabolic process	protein binding	Tubulin-specific chaperone A
IPI00220857.3	CAST	X	X				enzyme regulator activity	Isoform 2 of Calpastatin
IPI00302962.1	AMPH			X	cytoplasm	cell organization and biogenesis,transport,response to stimulus,cell communication		Amphiphysin I variant CT4 (Fragment)
IPI00022367.3	ASTN1	X	X	X	membrane	cellular component movement	protein binding	Isoform 2 of Astrotactin-1
IPI01021553.1	CFB			X	extracellular	metabolic process,regulation of biological process,response to stimulus	protein binding,catalytic activity	86 kDa protein
IPI00786946.1	LOC642249			X	cytoskeleton	cell organization and biogenesis		similar to Tektin-3
IPI00020201.1	ST8SIA4			X	membrane,cytoplasm,Golgi	cell organization and biogenesis,development,metabolic process,response to stimulus,cell differentiation	catalytic activity	CMP-N-acetylneuraminate-poly-alpha-2,8-sialyltransferase
IPI00003168.1	PRPSAP2	X	X			metabolic process,regulation of biological process	metal ion binding,catalytic activity,enzyme regulator activity	Phosphoribosyl pyrophosphate synthase-associated protein 2
IPI00784773.19	IGKC			X			protein binding	Putative uncharacterized protein
IPI00004084.3	ATF6B			X	membrane,endoplasmic reticulum,cytoplasm,nucleus	metabolic process,regulation of biological process,response to stimulus,cell communication	protein binding,DNA binding	Isoform 2 of Cyclic AMP-dependent transcription factor ATF-6 beta
IPI00013454.4	RGS10		X		membrane,cytoplasm	metabolic process,regulation of biological process,response to stimulus,cell communication	enzyme regulator activity	Isoform 1 of Regulator of G-protein signaling 10
IPI00008202.1	HECA			X		development		Headcase protein homolog
IPI00247295.4	SYNE1		X		membrane		protein binding	Isoform 4 of Nesprin-1
IPI00293723.2	NXPH4			X	extracellular	regulation of biological process,response to stimulus,cell communication		Neurexophilin-4
IPI00026800.1	SCRG1			X	extracellular	development		Scrapie-responsive protein 1
IPI00395694.1	TNPO3	X	X		cytoplasm,organelle lumen,nucleus	transport	protein binding,receptor activity	Isoform 2 of Transportin-3
IPI00289802.3	CSMD2			X	membrane			Isoform 1 of CUB and sushi domain-containing protein 2
IPI00006524.5	KIAA0319			X	membrane,cytoplasm,endosome	development,cell organization and biogenesis,regulation of biological process,cellular component movement,cell differentiation	protein binding	Isoform 1 of Dyslexia-associated protein KIAA0319
IPI00399318.1	COPE	X	X		membrane,cytoplasm,Golgi	transport	structural molecule activity	coatomer subunit epsilon isoform b

IPI	GENE	Alzheimer's Hippocampus	Control hippocampus	CSF	Cellular localization	Biological process	Molecular function	Protein Description
IPI00218342.11	MTHFD1	X	X		membrane	metabolic process	nucleotide binding,catalytic activity	C-1-tetrahydrofolate synthase, cytoplasmic
IPI00103552.4	MUC16			X	extracellular,membrane,cytoplasm,Golgi,organelle lumen	metabolic process,reproduction	protein binding	Mucin-16
IPI00827745.1	RBM24			X	cytoplasm,nucleus	development,metabolic process,regulation of biological process,cell differentiation	RNA binding,nucleotide binding	Isoform 1 of RNA-binding protein 24
IPI00007019.1	PPIL1	X			spliceosomal complex,nucleus	metabolic process	catalytic activity	Peptidyl-prolyl cis-trans isomerase-like 1
IPI00253281.8	EPS8L1			X			protein binding	Isoform 2 of Epidermal growth factor receptor kinase substrate 8-like protein 1
IPI00339392.1	RIMS1		X				protein binding	Isoform 4 of Regulating synaptic membrane exocytosis protein 1
IPI00145805.1	TNIK			X	cytoskeleton,cytoplasm,organelle lumen,nucleus,endosome	cell organization and biogenesis,development,metabolic process,regulation of biological process,response to stimulus,cell communication,cell differentiation	protein binding,nucleotide binding,catalytic activity,enzyme regulator activity	Isoform 1 of TRAF2 and NCK-interacting protein kinase
IPI00382938.4	IGLV4-3			X				V5-1 protein
IPI00465315.6	CYCS	X	X	X	mitochondrion,membrane,cytoplasm,cytosol,nucleus	cell death,cell organization and biogenesis,transport,metabolic process,regulation of biological process	protein binding,metal ion binding,catalytic activity	Cytochrome c
IPI00064739.2	THEM4		X		membrane,mitochondrion,cytoplasm,cytosol	regulation of biological process,response to stimulus,cell communication		Thioesterase superfamily member 4
IPI00217143.4	SDHA	X	X			metabolic process	catalytic activity	Uncharacterized protein
IPI00645452.1	TUBB	X	X		cytoskeleton	cell organization and biogenesis,metabolic process,cellular component movement	nucleotide binding,structural molecule activity,catalytic activity	Uncharacterized protein
IPI00383951.7	SDK1			X	membrane		protein binding	Uncharacterized protein
IPI00329389.8	RPL6	X	X		cytoplasm,ribosome,cytosol	cell organization and biogenesis,transport,metabolic process,regulation of biological process,reproduction	DNA binding,RNA binding,structural molecule activity	60S ribosomal protein L6
IPI00020178.1	STAM	X	X		membrane,cytoplasm,cytosol,endosome	cell organization and biogenesis,transport,regulation of biological process,response to stimulus,cell communication	protein binding	Isoform 1 of Signal transducing adapter molecule 1
IPI00015029.1	PTGES3	X	X		cytoplasm,organelle lumen,chromosome,cytosol,nucleus	cell organization and biogenesis,metabolic process,regulation of biological process,response to stimulus,cell communication	protein binding,catalytic activity	Prostaglandin E synthase 3
IPI00220070.5	PFKFB4			X	cytoplasm,cytosol	metabolic process	protein binding,nucleotide binding,catalytic activity	6-phosphofructo-2-kinase/fructose-2,6-biphosphatase 4
IPI00152701.7	PLCD3	X	X		membrane,cytoplasm	cell proliferation,development,metabolic process,regulation of biological process,response to stimulus,cell communication	protein binding,signal transducer activity,metal ion binding,catalytic activity	1-phosphatidylinositol-4,5-bisphosphate phosphodiesterase delta-3
IPI00293434.2	SRP14	X	X		cytoplasm,cytosol	metabolic process,transport,regulation of biological process,response to stimulus	RNA binding	Signal recognition particle 14 kDa protein
IPI00176920.7	NPHP4			X	cytoskeleton,membrane,cytoplasm,cytosol	development,cell organization and biogenesis,regulation of biological process,response to stimulus,cell communication	protein binding,structural molecule activity	Nephrocystin-4
IPI00251507.2	SYN1		X	X	cytoplasm	transport,cell communication		Isoform IB of Synapsin-1
IPI00028520.2	NDUFV1	X	X	X	membrane,mitochondrion,cytoplasm	transport,metabolic process	metal ion binding,nucleotide binding,catalytic activity	Isoform 1 of NADH dehydrogenase [ubiquinone] flavoprotein 1, mitochondrial
IPI00215746.3	FABP4			X	cytoplasm,nucleus	transport,metabolic process,regulation of biological process,response to stimulus,defense response,cell differentiation	transporter activity	Fatty acid-binding protein, adipocyte
IPI00423683.1	EMID1			X			protein binding	Isoform 2 of EMI domain-containing protein 1
IPI00003965.5	USP7		X		cytoplasm,organelle lumen,nucleus,cytosol	cell death,development,metabolic process,regulation of biological process,response to stimulus	protein binding,catalytic activity	Ubiquitin carboxyl-terminal hydrolase 7
IPI00165528.2	USP47		X			metabolic process	catalytic activity	Isoform 2 of Ubiquitin carboxyl-terminal hydrolase 47
IPI00015983.2	S1PR3			X	membrane	cell proliferation,regulation of biological process,response to stimulus,defense response,cell communication,cellular homeostasis	signal transducer activity,receptor activity	Sphingosine 1-phosphate receptor 3
IPI00006547.4	ITFG1	X	X		extracellular,membrane			T-cell immunomodulatory protein
IPI00027507.1	CFHR3			X	extracellular			Complement factor H-related protein 3
IPI00642244.2	EMC1	X	X					Isoform 4 of Uncharacterized protein KIAA0090
IPI00014424.1	EEF1A2	X	X		cytoplasm,nucleus	cell death,metabolic process,regulation of biological process,response to stimulus	RNA binding,nucleotide binding,catalytic activity	Elongation factor 1-alpha 2

IPI	GENE	Alzheimer's Hippocampus	Control hippocampus	CSF	Cellular localization	Biological process	Molecular function	Protein Description
IPI00784808.1	Sep-09	X	X				nucleotide binding,structural molecule activity,catalytic activity	septin-9 isoform b
IPI00000023.4	GABRG2	X	X		membrane	transport,regulation of biological process,response to stimulus,cell communication	signal transducer activity,protein binding,transporter activity,receptor activity	Isoform 1 of Gamma-aminobutyric acid receptor subunit gamma-2
IPI00337741.4	APEH	X	X		membrane,cytoplasm,nucleus	metabolic process	catalytic activity	Acylamino-acid-releasing enzyme
IPI00419221.4	MBOAT2			X	membrane	metabolic process	catalytic activity	Lysophospholipid acyltransferase 2
IPI00385449.5	PRKCA	X	X		membrane,mitochondrion,endoplasmic reticulum,cytoplasm,organelle lumen,nucleus,cytosol	cell death,cell proliferation,cell organization and biogenesis,development,metabolic process,transport,regulation of biological process,response to stimulus,cellular component movement,cell communication,cell differentiation,coagulation	protein binding,metal ion binding,nucleotide binding,catalytic activity	Protein kinase C alpha type
IPI00216184.3	PICALM	X	X					Isoform 2 of Phosphatidylinositol-binding clathrin assembly protein
IPI00413781.4	CXCL12			X	extracellular	response to stimulus	protein binding	Isoform Gamma of Stromal cell-derived factor 1
IPI00032903.3	PTRH2		X		mitochondrion,cytoplasm	cell death	catalytic activity	Peptidyl-tRNA hydrolase 2, mitochondrial
IPI00748788.2	MTM1		X		membrane,cytoplasm,endosome	cell organization and biogenesis,metabolic process,transport,regulation of biological process	protein binding,catalytic activity	Myotubularin
IPI00216070.3	MYL1			X	cytoskeleton,cytoplasm,cytosol	development,cellular component movement	metal ion binding,structural molecule activity	Isoform MLC1 of Myosin light chain 1/3, skeletal muscle isoform
IPI00399089.4	MESDC2		X	X	membrane,endoplasmic reticulum,cytoplasm	metabolic process,regulation of biological process,response to stimulus,cell communication	protein binding	LDLR chaperone MESD
IPI00237884.4	AKAP12	X	X		cytoskeleton,membrane,cytoplasm	metabolic process,transport,regulation of biological process,response to stimulus,cell communication	protein binding	Isoform 1 of A-kinase anchor protein 12
IPI00008418.6	DIABLO	X	X		mitochondrion,cytoplasm	cell death,metabolic process,regulation of biological process		Diablo homolog, mitochondrial precursor
IPI00029561.1	NDUFA10	X	X		mitochondrion,membrane,cytoplasm,organelle lumen	transport,metabolic process,response to stimulus	nucleotide binding,catalytic activity	NADH dehydrogenase [ubiquinone] 1 alpha subcomplex subunit 10, mitochondrial
IPI00001506.1	NPY			X	extracellular	cell proliferation,cell organization and biogenesis,development,transport,regulation of biological process,response to stimulus,cellular component movement,cell communication,cell differentiation	signal transducer activity,protein binding,receptor activity	Pro-neuropeptide Y
IPI00003831.4	ATP2B3	X	X		membrane	metabolic process,transport,response to stimulus,coagulation	protein binding,transporter activity,metal ion binding,nucleotide binding,catalytic activity	Isoform XB of Plasma membrane calcium-transporting ATPase 3
IPI00411680.10	PCMT1			X	cytoplasm	metabolic process	catalytic activity	Protein-L-isoaspartate (D-aspartate) O-methyltransferase
IPI00411733.4	DHX30		X		mitochondrion,cytoplasm,organelle lumen		protein binding,RNA binding,nucleotide binding,catalytic activity	Isoform 1 of Putative ATP-dependent RNA helicase DHX30
IPI00012048.1	NME1	X	X	X	cytoskeleton,membrane,mitochondrion,cytoplasm,cytosol,nucleus	cell death,cell proliferation,development,transport,metabolic process,regulation of biological process,response to stimulus,cell differentiation,reproduction	protein binding,metal ion binding,nucleotide binding,catalytic activity	Isoform 1 of Nucleoside diphosphate kinase A
IPI00414896.1	RNASET2	X	X	X	extracellular	metabolic process	RNA binding,catalytic activity	Isoform 1 of Ribonuclease T2
IPI00019924.2	TBCC	X	X		cytoskeleton,cytoplasm	metabolic process	protein binding,catalytic activity	Tubulin-specific chaperone C
IPI00030328.1	SRR	X	X		membrane,cytoplasm	development,cell organization and biogenesis,metabolic process,response to stimulus	protein binding,metal ion binding,nucleotide binding,catalytic activity	Serine racemase
IPI00016339.4	RAB5C	X	X		membrane,cytoplasm,nucleus,endosome	cell organization and biogenesis,metabolic process,transport,regulation of biological process,response to stimulus,cell communication	nucleotide binding,catalytic activity	Ras-related protein Rab-5C
IPI00017339.1	SF3B4	X			spliceosomal complex,organelle lumen,nucleus	metabolic process	protein binding,RNA binding,nucleotide binding	Splicing factor 3B subunit 4
IPI00023407.4	NCKAP1L			X	membrane,cytoplasm,cytosol	cell death,cell proliferation,development,cell organization and biogenesis,metabolic process,transport,regulation of biological process,response to stimulus,cellular component movement,cell communication,cell differentiation	protein binding,enzyme regulator activity	Nck-associated protein 1-like
IPI00013216.1	ORC2			X	organelle lumen,chromosome,nucleus	metabolic process,regulation of biological process	protein binding,DNA binding	Origin recognition complex subunit 2
IPI00299095.2	SNX2	X	X		membrane,cytoplasm,endosome	transport,cell communication	protein binding,transporter activity	Sorting nexin-2

IPI	GENE	Alzheimer's Hippocampus	Control hippocampus	CSF	Cellular localization	Biological process	Molecular function	Protein Description
IPI00027851.2	HEXA			X		metabolic process	catalytic activity	cDNA FLJ53927, highly similar to Beta-hexosaminidase alpha chain
IPI00169115.2	OR13C3			X	membrane	regulation of biological process,response to stimulus,cell communication	signal transducer activity,receptor activity	Olfactory receptor 13C3
IPI00166039.2	SHISA5			X	membrane,endoplasmic reticulum,cytoplasm,nucleus	cell death,regulation of biological process,response to stimulus,cell communication	signal transducer activity,protein binding	Isoform 1 of Protein shisa-5
IPI00005198.2	ILF2	X	X		cytoplasm,organelle lumen,nucleus	metabolic process,regulation of biological process,response to stimulus	protein binding,DNA binding,RNA binding,nucleotide binding,catalytic activity	Interleukin enhancer-binding factor 2
IPI00293361.5	SGSM2			X		metabolic process,regulation of biological process	enzyme regulator activity	Isoform 2 of Small G protein signaling modulator 2
IPI00293793.4	SLC30A3	X	X		membrane,cytoplasm,endosome	transport,regulation of biological process,cellular homeostasis	transporter activity	Zinc transporter 3
IPI00787050.1	NPTX1			X	extracellular			similar to neuronal pentraxin I precursor
IPI00910084.1	GALNT7			X			catalytic activity	cDNA FLJ57113, highly similar to N-acetylgalactosaminyltransferase 7
IPI00293128.3	EXT1			X	membrane,endoplasmic reticulum,cytoplasm,Golgi	cell organization and biogenesis,development,metabolic process,regulation of biological process,response to stimulus,cell communication,cell differentiation	protein binding,catalytic activity	Exostosin-1
IPI00643115.1	STMN1			X		regulation of biological process,response to stimulus,cell communication		Stathmin
IPI00410013.4	ZC3H3			X	nucleus	transport,metabolic process,regulation of biological process	metal ion binding	Isoform 1 of Zinc finger CCCH domain-containing protein 3
IPI00019169.3	SH3GL1	X	X		membrane,cytoplasm,endosome	development,transport,regulation of biological process,response to stimulus,cell communication	protein binding	endophilin-A2 isoform 1
IPI00021257.1	ARFIP2	X	X		membrane,cytoplasm	cell organization and biogenesis,regulation of biological process,response to stimulus,cellular component movement,cell communication	protein binding,nucleotide binding	Arfaptin-2
IPI00451625.1	CRTAC1			X			metal ion binding	Isoform 2 of Cartilage acidic protein 1
IPI00410693.4	SERBP1		X		cytoplasm			SERPINE1 mRNA binding protein 1, isoform CRA_d
IPI00337541.3	NNT	X	X		mitochondrion,membrane,cytoplasm	metabolic process,transport	transporter activity,nucleotide binding,catalytic activity	NAD(P) transhydrogenase, mitochondrial
IPI00385003.1	TIGD1			X			DNA binding	Putative transposase
IPI00002925.1	CARTPT			X	extracellular,cytoplasm	development,transport,metabolic process,regulation of biological process,response to stimulus,cell communication,cellular homeostasis,cell differentiation	protein binding	Cocaine- and amphetamine-regulated transcript protein
IPI00010343.3	SLC8A2	X	X	X	cytoskeleton,membrane,mitochondrion,cytoplasm	development,transport,response to stimulus,cell communication,cellular homeostasis,coagulation	protein binding,transporter activity	Sodium/calcium exchanger 2
IPI00218493.7	HPRT1	X	X	X	cytoplasm,cytosol	cell death,cell proliferation,cell organization and biogenesis,development,metabolic process,regulation of biological process,response to stimulus,reproduction,cell differentiation	protein binding,metal ion binding,nucleotide binding,catalytic activity	Hypoxanthine-guanine phosphoribosyltransferase
IPI00446588.1	PLXNA4			X			protein binding	Isoform 2 of Plexin-A4
IPI00015952.2	EIF4G2		X			metabolic process	protein binding,DNA binding,RNA binding	cDNA FLJ59571, highly similar to Eukaryotic translation initiation factor 4gamma 2
IPI00759479.2	IQSEC2		X		cytoplasm,nucleus	cell organization and biogenesis,transport,regulation of biological process,response to stimulus,cell communication	protein binding,enzyme regulator activity	Isoform 1 of IQ motif and SEC7 domain-containing protein 2
IPI00030706.1	AHSA1	X	X	X	endoplasmic reticulum,cytoplasm,cytosol	metabolic process,regulation of biological process,response to stimulus	protein binding,enzyme regulator activity	Activator of 90 kDa heat shock protein ATPase homolog 1
IPI00166865.3	CISD2	X	X	X	membrane,mitochondrion,endoplasmic reticulum,cytoplasm	development,metabolic process,regulation of biological process	protein binding,metal ion binding	CDGSH iron-sulfur domain-containing protein 2
IPI00292304.2	FRRS1L			X	membrane			Uncharacterized protein C9orf4
IPI00003566.3	HOMER1		X		cytoskeleton,membrane,cytoplasm	development,transport,regulation of biological process,response to stimulus,cell communication,cell differentiation	protein binding	Isoform 1 of Homer protein homolog 1
IPI00010369.2	TEX15			X		cell organization and biogenesis,development,metabolic process,regulation of biological process,response to stimulus,reproduction		Testis expressed sequence 15, isoform CRA_a
IPI00032063.7	LRP1B			X			protein binding,metal ion binding	Low-density lipoprotein receptor-related protein 1B precursor
IPI00015602.1	TOMM70A	X	X		membrane,mitochondrion,cytoplasm	cell organization and biogenesis,transport,metabolic process	protein binding,transporter activity	Mitochondrial import receptor subunit TOM70

IPI	GENE	Alzheimer's Hippocampus	Control hippocampus	CSF	Cellular localization	Biological process	Molecular function	Protein Description
IPI00292496.1	TUBB8			X	cytoskeleton,cytoplasm	cell organization and biogenesis,metabolic process,cellular component movement	nucleotide binding,structural molecule activity,catalytic activity	Tubulin beta-8 chain
IPI00025874.2	RPN1	X	X		membrane,endoplasmic reticulum,cytoplasm	metabolic process	catalytic activity	Dolichyl-diphosphooligosaccharide--protein glycosyltransferase subunit 1 precursor
IPI00020407.1	MGAT5			X	membrane,cytoplasm,Golgi	metabolic process	catalytic activity	Alpha-1,6-mannosylglycoprotein 6-beta-N-acetylglucosaminyltransferase A
IPI00215780.5	RPS19	X	X		cytoplasm,ribosome,organelle lumen,cytosol,nucleus	development,cell organization and biogenesis,transport,metabolic process,regulation of biological process,response to stimulus,cellular component movement,cell differentiation,reproduction	protein binding,RNA binding,structural molecule activity	40S ribosomal protein S19
IPI00018968.2	NAE1	X	X		membrane,cytoplasm	cell death,metabolic process,regulation of biological process,response to stimulus,cell communication	protein binding,nucleotide binding,catalytic activity	NEDD8-activating enzyme E1 regulatory subunit
IPI00023315.1	GDF10			X	extracellular	development,regulation of biological process,response to stimulus,cell communication,reproduction,cell differentiation	protein binding	Bone morphogenetic protein 3B
IPI00551062.2	CNPY3	X	X		endoplasmic reticulum,cytoplasm,organelle lumen	response to stimulus,defense response	protein binding	Isoform 1 of Protein canopy homolog 3
IPI00022648.2	EIF5	X	X		cytoplasm,cytosol	metabolic process,regulation of biological process	protein binding,RNA binding,nucleotide binding,catalytic activity	Eukaryotic translation initiation factor 5
IPI00152850.2	JAM3	X	X	X				Junctional adhesion molecule 3 precursor
IPI00172636.5	CAMK2D	X	X	X		metabolic process	protein binding,nucleotide binding,catalytic activity	Isoform Delta 6 of Calcium/calmodulin-dependent protein kinase type II subunit delta
IPI00217882.3	SORT1	X	X	X	cell surface,membrane,endoplasmic reticulum,cytoplasm,Golgi,vacuole,nucleus,endosome	cell death,cell organization and biogenesis,development,transport,metabolic process,regulation of biological process,response to stimulus,cell communication,cell differentiation	protein binding,signal transducer activity,receptor activity	Sortilin
IPI00215884.4	SRSF1	X	X		spliceosomal complex,cytoplasm,organelle lumen,nucleus	development,cell organization and biogenesis,transport,metabolic process	protein binding,RNA binding,nucleotide binding	Isoform ASF-1 of Serine/arginine-rich splicing factor 1
IPI00335589.5	RNMTL1			X	mitochondrion,cytoplasm	metabolic process	RNA binding,catalytic activity	RNA methyltransferase-like protein 1
IPI00294713.5	MASP2			X	extracellular	metabolic process,regulation of biological process,response to stimulus,defense response	protein binding,metal ion binding,catalytic activity	Isoform 1 of Mannan-binding lectin serine protease 2
IPI00215983.3	CA1	X	X	X	cytoplasm,Golgi	metabolic process	metal ion binding,catalytic activity	Carbonic anhydrase 1
IPI00026031.3	GINM1			X	membrane			Uncharacterized protein C6orf72
IPI00179851.3	C11orf9			X	membrane,nucleus	development,metabolic process,regulation of biological process,cell communication,cellular homeostasis,cell differentiation	DNA binding	Isoform 1 of Myelin gene regulatory factor
IPI00384280.5	PCYOX1	X	X		extracellular,membrane,cytoplasm,vacuole	metabolic process,transport	transporter activity,catalytic activity	Prenylcysteine oxidase 1
IPI00103891.1	MTMR14			X		metabolic process	catalytic activity	Putative uncharacterized protein
IPI00040900.5	HS2ST1			X	membrane		catalytic activity	Uncharacterized protein
IPI00293126.1	TBCB	X	X		cytoskeleton,cytoplasm,organelle lumen,nucleus	development,metabolic process,cell differentiation	protein binding	Tubulin-folding cofactor B
IPI00301923.4	CDK9			X	cytoplasm,organelle lumen,nucleus	cell proliferation,cell organization and biogenesis,metabolic process,regulation of biological process,response to stimulus,reproduction	protein binding,DNA binding,RNA binding,nucleotide binding,catalytic activity	Isoform 1 of Cyclin-dependent kinase 9
IPI00065276.2	ASPSCR1	X	X	X			protein binding	Isoform 2 of Tether containing UBX domain for GLUT4
IPI00220334.3	SEZ6L			X				Isoform 3 of Seizure 6-like protein
IPI00167560.2	PAPD4			X	cytoplasm,nucleus	metabolic process	metal ion binding,nucleotide binding,catalytic activity	Isoform 1 of Poly(A) RNA polymerase GLD2
IPI00016910.1	EIF3C		X		cytoplasm,cytosol	metabolic process	protein binding,RNA binding	Eukaryotic translation initiation factor 3 subunit C
IPI00019359.4	KRT9		X	X	cytoskeleton,cytoplasm	cell organization and biogenesis,development,reproduction	structural molecule activity	Keratin, type I cytoskeletal 9
IPI00297160.4	CD44	X	X	X				Isoform 12 of CD44 antigen
IPI00143753.6	U2SURP			X	organelle lumen,nucleus	metabolic process	protein binding,RNA binding,nucleotide binding	Isoform 1 of U2 snRNP-associated SURP motif-containing protein
IPI00007240.2	F13B			X	extracellular	response to stimulus,coagulation		Coagulation factor XIII B chain

IPI	GENE	Alzheimer's Hippocampus	Control hippocampus	CSF	Cellular localization	Biological process	Molecular function	Protein Description
IPI00029273.2	MET			X	extracellular,membrane	cell proliferation,development,cell organization and biogenesis,metabolic process,regulation of biological process,response to stimulus,cell communication,cell differentiation	protein binding,signal transducer activity,nucleotide binding,receptor activity,catalytic activity	Isoform 1 of Hepatocyte growth factor receptor
IPI00470535.2	CACNA2D1	X	X	X			protein binding	Uncharacterized protein
IPI00005705.1	PPP1CC	X	X	X	mitochondrion,membrane,cytoplasm,organelle lumen,chromosome,cytosol,nucleus	cell organization and biogenesis,cell division,metabolic process,transport,regulation of biological process	protein binding,metal ion binding,catalytic activity	Isoform Gamma-1 of Serine/threonine-protein phosphatase PP1-gamma catalytic subunit
IPI00026516.1	OXCT1	X	X	X	mitochondrion,cytoplasm,organelle lumen	development,transport,metabolic process,regulation of biological process,response to stimulus,cell communication,cellular homeostasis	protein binding,catalytic activity	Succinyl-CoA:3-ketoacid-coenzyme A transferase 1, mitochondrial
IPI00010415.2	ACOT7	X	X		mitochondrion,cytoplasm,cytosol	metabolic process	catalytic activity	Isoform 1 of Cytosolic acyl coenzyme A thioester hydrolase
IPI00942902.1	GDA	X	X	X	cytoplasm,cytosol	development,metabolic process	metal ion binding,catalytic activity	Guanine deaminase
IPI00790536.1	BLOC1S6	X				cell organization and biogenesis,transport		hCG2001986, isoform CRA_g
IPI00018305.4	IGFBP3			X	extracellular,nucleus	cell death,cell proliferation,cell organization and biogenesis,development,metabolic process,regulation of biological process,response to stimulus,cellular component movement,cell communication,cell growth,cell differentiation	protein binding,metal ion binding,enzyme regulator activity	Insulin-like growth factor-binding protein 3
IPI00024880.3	SCUBE2			X	extracellular		protein binding,metal ion binding	Isoform 1 of Signal peptide, CUB and EGF-like domain-containing protein 2
IPI00030362.1	PLP2	X			membrane,endoplasmic reticulum,cytoplasm	transport,regulation of biological process,response to stimulus,cell communication	protein binding,transporter activity	Isoform 1 of Proteolipid protein 2
IPI00015964.4	GAP43			X	membrane	cell organization and biogenesis,development,regulation of biological process,response to stimulus,cell communication,cell differentiation	protein binding	Neuromodulin
IPI00024403.1	CPNE3	X	X		cytoplasm,cytosol	transport,metabolic process	protein binding,transporter activity,catalytic activity	Copine-3
IPI00219889.3	UQCC	X	X					Isoform 2 of Ubiquinol-cytochrome c reductase complex chaperone CBP3 homolog
IPI00029372.2	HAUS3			X	cytoskeleton,cytoplasm	cell organization and biogenesis,cell division		HAUS augmin-like complex subunit 3
IPI00420071.1	MAP6	X	X	X	cytoskeleton,membrane,cytoplasm,Golgi	cell organization and biogenesis,regulation of biological process,reproduction	protein binding	Isoform 1 of Microtubule-associated protein 6
IPI00295851.4	COPB1	X	X		membrane,cytoplasm,Golgi,cytosol	cell organization and biogenesis,transport	protein binding,structural molecule activity	Coatomer subunit beta
IPI00219861.3	ACP1	X	X		membrane,cytoplasm,nucleus	metabolic process	protein binding,catalytic activity	Isoform 1 of Low molecular weight phosphotyrosine protein phosphatase
IPI00374065.3	MIA3			X	membrane		protein binding	similar to melanoma inhibitory activity 3 isoform 1
IPI00064767.3	ARHGAP17	X			membrane,cytoplasm,cytosol	cell organization and biogenesis,transport,metabolic process,regulation of biological process,response to stimulus,cell communication	protein binding,enzyme regulator activity	Isoform 1 of Rho GTPase-activating protein 17
IPI00289113.4	LRSAM1	X	X				protein binding	Isoform 2 of E3 ubiquitin-protein ligase LRSAM1
IPI00788258.1	LOXL1			X		metabolic process	metal ion binding,catalytic activity	similar to lysyl oxidase-like 1 preproprotein
IPI00102543.4	SLITRK1			X	membrane	development,cell organization and biogenesis,cell differentiation	protein binding	SLIT and NTRK-like protein 1
IPI00304693.6	AGFG1	X	X			metabolic process,regulation of biological process	metal ion binding,enzyme regulator activity	Isoform 2 of Arf-GAP domain and FG repeats-containing protein 1
IPI00297251.3	MGRN1			X				Isoform 2 of E3 ubiquitin-protein ligase MGRN1
IPI00013299.4	C1orf151-NBL1			X	extracellular	regulation of biological process	protein binding	C1ORF15-NBL1 read-through protein isoform b
IPI00157144.2	GYS1	X	X			metabolic process	catalytic activity	glycogen [starch] synthase, muscle isoform 2
IPI00031768.1	HOOK3		X		cytoskeleton,membrane,cytoplasm,Golgi,vacuole	cell proliferation,cell organization and biogenesis,development,transport,regulation of biological process,cell differentiation	protein binding,transporter activity,motor activity,catalytic activity	Protein Hook homolog 3
IPI00295386.7	SETD4	X	X	X	cytoplasm	metabolic process	nucleotide binding,catalytic activity	Carbonyl reductase [NADPH] 1
IPI00552578.2	SAA1			X	extracellular	response to stimulus,defense response		Serum amyloid A protein
IPI00442294.3	NTM	X	X	X			protein binding	cDNA FLJ57258, highly similar to Neurotrimin

IPI	GENE	Alzheimer's Hippocampus	Control hippocampus	CSF	Cellular localization	Biological process	Molecular function	Protein Description
IPI00022463.2	TF	X	X	X	extracellular,membrane,mitochondrion,cytoplasm,endosome	transport,response to stimulus,cellular homeostasis,coagulation	protein binding,metal ion binding	Serotransferrin
IPI00746777.3	ADH5	X	X		mitochondrion,cytoplasm,nucleus	metabolic process,response to stimulus	protein binding,metal ion binding,nucleotide binding,catalytic activity	Alcohol dehydrogenase class-3
IPI00248596.9	ELFN1			X			protein binding	extracellular leucine-rich repeat and fibronectin type-III domain-containing protein 1
IPI00167941.1	MDN1			X	organelle lumen,nucleus	cell organization and biogenesis,metabolic process	protein binding,nucleotide binding,catalytic activity	Midasin
IPI00166044.1	RPTOR	X	X		cytoplasm,vacuole,cytosol	cell proliferation,cell organization and biogenesis,metabolic process,regulation of biological process,response to stimulus,cell communication,cell growth	protein binding,DNA binding	Isoform 1 of Regulatory-associated protein of mTOR
IPI00014376.5	RAB31	X	X		cytoplasm,nucleus	transport,regulation of biological process,response to stimulus,cell communication	nucleotide binding,catalytic activity	ras-related protein Rab-31
IPI00306159.8	MECR	X	X		mitochondrion,cytoplasm	metabolic process	protein binding,metal ion binding,nucleotide binding,catalytic activity	Isoform 1 of Trans-2-enoyl-CoA reductase, mitochondrial
IPI00328268.10	EIF4G3		X			metabolic process,regulation of biological process	protein binding,DNA binding,RNA binding,enzyme regulator activity	eukaryotic translation initiation factor 4 gamma 3 isoform 1
IPI00328650.5	C14orf159	X	X		mitochondrion,cytoplasm			Isoform 1 of UPF0317 protein C14orf159, mitochondrial
IPI00177937.6	MICAL3		X		cytoskeleton,membrane,cytoplasm	cell organization and biogenesis,metabolic process,transport	protein binding,metal ion binding,catalytic activity	Isoform 1 of Protein MICAL-3
IPI00374563.3	AGRN	X	X	X	extracellular,cell surface,membrane	cell death,cell organization and biogenesis,development,transport,metabolic process,regulation of biological process,response to stimulus,cell communication,cell differentiation	protein binding,transporter activity,structural molecule activity	Agrin
IPI00024933.3	RPL12	X	X		cytoplasm,ribosome,cytosol	cell organization and biogenesis,transport,metabolic process,reproduction	RNA binding,structural molecule activity	Isoform 1 of 60S ribosomal protein L12
IPI00011201.1	ME2	X	X		mitochondrion,cytoplasm,organelle lumen	metabolic process	metal ion binding,nucleotide binding,catalytic activity	NAD-dependent malic enzyme, mitochondrial
IPI00902906.1	PTPRN2		X			metabolic process	catalytic activity	Isoform 3 of Receptor-type tyrosine-protein phosphatase N2
IPI00654755.3	HBB	X	X	X	cytoplasm,cytosol	cell death,cell organization and biogenesis,transport,metabolic process,regulation of biological process,response to stimulus,coagulation	antioxidant activity,protein binding,transporter activity,metal ion binding,catalytic activity	Hemoglobin subunit beta
IPI00006900.1	UTP3			X	organelle lumen,nucleus	development,cell organization and biogenesis,metabolic process,regulation of biological process		Something about silencing protein 10
IPI00385980.9	ROBO2			X	cell surface,membrane	cell death,cell organization and biogenesis,development,regulation of biological process,response to stimulus,cell communication,cell differentiation,reproduction	signal transducer activity,protein binding,receptor activity	Isoform 1 of Roundabout homolog 2
IPI00290462.5	CBR3	X	X		cytoplasm,organelle lumen,cytosol,nucleus	metabolic process	nucleotide binding,catalytic activity	Carbonyl reductase [NADPH] 3
IPI00014624.2	AP3S1	X	X					AP-3 complex subunit sigma-1
IPI00219465.5	TCN2			X	extracellular	metabolic process,transport	metal ion binding	Transcobalamin-2
IPI00090327.1	VPS45	X	X		membrane,cytoplasm,Golgi,endosome	transport,response to stimulus,coagulation		Vacuolar protein sorting-associated protein 45
IPI00015947.5	DNAJB1		X		cytoplasm,organelle lumen,nucleus	metabolic process,response to stimulus	protein binding	DnaJ homolog subfamily B member 1
IPI00555614.1	HSP90AB3P			X	cytoplasm	metabolic process,response to stimulus	protein binding,nucleotide binding	Putative heat shock protein HSP 90-beta-3
IPI00032313.1	S100A4			X	cytoplasm,nucleus	development,cell organization and biogenesis,regulation of biological process,response to stimulus,cell communication,cell differentiation	protein binding,metal ion binding	Protein S100-A4
IPI00328128.6	GNAZ	X	X		membrane,endoplasmic reticulum,cytoplasm,nucleus	metabolic process,regulation of biological process,response to stimulus,cell communication	signal transducer activity,protein binding,metal ion binding,nucleotide binding,catalytic activity	Guanine nucleotide-binding protein G(z) subunit alpha
IPI00410210.1	LPHN1			X	membrane	regulation of biological process,response to stimulus,cell communication	protein binding,signal transducer activity,receptor activity	Isoform 2 of Latrophilin-1
IPI00220342.5	DDAH1	X	X	X	mitochondrion,cytoplasm	development,metabolic process,regulation of biological process,response to stimulus,cell communication	metal ion binding,catalytic activity	N(G),N(G)-dimethylarginine dimethylaminohydrolase 1
IPI00023919.4	PSMC5	X	X		proteasome,cytoplasm,organelle lumen,nucleus,cytosol	cell death,metabolic process,regulation of biological process,response to stimulus,cell communication,reproduction	protein binding,nucleotide binding,catalytic activity	26S protease regulatory subunit 8
IPI00217963.3	KRT16			X	cytoskeleton	cell proliferation,development,cell organization and biogenesis	protein binding,structural molecule activity	Keratin, type I cytoskeletal 16

IPI	GENE	Alzheimer's Hippocampus	Control hippocampus	CSF	Cellular localization	Biological process	Molecular function	Protein Description
IPI00305010.4	CPPED1	X	X				metal ion binding,catalytic activity	Isoform 1 of Calcineurin-like phosphoesterase domain-containing protein 1
IPI00164066.4	CCDC136			X	membrane			Isoform 4 of Coiled-coil domain-containing protein 136
IPI00219930.7	CRABP1		X	X	cytoplasm	development,transport,regulation of biological process,response to stimulus,cell communication	transporter activity	Cellular retinoic acid-binding protein 1
IPI00180128.4	BZW1	X	X				protein binding	Isoform 2 of Basic leucine zipper and W2 domain-containing protein 1
IPI00007755.3	RAB21	X	X		membrane,endoplasmic reticulum,cytoplasm,Golgi,endosome	metabolic process,transport,regulation of biological process,response to stimulus,cell communication	protein binding,nucleotide binding,catalytic activity	Ras-related protein Rab-21
IPI00028561.1	KIF5C	X	X	X	cytoskeleton,cytoplasm	development,cell organization and biogenesis,response to stimulus,cellular component movement,cell differentiation	protein binding,motor activity,nucleotide binding,catalytic activity	Isoform 1 of Kinesin heavy chain isoform 5C
IPI00007627.1	KCNJ6		X		cell surface,membrane,cytoplasm,Golgi	transport,regulation of biological process,response to stimulus,cell communication	protein binding,transporter activity	G protein-activated inward rectifier potassium channel 2
IPI00333776.6	NRCAM	X	X	X	cell surface,membrane	development,cell organization and biogenesis,regulation of biological process,response to stimulus,cellular component movement,cell communication,cell growth,cell differentiation	protein binding	Isoform 1 of Neuronal cell adhesion molecule
IPI00025647.1	FBXO21		X	X			protein binding,DNA binding	Isoform 2 of F-box only protein 21
IPI00328389.1	NPM2	X			cytoplasm,organelle lumen,chromosome,nucleus	development,cell organization and biogenesis,regulation of biological process,reproduction,cell differentiation	protein binding	Nucleoplasmin-2
IPI00299588.2	ABI2	X	X		cytoplasm		protein binding	Uncharacterized protein
IPI00440828.1	FAHD1	X	X			metabolic process	catalytic activity	Isoform 2 of Fumarylacetoacetate hydrolase domain-containing protein 1
IPI00045360.4	CIC			X	nucleus	metabolic process,regulation of biological process	protein binding,DNA binding	Protein capicua homolog
IPI00000105.4	MVP	X	X		membrane,cytoplasm,nucleus	transport,response to stimulus	protein binding	Major vault protein
IPI00748890.1	LUZP2			X	extracellular			Isoform 1 of Leucine zipper protein 2
IPI00009396.1	CNR1	X	X	X	membrane	cell death,development,cell organization and biogenesis,metabolic process,transport,regulation of biological process,response to stimulus,defense response,cell communication,cellular homeostasis,reproduction,cell differentiation	signal transducer activity,receptor activity	Isoform 1 of Cannabinoid receptor 1
IPI00031461.2	GDI2			X		transport	enzyme regulator activity	cDNA FLJ60299, highly similar to Rab GDP dissociation inhibitor beta
IPI00010865.1	CSNK2B	X	X		membrane,cytoplasm,cytosol,nucleus	cell proliferation,development,cell organization and biogenesis,metabolic process,regulation of biological process,response to stimulus,cellular component movement,cell communication,cell differentiation	protein binding,metal ion binding,catalytic activity,enzyme regulator activity	Casein kinase II subunit beta
IPI00031008.2	TNC	X	X	X	extracellular	cell proliferation,cell organization and biogenesis,development,metabolic process,regulation of biological process,response to stimulus,cell communication,cell differentiation,reproduction	protein binding	Isoform 1 of Tenascin
IPI00018352.1	UCHL1	X	X	X	membrane,endoplasmic reticulum,cytoplasm,organelle lumen,nucleus	cell death,metabolic process,regulation of biological process,response to stimulus,cell communication	protein binding,catalytic activity	Ubiquitin carboxyl-terminal hydrolase isozyme L1
IPI00008511.2	ND5		X		mitochondrion,membrane,cytoplasm	metabolic process,transport,response to stimulus	catalytic activity	NADH-ubiquinone oxidoreductase chain 5
IPI00301364.3	SKP1	X	X	X	cytoplasm,organelle lumen,cytosol,nucleus	cell organization and biogenesis,metabolic process,regulation of biological process,response to stimulus,cell communication	protein binding,catalytic activity	Isoform 1 of S-phase kinase-associated protein 1
IPI00401264.5	ERP44		X	X	membrane,endoplasmic reticulum,cytoplasm,organelle lumen	metabolic process,regulation of biological process,response to stimulus,cellular homeostasis	metal ion binding,catalytic activity	Endoplasmic reticulum resident protein 44
IPI00023858.2	FCGR3B			X				30 kDa protein
IPI00297487.2	CTSH	X		X	extracellular,cytoplasm,vacuole,cytosol	cell death,cell proliferation,development,metabolic process,regulation of biological process,response to stimulus,cellular component movement,cell communication	protein binding,signal transducer activity,receptor activity,catalytic activity,enzyme regulator activity	Pro-cathepsin H
IPI00014832.3	PDK2	X	X				nucleotide binding	51 kDa protein
IPI00006181.1	EIF3D	X	X		cytoplasm,cytosol	cell organization and biogenesis,metabolic process	protein binding,RNA binding	Eukaryotic translation initiation factor 3 subunit D
IPI00299000.5	PA2G4	X	X		cytoplasm,organelle lumen,nucleus	cell proliferation,metabolic process,regulation of biological process	protein binding,DNA binding,RNA binding	Proliferation-associated protein 2G4

IPI	GENE	Alzheimer's Hippocampus	Control hippocampus	CSF	Cellular localization	Biological process	Molecular function	Protein Description
IPI00879665.1	SEZ6L			X				Isoform 6 of Seizure 6-like protein
IPI00157454.2	HS6ST2	X		X	membrane		catalytic activity	Isoform 1 of Heparan-sulfate 6-O-sulfotransferase 2
IPI00305551.3	GNA11	X	X		membrane,cytoplasm	development,metabolic process,regulation of biological process,response to stimulus,cell communication,cellular homeostasis,cell differentiation,coagulation	signal transducer activity,protein binding,metal ion binding,nucleotide binding,catalytic activity	Guanine nucleotide-binding protein subunit alpha-11
IPI00010796.1	P4HB	X	X	X	extracellular,cell surface,membrane,endoplasmic reticulum,cytoplasm,organelle lumen	metabolic process,regulation of biological process,cellular homeostasis	protein binding,metal ion binding,catalytic activity	Protein disulfide-isomerase
IPI00384297.3	CBR4	X	X		mitochondrion,cytoplasm,organelle lumen	cell organization and biogenesis,metabolic process	protein binding,nucleotide binding,catalytic activity	Isoform 1 of Carbonyl reductase family member 4
IPI00783874.2	HDHD2	X	X			metabolic process	metal ion binding,catalytic activity	Isoform 1 of Haloacid dehalogenase-like hydrolase domain-containing protein 2
IPI00871941.2	NBR1			X	cytoplasm,vacuole,cytosol,endosome	cell organization and biogenesis,development,metabolic process,regulation of biological process,response to stimulus,cell communication,cell differentiation	protein binding,metal ion binding	Isoform 1 of Next to BRCA1 gene 1 protein
IPI00394655.6	NFASC	X	X	X			protein binding	Isoform 4 of Neurofascin
IPI00645242.2	LOC643669			X				hypothetical protein LOC643669
IPI00470838.2	DENND2C			X	membrane			Isoform 1 of DENN domain-containing protein 2C
IPI00010895.4	TULP2			X	extracellular,cytoplasm			Tubby-related protein 2
IPI00013415.1	RPS7	X	X		cytoskeleton,cytoplasm,ribosome,organelle lumen,cytosol,nucleus	cell organization and biogenesis,metabolic process,transport,reproduction	protein binding,RNA binding,structural molecule activity	40S ribosomal protein S7
IPI00873924.2	IL18BP			X	extracellular	regulation of biological process,response to stimulus,cell communication	protein binding	Isoform A of Interleukin-18-binding protein
IPI00009881.3	GNAS			X	extracellular,cytoplasm	transport,reproduction		Neuroendocrine secretory protein 55
IPI00220907.5	PKIA	X	X		cytoplasm,nucleus	metabolic process,transport,regulation of biological process	enzyme regulator activity	cAMP-dependent protein kinase inhibitor alpha
IPI00010157.1	MAT2A	X	X		cytoplasm,cytosol	metabolic process,response to stimulus	metal ion binding,nucleotide binding,catalytic activity	S-adenosylmethionine synthase isoform type-2
IPI00000949.1	CRYM	X	X		membrane,cytoplasm,organelle lumen,nucleus	metabolic process,transport,regulation of biological process	protein binding,nucleotide binding,catalytic activity	Mu-crystallin homolog
IPI00005292.1	SPOCK1			X	extracellular,cytoskeleton,cytoplasm	cell organization and biogenesis,development,metabolic process,regulation of biological process,response to stimulus,cellular component movement,cell communication,cell differentiation,cell growth	protein binding,metal ion binding,enzyme regulator activity	Testican-1
IPI00019346.1	RAP2A	X	X		membrane,cytoplasm,cytosol,endosome	cell organization and biogenesis,development,metabolic process,transport,regulation of biological process,response to stimulus,cellular component movement,cell communication,cell differentiation	protein binding,transporter activity,nucleotide binding,catalytic activity	Ras-related protein Rap-2a
IPI00019244.1	NOVA1		X		nucleus	metabolic process,response to stimulus,cell communication	RNA binding	Isoform 1 of RNA-binding protein Nova-1
IPI00219684.3	FABP3	X	X	X	cytoplasm,cytosol	cell proliferation,metabolic process,transport,regulation of biological process,response to stimulus	protein binding,transporter activity	Fatty acid-binding protein, heart
IPI00028600.1	KLK7			X	extracellular	development,metabolic process	catalytic activity	Isoform 1 of Kallikrein-7
IPI00300631.3	SAFB	X			organelle lumen,nucleus	cell organization and biogenesis,metabolic process,regulation of biological process,response to stimulus,cell communication	protein binding,DNA binding,RNA binding,nucleotide binding	Scaffold attachment factor B1
IPI00978208.1	LOC256374			X		metabolic process	catalytic activity	peptidyl-prolyl cis-trans isomerase A-like
IPI00419836.1	DCBLD2			X	cell surface,membrane	cell organization and biogenesis,regulation of biological process,response to stimulus,cell communication,cell growth		Isoform 1 of Discoidin, CUB and LCCL domain-containing protein 2
IPI00183206.7	RIMBP2		X	X	membrane	metabolic process,regulation of biological process	protein binding	Isoform 1 of RIMS-binding protein 2
IPI00290332.2	ARHGAP10		X		membrane,cytoplasm,cytosol	cell death,cell organization and biogenesis,metabolic process,regulation of biological process,response to stimulus,cell communication	protein binding,enzyme regulator activity	Rho GTPase-activating protein 10
IPI00007102.3	GLOD4	X	X	X			catalytic activity	CGI-150 protein
IPI00376689.1	KIAA1199			X	cytoplasm			Isoform 1 of Protein KIAA1199

IPI	GENE	Alzheimer's Hippocampus	Control hippocampus	CSF	Cellular localization	Biological process	Molecular function	Protein Description
IPI00005732.1	ACVR1B			X	cell surface,membrane	cell death,development,cell organization and biogenesis,metabolic process,regulation of biological process,response to stimulus,cell communication,cell differentiation,cell growth	protein binding,signal transducer activity,metal ion binding,nucleotide binding,receptor activity,catalytic activity	Isoform 1 of Activin receptor type-1B
IPI00020987.1	PRELP		X	X	extracellular	development	protein binding,structural molecule activity	Prolargin
IPI00298961.3	XPO1	X	X		membrane,cytoplasm,organelle lumen,chromosome,cytosol,nucleus	cell organization and biogenesis,metabolic process,transport,reproduction	protein binding,transporter activity,RNA binding	Exportin-1
IPI00031765.1	PCDHGC4			X	membrane		metal ion binding	Isoform 2 of Protocadherin gamma-C4
IPI00745300.2	NAA40			X			catalytic activity	31 kDa protein
IPI00166817.4	ZNF561			X	nucleus	metabolic process,regulation of biological process	DNA binding,metal ion binding	Zinc finger protein 561
IPI00071059.4	BAX		X			cell death,regulation of biological process		Isoform Epsilon of Apoptosis regulator BAX
IPI00169259.1	SVIP	X	X	X	membrane,endoplasmic reticulum,cytoplasm,Golgi			Small VCP/p97-interacting protein
IPI00168806.3	MLL3			X	organelle lumen,nucleus	cell organization and biogenesis,metabolic process,regulation of biological process,response to stimulus,cell communication	protein binding,DNA binding,metal ion binding,catalytic activity	Isoform 1 of Histone-lysine N-methyltransferase MLL3
IPI00024034.2	CDH4	X		X	membrane	development,cell organization and biogenesis,regulation of biological process,response to stimulus,cell differentiation,cell growth	metal ion binding	Cadherin-4
IPI00550917.3	TWF2	X	X	X	cytoskeleton,cytoplasm	development,cell organization and biogenesis,regulation of biological process,response to stimulus,cell communication,cell differentiation,cell growth	protein binding,nucleotide binding	Twinfilin-2
IPI00339217.3	OVCH1	X			extracellular	metabolic process	metal ion binding,catalytic activity	Ovochymase-1
IPI01008917.1	IGHD			X			protein binding	FLJ00382 protein
IPI00170924.2	HINT3	X	X		mitochondrion,cytoplasm,organelle lumen,nucleus	metabolic process	catalytic activity	Histidine triad nucleotide-binding protein 3
IPI00002495.1	EPN1	X	X		membrane	cell organization and biogenesis,transport		EH domain-binding mitotic phosphoprotein
IPI00007010.3	LYZL6			X	extracellular	metabolic process	catalytic activity	Lysozyme-like protein 6
IPI00243221.2	NRD1			X	mitochondrion,cytoplasm	metabolic process	metal ion binding,catalytic activity	nardilysin isoform a
IPI00000871.4	PRL			X	extracellular,cytoplasm	cell proliferation,development,transport,regulation of biological process,response to stimulus,cell communication,reproduction	protein binding	Prolactin
IPI00165092.3	YARS2		X		mitochondrion,cytoplasm,organelle lumen	cell organization and biogenesis,metabolic process	protein binding,RNA binding,nucleotide binding,catalytic activity	Tyrosyl-tRNA synthetase, mitochondrial
IPI00014456.4	STRN		X		cytoskeleton,membrane,cytoplasm	cell proliferation,cell organization and biogenesis,development,regulation of biological process,response to stimulus,cell communication,cell differentiation	protein binding	Isoform 1 of Striatin
IPI00028160.1	HMBS	X			cytoplasm,chromosome,cytosol,nucleus	development,metabolic process,response to stimulus,cell differentiation	catalytic activity	Isoform 1 of Porphobilinogen deaminase
IPI00029534.1	PPAT	X	X		cytoplasm,cytosol	development,cell organization and biogenesis,transport,metabolic process,response to stimulus,reproduction	metal ion binding,catalytic activity	Amidophosphoribosyltransferase
IPI00940990.1	DKK3			X	extracellular	development,regulation of biological process,response to stimulus,cell communication	enzyme regulator activity	dickkopf homolog 3 (Xenopus laevis), isoform CRA_b
IPI00383046.3	CMBL	X	X		cytoplasm,cytosol	metabolic process	catalytic activity	Carboxymethylenebutenolidase homolog
IPI00030781.1	STAT1		X		cytoplasm,organelle lumen,cytosol,nucleus	cell death,cell proliferation,development,cell organization and biogenesis,metabolic process,regulation of biological process,response to stimulus,cellular component movement,defense response,cell communication,cell differentiation	signal transducer activity,protein binding,DNA binding,metal ion binding,catalytic activity	Isoform Alpha of Signal transducer and activator of transcription 1-alpha/beta
IPI00006957.3	DHRS7	X	X			metabolic process	nucleotide binding,catalytic activity	Isoform 1 of Dehydrogenase/reductase SDR family member 7
IPI00004247.2	PHKA1	X			membrane,cytoplasm,cytosol	metabolic process	protein binding,catalytic activity	Isoform 1 of Phosphorylase b kinase regulatory subunit alpha, skeletal muscle isoform
IPI01018924.1	FBXO2	X	X		membrane,endoplasmic reticulum,cytoplasm,cytosol	metabolic process,regulation of biological process	protein binding,catalytic activity	F-box only protein 2
IPI00221106.5	SF3B2	X			spliceosomal complex,organelle lumen,nucleus	metabolic process		Splicing factor 3B subunit 2

IPI	GENE	Alzheimer's Hippocampus	Control hippocampus	CSF	Cellular localization	Biological process	Molecular function	Protein Description
IPI00302927.6	CCT4	X	X		cytoskeleton,cytoplasm,organelle lumen,cytosol,nucleus	metabolic process	protein binding,nucleotide binding	T-complex protein 1 subunit delta
IPI00784231.2	GPX1	X	X			metabolic process,response to stimulus	antioxidant activity,catalytic activity	16 kDa protein
IPI00000861.1	LASP1	X	X		cytoskeleton,membrane,cytoplasm	transport	protein binding,transporter activity,metal ion binding	Isoform 1 of LIM and SH3 domain protein 1
IPI00024175.3	PSMA7	X	X		proteasome,cytoplasm,organelle lumen,nucleus,cytosol	cell death,metabolic process,regulation of biological process,response to stimulus,cell communication	protein binding,catalytic activity	Isoform 1 of Proteasome subunit alpha type-7
IPI00006631.6	SV2B	X	X		membrane,cytoplasm	transport	transporter activity,catalytic activity	Synaptic vesicle glycoprotein 2B
IPI00554676.2	HBG2			X	cytoplasm,cytosol	transport,response to stimulus,coagulation	transporter activity,metal ion binding	Hemoglobin subunit gamma-2
IPI00290856.4	LYVE1			X	membrane	transport,metabolic process,response to stimulus,cellular component movement	signal transducer activity,receptor activity	Lymphatic vessel endothelial hyaluronic acid receptor 1
IPI00411674.7	ZNF254			X	nucleus	metabolic process,regulation of biological process	DNA binding,metal ion binding	Isoform 1 of Zinc finger protein 254
IPI00554811.2	ARPC4	X	X		cytoskeleton,cytoplasm	cell organization and biogenesis,regulation of biological process	protein binding	Actin-related protein 2/3 complex subunit 4
IPI00237671.9	NEFL	X	X		cytoskeleton,cytoplasm,cytosol	cell organization and biogenesis,transport,cellular component movement,cell communication	protein binding,motor activity,structural molecule activity,catalytic activity	Neurofilament light polypeptide
IPI00973204.2	IGLC3			X			protein binding	IGL@ protein
IPI00166776.7	CREG2			X	extracellular,endoplasmic reticulum,cytoplasm,Golgi	metabolic process	nucleotide binding,catalytic activity	Protein CREG2
IPI00007935.6	PDLIM5	X	X		cytoskeleton,membrane,cytoplasm,cytosol	development,cell organization and biogenesis,regulation of biological process,cell communication,cell differentiation	protein binding,metal ion binding	Isoform 1 of PDZ and LIM domain protein 5
IPI00022254.4	ATG3		X		cytoplasm,cytosol	cell death,cell organization and biogenesis,transport,metabolic process,response to stimulus,cell communication	protein binding,catalytic activity	Isoform 1 of Ubiquitin-like-conjugating enzyme ATG3
IPI00747849.2	ATP1B1	X	X	X	membrane	transport,metabolic process,response to stimulus,cellular component movement,coagulation	protein binding,transporter activity,catalytic activity	Isoform 1 of Sodium/potassium-transporting ATPase subunit beta-1
IPI00027626.3	CCT6A	X	X	X	cytoskeleton,membrane,cytoplasm,cytosol	metabolic process	protein binding,nucleotide binding	T-complex protein 1 subunit zeta
IPI00555600.1	IDUA			X		metabolic process	catalytic activity	Solute carrier family 26, member 1 isoform a variant (Fragment)
IPI00215790.6	RPL38	X	X		cytoplasm,ribosome,cytosol	cell organization and biogenesis,metabolic process,transport,reproduction	RNA binding,structural molecule activity	60S ribosomal protein L38
IPI00939627.1	CYAT1			X			protein binding	25 kDa protein
IPI00016613.2	CSNK2A1	X	X			metabolic process	nucleotide binding,catalytic activity	Uncharacterized protein
IPI00000860.2	FMOD			X	extracellular	development,cell organization and biogenesis,regulation of biological process,response to stimulus,cell communication	protein binding	Fibromodulin
IPI00451429.1	NIF3L1			X				Uncharacterized protein
IPI00300990.5	CHTOP			X	organelle lumen,nucleus	metabolic process,regulation of biological process	RNA binding	Isoform 1 of Chromatin target of PRMT1 protein
IPI00000874.1	PRDX1	X	X	X	mitochondrion,cytoplasm,nucleus	cell proliferation,development,metabolic process,response to stimulus	antioxidant activity,protein binding,catalytic activity	Peroxiredoxin-1
IPI00022295.1	PF4V1			X	extracellular	response to stimulus	protein binding	Platelet factor 4 variant
IPI00028957.2	UBE4A	X	X			metabolic process	catalytic activity	Isoform 2 of Ubiquitin conjugation factor E4 A
IPI00015361.1	PFDN5	X	X		cytoplasm,cytosol,nucleus	metabolic process,regulation of biological process,response to stimulus,cell communication	protein binding	Prefoldin subunit 5
IPI00789398.3	LY75-CD302			X	membrane			Isoform 3 of Lymphocyte antigen 75
IPI00873959.4	MYO5A	X	X		extracellular,cytoskeleton,membrane,mitochondrion,cytoplasm,organelle lumen,chromosome,nucleus	cell organization and biogenesis,transport,metabolic process,reproduction	protein binding,nucleotide binding,motor activity,catalytic activity	Isoform 2 of Myosin-Va
IPI00012510.3	EMILIN2			X	extracellular		protein binding,structural molecule activity	EMILIN-2
IPI00290315.4	CHGA	X	X	X	extracellular,membrane,cytoplasm			Chromogranin-A
IPI00296913.1	NUDT5	X	X	X		metabolic process	metal ion binding,catalytic activity	ADP-sugar pyrophosphatase
IPI00022426.1	AMBP			X	extracellular,membrane	metabolic process,regulation of biological process,response to stimulus,cell communication,reproduction	protein binding,metal ion binding,enzyme regulator activity	Protein AMBP

IPI	GENE	Alzheimer's Hippocampus	Control hippocampus	CSF	Cellular localization	Biological process	Molecular function	Protein Description
IPI00394782.5	HDDC3	X	X				catalytic activity	Isoform 2 of Guanosine-3',5'-bis(diphosphate) 3'-pyrophosphohydrolase MESH1
IPI00000977.1	MAP3K11			X	cytoskeleton,cytoplasm	cell death,cell proliferation,metabolic process,regulation of biological process,response to stimulus,cell communication	protein binding,signal transducer activity,nucleotide binding,catalytic activity	Mitogen-activated protein kinase kinase kinase 11
IPI00103871.3	ROBO4			X	cell surface,membrane	development,regulation of biological process,cellular component movement,cell differentiation	protein binding,receptor activity	Isoform 1 of Roundabout homolog 4
IPI00011589.4	Sep-04	X	X				nucleotide binding,catalytic activity	Isoform 4 of Septin-4
IPI00395925.3	WWC3		X			metabolic process,regulation of biological process	protein binding	Isoform 1 of Protein WWC3
IPI00871467.2	L1CAM			X	cell surface,membrane	cell death,development,cell organization and biogenesis,regulation of biological process,response to stimulus,cellular component movement,cell communication,cell differentiation,coagulation	protein binding	Isoform 1 of Neural cell adhesion molecule L1
IPI00373823.2	CYP26C1			X	membrane,endoplasmic reticulum,cytoplasm	cell organization and biogenesis,development,metabolic process,regulation of biological process,response to stimulus,cell communication,cell differentiation	metal ion binding,catalytic activity	Cytochrome P450 26C1
IPI00161119.3	NFKBIB			X	cytoplasm,cytosol,nucleus	metabolic process,transport,regulation of biological process,response to stimulus,defense response,cell communication	protein binding,signal transducer activity	Isoform 1 of NF-kappa-B inhibitor beta
IPI00303105.3	SUMO1		X		membrane,cytoplasm,organelle lumen,nucleus	cell organization and biogenesis,metabolic process,regulation of biological process,response to stimulus,cell communication,defense response	protein binding,catalytic activity	Small ubiquitin-related modifier 1
IPI00023704.1	LPP	X			membrane,cytoplasm,nucleus	cell communication	protein binding,metal ion binding,structural molecule activity	Lipoma-preferred partner
IPI00171647.1	SIGLEC8			X	membrane	regulation of biological process,response to stimulus,cell communication	protein binding,signal transducer activity,receptor activity	Isoform 1 of Sialic acid-binding Ig-like lectin 8
IPI00029264.4	CYC1	X	X		membrane,mitochondrion,cytoplasm	metabolic process,transport,response to stimulus	metal ion binding	Cytochrome c1, heme protein, mitochondrial
IPI00003438.2	DNAJC8	X			organelle lumen,nucleus	metabolic process	protein binding	DnaJ homolog subfamily C member 8
IPI00019190.1	MYOC			X	extracellular,cytoskeleton,membrane,endoplasmic reticulum,cytoplasm,nucleus	transport,metabolic process	protein binding,motor activity,structural molecule activity,catalytic activity	Myocilin
IPI00026824.2	HMOX2	X	X			metabolic process	catalytic activity	Heme oxygenase 2
IPI00025465.2	OGN	X	X	X	endoplasmic reticulum,cytoplasm		protein binding	cDNA FLJ59205, highly similar to Mimecan
IPI00219661.2	PLP1	X	X		membrane	cell death,development,metabolic process,regulation of biological process,response to stimulus,cell communication,cellular homeostasis,cell differentiation	structural molecule activity	Isoform 1 of Myelin proteolipid protein
IPI00292290.1	EPB49	X	X		cytoskeleton,membrane,cytoplasm,nucleus	cell organization and biogenesis,regulation of biological process	protein binding	Isoform Long of Dematin
IPI01014563.1	FTL			X	cytoplasm,cytosol	cell death,cell organization and biogenesis,metabolic process,transport,cellular homeostasis	protein binding,metal ion binding,catalytic activity	Ferritin light chain
IPI00005592.1	PDE1B		X		cytoplasm,organelle lumen,nucleus,cytosol	cell death,development,metabolic process,regulation of biological process,response to stimulus,cell communication,cell differentiation,coagulation	protein binding,metal ion binding,catalytic activity	Isoform PDE1B1 of Calcium/calmodulin-dependent 3',5'-cyclic nucleotide phosphodiesterase 1B
IPI00424119.2	FZD3			X	membrane,cytoplasm	cell proliferation,development,cell organization and biogenesis,regulation of biological process,response to stimulus,cell communication,reproduction,cell differentiation	protein binding,signal transducer activity,receptor activity	Frizzled-3
IPI00169285.6	PLBD2		X	X	cytoplasm,vacuole,organelle lumen	metabolic process	catalytic activity	Putative phospholipase B-like 2
IPI00976937.1	LOC728307			X	membrane			hypothetical protein LOC728307
IPI00332155.2	GPRIN1	X	X		membrane	development,cell organization and biogenesis,cell differentiation	protein binding	Isoform 1 of G protein-regulated inducer of neurite outgrowth 1
IPI00877029.1	FGA			X	extracellular	cell organization and biogenesis,regulation of biological process,response to stimulus,cell communication,coagulation	protein binding	FGA protein
IPI00170796.1	VPS29	X	X		membrane,cytoplasm,endosome	transport	metal ion binding,catalytic activity	Isoform 1 of Vacuolar protein sorting-associated protein 29
IPI00329147.6	CCDC43		X					coiled-coil domain-containing protein 43 isoform 1
IPI00017562.1	LPHN2			X	membrane	regulation of biological process,response to stimulus,cell communication	protein binding,signal transducer activity,receptor activity	Isoform 2 of Latrophilin-2

IPI	GENE	Alzheimer's Hippocampus	Control hippocampus	CSF	Cellular localization	Biological process	Molecular function	Protein Description
IPI00396279.3	CLASP1		X		cytoskeleton,cytoplasm,Golgi,chromosome,cytosol	development,cell organization and biogenesis,cell division,regulation of biological process,response to stimulus,cell differentiation	protein binding	Isoform 1 of CLIP-associating protein 1
IPI00019345.1	RAP1A	X	X		membrane,cytoplasm,cytosol	metabolic process,transport,regulation of biological process,response to stimulus,cell communication,coagulation	protein binding,nucleotide binding,catalytic activity	Ras-related protein Rap-1A
IPI00081836.3	HIST1H2AH			X	chromosome,nucleus	cell organization and biogenesis,metabolic process	DNA binding	Histone H2A type 1-H
IPI00021453.1	PPAP2B	X	X		membrane,endoplasmic reticulum,cytoplasm,Golgi	development,metabolic process,regulation of biological process,response to stimulus,cellular component movement,cell communication,reproduction	protein binding,catalytic activity	Lipid phosphate phosphohydrolase 3
IPI00029123.3	EDNRB			X	membrane	development,metabolic process,regulation of biological process,response to stimulus,cellular component movement,cell communication,cell differentiation	signal transducer activity,receptor activity	Isoform A of Endothelin B receptor
IPI00296727.3	KIF2B			X	cytoskeleton,cytoplasm,organelle lumen,chromosome,cytosol,nucleus	cell organization and biogenesis,cell division,regulation of biological process,response to stimulus,cellular component movement,coagulation	motor activity,nucleotide binding,catalytic activity	Kinesin-like protein KIF2B
IPI00012948.3	HBEGF			X	extracellular,cell surface,membrane	cell proliferation,development,cell organization and biogenesis,metabolic process,regulation of biological process,response to stimulus,cellular component movement,cell communication,cell growth	protein binding,receptor activity	Proheparin-binding EGF-like growth factor
IPI00301503.8	TRA2B	X	X		nucleus	metabolic process,regulation of biological process	protein binding,RNA binding,nucleotide binding	Isoform 1 of Transformer-2 protein homolog beta
IPI00021985.4	TM9SF4		X	X	membrane			Transmembrane 9 superfamily member 4
IPI00028448.2	BAI3			X	membrane	development,regulation of biological process,response to stimulus,cell communication	signal transducer activity,receptor activity	Brain-specific angiogenesis inhibitor 3
IPI00016334.2	MCAM	X	X	X	cell surface,membrane	development,response to stimulus	protein binding	Isoform 1 of Cell surface glycoprotein MUC18
IPI00007257.4	CLSTN1	X	X	X	membrane		metal ion binding	Isoform 2 of Calsyntenin-1 (Fragment)
IPI00847670.1	PGAM1		X	X				Similar to Phosphoglycerate mutase 1
IPI00022420.3	RBP4			X	extracellular	cell proliferation,development,metabolic process,transport,regulation of biological process,response to stimulus,cell communication,reproduction	protein binding,transporter activity	Retinol-binding protein 4
IPI00159899.10	ANKFY1	X	X		membrane,cytoplasm,endosome		protein binding,metal ion binding	Isoform 1 of Ankyrin repeat and FYVE domain-containing protein 1
IPI00007166.3	IER3IP1	X	X		membrane,endoplasmic reticulum,cytoplasm,Golgi	cell death,regulation of biological process		Immediate early response 3-interacting protein 1
IPI00026105.1	SCP2		X		mitochondrion,cytoplasm,organelle lumen,nucleus	cell organization and biogenesis,transport,metabolic process	protein binding,catalytic activity	Isoform SCPx of Non-specific lipid-transfer protein
IPI00061977.1	IGHA1			X			protein binding	IGHA1 protein
IPI00101987.6	BABAM1	X	X		cytoplasm,nucleus	cell organization and biogenesis,metabolic process,regulation of biological process,response to stimulus	protein binding	Isoform 1 of BRISC and BRCA1-A complex member 1
IPI00796906.1	ABCF3			X	membrane			8 kDa protein
IPI00514676.1	MOG			X			protein binding	myelin-oligodendrocyte glycoprotein isoform beta2 precursor
IPI00021831.1	PRKAR1A	X	X	X	cytoplasm,cytosol	development,metabolic process,transport,regulation of biological process,response to stimulus,cell communication,coagulation	protein binding,nucleotide binding,catalytic activity,enzyme regulator activity	cAMP-dependent protein kinase type I-alpha regulatory subunit
IPI00003918.6	RPL4	X	X		cytoplasm,ribosome,organelle lumen,cytosol,nucleus	cell organization and biogenesis,metabolic process,transport,reproduction	RNA binding,structural molecule activity	60S ribosomal protein L4
IPI00019550.1	TSSC1	X	X				protein binding	Protein TSSC1 (Fragment)
IPI00012796.1	GAD2	X	X		membrane,cytoplasm,Golgi,cytosol	transport,metabolic process,response to stimulus,cell communication	protein binding,catalytic activity	Glutamate decarboxylase 2
IPI00013947.1	SYNGR3	X	X		membrane,cytoplasm	transport,regulation of biological process	protein binding	Synaptogyrin-3
IPI00009377.5	C3orf19			X				HSPC212
IPI00852633.1	RABL2B			X				16 kDa protein
IPI00043863.2	MAP4		X		cytoskeleton		protein binding	Uncharacterized protein

IPI	GENE	Alzheimer's Hippocampus	Control hippocampus	CSF	Cellular localization	Biological process	Molecular function	Protein Description
IPI00293925.2	FCN3			X	extracellular	metabolic process,regulation of biological process,response to stimulus,cell communication,defense response	protein binding	Isoform 1 of Ficolin-3
IPI00002818.1	KLK11			X	extracellular	metabolic process	catalytic activity	Isoform 1 of Kallikrein-11
IPI00006752.2	MAP4K4		X		cytoplasm	metabolic process,regulation of biological process,response to stimulus,cell communication	protein binding,nucleotide binding,catalytic activity,enzyme regulator activity	Isoform 1 of Mitogen-activated protein kinase kinase kinase kinase 4
IPI00299977.1	PHPT1	X	X		cytoplasm,cytosol	cell organization and biogenesis,metabolic process,regulation of biological process,response to stimulus,cellular component movement,cell communication	protein binding,catalytic activity	Isoform 1 of 14 kDa phosphohistidine phosphatase
IPI00297263.6	HEG1			X	extracellular,cell surface,membrane	cell organization and biogenesis,development,cell differentiation	protein binding,metal ion binding	Isoform 1 of Protein HEG homolog 1
IPI00152998.3	LRRC40	X	X				protein binding	Leucine-rich repeat-containing protein 40
IPI00296777.4	SPARCL1	X	X	X	extracellular	regulation of biological process,response to stimulus,cell communication	protein binding,metal ion binding	SPARC-like protein 1
IPI00514622.2	RANBP6		X	X	cytoplasm,nucleus	transport	protein binding	Ran-binding protein 6
IPI00550906.6	CSTF2T			X	nucleus	metabolic process	RNA binding,nucleotide binding	Cleavage stimulation factor subunit 2 tau variant
IPI00012269.3	MMRN1			X	extracellular,cytoplasm,organelle lumen	transport,response to stimulus,coagulation	protein binding	Isoform 1 of Multimerin-1
IPI00022895.8	A1BG		X	X	extracellular		protein binding	Isoform 1 of Alpha-1B-glycoprotein
IPI00176199.5	SOWAHA	X	X				protein binding	Ankyrin repeat domain-containing protein 43
IPI00299758.4	CHST12			X	membrane,cytoplasm,Golgi	metabolic process	nucleotide binding,catalytic activity	Carbohydrate sulfotransferase 12
IPI00642432.1	GSTM5		X			metabolic process	protein binding,catalytic activity	Uncharacterized protein
IPI00887175.1	LOC100131257	X					metal ion binding	similar to hCG19534
IPI00293590.6	MGLL	X	X			metabolic process	catalytic activity	monoglyceride lipase isoform 1
IPI00167215.6	HEPACAM	X	X	X	membrane,cytoplasm	regulation of biological process	protein binding	Isoform 1 of Hepatocyte cell adhesion molecule
IPI00018768.1	TSN	X	X		cytoplasm,nucleus	metabolic process	protein binding,DNA binding,RNA binding,catalytic activity	Translin
IPI00305383.1	UQCRC2	X	X		membrane,mitochondrion,cytoplasm	transport,metabolic process	protein binding,metal ion binding,catalytic activity	Cytochrome b-c1 complex subunit 2, mitochondrial
IPI00000816.1	YWHAE	X	X	X	cytoskeleton,mitochondrion,cytoplasm,cytosol	cell death,development,transport,metabolic process,regulation of biological process,response to stimulus,cellular component movement,cell communication,cell differentiation	protein binding	Isoform 1 of 14-3-3 protein epsilon
IPI00015525.2	MMRN2			X	extracellular,membrane	transport	protein binding	Multimerin-2
IPI00171856.1	DOHH	X			cytoplasm,cytosol	metabolic process	protein binding,metal ion binding,catalytic activity	Deoxyhypusine hydroxylase
IPI00006146.4	SAA2			X	extracellular	response to stimulus,defense response		serum amyloid A2 isoform a
IPI00220578.3	GNAI3	X	X	X	cytoskeleton,membrane,cytoplasm,Golgi	cell organization and biogenesis,metabolic process,cell division,transport,regulation of biological process,response to stimulus,cell communication,coagulation	protein binding,signal transducer activity,metal ion binding,nucleotide binding,catalytic activity	Guanine nucleotide-binding protein G(k) subunit alpha
IPI00414784.4	CD300A			X	membrane		protein binding,receptor activity	Isoform 1 of CMRF35-like molecule 8
IPI00043215.3	IGSF1	X		X	extracellular,membrane	metabolic process,regulation of biological process,response to stimulus,cell communication	protein binding,receptor activity	Isoform 1 of Immunoglobulin superfamily member 1
IPI00642017.1	IGHA2			X	membrane		protein binding	Putative uncharacterized protein DKFZp686C02218 (Fragment)
IPI00299402.1	PC	X	X		membrane,mitochondrion,cytoplasm,organelle lumen	metabolic process	metal ion binding,nucleotide binding,catalytic activity	Pyruvate carboxylase, mitochondrial
IPI00218487.3	GJA1	X	X	X	membrane,mitochondrion,endoplasmic reticulum,cytoplasm,Golgi,vacuole,cytosol,endosome	cell death,cell proliferation,development,cell organization and biogenesis,transport,metabolic process,regulation of biological process,response to stimulus,cellular component movement,cell communication,cell differentiation	signal transducer activity,protein binding,transporter activity	Gap junction alpha-1 protein
IPI00290826.1	FAM174A			X	membrane			Membrane protein FAM174A

IPI	GENE	Alzheimer's Hippocampus	Control hippocampus	CSF	Cellular localization	Biological process	Molecular function	Protein Description
IPI00382394.9	JMJD7	X	X				protein binding	JmjC domain-containing protein 7
IPI00878282.1	ALB			X	extracellular	transport		23 kDa protein
IPI00009070.1	HBS1L		X	X		metabolic process,regulation of biological process,response to stimulus,cell communication	RNA binding,nucleotide binding,catalytic activity	Isoform 1 of HBS1-like protein
IPI00909070.1	SCRN1		X			metabolic process	catalytic activity	cDNA FLJ51971, highly similar to Secernin-1
IPI00028786.5	PKD1			X	membrane	regulation of biological process,response to stimulus,cell communication	protein binding	Isoform 3 of Polycystin-1
IPI00026964.2	UQCRFS1	X	X		mitochondrion,membrane,cytoplasm	metabolic process,transport,response to stimulus	protein binding,transporter activity,metal ion binding,catalytic activity	Cytochrome b-c1 complex subunit Rieske, mitochondrial
IPI01011991.1	RIPK3		X				receptor activity,catalytic activity	Prothymosin a14
IPI00015568.1	KBTBD11	X	X				protein binding	Kelch repeat and BTB domain-containing protein 11
IPI00011252.1	C8A			X	extracellular,membrane	cell death,metabolic process,regulation of biological process,response to stimulus,defense response	protein binding	Complement component C8 alpha chain
IPI00021369.1	CRYAB	X	X		cytoplasm,nucleus	cell death,cell organization and biogenesis,transport,metabolic process,regulation of biological process	protein binding,structural molecule activity	Alpha-crystallin B chain
IPI00384697.2	ALB			X	extracellular			Isoform 2 of Serum albumin
IPI00030320.4	DDX6	X	X		cytoplasm,cytosol	cell organization and biogenesis,metabolic process	protein binding,RNA binding,nucleotide binding,catalytic activity	Probable ATP-dependent RNA helicase DDX6
IPI00256684.2	AP2A1	X	X		membrane,cytoplasm	transport		Isoform B of AP-2 complex subunit alpha-1
IPI00001872.3	PCDHGC3			X	membrane		metal ion binding	Isoform 1 of Protocadherin gamma-C3
IPI00001863.8	WIF1			X		regulation of biological process,cell differentiation	protein binding	cDNA FLJ54458, highly similar to Wnt inhibitory factor 1
IPI00020008.1	NEDD8	X	X	X	nucleus	metabolic process,regulation of biological process,response to stimulus	protein binding	NEDD8
IPI00012898.1	VAMP1	X	X		mitochondrion,membrane,cytoplasm	transport,cell communication	protein binding	Isoform 1 of Vesicle-associated membrane protein 1
IPI00011569.2	ACACA		X		mitochondrion,cytoplasm,cytosol	cell organization and biogenesis,metabolic process,regulation of biological process,response to stimulus	protein binding,metal ion binding,nucleotide binding,catalytic activity	Isoform 1 of Acetyl-CoA carboxylase 1
IPI00015161.1	PDE6D		X			metabolic process,regulation of biological process,response to stimulus	protein binding,catalytic activity,enzyme regulator activity	Retinal rod rhodopsin-sensitive cGMP 3',5'-cyclic phosphodiesterase subunit delta
IPI00030352.3	R3HCC1L			X				Conserved hypothetical protein
IPI00184997.4	DLGAP5			X		cell communication		disks large-associated protein 5 isoform b
IPI00022597.1	UBE2M	X	X			cell death,metabolic process,regulation of biological process	protein binding,nucleotide binding,catalytic activity	NEDD8-conjugating enzyme Ubc12
IPI00423570.3	KRAS	X	X		membrane	transport,metabolic process,regulation of biological process,response to stimulus,cell communication	transporter activity,nucleotide binding,catalytic activity	Isoform 2B of GTPase KRas
IPI00022389.1	CRP			X	extracellular	cell organization and biogenesis,transport,metabolic process,regulation of biological process,response to stimulus,defense response,cell differentiation	protein binding,metal ion binding	Isoform 1 of C-reactive protein
IPI00173346.4	PGM2L1	X	X		cytoplasm,cytosol	metabolic process	catalytic activity	Glucose 1,6-bisphosphate synthase
IPI00020983.2	KCNA1		X		membrane	cell organization and biogenesis,transport,regulation of biological process,cell communication	protein binding,transporter activity	Potassium voltage-gated channel subfamily A member 1
IPI00010442.4	FXYD1		X	X	membrane	transport,cellular homeostasis	transporter activity	Phospholemman
IPI00174442.2	FAM98A	X	X			cell organization and biogenesis,metabolic process,cellular component movement		Isoform 1 of Protein FAM98A
IPI00301098.2	DRAXIN			X	extracellular	cell organization and biogenesis,development,regulation of biological process,response to stimulus,cell communication,cell differentiation		Draxin
IPI00163391.7	METTL16			X		metabolic process	catalytic activity	Isoform 1 of Methyltransferase-like protein 16
IPI00020928.1	TFAM	X	X		mitochondrion,cytoplasm,organelle lumen	cell organization and biogenesis,metabolic process,regulation of biological process	protein binding,DNA binding	Transcription factor A, mitochondrial
IPI00216106.3	OLA1			X	membrane	transport	transporter activity,nucleotide binding	Isoform 3 of Obg-like ATPase 1

IPI	GENE	Alzheimer's Hippocampus	Control hippocampus	CSF	Cellular localization	Biological process	Molecular function	Protein Description
IPI00027239.1	TNFSF13			X	extracellular,membrane,cytoplasm,organelle lumen,cytosol,nucleus	cell proliferation,development,metabolic process,regulation of biological process,response to stimulus,cell communication	protein binding	Isoform Alpha of Tumor necrosis factor ligand superfamily member 13
IPI00552591.2	IGLV10-54			X				V1-20 protein
IPI00026544.4	GBP3	X					nucleotide binding,catalytic activity	64 kDa protein
IPI00297333.4	TBC1D24	X	X		cytoplasm	cell organization and biogenesis,development,metabolic process,regulation of biological process,cell differentiation	protein binding,enzyme regulator activity	Isoform 1 of TBC1 domain family member 24
IPI00165044.3	FAM198B			X				Isoform 2 of Protein FAM198B
IPI00383594.2	MUM1			X				PWWP domain-containing protein MUM1
IPI00026103.3	ACHE			X	extracellular,cell surface,membrane,endoplasmic reticulum,cytoplasm,Golgi,organelle lumen,nucleus	cell proliferation,development,cell organization and biogenesis,transport,metabolic process,regulation of biological process,response to stimulus,cell communication,cell differentiation	protein binding,catalytic activity	acetylcholinesterase isoform E4-E6 precursor
IPI00016577.2	CD22			X				CDNA: FLJ22814 fis, clone KAIA3004
IPI00402157.1	CBLN3			X	extracellular		protein binding	Cerebellin-3
IPI00790122.1	TNFSF13			X	membrane	response to stimulus	protein binding	hCG1991317, isoform CRA_c
IPI00298912.7	CORO2B	X	X		cytoskeleton,membrane,cytoplasm	cell organization and biogenesis	protein binding	Isoform 1 of Coronin-2B
IPI00301058.5	VASP			X	cytoskeleton,membrane,cytoplasm,cytosol	development,cell organization and biogenesis,regulation of biological process,response to stimulus,cell communication,cell differentiation	protein binding	Vasodilator-stimulated phosphoprotein
IPI00019988.1	SGSH			X	cytoplasm,vacuole	metabolic process	metal ion binding,catalytic activity	N-sulphoglucosamine sulphohydrolase
IPI00003384.1	CELSR1			X	membrane	development,cell organization and biogenesis,regulation of biological process,response to stimulus,cellular component movement,cell communication,cell differentiation	protein binding,signal transducer activity,metal ion binding,receptor activity	Isoform 1 of Cadherin EGF LAG seven-pass G-type receptor 1
IPI00170916.1	NECAP1	X	X		membrane,cytoplasm	transport		Isoform 1 of Adaptin ear-binding coat-associated protein 1
IPI00220503.9	DCTN2	X	X		cytoskeleton			Isoform 2 of Dynactin subunit 2
IPI00289870.6	PCDH7			X	membrane		metal ion binding	Isoform C of Protocadherin-7
IPI00217376.1	SCN4B			X	membrane	transport,regulation of biological process,cell communication,cellular homeostasis	protein binding,transporter activity	Isoform 1 of Sodium channel subunit beta-4
IPI00748490.1	PTGES3L-AARSD1		X		cytoplasm	metabolic process	nucleotide binding,catalytic activity	alanyl-tRNA editing protein Aarsd1 isoform 1
IPI00165394.3	TSPAN14	X			membrane			Isoform 2 of Tetraspanin-14
IPI00064607.3	MEGF10			X	membrane	cell proliferation,development,transport,regulation of biological process,cell differentiation	protein binding	Isoform 1 of Multiple epidermal growth factor-like domains protein 10
IPI00217023.2	MMAA			X			nucleotide binding,catalytic activity	Uncharacterized protein
IPI00023860.1	NAP1L1	X	X		cytoplasm,nucleus	cell proliferation,cell organization and biogenesis,metabolic process,regulation of biological process	protein binding	Nucleosome assembly protein 1-like 1
IPI00004413.1	TNFRSF21			X	membrane,cytoplasm	cell death,metabolic process,regulation of biological process,response to stimulus,cell communication	protein binding,receptor activity	Tumor necrosis factor receptor superfamily member 21
IPI00009607.1	RAP2C		X		membrane,cytoplasm,cytosol,endosome	metabolic process,transport,regulation of biological process,response to stimulus,cellular component movement,cell communication	transporter activity,nucleotide binding,catalytic activity	Ras-related protein Rap-2c
IPI00011635.3	BCL2L13	X	X		mitochondrion,membrane,cytoplasm,nucleus	cell death,metabolic process,regulation of biological process	enzyme regulator activity	Isoform 2 of Bcl-2-like protein 13
IPI00552771.3	IGLV3-16			X				V2-11 protein
IPI00012433.2	F8A3	X	X		nucleus		protein binding	Factor VIII intron 22 protein
IPI00027442.4	AARS	X	X		cytoplasm,cytosol	cell death,development,metabolic process,regulation of biological process,response to stimulus,cell communication	RNA binding,metal ion binding,nucleotide binding,catalytic activity	Alanyl-tRNA synthetase, cytoplasmic
IPI00013779.1	C16orf70	X	X				protein binding	Isoform 1 of UPF0183 protein C16orf70

IPI	GENE	Alzheimer's Hippocampus	Control hippocampus	CSF	Cellular localization	Biological process	Molecular function	Protein Description
IPI00829701.3	IGHV1-18			X				13 kDa protein
IPI00022256.3	AP2M1	X	X		membrane,mitochondrion,cytoplasm,vacuole,cytosol	cell organization and biogenesis,development,transport,regulation of biological process,response to stimulus,defense response,cell communication,cell differentiation	protein binding,transporter activity	Isoform 1 of AP-2 complex subunit mu
IPI00290142.5	CTPS1	X	X		cytoplasm,cytosol	metabolic process,response to stimulus	nucleotide binding,catalytic activity	CTP synthase 1
IPI00400983.3	KCTD21		X			cell organization and biogenesis	protein binding	BTB/POZ domain-containing protein KCTD21
IPI00151990.3	TXNDC15			X	membrane	regulation of biological process,cellular homeostasis		Isoform 1 of Thioredoxin domain-containing protein 15
IPI00478127.3	TCEAL3	X	X		nucleus	metabolic process,regulation of biological process		Transcription elongation factor A protein-like 3
IPI00168884.3	ATP6AP2			X	cell surface,membrane	transport,metabolic process,regulation of biological process,response to stimulus,cell communication	protein binding,transporter activity,receptor activity	Renin receptor
IPI00021167.3	PRKRA	X	X		cytoplasm	cell death,cell proliferation,development,metabolic process,regulation of biological process,response to stimulus	protein binding,RNA binding,enzyme regulator activity	Isoform 1 of Interferon-inducible double stranded RNA-dependent protein kinase activator A
IPI00644522.1	PNKP			X			protein binding	PNKP protein
IPI00025273.1	GART	X	X		cytoplasm,cytosol	development,metabolic process,response to stimulus	metal ion binding,nucleotide binding,catalytic activity	Isoform Long of Trifunctional purine biosynthetic protein adenosine-3
IPI00163851.6	EIF2AK4			X	cytoplasm,ribosome,cytosol	metabolic process,regulation of biological process,response to stimulus,cell communication	protein binding,RNA binding,nucleotide binding,catalytic activity	Isoform 1 of Eukaryotic translation initiation factor 2-alpha kinase 4
IPI00011865.2	PDGFD			X				Isoform 2 of Platelet-derived growth factor D
IPI00009923.1	P4HA1		X		mitochondrion,endoplasmic reticulum,cytoplasm,organelle lumen	cell organization and biogenesis,metabolic process	protein binding,metal ion binding,catalytic activity	Isoform 1 of Prolyl 4-hydroxylase subunit alpha-1
IPI00298860.6	LTF			X	extracellular	transport,cellular homeostasis	metal ion binding	cDNA FLJ78440, highly similar to Human lactoferrin
IPI00657883.3	ACSL6	X	X			metabolic process	catalytic activity	Isoform 1 of Long-chain-fatty-acid--CoA ligase 6
IPI00335280.3	RPE	X	X		cytoplasm,cytosol	metabolic process	protein binding,metal ion binding,catalytic activity	Isoform 1 of Ribulose-phosphate 3-epimerase
IPI00004433.1	CNTN6			X	membrane	cell organization and biogenesis,development,regulation of biological process,response to stimulus,cell communication,cell differentiation	protein binding	Contactin-6
IPI00028376.1	TIMM8A		X		mitochondrion,membrane,cytoplasm	development,cell organization and biogenesis,metabolic process,transport	metal ion binding	Mitochondrial import inner membrane translocase subunit Tim8 A
IPI00020019.1	ADIPOQ			X	extracellular,endoplasmic reticulum,cytoplasm	cell death,cell organization and biogenesis,development,metabolic process,regulation of biological process,response to stimulus,defense response,cell proliferation,transport,cellular component movement,cell communication,cellular homeostasis,cell differentiation	protein binding	Adiponectin
IPI00654777.2	EIF3F	X	X			metabolic process	protein binding,RNA binding	cDNA FLJ36192 fis, clone TESTI2027450, highly similar to Eukaryotic translation initiation factor 3 subunit 5
IPI00030887.1	TYRO3			X	membrane,endoplasmic reticulum,cytoplasm,nucleus	cell death,cell organization and biogenesis,development,metabolic process,regulation of biological process,response to stimulus,defense response,reproduction,transport,cellular component movement,cell communication,cellular homeostasis,cell differentiation,coagulation	protein binding,signal transducer activity,nucleotide binding,receptor activity,catalytic activity	Tyrosine-protein kinase receptor TYRO3
IPI00013988.2	ARHGAP5	X	X			regulation of biological process,response to stimulus,cell communication	nucleotide binding	Isoform 2 of Rho GTPase-activating protein 5
IPI00473014.5	DSTN		X		cytoskeleton,cytoplasm	cell organization and biogenesis,cell division,regulation of biological process,cellular component movement	protein binding	Destrin
IPI00007812.1	ATP6V1B2	X	X	X	membrane,cytoplasm,Golgi,cytosol	metabolic process,transport,regulation of biological process,response to stimulus,cell communication,cellular homeostasis	transporter activity,nucleotide binding,catalytic activity	V-type proton ATPase subunit B, brain isoform
IPI00016346.2	PROSC	X	X		mitochondrion,cytoplasm			Proline synthetase co-transcribed homolog (Bacterial), isoform CRA_b
IPI00443799.2	SLC38A10			X	membrane	transport		Isoform 1 of Putative sodium-coupled neutral amino acid transporter 10
IPI00025426.3	PZP			X	extracellular	metabolic process,regulation of biological process,reproduction	enzyme regulator activity	Isoform 1 of Pregnancy zone protein

IPI	GENE	Alzheimer's Hippocampus	Control hippocampus	CSF	Cellular localization	Biological process	Molecular function	Protein Description
IPI00645577.1	ROGDI	X	X		nucleus	cell proliferation,development,regulation of biological process		Protein rogdi homolog
IPI00030741.1	LCA5L			X	membrane	transport	transporter activity	Lebercilin-like protein
IPI00642329.2	MFN2	X	X		cytoskeleton,mitochondrion,membrane,cytoplasm,cytosol	cell proliferation,cell organization and biogenesis,development,transport,metabolic process,regulation of biological process,response to stimulus,cell communication,coagulation	protein binding,nucleotide binding,catalytic activity	Isoform 1 of Mitofusin-2
IPI00021088.1	KCNAB2	X	X		membrane,cytoplasm	transport,regulation of biological process,cell communication	transporter activity	Isoform 1 of Voltage-gated potassium channel subunit beta-2
IPI00930072.1	IGHG2			X	membrane		protein binding	Putative uncharacterized protein DKFZp686E23209
IPI00465142.2	KIAA0528		X				protein binding	Isoform 1 of Uncharacterized protein KIAA0528
IPI00015842.1	RCN1	X	X	X	endoplasmic reticulum,cytoplasm,organelle lumen	development	protein binding,metal ion binding	Reticulocalbin-1
IPI00010207.1	UFM1	X	X	X	cytoplasm,nucleus	metabolic process		Isoform 1 of Ubiquitin-fold modifier 1
IPI00252845.4	SYT9			X	membrane,cytoplasm	transport	protein binding,transporter activity	Uncharacterized protein
IPI00014516.2	CALD1	X	X	X	cytoskeleton,membrane,cytoplasm,cytosol	cell organization and biogenesis,cellular component movement	protein binding	Isoform 1 of Caldesmon
IPI00062266.6	SCRN2	X	X			metabolic process	catalytic activity	Secernin-2
IPI00445716.1	GFRA3			X	cell surface,membrane,cytoplasm	development,cell organization and biogenesis,regulation of biological process,response to stimulus,cellular component movement,cell communication,cell differentiation	protein binding,signal transducer activity,receptor activity	Isoform 1 of GDNF family receptor alpha-3
IPI00003021.1	ATP1A2	X	X	X	membrane,cytoplasm,endosome	transport,metabolic process,regulation of biological process,response to stimulus,cell communication,cellular homeostasis	transporter activity,metal ion binding,nucleotide binding,catalytic activity	Sodium/potassium-transporting ATPase subunit alpha-2
IPI00718821.1	C19orf55			X				Isoform 1 of Uncharacterized protein C19orf55
IPI00003944.1	DBT		X		cytoskeleton,mitochondrion,cytoplasm,organelle lumen	metabolic process	catalytic activity	Lipoamide acyltransferase component of branched-chain alpha-keto acid dehydrogenase complex, mitochondrial
IPI00026154.3	PRKCSH	X	X	X			protein binding,metal ion binding	cDNA FLJ59211, highly similar to Glucosidase 2 subunit beta
IPI00479722.2	PSME1	X	X	X	proteasome,cytoplasm,organelle lumen,cytosol,nucleus	cell death,metabolic process,regulation of biological process,response to stimulus,cell communication		Proteasome activator complex subunit 1
IPI00002335.1	HTT	X	X					huntingtin
IPI00013894.1	STIP1	X	X		cytoplasm,Golgi,nucleus	development,cell organization and biogenesis,response to stimulus,cell differentiation	protein binding	Stress-induced-phosphoprotein 1
IPI00043747.3	RFTN2	X	X		membrane	metabolic process	catalytic activity	Raftlin-2
IPI00002966.2	HSPA4	X	X	X	cytoplasm,organelle lumen,nucleus,cytosol	cell organization and biogenesis,transport,response to stimulus	nucleotide binding	Heat shock 70 kDa protein 4
IPI00386436.3	HOPX		X		nucleus	development,metabolic process,regulation of biological process,cell differentiation	DNA binding	Isoform 1 of Homeodomain-only protein
IPI00006154.1	CFHR2			X	extracellular			Isoform Long of Complement factor H-related protein 2
IPI00160552.4	TNR	X	X	X	extracellular,cell surface,membrane,cytoplasm,organelle lumen,nucleus	cell organization and biogenesis,development,regulation of biological process,response to stimulus,cellular component movement,cell communication,cell differentiation,cell growth	protein binding	Isoform 1 of Tenascin-R
IPI00019180.1	GPC5		X	X	extracellular,membrane		protein binding	Glypican-5
IPI00852979.1	KIAA1549L			X				Uncharacterized protein
IPI00008226.1	ISM2			X				thrombospondin, type I, domain containing 3, isoform CRA_d
IPI00941650.1	IGKC			X			protein binding	26 kDa protein
IPI00290893.5	USP12	X	X			metabolic process	protein binding,catalytic activity	Ubiquitin carboxyl-terminal hydrolase 12
IPI00106646.1	SDF4			X	membrane,cytoplasm,Golgi,organelle lumen,endosome	development,transport,response to stimulus,cell differentiation	protein binding,metal ion binding	Isoform 1 of 45 kDa calcium-binding protein
IPI00394781.4	C2CD2L		X					Isoform 2 of C2 domain-containing protein 2-like
IPI00456599.1	FAM220A			X	nucleus			STAT3-interacting protein as a repressor

IPI	GENE	Alzheimer's Hippocampus	Control hippocampus	CSF	Cellular localization	Biological process	Molecular function	Protein Description
IPI00291928.8	RAB14	X	X		membrane,endoplasmic reticulum,cytoplasm,Golgi,vacuole,cytosol,nucleus,endosome	development,metabolic process,transport,regulation of biological process,response to stimulus,cell communication	protein binding,nucleotide binding,catalytic activity	Ras-related protein Rab-14
IPI00016801.1	GLUD1	X	X		mitochondrion,cytoplasm,organelle lumen	transport,metabolic process,regulation of biological process,cell communication	protein binding,nucleotide binding,catalytic activity	Glutamate dehydrogenase 1, mitochondrial
IPI00017367.6	RDX	X	X		cytoskeleton,membrane,cytoplasm,Golgi,organelle lumen,nucleus		protein binding	Uncharacterized protein
IPI00177543.6	PAM	X	X	X		metabolic process	protein binding,metal ion binding,catalytic activity	Isoform 5 of Peptidyl-glycine alpha-amidating monooxygenase
IPI00375803.3	GON4L			X	cytoplasm,organelle lumen,nucleus	metabolic process,regulation of biological process	DNA binding	Isoform 1 of GON-4-like protein
IPI00003881.5	HNRNPF	X	X		spliceosomal complex,cytoplasm,organelle lumen,nucleus	metabolic process,regulation of biological process	protein binding,RNA binding,nucleotide binding	Heterogeneous nuclear ribonucleoprotein F
IPI00152542.4	DMXL2	X	X		membrane,cytoplasm		protein binding	DmX-like protein 2
IPI00003766.4	ETHE1		X		mitochondrion,cytoplasm,organelle lumen,nucleus	metabolic process	metal ion binding,catalytic activity	Protein ETHE1, mitochondrial
IPI00290158.6	MARK2	X	X			metabolic process	nucleotide binding,catalytic activity	Isoform 7 of Serine/threonine-protein kinase MARK2
IPI00299652.2	ADAM11		X	X	membrane	metabolic process,regulation of biological process,response to stimulus,cell communication	protein binding,metal ion binding,catalytic activity	Isoform Long of Disintegrin and metalloproteinase domain-containing protein 11
IPI00888758.1	DNM1	X	X				protein binding,nucleotide binding,catalytic activity	Isoform 4 of Dynamin-1
IPI00418262.5	ALDOC	X	X	X		metabolic process	catalytic activity	Fructose-bisphosphate aldolase
IPI00296678.4	CAMK2G	X	X			metabolic process	protein binding,nucleotide binding,catalytic activity	Isoform 5 of Calcium/calmodulin-dependent protein kinase type II subunit gamma
IPI00926256.1	SLC4A1			X	cytoskeleton,membrane,cytoplasm	transport,cellular homeostasis	protein binding,transporter activity	band 3 anion transport protein
IPI00024046.2	CDH13	X	X	X	membrane		metal ion binding	cDNA FLJ52398, highly similar to Cadherin-13
IPI00019642.1	CLIP2	X	X		extracellular,cytoskeleton,membrane,cytoplasm	development,cell organization and biogenesis,regulation of biological process,cellular component movement	protein binding,motor activity,catalytic activity	Isoform 1 of CAP-Gly domain-containing linker protein 2
IPI00017184.2	EHD1	X	X		membrane,cytoplasm,endosome	cell organization and biogenesis,transport,metabolic process,regulation of biological process,response to stimulus,coagulation	protein binding,metal ion binding,nucleotide binding,catalytic activity	EH domain-containing protein 1
IPI00021363.3	KDM5A			X	nucleus	metabolic process	protein binding,DNA binding,catalytic activity	Similar to Retinoblastoma binding protein 2 isoform 2
IPI00025365.1	EDN3			X	extracellular	cell proliferation,cell organization and biogenesis,development,transport,metabolic process,regulation of biological process,response to stimulus,cellular component movement,cell communication,cell differentiation	protein binding	Isoform Long of Endothelin-3
IPI00164861.3	KIF13A			X		cellular component movement	protein binding,nucleotide binding,motor activity,catalytic activity	Isoform 3 of Kinesin-like protein KIF13A
IPI00008167.1	ATP1B3	X	X		membrane,cytoplasm	transport,metabolic process,response to stimulus,cellular component movement,coagulation	transporter activity,catalytic activity	Sodium/potassium-transporting ATPase subunit beta-3
IPI00009030.1	LAMP2			X	membrane,cytoplasm,vacuole,endosome	transport,response to stimulus,coagulation		Isoform LAMP-2A of Lysosome-associated membrane glycoprotein 2
IPI00013621.4	THTPA	X	X		cytoplasm,organelle lumen,nucleus,cytosol	metabolic process	metal ion binding,catalytic activity	Thiamine-triphosphatase
IPI00007144.1	RPL26L1		X		cytoplasm,ribosome,cytosol	cell organization and biogenesis,transport,metabolic process,reproduction	structural molecule activity	60S ribosomal protein L26-like 1
IPI00044707.2	HAPLN4		X	X	extracellular		protein binding	Hyaluronan and proteoglycan link protein 4
IPI00853369.1	PLXNB2			X	membrane	cell proliferation,development,cell organization and biogenesis,metabolic process,regulation of biological process,response to stimulus,cellular component movement,cell communication,cell differentiation	signal transducer activity,protein binding,receptor activity,enzyme regulator activity	Plexin-B2
IPI00908318.4	OXR1	X	X			metabolic process		88 kDa protein
IPI00026087.1	BANF1	X			cytoplasm,organelle lumen,chromosome,cytosol,nucleus	metabolic process,response to stimulus,reproduction	DNA binding	Barrier-to-autointegration factor
IPI00375174.1	ANKS1B			X			protein binding	Ankyrin repeat and sterile alpha motif domain-containing protein 1B
IPI00033600.1	PPP1R7	X	X	X	cytoplasm,nucleus	metabolic process,regulation of biological process	protein binding,enzyme regulator activity	Isoform 1 of Protein phosphatase 1 regulatory subunit 7
IPI00025812.1	CA11			X	extracellular,membrane		catalytic activity	Carbonic anhydrase-related protein 11

IPI	GENE	Alzheimer's Hippocampus	Control hippocampus	CSF	Cellular localization	Biological process	Molecular function	Protein Description
IPI00845354.1	IGKC			X			protein binding	IGKC protein
IPI00006574.1	FAM49A	X	X					Protein FAM49A
IPI00328243.2	PLD3	X	X	X	membrane,endoplasmic reticulum,cytoplasm	metabolic process	protein binding,catalytic activity	Phospholipase D3
IPI00449906.4	ARHGEF7	X	X			regulation of biological process,response to stimulus,cell communication	protein binding,enzyme regulator activity	cDNA FLJ56152, highly similar to Rho guanine nucleotide exchange factor 7
IPI00028635.5	RPN2	X	X		membrane,endoplasmic reticulum,cytoplasm,nucleus	metabolic process,transport	catalytic activity	Dolichyl-diphosphooligosaccharide--protein glycosyltransferase subunit 2
IPI00291939.1	SMC1A			X	membrane,cytoplasm,organelle lumen,chromosome,nucleus	cell organization and biogenesis,cell division,metabolic process,regulation of biological process,response to stimulus,cell communication,reproduction	protein binding,nucleotide binding,motor activity,catalytic activity	Structural maintenance of chromosomes protein 1A
IPI00254338.2	FAM134C	X	X		membrane,endoplasmic reticulum,cytoplasm			Protein FAM134C
IPI00218097.4	SPAG9	X	X		membrane		protein binding	117 kDa protein
IPI00027252.6	PHB2	X	X		mitochondrion,membrane,cytoplasm,nucleus	metabolic process,regulation of biological process	protein binding,receptor activity	Prohibitin-2
IPI00415032.2	NRCAM			X			protein binding	Isoform 4 of Neuronal cell adhesion molecule
IPI00166613.4	GALNTL1			X	membrane,cytoplasm,Golgi	metabolic process	catalytic activity	Isoform 1 of Putative polypeptide N-acetylgalactosaminyltransferase-like protein 1
IPI00002714.8	DKK3	X	X	X	extracellular	development,regulation of biological process,response to stimulus,cell communication		cDNA FLJ52545, highly similar to Dickkopf-related protein 3
IPI00044748.6	FMNL2	X	X		cytoskeleton,membrane,cytoplasm	cell organization and biogenesis	protein binding	Similar to FMNL2
IPI00221259.2	MYLK	X	X		membrane	metabolic process	protein binding,nucleotide binding,catalytic activity	Isoform Del-1790 of Myosin light chain kinase, smooth muscle
IPI00021951.1	KIAA0247			X	membrane			Uncharacterized protein KIAA0247
IPI00024658.3	OTUD7B		X		cytoskeleton,cytoplasm,nucleus	metabolic process,regulation of biological process,response to stimulus,cell communication	protein binding,DNA binding,metal ion binding,catalytic activity	OTU domain-containing protein 7B
IPI00216991.1	GIT2	X	X			metabolic process,regulation of biological process	metal ion binding,enzyme regulator activity	Isoform 3 of ARF GTPase-activating protein GIT2
IPI00219604.3	MAP2K1	X	X		cytoskeleton,membrane,mitochondrion,cytoplasm,Golgi,cytosol,nucleus ,endosome	cell proliferation,cell organization and biogenesis,development,transport,metabolic process,regulation of biological process,response to stimulus,cellular component movement,defense response,cell communication,cell differentiation	protein binding,signal transducer activity,nucleotide binding,catalytic activity	Isoform 1 of Dual specificity mitogen-activated protein kinase kinase 1
IPI00019449.1	RNASE2			X	extracellular,cytoplasm,vacuole	metabolic process,response to stimulus	catalytic activity	Non-secretory ribonuclease
IPI00106502.5	KEAP1			X	cytoskeleton,membrane,endoplasmic reticulum,cytoplasm,organelle lumen,nucleus	development,transport,metabolic process,regulation of biological process,cell differentiation	protein binding	Kelch-like ECH-associated protein 1
IPI00027972.1	LILRA2			X	membrane	regulation of biological process,response to stimulus,cell communication,defense response	protein binding,receptor activity	Isoform 1 of Leukocyte immunoglobulin-like receptor subfamily A member 2
IPI01025667.1	SERPINA3			X			enzyme regulator activity	cDNA FLJ35730 fis, clone TESTI2003131, highly similar to ALPHA-1-ANTICHYMOTRYPSIN
IPI00376976.4	ASAP1		X			metabolic process,regulation of biological process	protein binding,metal ion binding,enzyme regulator activity	Isoform 1 of Arf-GAP with SH3 domain, ANK repeat and PH domain-containing protein 1
IPI00743859.3	CTNNA2	X	X		cytoskeleton		protein binding,structural molecule activity	Isoform 2 of Catenin alpha-2
IPI00293539.4	CDH11		X	X	membrane		metal ion binding	Isoform 2 of Cadherin-11
IPI00386879.1	IGHA1			X	membrane		protein binding	cDNA FLJ14473 fis, clone MAMMA1001080, highly similar to Homo sapiens SNC73 protein (SNC73) mRNA
IPI00296058.2	EFEMP2			X	extracellular,membrane	response to stimulus,coagulation	protein binding,signal transducer activity,metal ion binding,receptor activity,structural molecule activity	EGF-containing fibulin-like extracellular matrix protein 2
IPI00021552.1	B3GALNT1			X	membrane,cytoplasm,Golgi	metabolic process	catalytic activity	UDP-GalNAc:beta-1,3-N-acetylgalactosaminyltransferase 1
IPI00256861.4	MACF1	X	X		cytoskeleton	regulation of biological process	protein binding,motor activity,catalytic activity	Isoform 2 of Microtubule-actin cross-linking factor 1, isoforms 1/2/3/5
IPI00032409.1	LAMTOR2	X	X		membrane,cytoplasm,vacuole,endosome	metabolic process,regulation of biological process,response to stimulus,cell communication,cell growth	protein binding	Isoform 1 of Ragulator complex protein LAMTOR2
IPI00760721.3	IGHV3-53			X				Immunglobulin heavy chain variable region
IPI00167638.4	GTPBP10			X	membrane,mitochondrion,cytoplasm,organelle lumen,chromosome,nucleus	transport,metabolic process	transporter activity,metal ion binding,nucleotide binding,catalytic activity	Isoform 1 of GTP-binding protein 10

IPI	GENE	Alzheimer's Hippocampus	Control hippocampus	CSF	Cellular localization	Biological process	Molecular function	Protein Description
IPI00007778.1	CTBS			X	cytoplasm,vacuole	metabolic process	catalytic activity	Di-N-acetylchitobiase
IPI00816274.2	CMTM1			X	membrane			Uncharacterized protein
IPI00295098.3	SRPRB			X	membrane,endoplasmic reticulum,cytoplasm	metabolic process,transport,regulation of biological process,response to stimulus,cell communication	transporter activity,nucleotide binding,receptor activity,catalytic activity	Signal recognition particle receptor subunit beta
IPI00008527.3	RPLP1	X	X		cytoplasm,ribosome,cytosol	cell organization and biogenesis,transport,metabolic process,reproduction	protein binding,RNA binding,structural molecule activity	60S acidic ribosomal protein P1
IPI00009890.3	SERPINE2			X	extracellular,cell surface,membrane,cytoplasm,cytosol	development,metabolic process,regulation of biological process,response to stimulus,cellular component movement,cell differentiation,coagulation	protein binding,enzyme regulator activity	Isoform 1 of Glia-derived nexin
IPI00009654.1	TRAPPC1	X	X		endoplasmic reticulum,cytoplasm,Golgi	transport		Trafficking protein particle complex subunit 1
IPI00009301.1	COPS7B	X	X		cytoplasm,nucleus	metabolic process	protein binding	Isoform 1 of COP9 signalosome complex subunit 7b
IPI00008107.2	LRFN2			X	membrane		protein binding	Leucine-rich repeat and fibronectin type-III domain-containing protein 2
IPI00470468.3	EFR3A		X	X				Isoform 3 of Protein EFR3 homolog A
IPI00013290.5	HDGFRP2			X	nucleus	metabolic process	DNA binding	Isoform 2 of Hepatoma-derived growth factor-related protein 2
IPI00023191.5	TOM1	X	X	X		transport		cDNA FLJ54710, highly similar to Target of Myb protein 1
IPI00149964.6	C18orf8		X				protein binding	Uncharacterized protein C18orf8
IPI00299150.4	CTSS			X	extracellular,membrane,cytoplasm,organelle lumen,vacuole,endosome	metabolic process,response to stimulus,defense response	catalytic activity	Cathepsin S
IPI00654703.3	SBF2	X	X			metabolic process	protein binding,catalytic activity	Myotubularin-related protein 13
IPI00296337.2	PRKDC	X	X	X	organelle lumen,nucleus	cell death,cell organization and biogenesis,development,metabolic process,regulation of biological process,response to stimulus,cellular component movement,reproduction,cell differentiation	protein binding,DNA binding,nucleotide binding,catalytic activity	Isoform 1 of DNA-dependent protein kinase catalytic subunit
IPI00216171.3	ENO2	X	X	X	membrane,cytoplasm,cytosol	metabolic process,response to stimulus	protein binding,metal ion binding,catalytic activity	Gamma-enolase
IPI00009374.4	C14orf129		X		cytoplasm			GSK3-beta interaction protein
IPI00290456.4	ICAM5	X	X	X	membrane	transport	protein binding	Intercellular adhesion molecule 5
IPI00011662.1	SPINT2			X	extracellular,membrane,cytoplasm	metabolic process,regulation of biological process,cellular component movement	enzyme regulator activity	Kunitz-type protease inhibitor 2
IPI00027438.2	FLOT1	X	X	X	cytoskeleton,membrane,cytoplasm,nucleus,endosome	development,cell organization and biogenesis,regulation of biological process,response to stimulus,cell communication,cell differentiation	protein binding	Flotillin-1
IPI00654820.2	ATP6	X	X		membrane,mitochondrion,cytoplasm	metabolic process,transport,response to stimulus	transporter activity	ATP synthase subunit a
IPI00829759.3	LOC100291056			X			catalytic activity	Similar to Immunglobulin heavy chain variable region
IPI00233358.1	ICA1L			X			protein binding	islet cell autoantigen 1-like protein isoform 2
IPI00291893.4	DCUN1D1	X	X				protein binding	DCN1-like protein 1
IPI00002406.2	BCAM	X	X	X	cell surface,membrane	regulation of biological process,response to stimulus,cell communication	signal transducer activity,protein binding,receptor activity	Basal cell adhesion molecule
IPI00171196.2	KRT13			X				Isoform 3 of Keratin, type I cytoskeletal 13
IPI00735451.4	IGHV5-51			X				Immunolgoobulin heavy chain
IPI00220014.2	IDI1		X				catalytic activity	Isoform 2 of Isopentenyl-diphosphate Delta-isomerase 1
IPI00156689.3	VAT1	X	X	X	mitochondrion,membrane,cytoplasm	cell organization and biogenesis,metabolic process,regulation of biological process	metal ion binding,nucleotide binding,catalytic activity	Synaptic vesicle membrane protein VAT-1 homolog
IPI00171649.8	AKAP7		X			metabolic process	catalytic activity	Isoform Alpha of A-kinase anchor protein 7 isoforms alpha and beta
IPI00016179.1	S100A13	X	X		extracellular,cytoplasm,cytosol,nucleus	cell proliferation,cell organization and biogenesis,transport,regulation of biological process,response to stimulus,cell communication	protein binding,metal ion binding	Protein S100-A13
IPI00010896.3	CLIC1	X	X	X	membrane,cytoplasm,nucleus	transport,regulation of biological process,response to stimulus,cell communication	protein binding,transporter activity	Chloride intracellular channel protein 1

IPI	GENE	Alzheimer's Hippocampus	Control hippocampus	CSF	Cellular localization	Biological process	Molecular function	Protein Description
IPI00334667.6	PTPRN2			X		metabolic process	catalytic activity	Isoform 2 of Receptor-type tyrosine-protein phosphatase N2
IPI00024887.1	BMP6			X	extracellular	cell proliferation,development,transport,metabolic process,regulation of biological process,response to stimulus,cell communication,defense response,cell differentiation,reproduction	protein binding	Bone morphogenetic protein 6
IPI00641582.1	BAG3	X	X		membrane,cytoplasm,cytosol	cell death,development,metabolic process,regulation of biological process	protein binding	BAG family molecular chaperone regulator 3
IPI00022792.3	MFAP4			X	extracellular	regulation of biological process,response to stimulus,cell communication	protein binding	Microfibril-associated glycoprotein 4
IPI00025729.1	CAMLG	X	X		membrane,endoplasmic reticulum,cytoplasm	metabolic process,regulation of biological process,response to stimulus,cell communication,defense response	protein binding	Calcium signal-modulating cyclophilin ligand
IPI01024808.1	NDUFA13		X		mitochondrion,membrane,cytoplasm,organelle lumen,nucleus	cell death,cell organization and biogenesis,development,transport,metabolic process,regulation of biological process,response to stimulus,cell communication,cell differentiation,cell growth	nucleotide binding,catalytic activity	26 kDa protein
IPI00253036.5	CD99	X			membrane,cytoplasm			Isoform I of CD99 antigen
IPI00217911.2	NUDT18		X				catalytic activity	Nudix (nucleoside diphosphate linked moiety X)-type motif 18
IPI00169283.1	MOSPD2	X			membrane		structural molecule activity	Isoform 1 of Motile sperm domain-containing protein 2
IPI00454910.2	CDC42BPG			X	extracellular,cytoskeleton,membrane,cytoplasm	cell organization and biogenesis,metabolic process,transport,regulation of biological process,response to stimulus,cell communication	protein binding,metal ion binding,motor activity,nucleotide binding,catalytic activity,enzyme regulator activity	Serine/threonine-protein kinase MRCK gamma
IPI00001895.1	PCDH8			X	membrane	development,cell communication	metal ion binding	Isoform 1 of Protocadherin-8
IPI00220648.5	PMVK	X			cytoplasm,cytosol	metabolic process,response to stimulus	nucleotide binding,catalytic activity	Phosphomevalonate kinase
IPI00022608.2	SORL1	X	X	X	extracellular,membrane	metabolic process,transport	protein binding,signal transducer activity,receptor activity	Sortilin-related receptor
IPI00165009.6	MBTD1			X	nucleus	metabolic process,regulation of biological process		Isoform 3 of MBT domain-containing protein 1
IPI00007694.5	PPME1	X	X			metabolic process	protein binding,catalytic activity,enzyme regulator activity	Isoform 1 of Protein phosphatase methylesterase 1
IPI00063903.5	USMG5	X	X		membrane,mitochondrion,cytoplasm			Up-regulated during skeletal muscle growth protein 5
IPI00186581.8	OS9			X	membrane	transport	transporter activity	protein OS-9 isoform 2 precursor
IPI00100748.4	HSPBP1	X						cDNA FLJ55382, highly similar to Hsp70-binding protein 1
IPI00000102.1	GNRH1			X	extracellular	cell death,cell proliferation,development,metabolic process,regulation of biological process,response to stimulus,cell communication,reproduction	protein binding	Progonadoliberin-1
IPI00019353.4	AGK	X	X		membrane,mitochondrion,cytoplasm	metabolic process,regulation of biological process,response to stimulus,cell communication	nucleotide binding,catalytic activity	Isoform 1 of Acylglycerol kinase, mitochondrial
IPI00306301.2	PDHA1	X	X			metabolic process	catalytic activity	pyruvate dehydrogenase E1 component subunit alpha, somatic form, mitochondrial isoform 2 precursor
IPI00015049.6	RGMA			X	membrane			Isoform 2 of Repulsive guidance molecule A
IPI00449049.5	PARP1	X	X		organelle lumen,nucleus	cell organization and biogenesis,metabolic process,regulation of biological process,response to stimulus	protein binding,DNA binding,metal ion binding,nucleotide binding,catalytic activity	Poly [ADP-ribose] polymerase 1
IPI00025815.2	TARDBP	X	X					Isoform 2 of TAR DNA-binding protein 43
IPI00782965.2	HIP1	X	X		cytoskeleton,mitochondrion,membrane,cytoplasm,Golgi,nucleus	cell death,cell organization and biogenesis,transport,metabolic process,regulation of biological process,cell differentiation	protein binding,motor activity,structural molecule activity,catalytic activity	Huntingtin-interacting protein 1
IPI00645814.1	MAP7D1	X	X	X	membrane	regulation of biological process,response to stimulus,cell communication	signal transducer activity,receptor activity,structural molecule activity	Isoform 2 of MAP7 domain-containing protein 1
IPI00028277.8	FTO	X	X		nucleus	cell proliferation,development,metabolic process,regulation of biological process,response to stimulus	metal ion binding,catalytic activity	Isoform 1 of Alpha-ketoglutarate-dependent dioxygenase FTO
IPI00166786.3	SVOP		X		membrane,cytoplasm	transport	transporter activity	Synaptic vesicle 2-related protein
IPI00844578.1	DHX9	X	X		cytoskeleton,cytoplasm,organelle lumen,nucleus	metabolic process,regulation of biological process,response to stimulus	protein binding,DNA binding,RNA binding,nucleotide binding,catalytic activity	ATP-dependent RNA helicase A

IPI	GENE	Alzheimer's Hippocampus	Control hippocampus	CSF	Cellular localization	Biological process	Molecular function	Protein Description
IPI00166766.4	C16orf89			X	extracellular		protein binding	Isoform 2 of UPF0764 protein C16orf89
IPI00304527.4	FAM83B			X				Protein FAM83B
IPI00171230.6	ERC1		X	X	membrane	metabolic process,regulation of biological process		Isoform 2 of ELKS/Rab6-interacting/CAST family member 1
IPI00023754.5	NELL1			X			protein binding,metal ion binding	cDNA FLJ45906 fis, clone OCBBF3026979, highly similar to Protein kinase C-binding protein NELL1
IPI00008315.4	EPHB1			X	membrane,cytoplasm,endosome	cell proliferation,cell organization and biogenesis,development,metabolic process,regulation of biological process,response to stimulus,cellular component movement,cell communication,cell differentiation	protein binding,signal transducer activity,nucleotide binding,receptor activity,catalytic activity	Ephrin type-B receptor 1
IPI00013296.3	RPS18	X	X		cytoplasm,ribosome,cytosol	cell organization and biogenesis,transport,metabolic process,reproduction	RNA binding,structural molecule activity	40S ribosomal protein S18
IPI00295618.1	PECAM1			X	extracellular,membrane,cytoplasm	development,transport,regulation of biological process,response to stimulus,cellular component movement,cell communication,coagulation	protein binding	Isoform Long of Platelet endothelial cell adhesion molecule
IPI00017940.3	LMBRD2		X	X	membrane			LMBR1 domain-containing protein 2
IPI00032957.1	UBE2I	X	X		cytoplasm,organelle lumen,chromosome,nucleus	cell organization and biogenesis,metabolic process,cell division,regulation of biological process,response to stimulus,cell communication	protein binding,nucleotide binding,catalytic activity	SUMO-conjugating enzyme UBC9
IPI00006991.1	CDKN1B	X	X		cytoplasm,organelle lumen,cytosol,nucleus,endosome	cell death,cell proliferation,development,cell organization and biogenesis,metabolic process,transport,regulation of biological process,response to stimulus,cellular component movement,cell communication,cell growth,reproduction	protein binding,signal transducer activity,catalytic activity,enzyme regulator activity	Cyclin-dependent kinase inhibitor 1B
IPI00384832.4	PALM2	X	X		membrane	cell organization and biogenesis,regulation of biological process		Isoform 1 of Paralemmin-2
IPI00027444.1	SERPINB1			X	extracellular,cytoplasm	metabolic process,regulation of biological process	enzyme regulator activity	Leukocyte elastase inhibitor
IPI00006443.3	CRYL1	X	X		cytoplasm,cytosol	metabolic process	protein binding,nucleotide binding,catalytic activity	Isoform 1 of Lambda-crystallin homolog
IPI00218130.3	PYGM	X	X	X	endoplasmic reticulum,cytoplasm,cytosol	metabolic process,response to stimulus,cellular homeostasis	nucleotide binding,catalytic activity	Glycogen phosphorylase, muscle form
IPI00656087.2	SIRPA	X	X	X	membrane	response to stimulus,cellular component movement,coagulation	protein binding,receptor activity	Isoform 1 of Tyrosine-protein phosphatase non-receptor type substrate 1
IPI00216694.3	PLS3	X	X	X	cytoplasm		protein binding,metal ion binding	Plastin-3
IPI00293464.5	DDB1	X	X	X	cytoplasm,organelle lumen,nucleus	metabolic process,regulation of biological process,response to stimulus,cell communication	protein binding,DNA binding	DNA damage-binding protein 1
IPI00003406.4	DBN1			X	cytoskeleton,cytoplasm	development,cell organization and biogenesis,regulation of biological process,cell communication,cell differentiation	protein binding	Isoform 1 of Drebrin
IPI00004560.1	DCLK1	X	X		membrane	development,transport,metabolic process,regulation of biological process,response to stimulus,cell communication,cell differentiation	signal transducer activity,nucleotide binding,catalytic activity	Isoform 2 of Serine/threonine-protein kinase DCLK1
IPI00031552.4	ELAVL3	X				development,cell differentiation	RNA binding,nucleotide binding	Isoform 1 of ELAV-like protein 3
IPI00075013.3	C1QTNF1			X	extracellular	cell organization and biogenesis,metabolic process,regulation of biological process,response to stimulus,cell communication	protein binding	complement C1q tumor necrosis factor-related protein 1 isoform 1 precursor
IPI00435208.1	CNKSR2	X	X		membrane,cytoplasm	regulation of biological process,response to stimulus,cell communication	protein binding	Isoform 1 of Connector enhancer of kinase suppressor of ras 2
IPI00412272.2	SH3BGRL2	X	X		nucleus	regulation of biological process,cellular homeostasis	protein binding,catalytic activity	SH3 domain-binding glutamic acid-rich-like protein 2
IPI00033030.2	ADRM1	X		X	membrane,proteasome,cytoplasm,organelle lumen,nucleus	cell organization and biogenesis,metabolic process,regulation of biological process	protein binding,enzyme regulator activity	Proteasomal ubiquitin receptor ADRM1
IPI00018236.2	GM2A	X	X	X	mitochondrion,cytoplasm,organelle lumen,vacuole,nucleus	metabolic process,regulation of biological process,response to stimulus	catalytic activity,enzyme regulator activity	Ganglioside GM2 activator
IPI00221255.2	MYLK			X		metabolic process	protein binding,nucleotide binding,catalytic activity	Isoform 2 of Myosin light chain kinase, smooth muscle
IPI00100329.7	ITPK1	X	X		cytoplasm,cytosol	metabolic process,regulation of biological process,response to stimulus,cell communication,coagulation	metal ion binding,nucleotide binding,catalytic activity	Isoform 1 of Inositol-tetrakisphosphate 1-kinase
IPI00010360.2	COL4A3			X	extracellular	cell death,cell proliferation,development,cell organization and biogenesis,metabolic process,regulation of biological process,response to stimulus,cell communication,cell differentiation	protein binding,structural molecule activity,enzyme regulator activity	Isoform 1 of Collagen alpha-3(IV) chain

IPI	GENE	Alzheimer's Hippocampus	Control hippocampus	CSF	Cellular localization	Biological process	Molecular function	Protein Description
IPI00641181.5	MARCKSL1	X	X	X	membrane	cell proliferation,transport,regulation of biological process	protein binding	MARCKS-related protein
IPI00783156.1	BMPR2			X	cell surface,membrane,cytoplasm	cell proliferation,development,cell organization and biogenesis,metabolic process,regulation of biological process,response to stimulus,cellular component movement,cell communication,cell growth,cell differentiation	signal transducer activity,protein binding,metal ion binding,nucleotide binding,receptor activity,catalytic activity	Bone morphogenetic protein receptor type-2
IPI00017802.1	AUH	X	X		mitochondrion,cytoplasm,organelle lumen	metabolic process	RNA binding,catalytic activity	Isoform 1 of Methylglutaconyl-CoA hydratase, mitochondrial
IPI00216250.5	CNTNAP4			X	extracellular	regulation of biological process,response to stimulus,cell communication	protein binding,structural molecule activity	Uncharacterized protein
IPI00008497.1	ODC1			X	cytoplasm,cytosol	cell proliferation,development,metabolic process,regulation of biological process,response to stimulus	catalytic activity	Ornithine decarboxylase
IPI00017529.1	CD58			X	membrane	response to stimulus,cellular component movement,coagulation	protein binding	Isoform 1 of Lymphocyte function-associated antigen 3
IPI00289862.5	SCRN1	X	X	X	membrane,cytoplasm,nucleus	transport,metabolic process	catalytic activity	Secernin-1
IPI00026270.1	CPM			X	membrane,cytoplasm,organelle lumen,nucleus	metabolic process	metal ion binding,catalytic activity	Carboxypeptidase M
IPI00015479.5	C12orf49			X	extracellular			UPF0454 protein C12orf49
IPI00166339.4	EPHA10			X	extracellular,membrane	metabolic process,regulation of biological process,response to stimulus,cell communication	protein binding,signal transducer activity,nucleotide binding,receptor activity,catalytic activity	Isoform 1 of Ephrin type-A receptor 10
IPI00012998.2	CPSF6	X	X		organelle lumen,nucleus	cell organization and biogenesis,metabolic process	protein binding,RNA binding,nucleotide binding	Isoform 1 of Cleavage and polyadenylation specificity factor subunit 6
IPI00016891.1	RAB6B	X	X		membrane,cytoplasm,Golgi,nucleus	transport,metabolic process,regulation of biological process,response to stimulus,cell communication	transporter activity,nucleotide binding,catalytic activity	Ras-related protein Rab-6B
IPI00007128.2	PRKCG	X	X		membrane	metabolic process,regulation of biological process,response to stimulus,cell communication	protein binding,metal ion binding,nucleotide binding,catalytic activity	cDNA FLJ60619, highly similar to Protein kinase C gamma type
IPI00012772.8	RPL8	X	X		cytoplasm,ribosome,cytosol	cell organization and biogenesis,metabolic process,transport,reproduction	RNA binding,structural molecule activity	60S ribosomal protein L8
IPI00002449.1	PDE4D		X		cytoskeleton,membrane,cytoplasm,cytosol	metabolic process,regulation of biological process,response to stimulus,cell communication,cell differentiation	protein binding,metal ion binding,nucleotide binding,catalytic activity	Isoform 4 of cAMP-specific 3',5'-cyclic phosphodiesterase 4D
IPI00152072.1	FIBIN			X	extracellular,endoplasmic reticulum,cytoplasm,Golgi			Fin bud initiation factor homolog
IPI00009439.1	SYT1	X	X	X	membrane,cytoplasm	cell organization and biogenesis,transport,regulation of biological process,response to stimulus,cell communication	protein binding,transporter activity,metal ion binding	Synaptotagmin-1
IPI00414410.3	MRPL22		X		cytoplasm,ribosome	metabolic process	structural molecule activity	26 kDa protein
IPI00003985.1	BCS1L	X	X		mitochondrion,membrane,cytoplasm	cell organization and biogenesis,metabolic process	protein binding,DNA binding,RNA binding,nucleotide binding,catalytic activity	Mitochondrial chaperone BCS1
IPI00056414.1	MAL2	X			membrane,cytoplasm		protein binding	Protein MAL2
IPI00024425.5	KIAA0664		X				protein binding	Uncharacterized protein
IPI00022007.1	WASF1	X	X		cytoskeleton,mitochondrion,membrane,cytoplasm	cell organization and biogenesis,cellular component movement	protein binding	Wiskott-Aldrich syndrome protein family member 1
IPI00152898.1	AP1S1		X		membrane,cytoplasm,Golgi,vacuole,cytosol	cell organization and biogenesis,transport,regulation of biological process,response to stimulus,defense response	transporter activity	Isoform 1 of AP-1 complex subunit sigma-1A
IPI00787853.2	IMPAD1			X	membrane,cytoplasm,Golgi	development,metabolic process,cell differentiation	metal ion binding,catalytic activity	Inositol monophosphatase 3
IPI00027223.2	IDH1	X	X	X	mitochondrion,cytoplasm,organelle lumen,cytosol	metabolic process,response to stimulus	protein binding,metal ion binding,nucleotide binding,catalytic activity	Isocitrate dehydrogenase [NADP] cytoplasmic
IPI00029756.2	MERTK			X	extracellular,membrane,cytoplasm	development,cell organization and biogenesis,metabolic process,transport,regulation of biological process,response to stimulus,cellular component movement,cell communication,cell differentiation,coagulation	protein binding,signal transducer activity,nucleotide binding,receptor activity,catalytic activity	Tyrosine-protein kinase Mer
IPI00216139.3	Sep-06	X	X		cytoskeleton,cytoplasm		nucleotide binding	Isoform I of Septin-6
IPI00029193.1	HGFAC			X	extracellular,endoplasmic reticulum,cytoplasm	metabolic process	protein binding,catalytic activity	Hepatocyte growth factor activator
IPI00790795.1	GFM1		X				nucleotide binding,catalytic activity	G elongation factor, mitochondrial 1, isoform CRA_e

IPI	GENE	Alzheimer's Hippocampus	Control hippocampus	CSF	Cellular localization	Biological process	Molecular function	Protein Description
IPI00001960.4	CLIC4	X	X	X	cell surface,cytoskeleton,mitochondrion,membrane,cytoplasm,organelle lumen,cytosol,nucleus	development,cell organization and biogenesis,transport,regulation of biological process,response to stimulus,cellular component movement,cell differentiation	protein binding,transporter activity	Chloride intracellular channel protein 4
IPI00018803.4	HOXD12			X	membrane,cytoplasm	metabolic process,regulation of biological process,response to stimulus,cell communication	signal transducer activity,protein binding,metal ion binding,nucleotide binding,receptor activity,catalytic activity	HOXD12 protein
IPI00465439.5	ALDOA	X	X	X	extracellular,cytoskeleton,cytoplasm,organelle lumen,cytosol	cell organization and biogenesis,metabolic process,transport,regulation of biological process,response to stimulus,cellular homeostasis,coagulation	protein binding,catalytic activity	Fructose-bisphosphate aldolase A
IPI00017163.6	HECW2			X	cytoplasm,nucleus	metabolic process	protein binding,catalytic activity	Isoform 1 of E3 ubiquitin-protein ligase HECW2
IPI00329791.20	DDX46			X	organelle lumen,nucleus	metabolic process	RNA binding,nucleotide binding,catalytic activity	Probable ATP-dependent RNA helicase DDX46
IPI00023598.2	TUBB4A	X	X	X	cytoskeleton,cytoplasm,cytosol	cell organization and biogenesis,metabolic process,cellular component movement	protein binding,nucleotide binding,structural molecule activity,catalytic activity	Tubulin beta-4 chain
IPI00456359.1	ATXN2L		X		membrane,organelle lumen,nucleus	metabolic process		Isoform 1 of Ataxin-2-like protein
IPI00004860.2	RARS		X		mitochondrion,cytoplasm,organelle lumen,cytosol,nucleus	metabolic process	protein binding,RNA binding,nucleotide binding,catalytic activity	Isoform Complexed of Arginyl-tRNA synthetase, cytoplasmic
IPI00305461.4	ITIH2	X	X	X		metabolic process	protein binding,enzyme regulator activity	Inter-alpha (Globulin) inhibitor H2, isoform CRA_a
IPI00018941.5	CALCB			X	extracellular	regulation of biological process,response to stimulus,cell communication,cellular homeostasis	protein binding	Calcitonin gene-related peptide 2
IPI00418163.3	C4B	X	X	X	extracellular	metabolic process,regulation of biological process,response to stimulus,defense response	protein binding,enzyme regulator activity	complement C4-B preproprotein
IPI00739237.1	LOC653879			X	extracellular		protein binding	similar to complement component 3
IPI00396023.1	CPLX1	X	X		membrane,cytoplasm,cytosol	transport,cell communication	protein binding,transporter activity	Complexin-1
IPI00915869.3	MDH1			X		metabolic process	catalytic activity	malate dehydrogenase, cytoplasmic isoform 3
IPI00043978.1	PARD3B			X	membrane	cell division	protein binding	Isoform 1 of Partitioning defective 3 homolog B
IPI00018146.1	YWHAQ	X	X	X	cytoskeleton,cytoplasm,organelle lumen,nucleus	metabolic process,transport,regulation of biological process,response to stimulus,cell communication	protein binding	14-3-3 protein theta
IPI00015476.1	SLC1A4	X	X		cytoskeleton,membrane,cytoplasm	transport,cell communication	transporter activity	Neutral amino acid transporter A
IPI00022799.1	AQP4	X	X		cell surface,membrane,cytoplasm	development,transport,response to stimulus,defense response	transporter activity	Isoform 2 of Aquaporin-4
IPI00021476.3	EIF4EBP3			X	cytoplasm	metabolic process,regulation of biological process	protein binding,translation regulator activity	Eukaryotic translation initiation factor 4E-binding protein 3
IPI00419235.5	GSTM5		X		membrane,endoplasmic reticulum,cytoplasm	metabolic process,response to stimulus	protein binding,catalytic activity	Glutathione S-transferase Mu 5
IPI00011876.3	MTAP	X	X			metabolic process	catalytic activity	cDNA FLJ59758, highly similar to S-methyl-5-thioadenosine phosphorylase
IPI00098902.4	OGDH	X	X	X	mitochondrion,membrane,cytoplasm,organelle lumen	development,metabolic process,cellular component movement,cell differentiation	catalytic activity	2-oxoglutarate dehydrogenase, mitochondrial
IPI00031691.1	RPL9	X	X		cytoplasm,ribosome,organelle lumen,cytosol,nucleus	cell organization and biogenesis,metabolic process,transport,reproduction	RNA binding,structural molecule activity	60S ribosomal protein L9
IPI00410457.2	SHISA6			X	membrane			Isoform 1 of Protein shisa-6 homolog
IPI00027898.3	FAM207A			X				Isoform A of Uncharacterized protein C21orf70
IPI00011276.1	BCKDHB	X	X		mitochondrion,cytoplasm,organelle lumen	metabolic process,response to stimulus	protein binding,catalytic activity	2-oxoisovalerate dehydrogenase subunit beta, mitochondrial
IPI00166619.1	SVOPL			X	membrane	transport	transporter activity	Isoform 2 of Putative transporter SVOPL
IPI00029601.6	CTTN	X	X		cytoskeleton,cytoplasm		protein binding	Src substrate cortactin
IPI00306844.1	CRHBP			X	extracellular,cytoskeleton,endoplasmic reticulum,cytoplasm,Golgi,vacuole,nucleus,endosome	transport,metabolic process,regulation of biological process,response to stimulus,defense response,cell communication,reproduction	protein binding	Corticotropin-releasing factor-binding protein
IPI00465255.7	PRAP1			X	extracellular			Isoform 1 of Proline-rich acidic protein 1
IPI00022277.3	COA3			X	membrane,mitochondrion,cytoplasm			Coiled-coil domain-containing protein 56

IPI	GENE	Alzheimer's Hippocampus	Control hippocampus	CSF	Cellular localization	Biological process	Molecular function	Protein Description
IPI00029184.1	HAPLN2	X	X		extracellular		protein binding	Hyaluronan and proteoglycan link protein 2
IPI00016342.1	RAB7A	X	X		cytoplasm,Golgi,vacuole,nucleus,endosome	cell organization and biogenesis,transport,metabolic process,regulation of biological process,response to stimulus,cell communication	protein binding,nucleotide binding,catalytic activity	Ras-related protein Rab-7a
IPI00298949.1	GAK	X	X		membrane,cytoplasm,Golgi	metabolic process	protein binding,nucleotide binding,catalytic activity	Cyclin-G-associated kinase
IPI00792626.1	TF			X	extracellular	transport,cellular homeostasis	metal ion binding	14 kDa protein
IPI00017968.1	ADM			X	extracellular,cytoplasm	cell proliferation,development,cell organization and biogenesis,metabolic process,transport,regulation of biological process,response to stimulus,cell communication,cellular homeostasis,reproduction,cell differentiation	protein binding	ADM
IPI00643920.4	TKT	X	X	X	membrane,organelle lumen,nucleus	metabolic process	catalytic activity	cDNA FLJ54957, highly similar to Transketolase
IPI01023063.1	FAR2			X		metabolic process	nucleotide binding,catalytic activity	unnamed protein product
IPI00031510.1	SEMA3A			X	extracellular,membrane	cell death,development,cell organization and biogenesis,regulation of biological process,response to stimulus,cellular component movement,cell communication,cell differentiation,cell growth	protein binding,receptor activity	Semaphorin-3A
IPI00869000.2	HECTD4	X	X		membrane	metabolic process	protein binding,catalytic activity	probable E3 ubiquitin-protein ligase C12orf51
IPI00059366.4	H2AFY	X	X		chromosome,nucleus	cell organization and biogenesis,metabolic process	DNA binding	Isoform 3 of Core histone macro-H2A.1
IPI00014238.2	KARS		X		extracellular,cytoskeleton,mitochondrion,membrane,cytoplasm,organelle lumen,cytosol,nucleus	metabolic process	RNA binding,metal ion binding,nucleotide binding,catalytic activity	Isoform Cytoplasmic of Lysyl-tRNA synthetase
IPI00218733.6	SOD1	X	X	X	extracellular,membrane,mitochondrion,cytoplasm,organelle lumen,cytosol,nucleus	cell death,cell organization and biogenesis,development,metabolic process,regulation of biological process,response to stimulus,reproduction,transport,cellular component movement,cell communication,cellular homeostasis,cell differentiation,coagulation	antioxidant activity,protein binding,metal ion binding,catalytic activity	Superoxide dismutase [Cu-Zn]
IPI00011400.2	TIAM1			X	membrane,cytoplasm,cytosol	cell death,development,cell organization and biogenesis,regulation of biological process,response to stimulus,cell communication,cell differentiation	protein binding,signal transducer activity,enzyme regulator activity	T-lymphoma invasion and metastasis-inducing protein 1
IPI00867509.1	CORO1C	X	X	X			protein binding	Coronin-1C_i3 protein
IPI00219038.9	H3F3AP4	X	X		extracellular,organelle lumen,chromosome,nucleus	cell organization and biogenesis,metabolic process,response to stimulus,coagulation	protein binding,DNA binding,metal ion binding	Histone H3.3
IPI00298702.2	SLC39A6			X	membrane,endoplasmic reticulum,cytoplasm	transport,cellular homeostasis	transporter activity	Isoform 1 of Zinc transporter ZIP6
IPI00297714.2	SNCG	X	X	X	cytoskeleton,cytoplasm	transport,regulation of biological process,response to stimulus,cell communication		Gamma-synuclein
IPI00297579.4	C15orf57	X	X		cytoskeleton,membrane,organelle lumen,chromosome,nucleus	cell organization and biogenesis,metabolic process,regulation of biological process	protein binding	Chromobox protein homolog 3
IPI00956556.1	NPAS2			X	cytoplasm,organelle lumen,nucleus	metabolic process,regulation of biological process,response to stimulus,cell communication	signal transducer activity,protein binding	neuronal PAS domain protein 2, isoform CRA_e
IPI00002142.2	PCDH10			X	membrane		metal ion binding	Protocadherin-10
IPI00025717.1	MTX2	X	X		membrane,mitochondrion,cytoplasm	cell organization and biogenesis,transport,metabolic process		Metaxin-2
IPI00009335.2	FAM203A			X				Protein FAM203A
IPI00029107.2	WRN			X	cytoskeleton,cytoplasm,organelle lumen,nucleus	cell death,cell organization and biogenesis,development,transport,metabolic process,regulation of biological process,response to stimulus,cell communication	protein binding,DNA binding,metal ion binding,nucleotide binding,catalytic activity	Werner syndrome ATP-dependent helicase
IPI00440693.4	FDX1L		X					20 kDa protein
IPI00022337.1	RBP3			X	extracellular	metabolic process,transport	catalytic activity	Retinol-binding protein 3
IPI00100160.3	CAND1	X	X		membrane,cytoplasm,nucleus	transport,metabolic process,regulation of biological process,cell differentiation	protein binding	Isoform 1 of Cullin-associated NEDD8-dissociated protein 1
IPI00219344.4	HPCAL1	X	X		extracellular	regulation of biological process,response to stimulus,cell communication	metal ion binding	Hippocalcin-like protein 1
IPI00784090.2	CCT8	X	X		cytoskeleton,cytoplasm,cytosol	metabolic process	protein binding,nucleotide binding,catalytic activity	T-complex protein 1 subunit theta

IPI	GENE	Alzheimer's Hippocampus	Control hippocampus	CSF	Cellular localization	Biological process	Molecular function	Protein Description
IPI00007834.2	ANK2	X	X	X	membrane,nucleus	metabolic process,regulation of biological process,response to stimulus,cell communication,reproduction	protein binding	Isoform 1 of Ankyrin-2
IPI00004480.1	ADAMDEC1			X	extracellular	metabolic process,regulation of biological process,response to stimulus	metal ion binding,catalytic activity	ADAM DEC1
IPI00872555.2	CFI			X	membrane	metabolic process	protein binding,receptor activity,catalytic activity	unnamed protein product
IPI00745660.2	IGL@			X			protein binding	IGL@ protein
IPI00014577.1	RAB18	X	X		membrane,cytoplasm,nucleus	development,transport,metabolic process,regulation of biological process,response to stimulus,cell communication	nucleotide binding,catalytic activity	Ras-related protein Rab-18
IPI00010808.1	IFNGR1			X	membrane	regulation of biological process,response to stimulus,cell communication,defense response	signal transducer activity,protein binding,receptor activity	Interferon gamma receptor 1
IPI00604430.1	REEP2	X	X	X			receptor activity	Isoform 2 of Receptor expression-enhancing protein 2
IPI00021983.1	NCSTN			X	membrane,endoplasmic reticulum,cytoplasm,Golgi,vacuole	cell death,metabolic process,regulation of biological process,response to stimulus,cell communication	protein binding,catalytic activity	Isoform 1 of Nicastrin
IPI00879709.3	C6			X			protein binding	Complement component C6 precursor
IPI00027721.1	PDGFRA			X	membrane,cytoplasm,nucleus	cell proliferation,cell organization and biogenesis,development,metabolic process,regulation of biological process,response to stimulus,cellular component movement,cell communication,cellular homeostasis,cell differentiation,reproduction,coagulation	signal transducer activity,protein binding,nucleotide binding,receptor activity,catalytic activity	Isoform 1 of Alpha-type platelet-derived growth factor receptor
IPI00032206.2	PDLIM4	X	X				protein binding,metal ion binding	Isoform 1 of PDZ and LIM domain protein 4
IPI00152358.1	CPT1C		X		membrane,mitochondrion,endoplasmic reticulum,cytoplasm	metabolic process	catalytic activity	Isoform 1 of Carnitine O-palmitoyltransferase 1, brain isoform
IPI00150881.3	CEP85L			X	cytoskeleton,cytoplasm			Isoform 1 of Coiled-coil domain-containing protein C6orf204
IPI00024036.1	CDH8			X	membrane	cell organization and biogenesis	metal ion binding	Cadherin-8
IPI00853219.1	RAPGEF2	X	X		membrane	metabolic process,regulation of biological process,response to stimulus,cell communication	protein binding,signal transducer activity,metal ion binding,enzyme regulator activity	Rap guanine nucleotide exchange factor 2
IPI00025160.2	VAC14	X	X		membrane,endoplasmic reticulum,cytoplasm,endosome	regulation of biological process,response to stimulus,cell communication	receptor activity	Protein VAC14 homolog
IPI00152692.2	DTD1	X	X		cytoplasm,organelle lumen,nucleus	metabolic process	catalytic activity	D-tyrosyl-tRNA(Tyr) deacylase 1
IPI00294395.1	C8B			X	extracellular,membrane	cell death,metabolic process,regulation of biological process,response to stimulus,defense response	protein binding	Complement component C8 beta chain
IPI00103599.1	BRI3BP	X	X		mitochondrion,membrane,cytoplasm			BRI3-binding protein
IPI00003843.4	TJP2	X	X		membrane,cytoplasm,organelle lumen,cytosol,nucleus	cell death,cell organization and biogenesis,regulation of biological process,response to stimulus,cell communication	protein binding,catalytic activity	Isoform A1 of Tight junction protein ZO-2
IPI00021458.1	EHD3	X	X				nucleotide binding,catalytic activity	EH domain-containing protein 3
IPI00827940.1	IGHV3-23			X			protein binding	Mu-chain (Fragment)
IPI00328431.3	UNC5B			X	membrane	cell death,cell organization and biogenesis,development,regulation of biological process,response to stimulus,cell communication,cell differentiation	signal transducer activity,protein binding,receptor activity	Isoform 1 of Netrin receptor UNC5B
IPI00304435.3	NIPSNAP1	X	X		membrane,mitochondrion,cytoplasm			Protein NipSnap homolog 1
IPI00033132.3	RNF7	X	X		cytoplasm,organelle lumen,nucleus	cell death,metabolic process,regulation of biological process,response to stimulus	protein binding,metal ion binding,catalytic activity	Isoform 1 of RING-box protein 2
IPI00003148.1	MAPK10	X	X			metabolic process	nucleotide binding,catalytic activity	Isoform Alpha-1 of Mitogen-activated protein kinase 10
IPI00465045.2	DIP2B	X	X	X	nucleus	metabolic process	protein binding,catalytic activity	Disco-interacting protein 2 homolog B
IPI00027416.1	BBOX1	X	X		cytoskeleton,cytoplasm,cytosol	metabolic process	metal ion binding,catalytic activity	Gamma-butyrobetaine dioxygenase
IPI00292300.3	CNTNAP5			X	membrane	regulation of biological process,response to stimulus,cell communication	protein binding	Contactin-associated protein-like 5
IPI00005129.8	SCAMP1	X	X	X	membrane,cytoplasm,Golgi,endosome	transport	protein binding	Isoform 1 of Secretory carrier-associated membrane protein 1
IPI00374129.1	NLRP3			X			catalytic activity	Isoform 1 of NACHT, LRR and PYD domains-containing protein 3

IPI	GENE	Alzheimer's Hippocampus	Control hippocampus	CSF	Cellular localization	Biological process	Molecular function	Protein Description
IPI00009471.1	WDR3			X	membrane,organelle lumen,nucleus		protein binding	WD repeat-containing protein 3
IPI00011261.2	C8G			X	extracellular,membrane	cell death,metabolic process,regulation of biological process,response to stimulus,defense response	protein binding	Complement component C8 gamma chain
IPI00419966.2	ABI3BP			X	membrane		protein binding	Uncharacterized protein
IPI00444262.3	NCL	X	X				nucleotide binding	cDNA FLJ45706 fis, clone FEBRA2028457, highly similar to Nucleolin
IPI00025683.2	TACC1		X		cytoskeleton,membrane,cytoplasm,nucleus	cell proliferation,development,cell organization and biogenesis,cell division,transport,regulation of biological process,cell differentiation	protein binding,transporter activity,motor activity,catalytic activity	Isoform 1 of Transforming acidic coiled-coil-containing protein 1
IPI00031140.3	C2orf88	X	X					UPF0733 protein C2orf88
IPI00291751.6	C2orf47		X		mitochondrion,cytoplasm			Uncharacterized protein C2orf47, mitochondrial
IPI00013495.1	ABCF1			X		metabolic process	nucleotide binding,catalytic activity	Isoform 2 of ATP-binding cassette sub-family F member 1
IPI00783492.1	LTBP4			X			metal ion binding	Isoform 2 of Latent-transforming growth factor beta-binding protein 4
IPI00045536.2	CHID1			X		metabolic process	catalytic activity	Isoform 3 of Chitinase domain-containing protein 1
IPI00887421.1	LOC652460		X				protein binding	similar to plectin 1, partial
IPI00218236.6	PPP1CB	X	X		cytoplasm,organelle lumen,nucleus	cell division,metabolic process,regulation of biological process	protein binding,metal ion binding,catalytic activity	Serine/threonine-protein phosphatase PP1-beta catalytic subunit
IPI00011994.1	ENPP5			X	extracellular,membrane	metabolic process,cell communication	catalytic activity	Ectonucleotide pyrophosphatase/phosphodiesterase family member 5
IPI00399212.3	LOC389842		X			transport	protein binding	similar to RAN binding protein 1
IPI00008303.6	NAGPA			X	membrane,cytoplasm,Golgi	cell organization and biogenesis,transport,metabolic process	protein binding,catalytic activity	Isoform 1 of N-acetylglucosamine-1-phosphodiester alpha-N-acetylglucosaminidase
IPI00220303.6	MAN2A2			X	membrane,cytoplasm,Golgi	metabolic process	metal ion binding,catalytic activity	Isoform 3 of Alpha-mannosidase 2x
IPI00011518.2	BACE1			X	cell surface,membrane,endoplasmic reticulum,cytoplasm,Golgi,endosome	metabolic process	protein binding,catalytic activity	Isoform A of Beta-secretase 1
IPI00871326.1	PLXNA1			X	membrane	development,regulation of biological process,response to stimulus,cell communication	signal transducer activity,protein binding,receptor activity	Uncharacterized protein
IPI00012877.3	IFNAR1			X	membrane	metabolic process,regulation of biological process,response to stimulus,defense response,cell communication	signal transducer activity,protein binding,receptor activity	Isoform 1 of Interferon alpha/beta receptor 1
IPI00154742.6	IGL@	X	X	X			protein binding	IGL@ protein
IPI00217775.1	CD74			X	membrane	transport,response to stimulus	protein binding	Isoform 2 of HLA class II histocompatibility antigen gamma chain
IPI00182944.2	CAMK2B			X		metabolic process	protein binding,nucleotide binding,catalytic activity	Isoform 3 of Calcium/calmodulin-dependent protein kinase type II subunit beta
IPI00027255.1	MYL6B		X		cytoskeleton,cytoplasm,cytosol	development,cellular component movement	protein binding,metal ion binding,motor activity,structural molecule activity,catalytic activity	Myosin light chain 6B
IPI00843910.2	FUCA1			X	cytoplasm,vacuole	metabolic process	catalytic activity	Tissue alpha-L-fucosidase
IPI00023019.1	SHBG			X	extracellular	transport,cell growth,reproduction,cell differentiation	protein binding	Isoform 1 of Sex hormone-binding globulin
IPI00553215.3	IGLV2-18			X				Similar to V1-5 protein
IPI00000856.7	FERMT2	X	X		cytoskeleton,membrane,cytoplasm,organelle lumen,cytosol,nucleus	cell organization and biogenesis,regulation of biological process	protein binding	Isoform 1 of Fermitin family homolog 2
IPI00306850.4	MEGF6			X	extracellular		protein binding,metal ion binding	Isoform 1 of Multiple epidermal growth factor-like domains protein 6
IPI00328550.3	THBS4			X	extracellular	cell proliferation,development,cell organization and biogenesis,cell division,metabolic process,regulation of biological process,response to stimulus,cellular component movement,cell differentiation	protein binding,metal ion binding	Thrombospondin-4
IPI00440932.1	ADAM9			X	extracellular,cell surface,membrane,cytoplasm	cell organization and biogenesis,development,transport,metabolic process,regulation of biological process,response to stimulus,cellular component movement,cell communication,cell differentiation	protein binding,metal ion binding,catalytic activity	Isoform 1 of Disintegrin and metalloproteinase domain-containing protein 9

IPI	GENE	Alzheimer's Hippocampus	Control hippocampus	CSF	Cellular localization	Biological process	Molecular function	Protein Description
IPI00000076.2	NGF			X	extracellular,endoplasmic reticulum,cytoplasm,Golgi,organelle lumen,endosome	cell death,cell proliferation,cell organization and biogenesis,development,transport,metabolic process,regulation of biological process,response to stimulus,cell communication,defense response,cell growth,cell differentiation	signal transducer activity,protein binding	Beta-nerve growth factor
IPI00479116.2	CPN2			X	extracellular,membrane	metabolic process,regulation of biological process	protein binding,enzyme regulator activity	Carboxypeptidase N subunit 2
IPI00328609.3	SERPINA4			X	extracellular	metabolic process,regulation of biological process	enzyme regulator activity	Kallistatin
IPI00000190.1	CD81	X	X	X	membrane	cell proliferation,metabolic process,regulation of biological process,response to stimulus,cell communication,reproduction	protein binding	CD81 antigen
IPI00026059.2	SNTB1		X		cytoskeleton,membrane,cytoplasm		protein binding	Isoform 1 of Beta-1-syntrophin
IPI00024664.1	USP5	X	X	X	cytoplasm,vacuole	metabolic process,regulation of biological process	protein binding,metal ion binding,catalytic activity	Isoform Long of Ubiquitin carboxyl-terminal hydrolase 5
IPI00418125.3	LAYN			X	cell surface,membrane			Isoform 1 of Layilin
IPI00029723.1	FSTL1			X	extracellular	regulation of biological process,response to stimulus,cell communication	protein binding,metal ion binding	Follistatin-related protein 1
IPI00005690.3	MATN3			X	extracellular	development	protein binding,metal ion binding,structural molecule activity	Matrilin-3
IPI00002188.2	ARFGEF1		X		cytoplasm,Golgi,organelle lumen,nucleus	transport,regulation of biological process,response to stimulus,cell communication	protein binding,enzyme regulator activity	Brefeldin A-inhibited guanine nucleotide-exchange protein 1
IPI00332091.9	RPP40	X			organelle lumen,nucleus	metabolic process	catalytic activity	Isoform 2 of Ribonuclease P protein subunit p40
IPI00024368.1	ATP9A	X	X		membrane,cytoplasm,Golgi,endosome	cell organization and biogenesis,metabolic process,transport	transporter activity,metal ion binding,nucleotide binding,catalytic activity	Isoform Long of Probable phospholipid-transporting ATPase IIA
IPI00216572.1	BARX2			X	membrane,organelle lumen,nucleus	development,transport,metabolic process,regulation of biological process,cell differentiation	DNA binding,transporter activity	Homeobox protein BarH-like 2
IPI00024673.2	MAPK9	X	X		mitochondrion,cytoplasm,organelle lumen,nucleus,cytosol	cell death,metabolic process,regulation of biological process,response to stimulus,defense response,cell communication,cell differentiation	signal transducer activity,protein binding,nucleotide binding,catalytic activity	Isoform Alpha-2 of Mitogen-activated protein kinase 9
IPI00749514.1	ATRNL1			X	membrane	regulation of biological process,response to stimulus,cell communication	protein binding	Isoform 1 of Attractin-like protein 1
IPI00009982.1	TDRKH		X				RNA binding	Tudor and KH domain-containing protein
IPI00554541.2	ILVBL	X	X		membrane	metabolic process	metal ion binding,catalytic activity	Isoform 1 of Acetolactate synthase-like protein
IPI00219420.3	SMC3			X	extracellular,cytoskeleton,cytoplasm,organelle lumen,chromosome,nucleus	cell organization and biogenesis,metabolic process,cell division,regulation of biological process,response to stimulus,cell communication,reproduction	protein binding,nucleotide binding,motor activity,catalytic activity	Structural maintenance of chromosomes protein 3
IPI00385480.5	CASKIN1	X	X	X	cytoplasm	regulation of biological process,response to stimulus,cell communication	protein binding	Caskin-1
IPI00012970.1	PPP6C		X		cytoplasm,cytosol	metabolic process	protein binding,metal ion binding,catalytic activity	Isoform 1 of Serine/threonine-protein phosphatase 6 catalytic subunit
IPI00063025.1	ACY3	X	X		membrane,cytoplasm	metabolic process	protein binding,metal ion binding,catalytic activity	Aspartoacylase-2
IPI00219568.4	PGK2		X		cytoplasm,cytosol	metabolic process	nucleotide binding,catalytic activity	Phosphoglycerate kinase 2
IPI00385901.1	COQ9		X					Isoform 2 of Ubiquinone biosynthesis protein COQ9, mitochondrial
IPI00294193.4	ITIH4			X		metabolic process	protein binding,enzyme regulator activity	Isoform 1 of Inter-alpha-trypsin inhibitor heavy chain H4
IPI00477597.2	HPR			X	extracellular	metabolic process	protein binding,catalytic activity	Isoform 1 of Haptoglobin-related protein
IPI00465322.3	BOC			X			protein binding	Isoform 3 of Brother of CDO
IPI00376005.2	EIF5A	X	X			cell organization and biogenesis,metabolic process,regulation of biological process	RNA binding	Isoform 2 of Eukaryotic translation initiation factor 5A-1
IPI00000104.1	RNGTT			X	organelle lumen,nucleus	metabolic process,response to stimulus	nucleotide binding,catalytic activity	Isoform 1 of mRNA-capping enzyme
IPI00024919.3	PRDX3	X	X	X	mitochondrion,cytoplasm,cytosol,endosome	cell death,cell proliferation,cell organization and biogenesis,development,metabolic process,regulation of biological process,response to stimulus,cellular homeostasis,cell differentiation	antioxidant activity,protein binding,catalytic activity,enzyme regulator activity	Thioredoxin-dependent peroxide reductase, mitochondrial
IPI00219703.3	PVALB	X	X	X	cytoplasm		metal ion binding	Parvalbumin alpha
IPI00072917.2	COL6A3			X			protein binding,enzyme regulator activity	Uncharacterized protein

IPI	GENE	Alzheimer's Hippocampus	Control hippocampus	CSF	Cellular localization	Biological process	Molecular function	Protein Description
IPI00297931.4	SYNRG		X		membrane,cytoplasm,Golgi	transport	protein binding,metal ion binding	Isoform 1 of Synergin gamma
IPI00743825.1	COPS2	X	X		cytoplasm,nucleus	metabolic process,regulation of biological process,response to stimulus,cell communication	protein binding,signal transducer activity	Isoform 1 of COP9 signalosome complex subunit 2
IPI00163505.2	RBM39	X			cytoskeleton,cytoplasm,organelle lumen,nucleus	metabolic process,regulation of biological process	protein binding,RNA binding,nucleotide binding	Isoform 1 of RNA-binding protein 39
IPI00419720.3	DMKN			X	cytoplasm			dermokine epsilon-3
IPI00008529.1	RPLP2	X	X		cytoplasm,ribosome,cytosol	cell organization and biogenesis,transport,metabolic process,reproduction	RNA binding,structural molecule activity	60S acidic ribosomal protein P2
IPI00022392.1	C1QA			X	extracellular,membrane	metabolic process,regulation of biological process,response to stimulus,defense response,cell communication	protein binding	Complement C1q subcomponent subunit A
IPI00019485.6	ECHDC2			X		metabolic process	catalytic activity	Isoform 2 of Enoyl-CoA hydratase domain-containing protein 2, mitochondrial
IPI00432525.4	SIGLEC14			X	membrane		protein binding	Sialic acid-binding Ig-like lectin 14
IPI00022744.5	CSE1L	X	X		cytoplasm,nucleus	cell death,cell proliferation,transport	protein binding,transporter activity	Isoform 1 of Exportin-2
IPI00033025.9	Sep-07	X	X		cytoskeleton,membrane,cytoplasm,organelle lumen,nucleus		nucleotide binding	Uncharacterized protein
IPI00647297.2	PRR24			X	membrane			Proline-rich protein 24
IPI00003964.4	USP9X	X	X			metabolic process	catalytic activity	Isoform 2 of Probable ubiquitin carboxyl-terminal hydrolase FAF-X
IPI00017305.2	RPS6KA1	X	X		membrane,cytoplasm,organelle lumen,nucleus,cytosol	cell death,cell organization and biogenesis,development,metabolic process,regulation of biological process,response to stimulus,defense response,cell communication,cell growth,cell differentiation	protein binding,metal ion binding,nucleotide binding,catalytic activity,enzyme regulator activity	Isoform 1 of Ribosomal protein S6 kinase alpha-1
IPI00221232.9	GNG12	X	X		cytoskeleton,membrane	development,metabolic process,regulation of biological process,response to stimulus,cell communication	signal transducer activity	Guanine nucleotide-binding protein G(I)/G(S)/G(O) subunit gamma-12
IPI00376394.4	QSOX2			X	extracellular,membrane,nucleus	metabolic process,regulation of biological process,cellular homeostasis	catalytic activity	Sulfhydryl oxidase 2
IPI00855824.1	DPP6			X	membrane	metabolic process	catalytic activity	dipeptidyl aminopeptidase-like protein 6 isoform 3
IPI00735934.2	LOC646070			X			protein binding	similar to capicua homolog
IPI00219472.4	SYT12		X				protein binding	Synaptotagmin XII
IPI00013488.4	CHN1	X	X		cytoplasm,cytosol	cell organization and biogenesis,development,metabolic process,regulation of biological process,response to stimulus,cell communication,cell differentiation	protein binding,metal ion binding,enzyme regulator activity	Isoform Alpha-2 of N-chimaerin
IPI00024787.2	SLC27A2		X		membrane,mitochondrion,endoplasmic reticulum,cytoplasm,organelle lumen	transport,metabolic process	protein binding,transporter activity,nucleotide binding,catalytic activity	Very long-chain acyl-CoA synthetase
IPI00013459.1	NDUFB5		X		mitochondrion,membrane,cytoplasm	metabolic process,transport	catalytic activity	NADH dehydrogenase [ubiquinone] 1 beta subcomplex subunit 5, mitochondrial
IPI00374054.3	ENAH	X	X				protein binding	Isoform 2 of Protein enabled homolog
IPI00375746.4	GBP6			X		metabolic process	nucleotide binding,catalytic activity	Isoform 1 of Guanylate-binding protein 6
IPI00030739.1	APOM			X	extracellular,membrane	cell organization and biogenesis,metabolic process,transport,regulation of biological process,response to stimulus	antioxidant activity,transporter activity,catalytic activity	Apolipoprotein M
IPI00006803.2	CHST10			X	membrane	metabolic process	catalytic activity	cDNA FLJ55226, highly similar to Carbohydrate sulfotransferase 10
IPI00181174.1	NLGN4X			X	cytoskeleton,cell surface,membrane	cell organization and biogenesis,development,metabolic process,regulation of biological process,response to stimulus,cell communication,cellular homeostasis	protein binding,receptor activity,catalytic activity	Isoform 1 of Neuroligin-4, X-linked
IPI00024364.2	TNPO1	X	X		cytoplasm,cytosol,nucleus	metabolic process,transport	protein binding,transporter activity	Isoform 1 of Transportin-1
IPI00397904.6	NUP93		X		membrane,nucleus	transport,metabolic process,regulation of biological process,response to stimulus,cell communication		Nuclear pore complex protein Nup93
IPI00217493.5	MB			X		development,transport,response to stimulus,cell differentiation	transporter activity,metal ion binding	Myoglobin
IPI00062598.3	PHYHD1	X	X				catalytic activity	Isoform 3 of Phytanoyl-CoA dioxygenase domain-containing protein 1

IPI	GENE	Alzheimer's Hippocampus	Control hippocampus	CSF	Cellular localization	Biological process	Molecular function	Protein Description
IPI00107555.5	PFN2	X	X		cytoskeleton	cell organization and biogenesis	protein binding	Profilin
IPI00410488.2	CD276			X	cell surface,membrane	cell proliferation,development,metabolic process,regulation of biological process,response to stimulus,defense response,cell differentiation	protein binding	Isoform 1 of CD276 antigen
IPI00020470.4	GLT8D1			X	mitochondrion,membrane,cytoplasm		catalytic activity	Isoform 1 of Glycosyltransferase 8 domain-containing protein 1
IPI00219301.7	MARCKS	X	X	X	cytoskeleton,membrane,cytoplasm,nucleus	transport,metabolic process,regulation of biological process,cell communication	protein binding	Myristoylated alanine-rich C-kinase substrate
IPI00029533.1	ITGB8		X	X	membrane	development,metabolic process,regulation of biological process,response to stimulus,cell communication	protein binding,receptor activity	Integrin beta-8
IPI00176903.2	PTRF	X	X		membrane,mitochondrion,endoplasmic reticulum,cytoplasm,organelle lumen,cytosol,nucleus	metabolic process,regulation of biological process	protein binding,RNA binding	Isoform 1 of Polymerase I and transcript release factor
IPI00099650.2	JAG1			X	extracellular,membrane	cell proliferation,development,metabolic process,regulation of biological process,response to stimulus,cellular component movement,cell communication,cell differentiation	protein binding,metal ion binding,structural molecule activity	Protein jagged-1
IPI00103525.1	PSPC1		X		cytoplasm,organelle lumen,nucleus	metabolic process,regulation of biological process	protein binding,RNA binding,nucleotide binding	Isoform 1 of Paraspeckle component 1
IPI00292950.4	SERPIND1			X			enzyme regulator activity	SERPIND1
IPI00550020.3	PTMS	X	X		cytoplasm,cytosol,nucleus	metabolic process		Parathymosin
IPI00152524.2	NETO1			X	extracellular,cytoskeleton,membrane	regulation of biological process,response to stimulus,cell communication	protein binding,receptor activity	Isoform 3 of Neuropilin and tolloid-like protein 1
IPI00039626.3	FAM120A		X		membrane	metabolic process,response to stimulus	catalytic activity	Isoform D of Constitutive coactivator of PPAR-gamma-like protein 1
IPI00455165.2	PANX1	X			membrane,endoplasmic reticulum,cytoplasm	cell organization and biogenesis,transport,regulation of biological process,response to stimulus,defense response,cell communication	protein binding,transporter activity	Isoform 1 of Pannexin-1
IPI00329125.1	TRAPPC6B	X						Isoform 2 of Trafficking protein particle complex subunit 6B
IPI00296360.4	DCLK2	X	X			metabolic process,regulation of biological process,response to stimulus,cell communication	nucleotide binding,catalytic activity	Isoform 3 of Serine/threonine-protein kinase DCLK2
IPI00168728.1	IGHM		X				protein binding	FLJ00385 protein (Fragment)
IPI00410277.3	CELF2		X					Isoform 3 of CUGBP Elav-like family member 2
IPI00032371.3	GAS7		X			metabolic process,regulation of biological process	protein binding	Isoform 1 of Growth arrest-specific protein 7
IPI00166132.3	VPS52	X	X		membrane,cytoplasm,Golgi,endosome	transport		Vacuolar protein sorting-associated protein 52 homolog
IPI00017799.5	TXN2	X	X		mitochondrion,cytoplasm,organelle lumen,nucleus	metabolic process,regulation of biological process,response to stimulus,cell communication,cellular homeostasis	catalytic activity	cDNA FLJ55158, highly similar to Thioredoxin, mitochondrial
IPI00061354.5	BAZ2B			X	nucleus		protein binding,DNA binding	Isoform 2 of Bromodomain adjacent to zinc finger domain protein 2B
IPI00643348.4	COMP			X	extracellular	development	protein binding,metal ion binding	cDNA FLJ60724, highly similar to Cartilage oligomeric matrix protein
IPI00026262.3	RASA1		X	X	membrane,cytoplasm,cytosol	cell death,development,cell organization and biogenesis,metabolic process,cell division,regulation of biological process,response to stimulus,cell communication,cell differentiation	protein binding,enzyme regulator activity	Isoform 1 of Ras GTPase-activating protein 1
IPI00022143.3	ESYT1	X	X		membrane		protein binding	Isoform 1 of Extended synaptotagmin-1
IPI00165616.4	SENP8		X			metabolic process	catalytic activity	Sentrin-specific protease 8
IPI00479217.1	HNRNPU			X			protein binding	Isoform Short of Heterogeneous nuclear ribonucleoprotein U
IPI00008282.2	PDE1A			X	cytoplasm,cytosol	metabolic process,regulation of biological process,response to stimulus,cell communication,coagulation	protein binding,metal ion binding,catalytic activity	Isoform 1 of Calcium/calmodulin-dependent 3',5'-cyclic nucleotide phosphodiesterase 1A
IPI00033466.5	CLEC11A			X	extracellular,cytoskeleton,cytoplasm	cell proliferation,regulation of biological process	protein binding	C-type lectin domain family 11 member A
IPI00012303.5	SELENBP1	X	X	X	membrane,cytoplasm,organelle lumen,nucleus,cytosol	transport	protein binding	Isoform 1 of Selenium-binding protein 1
IPI00219871.5	NAA38	X			nucleus	metabolic process	protein binding,RNA binding	N-alpha-acetyltransferase 38, NatC auxiliary subunit
IPI00000828.3	PENK		X	X	extracellular	regulation of biological process,response to stimulus,cell communication,defense response	protein binding	Proenkephalin-A

IPI	GENE	Alzheimer's Hippocampus	Control hippocampus	CSF	Cellular localization	Biological process	Molecular function	Protein Description
IPI00328709.4	GREM2			X	extracellular	regulation of biological process,response to stimulus,cell communication	protein binding	Gremlin-2
IPI00477957.3	CLYBL	X	X		mitochondrion,cytoplasm	metabolic process	metal ion binding,catalytic activity	Isoform 1 of Citrate lyase subunit beta-like protein, mitochondrial
IPI00003527.5	SLC9A3R1	X	X	X	cytoskeleton,membrane,cytoplasm,nucleus	cell death,cell proliferation,cell organization and biogenesis,transport,metabolic process,regulation of biological process,response to stimulus,cellular component movement,cell communication,cellular homeostasis	protein binding	Na(+)/H(+) exchange regulatory cofactor NHE-RF1
IPI00006601.5	CHGB			X	cytoplasm			chromogranin B (secretogranin 1)
IPI00163563.3	PEBP4			X	cytoplasm,vacuole			Phosphatidylethanolamine-binding protein 4
IPI00010183.2	TTYH1	X	X					Isoform 2 of Protein tweety homolog 1
IPI00930688.1	TUBA1B	X	X		cytoskeleton,cytoplasm	cell organization and biogenesis,cell division,transport,metabolic process,cellular component movement	protein binding,nucleotide binding,structural molecule activity,catalytic activity	Tubulin alpha-1B chain
IPI00783109.1	MTSS1L	X	X			cell organization and biogenesis,regulation of biological process,response to stimulus,cell communication	protein binding	Isoform 1 of MTSS1-like protein
IPI00022542.1	ROCK1			X	cytoskeleton,membrane,cytoplasm,Golgi,cytosol	cell death,development,cell organization and biogenesis,metabolic process,cell division,transport,regulation of biological process,response to stimulus,cellular component movement,cell communication,cell differentiation	protein binding,metal ion binding,motor activity,nucleotide binding,catalytic activity	Rho-associated protein kinase 1
IPI00037448.3	GRHPR	X	X		cytoplasm,organelle lumen	cell organization and biogenesis,metabolic process	protein binding,nucleotide binding,catalytic activity	Glyoxylate reductase/hydroxypyruvate reductase
IPI00023643.6	SEMA6C			X	membrane	development,cell differentiation	protein binding,receptor activity	Isoform 1 of Semaphorin-6C
IPI00329692.3	NMT1	X	X		cytoskeleton,membrane,cytoplasm,cytosol	cell death,development,metabolic process,regulation of biological process	catalytic activity	Isoform Long of Glycylpeptide N-tetradecanoyltransferase 1
IPI00217467.3	HIST1H1E	X	X	X	chromosome,nucleus	cell organization and biogenesis,metabolic process	protein binding,DNA binding	Histone H1.4
IPI00219526.6	PGM1	X	X	X	cytoskeleton,cytoplasm,cytosol	metabolic process	metal ion binding,catalytic activity	Isoform 1 of Phosphoglucomutase-1
IPI00290744.4	FNDC9			X	membrane		protein binding	Fibronectin type III domain-containing protein 9
IPI00328195.6	NBEA	X	X		membrane,cytoplasm,cytosol		protein binding	Isoform 1 of Neurobeachin
IPI00010740.1	SFPQ	X	X		cytoplasm,organelle lumen,nucleus	metabolic process,regulation of biological process,response to stimulus	protein binding,DNA binding,RNA binding,nucleotide binding	Isoform Long of Splicing factor, proline- and glutamine-rich
IPI00183321.5	CHST15			X	membrane,cytoplasm,Golgi	metabolic process	nucleotide binding,catalytic activity	Isoform 1 of Carbohydrate sulfotransferase 15
IPI00296219.4	GLS2			X	mitochondrion,cytoplasm,organelle lumen	cell death,metabolic process,transport,regulation of biological process,cell communication	protein binding,catalytic activity	Glutaminase liver isoform, mitochondrial
IPI00329742.2	FAHD2A	X	X			metabolic process	metal ion binding,catalytic activity	Fumarylacetoacetate hydrolase domain-containing protein 2A
IPI00218345.5	TUBA3C			X	cytoskeleton	cell organization and biogenesis,metabolic process,cellular component movement	nucleotide binding,structural molecule activity,catalytic activity	Isoform 2 of Tubulin alpha-3C/D chain
IPI00013978.1	SPOP			X	nucleus	metabolic process	protein binding	Speckle-type POZ protein
IPI00166622.10	TMEM132C			X	membrane			Transmembrane protein 132C
IPI00166392.2	CADM1			X				Isoform 2 of Cell adhesion molecule 1
IPI00014219.1	SNX17		X		membrane,cytoplasm,Golgi,cytosol,endosome	cell organization and biogenesis,metabolic process,transport,regulation of biological process,response to stimulus,cell communication	protein binding	Sorting nexin-17
