# Supplementary material for: Semiquantitative proteomic analysis of human hippocampal tissues from Alzheimer’s disease and age-matched control brains
Source: Clin Proteomics. 2013 May 1;10(1):5. doi: 10.1186/1559-0275-10-5 (PMC3648498; doi:10.1186/1559-0275-10-5)
Supplement: Additional file 2 — List of over-represented pathways predicted by Protein Center Pathway analysis. [file 1559-0275-10-5-S2.pdf]

Additional table 2: List of over-represented pathways predicted by Protein Center Pathway analysis plugin. Proteins identified in Alzheimer's tissues were compared against human proteome database to calculate the enrichment. Ref.Count represents the total number of proteins involved in the corresponding pathway, whereas Count represents the number of proteins identified in AD tissues in the current study.

Description	Count*	Ref. Count#	p-value
Ribosome (hsa03010)	47	83	2.11E-20
Parkinson's disease (hsa05012)	45	117	1.21E-11
Valine, leucine and isoleucine degradation (hsa00280)	23	40	8.59E-11
Oxidative phosphorylation (hsa00190)	43	121	6.83E-10
Alzheimer's disease (hsa05010)	49	157	6.90E-09
Pathogenic Escherichia coli infection (hsa05130)	23	49	1.66E-08
Citrate cycle (TCA cycle) (hsa00020)	17	29	1.77E-08
Metabolic pathways (hsa01100)	204	1068	1.94E-08
Proteasome (hsa03050)	20	41	6.44E-08
Glyoxylate and dicarboxylate metabolism (hsa00630)	14	24	3.66E-07
Huntington's disease (hsa05016)	47	170	8.26E-07
Propanoate metabolism (hsa00640)	15	30	1.99E-06
Fatty acid metabolism (hsa00071)	17	40	6.89E-06
Pyruvate metabolism (hsa00620)	17	40	6.89E-06
beta-Alanine metabolism (hsa00410)	12	24	2.17E-05
Protein processing in endoplasmic reticulum (hsa04141)	40	156	3.84E-05
Aminoacyl-tRNA biosynthesis (hsa00970)	15	39	9.70E-05
Vasopressin-regulated water reabsorption (hsa04962)	15	41	1.88E-04
Glycolysis / Gluconeogenesis (hsa00010)	19	59	1.95E-04
Regulation of actin cytoskeleton (hsa04810)	44	190	2.14E-04
Arginine and proline metabolism (hsa00330)	17	51	2.63E-04
Legionellosis (hsa05134)	17	51	2.63E-04
Proximal tubule bicarbonate reclamation (hsa04964)	10	22	2.92E-04
Dopaminergic synapse (hsa04728)	30	116	3.00E-04
Synaptic vesicle cycle (hsa04721)	19	61	3.17E-04
Salmonella infection (hsa05132)	23	82	4.32E-04
Glycine, serine and threonine metabolism (hsa00260)	13	36	5.80E-04
GABAergic synapse (hsa04727)	23	84	6.29E-04

Description	Count*	Ref. Count#	p-value
Adherens junction (hsa04520)	20	70	7.78E-04
Phagosome (hsa04145)	32	137	1.30E-03
Alanine, aspartate and glutamate metabolism (hsa00250)	11	30	1.32E-03
Shigellosis (hsa05131)	17	58	1.39E-03
Butanoate metabolism (hsa00650)	10	26	1.43E-03
Bacterial invasion of epithelial cells (hsa05100)	18	64	1.71E-03
Renal cell carcinoma (hsa05211)	18	64	1.71E-03
Thyroid cancer (hsa05216)	10	27	2.00E-03
Glutathione metabolism (hsa00480)	13	42	2.92E-03
Long-term potentiation (hsa04720)	17	62	3.06E-03
Cysteine and methionine metabolism (hsa00270)	11	33	3.18E-03
Vitamin B6 metabolism (hsa00750)	4	6	4.12E-03
Fc gamma R-mediated phagocytosis (hsa04666)	21	85	4.21E-03
Amino sugar and nucleotide sugar metabolism (hsa00520)	13	44	4.58E-03
Fatty acid elongation (hsa00062)	8	21	4.60E-03
Gap junction (hsa04540)	20	82	6.02E-03
Prion diseases (hsa05020)	10	31	6.31E-03
Cardiac muscle contraction (hsa04260)	18	72	6.82E-03
Focal adhesion (hsa04510)	36	179	9.63E-03
Histidine metabolism (hsa00340)	8	24	1.14E-02
Alcoholism (hsa05034)	28	134	1.27E-02
Vibrio cholerae infection (hsa05110)	13	50	1.44E-02
Phenylalanine metabolism (hsa00360)	6	16	1.51E-02
Retrograde endocannabinoid signaling (hsa04723)	21	95	1.57E-02
Pancreatic secretion (hsa04972)	20	90	1.71E-02
Long-term depression (hsa04730)	15	62	1.74E-02
