# Supplementary material for: Semiquantitative proteomic analysis of human hippocampal tissues from Alzheimer’s disease and age-matched control brains
Source: Clin Proteomics. 2013 May 1;10(1):5. doi: 10.1186/1559-0275-10-5 (PMC3648498; doi:10.1186/1559-0275-10-5)
Supplement: Additional file 3 — List of 40 CSF proteins that were found exclusively in Alzheimer's hippocampal tissues. [file 1559-0275-10-5-S3.pdf]

Addtional table 3: List of 40 CSF proteins that were found exlcusively in Alzheimer's hippocampal tissues. Gene Ontology information was retrived from Protein Center.

GENE	Protein description	Cellular localization	Biological process	Molecular function
ACAN	aggrecan core protein isoform 2 precursor	extracellular	development,cell organization and biogenesis,metabolic process,regulation of biological process,response to stimulus,cellular component movement,cell differentiation	protein binding,metal ion binding
PTN	Pleiotrophin	extracellular,endoplasmic reticulum,cytoplasm	cell proliferation,development,cell division,regulation of biological process,response to stimulus,cell communication	protein binding,enzyme regulator activity
SEMA4C	Semaphorin-4C	cytoskeleton,membrane,cytoplasm	development,regulation of biological process,response to stimulus,cellular component movement,cell communication,cell differentiation	protein binding,receptor activity
ENDOD1	Endonuclease domain-containing 1 protein	extracellular	metabolic process	metal ion binding,catalytic activity
PI16	Isoform 1 of Peptidase inhibitor 16	extracellular,membrane	metabolic process,regulation of biological process	enzyme regulator activity
BID	Isoform 1 of BH3-interacting domain death agonist	mitochondrion,membrane,cytoplasm,cytosol	cell death,cell proliferation,development,cell organization and biogenesis,transport,regulation of biological process,response to stimulus,cell communication	protein binding
EFEMP1	Isoform 1 of EGF-containing fibulin-like extracellular matrix protein 1	extracellular,cell surface,membrane	development,metabolic process,regulation of biological process,response to stimulus,cell communication,cell differentiation	protein binding,signal transducer activity,metal ion binding,receptor activity,catalytic activity
CREG1	Protein CREG1	extracellular,organelle lumen,nucleus	cell proliferation,development,metabolic process,regulation of biological process	protein binding,nucleotide binding,catalytic activity
SUMF2	sulfatase-modifying factor 2 isoform b precursor			
LAMP1	Lysosome-associated membrane glycoprotein 1	cell surface,membrane,cytoplasm,vacuole,endosome	cell death,metabolic process	
SEMA7A	Semaphorin-7A	cell surface,membrane	cell organization and biogenesis,development,regulation of biological process,response to stimulus,cell communication,defense response,cell growth,cell differentiation	protein binding,receptor activity

GENE	Protein description	Cellular localization	Biological process	Molecular function
SCG5	Isoform 1 of Neuroendocrine protein 7B2	extracellular,cytoplasm	metabolic process,transport,regulation of biological process,response to stimulus,cell communication	protein binding,nucleotide binding,enzyme regulator activity
SPARC	Secreted protein, acidic, cysteine-rich (Osteonectin), isoform CRA_a	extracellular,cytoplasm,nucleus	cell proliferation,development,regulation of biological process,response to stimulus,cell communication	protein binding,metal ion binding
VASN	Vasorin	extracellular,membrane		protein binding
ADRM1	Proteasomal ubiquitin receptor ADRM1	membrane,cytoplasm,proteasome,organelle lumen,nucleus	cell organization and biogenesis,metabolic process,regulation of biological process	protein binding,enzyme regulator activity
IGSF1	Isoform 1 of Immunoglobulin superfamily member 1	extracellular,membrane	metabolic process,regulation of biological process,response to stimulus,cell communication	signal transducer activity,protein binding,receptor activity
LYPLA1	cDNA FLJ60607, highly similar to Acyl-protein thioesterase 1	mitochondrion,cytoplasm		catalytic activity
CD14	Monocyte differentiation antigen CD14	extracellular,cell surface,membrane,cytoplasm,endosome	cell death,cell organization and biogenesis,transport,regulation of biological process,response to stimulus,defense response,cell communication	signal transducer activity,protein binding,receptor activity
SNX12	Isoform 1 of Sorting nexin-12	membrane	transport,cell communication	protein binding
NID2	Isoform 1 of Nidogen-2	extracellular,cell surface,membrane		protein binding,metal ion binding
LMAN2	Vesicular integral-membrane protein VIP36	membrane,endoplasmic reticulum,cytoplasm,Golgi	transport	metal ion binding
DDRGK1	Isoform 1 of DDRGK domain-containing protein 1	endoplasmic reticulum,cytoplasm		protein binding
PODXL2	Isoform 1 of Podocalyxin-like protein 2	membrane	cellular component movement	protein binding
CTSH	Pro-cathepsin H	extracellular,cytoplasm,vacuole,cytosol	cell death,cell proliferation,development,metabolic process,regulation of biological process,response to stimulus,cellular component movement,cell communication	protein binding,signal transducer activity,receptor activity,catalytic activity,enzyme regulator activity
SDC4	Syndecan-4	extracellular,cell surface,membrane,cytoplasm,Golgi,vacuole,organelle lumen	development,cell organization and biogenesis,metabolic process,regulation of biological process	protein binding,signal transducer activity,receptor activity
TXNDC5	Thioredoxin domain-containing protein 5	endoplasmic reticulum,cytoplasm,organelle lumen,vacuole	cell death,cell organization and biogenesis,transport,metabolic process,regulation of biological process,cellular homeostasis	catalytic activity
NLN	Neurolysin, mitochondrial	mitochondrion,cytoplasm	metabolic process	metal ion binding,catalytic activity
HS6ST2	Isoform 1 of Heparan-sulfate 6-O-sulfotransferase 2	membrane,cytoplasm,Golgi	metabolic process	catalytic activity

GENE	Protein description	Cellular localization	Biological process	Molecular function
LAMC1	Laminin subunit gamma-1	extracellular,cytoskeleton,organelle lumen,chromosome,nucleus	cell proliferation,development,cell organization and biogenesis,regulation of biological process,response to stimulus,cellular component movement,reproduction,cell differentiation	protein binding,motor activity,structural molecule activity,catalytic activity
PHOSPHO2	Pyridoxal phosphate phosphatase PHOSPHO2		metabolic process	metal ion binding,catalytic activity
LAMA5	Laminin subunit alpha-5	extracellular,cytoskeleton,membrane,cytoplasm	cell proliferation,development,cell organization and biogenesis,transport,regulation of biological process,response to stimulus,cellular component movement,cell communication,cell differentiation	protein binding,motor activity,structural molecule activity,catalytic activity
LGI3	Leucine-rich repeat LGI family member 3	extracellular,cytoplasm	transport	protein binding
CDH4	Cadherin-4	membrane	cell organization and biogenesis,development,regulation of biological process,response to stimulus,cell growth,cell differentiation	metal ion binding
PODXL	Podocalyxin-like isoform 2 precursor	membrane	regulation of biological process,cellular component movement	catalytic activity
COL6A1	Collagen alpha-1(VI) chain	extracellular,membrane,endoplasmic reticulum,cytoplasm,organelle lumen	cell organization and biogenesis,development,response to stimulus,cell differentiation	protein binding
SCN1B	Sodium channel subunit beta-1	extracellular,membrane	development,cell organization and biogenesis,transport,regulation of biological process,response to stimulus,cell communication,cellular homeostasis,cell differentiation	transporter activity
S100A6	Protein S100-A6	membrane,cytoplasm,cytosol,nucleus	cell proliferation,cell organization and biogenesis,development,transport,regulation of biological process,response to stimulus,cell communication,cell differentiation	protein binding,transporter activity,metal ion binding
CNDP1	Beta-Ala-His dipeptidase	extracellular	metabolic process	metal ion binding,catalytic activity
IL6ST	Isoform 1 of Interleukin-6 receptor subunit beta	extracellular,cell surface,membrane	cell proliferation,development,metabolic process,regulation of biological process,response to stimulus,cellular component movement,cell communication,defense response,cellular homeostasis,cell differentiation	protein binding,signal transducer activity,receptor activity

GENE	Protein description	Cellular localization	Biological process	Molecular function
LAMA2	laminin subunit alpha-2 isoform b precursor	extracellular,cytoskeleton,membrane,mitochondrion,cytoplasm,organelle lumen,chromosome,cytosol,nucleus	development,cell organization and biogenesis,metabolic process,transport,regulation of biological process,response to stimulus,cellular component movement,cell communication,reproduction	signal transducer activity,protein binding,motor activity,catalytic activity
