# Supplementary material for: Semiquantitative proteomic analysis of human hippocampal tissues from Alzheimer’s disease and age-matched control brains
Source: Clin Proteomics. 2013 May 1;10(1):5. doi: 10.1186/1559-0275-10-5 (PMC3648498; doi:10.1186/1559-0275-10-5)
Supplement: Additional file 4 — One hundred six CSF proteins that were identified exclusively in Control tissue pools. [file 1559-0275-10-5-S4.pdf]

Additional table 4: One hundred six CSF proteins that were identified exclusively in Control tissue pools. Gene ontology information was retrived from Protein Center.

GENE	Protein Description	Cellular localization	Biological process	Molecular function
FBXO21	Isoform 2 of F-box only protein 21			protein binding,DNA binding
HIST1H1C	Histone H1.2	chromosome,nucleus	cell organization and biogenesis,metabolic process	DNA binding
LUM	Lumican	extracellular,cytoplasm,Golgi,vacuole,organelle lumen	development,cell organization and biogenesis,metabolic process,regulation of biological process,response to stimulus	protein binding,structural molecule activity
FXYD1	Phospholemman	membrane	transport,cellular homeostasis	transporter activity
GPC1	Glypican-1	extracellular,membrane,cytoplasm,Golgi,vacuole,organelle lumen,endosome	cell organization and biogenesis,development,metabolic process,regulation of biological process,response to stimulus,cell communication,cellular homeostasis,cell differentiation	protein binding,metal ion binding
MCCC1	Methylcrotonoyl-CoA carboxylase subunit alpha, mitochondrial	membrane,mitochondrion,cytoplasm,organelle lumen	metabolic process	metal ion binding,nucleotide binding,catalytic activity
DAAM2	Disheveled-associated activator of morphogenesis 2		cell organization and biogenesis	protein binding
KRT9	Keratin, type I cytoskeletal 9	cytoskeleton,cytoplasm	cell organization and biogenesis,development,reproduction	structural molecule activity
PPFIA4	Protein tyrosine phosphatase, receptor type, f polypeptide (PTPRF), interacting protein (Liprin), alpha 4	extracellular,membrane,mitochondrion,cytoplasm	cell organization and biogenesis,metabolic process,transport	protein binding
VTN	Vitronectin	extracellular,membrane	cell organization and biogenesis,transport,metabolic process,regulation of biological process,response to stimulus,cellular component movement,cell communication,coagulation	protein binding,receptor activity
MCFD2	Multiple coagulation factor deficiency protein 2	extracellular,membrane,endoplasmic reticulum,cytoplasm,Golgi	metabolic process,transport	metal ion binding
EHD2	EH domain-containing protein 2	membrane,cytoplasm,nucleus,endosome	cell organization and biogenesis,transport,metabolic process,response to stimulus,coagulation	protein binding,metal ion binding,nucleotide binding,catalytic activity
PSMB6	Proteasome subunit beta type-6	proteasome,cytoplasm,organelle lumen,cytosol,nucleus	cell death,development,metabolic process,regulation of biological process,response to stimulus,cell communication,reproduction	catalytic activity
TMEM132B	Isoform 1 of Transmembrane protein 132B	membrane		
TM9SF4	Transmembrane 9 superfamily member 4	membrane		

GENE	Protein Description	Cellular localization	Biological process	Molecular function
C10orf35	Uncharacterized protein C10orf35	membrane		
ROBO1	Uncharacterized protein			protein binding
CPVL	Probable serine carboxypeptidase CPVL		metabolic process	catalytic activity
IGHD	Isoform 2 of Ig delta chain C region			
PGAM1	Similar to Phosphoglycerate mutase 1			
EFR3A	Isoform 3 of Protein EFR3 homolog A			
ARF1	ADP-ribosylation factor 1	membrane,cytoplasm,Golgi,nucleus,cytosol	development,cell organization and biogenesis,transport,metabolic process,regulation of biological process,response to stimulus,cell communication,defense response,cellular homeostasis	signal transducer activity,protein binding,metal ion binding,nucleotide binding,catalytic activity,enzyme regulator activity
TMEM132A	Isoform 1 of Transmembrane protein 132A	membrane,endoplasmic reticulum,cytoplasm,Golgi		
C1QC	Complement C1q subcomponent subunit C	extracellular,membrane	development,metabolic process,regulation of biological process,response to stimulus,defense response,cell differentiation	protein binding
ITGB8	Integrin beta-8	membrane	development,metabolic process,regulation of biological process,response to stimulus,cell communication	protein binding,receptor activity
NRN1	Neuritin	membrane	development	
EFNB2	Ephrin-B2	membrane	development,regulation of biological process,response to stimulus,cellular component movement,cell communication,reproduction,cell differentiation	protein binding
ACP2	Lysosomal acid phosphatase	membrane,cytoplasm,organelle lumen,vacuole	cell death,development,cell organization and biogenesis,response to stimulus	protein binding,catalytic activity
SEL1L	Isoform 1 of Protein sel-1 homolog 1	membrane,endoplasmic reticulum,cytoplasm	regulation of biological process,response to stimulus,cell communication	protein binding
GPC5	Glypican-5	extracellular,membrane,cytoplasm,Golgi,vacuole,organelle lumen	metabolic process	protein binding
APOC3	Apolipoprotein C-III	extracellular	cell organization and biogenesis,transport,metabolic process,regulation of biological process,response to stimulus,cell communication	protein binding,enzyme regulator activity
LYNX1	Ly-6/neurotoxin-like protein 1			

GENE	Protein Description	Cellular localization	Biological process	Molecular function
ITM2C	Isoform 1 of Integral membrane protein 2C	membrane,cytoplasm,Golgi,vacuole	cell death,cell organization and biogenesis,development,regulation of biological process,cell differentiation	protein binding,nucleotide binding
ERP44	Endoplasmic reticulum resident protein 44	membrane,endoplasmic reticulum,cytoplasm,organelle lumen	metabolic process,regulation of biological process,response to stimulus,cellular homeostasis	metal ion binding,catalytic activity
SPTB	Isoform 2 of Spectrin beta chain, erythrocyte			protein binding
SCN2B	Sodium channel subunit beta-2	membrane	development,transport,regulation of biological process,response to stimulus,cell communication,cellular homeostasis	protein binding,transporter activity
APOC1	Apolipoprotein C-I	extracellular,endoplasmic reticulum,cytoplasm	cell organization and biogenesis,metabolic process,transport,regulation of biological process	enzyme regulator activity
FERMT3	Isoform 2 of Fermitin family homolog 3			protein binding
IGLON5	IgLON family member 5	extracellular		protein binding
ZCCHC11	Isoform 1 of Terminal uridylyltransferase 4	cytoplasm,organelle lumen,nucleus	metabolic process,regulation of biological process,response to stimulus,cell differentiation	protein binding,metal ion binding,catalytic activity
RPL24	60S ribosomal protein L24	cytoplasm,ribosome,cytosol	cell organization and biogenesis,metabolic process,transport,reproduction	RNA binding,structural molecule activity
C16orf45	Uncharacterized protein C16orf45			
CLEC3B	Tetranectin	extracellular,cytoplasm,organelle lumen,nucleus	development,metabolic process,regulation of biological process,response to stimulus	protein binding,metal ion binding
ADAM11	Isoform Long of Disintegrin and metalloproteinase domain-containing protein 11	membrane	metabolic process,regulation of biological process,response to stimulus,cell communication	protein binding,metal ion binding,catalytic activity
FCGBP	IgGFc-binding protein	extracellular,cytoplasm		protein binding
SOD3	Extracellular superoxide dismutase [Cu-Zn]	extracellular,mitochondrion,cytoplasm,nucleus,cytosol	metabolic process,response to stimulus	antioxidant activity,protein binding,metal ion binding,catalytic activity
ORM1	Alpha-1-acid glycoprotein 1	extracellular	transport,regulation of biological process,response to stimulus,defense response	protein binding
PCLO	Isoform 1 of Protein piccolo	cytoskeleton,membrane,cytoplasm	cell organization and biogenesis,transport,regulation of biological process,response to stimulus,cell communication	protein binding,transporter activity,metal ion binding
DCD	Dermcidin	extracellular	metabolic process,response to stimulus,defense response	catalytic activity
RANBP6	Ran-binding protein 6	cytoplasm,nucleus	transport	protein binding

GENE	Protein Description	Cellular localization	Biological process	Molecular function
RASA1	Isoform 1 of Ras GTPase-activating protein 1	membrane,cytoplasm,cytosol	cell death,cell organization and biogenesis,development,metabolic process,cell division,regulation of biological process,response to stimulus,cell communication,cell differentiation	protein binding,enzyme regulator activity
NELL2	Protein kinase C-binding protein NELL2	extracellular		protein binding,metal ion binding,structural molecule activity
F13A1	Coagulation factor XIII A chain	extracellular,cytoplasm,organelle lumen	metabolic process,transport,response to stimulus,coagulation	metal ion binding,catalytic activity
HAPLN4	Hyaluronan and proteoglycan link protein 4	extracellular		protein binding
NME3	Nucleoside diphosphate kinase 3	mitochondrion,cytoplasm	cell death,metabolic process,regulation of biological process	metal ion binding,nucleotide binding,catalytic activity
GRIA4	Glutamate receptor 4	cytoskeleton,membrane,cytoplasm	transport,regulation of biological process,response to stimulus,cell communication	signal transducer activity,transporter activity,receptor activity
A1BG	Isoform 1 of Alpha-1B-glycoprotein	extracellular		protein binding
LPHN1	Isoform 1 of Latrophilin-1	cytoskeleton,membrane	development,regulation of biological process,response to stimulus,cell communication	protein binding,signal transducer activity,receptor activity
PTPRN	Receptor-type tyrosine-protein phosphatase-like N	membrane	metabolic process,regulation of biological process,response to stimulus,cell communication	protein binding,signal transducer activity,receptor activity,catalytic activity
PCSK2	Neuroendocrine convertase 2	extracellular,membrane,cytoplasm	development,metabolic process	protein binding,catalytic activity
GPR158	Probable G-protein coupled receptor 158	membrane	regulation of biological process,response to stimulus,cell communication	signal transducer activity,receptor activity
NID1	Isoform 1 of Nidogen-1	extracellular,cell surface,membrane	development,cell organization and biogenesis,regulation of biological process	protein binding,metal ion binding
NRP2	Isoform A22 of Neuropilin-2	membrane	cell proliferation,development,cell organization and biogenesis,regulation of biological process,response to stimulus,cellular component movement,cell communication,cell differentiation	protein binding,signal transducer activity,metal ion binding,receptor activity,catalytic activity

GENE	Protein Description	Cellular localization	Biological process	Molecular function
EPHA5	EPHA5 protein	membrane	metabolic process,regulation of biological process,response to stimulus,cell communication	protein binding,signal transducer activity,nucleotide binding,receptor activity,catalytic activity
SYN1	Isoform IB of Synapsin-1	cytoplasm	transport,cell communication	
CYFIP1	Isoform 1 of Cytoplasmic FMR1-interacting protein 1	cytoplasm	development,cell organization and biogenesis,regulation of biological process,cell growth,cell differentiation	protein binding
SCN3B	Sodium channel subunit beta-3	membrane,cytoplasm	development,cell organization and biogenesis,transport,regulation of biological process,response to stimulus,cell communication,cellular homeostasis,cell differentiation	protein binding,transporter activity
NTRK2	Isoform TrkB of BDNF/NT-3 growth factors receptor	cell surface,cytoskeleton,membrane,cytoplasm,Golgi,cytosol,endosome	cell death,cell proliferation,development,cell organization and biogenesis,transport,metabolic process,regulation of biological process,response to stimulus,cellular component movement,cell communication,cell differentiation	protein binding,signal transducer activity,nucleotide binding,receptor activity,catalytic activity
NLGN4Y	Isoform 1 of Neuroligin-4, Y-linked	cell surface,cytoskeleton,membrane	cell organization and biogenesis,development,metabolic process	protein binding,receptor activity,catalytic activity
C2	cDNA FLJ55673, highly similar to Complement factor B		metabolic process	protein binding,catalytic activity
PENK	Proenkephalin-A	extracellular	regulation of biological process,response to stimulus,cell communication,defense response	protein binding
CLIC6	Isoform A of Chloride intracellular channel protein 6			
SPON1	Spondin-1	extracellular		protein binding
CRELD1	Isoform 2 of Cysteine-rich with EGF-like domain protein 1			metal ion binding
MXRA7	Isoform 2 of Matrix-remodeling-associated protein 7			
ACYP1	Acylphosphatase-1		metabolic process	catalytic activity
PLG	Plasminogen	extracellular,cell surface,membrane,cytoplasm,organelle lumen	cell proliferation,cell organization and biogenesis,metabolic process,transport,regulation of biological process,response to stimulus,coagulation	protein binding,metal ion binding,catalytic activity

GENE	Protein Description	Cellular localization	Biological process	Molecular function
CHGB	cDNA FLJ58131, highly similar to Secretogranin-1	cytoplasm		
RIMBP2	Isoform 1 of RIMS-binding protein 2	membrane	metabolic process,regulation of biological process	protein binding
GBA	cDNA FLJ56157, highly similar to Glucosylceramidase	membrane,cytoplasm,vacuole	cell organization and biogenesis,metabolic process	catalytic activity
NPTX2	Neuronal pentraxin-2	extracellular	cell communication	metal ion binding
CST3	Cystatin-C	extracellular	cell organization and biogenesis,development,metabolic process,regulation of biological process,response to stimulus,defense response	protein binding,enzyme regulator activity
LGI1	Isoform 1 of Leucine-rich glioma-inactivated protein 1	extracellular	cell proliferation,development,cell organization and biogenesis,regulation of biological process,response to stimulus,cell communication,cell growth,cell differentiation	protein binding
PRELP	Prolargin	extracellular,cytoplasm,Golgi,vacuole,organelle lumen	development,metabolic process	protein binding,structural molecule activity
GSS	Glutathione synthetase	cytoplasm,cytosol	development,metabolic process,response to stimulus	protein binding,metal ion binding,nucleotide binding,catalytic activity
HBS1L	Isoform 1 of HBS1-like protein		metabolic process,regulation of biological process,response to stimulus,cell communication	RNA binding,nucleotide binding,catalytic activity
AIMP1	Aminoacyl tRNA synthase complex-interacting multifunctional protein 1	extracellular,endoplasmic reticulum,cytoplasm,Golgi,cytosol,nucleus	cell death,cell proliferation,development,metabolic process,regulation of biological process,response to stimulus,cellular component movement,defense response,cell communication	protein binding,RNA binding
RYR2	Isoform 1 of Ryanodine receptor 2	membrane,endoplasmic reticulum,cytoplasm	cell death,development,transport,regulation of biological process,response to stimulus,cell communication,cellular homeostasis	protein binding,transporter activity,metal ion binding
SNX3	Isoform 2 of Sorting nexin-3		cell communication	protein binding
CACNA2D2	Isoform 3 of Voltage-dependent calcium channel subunit alpha-2/delta-2			protein binding
PTPRF	Isoform 1 of Receptor-type tyrosine-protein phosphatase F	membrane	metabolic process,regulation of biological process,response to stimulus,cell communication	protein binding,signal transducer activity,receptor activity,catalytic activity

GENE	Protein Description	Cellular localization	Biological process	Molecular function
SYT11	Synaptotagmin-11	membrane,cytoplasm	transport	protein binding,transporter activity,metal ion binding
CSPG5	Isoform 1 of Chondroitin sulfate proteoglycan 5	extracellular,membrane,endoplasmic reticulum,cytoplasm,Golgi,organelle lumen,vacuole	development,transport,metabolic process,regulation of biological process,cell communication,cell differentiation	protein binding
ERC1	Isoform 2 of ELKS/Rab6-interacting/CAST family member 1	membrane	metabolic process,regulation of biological process	
PLBD2	Putative phospholipase B-like 2	cytoplasm,organelle lumen,vacuole	metabolic process	catalytic activity
SERPINA3	Isoform 2 of Alpha-1-antichymotrypsin			enzyme regulator activity
MESDC2	LDLR chaperone MESD	membrane,endoplasmic reticulum,cytoplasm	metabolic process,regulation of biological process,response to stimulus,cell communication	protein binding
SCO2	Thymidine phosphorylase	cytoplasm,cytosol	cell organization and biogenesis,development,metabolic process,response to stimulus,cell differentiation	protein binding,catalytic activity
SELM	Selenoprotein M	cytoplasm,Golgi	metabolic process	metal ion binding,catalytic activity
CRABP1	Cellular retinoic acid-binding protein 1	cytoplasm	development,transport,regulation of biological process,response to stimulus,cell communication	transporter activity
SEZ6L2	Isoform 3 of Seizure 6-like protein 2	membrane		signal transducer activity
CDH11	Isoform 2 of Cadherin-11	membrane		metal ion binding
LBP	Lipopolysaccharide-binding protein	extracellular	metabolic process,transport,regulation of biological process,response to stimulus,cellular component movement,cell communication,defense response	protein binding
HNRNPH2	Heterogeneous nuclear ribonucleoprotein H2	cytoskeleton,cytoplasm,organelle lumen,nucleus	metabolic process	RNA binding,nucleotide binding
LMBRD2	LMBR1 domain-containing protein 2	membrane		
RILPL1	Isoform 1 of RILP-like protein 1	membrane,cytoplasm,organelle lumen,cytosol,nucleus	cell death,regulation of biological process	
