# Supplementary material for: Semiquantitative proteomic analysis of human hippocampal tissues from Alzheimer’s disease and age-matched control brains
Source: Clin Proteomics. 2013 May 1;10(1):5. doi: 10.1186/1559-0275-10-5 (PMC3648498; doi:10.1186/1559-0275-10-5)
Supplement: Additional file 5 — Up-regulated proteins in Alzheimer's (AD) tissues in comparison to Control tissues. [file 1559-0275-10-5-S5.pdf]

**Additional table 5: Upregulated proteins in Alzheimer's (AD) tissues in comparison to Control tissues. A cut-off of 1.5-fold change and a minimum of 2-peptide hit identification was used.**

| Gene/Protein description | IPI accession number | Molecular weight | Spectral counts 'AD' | Spectral counts 'Control' | Fold-change |
|---|---|---|---|---|---|
| MSN Moesin | IPI00219365 (+1) | 68 kDa | 48.014 | 8.7611 | 5.480362055 |
| HSPB1 Heat shock protein beta-1 | IPI00025512 | 23 kDa | 41.506 | 8.1155 | 5.114410696 |
| S100B Protein S100-B | IPI00299399 | 11 kDa | 197.88 | 39.578 | 4.999747334 |
| CLIC1 Chloride intracellular channel protein 1 | IPI00010896 | 27 kDa | 29.57 | 6.2272 | 4.74852261 |
| DDAH2 N(G),N(G)-dimethylarginine dimethylaminohydrolase 2 | IPI00000760 | 30 kDa | 34.674 | 8.2401 | 4.207958641 |
| RDX Radixin, isoform CRA_a | IPI00017367 (+1) | 71 kDa | 41.154 | 11.014 | 3.73651716 |
| NDRG2 Isoform 1 of Protein NDRG2 | IPI00008994 | 41 kDa | 229.94 | 67.604 | 3.401278031 |
| PRDX6 Peroxiredoxin-6 | IPI00220301 | 25 kDa | 154.51 | 45.81 | 3.372844357 |
| EPHX1 Epoxide hydrolase 1 | IPI00009896 | 53 kDa | 28.37 | 8.7142 | 3.255605793 |
| AHCY Adenosylhomocysteinase | IPI00012007 | 48 kDa | 16.93 | 5.2229 | 3.241494189 |
| PLCD1 1-phosphatidylinositol-4,5-bisphosphate phosphodiesterase delta-1 | IPI00746030 | 86 kDa | 21.136 | 6.6492 | 3.178728268 |
| LMNA Isoform A of Lamin-A/C | IPI00021405 (+2) | 74 kDa | 68.96 | 21.927 | 3.144981074 |
| MAOB Amine oxidase [flavin-containing] B | IPI00328156 | 59 kDa | 44.478 | 15.107 | 2.944198054 |
| GSTM1 Isoform 1 of Glutathione S-transferase Mu 1 | IPI00218831 | 26 kDa | 16.927 | 5.782 | 2.927533725 |
| GLTP Glycolipid transfer protein | IPI00184363 | 24 kDa | 30.552 | 10.542 | 2.898121799 |
| GJA1 Gap junction alpha-1 protein | IPI00218487 | 43 kDa | 21.713 | 7.5632 | 2.870874762 |
| GSTP1 Glutathione S-transferase P | IPI00219757 | 23 kDa | 152.92 | 53.406 | 2.863348687 |
| PRDX1 Peroxiredoxin-1 | IPI00000874 | 22 kDa | 106.53 | 38.109 | 2.795402661 |
| GFAP Isoform 1 of Glial fibrillary acidic protein | IPI00025363 | 50 kDa | 2887.7 | 1046.5 | 2.759388438 |
| ENPP6 Ectonucleotide pyrophosphatase/phosphodiesterase family member 6 | IPI00157414 | 50 kDa | 15.503 | 5.6318 | 2.752761107 |
| VAT1 Synaptic vesicle membrane protein VAT-1 homolog | IPI00156689 | 42 kDa | 34.808 | 12.707 | 2.739277564 |
| STMN1 Stathmin | IPI00479997 | 17 kDa | 18.812 | 7.0883 | 2.653950877 |
| LGALS1 Galectin-1 | IPI00219219 | 15 kDa | 54.311 | 22.169 | 2.44986242 |
| ESD S-formylglutathione hydrolase | IPI00411706 | 31 kDa | 33.515 | 13.697 | 2.446886179 |
| SEPT9 septin 9 isoform b | IPI00784808 | 65 kDa | 32.339 | 13.442 | 2.405817587 |
| - 104 kDa protein | IPI00791534 (+1) | 104 kDa | 14.691 | 6.2245 | 2.360189573 |
| EZR Ezrin | IPI00843975 (+1) | 69 kDa | 27.743 | 11.762 | 2.3586975 |
| HAGH Isoform 1 of Hydroxyacylglutathione hydrolase, mitochondrial | IPI00003933 (+1) | 34 kDa | 12.293 | 5.3209 | 2.310323442 |
| VIM Vimentin | IPI00418471 | 54 kDa | 309.62 | 136.13 | 2.274443547 |
| VCL Isoform 1 of Vinculin | IPI00291175 (+1) | 117 kDa | 28.036 | 12.359 | 2.268468323 |
| CRYAB Alpha-crystallin B chain | IPI00021369 | 20 kDa | 41.727 | 18.496 | 2.256001298 |
| CAPN2 Calpain-2 catalytic subunit | IPI00289758 | 80 kDa | 44.345 | 19.904 | 2.227944132 |
| GRHPR Glyoxylate reductase/hydroxypyruvate reductase | IPI00037448 | 36 kDa | 13.221 | 6.0103 | 2.199723807 |
| PADI2 Protein-arginine deiminase type-2 | IPI00294187 | 76 kDa | 82.687 | 37.757 | 2.189978017 |
| PHGDH D-3-phosphoglycerate dehydrogenase | IPI00011200 | 57 kDa | 49.772 | 22.801 | 2.182886715 |
| GSTM2 Glutathione S-transferase Mu 2 | IPI00219067 (+2) | 26 kDa | 34.116 | 15.802 | 2.158967219 |
| PGD 6-phosphogluconate dehydrogenase, decarboxylating | IPI00219525 | 53 kDa | 43.241 | 20.151 | 2.145848841 |
| MAG Myelin-associated glycoprotein | IPI00026237 | 69 kDa | 16.786 | 7.8647 | 2.134347146 |
| CLU Isoform 1 of Clusterin | IPI00291262 (+3) | 52 kDa | 16.544 | 7.7709 | 2.128968331 |
| CDC42 Isoform 2 of Cell division control protein 42 homolog | IPI00016786 (+1) | 21 kDa | 17.849 | 8.4084 | 2.122758194 |
| CAT Catalase | IPI00465436 | 60 kDa | 10.976 | 5.1754 | 2.120802257 |
| ENO2 Gamma-enolase | IPI00216171 | 47 kDa | 223.46 | 106.55 | 2.097231347 |

| Gene/Protein description | IPI accession number | Molecular weight | Spectral counts 'AD' | Spectral counts 'Control' | Fold-change |
|---|---|---|---|---|---|
| TPPP Tubulin polymerization-promoting protein | IPI00013043 | 24 kDa | 72.383 | 34.916 | 2.073061061 |
| TKT cDNA FLJ54957, highly similar to Transketolase | IPI00643920 (+1) | 69 kDa | 48.538 | 23.49 | 2.066326096 |
| CP Ceruloplasmin | IPI00017601 | 122 kDa | 19.366 | 9.3834 | 2.06385745 |
| TNC Isoform 1 of Tenascin | IPI00031008 | 241 kDa | 75.166 | 36.483 | 2.060302058 |
| CD81 CD81 antigen | IPI00000190 (+1) | 26 kDa | 26.669 | 13.104 | 2.035180098 |
| NCEH1 arylacetamide deacetylase-like 1 isoform b | IPI00002230 (+2) | 49 kDa | 15.902 | 7.8216 | 2.033087859 |
| GSTM3 Glutathione S-transferase Mu 3 | IPI00246975 | 27 kDa | 40.993 | 20.37 | 2.012420226 |
| HIST1H4K;HIST1H4F;HIST1H4I;HIST2H4A;HIST1H4A;HIST1H4C;HIST2H4B;HIST1H4L;HIST | IPI00453473 | 11 kDa | 23.906 | 11.923 | 2.005032291 |
| FABP5L9;FABP5L2;FABP5L7;FABP5 Fatty acid-binding protein, epidermal | IPI00007797 | 15 kDa | 64.753 | 32.968 | 1.964116719 |
| ISOC1 Isochorismatase domain-containing protein 1 | IPI00304082 (+1) | 32 kDa | 10.966 | 5.5889 | 1.962103455 |
| CAPZA1 F-actin-capping protein subunit alpha-1 | IPI00005969 (+1) | 33 kDa | 14.965 | 7.7255 | 1.93709145 |
| AHNAK Neuroblast differentiation-associated protein AHNAK | IPI00021812 | 629 kDa | 385.71 | 199.85 | 1.929997498 |
| S100A1 Protein S100-A1 | IPI00645016 | 11 kDa | 37.023 | 19.232 | 1.925072795 |
| BCAN Isoform 1 of Brevican core protein | IPI00456623 | 99 kDa | 38.985 | 20.712 | 1.882242178 |
| PLEC Isoform 3 of Plectin-1 | IPI00398002 | 518 kDa | 913.08 | 487.52 | 1.872907778 |
| BCAS1 Isoform 2 of Breast carcinoma-amplified sequence 1 | IPI00744780 | 66 kDa | 30.913 | 16.774 | 1.842911649 |
| NME1-NME2;NME1;NME2 Nucleoside diphosphate kinase | IPI00604590 (+1) | 33 kDa | 17.052 | 9.2967 | 1.834199232 |
| VCAN Isoform V0 of Versican core protein | IPI00009802 (+4) | 373 kDa | 249.8 | 136.64 | 1.828161593 |
| ALDH9A1 aldehyde dehydrogenase 9A1 | IPI00479877 | 56 kDa | 25.653 | 14.051 | 1.825706355 |
| NEFH Isoform 1 of Neurofilament heavy polypeptide | IPI00910602 | 112 kDa | 122.44 | 67.624 | 1.810599787 |
| XPNPEP1 X-Pro aminopeptidase 1, soluble isoform 1 | IPI00793375 | 75 kDa | 10.389 | 5.7638 | 1.802456713 |
| GNAQ Guanine nucleotide-binding protein G(q) subunit alpha | IPI00288947 | 42 kDa | 12.706 | 7.0719 | 1.796688302 |
| ALDH7A1 aldehyde dehydrogenase 7 family, member A1 | IPI00221234 (+1) | 58 kDa | 112.81 | 63.288 | 1.782486411 |
| HMGB1 High mobility group protein B1 | IPI00419258 (+4) | 25 kDa | 31.41 | 17.657 | 1.778897888 |
| CNDP2 Isoform 1 of Cytosolic non-specific dipeptidase | IPI00177728 | 53 kDa | 109.58 | 61.62 | 1.778318728 |
| SNCG Gamma-synuclein | IPI00297714 | 13 kDa | 34.835 | 19.729 | 1.765674895 |
| GCSH Glycine cleavage system H protein, mitochondrial | IPI00011604 | 19 kDa | 15.716 | 8.9383 | 1.758276182 |
| IQGAP1 Ras GTPase-activating-like protein IQGAP1 | IPI00009342 | 189 kDa | 14.511 | 8.2667 | 1.755355825 |
| CDC37 Hsp90 co-chaperone Cdc37 | IPI00013122 | 44 kDa | 27.008 | 15.492 | 1.743351407 |
| MAP4 Isoform 1 of Microtubule-associated protein 4 | IPI00396171 (+2) | 121 kDa | 44.175 | 25.459 | 1.735142779 |
| PDIA3 Protein disulfide-isomerase A3 | IPI00025252 | 57 kDa | 17.127 | 10 | 1.7127 |
| FABP3 Fatty acid-binding protein, heart | IPI00219684 | 15 kDa | 9.4686 | 5.5477 | 1.706761361 |
| KBTBD11 Kelch repeat and BTB domain-containing protein 11 | IPI00015568 | 66 kDa | 13.806 | 8.0946 | 1.705581499 |
| EPB41L2 Band 4.1-like protein 2 | IPI00015973 | 113 kDa | 27.991 | 16.42 | 1.704689403 |
| LOC442497;SLC3A2 Isoform 2 of 4F2 cell-surface antigen heavy chain | IPI00027493 (+5) | 58 kDa | 24.474 | 14.557 | 1.681253005 |
| MBP Isoform 3 of Myelin basic protein | IPI00216475 | 21 kDa | 375.98 | 224.54 | 1.674445533 |
| CAP1 Isoform 1 of Adenylyl cyclase-associated protein 1 | IPI00008274 | 52 kDa | 47.379 | 28.357 | 1.670804387 |
| SLC9A3R1 Na(+)/H(+) exchange regulatory cofactor NHE-RF1 | IPI00003527 | 39 kDa | 28.284 | 16.936 | 1.67005196 |
| INA Alpha-internexin | IPI00001453 | 55 kDa | 258.73 | 155.62 | 1.662575504 |
| C8orf62 Phosphoserine aminotransferase | IPI00001734 (+2) | 45 kDa | 11.175 | 6.7733 | 1.649860482 |
| AKAP12 Isoform 1 of A-kinase anchor protein 12 | IPI00237884 | 191 kDa | 47.28 | 28.825 | 1.640242845 |
| GMFB Glia maturation factor, beta | IPI00412987 (+1) | 18 kDa | 30.823 | 18.792 | 1.640219242 |
| HIST1H2AA Histone H2A type 1-A | IPI00045109 (+2) | 14 kDa | 80.693 | 49.662 | 1.624843945 |
| TLN1 Talin-1 | IPI00298994 | 270 kDa | 117.81 | 72.54 | 1.624069479 |

| Gene/Protein description | IPI accession number | Molecular weight | Spectral counts 'AD' | Spectral counts 'Control' | Fold-change |
|---|---|---|---|---|---|
| G6PD Isoform Long of Glucose-6-phosphate 1-dehydrogenase | IPI00216008 (+2) | 64 kDa | 12.759 | 7.8952 | 1.616045192 |
| ENO1 Isoform alpha-enolase of Alpha-enolase | IPI00465248 | 47 kDa | 541.48 | 335.47 | 1.61409366 |
| RAB11B Ras-related protein Rab-11B | IPI00020436 | 24 kDa | 8.5361 | 5.3492 | 1.59577133 |
| CISD1 CDGSH iron sulfur domain-containing protein 1 | IPI00020510 | 12 kDa | 16.973 | 10.65 | 1.59370892 |
| SCCPDH Probable saccharopine dehydrogenase | IPI00329600 | 47 kDa | 10.053 | 6.3246 | 1.589507637 |
| NEFL Neurofilament light polypeptide | IPI00237671 | 62 kDa | 283.44 | 179.21 | 1.581608169 |
| TUBB2C Tubulin beta-2C chain | IPI00007752 | 50 kDa | 319.9 | 203.07 | 1.575318856 |
| GDI1 Rab GDP dissociation inhibitor alpha | IPI00010154 | 51 kDa | 518.42 | 329.49 | 1.573401317 |
| GDI2 Rab GDP dissociation inhibitor beta | IPI00940148 | 51 kDa | 131.53 | 83.747 | 1.570563722 |
| CTNNB1 Isoform 1 of Catenin beta-1 | IPI00017292 (+2) | 85 kDa | 17.679 | 11.261 | 1.569931622 |
| SELENBP1 Selenium binding protein 1 | IPI00012303 (+1) | 57 kDa | 95.446 | 61.039 | 1.563688789 |
| RPL12 Isoform 1 of 60S ribosomal protein L12 | IPI00024933 | 18 kDa | 17.055 | 10.935 | 1.559670782 |
| DDAH1 N(G),N(G)-dimethylarginine dimethylaminohydrolase 1 | IPI00220342 | 31 kDa | 133.82 | 86.146 | 1.553409328 |
| GNB2 Guanine nucleotide-binding protein G(I)/G(S)/G(T) subunit beta-2 | IPI00003348 | 37 kDa | 31.728 | 20.56 | 1.543190661 |
| SLC25A11 Mitochondrial 2-oxoglutarate/malate carrier protein | IPI00219729 (+2) | 34 kDa | 8.3692 | 5.4564 | 1.533831831 |
| LDHB L-lactate dehydrogenase B chain | IPI00219217 | 37 kDa | 319.56 | 209.71 | 1.523818607 |
| SIRT2 Isoform 1 of NAD-dependent deacetylase sirtuin-2 | IPI00179109 | 43 kDa | 78.08 | 51.267 | 1.523007003 |
| ANXA6 Annexin A6 | IPI00221226 | 76 kDa | 108.89 | 71.738 | 1.517884524 |
| ILF2 Interleukin enhancer-binding factor 2 | IPI00005198 | 43 kDa | 11.589 | 7.6371 | 1.517460816 |
