# Supplementary material for: Semiquantitative proteomic analysis of human hippocampal tissues from Alzheimer’s disease and age-matched control brains
Source: Clin Proteomics. 2013 May 1;10(1):5. doi: 10.1186/1559-0275-10-5 (PMC3648498; doi:10.1186/1559-0275-10-5)
Supplement: Additional file 6 — Up-regulated proteins in ‘Control’ tissues in comparison to Alzheimer's (AD) tissues. [file 1559-0275-10-5-S6.pdf]

Additional table 6: Upregulated proteins in 'Control' tissues in comparison to Alzheimer's (AD) tissues. A cut-off of 1.5-fold change and a minimum of 2-peptide hit identification was used.

Gene/Protein description	IPI accession number	Molecular weight	Spectral counts 'AD'	Spectral counts 'Control'	Fold-change
EEF1A2 Elongation factor 1-alpha 2	IPI00014424	50 kDa	5.0608	37.246	7.359705975
OGDH 2-oxoglutarate dehydrogenase, mitochondrial	IPI00098902	116 kDa	10.106	47.946	4.744310311
IGSF8 Isoform 1 of Immunoglobulin superfamily member 8	IPI00056478 (+1)	65 kDa	5.654	26.058	4.60877255
ACTR2 Actin-related protein 2	IPI00005159 (+1)	45 kDa	9.9258	44.048	4.437727941
IGHV4-31;LOC100294459;IGHG1;LOC100290320 Putative uncharacterized protein	IPI00930124	52 kDa	17.548	76.613	4.365910645
DCTN1 137 kDa protein	IPI00916757 (+1)	137 kDa	8.5997	37.287	4.335848925
RPLP2 60S acidic ribosomal protein P2	IPI00008529	12 kDa	10.244	43.402	4.236821554
PAFAH1B1 Isoform 1 of Platelet-activating factor acetylhydrolase IB subunit alpha	IPI00218728 (+1)	47 kDa	5.7947	22.782	3.931523634
SPTBN2 Isoform 1 of Spectrin beta chain, brain 2	IPI00012645	271 kDa	33.94	131.5	3.874484384
TF Serotransferrin	IPI00022463	77 kDa	8.5466	32.616	3.816254417
PDCD6IP Programmed cell death 6-interacting protein	IPI00246058 (+1)	96 kDa	5.1036	19.277	3.777137707
ATP1B2 Sodium/potassium-transporting ATPase subunit beta-2	IPI00293971	33 kDa	5.2065	19.089	3.666378565
IDH3A Isoform 1 of Isocitrate dehydrogenase [NAD] subunit alpha, mitochondrial	IPI00030702	40 kDa	10.633	38.326	3.604439011
DLAT Dihydrolipoyllysine-residue acetyltransferase component of pyruvate dehydrogenase complex, mitochondrial	IPI00021338	69 kDa	12.357	42.377	3.429392247
PREPL Isoform 1 of Prolyl endopeptidase-like	IPI00442171 (+1)	84 kDa	9.5902	32.794	3.419532439
OXCT1 Succinyl-CoA:3-ketoacid-coenzyme A transferase 1, mitochondrial	IPI00026516	56 kDa	11.1	37.515	3.37972973
SNAP91 Isoform 1 of Clathrin coat assembly protein AP180	IPI00006612	93 kDa	20.351	68.682	3.374871014
GOT2 Aspartate aminotransferase, mitochondrial	IPI00018206	47 kDa	16.189	54.632	3.374637099
CAMKV Isoform 3 of CaM kinase-like vesicle-associated protein	IPI00304600	52 kDa	26.257	87.255	3.323113836
ATP2B1 Isoform D of Plasma membrane calcium-transporting ATPase 1	IPI00021695 (+5)	139 kDa	7.0309	23.14	3.29118605
DBNL Isoform 1 of Drebrin-like protein	IPI00456925	48 kDa	5.5651	17.772	3.193473612
PCCA cDNA FLJ56469, highly similar to Propionyl-CoA carboxylase alpha chain,	IPI00552419 (+2)	75 kDa	8.1638	25.824	3.163232808
GPM6A Neuronal membrane glycoprotein M6-a	IPI00019952 (+1)	31 kDa	7.9086	24.322	3.075386288
ARPC1A Actin-related protein 2/3 complex subunit 1A	IPI00333068	42 kDa	9.6747	29.71	3.070896255
CKMT1B;CKMT1A Isoform 1 of Creatine kinase U-type, mitochondrial	IPI00658109	47 kDa	19.194	58.2	3.032197562
OPA1 Isoform 1 of Dynamin-like 120 kDa protein, mitochondrial	IPI00006721 (+3)	112 kDa	18.572	55.558	2.991492569
ALDH6A1 Methylmalonate-semialdehyde dehydrogenase [acylating], mitochondrial	IPI00024990	58 kDa	15.276	45.187	2.958038754
MARCKSL1 MARCKS-related protein	IPI00641181	20 kDa	21.082	61.08	2.897258325
PDHB Isoform 1 of Pyruvate dehydrogenase E1 component subunit beta, mitochondrial	IPI00003925 (+1)	39 kDa	8.4759	23.994	2.830849821
ATP2B2 Isoform WB of Plasma membrane calcium-transporting ATPase 2	IPI00009791 (+5)	137 kDa	23.032	64.716	2.809829802
DNM1L Isoform 1 of Dynamin-1-like protein	IPI00146935 (+2)	82 kDa	39.922	110.85	2.776664496
IDH2 Isocitrate dehydrogenase [NADP], mitochondrial	IPI00011107	51 kDa	30.129	83.298	2.76471174
NDUFS1 NADH-ubiquinone oxidoreductase 75 kDa subunit	IPI00604664 (+2)	81 kDa	22.776	62.848	2.759395855
PPM1H Protein phosphatase 1H	IPI00736251	56 kDa	7.2234	19.32	2.674640751
DNM1 Isoform 4 of Dynamin-1	IPI00888758	96 kDa	23.112	61.691	2.669219453
PPP2CA Serine/threonine-protein phosphatase 2A catalytic subunit alpha isoform	IPI00008380	36 kDa	15.204	40.57	2.668376743
ATL1 Atlastin-1	IPI00103530	64 kDa	6.8906	18.172	2.637215917
SH3BGRL2 SH3 domain-binding glutamic acid-rich-like protein 2	IPI00412272 (+1)	12 kDa	8.2567	21.609	2.617147286
RHOA Transforming protein RhoA	IPI00478231	22 kDa	13.697	35.548	2.595312842

Gene/Protein description	IPI accession number	Molecular weight	Spectral counts 'AD'	Spectral counts 'Control'	Fold-change
PTPN11 Isoform 2 of Tyrosine-protein phosphatase non-receptor type 11	IPI00298347 (+1)	68 kDa	9.6955	25.084	2.587179619
ATP8A1 Isoform Long of Probable phospholipid-transporting ATPase IA	IPI00032402 (+3)	131 kDa	6.235	16.011	2.567923015
PPP3R1 Calcineurin subunit B type 1	IPI00027464 (+2)	19 kDa	5.4993	14.109	2.565599258
PGM2L1 Glucose 1,6-bisphosphate synthase	IPI00173346	70 kDa	25.224	64.608	2.561370124
MACF1 Isoform 2 of Microtubule-actin cross-linking factor 1, isoforms 1/2/3/5	IPI00256861 (+1)	620 kDa	14.951	37.233	2.490335095
RPL4 60S ribosomal protein L4	IPI00003918	48 kDa	5.9516	14.794	2.485718126
CCT3 chaperonin containing TCP1, subunit 3 isoform b	IPI00290770 (+1)	60 kDa	10.06	24.98	2.483101392
SYN2 Isoform IIa of Synapsin-2	IPI00023302	63 kDa	53.652	132.76	2.474465071
STXBP1 Isoform 1 of Syntaxin-binding protein 1	IPI00084828	68 kDa	141.08	342.14	2.425148852
VDAC2 Isoform 2 of Voltage-dependent anion-selective channel protein 2	IPI00024145 (+4)	30 kDa	8.9735	21.444	2.389703014
HNRNPU Isoform Long of Heterogeneous nuclear ribonucleoprotein U	IPI00883857	91 kDa	6.9826	16.626	2.381061496
KPNB1 Importin subunit beta-1	IPI00001639	97 kDa	12.421	29.47	2.372594799
APOA1 Apolipoprotein A-I	IPI00021841	31 kDa	7.9276	18.635	2.350648368
CAMK2A calcium/calmodulin-dependent protein kinase II alpha isoform 1	IPI00215715 (+1)	55 kDa	27.213	63.734	2.342042406
HSD17B10 Isoform 1 of 3-hydroxyacyl-CoA dehydrogenase type-2	IPI00017726	27 kDa	8.9356	20.746	2.321724339
SNAP25 Isoform SNAP-25b of Synaptosomal-associated protein 25	IPI00010470	23 kDa	12.676	29.347	2.315162512
NDUFA9 NADH dehydrogenase [ubiquinone] 1 alpha subcomplex subunit 9, mitochondrial	IPI00003968	43 kDa	8.7815	20.287	2.310197574
STX1A Isoform 1 of Syntaxin-1A	IPI00003370	33 kDa	25.576	59.02	2.307632155
NAPA Alpha-soluble NSF attachment protein	IPI00009253	33 kDa	21.63	49.907	2.307304669
NCDN Isoform 1 of Neurochondrin	IPI00549543 (+2)	79 kDa	23.454	53.558	2.283533726
LANCL1 LanC-like protein 1	IPI00005724	45 kDa	15.929	36.331	2.280808588
GLUL Glutamine synthetase	IPI00010130	42 kDa	18.911	43.033	2.27555391
HSPA12A Heat shock 70 kDa protein 12A	IPI00011932	75 kDa	11.199	25.1	2.241271542
ATP2A2 Isoform SERCA2B of Sarcoplasmic/endoplasmic reticulum calcium ATPase 2	IPI00219078	115 kDa	15.172	33.878	2.23292908
KLC1 Isoform A of Kinesin light chain 1	IPI00020096 (+10)	65 kDa	5.5948	12.46	2.227067992
CA1 Carbonic anhydrase 1	IPI00215983	29 kDa	14.083	31.182	2.214158915
PFKP 6-phosphofructokinase type C	IPI00009790	86 kDa	13.736	30.381	2.211779266
CYFIP2 Isoform 2 of Cytoplasmic FMR1-interacting protein 2	IPI00719600 (+1)	146 kDa	15.504	33.945	2.189434985
TUBA4A Tubulin alpha-4A chain	IPI00007750 (+1)	50 kDa	27.764	60.653	2.184591557
HSPA4 Heat shock 70 kDa protein 4	IPI00002966	94 kDa	24.432	53.318	2.182301899
PRKAR2B cAMP-dependent protein kinase type II-beta regulatory subunit	IPI00554752	46 kDa	14.845	32.233	2.171303469
HYOU1 Hypoxia up-regulated protein 1	IPI00000877	111 kDa	10.925	23.225	2.125858124
STX1B Syntaxin-1B	IPI00410675 (+1)	33 kDa	46.637	99.055	2.123957373
HSD17B12 Estradiol 17-beta-dehydrogenase 12	IPI00007676 (+1)	34 kDa	7.2579	15.323	2.111216743
STRAP cDNA FLJ51909, highly similar to Serine-threonine kinase receptor-associatedprotein	IPI00294536 (+1)	40 kDa	9.5539	20.121	2.106050932
YWHAH 14-3-3 protein eta	IPI00216319	28 kDa	13.833	29.109	2.104315767
ICAM5 intercellular adhesion molecule 5 precursor	IPI00743302	97 kDa	15.699	33.01	2.102681699
PRKCA Protein kinase C alpha type	IPI00385449	77 kDa	5.0032	10.3	2.058682443
PCSK1N ProSAAS	IPI00002280	27 kDa	8.3002	17.024	2.051034915
MAP6 Isoform 1 of Microtubule-associated protein 6	IPI00420071	87 kDa	6.9186	14.183	2.04998121
CRMP1 collapsin response mediator protein 1 isoform 1	IPI00556376	74 kDa	83.701	170.99	2.042866871
MDH2 Malate dehydrogenase, mitochondrial	IPI00291006	36 kDa	107.91	220.25	2.041052729

Gene/Protein description	IPI accession number	Molecular weight	Spectral counts 'AD'	Spectral counts 'Control'	Fold-change
PRKCG cDNA FLJ60619, highly similar to Protein kinase C gamma type	IPI00007128	80 kDa	5.5299	11.282	2.040181558
SYNJ1 Isoform 1 of Synaptojanin-1	IPI00012441 (+3)	173 kDa	25.487	51.967	2.038961039
PDXP Pyridoxal phosphate phosphatase	IPI00025340	32 kDa	15.235	30.976	2.033212996
HINT1 Histidine triad nucleotide-binding protein 1	IPI00239077	14 kDa	8.7831	17.794	2.025936173
PFKM Isoform 2 of 6-phosphofructokinase, muscle type	IPI00219585 (+2)	82 kDa	47.591	96.247	2.022378181
CCT7 T-complex protein 1 subunit eta	IPI00018465	59 kDa	11.828	23.885	2.019360839
KTN1 Isoform 1 of Kinectin	IPI00328753 (+2)	156 kDa	5.5769	11.255	2.018146282
AP1B1 Isoform A of AP-1 complex subunit beta-1	IPI00328257 (+2)	105 kDa	20.242	40.812	2.016203932
ATP6V1C1 V-type proton ATPase subunit C 1	IPI00007814	44 kDa	16.075	32.28	2.008087092
RAB3A Ras-related protein Rab-3A	IPI00023504	25 kDa	30.516	61.23	2.0064884
AP2A1 Isoform B of AP-2 complex subunit alpha-1	IPI00256684 (+1)	105 kDa	42.936	85.778	1.997810695
NFASC Isoform 4 of Neurofascin	IPI00394655 (+2)	119 kDa	14.156	27.903	1.971107658
YWHAQ 14-3-3 protein theta	IPI00018146	28 kDa	35.666	69.964	1.961644143
CCT8 T-complex protein 1 subunit theta	IPI00784090	60 kDa	15.431	30.217	1.958201024
LANCL2 LanC-like protein 2	IPI00032995	51 kDa	12.197	23.84	1.954578995
USP14 Ubiquitin carboxyl-terminal hydrolase 14	IPI00219913	56 kDa	10.58	20.608	1.947826087
RTN1 Isoform RTN1-A of Reticulon-1	IPI00003971	84 kDa	25.513	49.374	1.935248697
YWHAG 14-3-3 protein gamma	IPI00220642	28 kDa	28.403	54.945	1.934478752
C3 Complement C3 (Fragment)	IPI00783987	187 kDa	33.486	64.735	1.933195962
NAPB Beta-soluble NSF attachment protein	IPI00748037	34 kDa	66.541	127.41	1.914759321
SKP1 Isoform 1 of S-phase kinase-associated protein 1	IPI00301364	19 kDa	24.075	45.935	1.907995846
ATP5O ATP synthase subunit O, mitochondrial	IPI00007611	23 kDa	9.2338	17.539	1.899434686
TUBB Tubulin, beta	IPI00645452	48 kDa	48.21	91.176	1.891225887
CCT6A T-complex protein 1 subunit zeta	IPI00027626	58 kDa	14.974	28.219	1.884533191
GAP43 growth associated protein 43 isoform 1	IPI00791316	29 kDa	108.32	204.1	1.884231905
CCT2 T-complex protein 1 subunit beta	IPI00297779	57 kDa	16.812	31.556	1.876992624
PNPO Pyridoxine-5'-phosphate oxidase	IPI00018272	30 kDa	6.0192	11.275	1.873172515
ETFA Electron transfer flavoprotein subunit alpha, mitochondrial	IPI00010810	35 kDa	20.657	38.288	1.853512127
ATP6V1H Isoform 1 of V-type proton ATPase subunit H	IPI00296191 (+1)	56 kDa	16.288	30.177	1.852713654
PACSIN1 Protein kinase C and casein kinase substrate in neurons protein 1	IPI00011515	51 kDa	28.817	53.282	1.848978034
ALB Isoform 1 of Serum albumin	IPI00745872	69 kDa	207.8	383.22	1.844177093
ATP6V1E1 V-type proton ATPase subunit E 1	IPI00003856	26 kDa	23.646	43.264	1.829654064
AP2B1 Isoform 1 of AP-2 complex subunit beta	IPI00784156 (+1)	105 kDa	110.2	201.57	1.829128857
RAP1GDS1 Isoform 1 of Rap1 GTPase-GDP dissociation stimulator 1	IPI00607591 (+1)	66 kDa	13.584	24.836	1.828327444
A2M Alpha-2-macroglobulin	IPI00478003	163 kDa	29.683	54.244	1.827443318
HNRNPL Heterogeneous nuclear ribonucleoprotein L	IPI00027834	64 kDa	11.341	20.707	1.825853099
ACAT1 Acetyl-CoA acetyltransferase, mitochondrial	IPI00030363	45 kDa	68.137	123.99	1.81971616
CAPZA2 F-actin-capping protein subunit alpha-2	IPI00026182	33 kDa	10.903	19.298	1.769971567
PLCB1 Isoform A of 1-phosphatidylinositol-4,5-bisphosphate phosphodiesterase beta-1	IPI00219563	139 kDa	27.372	48.446	1.769910858
SLC1A2 Isoform 1 of Excitatory amino acid transporter 2	IPI00300020 (+1)	62 kDa	103.1	182.07	1.765955383
TAGLN2 Transgelin-2	IPI00550363 (+2)	22 kDa	15.885	27.773	1.748378974
FASN Fatty acid synthase	IPI00026781	273 kDa	65.592	114.09	1.73938895
DLD Dihydrolipoyl dehydrogenase, mitochondrial	IPI00015911	54 kDa	37.934	65.307	1.721595403
MYO5A myosin VA isoform 2	IPI00873959 (+1)	212 kDa	14.794	25.43	1.718940111

Gene/Protein description	IPI accession number	Molecular weight	Spectral counts 'AD'	Spectral counts 'Control'	Fold-change
CYCS Cytochrome c	IPI00465315 (+1)	12 kDa	6.7255	11.476	1.706341536
MYH10 Isoform 3 of Myosin-10	IPI00790503	231 kDa	20.484	34.867	1.702157782
PPP3CA Isoform 1 of Serine/threonine-protein phosphatase 2B catalytic subunit alpha isoform	IPI00179415	59 kDa	19.495	33.17	1.701461913
GNB2L1 Guanine nucleotide-binding protein subunit beta-2-like 1	IPI00848226	35 kDa	23.763	40.283	1.695198418
PPP1CB Serine/threonine-protein phosphatase PP1-beta catalytic subunit	IPI00218236	37 kDa	27.509	46.566	1.692755098
UQCRC2 Cytochrome b-c1 complex subunit 2, mitochondrial	IPI00305383	48 kDa	26.632	44.999	1.689659057
PPP2R2A Serine/threonine-protein phosphatase 2A 55 kDa regulatory subunit B alpha isoform	IPI00332511 (+1)	52 kDa	5.4884	9.2472	1.684862619
NPEPPS Puromycin-sensitive aminopeptidase	IPI00026216	103 kDa	56.573	95.214	1.683029007
CALR Calreticulin	IPI00020599	48 kDa	17.231	28.929	1.678892693
TUBA8 Tubulin alpha-8 chain	IPI00646909 (+2)	50 kDa	7.2701	12.166	1.673429526
ILK-2;CCT4 T-complex protein 1 subunit delta	IPI00302927 (+1)	58 kDa	15.178	25.219	1.661549611
RCTPI1;TPI1 triosephosphate isomerase 1 isoform 2	IPI00465028 (+1)	31 kDa	59.122	98.021	1.657944589
PPP5C Serine/threonine-protein phosphatase 5	IPI00019812	57 kDa	6.4107	10.385	1.6199479
HSPD1 60 kDa heat shock protein, mitochondrial	IPI00784154	61 kDa	211.65	341.19	1.612048193
MAP2 Isoform 1 of Microtubule-associated protein 2	IPI00003842 (+1)	200 kDa	166.29	267.92	1.611161224
HK1 Isoform 1 of Hexokinase-1	IPI00018246 (+4)	102 kDa	109.19	173.87	1.592361938
CPNE6 cDNA FLJ55997, highly similar to Copine-6	IPI00295469	68 kDa	10.445	16.542	1.58372427
NDUFS3 NADH dehydrogenase [ubiquinone] iron-sulfur protein 3, mitochondrial	IPI00025796	30 kDa	7.2892	11.52	1.580420348
ACOT7 Isoform 1 of Cytosolic acyl coenzyme A thioester hydrolase	IPI00010415 (+3)	42 kDa	34.015	53.479	1.572218139
ANK2 Isoform 3 of Ankyrin-2	IPI00074962	434 kDa	212.82	334.32	1.57090499
RAB2A Ras-related protein Rab-2A	IPI00031169	24 kDa	5.9272	9.2963	1.568413416
UCHL1 Ubiquitin carboxyl-terminal hydrolase isozyme L1	IPI00018352	25 kDa	159.01	248.91	1.565373247
VAT1L Synaptic vesicle membrane protein VAT-1 homolog-like	IPI00030578	46 kDa	8.7426	13.635	1.559604694
NARS Asparaginyl-tRNA synthetase, cytoplasmic	IPI00306960	63 kDa	8.5545	13.23	1.546554445
TLN2 Talin-2	IPI00219299	272 kDa	35.24	53.837	1.527724177
OLA1 Isoform 1 of Obg-like ATPase 1	IPI00290416 (+1)	45 kDa	21.019	32.035	1.524097245
UQCRB Cytochrome b-c1 complex subunit 7	IPI00220416	14 kDa	6.5587	9.9374	1.515147819
